# Shotgun proteomics of *Brassica rapa* seed proteins identifies vicilin as a major seed storage protein in the mature seed

**DOI:** 10.1371/journal.pone.0253384

**Published:** 2021-07-09

**Authors:** Mahmudur Rahman, Qi Guo, Abdul Baten, Ramil Mauleon, Amina Khatun, Lei Liu, Bronwyn J. Barkla

**Affiliations:** 1 Southern Cross Plant Science, Faculty of Science and Engineering, Southern Cross University, Lismore, New South Wales, Australia; 2 Institute of Precision Medicine & Bioinformatics, Sydney Local Health District, Royal Prince Alfred Hospital, Camperdown, New South Wales, Australia; INRA, FRANCE

## Abstract

Proteins make up a large percentage of the Brassica seed and are second only to the oil in economic importance with uses for both animal and human nutrition. The most abundant proteins reported in the seeds of Brassica are the seed storage proteins cruciferin and napin, belonging to the 12S globulin and 2S albumin families of proteins, respectively. To gain insight into the *Brassica rapa* seed proteome and to confirm the presence and relative quantity of proteins encoded by candidate seed storage genes in the mature seed, shotgun proteomics was carried out on protein extracts from seeds of *B*. *rapa* inbred line R-o-18. Following liquid chromatography tandem mass spectrometry, a total of 34016 spectra were mapped to 323 proteins, where 233 proteins were identified in 3 out of 4 biological replicates by at least 2 unique peptides. 2S albumin like napin seed storage proteins (SSPs), 11/12S globulin like cruciferin SSPs and 7S globulin like vicilin SSPs were identified in the samples, along with other notable proteins including oil body proteins, namely ten oleosins and two oil body-associated proteins. The identification of vicilin like proteins in the mature *B*. *rapa* seed represents the first account of these proteins in the Brassicaceae and analysis indicates high conservation of sequence motifs to other 7S vicilin-like allergenic proteins as well as conservation of major allergenic epitopes in the proteins. This study enriches our existing knowledge on rapeseed seed proteins and provides a robust foundation and rational basis for plant bioengineering of seed storage proteins.

## Introduction

It is estimated that up to a billion people worldwide, from both developing and developed nations, do not have an adequate intake of protein in their diet which leads to impaired growth and increased risk of disease [1, 2]. To overcome this deficiency there is a push to identify sustainable supplies of both animal and plant protein for human consumption in order to lower greenhouse gas emissions and improve the protein supply chain in agriculture.

Rapeseed is the second-most abundantly cultivated oilseed crop globally [3–5]. After extraction of oil, about 1.2 million tons of defatted rapeseed meal is produced globally on an annual basis. This meal contains about 35–45% protein (dry weight) bringing it close to soybean for protein value [6–8]. This protein rich by-product has a nutritional content comparable to milk casein [9], egg protein [10] and animal proteins [7], with a balanced amino acid profile high in methionine and cysteine [10], providing important sulphur containing amino acids. However, the meal is primarily used to boost the nutritional value of animal or aquaculture feed or as a soil supplementation product [11, 12], rather than as a source of proteins for human consumption. This is primarily due to the presence of proteins that may cause allergenic responses as they pass through the human digestive system [13–15].

*B*. *rapa* is the highest oil-bearing species and therefore one of the economically most important species in the tribe Brassiceae belonging to the crucifer family [16]. In contrast to allopolyploid *B*. *napus* (AC genome), *B*. *juncea* (AB genome) and *B*. *carinata* (BC genome), *B*. *rapa* is a diploid species and contains only the Brassica A genome [17, 18]. In addition to oilseed crops (subsp. *Trilocularis* and *oleifera*), *B*. *rapa* encompasses edible leafy vegetables like bok choy or pak choi (subsp. *chinensis*), bomdong (var. *glabra*), Chinese cabbage (subsp. *pekinensis*), choy sum (subsp. *parachinensis*), komatsuna (subsp. *perviridis*), napa cabbage (subsp. *pekinensis*), rapini or broccoli rabe (var. *ruvo*) and tatosi, spoon mustard or tat choy (subsp. *narinosa*); root and tuber crops like Japanese vegetable turnip (subsp. *rapa*) [19–22]. Although some seed storage proteins have been partially characterised and allergenicity studies have been carried out on a few proteins from *B*. *napus* and *S*. *alba*, seed storage proteins and their potential allergenicity have not been well explored for *B*. *rapa* [23–31].

The Brassica seed storage proteins napin and cruciferin, two of the most abundant proteins in the seed, have been identified as the most likely candidates for triggering allergies, with key epitopes identified in their sequences [15, 32–35], however evidence shows other seed proteins present in other species outside the Brassica family, namely 7S globulin type vicilins, and oil-body proteins like oleosins and non-specific lipid transfer proteins may also elicit an allergenic response [36, 37].

More information is needed on the protein sequence, structure and biochemistry of *Brassica rapa* allergens. This would allow for biotechnological approaches which could manipulate the quantities of these proteins in the seed and lower the amount of allergenic proteins while not compromising the total amount of protein. This approach has recently been carried out using CRISPR/Cas9 (clustered regularly interspaced short palindromic repeat-associated protein-9 nuclease) gene editing technology in the Brassica, *Camelina sativa* whereby deletions were generated in three of the 12 genes encoding cruciferin to successfully improve seed protein composition [38].

Recent sequencing of the genomes of *Brassica rapa* inbred lines Chifu-401 v3 [39] and R-o-18 v2.2 [40], two of the most widely cultivated genotypes of rapeseed and Canola, has provided important information on the gene families for the seed storage proteins and their similarity to known allergenic proteins [12]. In this study a proteomics approach using LC-MS/MS was carried out on single seeds of *B*. *rapa* R-o-18 to validate the presence of predicted seed storage protein-coding genes, and to catalogue the proteins in the seed which will further understanding of their functions and identify regulatory proteins that may be targeted to alter the amount of proteins of interest including those involved in oil accumulation, disease and pest sensitivity/resistance and those considered allergenic. Using this approach, we were able to identify the presence of the seed storage protein vicilin for the first time within the Brassica family.

## Materials and methods

### Seed materials

Non-genetically modified seeds of *Brassica rapa* inbred line R-o-18, were collected from the Southern Cross Plant Science seed repository. In this study, four pooled populations, each consisting of seeds from 4 different plants were used.

### Extraction of protein from seeds

Protein was extracted from seeds according to the method described in [41] with some modifications. In summary, seeds were placed in 2.0 mL PCR grade Safe Lock microcentrifuge tubes (Eppendorf AG, Germany, Lot: D15893R) with a single 5 mm stainless steel bead (Qiagen GmbH, D-40724, Hilden, Cat. No. 69989). Methanol (200 μL of 100%) was added as solvent and seeds were macerated using a ball grinder/homogeniser (TissueLyser Qiagen, Retsch GmbH, Germany) by shaking in a precooled adapter for 20 cycles per second for two min with a one min rest interval. Extracts were then centrifuged at 4°C in a Sigma 4K15 Laboratory centrifuge (Sigma Laboratory Centrifuge 4K15, Germany), at 10,000g for 20 min, followed by drying in a speed vacuum dryer (Eppendorf concentrator plus 5301 Eppendorf, USA) at 30°C under vacuum.

### TCA precipitation

Trichloroacetic acid (TCA) precipitation of the dried samples was carried out according to the protocol of Barkla et al. [42] with minor modification. In summary, 500 μl MilliQ water (Advantage A 10, Millipore, USA) was added to the dried samples and vortexed lightly. This was followed by addition of 100 μl of 10X TE (standard Tris-EDTA buffer) [43] and 100 μl of 0.3% NaDoC (Sodium deoxycholate). Finally, 100 μl of 72% TCA was added, and the samples were vortexed and incubated on ice for one hour. The samples were then centrifuged at 4°C for 20 min at 11000*g*. The supernatant was carefully aspirated, and the pellet was resuspended in 500 μl of 90% methanol. The sample was incubated in a -20°C freezer overnight. The samples were then centrifuged at 4°C for 20 min at 11000*g* and the supernatant aspirated. The pellets were air dried and then stored at -80°C.

### NanoHPLC, mass spectrometry and protein identification

Mass spectroscopic analysis was carried out on the protein samples at the Institute for Molecular Biosciences proteomics facility at the University of Queensland, Brisbane. The extracts were analysed by Shimadzu Prominence NanoHPLC- MS/MS on an Eksigent, Ekspert nano LC400 uHPLC (SCIEX, Canada) coupled to a Triple Time of Flight (TOF) 6600 mass spectrometer (SCIEX, Canada) equipped with a PicoView nanoflow (New Objective, USA) ion source. Five μL of each trypsin digested extract was injected onto a 75μm x 150mm ChromXP C18 CL 3 μm column (SCIEX, Canada) at 400 nL/min and a column temperature of 45°C. Mobile phase contained two solvents, solvent A and solvent B. Solvent A consisted of 0.1% formic acid in water and solvent B contained 0.1% formic acid in acetonitrile. Linear gradients of 5–30% solvent B were run over 120 min at 400 nL/minute flow rate, followed by a steeper gradient of 30% to 90% solvent B for 3 min, then 90% solvent B for 17 min, for peptide elution. The gradient was then returned to 5% solvent B for equilibration prior to the next sample injection. The ion spray voltage was set to 2600V, declustering potential (DP) 80V, curtain gas flow 25 psi, nebuliser gas 1 (GS1) 30 psi, interface heater at 150°C. The mass spectrometer acquired 100ms full scan TOF-MS data followed by up to fifty 50ms full scan product ion data in an Information Dependent Acquisition (IDA) mode. Full scan TOF-MS data was acquired over the mass range 350–1500 *m/z* and for product ion MS/MS, 100–1500 *m/z*. Ions observed in the TOF-MS scan exceeding a threshold of 100 counts and a charge state of +2 to +5 were set to trigger the acquisition of product ion MS/MS spectra of the resultant 50 most intense ions followed by data acquisition. Raw spectral data was submitted to the Southern Cross University research portal (https://researchportal.scu.edu.au/) and is available under 10.25918/data.133.

### Analysis of proteomic data

**Fig 1:**
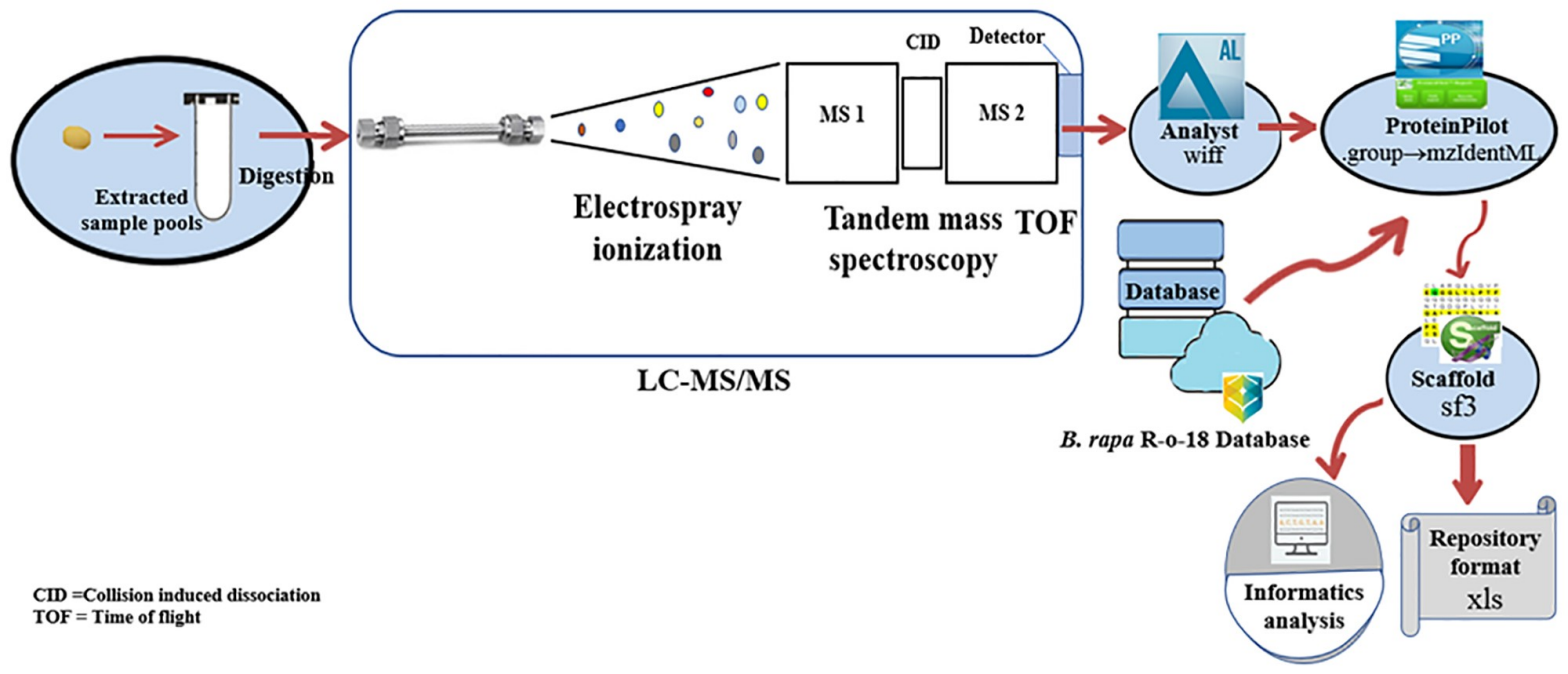
Overview graph of proteomics pipeline. The LC-MS/MS proteomics workflow is illustrated using ovals to represent the key steps within the workflow and the arrows connecting them.

Spectral data from the mass spectrometer was acquired and processed using Analyst TF 1.7 software (SCIEX, Canada). A combined database was generated from the *B*. *rapa* R-o-18 v2.2 database annotated in house at Southern Cross Plant Science, Southern Cross University [43, 245 proteins) [40], merged with previously reported sequences publicly available in the Uniprot and NCBI protein databases including (a) 1.7/2S albumin or napin (b) 11/12S globulin or cruciferin (c) 7S globulin or vicilin (d) oleosin, and (e) putative napin sequences characterised in *B*. *rapa* [15], (S1 and S2 Tables in S1 File) (f) putative napin, cruciferin and vicilin sequences characterised in *B*. *rapa* determined by performing BLASTP analysis with the corresponding cruciferin and vicilin sequences from *A*. *thaliana* [15]. ProteinPilot 5.0.2 (SCIEX, Canada) was used to search spectra against the combined database, visualize fragmentation evidence of identified peptides, process qualitative and quantitative proteomics data and encode the output mzIdentML file (Fig 1) [44]. To interpret, organize, validate and visualize the information, Scaffold 4.8.7 (Proteome Science) was used [45, 46].

### Gene ontology enrichment analysis

Proteins identified in at least 3 out of 4 biological replicates with at least 2 unique peptides were selected for downstream analysis. Sequences of identified proteins were compared against the UniprotKB *Arabidopsis thaliana* database using the Basic Local Alignment Search Tool (BLASTP). Uniprot IDs of matched Arabidopsis homologs were then submitted to DAVID v6.8 (Functional Annotation Bioinformatics Microarray Analysis database https://david.ncifcrf.gov/, 20.04.2020) [47] for functional annotation of identified proteins and Gene Ontology (GO) enrichment analysis using the hypergeometric method with Benjamini false discovery rate (FDR) correction.

### Multiple sequence alignment and phylogenetic analysis

Multiple sequence alignment was performed using Clustal Omega (https://www.ebi.ac.uk/Tools/msa/clustalo/) (S1 Table in S1 File) [48]. Aligned sequences were formatted using MView [49]. Evolutionary relationships were determined by phylogenetic analysis using the online NGPhylogeny.fr web resource (https://ngphylogeny.fr/workflows/oneclick/, 30/05/2020).

### Sequence analysis and epitope mapping and of vicilin proteins

To obtain more insight into the allergenicity of vicilin like proteins, epitope mapping was performed according to the method described in [15, 50]. In summary, previously documented vicilin proteins from diverse species representing known allergens reported in the protein databases (S1 Table in S1 File) were mapped to the sequences identified in this study (S5 Table in S1 File), to determine conservation of IgE-binding epitopes [50–52].

## Results

Mass spectrometric data was searched against a database made up of the *Brassica rapa* R-o-18 genome annotation version 2.2 comprising 43,245 protein entries [53] which was combined with putative and published sequences of seed storage and oil body proteins [15] (S1 and S2 Tables in S1 File). The data obtained were analysed with ProteinPilot 5.0.2 software (SCIEX), then visualized and validated using Scaffold 4.8.7 (Proteome Science) employing a minimum protein threshold of 99.9% and peptide threshold of 95% (S3 Table in S1 File).

**Fig 2:**
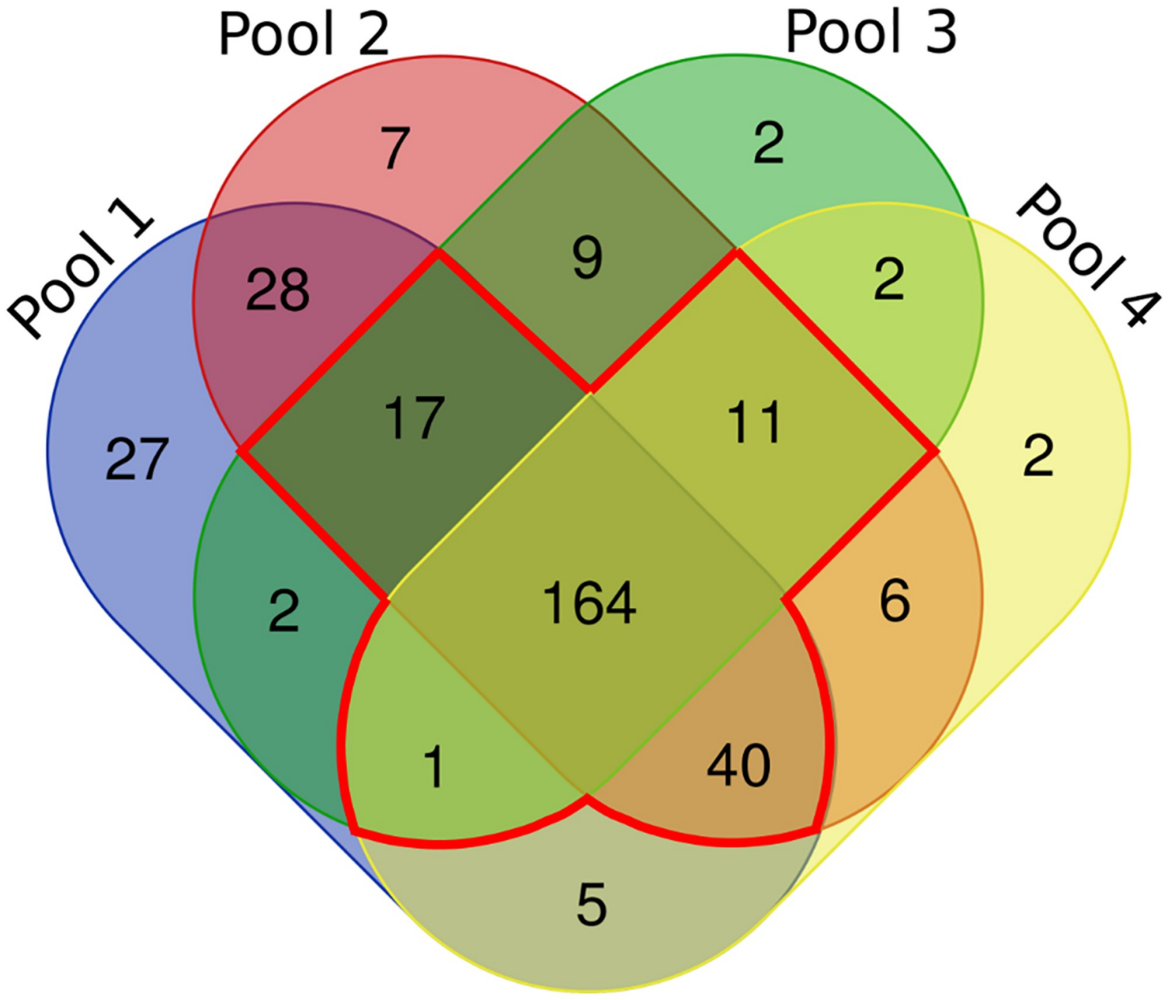
Venn diagram showing the distribution of unique and common non-redundant proteins among the four biological replicates. The overlapping regions show numbers demonstrating the proteins that were expressed in at least three biological replicates with 2 unique peptides Proteins within the red outline are those present in all biological replicates (Pool 1 to 4). The identities of the expressed proteins are detailed in S4 Table in S1 File.

A total of 34016 spectra were mapped to 323 proteins (S3 Table in S1 File). These proteins ranged in theoretical molecular weight from 7 kDa (Bra000692) to 175 kDa (Bra026064) (S4 Table in S1 File). A Venn diagram was constructed (Fig 2) to show how the proteins were distributed between the biological replicates (Pool 1 to 4). A total of 233 proteins were identified as present in at least 3 out of 4 biological replicates with at least 2 unique peptides (S4 Table in S1 File) as indicated by the numbers within the red outline (Fig 2). Of these 164 were identified in all four biological replicates as indicated by the middle square inside the red outline (Fig 2).

**Fig 3:**
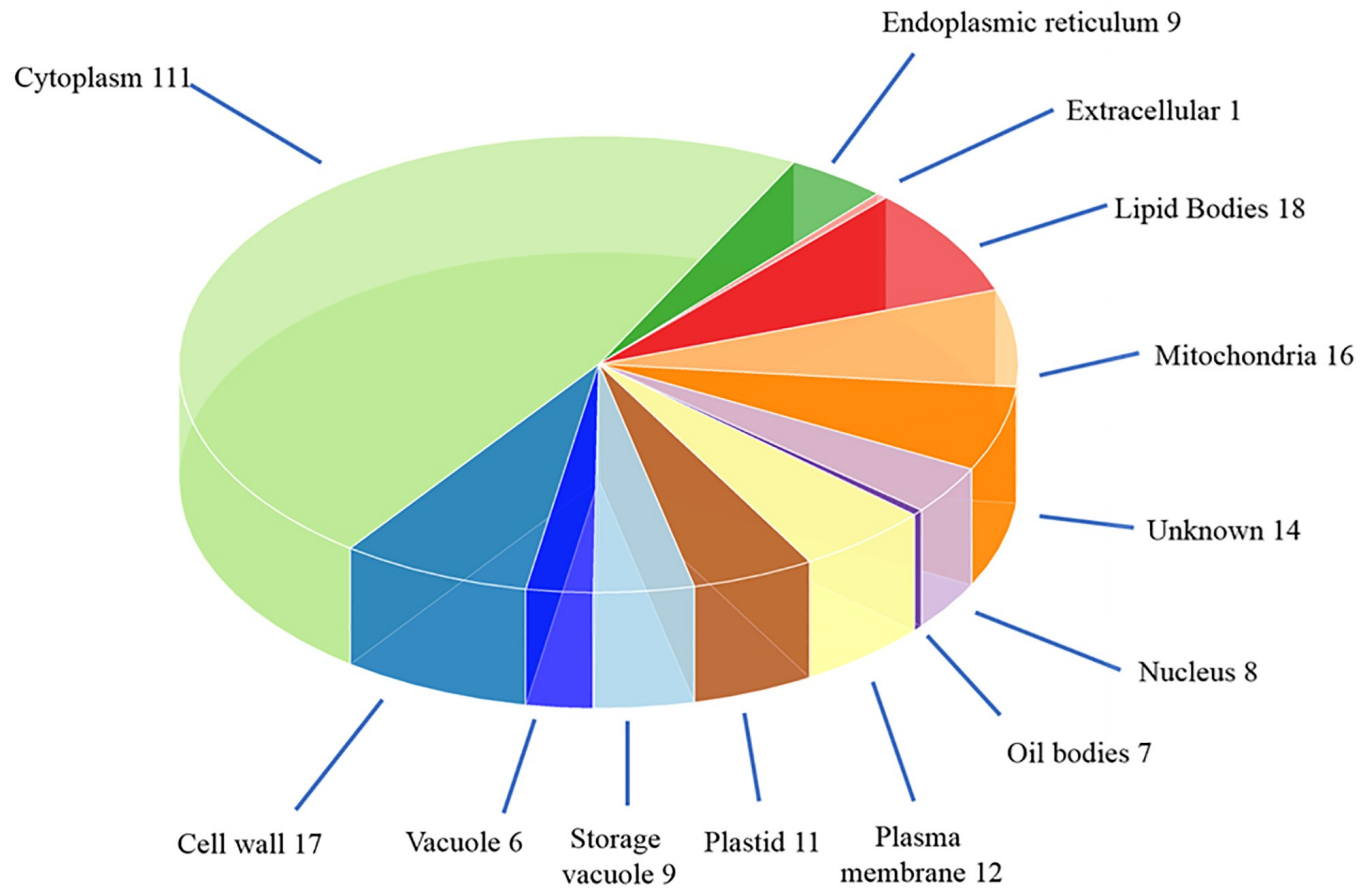
Distribution of proteins identified from *Brassica rapa* R-o-18 seed extract according to GO cellular component annotations. The proteins identified from *Brassica rapa* R-o-18 seed extract using LC-MS/MS were categorized based on Gene Ontology (GO) annotation as described in Material and Methods. Ten ‘cellular component’ categories were assigned to 219 proteins with 14 unknown proteins remaining uncategorized.

Gene ontology (GO) terms for the identified proteins were initially obtained by BLASTP search against protein sequences in the Arabidopsis database in NCBI. This was refined using manual curation taking into account both experimental evidence available in the literature for protein homologs, detailed sequence analysis, as well as verified information from the Uniprot database [54]. The characterised proteins were then grouped according to their gene ontology (GO) terms within the categories of cellular component and molecular function. Ten different cellular component categories were mapped to the identified proteins (Fig 3). The majority of the proteins identified were associated to the cytoplasm (50.6%), while 8.1% were associated to lipid/oil bodies and 6.4% to vacuoles. Fourteen of the identified proteins were unable to be assigned to any cellular component.

**Fig 4:**
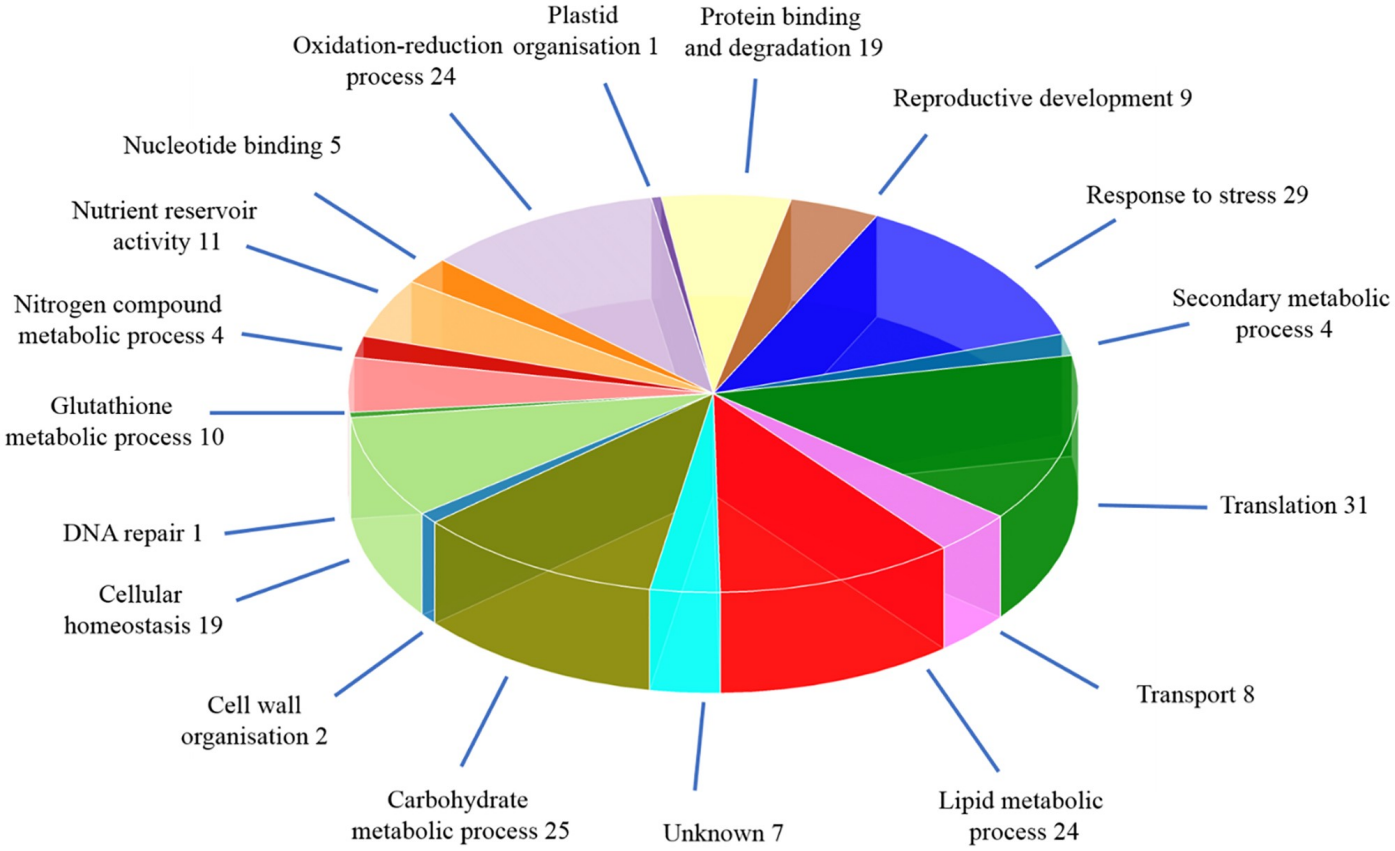
Distribution of proteins identified from *Brassica rapa* R-o-18 seed extract according to GO molecular function annotations. The proteins identified from *B*. *rapa* R-o-18 seed extract using LC-MS/MS were categorized based on Gene Ontology (GO) annotation as described in Material and Methods. Seventeen ‘molecular function’ categories were assigned to 226 proteins with 7 unknown proteins remaining uncategorized.

Identified proteins were then grouped according to their molecular function [55], and the percent contribution of proteins to each functional group compared in Fig 4. The majority of these were directly linked to cellular metabolic processes, including carbohydrate and lipid metabolic processes, as well as cellular homeostasis. There was also a considerable grouping of proteins in the category of response to stress and translation. Proteins grouped in the nutrient reservoir category were all classified as seed storage proteins (SSP), representing 4.72% of all non-redundant proteins identified. This group was made up of 2S albumin like napins, 11/12S globulin-like cruciferins and 7S globulin like vicilins [56]. In addition to the napins Bra041165, BraA03000889 and BraA01001883, all previously predicted *Brassica rapa* napin gene products [15] were found to have high percent identity and coverage with the napins identified by LC-MS/MS at the protein level, and the percent identity and coverage for these are reported in S8 Table in S1 File. In total, seed storage proteins made up on average 20.3% of the total spectra identified, with 14.8% of the spectra associated to cruciferins, and 2.6% associated with napins. Another 2.8% of the seed storage protein spectra were associated with 7S-globulin like vicilins, suggesting that these proteins are also a major seed storage protein in the seeds of *Brassica rapa*.

As expected for an oil seed crop, a large number of oil/lipid body proteins, namely oleosins and oil-body associated proteins as well as lipid transfer proteins were identified (Fig 3 and S5 Table in S1 File) [57–59]. Oleosins and other oil body associated proteins accounted for 19.8 and 1.3%, respectively of the total spectra in the seed.

**Fig 5:**
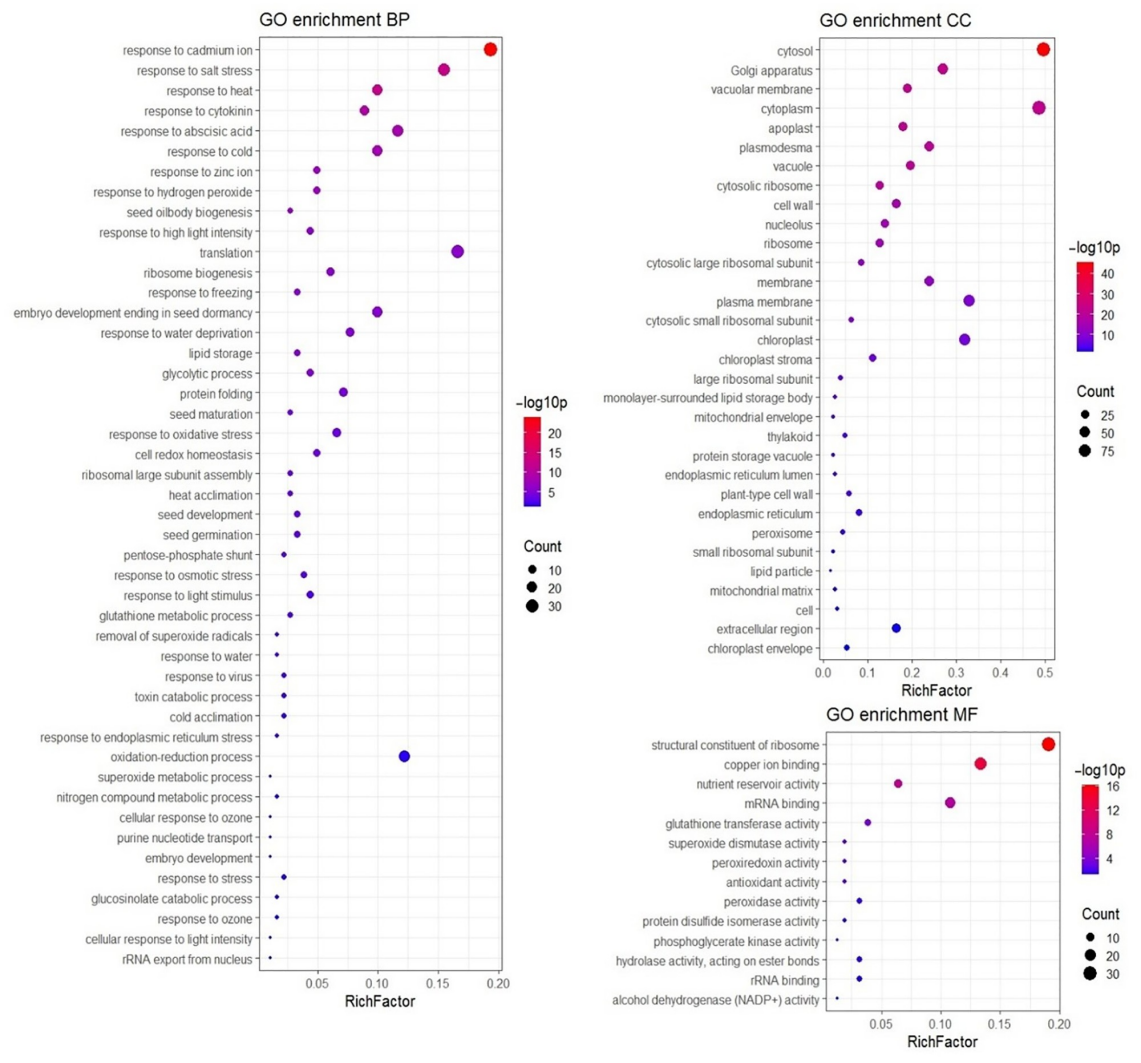
Gene Ontology (GO) term enrichment analysis of identified *Brassica rapa* seed proteins by DAVID. Gene Ontology (GO) term enrichment analysis was carried out using the hypergeometric method with Benjamini false discovery rate (FDR) correction. MF, CC and BP represents molecular functions, cellular components and biological processes, respectively. Rich factor is the ratio of the number of identified proteins annotated in the given GO term pathway to the number of all proteins from the database annotated in the pathway.

Gene Ontology (GO) term enrichment analysis using the DAVID functional annotation tool, was undertaken to test the over-representation of GO terms within the list of identified proteins compared to the natural abundance in the reference background Arabidopsis dataset to gain insight into their biological significance in the seed proteome. A total of 181, 189, and 157 proteins from the identified proteome were assigned to a biological process, cellular component, or molecular function GO annotation term, respectively. GO enrichment of molecular function identified 14 categories showing enrichment (Fig 5) with the most significantly enriched in our dataset being structural constituent of ribosome (*p*-value: 6.4E-17), copper ion binding (*p*-value: 2.2E-14), nutrient reservoir activity (*p*-value: 0.000000011), mRNA binding (*p*-value: 0.000000061) and glutathione transferase activity (*p*-value: 0.00019). The enriched GO terms associated with biological processes totaled 46 (Fig 5B), with those relating to stress responses showing the most significant enrichment compared to the background dataset, including response to cadmium ion (*p*-value: 1.10E-24), response to salt stress (*p*-value: 2.1E-13), response to heat (*p*-value: 3E-13), response to cytokinin (*p*-value: 3.3E-10), response to abscisic acid (*p*-value: 1.8E-09); and response to cold (*p*-value: 5.9E-09). Translation and oxidation-reduction processes were also enriched significantly (*p*-value: 0.0000013 and *p*-value: 0.019, respectively). Cellular component GO terms significantly enriched totaled 32 (Fig 5), and included the most enriched related terms of cytosol (*p*-value: 2.2E-46), and cytoplasm (*p*-value: 1.5E-22) as well as membrane and plasma membrane (*p*-value: 1.6E-12 and 6.6E-10, respectively).

**Fig 6:**
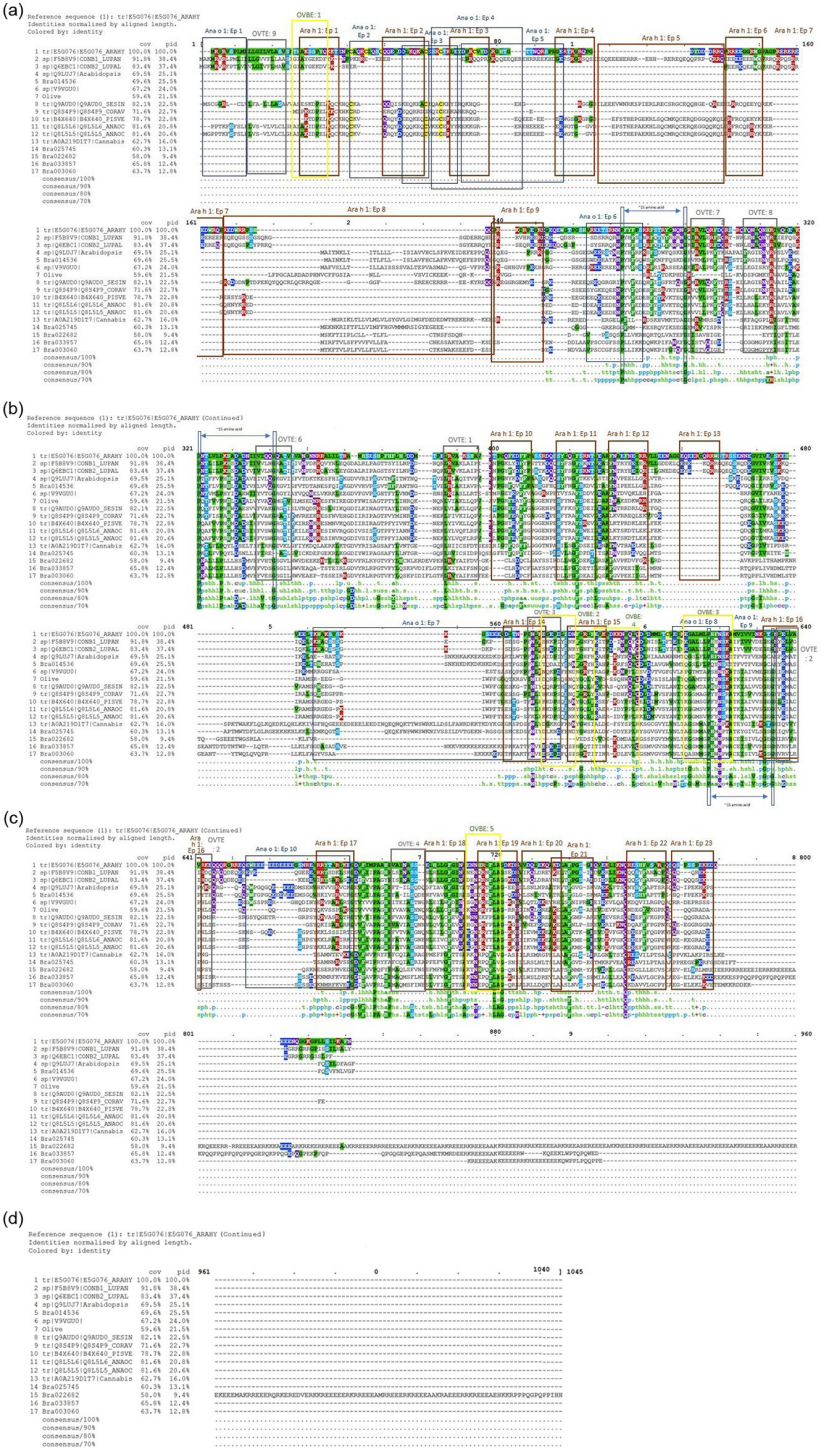
Multiple sequence alignment of the 7S globulin-like vicilin protein sequences identified in *Brassica rapa*. 7S globulin-like vicilin protein sequences from *B*. *rapa* R-o-18 seed proteome were aligned with sequences from *Arabidopsis thaliana*, *Cannabis sativa*, cashew (*Anacardium occidental*), narrow-leaved blue lupine (*Lupinus angustifolius*), *w*hite lupine (*Lupinus albus*), sesame (*Sesamum indicum*), pistachio (*Pistacia vera*), European hazel (*Corylus avellana*) and peanut (*Arachis hypogaea*) available in public domain databases. Ep = epitopes. The orange boxes delineate known Ara h 1 epitopes [50], dark blue boxes outline known Ana o 1 epitopes [52], grey boxes delineate known olive vicilin T-cell epitopes abbreviated as OVTE and yellow boxes bound the known olive vicilin B-cell epitopes abbreviated as OVBE [51]. All the epitopes are listed in Table 1 and the corresponding sequences identified in the vicilin-like proteins in *B*. *rapa*. pid = percent sequence identity. Blue boxes show the location of the conserved proline and glycine residues.

The identification of five 7S globulin-like vicilin proteins provides the first experimental evidence of these proteins in Brassica (S5 Table in S1 File), fragmentation evidence for all high confidence peptides (>95%) that matched the vicilin sequences in all four pooled populations are shown in S1 Fig. Previously vicilin proteins in the *B*. *rapa* R-o-18 genome had only been inferred from partial complementary DNA (cDNA) sequences, RNAseq data or expressed sequence tags (ESTs), and based on sequence similarities to known legume vicilins [59, 60]. Multiple sequence alignment of the five identified *B*. *rapa* vicilins with vicilin and vicilin-like protein sequences from *Arabidopsis thaliana*, *Cannabis sativa*, cashew (*Anacardium occidental*), narrow-leaf blue lupine (*Lupinus angustifolius*), *w*hite lupine (*Lupinus albus*), sesame (*Sesamum indicum*), pistachio (*Pistacia vera*), European hazel (*Corylus avellana*) and peanut (*Arachis hypogaea*) can be seen in (Fig 6). The alignment indicates significant sequence identity of the five identified *B*. *rapa* vicilins with other vicilin and vicilin like proteins [51, 61, 62]. To confirm this, a pairwise sequence comparison analyses for percent sequence identities (PIDs) was performed comparing the known vicilin sequences in S1 Table in S1 File with the identified 7S globulin like vicilins using CLC Genomics Workbench v3.6.5 (QIAGEN Bioinformatics). A distance matrix, expressing the nearest neighbour distance was created from a multiple sequence alignment with the sequences of similar length using the distmat server (https://www.bioinformatics.nl/cgi-bin/emboss/distmat, 3, 08, 2020). The pairwise comparison shows that there are high sequence similarities among the identified *B*. *rapa* 7S globulin like vicilins and previously identified vicilin sequences suggesting there are close evolutionary relationships between members of this cupin superfamily [15, 63, 64]. A distance matrix was constructed based on the score of the evolutionary distance between each pair of sequences (S2 and S3 Figs) [65, 66].

**Fig 7:**
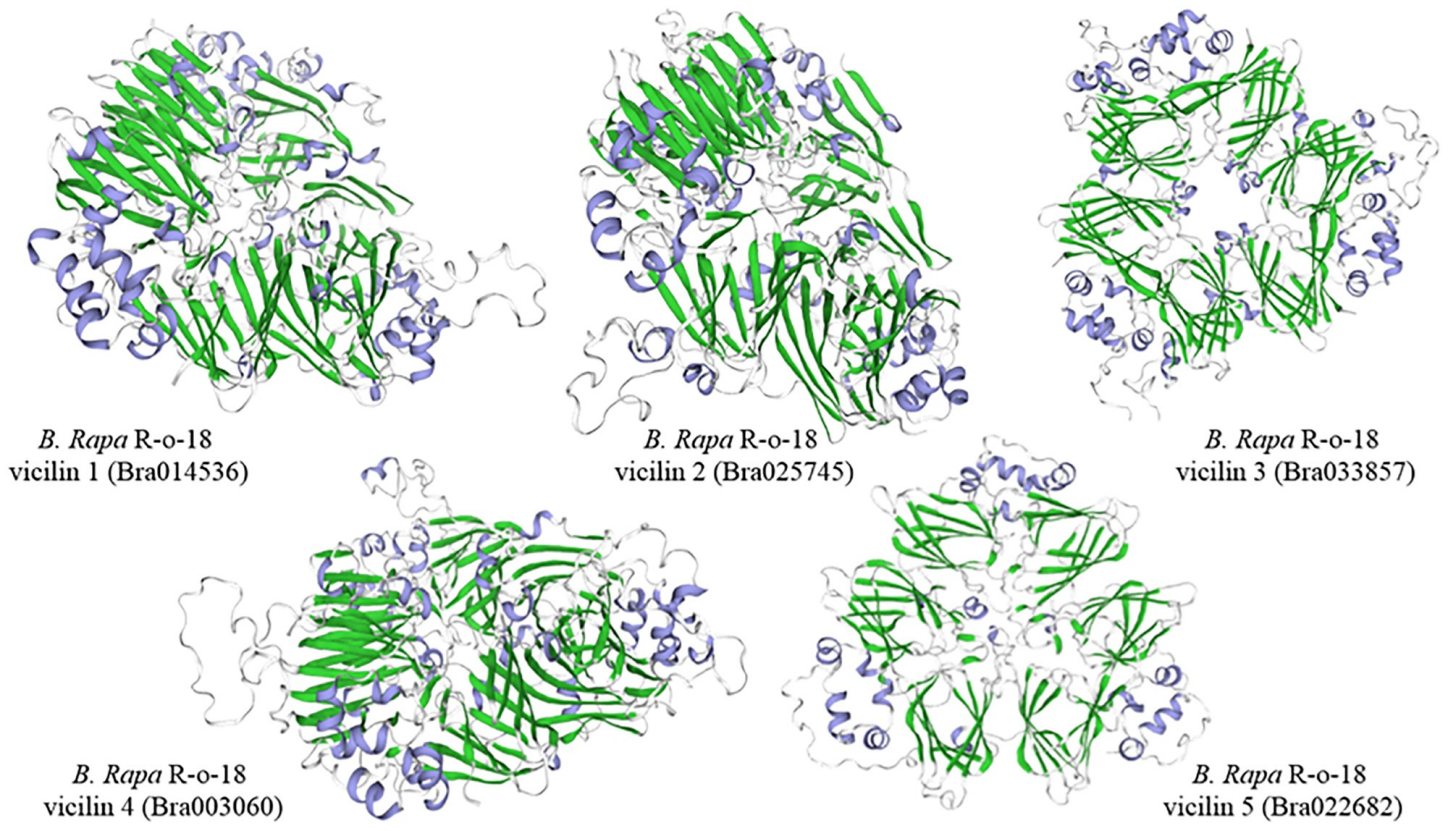
Three-dimensional structure of the vicilin like seed storage proteins identified in *Brassica rapa*. Structural modelling was carried out using the SWISS-MODEL program and was based on the available crystal structures of vicilin (S6 Table in S1 File). The green shading indicates the location of β-sheets in the N-terminal region and the blue shading indicates α-helices at the C-terminus.

Crystallographic studies suggest that all vicilin proteins are composed of two domains, consisting of 11 β-sheets within the N-terminal region, and 3 to 4 α-helices at the C-terminus of the protein [51, 61, 67]. To confirm the presence of these domains in the *B*. *rapa*, vicilin proteins identified in this study, the full-length sequences of identified vicilins were analysed using SWISS-MODEL (https://swissmodel.expasy.org/, 20.06.20) to perform tertiary structure prediction. High-resolution three-dimensional structure models of the identified vicilin proteins were drawn without ligands, as homo-trimers, based on the available crystal structures of vicilins (https://www.rcsb.org/) as template. Details of the 3D models with the quality scores and global model quality estimate (GMQE) and qualitative model energy analysis (QMEAN) values are presented in S6 Table in S1 File [15, 68]. The best models from the tertiary structure analysis based on GMQE and QMEAN values confirmed that the identified vicilin proteins in the *B*. *rapa* R-o-18 seed proteome possess this uniquely shared characteristic property of vicilins, with β-sheets in the N-terminal region and α-helices at the C-terminus (Fig 7) [51].

Within these two domains vicilin proteins show conserved glycine and proline residues located 15 amino acid residues apart [67, 69]. This motif is conserved in the *B*. *rapa* vicilins (Fig 6, blue boxes). Additionally, as noted in other vicilins, there was a lack of cysteine residues and a high abundance of glycine, lysine, glutamic and aspartic acid residues [70–72].

**Fig 8:**
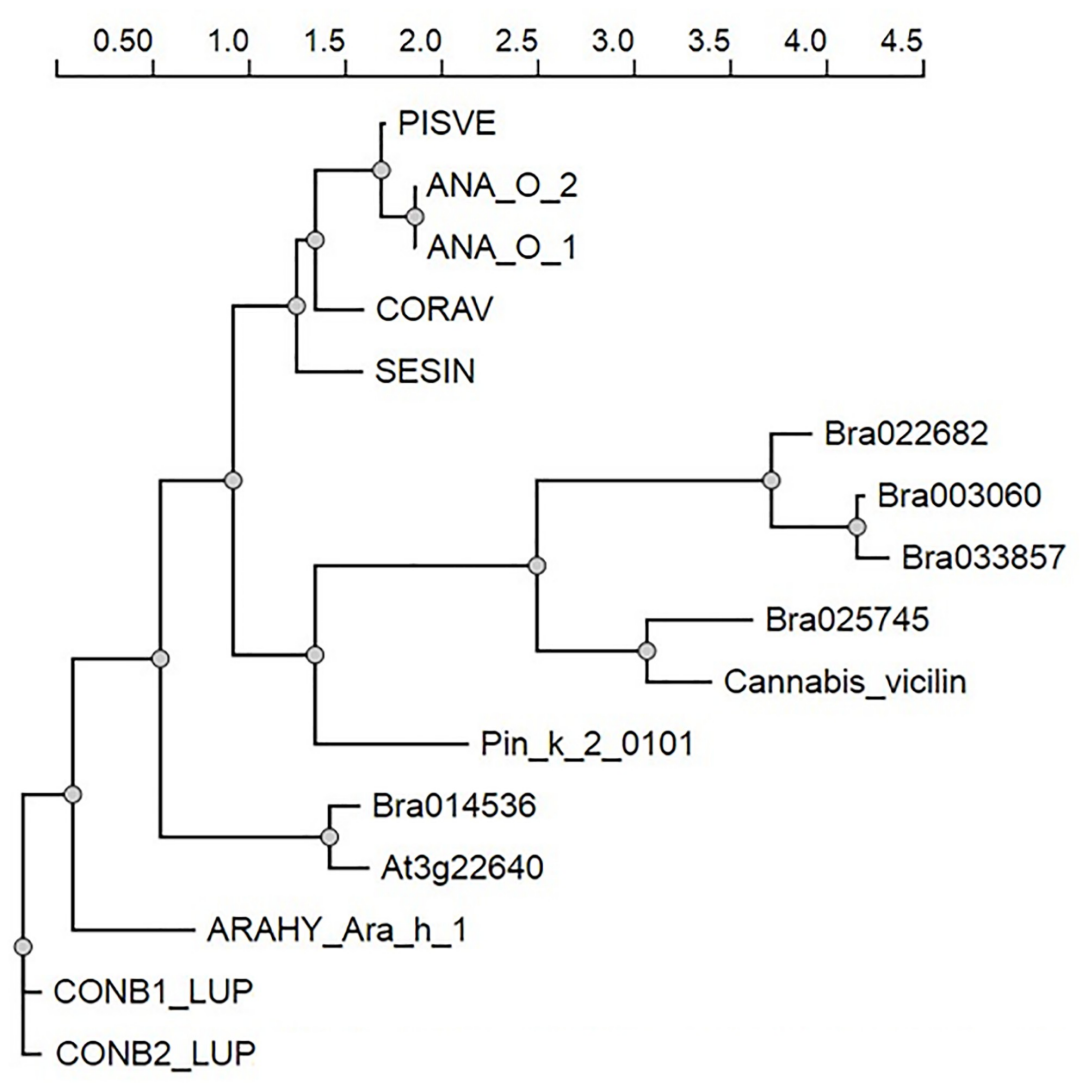
Phylogenetic tree of vicilin-like proteins. The phylogenetic tree was constructed using the maximum likelihood method in NGPhylogeny.fr web resource (https://ngphylogeny.fr/workflows/oneclick/, 30/05/2020). The tree was constructed using the five 7S globulin-like vicilin protein sequences identified in *Brassica rapa* R-o-18, as well as 7S globulin like vicilin protein sequences reported in different plant species including *Arabidopsis thaliana* (At3g22640), *Cannabis sativa* (Cannabis_vicilin), cashew (*Anacardium occidental*) (ANA_O-1 and ANA_O_2), narrow-leaved blue lupine (*Lupinus angustifolius*) (CONB1_LUP), *w*hite lupine (*Lupinus albus*) (CONB2_LUP), sesame (*Sesamum indicum*) (SESIN), *Korean pine (Pinus koraiensis) (Pin_k_2_0101)*, pistachio (*Pistacia vera*) (PISVE), European hazel (*Corylus avellana*) (CORAV) and peanut (*Arachis hypogaea*) (ARAHY_Ara_h_1), all available in public domain databases. The full-length sequences of identified vicilins are presented in S5 Table in S1 File, while the other sequences are presented in S1 Table in S1 File. The scale above the tree represents the in-built distance matrix employed in NGPhylogeny.fr web resource and indicates the distance based on the sequence similarity of their features.

Phylogenetic analysis carried out with well characterized full length vicilin proteins [50–52] showed the evolutionary relationship of the five identified vicilin proteins from *B*. *rapa* to vicilins from other species (Fig 8). *B*. *rapa* vicilin Bra014536 was observed to cluster with the *A*. *thaliana* vicilin (At3g22640), whereas vicilin Bra 025745 was more closely related to the *Cannabis sativa* 7S vicilin-like protein (Cs7S, A0A219D1T7) (Fig 8, S1 Table in S1 File). The other *B*. *rapa* vicilin proteins identified (Bra 033857, Bra 003060 and Bra022682), clustered together in a branch distinct from vicilins from other species.

Vicilins have been shown to induce allergenic responses in humans [36, 50, 52, 73, 74]. To investigate potential allergenicity of the *B*. *rapa* vicilin proteins, epitope mapping was performed using the sequences of *B*. *rapa* vicilins and comparing them to the twenty-three reported epitopes of the well-studied peanut vicilin allergen Ara h 1 [50], eleven epitopes of a cashew allergen Ana o 1 [52] and T- and B- cell epitopes characterized in olive 7S vicilins corresponding to lupine Lup 1, sesame Ses i 3, pistachio Pis v 3 and European hazel Cor a 11 [51] (Table 1, Fig 6, deep blue, orange, grey and yellow boxes, respectively).

**Table 1:** Epitope mapping of *Brassica rapa* vicilin sequences with the twenty-three reported epitopes of the well-studied peanut vicilin allergen Ara h 1.

Epitope number	A.A. sequences	A.A. position	Identified *B*. *rapa* vicilins				
			Bra 025745	Bra 022682	Bra 033857	Bra 003060	Bra 014536
Ara h 1 epitopes (peanut, *Arachis hypogaea*)							
1	*AKSSPYQKKT*	25–34	x	x	x	x	x
2	QEPDDLKQKA	48–57	x	x	x	x	x
3	*LEYDPRCVYD*	65–74	x	x	x	x	x
4	*GERTRGRQPG*	89–98	x	x	x	x	x
5	PGDYDDDRRQ	97–105	x	x	x	x	x
6	PRREEGGRWG	107–116	x	x	x	x	x
7	REREEDWRQP	123–132	x	x	x	x	x
8	EDWRRPSHQQ	134–143	PC	PC	PC	PC	PC
9	QPRKIRPEGR	143–152	PC	PC	PC	PC	PC
10	TPGQFEDFFP	294–303	HC	HC	HC	HC	HC
11	SYLQEFSRNT	311–320	HC	HC	HC	HC	HC
12	FNAEFNEIRR	325–334	HC	HC	HC	HC	HC
13	*EQEERGQRRW*	344–353	x	x	x	x	x
14	DITNPINLRE	393–402	HC	x	HC	HC	HC
15	DNFGRLFEVK	409–418	HC	x	HC	HC	HC
16	GTGNLELVAV	461–470	HC	HC	HC	HC	HC
17	*RRYTARLKEG*	498–507	HC	HC	HC	HC	HC
18	ELHLLGFGIN	525–534	HC	HC	HC	HC	HC
19	HRIFLAGDKD	539–548	HC	HC	HC	HC	HC
20	VDQIEKOAKD	551–560	HC	HC	HC	HC	HC
21	KDLAFPGSGE	559–568	HC	HC	HC	HC	HC
22	*RESHFVSARP*	578–587	HC	PC	HC	HC	HC
23	QSPSSPEKED	597–606	HC	PC	HC	HC	HC
Ana o 1 epitopes (cashew, *Anacardium occidental*)							
1	MGPPTKFSFSLFL	1–15	x	x	x	x	x
2	CKVQRQYDEQQKEQC	41–55	x	x	x	x	x
3	EQQKEQCVKECEKYY	49–53	x	x	x	x	x
4	**KECEKYYKEKKGRER**	57–71	x	x	x	x	x
5	EKKGREREHEEEEEE	65–79	x	x	x	x	x
6	DEAEEEDENPYVFED	145–159	x	x	x	x	x
7	RRGEGPKIWPFTEES	337–351	x	x	x	x	x
8	NITKGGMSVPFYNSR	393–407	PC	PC	PC	PC	PC
9	TKIAIVVSGEGCVEI	409–423	PC	PC	PC	PC	PC
10	SSHPSYKKLRARIRK	433–447	x	x	x	x	x
11	**EEFFFQGPEWRKEKE**	521–535	PC	PC	PC	PC	PC
Olive 7S vicilin T-cell epitopes (OVTE)							
T1, T5	LVIAKLLQP	140–148	x	x	x	x	x
T2	FEMACPHLS	297–305	x	x	x	x	x
T3	INIHDQRPS	231–239	x	x	x	x	x
T4	YVAVASNNQ	336–345	x	x	x	x	x
T6	YVAQGMGTV	194–102	x	x	x	x	x
T7	VVLLPKFTQ		x	x	x	x	x
T8	DSPGMKYRV	63–71	x	x	x	x	x
T9	LVSVLVLCL (in AANOC)	13–22					
Olive 7S vicilin B-cell epitopes (OBVE)							
B1	KHQGEHGRGGGDIL	Sesame	x	x	x	x	x
B2	DQRPSQFNQ	Olive	x	x	x	x	x
B3	QGAMTTPYYNSKA	Olive	PC	PC	PC	PC	PC
B4	EITPDRNPQVQ	Lupine	x	x	x	x	PC
B5	KNNKRYPLA	Olive	PC	PC	PC	PC	PC

The sequences of Ara h 1 and corresponding sequences identified as vicilin-like proteins in *B*. *rapa*. are taken from Fig 6. X = not conserved, PC = partially conserved (≥50% identity in corresponding sequence alignment), HC = highly conserved (>90% identity in corresponding sequence alignment) depending on the degree of amino acid similarity and identity among the Ara h 1 and corresponding *Brassica rapa* vicilin sequences. The italicized epitopes were the most commonly recognised peptides in Ara h 1 sequence by IgE in at least 80% of the sera from sensitive individuals [50]. Peptides reported as immunodominant epitopes are shown in bold [52].

Epitope mapping to compare the known epitope sequences of Ara h 1, Ana o 1, Olive 7S vicilin T and B-cell epitopes and the linear epitopes in lupine and sesame with corresponding sequence motifs in the *B*. *rapa* vicilin proteins indicates that a large number (13 out of 23) of the peanut vicilin epitopes are highly conserved, while others, including cashew and olive vicilin B-cell epitopes are partially conserved based on the degree of amino acid similarity and identity (Fig 6, S5 Table in S1 File). Most of the highly conserved Ara h 1 epitopes are found in the second half of the proteins comprising epitopes 10 to 23. This corresponds to the region of the protein that contains the α-helices at the C-terminus [50, 51, 67]. Sequence analysis of the identified 7S globulin-like vicilins is presented in S9 Table in S1 File.

## Discussion

A shotgun proteomic approach was carried out on extracts from *B*. *rapa* mature seeds to profile seed proteins with a specific focus to identify and catalogue SSP. From a total of 34016 spectra, 323 proteins were identified (Fig 2). This number is similar to protein numbers reported for shotgun seed proteomics of soybean and quinoa seeds, (243 and 352 respectively) [75, 76], although less than the number of proteins identified in seeds from the tree *Camellia oleifera* (1691 proteins) [77], or from rice and barley grains (822 and 1168 proteins, respectively) [78, 79].

Previous seed focused proteomic studies in Brassica oil-seed crops have primarily employed gel-based approaches to identify proteins showing changes in abundance through seed development or under changing conditions [31, 80–86]. While these studies have advanced understanding of specific biological processes important for the seed, they have not provided a general overview of the proteins in the seed that can be obtained by a bottom-up type approach.

In this study five cruciferin (Bra022801, Bra035434, Bra002906, BraA09006896 and P15456], and three napin proteins (Bra041165, BraA03000889 and BraA01001883] were identified in the proteome of the *B*. *rapa* R-o-18 seed extracts (S4 Table in S1 File). Molecular genetic analysis indicated the presence of five genes encoding napin proteins in the *B*. *rapa* R-o-18 genome and 10 genes encoding napin proteins in the Chiifu genome [15] (S8 Table in S1 File). Direct identification of three of these in this study provides protein-level evidence of gene expression which can help to refine gene models.

Previous studies have reported that in Brassica, 12S globulin type cruciferins and 2S albumin type napins together constitute approximately 85%–90% of the total protein in the seed [59, 87]. Cruciferin is the most abundant of these, roughly accounting for 60% of the total protein in the seed. Napin is reported as the second most abundant SSP, representing 20–30% of the total protein [88]. Generally, the ratio of cruciferin to napin, although variable, is in the range of 0.6 to 0.2 [58, 59]. In this study, based on spectral counting of identified proteins, cruciferin accounted for 14.8% of the seed proteome and napin accounted for an additional 2.6%. This represented a napin to cruciferin ratio of 1 to 5 and agrees with the range previously reported.

Identification of vicilin SSP in the *B*. *rapa* seed provides evidence for an important role for this family of SSP in Brassica. Vicilins have been predominantly mentioned as SSP in legumes [70] and tree nuts [36, 52, 89], with only partial gene sequences corresponding to incomplete clones identified in Arabidopsis [59].

Previously it was assumed that cruciferins and napins were the main seed storage proteins in the Brassica seed [59]. However, based on spectral counts, vicilins appear to be close in abundance to napins in the seeds of *B*. *rapa*. The molecular weight of identified vicilins ranges from 52–83 kDa (S5 Table in S1 File), and analysis of seed extract protein profiles from 1D-SDS-PAGE identified a strong band within this range (S4 Fig). Western blotting with a vicilin specific antibody would help to confirm this.

The identification of vicilins in the seed extract of *Brassica rapa* is an important finding, as vicilin-like seed storage proteins are one of the major allergens in tree nuts, including almond (*Prunus dulcis*) [73, 74], cashew (*Anacardium occidental*) [52], *Pinus koraiensis* (Pinaceae) [90, 91] and walnut (*Juglans regia*) [36]. Vicilins from pulses and legumes, including peanut (*Arachis hypogaea*) [50], pea [92], chickpea [93], and lentils [62] have also been shown to elicit strong allergenic responses in humans. Sequence analysis revealed that sequence identity of the *B*. *rapa* vicilins to those of other species ranged from 9.4% to 25.5%, with the highest value obtained for Bra014536 with reference to peanut vicilin Ara h 1 (Fig 6) [51]. Sequence comparisons reported among other vicilins also show quite low sequence identity, for example, cashew vicilin and the peanut vicilin have 27% sequence identity although both are predominant allergens [61]. Similarly, the identity between lentil Len c 1.0101 and sesame Ses i 3 is only 31% [62]. Despite low overall sequence identity, a number of epitopes identified in the peanut Ara h 1 vicilin protein were highly conserved in the vicilins from *B*. *rapa*, including epitopes 10, 11, 12 and 14–24, inclusive (Table 1) [50], with two of these, 17 and 22 corresponding to peptides in the Ara h 1 sequence recognised by IgE antibodies in at least 80% of the sera from sensitive individuals [50]. The epitope modelling shows that identified *B*. *rapa* R-o-18 vicilin like proteins have higher epitope conservation with the Ara h 1 epitopes than that of Ano a 1 and olive vicilin and its homologues. Further clinical testing would need to be carried out to determine the *B*. *rapa* R-o-18 vicilin epitopes which elicit the strongest immune response.

Phylogenetic analysis showed the evolutionary relationship among the identified *B*. *rapa* R-o-18 vicilins to known vicilins and vicilin like proteins (Fig 8). The *B*. *rapa* R-o-18 vicilins which have demonstrated higher sequence conservation of allergenic epitopes with Ara h 1 (Table 1), were found to be rooted from the same evolutionary ancestor and distant from the Ano a 1 vicilin which shared fewer epitopes (Fig 8).

**Fig 9:**
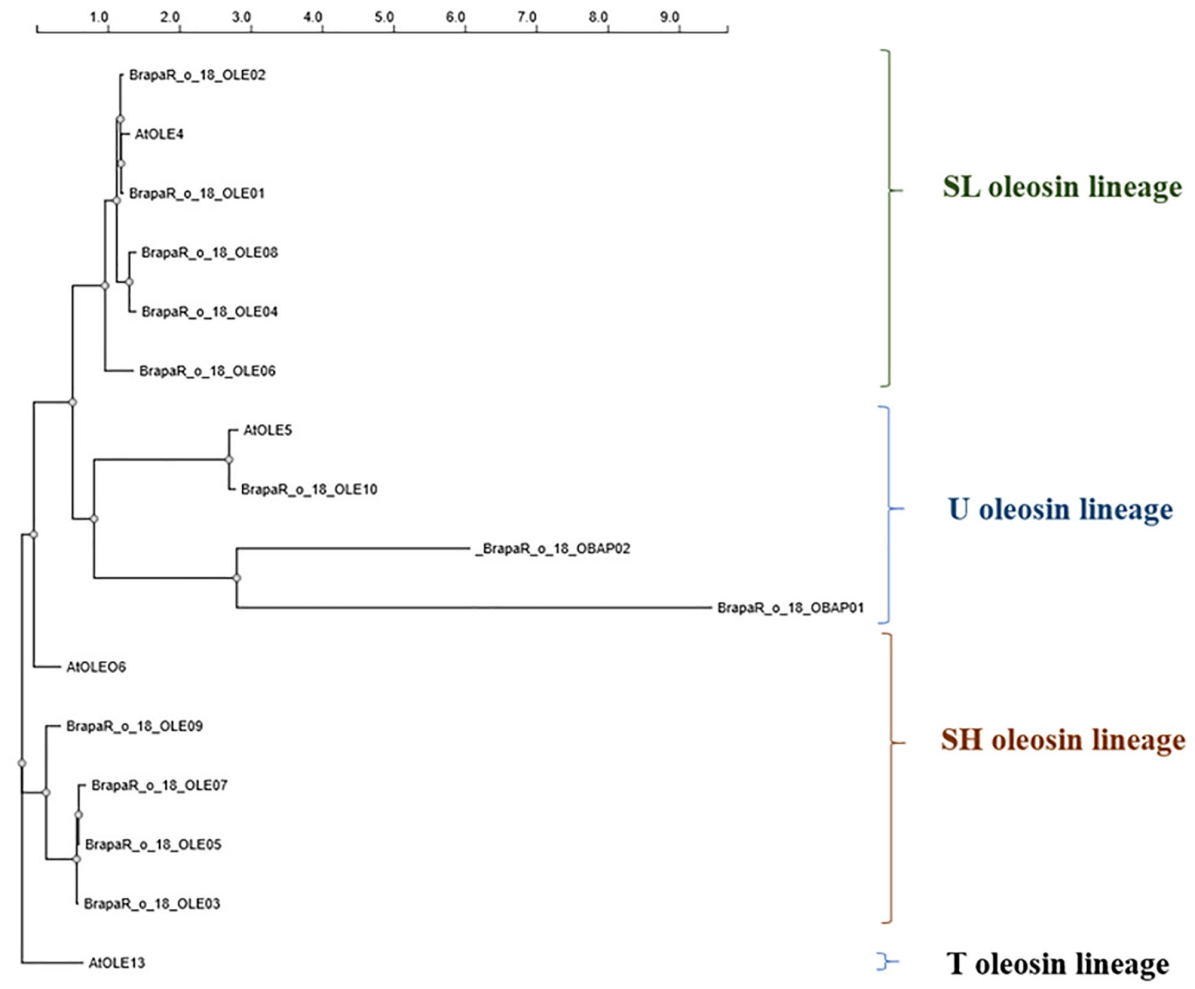
Phylogenetic analysis of identified *Brassica rapa* oleosin and oil body associated proteins. The linear phylogram was built using PRESTO-Phylogenetic tReE viSualisaTiOn server (https://ngphylogeny.fr/data/displaytree//, 29/09/2020) and shows the relationship to the Arabidopsis oleosin genes (S7 Table in S1 File). The branch length scale at the top of the figure indicates the distance between the sequences based on the sequence similarity of their features. The identified oleosin proteins Bra019493, Bra000167, A0A078GHK4, M4E9X1, A0A397KW15, A0A397ZZI8, M4DBK6, M4EI43, Bra035756, Bra032113 and oil-body associated proteins Bra036039 and Bra036039 are designated as OLE01, OLE02, OLE03, OLE04, OLE05, OLE06, OLE07, OLE08, OLE09, OLE10, OBAP 01 and OBAP 02 respectively.

A number of proteins associated with lipid biosynthesis and oil body structure were also identified in the *B*. *rapa* proteome (S5 Table in S1 File) including ten oleosins and two oil body associated proteins. Previous studies have identified up to 30 oleosin genes in *B*. *rapa* after analysis of the draft genome sequence of inbred line Chiifu-401-42 [94], and in this study we were able to identify 12 proteins corresponding to these genes in the seeds. Identified proteins can be placed into three out of the five oleosin lineages described by Huang and Huang, 2015 (Fig 9) [95]. These described lineages include primitive (in green algae, mosses and ferns); universal (U) encompassing all land plants; seed low-molecular weight (SL) from seed plants; seed high molecular weight (SH) from angiosperms and tapetum (T) from Brassicaceae. *B*. *rapa* oil body associated protein Bra0360391, 2 and Bra036099, designated as OBAP01 and OBAP02, respectively, and oleosin Bra032113 (designated as OLE10) originate from U lineage, *B*. *rapa* oleosin Bra0194931, Bra0001672, M4E9X1, A0A397ZZI8, M4EI43, (designated as OLE01, OLE02, OLE04, OLE06 and OLE08, respectively) from SL lineage, *B*. *rapa* oleosin A0A078GHK4, A0A397KW15, M4DBK6 and Bra035756 (designated as OLE03, OLE05, OLE07 and OLE09, respectively) from SH lineage (Fig 8, S6 Table in S1 File) [94]. No oleosin from the T lineage was detected in *B*. *rapa*. Oleosins have been shown to have important roles in determining oil body size, seed size and seed weight which in turn would likely influence oil production [96].

Many of the proteins identified relate to well-studied seed physiological processes. Brassicaceae crops have been reported to synthesize many unique S-containing secondary metabolites which play an important role in oxidative stress signalling and resistance to herbivores [97, 98], the most notable of these is glutathione. In this study six glutathione S-transferases were identified in the seed extracts along with proteins involved in glutathione-linked responses including but not limited to glutathione peroxidase, thioredoxin. and S-formylglutathione hydrolase as well as a sulphur transporter. Several enzymes involved in the cysteine/methionine metabolism were also identified (O-acetylserine (thiol) lyase, cysteine synthase, adenosylhomocysteinase, gltaredoxin, sulfotransferase, sulfate transporter, 5-methyltetrahydropteroyltriglutamate—homocysteine methyltransferase, glutathione S-transferase, adenylate kinate).

There was also a large number of proteins identified in the seed associated with translation (Fig 4). These proteins would likely play a key role in translation of stored mRNA’s during the initial stages of seed imbibition, and evidence has shown that stored translational machinery is already effective during the very first hours upon imbibition [99]. There were also numerous proteins related to desiccation tolerance identified in the seed, including late embryogenesis related proteins (LEA), dehydrins, seed maturation protein (SMP), as well as embryonic maturation protein (EM). These proteins, as well as being important for seed desiccation, play a key role in the early stages of seed imbibition leading to germination [99]. We also identified the repair protein l-isoaspartyl methyltransferase (PIMT) which has been shown to be involved in isoAsp protein repair functions in the seed which functions to increase seed longevity and germination vigour [100, 101].

## Conclusion

This study has catalogued the proteins in *B*. *rapa* R-o-18 seeds which can serve as a reference for future rapeseed protein analyses and provides information that can help to gain insight into the regulation, molecular mechanism and subcellular localization of the identified proteins. Major seed storage proteins were identified, including for the first time in *B*. *rapa*, proteins belonging to the 7S vicilin family of SSPs. Analysis of the sequences of the identified vicilin proteins provided evidence for their potential allergenicity due to high conservation of sequences that have shown to be major allergenic epitopes in vicilins of other species. This epitope identification could be used in the development of recombinant proteins for diagnosis and therapeutic purposes against rapeseed allergy [50, 102].

This study enriches our existing knowledge on rapeseed seed proteins and because of the close evolutionary relationship of *B*. *rapa* with other Brassicas, the knowledge obtained from this study should be useful to further understanding of the proteins of its other progenitors [103, 104], as well as construct a robust foundation and rational basis for plant bioengineering of seed storage proteins to allow improvement of seed protein which will allow the utilization of such an abundant and low cost rapeseed by-product for human consumption.

## Supporting information

S1 Fig Fragmentation evidence of high confidence peptides matching vicilin sequences in all four pooled populations. (DOCX)

S2 Fig Pairwise analysis of full length 7S vicilin sequences. (DOCX)

S3 Fig Distance matrix of the 18 full length vicilin sequences aligned in Supplementary Tables 1 and 5 in S1 File. (DOCX)

S4 Fig SDS-PAGE of proteins extracted from single seeds to demonstrate quality of protein used for MS analysis. (DOCX)

S1 Raw image (PPTX)

S1 File Supplementary tables. (DOCX)

## Abbreviations

1-D-SDS-PAGE: One-Dimensional Sodium Dodecyl Sulfate -Polyacrylamide Gel Electrophoresis
1H NMR: Proton Nuclear Magnetic Resonance
3D-structure: Three Dimensional Structure
BLAST: Basic Local Alignment Search Tool
BP: Biological Process
CC: Cellular Component
cDNA: Complementary Deoxyribonucleic Acid
COV: Sequence Coverage
CRISPR/Cas9: Clustered Regularly Interspaced Short Palindromic Repeats-Associated Protein-9 Nuclease
DNA: Deoxyribonucleic Acid
ESI: Electron Spray Ionization
EST: Expressed Sequence Tag
FASTA: Fast Adaptive Shrinkage Threshold Algorithm
FDR: False Discovery Rate
g: Relative Centrifugal Force, Gravity
GM: Genetically Modified
GMQE: Global Model Quality Estimation
GO: Gene Ontology
HPLC: High Performance Liquid Chromatography
IDA: Information Dependent Acquisition
IgE: Immunoglobulin E
kDa: Kilo Dalton
LC-MS/MS: Liquid Chromatography Coupled to Tandem Mass Spectrometry
MF: Molecular Function
min: Minute
mL: Mili Litre
mm: Milli Metre
MS: Mass Spectrometry, Mass Spectrometer
MSA: Multiple Sequence Alignment
NCBI: National Center for Biotechnology Infromation
PID: Percent Sequence Identity, Pairwise Identity
QMEAN: Qualitative Model Energy Analysis
RNA: Ribonucleic Acid
SDS-PAGE: Sodium Dodecyl Sulfate- Polyacrylamide Gel Electrophoresis
SSP: SSPs, Seed Storage Protein, Seed Storage Proteins
TCA: Trichloroacetic Acid
TOF: Time-of-Flight
wiff: File Extension of Applied Biosystems Raw Data Files
μg: Micro Gram
μL: Micro Litre
