# Supplementary material for: Shotgun proteomics of Brassica rapa seed proteins identifies vicilin as a major seed storage protein in the mature seed
Source: PLoS One. 2021 Jul 9;16(7):e0253384. doi: 10.1371/journal.pone.0253384 (PMC8270179; doi:10.1371/journal.pone.0253384)
Supplement: S1 Fig — (DOCX) [file pone.0253384.s001.docx]

Fragmentation evidence of all the high confidence peptide (95%) that matched the vicilin sequences in all four pooled populations

*The **Protein Sequence Coverage** map shows the protein sequence with matching peptides indicated in color: green for highest confidence, yellow for moderate confidence, and red for lowest confidence. Gray text indicates portions of the protein sequence for which no peptides are identified. The **Fragmentation Evidence** lists the theoretical fragment ions for the peptide sequence, with matching ions shown in **bold** text in the table. The matched ions are drawn on the MS/MS spectrum at the *m/z* for the theoretical ion.

# Sample Pool 1

## Protein: B.rapa.Ro18.Candidate-1, 12 peptides (95%)

*Protein sequence coverage*

MEKNKRIFTFLLVIVFFHGVMMMRSIGYEGEEEQGGGGRERGGFMMKESRQVIKSEGGEMRVVISPRGR**IIEKPMHIGFLTMEPKTLFVPQYLDSNLLIFIR**QGEATLGVICKDEFGEKR**LKGGDIYWIPAGSAFYLLNTGR**GQRLHVICSIDPSQSLGFETFQPFYIGGGPSSVLAGFDPDTITSALNVSRPEVQQLMTSQVRGPIVHITEHAPTMWTDFLGLRGEEKHKHLKKLLELKQGTSQEQEDNPWWSWKNIVSSILDVTGEKNRGSGSSKCEDSYNIYDR**KNDFENDYGWSKALDYDDYEPLR**YSGVGVYLVNLTAGSMMAPHMNPTATEYGIVLSGSGEIQVVLPNGTSAMNMRVSPGDVFWIPRYFAFCQIASR**IAPFEFVGFTTSAYKNRPQFLVGSNSLLR**SLNLTSLAMAFGVDEGTMKRFVEAQR**EAVILPTASAAPPHEGEPER**FGSDHIFT

*Peptide sequence 1:* ALDYDDYEPLR

| Residue | b | b+2 | y | y+2 |
| --- | --- | --- | --- | --- |
| A | 72.0444 | 36.5258 | 1369.6270 | **685.3172** |
| L | **185.1285** | 93.0679 | **1298.5899** | **649.7986** |
| D | **300.1554** | 150.5813 | **1185.5059** | **593.2566** |
| Y | **463.2187** | **232.1130** | **1070.4789** | **535.7431** |
| D | **578.2457** | 289.6265 | **907.4156** | **454.2114** |
| D | **693.2726** | 347.1399 | **792.3886** | 396.6980 |
| Y | **856.3359** | 428.6716 | **677.3617** | 339.1845 |
| E | **985.3785** | 493.1929 | **514.2984** | 257.6528 |
| P | **1082.4313** | 541.7193 | **385.2558** | 193.1315 |
| L | **1195.5154** | **598.2613** | **288.2030** | 144.6051 |
| R | 1351.6165 | **676.3119** | **175.1190** | 88.0631 |

*Peptide sequence 2:* EAVILPTASAAPPHEGEPER

| Residue | b | b+2 | y | y+2 |
| --- | --- | --- | --- | --- |
| E | 130.0499 | 65.5286 | 2071.0455 | 1036.0264 |
| A | **201.0870** | **101.0471** | 1942.0029 | 971.5051 |
| V | **300.1554** | 150.5813 | 1870.9658 | **935.9865** |
| I | **413.2395** | 207.1234 | 1771.8973 | **886.4523** |
| L | **526.3235** | 263.6654 | 1658.8133 | **829.9103** |
| P | 623.3763 | 312.1918 | 1545.7292 | **773.3682** |
| T | 724.4240 | 362.7156 | **1448.6764** | **724.8419** |
| A | 795.4611 | 398.2342 | **1347.6288** | **674.3180** |
| S | 882.4931 | 441.7502 | **1276.5917** | **638.7995** |
| A | 953.5302 | 477.2688 | **1189.5596** | 595.2835 |
| A | 1024.5673 | 512.7873 | **1118.5225** | 559.7649 |
| P | 1121.6201 | 561.3137 | **1047.4854** | **524.2463** |
| P | 1218.6729 | 609.8401 | **950.4326** | 475.7200 |
| H | 1355.7318 | 678.3695 | **853.3799** | 427.1936 |
| E | 1484.7744 | 742.8908 | **716.3210** | 358.6641 |
| G | 1541.7958 | 771.4016 | **587.2784** | 294.1428 |
| E | 1670.8384 | 835.9229 | 530.2569 | 265.6321 |
| P | 1767.8912 | 884.4492 | **401.2143** | **201.1108** |
| E | 1896.9338 | 948.9705 | 304.1615 | 152.5844 |
| R | 2053.0349 | 1027.0211 | **175.1190** | 88.0631 |

*Peptide sequence 3:* GGDIYWIPAGSAFYLLNTGR

| Residue | b | b+2 | y | y+2 |
| --- | --- | --- | --- | --- |
| G | 58.0287 | 29.5180 | 2171.0920 | **1086.0496** |
| G | **115.0502** | 58.0287 | 2114.0706 | 1057.5389 |
| D | **230.0771** | 115.5422 | 2057.0491 | 1029.0282 |
| I | **343.1612** | 172.0842 | 1942.0221 | 971.5147 |
| Y | **506.2245** | 253.6159 | 1828.9381 | 914.9727 |
| W | **692.3039** | 346.6556 | 1665.8748 | 833.4410 |
| I | **805.3879** | 403.1976 | **1479.7954** | **740.4014** |
| P | **902.4407** | 451.7240 | **1366.7114** | **683.8593** |
| A | **973.4778** | 487.2425 | **1269.6586** | 635.3329 |
| G | **1030.4993** | 515.7533 | **1198.6215** | 599.8144 |
| S | **1117.5313** | 559.2693 | **1141.6000** | 571.3037 |
| A | **1188.5684** | 594.7878 | **1054.5680** | 527.7876 |
| F | **1335.6368** | 668.3220 | **983.5309** | 492.2691 |
| Y | **1498.7001** | 749.8537 | **836.4625** | 418.7349 |
| L | 1611.7842 | 806.3957 | **673.3991** | 337.2032 |
| L | 1724.8683 | 862.9378 | **560.3151** | 280.6612 |
| N | 1838.9112 | 919.9592 | **447.2310** | 224.1191 |
| T | 1939.9589 | 970.4831 | **333.1881** | 167.0977 |
| G | 1996.9803 | 998.9938 | **232.1404** | 116.5738 |
| R | 2153.0815 | 1077.0444 | **175.1190** | 88.0631 |

*Peptide sequence 4:* IAPFEFVGFTTSAYK

| Residue | b | b+2 | y | y+2 |
| --- | --- | --- | --- | --- |
| I | 114.0913 | 57.5493 | 1677.8523 | **839.4298** |
| A | **185.1285** | 93.0679 | 1564.7682 | **782.8877** |
| P | **282.1812** | 141.5942 | **1493.7311** | **747.3692** |
| F | **429.2496** | 215.1285 | **1396.6783** | **698.8428** |
| E | **558.2922** | 279.6498 | **1249.6099** | **625.3086** |
| F | **705.3606** | 353.1840 | **1120.5673** | **560.7873** |
| V | **804.4291** | 402.7182 | **973.4989** | **487.2531** |
| G | **861.4505** | 431.2289 | **874.4305** | 437.7189 |
| F | **1008.5189** | **504.7631** | **817.4090** | 409.2082 |
| T | **1109.5666** | 555.2869 | **670.3406** | 335.6740 |
| T | 1210.6143 | 605.8108 | **569.2930** | 285.1501 |
| S | **1297.6463** | **649.3268** | **468.2453** | 234.6263 |
| A | **1368.6834** | 684.8454 | **381.2132** | 191.1103 |
| Y | 1531.7468 | 766.3770 | **310.1761** | 155.5917 |
| K | 1659.8417 | **830.4245** | **147.1128** | 74.0600 |

*Peptide sequence 5:* IIEKPMHIGFLTMEPK

| Residue | b | b+2 | y | y+2 |
| --- | --- | --- | --- | --- |
| I | 114.0913 | 57.5493 | 1884.0122 | 942.5097 |
| I | **227.1754** | 114.0913 | 1770.9281 | 885.9677 |
| E | **356.2180** | 178.6126 | 1657.8440 | **829.4257** |
| K | **484.3130** | 242.6601 | 1528.8015 | **764.9044** |
| P | 581.3657 | 291.1865 | 1400.7065 | **700.8569** |
| M | **712.4062** | 356.7067 | **1303.6537** | **652.3305** |
| H | **849.4651** | **425.2362** | **1172.6132** | **586.8103** |
| I | **962.5492** | **481.7782** | **1035.5543** | **518.2808** |
| G | **1019.5706** | **510.2890** | **922.4703** | **461.7388** |
| F | **1166.6391** | **583.8232** | **865.4488** | **433.2280** |
| L | **1279.7231** | **640.3652** | **718.3804** | **359.6938** |
| T | 1380.7708 | **690.8890** | **605.2963** | **303.1518** |
| M | 1511.8113 | **756.4093** | **504.2486** | **252.6280** |
| E | 1640.8539 | **820.9306** | **373.2082** | **187.1077** |
| P | 1737.9066 | **869.4570** | **244.1656** | 122.5864 |
| K | 1866.0016 | 933.5044 | **147.1128** | 74.0600 |

*Peptide sequence 6:* KNDFENDYGWSK

| Residue | b | b+2 | y | y+2 |
| --- | --- | --- | --- | --- |
| K | **129.1022** | 65.0548 | 1502.6546 | **751.8310** |
| N | **243.1452** | 122.0762 | **1374.5597** | **687.7835** |
| D | **358.1721** | 179.5897 | **1260.5168** | 630.7620 |
| F | **505.2405** | 253.1239 | **1145.4898** | 573.2485 |
| E | **634.2831** | 317.6452 | **998.4214** | 499.7143 |
| N | **748.3260** | 374.6667 | **869.3788** | 435.1930 |
| D | **863.3530** | 432.1801 | **755.3359** | 378.1716 |
| Y | **1026.4163** | 513.7118 | **640.3089** | 320.6581 |
| G | **1083.4378** | **542.2225** | **477.2456** | 239.1264 |
| W | **1269.5171** | 635.2622 | **420.2241** | 210.6157 |
| S | **1356.5491** | **678.7782** | **234.1448** | 117.5761 |
| K | 1484.6441 | **742.8257** | **147.1128** | 74.0600 |

*Peptide sequence 7:* LKGGDIYWIPAGSAFYLLNTGR

| Residue | b | b+2 | y | y+2 |
| --- | --- | --- | --- | --- |
| L | 114.0913 | 57.5493 | 2412.2710 | 1206.6392 |
| K | **242.1863** | 121.5968 | 2299.1870 | 1150.0971 |
| G | 299.2078 | 150.1075 | 2171.0920 | 1086.0496 |
| G | 356.2292 | 178.6183 | 2114.0706 | 1057.5389 |
| D | **471.2562** | 236.1317 | 2057.0491 | 1029.0282 |
| I | **584.3402** | 292.6738 | 1942.0221 | 971.5147 |
| Y | **747.4036** | 374.2054 | 1828.9381 | **914.9727** |
| W | **933.4829** | **467.2451** | 1665.8748 | **833.4410** |
| I | **1046.5669** | **523.7871** | **1479.7954** | **740.4014** |
| P | **1143.6197** | **572.3135** | **1366.7114** | **683.8593** |
| A | **1214.6568** | **607.8320** | **1269.6586** | **635.3329** |
| G | 1271.6783 | **636.3428** | **1198.6215** | **599.8144** |
| S | **1358.7103** | **679.8588** | **1141.6000** | 571.3037 |
| A | **1429.7474** | **715.3774** | **1054.5680** | 527.7876 |
| F | 1576.8158 | **788.9116** | **983.5309** | **492.2691** |
| Y | 1739.8792 | **870.4432** | **836.4625** | **418.7349** |
| L | 1852.9632 | **926.9853** | **673.3991** | **337.2032** |
| L | 1966.0473 | **983.5273** | **560.3151** | 280.6612 |
| N | 2080.0902 | **1040.5488** | **447.2310** | 224.1191 |
| T | 2181.1379 | 1091.0726 | **333.1881** | 167.0977 |
| G | 2238.1594 | 1119.5833 | **232.1404** | 116.5738 |
| R | 2394.2605 | 1197.6339 | **175.1190** | 88.0631 |

*Peptide sequence 8:* NRPQFLVGSNSLLR

| Residue | b | b+2 | y | y+2 |
| --- | --- | --- | --- | --- |
| N | **115.0502** | 58.0287 | 1600.8918 | 800.9495 |
| R | **271.1513** | **136.0793** | 1486.8489 | 743.9281 |
| P | **368.2041** | 184.6057 | 1330.7478 | **665.8775** |
| Q | **496.2627** | **248.6350** | 1233.6950 | 617.3511 |
| F | **643.3311** | **322.1692** | **1105.6364** | **553.3218** |
| L | **756.4151** | **378.7112** | **958.5680** | **479.7876** |
| V | **855.4835** | **428.2454** | **845.4839** | **423.2456** |
| G | **912.5050** | **456.7561** | **746.4155** | **373.7114** |
| S | **999.5370** | **500.2722** | **689.3941** | **345.2007** |
| N | **1113.5800** | **557.2936** | **602.3620** | **301.6847** |
| S | **1200.6120** | **600.8096** | **488.3191** | 244.6632 |
| L | 1313.6961 | **657.3517** | **401.2871** | **201.1472** |
| L | 1426.7801 | **713.8937** | **288.2030** | 144.6051 |
| R | 1582.8812 | 791.9443 | **175.1190** | 88.0631 |

*Peptide sequence 9:* TLFVPQYLDSNLLIFIR

| Residue | b | b+2 | y | y+2 |
| --- | --- | --- | --- | --- |
| T | 102.0550 | 51.5311 | 2052.1528 | 1026.5801 |
| L | **215.1390** | 108.0731 | 1951.1051 | 976.0562 |
| F | **362.2074** | 181.6074 | 1838.0211 | **919.5142** |
| V | **461.2758** | 231.1416 | 1690.9527 | **845.9800** |
| P | **558.3286** | 279.6679 | 1591.8843 | **796.4458** |
| Q | **686.3872** | **343.6972** | 1494.8315 | 747.9194 |
| Y | **849.4505** | **425.2289** | **1366.7729** | 683.8901 |
| L | **962.5346** | 481.7709 | **1203.7096** | **602.3584** |
| D | **1077.5615** | 539.2844 | **1090.6255** | 545.8164 |
| S | **1164.5936** | 582.8004 | **975.5986** | 488.3029 |
| N | **1278.6365** | **639.8219** | **888.5665** | 444.7869 |
| L | **1391.7205** | **696.3639** | **774.5236** | 387.7654 |
| L | 1504.8046 | **752.9059** | **661.4396** | **331.2234** |
| I | 1617.8887 | **809.4480** | **548.3555** | **274.6814** |
| F | 1764.9571 | 882.9822 | **435.2714** | 218.1394 |
| I | 1878.0411 | 939.5242 | **288.2030** | 144.6051 |
| R | 2034.1423 | 1017.5748 | **175.1190** | 88.0631 |

*Peptide sequence 10:* NRPQFLVGSN[Dea]SLLR

| Residue | b | b+2 | y | y+2 |
| --- | --- | --- | --- | --- |
| N | 115.0502 | 58.0287 | 1601.8758 | 801.4415 |
| R | **271.1513** | **136.0793** | 1487.8329 | 744.4201 |
| P | 368.2041 | 184.6057 | 1331.7318 | 666.3695 |
| Q | **496.2627** | 248.6350 | 1234.6790 | 617.8431 |
| F | **643.3311** | **322.1692** | **1106.6204** | 553.8139 |
| L | **756.4151** | **378.7112** | **959.5520** | 480.2796 |
| V | **855.4835** | **428.2454** | **846.4680** | **423.7376** |
| G | **912.5050** | **456.7561** | **747.3995** | **374.2034** |
| S | **999.5370** | 500.2722 | **690.3781** | **345.6927** |
| N[Dea] | 1114.5640 | 557.7856 | **603.3461** | 302.1767 |
| S | 1201.5960 | 601.3016 | **488.3191** | 244.6632 |
| L | 1314.6801 | 657.8437 | **401.2871** | **201.1472** |
| L | 1427.7641 | 714.3857 | **288.2030** | 144.6051 |
| R | 1583.8653 | 792.4363 | **175.1190** | 88.0631 |

*Peptide sequence 11:* IIEKPMHIGFLTM[Oxi]EPK

| Residue | b | b+2 | y | y+2 |
| --- | --- | --- | --- | --- |
| I | 114.0913 | 57.5493 | 1900.0071 | 950.5072 |
| I | **227.1754** | 114.0913 | 1786.9230 | 893.9652 |
| E | **356.2180** | 178.6126 | 1673.8390 | **837.4231** |
| K | **484.3130** | 242.6601 | 1544.7964 | **772.9018** |
| P | 581.3657 | 291.1865 | **1416.7014** | **708.8543** |
| M | **712.4062** | 356.7067 | 1319.6486 | 660.3280 |
| H | **849.4651** | **425.2362** | **1188.6082** | **594.8077** |
| I | **962.5492** | **481.7782** | **1051.5492** | **526.2783** |
| G | 1019.5706 | **510.2890** | **938.4652** | **469.7362** |
| F | **1166.6391** | **583.8232** | **881.4437** | 441.2255 |
| L | 1279.7231 | **640.3652** | **734.3753** | **367.6913** |
| T | 1380.7708 | **690.8890** | **621.2912** | **311.1493** |
| M[Oxi] | 1527.8062 | **764.4067** | **520.2436** | **260.6254** |
| E | 1656.8488 | 828.9280 | **373.2082** | 187.1077 |
| P | 1753.9016 | 877.4544 | **244.1656** | 122.5864 |
| K | 1881.9965 | **941.5019** | **147.1128** | 74.0600 |

*Peptide sequence 12:* IIEKPM[Oxi]HIGFLTMEPK

| Residue | b | b+2 | y | y+2 |
| --- | --- | --- | --- | --- |
| I | 114.0913 | 57.5493 | 1900.0071 | 950.5072 |
| I | **227.1754** | 114.0913 | 1786.9230 | 893.9652 |
| E | **356.2180** | 178.6126 | 1673.8390 | 837.4231 |
| K | 484.3130 | 242.6601 | 1544.7964 | **772.9018** |
| P | 581.3657 | 291.1865 | 1416.7014 | **708.8543** |
| M[Oxi] | **728.4011** | 364.7042 | 1319.6486 | **660.3280** |
| H | **865.4600** | **433.2337** | **1172.6132** | 586.8103 |
| I | **978.5441** | **489.7757** | **1035.5543** | **518.2808** |
| G | **1035.5656** | **518.2864** | **922.4703** | **461.7388** |
| F | **1182.6340** | **591.8206** | **865.4488** | **433.2280** |
| L | 1295.7180 | **648.3627** | **718.3804** | **359.6938** |
| T | 1396.7657 | **698.8865** | **605.2963** | **303.1518** |
| M | 1527.8062 | **764.4067** | **504.2486** | **252.6280** |
| E | 1656.8488 | 828.9280 | **373.2082** | 187.1077 |
| P | 1753.9016 | 877.4544 | **244.1656** | 122.5864 |
| K | 1881.9965 | 941.5019 | **147.1128** | 74.0600 |

## Protein: B.rapa.Ro18.Candidate-2, 14 peptides (95%)

*Protein sequence coverage*

MIRFTVLSFFVVFVLLFACNESSAKTAKYDKSDESVENDDLAAVPSCCGFSSPLLIK**KDQWKPIFANK**FGQISTVQIGDGCGGMGPYKIHSITLEPNALMLPLLLHSDMVFFVDSGSGILNWVEAQATSTEIRLGDVYR**LRPGTVFYLQSKPVDIFLGTK**LKIYAIFSNSQECLHDPCFGAYSSVTDLLFGFDETILK**SAFGVPEEIIGLMR**NRTQPPLIVHDMLTTPGEANTDTDTNTWPLQTRLLKLFSGDASADSVENKKVKKEKKEKKEKKKKPK**KATTFNVFESEPDFQSPNGQTITINRK**DLKVLQGSMVGVSMVNLTQGSMMGPHWNPWACEISVVVRGSGMVRVLRNSISRSSSECKNMR**FKVEKGDIFAVPR**LHPMAQMSFLNDSLVFVGFTTSAK**NNEPQFLAGKNSALWSLDR**EVLAASFNVSSFMIAGLLEAQKEAAVLGCPACAEGELEKLKEDEEKKESPPQQPPQPFQPQPPGEKPQQPPQPFQPQPPQGEPQKPPQGEPQKPPQGEPEGPQKPFQPQPGQGEPQEPQASMETKMRDEERKREEEEAKKEEEERWKQEEKLWPTQPQWED

*Peptide sequence 1:* DQWKPIFANK

| Residue | b | b+2 | y | y+2 |
| --- | --- | --- | --- | --- |
| D | 116.0342 | 58.5207 | 1246.6579 | 623.8326 |
| Q | **244.0928** | 122.5500 | 1131.6309 | 566.3191 |
| W | **430.1721** | 215.5897 | 1003.5724 | **502.2898** |
| K | **558.2671** | **279.6372** | **817.4931** | **409.2502** |
| P | **655.3198** | **328.1636** | **689.3981** | **345.2027** |
| I | **768.4039** | **384.7056** | **592.3453** | 296.6763 |
| F | **915.4723** | 458.2398 | **479.2613** | **240.1343** |
| A | **986.5094** | **493.7584** | **332.1928** | 166.6001 |
| N | 1100.5524 | 550.7798 | **261.1557** | 131.0815 |
| K | 1228.6473 | 614.8273 | **147.1128** | 74.0600 |

*Peptide sequence 2:* FKVEKGDIFAVPR

| Residue | b | b+2 | y | y+2 |
| --- | --- | --- | --- | --- |
| F | 148.0757 | 74.5415 | 1505.8475 | 753.4274 |
| K | **276.1707** | 138.5890 | 1358.7791 | **679.8932** |
| V | **375.2391** | 188.1232 | 1230.6841 | **615.8457** |
| E | **504.2817** | 252.6445 | 1131.6157 | **566.3115** |
| K | **632.3766** | 316.6919 | **1002.5731** | **501.7902** |
| G | **689.3981** | 345.2027 | **874.4781** | 437.7427 |
| D | **804.4250** | **402.7162** | **817.4567** | 409.2320 |
| I | **917.5091** | **459.2582** | **702.4297** | 351.7185 |
| F | 1064.5775 | **532.7924** | **589.3457** | **295.1765** |
| A | **1135.6146** | **568.3109** | **442.2772** | 221.6423 |
| V | 1234.6830 | 617.8452 | **371.2401** | 186.1237 |
| P | 1331.7358 | 666.3715 | **272.1717** | 136.5895 |
| R | 1487.8369 | 744.4221 | **175.1190** | 88.0631 |

*Peptide sequence 3:* GDIFAVPR

| Residue | b | b+2 | y | y+2 |
| --- | --- | --- | --- | --- |
| G | 58.0287 | 29.5180 | 874.4781 | **437.7427** |
| D | **173.0557** | 87.0315 | **817.4567** | **409.2320** |
| I | **286.1397** | 143.5735 | **702.4297** | **351.7185** |
| F | **433.2082** | **217.1077** | **589.3457** | **295.1765** |
| A | **504.2453** | 252.6263 | **442.2772** | 221.6423 |
| V | **603.3137** | 302.1605 | **371.2401** | 186.1237 |
| P | **700.3665** | 350.6869 | **272.1717** | 136.5895 |
| R | 856.4676 | **428.7374** | **175.1190** | 88.0631 |

*Peptide sequence 4:* KATTFNVFESEPDFQSPNGQTITINR

| Residue | b | b+2 | y | y+2 |
| --- | --- | --- | --- | --- |
| K | **129.1022** | 65.0548 | 2941.4326 | 1471.2200 |
| A | **200.1394** | 100.5733 | 2813.3377 | 1407.1725 |
| T | **301.1870** | 151.0972 | 2742.3006 | 1371.6539 |
| T | **402.2347** | 201.6210 | 2641.2529 | 1321.1301 |
| F | **549.3031** | 275.1552 | 2540.2052 | 1270.6062 |
| N | **663.3461** | **332.1767** | 2393.1368 | 1197.0720 |
| V | **762.4145** | 381.7109 | 2279.0939 | 1140.0506 |
| F | **909.4829** | 455.2451 | 2180.0255 | **1090.5164** |
| E | **1038.5255** | 519.7664 | 2032.9570 | 1016.9822 |
| S | **1125.5575** | **563.2824** | 1903.9144 | 952.4609 |
| E | **1254.6001** | 627.8037 | 1816.8824 | **908.9448** |
| P | 1351.6529 | 676.3301 | 1687.8398 | **844.4236** |
| D | **1466.6798** | 733.8435 | 1590.7871 | 795.8972 |
| F | 1613.7482 | **807.3777** | **1475.7601** | **738.3837** |
| Q | 1741.8068 | **871.4070** | **1328.6917** | **664.8495** |
| S | 1828.8388 | **914.9230** | **1200.6331** | **600.8202** |
| P | 1925.8916 | **963.4494** | **1113.6011** | **557.3042** |
| N | 2039.9345 | 1020.4709 | 1016.5483 | 508.7778 |
| G | 2096.9560 | 1048.9816 | **902.5054** | 451.7563 |
| Q | 2225.0146 | 1113.0109 | **845.4839** | **423.2456** |
| T | 2326.0622 | 1163.5348 | **717.4254** | 359.2163 |
| I | 2439.1463 | 1220.0768 | 616.3777 | 308.6925 |
| T | 2540.1940 | 1270.6006 | 503.2936 | 252.1504 |
| I | 2653.2780 | 1327.1427 | 402.2459 | 201.6266 |
| N | 2767.3210 | 1384.1641 | 289.1619 | 145.0846 |
| R | 2923.4221 | 1462.2147 | 175.1190 | 88.0631 |

*Peptide sequence 5:* KATTFNVFESEPDFQSPNGQTITINRK

| Residue | b | b+2 | y | y+2 |
| --- | --- | --- | --- | --- |
| K | **129.1022** | 65.0548 | 3069.5276 | 1535.2674 |
| A | **200.1394** | 100.5733 | 2941.4326 | 1471.2200 |
| T | **301.1870** | 151.0972 | 2870.3955 | 1435.7014 |
| T | **402.2347** | 201.6210 | 2769.3478 | 1385.1776 |
| F | **549.3031** | 275.1552 | 2668.3002 | 1334.6537 |
| N | **663.3461** | **332.1767** | 2521.2318 | 1261.1195 |
| V | **762.4145** | **381.7109** | 2407.1888 | 1204.0981 |
| F | 909.4829 | **455.2451** | 2308.1204 | **1154.5638** |
| E | **1038.5255** | **519.7664** | 2161.0520 | **1081.0296** |
| S | **1125.5575** | **563.2824** | 2032.0094 | **1016.5083** |
| E | **1254.6001** | **627.8037** | 1944.9774 | **972.9923** |
| P | 1351.6529 | 676.3301 | 1815.9348 | **908.4710** |
| D | **1466.6798** | 733.8435 | 1718.8820 | **859.9446** |
| F | 1613.7482 | **807.3777** | 1603.8551 | **802.4312** |
| Q | 1741.8068 | 871.4070 | **1456.7867** | **728.8970** |
| S | 1828.8388 | 914.9230 | **1328.7281** | **664.8677** |
| P | 1925.8916 | 963.4494 | **1241.6961** | **621.3517** |
| N | 2039.9345 | 1020.4709 | **1144.6433** | **572.8253** |
| G | 2096.9560 | 1048.9816 | **1030.6004** | **515.8038** |
| Q | 2225.0146 | 1113.0109 | **973.5789** | **487.2931** |
| T | 2326.0622 | 1163.5348 | **845.5203** | **423.2638** |
| I | 2439.1463 | 1220.0768 | **744.4726** | **372.7400** |
| T | 2540.1940 | 1270.6006 | **631.3886** | **316.1979** |
| I | 2653.2780 | 1327.1427 | **530.3409** | 265.6741 |
| N | 2767.3210 | 1384.1641 | **417.2568** | 209.1321 |
| R | 2923.4221 | 1462.2147 | **303.2139** | 152.1106 |
| K | 3051.5170 | 1526.2622 | **147.1128** | 74.0600 |

*Peptide sequence 6:* KDQWKPIFANK

| Residue | b | b+2 | y | y+2 |
| --- | --- | --- | --- | --- |
| K | **129.1022** | 65.0548 | 1374.7528 | 687.8801 |
| D | **244.1292** | 122.5682 | 1246.6579 | **623.8326** |
| Q | **372.1878** | 186.5975 | 1131.6309 | **566.3191** |
| W | **558.2671** | 279.6372 | **1003.5724** | 502.2898 |
| K | **686.3620** | **343.6847** | **817.4931** | 409.2502 |
| P | 783.4148 | **392.2110** | **689.3981** | **345.2027** |
| I | **896.4989** | **448.7531** | **592.3453** | 296.6763 |
| F | 1043.5673 | 522.2873 | **479.2613** | 240.1343 |
| A | **1114.6044** | **557.8058** | **332.1928** | 166.6001 |
| N | 1228.6473 | **614.8273** | **261.1557** | 131.0815 |
| K | 1356.7423 | 678.8748 | **147.1128** | 74.0600 |

*Peptide sequence 7:* LRPGTVFYLQSKPVDIFLGTK

| Residue | b | b+2 | y | y+2 |
| --- | --- | --- | --- | --- |
| L | 114.0913 | 57.5493 | 2379.3435 | 1190.1754 |
| R | **270.1925** | 135.5999 | 2266.2594 | 1133.6333 |
| P | 367.2452 | 184.1262 | 2110.1583 | 1055.5828 |
| G | 424.2667 | 212.6370 | 2013.1055 | 1007.0564 |
| T | **525.3144** | 263.1608 | 1956.0841 | 978.5457 |
| V | **624.3828** | **312.6950** | 1855.0364 | 928.0218 |
| F | **771.4512** | **386.2292** | 1755.9680 | **878.4876** |
| Y | **934.5145** | **467.7609** | 1608.8996 | **804.9534** |
| L | **1047.5986** | **524.3029** | 1445.8362 | **723.4218** |
| Q | **1175.6572** | 588.3322 | **1332.7522** | **666.8797** |
| S | **1262.6892** | 631.8482 | **1204.6936** | **602.8504** |
| K | 1390.7841 | 695.8957 | **1117.6616** | **559.3344** |
| P | 1487.8369 | **744.4221** | **989.5666** | **495.2869** |
| V | 1586.9053 | 793.9563 | **892.5138** | 446.7606 |
| D | 1701.9323 | **851.4698** | **793.4454** | 397.2264 |
| I | 1815.0163 | 908.0118 | **678.4185** | 339.7129 |
| F | 1962.0847 | 981.5460 | **565.3344** | 283.1709 |
| L | 2075.1688 | 1038.0880 | **418.2660** | 209.6366 |
| G | 2132.1903 | 1066.5988 | **305.1819** | 153.0946 |
| T | 2233.2380 | 1117.1226 | **248.1605** | 124.5839 |
| K | 2361.3329 | 1181.1701 | **147.1128** | 74.0600 |

*Peptide sequence 8:* NNEPQFLAGK

| Residue | b | b+2 | y | y+2 |
| --- | --- | --- | --- | --- |
| N | **115.0502** | 58.0287 | 1117.5636 | **559.2855** |
| N | **229.0931** | **115.0502** | **1003.5207** | **502.2640** |
| E | **358.1357** | 179.5715 | **889.4778** | **445.2425** |
| P | **455.1885** | 228.0979 | **760.4352** | **380.7212** |
| Q | **583.2471** | 292.1272 | **663.3824** | **332.1949** |
| F | **730.3155** | 365.6614 | **535.3239** | 268.1656 |
| L | **843.3995** | 422.2034 | **388.2554** | 194.6314 |
| A | **914.4367** | 457.7220 | **275.1714** | 138.0893 |
| G | **971.4581** | 486.2327 | **204.1343** | 102.5708 |
| K | 1099.5531 | **550.2802** | **147.1128** | 74.0600 |
|  |  |  |  |  |

*Peptide sequence 9:* NSALWSLDR

| Residue | b | b+2 | y | y+2 |
| --- | --- | --- | --- | --- |
| N | **115.0502** | 58.0287 | 1061.5374 | **531.2724** |
| S | **202.0822** | 101.5448 | **947.4945** | **474.2509** |
| A | **273.1193** | 137.0633 | **860.4625** | 430.7349 |
| L | **386.2034** | 193.6053 | **789.4254** | **395.2163** |
| W | **572.2827** | 286.6450 | **676.3413** | **338.6743** |
| S | **659.3148** | 330.1610 | **490.2620** | 245.6346 |
| L | **772.3988** | 386.7030 | **403.2300** | 202.1186 |
| D | 887.4258 | 444.2165 | **290.1459** | 145.5766 |
| R | 1043.5269 | **522.2671** | **175.1190** | 88.0631 |

*Peptide sequence 10:* SAFGVPEEIIGLMR

| Residue | b | b+2 | y | y+2 |
| --- | --- | --- | --- | --- |
| S | 88.0393 | 44.5233 | 1518.7985 | **759.9029** |
| A | **159.0764** | 80.0418 | 1431.7664 | **716.3869** |
| F | **306.1448** | 153.5761 | **1360.7293** | **680.8683** |
| G | **363.1663** | 182.0868 | **1213.6609** | **607.3341** |
| V | **462.2347** | 231.6210 | **1156.6395** | **578.8234** |
| P | **559.2875** | 280.1474 | **1057.5710** | **529.2892** |
| E | **688.3301** | 344.6687 | **960.5183** | 480.7628 |
| E | **817.3727** | 409.1900 | **831.4757** | **416.2415** |
| I | **930.4567** | 465.7320 | **702.4331** | 351.7202 |
| I | **1043.5408** | 522.2740 | **589.3490** | 295.1782 |
| G | **1100.5623** | 550.7848 | **476.2650** | 238.6361 |
| L | **1213.6463** | **607.3268** | **419.2435** | 210.1254 |
| M | **1344.6868** | 672.8470 | **306.1594** | 153.5834 |
| R | 1500.7879 | **750.8976** | **175.1190** | 88.0631 |

*Peptide sequence 11:* KATTFNVFESEPDFQSPN[Dea]GQTITINR

| Residue | b | b+2 | y | y+2 |
| --- | --- | --- | --- | --- |
| K | **129.1022** | 65.0548 | 2942.4167 | 1471.7120 |
| A | 200.1394 | 100.5733 | 2814.3217 | 1407.6645 |
| T | **301.1870** | 151.0972 | 2743.2846 | 1372.1459 |
| T | **402.2347** | 201.6210 | 2642.2369 | 1321.6221 |
| F | 549.3031 | 275.1552 | 2541.1892 | 1271.0982 |
| N | **663.3461** | 332.1767 | 2394.1208 | 1197.5640 |
| V | **762.4145** | 381.7109 | 2280.0779 | 1140.5426 |
| F | **909.4829** | 455.2451 | 2181.0095 | 1091.0084 |
| E | **1038.5255** | 519.7664 | 2033.9411 | 1017.4742 |
| S | 1125.5575 | **563.2824** | 1904.8985 | 952.9529 |
| E | **1254.6001** | 627.8037 | 1817.8664 | 909.4369 |
| P | 1351.6529 | 676.3301 | 1688.8238 | **844.9156** |
| D | 1466.6798 | 733.8435 | 1591.7711 | 796.3892 |
| F | 1613.7482 | 807.3777 | 1476.7441 | 738.8757 |
| Q | 1741.8068 | 871.4070 | 1329.6757 | **665.3415** |
| S | 1828.8388 | 914.9230 | **1201.6171** | 601.3122 |
| P | 1925.8916 | 963.4494 | **1114.5851** | 557.7962 |
| N[Dea] | 2040.9185 | 1020.9629 | 1017.5323 | 509.2698 |
| G | 2097.9400 | 1049.4736 | **902.5054** | 451.7563 |
| Q | 2225.9986 | 1113.5029 | 845.4839 | 423.2456 |
| T | 2327.0462 | 1164.0268 | **717.4254** | 359.2163 |
| I | 2440.1303 | 1220.5688 | **616.3777** | 308.6925 |
| T | 2541.1780 | 1271.0926 | **503.2936** | 252.1504 |
| I | 2654.2621 | 1327.6347 | **402.2459** | 201.6266 |
| N | 2768.3050 | 1384.6561 | **289.1619** | **145.0846** |
| R | 2924.4061 | 1462.7067 | **175.1190** | 88.0631 |

*Peptide sequence 12:* KATTFNVFESEPDFQSPN[Dea]GQTITINRK

| Residue | b | b+2 | y | y+2 |
| --- | --- | --- | --- | --- |
| K | **129.1022** | 65.0548 | 3070.5116 | 1535.7594 |
| A | **200.1394** | 100.5733 | 2942.4167 | 1471.7120 |
| T | **301.1870** | 151.0972 | 2871.3795 | 1436.1934 |
| T | **402.2347** | 201.6210 | 2770.3319 | 1385.6696 |
| F | **549.3031** | 275.1552 | 2669.2842 | 1335.1457 |
| N | **663.3461** | **332.1767** | 2522.2158 | 1261.6115 |
| V | **762.4145** | **381.7109** | 2408.1728 | 1204.5901 |
| F | **909.4829** | **455.2451** | 2309.1044 | **1155.0559** |
| E | **1038.5255** | **519.7664** | 2162.0360 | **1081.5216** |
| S | **1125.5575** | **563.2824** | 2032.9934 | **1017.0004** |
| E | **1254.6001** | **627.8037** | 1945.9614 | **973.4843** |
| P | 1351.6529 | 676.3301 | 1816.9188 | **908.9630** |
| D | **1466.6798** | 733.8435 | 1719.8660 | **860.4367** |
| F | 1613.7482 | **807.3777** | 1604.8391 | **802.9232** |
| Q | 1741.8068 | 871.4070 | **1457.7707** | **729.3890** |
| S | 1828.8388 | 914.9230 | **1329.7121** | **665.3597** |
| P | 1925.8916 | 963.4494 | **1242.6801** | **621.8437** |
| N[Dea] | 2040.9185 | 1020.9629 | **1145.6273** | **573.3173** |
| G | 2097.9400 | 1049.4736 | **1030.6004** | 515.8038 |
| Q | 2225.9986 | 1113.5029 | **973.5789** | 487.2931 |
| T | 2327.0462 | 1164.0268 | **845.5203** | 423.2638 |
| I | 2440.1303 | 1220.5688 | **744.4726** | 372.7400 |
| T | 2541.1780 | 1271.0926 | **631.3886** | **316.1979** |
| I | 2654.2621 | 1327.6347 | **530.3409** | 265.6741 |
| N | 2768.3050 | 1384.6561 | **417.2568** | 209.1321 |
| R | 2924.4061 | 1462.7067 | **303.2139** | 152.1106 |
| K | 3052.5011 | 1526.7542 | **147.1128** | 74.0600 |

*Peptide sequence 13:* KATTFN[Dea]VFESEPDFQSPNGQTITINR

| Residue | b | b+2 | y | y+2 |
| --- | --- | --- | --- | --- |
| K | **129.1022** | 65.0548 | 2942.4167 | 1471.7120 |
| A | 200.1394 | 100.5733 | 2814.3217 | 1407.6645 |
| T | **301.1870** | 151.0972 | 2743.2846 | 1372.1459 |
| T | **402.2347** | 201.6210 | 2642.2369 | 1321.6221 |
| F | 549.3031 | 275.1552 | 2541.1892 | 1271.0982 |
| N[Dea] | **664.3301** | 332.6687 | 2394.1208 | 1197.5640 |
| V | **763.3985** | 382.2029 | 2279.0939 | 1140.0506 |
| F | **910.4669** | 455.7371 | 2180.0255 | 1090.5164 |
| E | **1039.5095** | 520.2584 | 2032.9570 | 1016.9822 |
| S | 1126.5415 | 563.7744 | 1903.9144 | 952.4609 |
| E | **1255.5841** | 628.2957 | 1816.8824 | 908.9448 |
| P | 1352.6369 | 676.8221 | 1687.8398 | **844.4236** |
| D | 1467.6638 | 734.3355 | 1590.7871 | 795.8972 |
| F | 1614.7322 | 807.8698 | 1475.7601 | 738.3837 |
| Q | 1742.7908 | 871.8990 | 1328.6917 | 664.8495 |
| S | 1829.8228 | 915.4151 | **1200.6331** | 600.8202 |
| P | 1926.8756 | 963.9414 | **1113.6011** | **557.3042** |
| N | 2040.9185 | 1020.9629 | 1016.5483 | 508.7778 |
| G | 2097.9400 | 1049.4736 | **902.5054** | 451.7563 |
| Q | 2225.9986 | 1113.5029 | 845.4839 | 423.2456 |
| T | 2327.0462 | 1164.0268 | **717.4254** | 359.2163 |
| I | 2440.1303 | 1220.5688 | **616.3777** | 308.6925 |
| T | 2541.1780 | 1271.0926 | **503.2936** | 252.1504 |
| I | 2654.2621 | 1327.6347 | **402.2459** | 201.6266 |
| N | 2768.3050 | 1384.6561 | **289.1619** | **145.0846** |
| R | 2924.4061 | 1462.7067 | **175.1190** | 88.0631 |

*Peptide sequence 14:* NNEPQ[Dea]FLAGK

| Residue | b | b+2 | y | y+2 |
| --- | --- | --- | --- | --- |
| N | 115.0502 | 58.0287 | 1118.5477 | **559.7775** |
| N | **229.0931** | 115.0502 | **1004.5047** | **502.7560** |
| E | **358.1357** | 179.5715 | **890.4618** | 445.7345 |
| P | 455.1885 | 228.0979 | **761.4192** | **381.2132** |
| Q[Dea] | 584.2311 | 292.6192 | **664.3665** | 332.6869 |
| F | **731.2995** | 366.1534 | **535.3239** | 268.1656 |
| L | **844.3836** | 422.6954 | **388.2554** | 194.6314 |
| A | 915.4207 | 458.2140 | **275.1714** | 138.0893 |
| G | 972.4421 | 486.7247 | **204.1343** | 102.5708 |
| K | 1100.5371 | **550.7722** | **147.1128** | 74.0600 |

## Protein: B.rapa.Ro18.Candidate-3, 2 peptides (95%)

*Protein sequence coverage*

MTKFTVLPLFVLLFLVLLCTKSWAKSEEFDESSDEENDVAAVPSCCGFSSPLLIKKDQWKPIFGTQFGQISTVQIGEGCGGMGPYKIHSITLEPNALLLPLLLHSDMVFFVESGSGILNWVEAEPTSSEIRRGDVYRLRPGTVFYLQSKPIDIFLGTKLRVYAIFSNTEECLHDPCFGAYSSITDLLFGFDEAILQSAFGVPEEIIGLMTNRTQPPLIVHDMLSTPGEANTYTWQLQVQPRLLKLFAGYVSAAEKKKKEKKTKKAKTFNVFESEPDFQSPSGRTITINRKDLEVLSGSMVGVSMVNLTQASMMGPHWNPWACEISIVLKGSGMVRVLRSSISSTSSSSSSSECKNMR**FKVEEGDIFAVPR**LHPMAQMSFINESLVFIGFTTSARNNEPQFLAGQRSALRLLDQEVLAASLNVSSVMIEGLLGAQKDAVVLGCPYCAEGELEKLKVETEMKKRDDERKREEEEAKKEEEERRKREEEEEEEKQWPPLPQQPPE

*Peptide sequence 1:* FKVEEGDIFAVPR

| Residue | b | b+2 | y | y+2 |
| --- | --- | --- | --- | --- |
| F | 148.0757 | 74.5415 | 1506.7951 | 753.9012 |
| K | **276.1707** | 138.5890 | 1359.7267 | **680.3670** |
| V | **375.2391** | 188.1232 | 1231.6317 | **616.3195** |
| E | **504.2817** | 252.6445 | 1132.5633 | 566.7853 |
| E | **633.3243** | 317.1658 | **1003.5207** | 502.2640 |
| G | **690.3457** | 345.6765 | **874.4781** | 437.7427 |
| D | **805.3727** | **403.1900** | **817.4567** | 409.2320 |
| I | **918.4567** | **459.7320** | **702.4297** | **351.7185** |
| F | **1065.5251** | **533.2662** | **589.3457** | **295.1765** |
| A | **1136.5623** | **568.7848** | **442.2772** | **221.6423** |
| V | **1235.6307** | **618.3190** | **371.2401** | **186.1237** |
| P | 1332.6834 | 666.8454 | **272.1717** | 136.5895 |
| R | 1488.7845 | 744.8959 | **175.1190** | 88.0631 |

## Protein: B.rapa.Ro18.Candidate-4, 2 peptides (95%)

*Protein sequence coverage*

MSKFTIIPLCLLTLFLCTNSFSDQNDGVPSSQSPLLVKRHQRTQLVATEFGEISAVHIGEEYTIQFITLEPNALLLPLLLHSDMVFFVHTGSGVLNWVDEEKERTLELKRGDVFRLRYGTVFYLHCNLERDEVPEKLR**VYAIFDVGK**CLSDQCLGAYSSIRDLLWGFDEKTLRSAFAVPKDVFGRLRDAVKPPLITHAMPKNRTQGSEEETWGSRLAKLFVRVEDVTDHLEMKPVVNKKKKKKKKKSSAYNVFESDPDFENDNGQSIVVDEKDMDALKGSSFGVYMVNLTKGSMMGPHWNPNACEISIVLQGEGMIRVVNHPSYQSKNESERFMVEDGDVFVVPQFYPMAQLSFVNSSFMFMGFSTSAK**TNHPQFLVGQNSVLK**IFNRDVLATSFNMRYATVERLLGAQKDGLLLECVSCAEVELSRLMREIEERRRREEEEIERRKREEEEAKRQEEERRRREEEEAERKKKAEEEARKREKEREREEEAAKRREEERRRREEEEAERKRKEEEEARKREEERKREEEAAKKREEERRKREKEEEEARKREEAREREEEEAKKREEERRKREEEEAERKRRAEEEAREREEEEAKKREEEKEAARRREEEREKEEEMAKRREEERQRKEREDVERKKREEEEERKRREEEAMRREEERKREEEAAKRAEEERRKREEEAEHKKRPPPQGPQPPIHH

*Peptide sequence 1:* TNHPQFLVGQNSVLK

| Residue | b | b+2 | y | y+2 |
| --- | --- | --- | --- | --- |
| T | 102.0550 | 51.5311 | 1681.9020 | **841.4547** |
| N | 216.0979 | 108.5526 | 1580.8544 | 790.9308 |
| H | **353.1568** | 177.0820 | 1466.8114 | 733.9094 |
| P | **450.2096** | 225.6084 | 1329.7525 | **665.3799** |
| Q | **578.2681** | 289.6377 | 1232.6997 | 616.8535 |
| F | **725.3365** | **363.1719** | 1104.6412 | 552.8242 |
| L | **838.4206** | **419.7139** | 957.5728 | 479.2900 |
| V | **937.4890** | **469.2482** | **844.4887** | **422.7480** |
| G | **994.5105** | 497.7589 | **745.4203** | **373.2138** |
| Q | 1122.5691 | **561.7882** | **688.3988** | 344.7030 |
| N | 1236.6120 | 618.8096 | **560.3402** | 280.6738 |
| S | 1323.6440 | 662.3257 | **446.2973** | 223.6523 |
| V | 1422.7124 | 711.8599 | 359.2653 | 180.1363 |
| L | 1535.7965 | 768.4019 | 260.1969 | 130.6021 |
| K | 1663.8915 | 832.4494 | **147.1128** | 74.0600 |

*Peptide sequence 2:* VYAIFDVGK

| Residue | b | b+2 | y | y+2 |
| --- | --- | --- | --- | --- |
| V | 100.0757 | 50.5415 | 1011.5510 | **506.2791** |
| Y | **263.1390** | 132.0731 | **912.4825** | **456.7449** |
| A | **334.1761** | 167.5917 | **749.4192** | **375.2132** |
| I | **447.2602** | 224.1337 | **678.3821** | 339.6947 |
| F | 594.3286 | 297.6679 | **565.2980** | 283.1527 |
| D | 709.3556 | 355.1814 | **418.2296** | 209.6185 |
| V | 808.4240 | 404.7156 | **303.2027** | 152.1050 |
| G | 865.4454 | 433.2264 | **204.1343** | 102.5708 |
| K | 993.5404 | 497.2738 | **147.1128** | 74.0600 |

## Protein: B.rapa.Ro18.Candidate-5, 21 peptides (95%)

*Protein sequence coverage*

MAINKLTITLFLLISLAVFHCLAFRVEVQEFEPPRQEGQEGPGGGSGEGWDEEATK**NPYHFGQWSFKNFFQSK**DGFVKMLPKFTKR**SSTLFR**GIENYR**FLFQEMQPNTFLVPHHLDADYVFLVVQGK**GVIGFVTDTANESFQITKGDVVR**VPSSVTHFFANTNGTVPLR**LAK**IAVPANVPGHFQVFFPAHSGFHQSYFNGFSKDVLTASFNIPEELLGRLIRGPQQEVGQGIIRRVSPEQIKELTEHEHATSPSNK**HKDKKDKHKDKDR**STFGSPFNLLTQDAIYSNNFGR**YHEAHPKRFSQLQDLDIAVGWVNMTQGSLFLPQYNSETTFVTFVENGCARYEMASPYTFQGEQQQPWFGPGQEEEVEEEMSGQVHKIVSRVCKGEVFILPAGHPFAILSQDENFVAVGFGIHASNSTR**TFLAGQDNMLSNINTVATRLSFGLGSK**MAEKLFTSQNYSHFAPTTPSHQFPEKPKPSFQSVFNLVGF

*Peptide sequence 1:* DVLTASFNIPEELLGR

| Residue | b | b+2 | y | y+2 |
| --- | --- | --- | --- | --- |
| D | 116.0342 | 58.5207 | 1773.9381 | **887.4727** |
| V | **215.1026** | 108.0550 | 1658.9112 | 829.9592 |
| L | **328.1867** | 164.5970 | 1559.8428 | **780.4250** |
| T | **429.2344** | **215.1208** | **1446.7587** | **723.8830** |
| A | **500.2715** | 250.6394 | **1345.7110** | **673.3592** |
| S | **587.3035** | **294.1554** | **1274.6739** | **637.8406** |
| F | **734.3719** | 367.6896 | **1187.6419** | **594.3246** |
| N | **848.4149** | 424.7111 | **1040.5735** | 520.7904 |
| I | **961.4989** | **481.2531** | **926.5306** | **463.7689** |
| P | **1058.5517** | 529.7795 | **813.4465** | 407.2269 |
| E | 1187.5943 | 594.3008 | **716.3937** | 358.7005 |
| E | **1316.6369** | 658.8221 | **587.3511** | **294.1792** |
| L | **1429.7209** | **715.3641** | **458.3085** | 229.6579 |
| L | 1542.8050 | **771.9061** | **345.2245** | **173.1159** |
| G | 1599.8265 | 800.4169 | **232.1404** | 116.5738 |
| R | 1755.9276 | **878.4674** | **175.1190** | 88.0631 |

*Peptide sequence 2:* ELTEHEHATSPSNK

| Residue | b | b+2 | y | y+2 |
| --- | --- | --- | --- | --- |
| E | 130.0499 | 65.5286 | 1579.7347 | 790.3710 |
| L | **243.1339** | 122.0706 | 1450.6921 | **725.8497** |
| T | **344.1816** | 172.5944 | 1337.6080 | **669.3077** |
| E | 473.2242 | 237.1157 | 1236.5604 | **618.7838** |
| H | **610.2831** | 305.6452 | 1107.5178 | **554.2625** |
| E | **739.3257** | 370.1665 | **970.4588** | **485.7331** |
| H | **876.3846** | **438.6959** | **841.4163** | **421.2118** |
| A | 947.4217 | **474.2145** | **704.3573** | 352.6823 |
| T | **1048.4694** | **524.7383** | **633.3202** | **317.1638** |
| S | **1135.5014** | 568.2544 | **532.2726** | **266.6399** |
| P | 1232.5542 | 616.7807 | **445.2405** | **223.1239** |
| S | 1319.5862 | **660.2968** | **348.1878** | 174.5975 |
| N | 1433.6292 | **717.3182** | **261.1557** | 131.0815 |
| K | 1561.7241 | 781.3657 | **147.1128** | 74.0600 |

*Peptide sequence 3:* FLFQEMQPNTFLVPHHLDADYVFLVVQGK

| Residue | b | b+2 | y | y+2 |
| --- | --- | --- | --- | --- |
| F | 148.0757 | 74.5415 | 3432.7449 | 1716.8761 |
| L | **261.1598** | 131.0835 | 3285.6765 | 1643.3419 |
| F | **408.2282** | 204.6177 | 3172.5925 | 1586.7999 |
| Q | **536.2867** | 268.6470 | 3025.5240 | 1513.2657 |
| E | **665.3293** | **333.1683** | 2897.4655 | 1449.2364 |
| M | **796.3698** | 398.6885 | 2768.4229 | 1384.7151 |
| Q | **924.4284** | 462.7178 | 2637.3824 | 1319.1948 |
| P | 1021.4812 | 511.2442 | 2509.3238 | **1255.1655** |
| N | 1135.5241 | 568.2657 | 2412.2710 | 1206.6392 |
| T | **1236.5718** | 618.7895 | 2298.2281 | 1149.6177 |
| F | **1383.6402** | 692.3237 | 2197.1804 | **1099.0939** |
| L | 1496.7242 | 748.8658 | 2050.1120 | **1025.5596** |
| V | 1595.7927 | 798.4000 | 1937.0280 | **969.0176** |
| P | 1692.8454 | 846.9264 | 1837.9595 | **919.4834** |
| H | 1829.9043 | 915.4558 | 1740.9068 | 870.9570 |
| H | 1966.9633 | 983.9853 | 1603.8479 | **802.4276** |
| L | 2080.0473 | 1040.5273 | **1466.7890** | 733.8981 |
| D | 2195.0743 | 1098.0408 | **1353.7049** | 677.3561 |
| A | 2266.1114 | 1133.5593 | 1238.6780 | 619.8426 |
| D | 2381.1383 | 1191.0728 | 1167.6408 | 584.3241 |
| Y | 2544.2016 | **1272.6045** | **1052.6139** | 526.8106 |
| V | 2643.2701 | 1322.1387 | **889.5506** | 445.2789 |
| F | 2790.3385 | 1395.6729 | **790.4822** | 395.7447 |
| L | 2903.4225 | 1452.2149 | **643.4137** | 322.2105 |
| V | 3002.4909 | 1501.7491 | **530.3297** | 265.6685 |
| V | 3101.5594 | 1551.2833 | **431.2613** | 216.1343 |
| Q | 3229.6179 | 1615.3126 | **332.1928** | 166.6001 |
| G | 3286.6394 | 1643.8233 | **204.1343** | 102.5708 |
| K | 3414.7344 | 1707.8708 | **147.1128** | 74.0600 |

*Peptide sequence 4:* GPQQEVGQGIIR

| Residue | b | b+2 | y | y+2 |
| --- | --- | --- | --- | --- |
| G | 58.0287 | 29.5180 | 1281.6910 | **641.3491** |
| P | **155.0815** | 78.0444 | 1224.6695 | **612.8384** |
| Q | **283.1401** | 142.0737 | **1127.6167** | **564.3120** |
| Q | **411.1987** | 206.1030 | **999.5582** | **500.2827** |
| E | **540.2413** | 270.6243 | **871.4996** | **436.2534** |
| V | **639.3097** | 320.1585 | **742.4570** | **371.7321** |
| G | **696.3311** | 348.6692 | **643.3886** | 322.1979 |
| Q | **824.3897** | 412.6985 | **586.3671** | 293.6872 |
| G | **881.4112** | 441.2092 | **458.3085** | 229.6579 |
| I | **994.4952** | 497.7513 | **401.2871** | **201.1472** |
| I | **1107.5793** | **554.2933** | **288.2030** | 144.6051 |
| R | 1263.6804 | **632.3438** | **175.1190** | 88.0631 |

*Peptide sequence 5:* IAVPANVPGHFQVFFPAHSGFHQSYFNGFSK

| Residue | b | b+2 | y | y+2 |
| --- | --- | --- | --- | --- |
| I | 114.0913 | 57.5493 | 3434.6858 | 1717.8465 |
| A | **185.1285** | 93.0679 | 3321.6017 | 1661.3045 |
| V | **284.1969** | 142.6021 | 3250.5646 | 1625.7859 |
| P | **381.2496** | 191.1285 | 3151.4962 | 1576.2517 |
| A | **452.2867** | 226.6470 | 3054.4434 | 1527.7253 |
| N | **566.3297** | 283.6685 | 2983.4063 | 1492.2068 |
| V | **665.3981** | 333.2027 | 2869.3634 | 1435.1853 |
| P | 762.4509 | 381.7291 | 2770.2950 | **1385.6511** |
| G | 819.4723 | 410.2398 | 2673.2422 | **1337.1247** |
| H | 956.5312 | 478.7693 | 2616.2207 | **1308.6140** |
| F | 1103.5996 | 552.3035 | 2479.1618 | **1240.0846** |
| Q | 1231.6582 | **616.3327** | 2332.0934 | **1166.5503** |
| V | 1330.7266 | 665.8670 | 2204.0348 | **1102.5211** |
| F | 1477.7950 | **739.4012** | 2104.9664 | **1052.9868** |
| F | 1624.8635 | 812.9354 | 1957.8980 | **979.4526** |
| P | 1721.9162 | 861.4618 | 1810.8296 | **905.9184** |
| A | 1792.9533 | 896.9803 | 1713.7768 | **857.3921** |
| H | 1930.0122 | 965.5098 | 1642.7397 | **821.8735** |
| S | 2017.0443 | 1009.0258 | 1505.6808 | **753.3440** |
| G | 2074.0657 | 1037.5365 | **1418.6488** | **709.8280** |
| F | 2221.1342 | 1111.0707 | **1361.6273** | **681.3173** |
| H | 2358.1931 | 1179.6002 | **1214.5589** | **607.7831** |
| Q | 2486.2516 | 1243.6295 | **1077.5000** | **539.2536** |
| S | 2573.2837 | 1287.1455 | **949.4414** | **475.2243** |
| Y | 2736.3470 | 1368.6771 | **862.4094** | **431.7083** |
| F | 2883.4154 | 1442.2113 | **699.3461** | 350.1767 |
| N | 2997.4583 | 1499.2328 | **552.2776** | 276.6425 |
| G | 3054.4798 | 1527.7435 | **438.2347** | 219.6210 |
| F | 3201.5482 | 1601.2777 | **381.2132** | 191.1103 |
| S | 3288.5802 | 1644.7938 | **234.1448** | 117.5761 |
| K | 3416.6752 | 1708.8412 | **147.1128** | 74.0600 |

*Peptide sequence 6:* LIRGPQQEVGQGIIR

| Residue | b | b+2 | y | y+2 |
| --- | --- | --- | --- | --- |
| L | 114.0913 | 57.5493 | 1663.9602 | 832.4837 |
| I | **227.1754** | 114.0913 | 1550.8761 | **775.9417** |
| R | **383.2765** | 192.1419 | 1437.7921 | **719.3997** |
| G | **440.2980** | 220.6526 | 1281.6910 | **641.3491** |
| P | 537.3507 | 269.1790 | 1224.6695 | 612.8384 |
| Q | **665.4093** | 333.2083 | 1127.6167 | 564.3120 |
| Q | 793.4679 | 397.2376 | **999.5582** | **500.2827** |
| E | **922.5105** | 461.7589 | **871.4996** | 436.2534 |
| V | **1021.5789** | 511.2931 | **742.4570** | **371.7321** |
| G | **1078.6004** | 539.8038 | **643.3886** | **322.1979** |
| Q | **1206.6589** | 603.8331 | **586.3671** | **293.6872** |
| G | 1263.6804 | 632.3438 | **458.3085** | 229.6579 |
| I | 1376.7645 | 688.8859 | **401.2871** | **201.1472** |
| I | 1489.8485 | 745.4279 | **288.2030** | 144.6051 |
| R | 1645.9496 | 823.4785 | **175.1190** | 88.0631 |

*Peptide sequence 7:* LSFGLGSK

| Residue | b | b+2 | y | y+2 |
| --- | --- | --- | --- | --- |
| L | 114.0913 | 57.5493 | 808.4563 | **404.7318** |
| S | **201.1234** | 101.0653 | **695.3723** | **348.1898** |
| F | **348.1918** | 174.5995 | **608.3402** | **304.6738** |
| G | **405.2132** | 203.1103 | **461.2718** | 231.1396 |
| L | **518.2973** | 259.6523 | **404.2504** | 202.6288 |
| G | 575.3188 | 288.1630 | **291.1663** | 146.0868 |
| S | 662.3508 | 331.6790 | **234.1448** | 117.5761 |
| K | 790.4458 | **395.7265** | **147.1128** | 74.0600 |

*Peptide sequence 8:* NPYHFGQWSFK

| Residue | b | b+2 | y | y+2 |
| --- | --- | --- | --- | --- |
| N | 115.0502 | 58.0287 | 1410.6589 | 705.8331 |
| P | **212.1030** | 106.5551 | 1296.6160 | **648.8116** |
| Y | **375.1663** | 188.0868 | **1199.5633** | **600.2853** |
| H | **512.2252** | 256.6162 | **1036.4999** | **518.7536** |
| F | **659.2936** | 330.1504 | **899.4410** | **450.2241** |
| G | **716.3151** | **358.6612** | **752.3726** | 376.6899 |
| Q | **844.3737** | **422.6905** | **695.3511** | 348.1792 |
| W | **1030.4530** | 515.7301 | **567.2926** | **284.1499** |
| S | **1117.4850** | 559.2461 | **381.2132** | **191.1103** |
| F | 1264.5534 | 632.7803 | **294.1812** | 147.5942 |
| K | 1392.6484 | 696.8278 | **147.1128** | 74.0600 |

*Peptide sequence 9:* STFGSPFNLLTQDAIYSNNFGR

| Residue | b | b+2 | y | y+2 |
| --- | --- | --- | --- | --- |
| S | 88.0393 | 44.5233 | 2449.1783 | 1225.0928 |
| T | **189.0870** | 95.0471 | 2362.1462 | 1181.5768 |
| F | **336.1554** | 168.5813 | 2261.0986 | 1131.0529 |
| G | **393.1769** | 197.0921 | 2114.0301 | **1057.5187** |
| S | **480.2089** | 240.6081 | 2057.0087 | **1029.0080** |
| P | **577.2617** | 289.1345 | 1969.9767 | **985.4920** |
| F | **724.3301** | 362.6687 | 1872.9239 | 936.9656 |
| N | **838.3730** | **419.6901** | 1725.8555 | **863.4314** |
| L | **951.4571** | **476.2322** | 1611.8125 | **806.4099** |
| L | **1064.5411** | **532.7742** | **1498.7285** | **749.8679** |
| T | **1165.5888** | **583.2980** | **1385.6444** | **693.3258** |
| Q | **1293.6474** | 647.3273 | **1284.5967** | **642.8020** |
| D | **1408.6743** | **704.8408** | **1156.5382** | 578.7727 |
| A | **1479.7114** | **740.3594** | **1041.5112** | **521.2592** |
| I | 1592.7955 | **796.9014** | **970.4741** | **485.7407** |
| Y | 1755.8588 | **878.4331** | **857.3900** | **429.1987** |
| S | 1842.8909 | 921.9491 | **694.3267** | **347.6670** |
| N | 1956.9338 | 978.9705 | **607.2947** | **304.1510** |
| N | 2070.9767 | 1035.9920 | **493.2518** | **247.1295** |
| F | 2218.0451 | **1109.5262** | **379.2088** | **190.1081** |
| G | 2275.0666 | 1138.0369 | **232.1404** | 116.5738 |
| R | 2431.1677 | 1216.0875 | **175.1190** | 88.0631 |

*Peptide sequence 10:* TFLAGQDNMLSNINTVATR

| Residue | b | b+2 | y | y+2 |
| --- | --- | --- | --- | --- |
| T | 102.0550 | 51.5311 | 2066.0335 | 1033.5204 |
| F | **249.1234** | 125.0653 | 1964.9858 | 982.9966 |
| L | **362.2074** | 181.6074 | 1817.9174 | **909.4623** |
| A | **433.2445** | **217.1259** | 1704.8334 | **852.9203** |
| G | **490.2660** | 245.6366 | 1633.7962 | **817.4018** |
| Q | **618.3246** | 309.6659 | 1576.7748 | **788.8910** |
| D | **733.3515** | 367.1794 | **1448.7162** | 724.8617 |
| N | **847.3945** | **424.2009** | **1333.6893** | 667.3483 |
| M | **978.4349** | **489.7211** | **1219.6463** | 610.3268 |
| L | **1091.5190** | **546.2631** | **1088.6058** | **544.8066** |
| S | **1178.5510** | **589.7792** | **975.5218** | **488.2645** |
| N | **1292.5940** | 646.8006 | **888.4898** | **444.7485** |
| I | **1405.6780** | **703.3427** | **774.4468** | **387.7271** |
| N | 1519.7210 | 760.3641 | **661.3628** | **331.1850** |
| T | 1620.7686 | **810.8880** | **547.3198** | 274.1636 |
| V | 1719.8370 | 860.4222 | **446.2722** | 223.6397 |
| A | 1790.8742 | 895.9407 | **347.2037** | 174.1055 |
| T | 1891.9218 | 946.4646 | **276.1666** | 138.5870 |
| R | 2048.0229 | 1024.5151 | **175.1190** | 88.0631 |

*Peptide sequence 11:* VPSSVTHFFANTNGTVPLR

| Residue | b | b+2 | y | y+2 |
| --- | --- | --- | --- | --- |
| V | 100.0757 | 50.5415 | 2044.0610 | 1022.5342 |
| P | **197.1285** | 99.0679 | 1944.9926 | **973.0000** |
| S | **284.1605** | 142.5839 | 1847.9399 | **924.4736** |
| S | **371.1925** | **186.0999** | 1760.9078 | **880.9576** |
| V | **470.2609** | 235.6341 | 1673.8758 | **837.4415** |
| T | **571.3086** | 286.1579 | 1574.8074 | **787.9073** |
| H | **708.3675** | **354.6874** | **1473.7597** | **737.3835** |
| F | **855.4359** | **428.2216** | **1336.7008** | 668.8540 |
| F | **1002.5043** | **501.7558** | **1189.6324** | 595.3198 |
| A | **1073.5415** | **537.2744** | **1042.5640** | 521.7856 |
| N | **1187.5844** | **594.2958** | **971.5269** | **486.2671** |
| T | **1288.6321** | **644.8197** | **857.4839** | **429.2456** |
| N | 1402.6750 | **701.8411** | **756.4363** | 378.7218 |
| G | **1459.6965** | **730.3519** | **642.3933** | 321.7003 |
| T | 1560.7441 | **780.8757** | **585.3719** | 293.1896 |
| V | 1659.8125 | **830.4099** | **484.3242** | 242.6657 |
| P | 1756.8653 | 878.9363 | **385.2558** | **193.1315** |
| L | 1869.9494 | 935.4783 | **288.2030** | 144.6051 |
| R | 2026.0505 | 1013.5289 | **175.1190** | 88.0631 |

*Peptide sequence 12:* VPSSVTHFFAN[Dea]TNGTVPLR

| Residue | b | b+2 | y | y+2 |
| --- | --- | --- | --- | --- |
| V | 100.0757 | 50.5415 | 2045.0451 | 1023.0262 |
| P | **197.1285** | 99.0679 | 1945.9767 | **973.4920** |
| S | **284.1605** | 142.5839 | 1848.9239 | **924.9656** |
| S | **371.1925** | **186.0999** | 1761.8919 | **881.4496** |
| V | **470.2609** | 235.6341 | 1674.8598 | 837.9336 |
| T | 571.3086 | 286.1579 | 1575.7914 | **788.3993** |
| H | **708.3675** | **354.6874** | **1474.7437** | **737.8755** |
| F | **855.4359** | **428.2216** | **1337.6848** | 669.3461 |
| F | **1002.5043** | **501.7558** | **1190.6164** | 595.8118 |
| A | **1073.5415** | **537.2744** | **1043.5480** | 522.2776 |
| N[Dea] | 1188.5684 | **594.7878** | **972.5109** | **486.7591** |
| T | **1289.6161** | **645.3117** | 857.4839 | 429.2456 |
| N | 1403.6590 | **702.3331** | **756.4363** | 378.7218 |
| G | **1460.6805** | 730.8439 | **642.3933** | 321.7003 |
| T | 1561.7281 | **781.3677** | **585.3719** | 293.1896 |
| V | 1660.7966 | 830.9019 | **484.3242** | 242.6657 |
| P | 1757.8493 | 879.4283 | **385.2558** | 193.1315 |
| L | 1870.9334 | 935.9703 | 288.2030 | 144.6051 |
| R | 2027.0345 | 1014.0209 | **175.1190** | 88.0631 |

*Peptide sequence 13:* TFLAGQDNMLSN[Dea]INTVATR

| Residue | b | b+2 | y | y+2 |
| --- | --- | --- | --- | --- |
| T | 102.0550 | 51.5311 | 2067.0175 | 1034.0124 |
| F | **249.1234** | 125.0653 | 1965.9699 | 983.4886 |
| L | **362.2074** | 181.6074 | 1818.9014 | 909.9544 |
| A | **433.2445** | **217.1259** | 1705.8174 | 853.4123 |
| G | 490.2660 | 245.6366 | 1634.7803 | 817.8938 |
| Q | **618.3246** | 309.6659 | 1577.7588 | 789.3830 |
| D | **733.3515** | 367.1794 | 1449.7002 | 725.3537 |
| N | **847.3945** | 424.2009 | **1334.6733** | 667.8403 |
| M | **978.4349** | 489.7211 | 1220.6303 | 610.8188 |
| L | **1091.5190** | **546.2631** | **1089.5899** | 545.2986 |
| S | **1178.5510** | 589.7792 | **976.5058** | 488.7565 |
| N[Dea] | **1293.5780** | 647.2926 | **889.4738** | **445.2405** |
| I | **1406.6620** | **703.8347** | **774.4468** | 387.7271 |
| N | 1520.7050 | 760.8561 | **661.3628** | 331.1850 |
| T | 1621.7526 | 811.3800 | **547.3198** | 274.1636 |
| V | 1720.8211 | 860.9142 | **446.2722** | 223.6397 |
| A | 1791.8582 | 896.4327 | **347.2037** | 174.1055 |
| T | 1892.9059 | 946.9566 | **276.1666** | 138.5870 |
| R | 2049.0070 | 1025.0071 | **175.1190** | 88.0631 |

*Peptide sequence 14:* VPSSVTHFFANTN[Dea]GTVPLR

| Residue | b | b+2 | y | y+2 |
| --- | --- | --- | --- | --- |
| V | 100.0757 | 50.5415 | 2045.0451 | 1023.0262 |
| P | **197.1285** | 99.0679 | 1945.9767 | **973.4920** |
| S | **284.1605** | 142.5839 | 1848.9239 | **924.9656** |
| S | **371.1925** | **186.0999** | 1761.8919 | **881.4496** |
| V | **470.2609** | 235.6341 | 1674.8598 | 837.9336 |
| T | 571.3086 | 286.1579 | 1575.7914 | **788.3993** |
| H | **708.3675** | **354.6874** | **1474.7437** | **737.8755** |
| F | **855.4359** | **428.2216** | **1337.6848** | 669.3461 |
| F | **1002.5043** | **501.7558** | **1190.6164** | 595.8118 |
| A | **1073.5415** | **537.2744** | **1043.5480** | 522.2776 |
| N | 1187.5844 | **594.2958** | **972.5109** | **486.7591** |
| T | **1288.6321** | **644.8197** | **858.4680** | **429.7376** |
| N[Dea] | 1403.6590 | **702.3331** | **757.4203** | **379.2138** |
| G | **1460.6805** | 730.8439 | **642.3933** | 321.7003 |
| T | 1561.7281 | **781.3677** | **585.3719** | 293.1896 |
| V | 1660.7966 | 830.9019 | **484.3242** | 242.6657 |
| P | 1757.8493 | 879.4283 | **385.2558** | 193.1315 |
| L | 1870.9334 | 935.9703 | 288.2030 | 144.6051 |
| R | 2027.0345 | 1014.0209 | **175.1190** | 88.0631 |

*Peptide sequence 15:* STFGSPFNLLTQDAIYSN[Dea]NFGR

| Residue | b | b+2 | y | y+2 |
| --- | --- | --- | --- | --- |
| S | 88.0393 | 44.5233 | 2450.1623 | 1225.5848 |
| T | **189.0870** | 95.0471 | 2363.1303 | 1182.0688 |
| F | 336.1554 | 168.5813 | 2262.0826 | **1131.5449** |
| G | **393.1769** | 197.0921 | 2115.0142 | 1058.0107 |
| S | **480.2089** | 240.6081 | 2057.9927 | **1029.5000** |
| P | 577.2617 | 289.1345 | 1970.9607 | **985.9840** |
| F | **724.3301** | 362.6687 | 1873.9079 | 937.4576 |
| N | **838.3730** | 419.6901 | 1726.8395 | 863.9234 |
| L | **951.4571** | **476.2322** | 1612.7966 | 806.9019 |
| L | **1064.5411** | 532.7742 | **1499.7125** | 750.3599 |
| T | **1165.5888** | 583.2980 | **1386.6284** | 693.8179 |
| Q | 1293.6474 | 647.3273 | **1285.5808** | **643.2940** |
| D | **1408.6743** | 704.8408 | **1157.5222** | 579.2647 |
| A | **1479.7114** | 740.3594 | **1042.4952** | 521.7513 |
| I | 1592.7955 | 796.9014 | **971.4581** | 486.2327 |
| Y | 1755.8588 | 878.4331 | **858.3741** | **429.6907** |
| S | 1842.8909 | 921.9491 | **695.3107** | **348.1590** |
| N[Dea] | 1957.9178 | 979.4625 | **608.2787** | 304.6430 |
| N | 2071.9607 | 1036.4840 | **493.2518** | 247.1295 |
| F | 2219.0291 | 1110.0182 | **379.2088** | 190.1081 |
| G | 2276.0506 | 1138.5289 | **232.1404** | 116.5738 |
| R | 2432.1517 | 1216.5795 | **175.1190** | 88.0631 |

*Peptide sequence 16:* STFGSPFNLLTQDAIYSNN[Dea]FGR

| Residue | b | b+2 | y | y+2 |
| --- | --- | --- | --- | --- |
| S | **88.0393** | 44.5233 | 2450.1623 | 1225.5848 |
| T | **189.0870** | 95.0471 | 2363.1303 | 1182.0688 |
| F | **336.1554** | 168.5813 | 2262.0826 | 1131.5449 |
| G | **393.1769** | 197.0921 | 2115.0142 | 1058.0107 |
| S | **480.2089** | 240.6081 | 2057.9927 | 1029.5000 |
| P | 577.2617 | 289.1345 | 1970.9607 | 985.9840 |
| F | **724.3301** | 362.6687 | 1873.9079 | 937.4576 |
| N | **838.3730** | 419.6901 | 1726.8395 | 863.9234 |
| L | **951.4571** | **476.2322** | 1612.7966 | 806.9019 |
| L | 1064.5411 | 532.7742 | **1499.7125** | 750.3599 |
| T | **1165.5888** | 583.2980 | 1386.6284 | 693.8179 |
| Q | **1293.6474** | 647.3273 | **1285.5808** | 643.2940 |
| D | **1408.6743** | 704.8408 | 1157.5222 | 579.2647 |
| A | 1479.7114 | 740.3594 | **1042.4952** | 521.7513 |
| I | 1592.7955 | 796.9014 | **971.4581** | 486.2327 |
| Y | 1755.8588 | 878.4331 | **858.3741** | **429.6907** |
| S | 1842.8909 | 921.9491 | **695.3107** | 348.1590 |
| N | 1956.9338 | 978.9705 | **608.2787** | 304.6430 |
| N[Dea] | 2071.9607 | 1036.4840 | **494.2358** | 247.6215 |
| F | 2219.0291 | 1110.0182 | **379.2088** | 190.1081 |
| G | 2276.0506 | 1138.5289 | **232.1404** | 116.5738 |
| R | 2432.1517 | 1216.5795 | **175.1190** | 88.0631 |

*Peptide sequence 17:* IAVPANVPGHFQVFFPAHSGFHQSYFN[Dea]GFSK

| Residue | b | b+2 | y | y+2 |
| --- | --- | --- | --- | --- |
| I | 114.0913 | 57.5493 | 3435.6698 | 1718.3385 |
| A | **185.1285** | 93.0679 | 3322.5857 | 1661.7965 |
| V | **284.1969** | 142.6021 | 3251.5486 | 1626.2779 |
| P | 381.2496 | 191.1285 | 3152.4802 | 1576.7437 |
| A | **452.2867** | 226.6470 | 3055.4274 | 1528.2174 |
| N | **566.3297** | 283.6685 | 2984.3903 | 1492.6988 |
| V | 665.3981 | 333.2027 | 2870.3474 | 1435.6773 |
| P | 762.4509 | 381.7291 | 2771.2790 | **1386.1431** |
| G | 819.4723 | 410.2398 | 2674.2262 | **1337.6167** |
| H | 956.5312 | 478.7693 | 2617.2048 | **1309.1060** |
| F | 1103.5996 | **552.3035** | 2480.1458 | **1240.5766** |
| Q | 1231.6582 | **616.3327** | 2333.0774 | **1167.0424** |
| V | 1330.7266 | 665.8670 | 2205.0189 | **1103.0131** |
| F | 1477.7950 | **739.4012** | 2105.9504 | **1053.4789** |
| F | 1624.8635 | 812.9354 | 1958.8820 | **979.9446** |
| P | 1721.9162 | 861.4618 | 1811.8136 | **906.4104** |
| A | 1792.9533 | 896.9803 | 1714.7608 | **857.8841** |
| H | 1930.0122 | 965.5098 | 1643.7237 | **822.3655** |
| S | 2017.0443 | 1009.0258 | 1506.6648 | **753.8360** |
| G | 2074.0657 | 1037.5365 | **1419.6328** | **710.3200** |
| F | 2221.1342 | 1111.0707 | **1362.6113** | 681.8093 |
| H | 2358.1931 | 1179.6002 | **1215.5429** | **608.2751** |
| Q | 2486.2516 | **1243.6295** | **1078.4840** | **539.7456** |
| S | 2573.2837 | 1287.1455 | **950.4254** | **475.7164** |
| Y | 2736.3470 | 1368.6771 | **863.3934** | **432.2003** |
| F | 2883.4154 | 1442.2113 | **700.3301** | 350.6687 |
| N[Dea] | 2998.4424 | 1499.7248 | **553.2617** | **277.1345** |
| G | 3055.4638 | 1528.2355 | **438.2347** | 219.6210 |
| F | 3202.5322 | 1601.7698 | **381.2132** | 191.1103 |
| S | 3289.5643 | 1645.2858 | **234.1448** | 117.5761 |
| K | 3417.6592 | 1709.3333 | **147.1128** | 74.0600 |

*Peptide sequence 18:* DVLTASFN[Dea]IPEELLGR

| Residue | b | b+2 | y | y+2 |
| --- | --- | --- | --- | --- |
| D | 116.0342 | 58.5207 | 1774.9222 | 887.9647 |
| V | **215.1026** | 108.0550 | 1659.8952 | 830.4512 |
| L | **328.1867** | 164.5970 | 1560.8268 | **780.9170** |
| T | **429.2344** | **215.1208** | 1447.7427 | 724.3750 |
| A | 500.2715 | 250.6394 | **1346.6951** | 673.8512 |
| S | **587.3035** | 294.1554 | 1275.6579 | **638.3326** |
| F | **734.3719** | 367.6896 | **1188.6259** | 594.8166 |
| N[Dea] | 849.3989 | 425.2031 | **1041.5575** | 521.2824 |
| I | 962.4829 | 481.7451 | **926.5306** | 463.7689 |
| P | 1059.5357 | 530.2715 | **813.4465** | **407.2269** |
| E | 1188.5783 | 594.7928 | **716.3937** | 358.7005 |
| E | 1317.6209 | 659.3141 | **587.3511** | 294.1792 |
| L | 1430.7050 | 715.8561 | **458.3085** | 229.6579 |
| L | 1543.7890 | 772.3981 | **345.2245** | 173.1159 |
| G | 1600.8105 | 800.9089 | **232.1404** | 116.5738 |
| R | 1756.9116 | 878.9594 | 175.1190 | 88.0631 |

*Peptide sequence 19:* STFGSPFN[Dea]LLTQDAIYSNNFGR

| Residue | b | b+2 | y | y+2 |
| --- | --- | --- | --- | --- |
| S | 88.0393 | 44.5233 | 2450.1623 | 1225.5848 |
| T | **189.0870** | 95.0471 | 2363.1303 | 1182.0688 |
| F | **336.1554** | 168.5813 | 2262.0826 | **1131.5449** |
| G | **393.1769** | 197.0921 | 2115.0142 | 1058.0107 |
| S | **480.2089** | 240.6081 | 2057.9927 | **1029.5000** |
| P | **577.2617** | 289.1345 | 1970.9607 | **985.9840** |
| F | **724.3301** | 362.6687 | 1873.9079 | 937.4576 |
| N[Dea] | **839.3570** | 420.1821 | 1726.8395 | 863.9234 |
| L | **952.4411** | **476.7242** | 1611.8125 | **806.4099** |
| L | **1065.5251** | 533.2662 | **1498.7285** | 749.8679 |
| T | **1166.5728** | 583.7900 | **1385.6444** | **693.3258** |
| Q | **1294.6314** | 647.8193 | **1284.5967** | 642.8020 |
| D | **1409.6583** | **705.3328** | **1156.5382** | 578.7727 |
| A | **1480.6955** | **740.8514** | **1041.5112** | 521.2592 |
| I | 1593.7795 | **797.3934** | **970.4741** | 485.7407 |
| Y | 1756.8428 | 878.9251 | **857.3900** | **429.1987** |
| S | 1843.8749 | 922.4411 | **694.3267** | 347.6670 |
| N | 1957.9178 | 979.4625 | **607.2947** | 304.1510 |
| N | 2071.9607 | 1036.4840 | **493.2518** | 247.1295 |
| F | 2219.0291 | 1110.0182 | **379.2088** | 190.1081 |
| G | 2276.0506 | 1138.5289 | **232.1404** | 116.5738 |
| R | 2432.1517 | 1216.5795 | **175.1190** | 88.0631 |

*Peptide sequence 20:* STFGSPFNLLTQ[Dea]DAIYSNNFGR

| Residue | b | b+2 | y | y+2 |
| --- | --- | --- | --- | --- |
| S | 88.0393 | 44.5233 | 2450.1623 | 1225.5848 |
| T | **189.0870** | 95.0471 | 2363.1303 | 1182.0688 |
| F | **336.1554** | 168.5813 | 2262.0826 | **1131.5449** |
| G | **393.1769** | 197.0921 | 2115.0142 | 1058.0107 |
| S | **480.2089** | 240.6081 | 2057.9927 | **1029.5000** |
| P | 577.2617 | 289.1345 | 1970.9607 | **985.9840** |
| F | **724.3301** | 362.6687 | 1873.9079 | 937.4576 |
| N | **838.3730** | 419.6901 | 1726.8395 | 863.9234 |
| L | **951.4571** | **476.2322** | 1612.7966 | 806.9019 |
| L | **1064.5411** | 532.7742 | **1499.7125** | 750.3599 |
| T | **1165.5888** | 583.2980 | **1386.6284** | 693.8179 |
| Q[Dea] | 1294.6314 | 647.8193 | **1285.5808** | 643.2940 |
| D | **1409.6583** | **705.3328** | **1156.5382** | 578.7727 |
| A | **1480.6955** | **740.8514** | **1041.5112** | 521.2592 |
| I | 1593.7795 | 797.3934 | **970.4741** | 485.7407 |
| Y | 1756.8428 | 878.9251 | **857.3900** | **429.1987** |
| S | 1843.8749 | 922.4411 | **694.3267** | 347.6670 |
| N | 1957.9178 | 979.4625 | **607.2947** | 304.1510 |
| N | 2071.9607 | 1036.4840 | **493.2518** | **247.1295** |
| F | 2219.0291 | 1110.0182 | **379.2088** | 190.1081 |
| G | 2276.0506 | 1138.5289 | **232.1404** | 116.5738 |
| R | 2432.1517 | 1216.5795 | **175.1190** | 88.0631 |

*Peptide sequence 21:* FLFQEM[Oxi]QPNTFLVPHHLDADYVFLVVQGK

| Residue | b | b+2 | y | y+2 |
| --- | --- | --- | --- | --- |
| F | 148.0757 | 74.5415 | 3448.7398 | 1724.8736 |
| L | **261.1598** | 131.0835 | 3301.6714 | 1651.3394 |
| F | **408.2282** | 204.6177 | 3188.5874 | 1594.7973 |
| Q | **536.2867** | 268.6470 | 3041.5190 | 1521.2631 |
| E | **665.3293** | **333.1683** | 2913.4604 | 1457.2338 |
| M[Oxi] | **812.3647** | 406.6860 | 2784.4178 | 1392.7125 |
| Q | **940.4233** | 470.7153 | 2637.3824 | 1319.1948 |
| P | 1037.4761 | **519.2417** | 2509.3238 | **1255.1655** |
| N | **1151.5190** | 576.2631 | 2412.2710 | **1206.6392** |
| T | **1252.5667** | **626.7870** | 2298.2281 | **1149.6177** |
| F | **1399.6351** | 700.3212 | 2197.1804 | **1099.0939** |
| L | 1512.7192 | 756.8632 | 2050.1120 | **1025.5596** |
| V | 1611.7876 | 806.3974 | 1937.0280 | **969.0176** |
| P | 1708.8403 | 854.9238 | 1837.9595 | **919.4834** |
| H | 1845.8993 | 923.4533 | 1740.9068 | **870.9570** |
| H | 1982.9582 | 991.9827 | 1603.8479 | **802.4276** |
| L | 2096.0422 | 1048.5248 | **1466.7890** | 733.8981 |
| D | 2211.0692 | 1106.0382 | **1353.7049** | 677.3561 |
| A | 2282.1063 | 1141.5568 | **1238.6780** | 619.8426 |
| D | 2397.1332 | **1199.0703** | **1167.6408** | **584.3241** |
| Y | 2560.1966 | **1280.6019** | **1052.6139** | 526.8106 |
| V | 2659.2650 | **1330.1361** | **889.5506** | 445.2789 |
| F | 2806.3334 | **1403.6703** | **790.4822** | **395.7447** |
| L | 2919.4174 | 1460.2124 | **643.4137** | 322.2105 |
| V | 3018.4859 | 1509.7466 | **530.3297** | 265.6685 |
| V | 3117.5543 | 1559.2808 | **431.2613** | 216.1343 |
| Q | 3245.6129 | 1623.3101 | **332.1928** | 166.6001 |
| G | 3302.6343 | 1651.8208 | **204.1343** | 102.5708 |
| K | 3430.7293 | 1715.8683 | **147.1128** | 74.0600 |

# Sample Pool 2

## Protein: B.rapa.Ro18.Candidate-1, 11 peptides (95%)

*Protein sequence coverage*

MEKNKRIFTFLLVIVFFHGVMMMRSIGYEGEEEQGGGGRERGGFMMKESRQVIKSEGGEMRVVISPR**GRIIEKPMHIGFLTMEPKTLFVPQYLDSNLLIFIR**QGEATLGVICKDEFGEKR**LKGGDIYWIPAGSAFYLLNTGR**GQRLHVICSIDPSQSLGFETFQPFYIGGGPSSVLAGFDPDTITSALNVSRPEVQQLMTSQVRGPIVHITEHAPTMWTDFLGLRGEEKHKHLKKLLELKQGTSQEQEDNPWWSWKNIVSSILDVTGEKNRGSGSSKCEDSYNIYDR**KNDFENDYGWSKALDYDDYEPLR**YSGVGVYLVNLTAGSMMAPHMNPTATEYGIVLSGSGEIQVVLPNGTSAMNMRVSPGDVFWIPRYFAFCQIASR**IAPFEFVGFTTSAYKNRPQFLVGSNSLLR**SLNLTSLAMAFGVDEGTMKRFVEAQREAVILPTASAAPPHEGEPERFGSDHIFT

*Peptide sequence 1:* ALDYDDYEPLR

| Residue | b | b+2 | y | y+2 |
| --- | --- | --- | --- | --- |
| I | 114.0913 | 57.5493 | 1900.0071 | 950.5072 |
| I | **227.1754** | 114.0913 | 1786.9230 | 893.9652 |
| E | **356.2180** | 178.6126 | 1673.8390 | 837.4231 |
| K | 484.3130 | 242.6601 | 1544.7964 | **772.9018** |
| P | 581.3657 | 291.1865 | **1416.7014** | **708.8543** |
| M[Oxi] | **728.4011** | 364.7042 | 1319.6486 | 660.3280 |
| H | **865.4600** | **433.2337** | **1172.6132** | 586.8103 |
| I | 978.5441 | **489.7757** | **1035.5543** | **518.2808** |
| G | **1035.5656** | **518.2864** | **922.4703** | **461.7388** |
| F | **1182.6340** | **591.8206** | **865.4488** | **433.2280** |
| L | 1295.7180 | **648.3627** | **718.3804** | **359.6938** |
| T | 1396.7657 | **698.8865** | **605.2963** | **303.1518** |
| M | 1527.8062 | **764.4067** | **504.2486** | **252.6280** |
| E | 1656.8488 | 828.9280 | **373.2082** | 187.1077 |
| P | 1753.9016 | 877.4544 | **244.1656** | 122.5864 |
| K | 1881.9965 | **941.5019** | **147.1128** | 74.0600 |

*Peptide sequence 2:* GGDIYWIPAGSAFYLLNTGR

| Residue | b | b+2 | y | y+2 |
| --- | --- | --- | --- | --- |
| G | 58.0287 | 29.5180 | 2171.0920 | 1086.0496 |
| G | **115.0502** | 58.0287 | 2114.0706 | 1057.5389 |
| D | **230.0771** | 115.5422 | 2057.0491 | 1029.0282 |
| I | **343.1612** | 172.0842 | 1942.0221 | 971.5147 |
| Y | **506.2245** | 253.6159 | 1828.9381 | **914.9727** |
| W | **692.3039** | 346.6556 | 1665.8748 | **833.4410** |
| I | **805.3879** | 403.1976 | 1479.7954 | **740.4014** |
| P | **902.4407** | 451.7240 | **1366.7114** | **683.8593** |
| A | **973.4778** | 487.2425 | **1269.6586** | 635.3329 |
| G | **1030.4993** | 515.7533 | **1198.6215** | 599.8144 |
| S | **1117.5313** | 559.2693 | **1141.6000** | 571.3037 |
| A | **1188.5684** | 594.7878 | **1054.5680** | 527.7876 |
| F | **1335.6368** | 668.3220 | **983.5309** | **492.2691** |
| Y | **1498.7001** | 749.8537 | **836.4625** | **418.7349** |
| L | 1611.7842 | **806.3957** | **673.3991** | **337.2032** |
| L | 1724.8683 | 862.9378 | **560.3151** | 280.6612 |
| N | 1838.9112 | 919.9592 | **447.2310** | 224.1191 |
| T | 1939.9589 | 970.4831 | **333.1881** | 167.0977 |
| G | 1996.9803 | 998.9938 | **232.1404** | 116.5738 |
| R | 2153.0815 | 1077.0444 | **175.1190** | 88.0631 |

*Peptide sequence 3:* GRIIEKPMHIGFLTMEPK

| Residue | b | b+2 | y | y+2 |
| --- | --- | --- | --- | --- |
| G | 58.0287 | 29.5180 | 2097.1347 | 1049.0710 |
| R | **214.1299** | 107.5686 | 2040.1133 | 1020.5603 |
| I | **327.2139** | 164.1106 | 1884.0122 | 942.5097 |
| I | **440.2980** | 220.6526 | 1770.9281 | 885.9677 |
| E | **569.3406** | 285.1739 | 1657.8440 | **829.4257** |
| K | **697.4355** | 349.2214 | 1528.8015 | 764.9044 |
| P | 794.4883 | 397.7478 | 1400.7065 | **700.8569** |
| M | **925.5288** | **463.2680** | 1303.6537 | 652.3305 |
| H | 1062.5877 | **531.7975** | **1172.6132** | **586.8103** |
| I | 1175.6718 | **588.3395** | **1035.5543** | **518.2808** |
| G | 1232.6932 | **616.8502** | **922.4703** | 461.7388 |
| F | 1379.7616 | **690.3845** | **865.4488** | 433.2280 |
| L | 1492.8457 | **746.9265** | **718.3804** | 359.6938 |
| T | 1593.8934 | **797.4503** | **605.2963** | **303.1518** |
| M | 1724.9339 | **862.9706** | **504.2486** | 252.6280 |
| E | 1853.9765 | 927.4919 | **373.2082** | 187.1077 |
| P | 1951.0292 | 976.0182 | **244.1656** | 122.5864 |
| K | 2079.1242 | 1040.0657 | **147.1128** | 74.0600 |

*Peptide sequence 4:* IAPFEFVGFTTSAYK

| Residue | b | b+2 | y | y+2 |
| --- | --- | --- | --- | --- |
| I | 114.0913 | 57.5493 | 1677.8523 | **839.4298** |
| A | **185.1285** | 93.0679 | 1564.7682 | **782.8877** |
| P | **282.1812** | 141.5942 | **1493.7311** | **747.3692** |
| F | **429.2496** | 215.1285 | **1396.6783** | **698.8428** |
| E | **558.2922** | 279.6498 | **1249.6099** | **625.3086** |
| F | **705.3606** | 353.1840 | **1120.5673** | **560.7873** |
| V | **804.4291** | 402.7182 | **973.4989** | **487.2531** |
| G | **861.4505** | 431.2289 | **874.4305** | 437.7189 |
| F | **1008.5189** | 504.7631 | **817.4090** | 409.2082 |
| T | **1109.5666** | 555.2869 | **670.3406** | 335.6740 |
| T | **1210.6143** | 605.8108 | **569.2930** | 285.1501 |
| S | 1297.6463 | **649.3268** | **468.2453** | 234.6263 |
| A | **1368.6834** | 684.8454 | **381.2132** | 191.1103 |
| Y | 1531.7468 | 766.3770 | **310.1761** | 155.5917 |
| K | 1659.8417 | **830.4245** | **147.1128** | 74.0600 |

*Peptide sequence 5:* IIEKPMHIGFLTMEPK

| Residue | b | b+2 | y | y+2 |
| --- | --- | --- | --- | --- |
| I | 114.0913 | 57.5493 | 1884.0122 | 942.5097 |
| I | **227.1754** | 114.0913 | 1770.9281 | 885.9677 |
| E | **356.2180** | 178.6126 | 1657.8440 | **829.4257** |
| K | **484.3130** | 242.6601 | 1528.8015 | **764.9044** |
| P | 581.3657 | 291.1865 | 1400.7065 | **700.8569** |
| M | **712.4062** | 356.7067 | 1303.6537 | 652.3305 |
| H | **849.4651** | **425.2362** | **1172.6132** | **586.8103** |
| I | **962.5492** | **481.7782** | **1035.5543** | **518.2808** |
| G | **1019.5706** | **510.2890** | **922.4703** | **461.7388** |
| F | **1166.6391** | **583.8232** | **865.4488** | 433.2280 |
| L | **1279.7231** | **640.3652** | **718.3804** | **359.6938** |
| T | 1380.7708 | **690.8890** | **605.2963** | **303.1518** |
| M | 1511.8113 | **756.4093** | **504.2486** | **252.6280** |
| E | 1640.8539 | **820.9306** | **373.2082** | **187.1077** |
| P | 1737.9066 | 869.4570 | **244.1656** | 122.5864 |
| K | 1866.0016 | 933.5044 | **147.1128** | 74.0600 |

*Peptide sequence 6:* KNDFENDYGWSK

| Residue | b | b+2 | y | y+2 |
| --- | --- | --- | --- | --- |
| K | **129.1022** | 65.0548 | 1502.6546 | 751.8310 |
| N | **243.1452** | 122.0762 | 1374.5597 | 687.7835 |
| D | **358.1721** | 179.5897 | 1260.5168 | 630.7620 |
| F | **505.2405** | 253.1239 | 1145.4898 | 573.2485 |
| E | **634.2831** | **317.6452** | 998.4214 | **499.7143** |
| N | **748.3260** | **374.6667** | **869.3788** | **435.1930** |
| D | **863.3530** | **432.1801** | **755.3359** | **378.1716** |
| Y | **1026.4163** | 513.7118 | **640.3089** | **320.6581** |
| G | **1083.4378** | 542.2225 | **477.2456** | **239.1264** |
| W | 1269.5171 | **635.2622** | **420.2241** | 210.6157 |
| S | 1356.5491 | 678.7782 | **234.1448** | 117.5761 |
| K | 1484.6441 | 742.8257 | **147.1128** | 74.0600 |

*Peptide sequence 7:* LKGGDIYWIPAGSAFYLLNTGR

| Residue | b | b+2 | y | y+2 |
| --- | --- | --- | --- | --- |
| L | 114.0913 | 57.5493 | 2412.2710 | 1206.6392 |
| K | **242.1863** | 121.5968 | 2299.1870 | 1150.0971 |
| G | 299.2078 | 150.1075 | 2171.0920 | 1086.0496 |
| G | 356.2292 | 178.6183 | 2114.0706 | **1057.5389** |
| D | **471.2562** | 236.1317 | 2057.0491 | 1029.0282 |
| I | **584.3402** | 292.6738 | 1942.0221 | **971.5147** |
| Y | **747.4036** | **374.2054** | 1828.9381 | **914.9727** |
| W | **933.4829** | **467.2451** | 1665.8748 | **833.4410** |
| I | **1046.5669** | **523.7871** | **1479.7954** | **740.4014** |
| P | **1143.6197** | **572.3135** | **1366.7114** | **683.8593** |
| A | **1214.6568** | **607.8320** | **1269.6586** | **635.3329** |
| G | 1271.6783 | **636.3428** | **1198.6215** | **599.8144** |
| S | **1358.7103** | **679.8588** | **1141.6000** | **571.3037** |
| A | **1429.7474** | **715.3774** | **1054.5680** | **527.7876** |
| F | 1576.8158 | **788.9116** | **983.5309** | **492.2691** |
| Y | 1739.8792 | **870.4432** | **836.4625** | **418.7349** |
| L | 1852.9632 | **926.9853** | **673.3991** | **337.2032** |
| L | 1966.0473 | **983.5273** | **560.3151** | 280.6612 |
| N | 2080.0902 | **1040.5488** | **447.2310** | 224.1191 |
| T | 2181.1379 | 1091.0726 | **333.1881** | 167.0977 |
| G | 2238.1594 | **1119.5833** | **232.1404** | 116.5738 |
| R | 2394.2605 | 1197.6339 | **175.1190** | 88.0631 |

*Peptide sequence 8:* NRPQFLVGSNSLLR

| Residue | b | b+2 | y | y+2 |
| --- | --- | --- | --- | --- |
| N | **115.0502** | 58.0287 | 1600.8918 | 800.9495 |
| R | **271.1513** | **136.0793** | 1486.8489 | 743.9281 |
| P | **368.2041** | 184.6057 | 1330.7478 | **665.8775** |
| Q | **496.2627** | **248.6350** | 1233.6950 | 617.3511 |
| F | **643.3311** | **322.1692** | **1105.6364** | **553.3218** |
| L | **756.4151** | **378.7112** | **958.5680** | **479.7876** |
| V | **855.4835** | **428.2454** | **845.4839** | **423.2456** |
| G | **912.5050** | **456.7561** | **746.4155** | **373.7114** |
| S | **999.5370** | **500.2722** | **689.3941** | **345.2007** |
| N | **1113.5800** | **557.2936** | **602.3620** | **301.6847** |
| S | **1200.6120** | **600.8096** | **488.3191** | **244.6632** |
| L | **1313.6961** | **657.3517** | **401.2871** | **201.1472** |
| L | 1426.7801 | **713.8937** | **288.2030** | 144.6051 |
| R | 1582.8812 | 791.9443 | **175.1190** | 88.0631 |

*Peptide sequence 9:* TLFVPQYLDSNLLIFIR

| Residue | b | b+2 | y | y+2 |
| --- | --- | --- | --- | --- |
| T | 102.0550 | 51.5311 | 2052.1528 | 1026.5801 |
| L | **215.1390** | 108.0731 | 1951.1051 | 976.0562 |
| F | **362.2074** | 181.6074 | 1838.0211 | 919.5142 |
| V | **461.2758** | 231.1416 | 1690.9527 | 845.9800 |
| P | **558.3286** | 279.6679 | 1591.8843 | **796.4458** |
| Q | **686.3872** | 343.6972 | 1494.8315 | 747.9194 |
| Y | **849.4505** | **425.2289** | **1366.7729** | 683.8901 |
| L | **962.5346** | 481.7709 | **1203.7096** | 602.3584 |
| D | 1077.5615 | 539.2844 | **1090.6255** | 545.8164 |
| S | 1164.5936 | 582.8004 | **975.5986** | 488.3029 |
| N | **1278.6365** | 639.8219 | **888.5665** | 444.7869 |
| L | **1391.7205** | **696.3639** | **774.5236** | 387.7654 |
| L | 1504.8046 | 752.9059 | **661.4396** | **331.2234** |
| I | 1617.8887 | 809.4480 | **548.3555** | 274.6814 |
| F | 1764.9571 | 882.9822 | **435.2714** | 218.1394 |
| I | 1878.0411 | 939.5242 | **288.2030** | 144.6051 |
| R | 2034.1423 | 1017.5748 | **175.1190** | 88.0631 |

*Peptide sequence 10:* IIEKPMHIGFLTM[Oxi]EPK

| Residue | b | b+2 | y | y+2 |
| --- | --- | --- | --- | --- |
| I | 114.0913 | 57.5493 | 1900.0071 | 950.5072 |
| I | **227.1754** | 114.0913 | 1786.9230 | 893.9652 |
| E | **356.2180** | 178.6126 | 1673.8390 | 837.4231 |
| K | 484.3130 | 242.6601 | 1544.7964 | **772.9018** |
| P | 581.3657 | 291.1865 | **1416.7014** | **708.8543** |
| M | **712.4062** | 356.7067 | 1319.6486 | 660.3280 |
| H | **849.4651** | **425.2362** | **1188.6082** | **594.8077** |
| I | 962.5492 | **481.7782** | **1051.5492** | **526.2783** |
| G | **1019.5706** | **510.2890** | **938.4652** | **469.7362** |
| F | **1166.6391** | **583.8232** | **881.4437** | 441.2255 |
| L | 1279.7231 | **640.3652** | **734.3753** | **367.6913** |
| T | 1380.7708 | 690.8890 | **621.2912** | **311.1493** |
| M[Oxi] | 1527.8062 | **764.4067** | **520.2436** | **260.6254** |
| E | 1656.8488 | 828.9280 | **373.2082** | 187.1077 |
| P | 1753.9016 | 877.4544 | **244.1656** | 122.5864 |
| K | 1881.9965 | **941.5019** | **147.1128** | 74.0600 |

*Peptide sequence 11:* IIEKPM[Oxi]HIGFLTMEPK

| Residue | b | b+2 | y | y+2 |
| --- | --- | --- | --- | --- |
| I | 114.0913 | 57.5493 | 1900.0071 | 950.5072 |
| I | **227.1754** | 114.0913 | 1786.9230 | 893.9652 |
| E | **356.2180** | 178.6126 | 1673.8390 | 837.4231 |
| K | 484.3130 | 242.6601 | 1544.7964 | **772.9018** |
| P | 581.3657 | 291.1865 | **1416.7014** | **708.8543** |
| M[Oxi] | **728.4011** | 364.7042 | 1319.6486 | 660.3280 |
| H | **865.4600** | **433.2337** | **1172.6132** | 586.8103 |
| I | 978.5441 | **489.7757** | **1035.5543** | **518.2808** |
| G | **1035.5656** | **518.2864** | **922.4703** | **461.7388** |
| F | **1182.6340** | **591.8206** | **865.4488** | **433.2280** |
| L | 1295.7180 | **648.3627** | **718.3804** | **359.6938** |
| T | 1396.7657 | **698.8865** | **605.2963** | **303.1518** |
| M | 1527.8062 | **764.4067** | **504.2486** | **252.6280** |
| E | 1656.8488 | 828.9280 | **373.2082** | 187.1077 |
| P | 1753.9016 | 877.4544 | **244.1656** | 122.5864 |
| K | 1881.9965 | **941.5019** | **147.1128** | 74.0600 |

## Protein: B.rapa.Ro18.Candidate-2, 12 peptides (95%)

*Protein sequence coverage*

MIRFTVLSFFVVFVLLFACNESSAKTAKYDKSDESVENDDLAAVPSCCGFSSPLLIK**KDQWKPIFANK**FGQISTVQIGDGCGGMGPYKIHSITLEPNALMLPLLLHSDMVFFVDSGSGILNWVEAQATSTEIRLGDVYR**LRPGTVFYLQSKPVDIFLGTK**LKIYAIFSNSQECLHDPCFGAYSSVTDLLFGFDETILK**SAFGVPEEIIGLMR**NRTQPPLIVHDMLTTPGEANTDTDTNTWPLQTRLLKLFSGDASADSVENKKVKKEKKEKKEKKKKPK**KATTFNVFESEPDFQSPNGQTITINRK**DLKVLQGSMVGVSMVNLTQGSMMGPHWNPWACEISVVVRGSGMVRVLRNSISRSSSECKNMR**FKVEKGDIFAVPR**LHPMAQMSFLNDSLVFVGFTTSAK**NNEPQFLAGKNSALWSLDR**EVLAASFNVSSFMIAGLLEAQKEAAVLGCPACAEGELEKLKEDEEKKESPPQQPPQPFQPQPPGEKPQQPPQPFQPQPPQGEPQKPPQGEPQKPPQGEPEGPQKPFQPQPGQGEPQEPQASMETKMRDEERKREEEEAKKEEEERWKQEEKLWPTQPQWED

*Peptide sequence 1:* ATTFNVFESEPDFQSPNGQTITINRK

| Residue | b | b+2 | y | y+2 |
| --- | --- | --- | --- | --- |
| A | 72.0444 | 36.5258 | 2941.4326 | 1471.2200 |
| T | **173.0921** | 87.0497 | 2870.3955 | 1435.7014 |
| T | **274.1397** | 137.5735 | 2769.3478 | 1385.1776 |
| F | **421.2082** | 211.1077 | 2668.3002 | **1334.6537** |
| N | **535.2511** | 268.1292 | 2521.2318 | **1261.1195** |
| V | **634.3195** | 317.6634 | 2407.1888 | 1204.0981 |
| F | 781.3879 | 391.1976 | 2308.1204 | **1154.5638** |
| E | 910.4305 | 455.7189 | 2161.0520 | **1081.0296** |
| S | 997.4625 | 499.2349 | 2032.0094 | **1016.5083** |
| E | **1126.5051** | 563.7562 | 1944.9774 | 972.9923 |
| P | 1223.5579 | 612.2826 | 1815.9348 | **908.4710** |
| D | 1338.5848 | 669.7961 | 1718.8820 | 859.9446 |
| F | 1485.6533 | 743.3303 | 1603.8551 | 802.4312 |
| Q | 1613.7118 | 807.3596 | **1456.7867** | 728.8970 |
| S | 1700.7439 | 850.8756 | **1328.7281** | **664.8677** |
| P | 1797.7966 | 899.4019 | **1241.6961** | **621.3517** |
| N | 1911.8395 | 956.4234 | 1144.6433 | 572.8253 |
| G | 1968.8610 | 984.9341 | 1030.6004 | 515.8038 |
| Q | 2096.9196 | 1048.9634 | 973.5789 | 487.2931 |
| T | 2197.9673 | 1099.4873 | **845.5203** | 423.2638 |
| I | 2311.0513 | 1156.0293 | **744.4726** | 372.7400 |
| T | 2412.0990 | 1206.5531 | **631.3886** | 316.1979 |
| I | 2525.1831 | 1263.0952 | 530.3409 | 265.6741 |
| N | 2639.2260 | 1320.1166 | **417.2568** | 209.1321 |
| R | 2795.3271 | 1398.1672 | 303.2139 | 152.1106 |
| K | 2923.4221 | 1462.2147 | **147.1128** | 74.0600 |

*Peptide sequence 2:* FKVEKGDIFAVPR

| Residue | b | b+2 | y | y+2 |
| --- | --- | --- | --- | --- |
| F | 148.0757 | 74.5415 | 1505.8475 | 753.4274 |
| K | **276.1707** | 138.5890 | 1358.7791 | **679.8932** |
| V | **375.2391** | 188.1232 | 1230.6841 | **615.8457** |
| E | **504.2817** | 252.6445 | **1131.6157** | **566.3115** |
| K | **632.3766** | 316.6919 | **1002.5731** | **501.7902** |
| G | **689.3981** | 345.2027 | **874.4781** | 437.7427 |
| D | **804.4250** | **402.7162** | **817.4567** | 409.2320 |
| I | **917.5091** | **459.2582** | **702.4297** | 351.7185 |
| F | **1064.5775** | **532.7924** | **589.3457** | **295.1765** |
| A | **1135.6146** | **568.3109** | **442.2772** | 221.6423 |
| V | 1234.6830 | 617.8452 | **371.2401** | 186.1237 |
| P | 1331.7358 | 666.3715 | **272.1717** | 136.5895 |
| R | 1487.8369 | 744.4221 | **175.1190** | 88.0631 |

*Peptide sequence 3:* GDIFAVPR

| Residue | b | b+2 | y | y+2 |
| --- | --- | --- | --- | --- |
| G | 58.0287 | 29.5180 | 874.4781 | **437.7427** |
| D | **173.0557** | 87.0315 | **817.4567** | **409.2320** |
| I | **286.1397** | 143.5735 | **702.4297** | **351.7185** |
| F | **433.2082** | 217.1077 | **589.3457** | **295.1765** |
| A | **504.2453** | 252.6263 | **442.2772** | 221.6423 |
| V | **603.3137** | 302.1605 | **371.2401** | 186.1237 |
| P | **700.3665** | 350.6869 | **272.1717** | 136.5895 |
| R | 856.4676 | **428.7374** | **175.1190** | 88.0631 |

*Peptide sequence 4:* KATTFNVFESEPDFQSPNGQTITINR

| Residue | b | b+2 | y | y+2 |
| --- | --- | --- | --- | --- |
| K | **129.1022** | 65.0548 | 2941.4326 | 1471.2200 |
| A | **200.1394** | 100.5733 | 2813.3377 | 1407.1725 |
| T | **301.1870** | 151.0972 | 2742.3006 | 1371.6539 |
| T | **402.2347** | 201.6210 | 2641.2529 | 1321.1301 |
| F | **549.3031** | **275.1552** | 2540.2052 | 1270.6062 |
| N | **663.3461** | **332.1767** | 2393.1368 | 1197.0720 |
| V | **762.4145** | 381.7109 | 2279.0939 | 1140.0506 |
| F | **909.4829** | 455.2451 | 2180.0255 | 1090.5164 |
| E | **1038.5255** | 519.7664 | 2032.9570 | **1016.9822** |
| S | **1125.5575** | **563.2824** | 1903.9144 | 952.4609 |
| E | **1254.6001** | 627.8037 | 1816.8824 | **908.9448** |
| P | 1351.6529 | 676.3301 | 1687.8398 | **844.4236** |
| D | **1466.6798** | **733.8435** | 1590.7871 | 795.8972 |
| F | 1613.7482 | **807.3777** | 1475.7601 | **738.3837** |
| Q | 1741.8068 | **871.4070** | **1328.6917** | **664.8495** |
| S | 1828.8388 | **914.9230** | **1200.6331** | **600.8202** |
| P | 1925.8916 | 963.4494 | **1113.6011** | **557.3042** |
| N | 2039.9345 | **1020.4709** | **1016.5483** | 508.7778 |
| G | 2096.9560 | 1048.9816 | **902.5054** | 451.7563 |
| Q | 2225.0146 | 1113.0109 | **845.4839** | 423.2456 |
| T | 2326.0622 | 1163.5348 | **717.4254** | 359.2163 |
| I | 2439.1463 | 1220.0768 | **616.3777** | 308.6925 |
| T | 2540.1940 | 1270.6006 | **503.2936** | 252.1504 |
| I | 2653.2780 | 1327.1427 | **402.2459** | 201.6266 |
| N | 2767.3210 | 1384.1641 | **289.1619** | **145.0846** |
| R | 2923.4221 | 1462.2147 | **175.1190** | 88.0631 |

*Peptide sequence 5:* KATTFNVFESEPDFQSPNGQTITINRK

| Residue | b | b+2 | y | y+2 |
| --- | --- | --- | --- | --- |
| K | **129.1022** | 65.0548 | 3069.5276 | 1535.2674 |
| A | **200.1394** | 100.5733 | 2941.4326 | 1471.2200 |
| T | **301.1870** | 151.0972 | 2870.3955 | 1435.7014 |
| T | **402.2347** | 201.6210 | 2769.3478 | 1385.1776 |
| F | **549.3031** | 275.1552 | 2668.3002 | 1334.6537 |
| N | **663.3461** | **332.1767** | 2521.2318 | 1261.1195 |
| V | **762.4145** | **381.7109** | 2407.1888 | 1204.0981 |
| F | 909.4829 | **455.2451** | 2308.1204 | **1154.5638** |
| E | **1038.5255** | **519.7664** | 2161.0520 | **1081.0296** |
| S | **1125.5575** | **563.2824** | 2032.0094 | **1016.5083** |
| E | **1254.6001** | **627.8037** | 1944.9774 | **972.9923** |
| P | 1351.6529 | **676.3301** | 1815.9348 | **908.4710** |
| D | **1466.6798** | 733.8435 | 1718.8820 | **859.9446** |
| F | 1613.7482 | **807.3777** | 1603.8551 | **802.4312** |
| Q | 1741.8068 | **871.4070** | **1456.7867** | **728.8970** |
| S | 1828.8388 | 914.9230 | **1328.7281** | **664.8677** |
| P | 1925.8916 | 963.4494 | **1241.6961** | **621.3517** |
| N | 2039.9345 | **1020.4709** | **1144.6433** | **572.8253** |
| G | 2096.9560 | 1048.9816 | **1030.6004** | **515.8038** |
| Q | 2225.0146 | 1113.0109 | **973.5789** | **487.2931** |
| T | 2326.0622 | 1163.5348 | **845.5203** | **423.2638** |
| I | 2439.1463 | 1220.0768 | **744.4726** | **372.7400** |
| T | 2540.1940 | 1270.6006 | **631.3886** | **316.1979** |
| I | 2653.2780 | 1327.1427 | **530.3409** | 265.6741 |
| N | 2767.3210 | 1384.1641 | **417.2568** | 209.1321 |
| R | 2923.4221 | 1462.2147 | **303.2139** | 152.1106 |
| K | 3051.5170 | 1526.2622 | **147.1128** | 74.0600 |

*Peptide sequence 6:* KDQWKPIFANK

| Residue | b | b+2 | y | y+2 |
| --- | --- | --- | --- | --- |
| K | **129.1022** | 65.0548 | 1374.7528 | 687.8801 |
| D | **244.1292** | 122.5682 | 1246.6579 | **623.8326** |
| Q | **372.1878** | 186.5975 | 1131.6309 | **566.3191** |
| W | **558.2671** | 279.6372 | 1003.5724 | 502.2898 |
| K | **686.3620** | **343.6847** | 817.4931 | **409.2502** |
| P | 783.4148 | 392.2110 | **689.3981** | **345.2027** |
| I | 896.4989 | 448.7531 | **592.3453** | 296.6763 |
| F | **1043.5673** | 522.2873 | **479.2613** | 240.1343 |
| A | 1114.6044 | **557.8058** | **332.1928** | 166.6001 |
| N | 1228.6473 | **614.8273** | **261.1557** | **131.0815** |
| K | 1356.7423 | 678.8748 | **147.1128** | 74.0600 |

*Peptide sequence 7:* LRPGTVFYLQSKPVDIFLGTK

| Residue | b | b+2 | y | y+2 |
| --- | --- | --- | --- | --- |
| L | 114.0913 | 57.5493 | 2379.3435 | 1190.1754 |
| R | 270.1925 | 135.5999 | 2266.2594 | 1133.6333 |
| P | 367.2452 | 184.1262 | 2110.1583 | 1055.5828 |
| G | 424.2667 | 212.6370 | 2013.1055 | 1007.0564 |
| T | **525.3144** | 263.1608 | 1956.0841 | 978.5457 |
| V | **624.3828** | 312.6950 | 1855.0364 | 928.0218 |
| F | **771.4512** | **386.2292** | 1755.9680 | **878.4876** |
| Y | **934.5145** | **467.7609** | 1608.8996 | **804.9534** |
| L | **1047.5986** | **524.3029** | 1445.8362 | **723.4218** |
| Q | **1175.6572** | 588.3322 | **1332.7522** | **666.8797** |
| S | 1262.6892 | 631.8482 | **1204.6936** | **602.8504** |
| K | 1390.7841 | 695.8957 | 1117.6616 | 559.3344 |
| P | 1487.8369 | 744.4221 | **989.5666** | **495.2869** |
| V | 1586.9053 | 793.9563 | 892.5138 | 446.7606 |
| D | 1701.9323 | **851.4698** | **793.4454** | 397.2264 |
| I | 1815.0163 | 908.0118 | 678.4185 | 339.7129 |
| F | 1962.0847 | 981.5460 | 565.3344 | 283.1709 |
| L | 2075.1688 | 1038.0880 | **418.2660** | 209.6366 |
| G | 2132.1903 | 1066.5988 | **305.1819** | 153.0946 |
| T | 2233.2380 | 1117.1226 | 248.1605 | 124.5839 |
| K | 2361.3329 | 1181.1701 | **147.1128** | 74.0600 |

*Peptide sequence 8:* NNEPQFLAGK

| Residue | b | b+2 | y | y+2 |
| --- | --- | --- | --- | --- |
| N | **115.0502** | 58.0287 | 1117.5636 | **559.2855** |
| N | **229.0931** | **115.0502** | **1003.5207** | **502.2640** |
| E | **358.1357** | 179.5715 | **889.4778** | **445.2425** |
| P | **455.1885** | 228.0979 | **760.4352** | **380.7212** |
| Q | **583.2471** | 292.1272 | **663.3824** | **332.1949** |
| F | **730.3155** | 365.6614 | **535.3239** | 268.1656 |
| L | **843.3995** | 422.2034 | **388.2554** | 194.6314 |
| A | **914.4367** | 457.7220 | **275.1714** | 138.0893 |
| G | **971.4581** | 486.2327 | **204.1343** | 102.5708 |
| K | 1099.5531 | **550.2802** | **147.1128** | 74.0600 |

*Peptide sequence 9:* NSALWSLDR

| Residue | b | b+2 | y | y+2 |
| --- | --- | --- | --- | --- |
| N | 115.0502 | 58.0287 | 1061.5374 | **531.2724** |
| S | **202.0822** | 101.5448 | **947.4945** | **474.2509** |
| A | **273.1193** | 137.0633 | **860.4625** | **430.7349** |
| L | **386.2034** | 193.6053 | **789.4254** | **395.2163** |
| W | **572.2827** | 286.6450 | **676.3413** | 338.6743 |
| S | **659.3148** | 330.1610 | **490.2620** | 245.6346 |
| L | 772.3988 | 386.7030 | **403.2300** | 202.1186 |
| D | 887.4258 | 444.2165 | **290.1459** | 145.5766 |
| R | 1043.5269 | **522.2671** | **175.1190** | 88.0631 |

*Peptide sequence 10:* SAFGVPEEIIGLMR

| Residue | b | b+2 | y | y+2 |
| --- | --- | --- | --- | --- |
| S | 88.0393 | 44.5233 | 1518.7985 | **759.9029** |
| A | **159.0764** | 80.0418 | **1431.7664** | 716.3869 |
| F | **306.1448** | 153.5761 | **1360.7293** | **680.8683** |
| G | **363.1663** | 182.0868 | **1213.6609** | **607.3341** |
| V | **462.2347** | 231.6210 | **1156.6395** | **578.8234** |
| P | **559.2875** | 280.1474 | **1057.5710** | **529.2892** |
| E | **688.3301** | 344.6687 | **960.5183** | 480.7628 |
| E | **817.3727** | 409.1900 | **831.4757** | **416.2415** |
| I | **930.4567** | 465.7320 | **702.4331** | 351.7202 |
| I | **1043.5408** | 522.2740 | **589.3490** | 295.1782 |
| G | **1100.5623** | 550.7848 | **476.2650** | 238.6361 |
| L | **1213.6463** | **607.3268** | **419.2435** | 210.1254 |
| M | **1344.6868** | 672.8470 | **306.1594** | 153.5834 |
| R | 1500.7879 | **750.8976** | **175.1190** | 88.0631 |

*Peptide sequence 11:* KATTFNVFESEPDFQSPN[Dea]GQTITINRK

| Residue | b | b+2 | y | y+2 |
| --- | --- | --- | --- | --- |
| K | **129.1022** | 65.0548 | 3070.5116 | 1535.7594 |
| A | **200.1394** | 100.5733 | 2942.4167 | 1471.7120 |
| T | **301.1870** | 151.0972 | 2871.3795 | 1436.1934 |
| T | **402.2347** | 201.6210 | 2770.3319 | 1385.6696 |
| F | **549.3031** | 275.1552 | 2669.2842 | 1335.1457 |
| N | **663.3461** | **332.1767** | 2522.2158 | 1261.6115 |
| V | **762.4145** | **381.7109** | 2408.1728 | 1204.5901 |
| F | **909.4829** | **455.2451** | 2309.1044 | **1155.0559** |
| E | **1038.5255** | **519.7664** | 2162.0360 | **1081.5216** |
| S | **1125.5575** | **563.2824** | 2032.9934 | **1017.0004** |
| E | **1254.6001** | 627.8037 | 1945.9614 | 973.4843 |
| P | 1351.6529 | 676.3301 | 1816.9188 | **908.9630** |
| D | **1466.6798** | 733.8435 | 1719.8660 | **860.4367** |
| F | 1613.7482 | 807.3777 | 1604.8391 | **802.9232** |
| Q | 1741.8068 | 871.4070 | **1457.7707** | **729.3890** |
| S | 1828.8388 | 914.9230 | **1329.7121** | **665.3597** |
| P | 1925.8916 | 963.4494 | **1242.6801** | **621.8437** |
| N[Dea] | 2040.9185 | 1020.9629 | **1145.6273** | **573.3173** |
| G | 2097.9400 | 1049.4736 | **1030.6004** | **515.8038** |
| Q | 2225.9986 | 1113.5029 | **973.5789** | **487.2931** |
| T | 2327.0462 | 1164.0268 | **845.5203** | **423.2638** |
| I | 2440.1303 | **1220.5688** | **744.4726** | 372.7400 |
| T | 2541.1780 | 1271.0926 | **631.3886** | **316.1979** |
| I | 2654.2621 | 1327.6347 | **530.3409** | 265.6741 |
| N | 2768.3050 | 1384.6561 | **417.2568** | 209.1321 |
| R | 2924.4061 | 1462.7067 | **303.2139** | 152.1106 |
| K | 3052.5011 | 1526.7542 | **147.1128** | 74.0600 |

*Peptide sequence 12:* SAFGVPEEIIGLM[Oxi]R

| Residue | b | b+2 | y | y+2 |
| --- | --- | --- | --- | --- |
| S | 88.0393 | 44.5233 | 1534.7934 | **767.9003** |
| A | **159.0764** | 80.0418 | 1447.7614 | 724.3843 |
| F | **306.1448** | 153.5761 | **1376.7242** | **688.8658** |
| G | **363.1663** | 182.0868 | **1229.6558** | **615.3316** |
| V | **462.2347** | 231.6210 | **1172.6344** | **586.8208** |
| P | 559.2875 | 280.1474 | **1073.5660** | **537.2866** |
| E | 688.3301 | 344.6687 | **976.5132** | 488.7602 |
| E | **817.3727** | 409.1900 | **847.4706** | 424.2389 |
| I | **930.4567** | 465.7320 | **718.4280** | 359.7176 |
| I | **1043.5408** | 522.2740 | **605.3439** | 303.1756 |
| G | 1100.5623 | 550.7848 | **492.2599** | 246.6336 |
| L | 1213.6463 | 607.3268 | **435.2384** | 218.1228 |
| M[Oxi] | 1360.6817 | 680.8445 | **322.1544** | 161.5808 |
| R | 1516.7828 | **758.8951** | **175.1190** | 88.0631 |

## Protein: B.rapa.Ro18.Candidate-3, 2 peptides (95%)

*Protein sequence coverage*

MTKFTVLPLFVLLFLVLLCTKSWAKSEEFDESSDEENDVAAVPSCCGFSSPLLIKKDQWKPIFGTQFGQISTVQIGEGCGGMGPYKIHSITLEPNALLLPLLLHSDMVFFVESGSGILNWVEAEPTSSEIRRGDVYRLRPGTVFYLQSKPIDIFLGTKLRVYAIFSNTEECLHDPCFGAYSSITDLLFGFDEAILQSAFGVPEEIIGLMTNRTQPPLIVHDMLSTPGEANTYTWQLQVQPRLLKLFAGYVSAAEKKKKEKKTKKAK**TFNVFESEPDFQSPSGR**TITINRKDLEVLSGSMVGVSMVNLTQASMMGPHWNPWACEISIVLKGSGMVRVLRSSISSTSSSSSSSECKNMR**FKVEEGDIFAVPR**LHPMAQMSFINESLVFIGFTTSAR**NNEPQFLAGQR**SALRLLDQEVLAASLNVSSVMIEGLLGAQKDAVVLGCPYCAEGELEKLKVETEMKKRDDERKREEEEAKKEEEERRKREEEEEEEKQWPPLPQQPPE

*Peptide sequence 1:* FKVEEGDIFAVPR

| Residue | b | b+2 | y | y+2 |
| --- | --- | --- | --- | --- |
| F | 148.0757 | 74.5415 | 1506.7951 | 753.9012 |
| K | **276.1707** | 138.5890 | 1359.7267 | 680.3670 |
| V | **375.2391** | 188.1232 | 1231.6317 | **616.3195** |
| E | **504.2817** | 252.6445 | 1132.5633 | 566.7853 |
| E | **633.3243** | 317.1658 | **1003.5207** | 502.2640 |
| G | **690.3457** | 345.6765 | **874.4781** | 437.7427 |
| D | **805.3727** | **403.1900** | **817.4567** | 409.2320 |
| I | **918.4567** | **459.7320** | **702.4297** | **351.7185** |
| F | **1065.5251** | **533.2662** | **589.3457** | **295.1765** |
| A | **1136.5623** | **568.7848** | **442.2772** | **221.6423** |
| V | 1235.6307 | 618.3190 | **371.2401** | 186.1237 |
| P | 1332.6834 | 666.8454 | **272.1717** | 136.5895 |
| R | 1488.7845 | 744.8959 | **175.1190** | 88.0631 |

*Peptide sequence 2:* GDIFAVPR

| Residue | b | b+2 | y | y+2 |
| --- | --- | --- | --- | --- |
| G | 58.0287 | 29.5180 | 874.4781 | **437.7427** |
| D | **173.0557** | 87.0315 | **817.4567** | **409.2320** |
| I | **286.1397** | 143.5735 | **702.4297** | **351.7185** |
| F | **433.2082** | 217.1077 | **589.3457** | **295.1765** |
| A | **504.2453** | 252.6263 | **442.2772** | 221.6423 |
| V | **603.3137** | 302.1605 | **371.2401** | 186.1237 |
| P | **700.3665** | 350.6869 | **272.1717** | 136.5895 |
| R | 856.4676 | **428.7374** | **175.1190** | 88.0631 |

*Peptide sequence 3:* NNEPQFLAGQR

| Residue | b | b+2 | y | y+2 |
| --- | --- | --- | --- | --- |
| N | 115.0502 | 58.0287 | 1273.6284 | **637.3178** |
| N | **229.0931** | 115.0502 | 1159.5854 | 580.2964 |
| E | **358.1357** | 179.5715 | **1045.5425** | **523.2749** |
| P | **455.1885** | 228.0979 | **916.4999** | **458.7536** |
| Q | **583.2471** | 292.1272 | **819.4472** | 410.2272 |
| F | **730.3155** | 365.6614 | **691.3886** | 346.1979 |
| L | **843.3995** | 422.2034 | **544.3202** | 272.6637 |
| A | **914.4367** | 457.7220 | **431.2361** | 216.1217 |
| G | **971.4581** | 486.2327 | **360.1990** | 180.6031 |
| Q | 1099.5167 | 550.2620 | **303.1775** | 152.0924 |
| R | 1255.6178 | **628.3125** | **175.1190** | 88.0631 |

*Peptide sequence 4:* TFNVFESEPDFQSPSGR

| Residue | b | b+2 | y | y+2 |
| --- | --- | --- | --- | --- |
| T | **102.0550** | 51.5311 | 1943.8770 | **972.4421** |
| F | **249.1234** | 125.0653 | 1842.8293 | 921.9183 |
| N | **363.1663** | 182.0868 | 1695.7609 | 848.3841 |
| V | **462.2347** | 231.6210 | 1581.7180 | 791.3626 |
| F | **609.3031** | 305.1552 | **1482.6496** | **741.8284** |
| E | **738.3457** | 369.6765 | **1335.5811** | 668.2942 |
| S | **825.3777** | 413.1925 | **1206.5386** | 603.7729 |
| E | **954.4203** | 477.7138 | **1119.5065** | 560.2569 |
| P | **1051.4731** | 526.2402 | **990.4639** | 495.7356 |
| D | 1166.5000 | 583.7537 | **893.4112** | **447.2092** |
| F | 1313.5685 | 657.2879 | **778.3842** | 389.6958 |
| Q | **1441.6270** | **721.3172** | **631.3158** | **316.1615** |
| S | 1528.6591 | 764.8332 | **503.2572** | 252.1323 |
| P | 1625.7118 | 813.3596 | **416.2252** | 208.6162 |
| S | 1712.7439 | 856.8756 | **319.1724** | 160.0899 |
| G | 1769.7653 | 885.3863 | **232.1404** | 116.5738 |
| R | 1925.8664 | **963.4369** | **175.1190** | 88.0631 |

## Protein: B.rapa.Ro18.Candidate-4, 3 peptides (95%)

*Protein sequence coverage*

MSKFTIIPLCLLTLFLCTNSFSDQNDGVPSSQSPLLVKRHQRTQLVATEFGEISAVHIGEEYTIQFITLEPNALLLPLLLHSDMVFFVHTGSGVLNWVDEEKERTLELKRGDVFRLRYGTVFYLHCNLERDEVPEKLRVYAIFDVGKCLSDQCLGAYSSIRDLLWGFDEKTLR**SAFAVPK**DVFGR**LRDAVKPPLITHAMPK**NRTQGSEEETWGSRLAKLFVRVEDSIVVDEKDMDALKGSSFGVYMVNLTKGSMMGPHWNPNACEISIVLQGEGMIRVVNHPSYQSKNESERFMVEDGDVFVVPQFYPMAQLSFVNSSFMFMGFSTSAK**TNHPQFLVGQNSVLK**IFNRDVLATSFNMRYATVERLLGAQKDGLLLECVSCAEVELSRLMREIEERRRREEEEIERRKREEEEAKRQEEERRRREEEEAERKKKAEEEARKREKEREREEEAAKRREEERRRREEEEAERKRKEEEEARKREEERKREEEAAKKREEERRKREKEEEEARKREEAREREEEEAKKREEERRKREEEEAERKRRAEEEAREREEEEAKKREEEKEAARRREEEREKEEEMAKRREEERQRKEREDVERKKREEEEERKRREEEAMRREEERKREEEAAKRAEEERRKREEEAEHKKRPPPQGPQPPIHH

*Peptide sequence 1:* LRDAVKPPLITHAMPK

| Residue | b | b+2 | y | y+2 |
| --- | --- | --- | --- | --- |
| L | 114.0913 | 57.5493 | 1787.0360 | 894.0216 |
| R | **270.1925** | 135.5999 | 1673.9520 | 837.4796 |
| D | **385.2194** | 193.1133 | 1517.8508 | 759.4291 |
| A | 456.2565 | 228.6319 | 1402.8239 | 701.9156 |
| V | **555.3249** | 278.1661 | 1331.7868 | 666.3970 |
| K | **683.4199** | 342.2136 | 1232.7184 | 616.8628 |
| P | 780.4726 | **390.7400** | **1104.6234** | **552.8153** |
| P | 877.5254 | 439.2663 | **1007.5706** | 504.2890 |
| L | 990.6095 | **495.8084** | **910.5179** | 455.7626 |
| I | 1103.6935 | **552.3504** | **797.4338** | 399.2205 |
| T | **1204.7412** | 602.8742 | **684.3498** | 342.6785 |
| H | 1341.8001 | **671.4037** | 583.3021 | 292.1547 |
| A | 1412.8372 | **706.9223** | **446.2432** | 223.6252 |
| M | 1543.8777 | **772.4425** | **375.2061** | 188.1067 |
| P | 1640.9305 | 820.9689 | **244.1656** | 122.5864 |
| K | 1769.0255 | 885.0164 | **147.1128** | 74.0600 |

*Peptide sequence 2:* SAFAVPK

| Residue | b | b+2 | y | y+2 |
| --- | --- | --- | --- | --- |
| S | 88.0393 | 44.5233 | 719.4087 | **360.2080** |
| A | **159.0764** | 80.0418 | **632.3766** | **316.6919** |
| F | **306.1448** | 153.5761 | **561.3395** | **281.1734** |
| A | **377.1819** | 189.0946 | **414.2711** | 207.6392 |
| V | **476.2504** | 238.6288 | **343.2340** | 172.1206 |
| P | **573.3031** | 287.1552 | **244.1656** | 122.5864 |
| K | 701.3981 | **351.2027** | **147.1128** | 74.0600 |

*Peptide sequence 3:* TNHPQFLVGQNSVLK

| Residue | b | b+2 | y | y+2 |
| --- | --- | --- | --- | --- |
| T | 102.0550 | 51.5311 | 1681.9020 | 841.4547 |
| N | 216.0979 | 108.5526 | 1580.8544 | 790.9308 |
| H | **353.1568** | 177.0820 | 1466.8114 | 733.9094 |
| P | 450.2096 | 225.6084 | 1329.7525 | **665.3799** |
| Q | **578.2681** | 289.6377 | 1232.6997 | 616.8535 |
| F | **725.3365** | **363.1719** | 1104.6412 | 552.8242 |
| L | **838.4206** | **419.7139** | **957.5728** | 479.2900 |
| V | **937.4890** | **469.2482** | **844.4887** | **422.7480** |
| G | **994.5105** | 497.7589 | **745.4203** | **373.2138** |
| Q | 1122.5691 | **561.7882** | **688.3988** | 344.7030 |
| N | 1236.6120 | 618.8096 | **560.3402** | 280.6738 |
| S | 1323.6440 | 662.3257 | **446.2973** | 223.6523 |
| V | 1422.7124 | 711.8599 | 359.2653 | 180.1363 |
| L | 1535.7965 | 768.4019 | **260.1969** | 130.6021 |
| K | 1663.8915 | 832.4494 | **147.1128** | 74.0600 |

## Protein: B.rapa.Ro18.Candidate-5, 18 peptides (95%)

*Protein sequence coverage*

MAINKLTITLFLLISLAVFHCLAFRVEVQEFEPPRQEGQEGPGGGSGEGWDEEATK**NPYHFGQWSFKNFFQSK**DGFVKMLPKFTKRSSTLFRGIENYRFLFQEMQPNTFLVPHHLDADYVFLVVQGK**GVIGFVTDTANESFQITK**GDVVR**VPSSVTHFFANTNGTVPLR**LAK**IAVPANVPGHFQVFFPAHSGFHQSYFNGFSKDVLTASFNIPEELLGRLIRGPQQEVGQGIIRRVSPEQIKELTEHEHATSPSNK**HKDKKDKHKDKDR**STFGSPFNLLTQDAIYSNNFGR**YHEAHPKRFSQLQDLDIAVGWVNMTQGSLFLPQYNSETTFVTFVENGCARYEMASPYTFQGEQQQPWFGPGQEEEVEEEMSGQVHKIVSRVCKGEVFILPAGHPFAILSQDENFVAVGFGIHASNSTR**TFLAGQDNMLSNINTVATRLSFGLGSK**MAEKLFTSQNYSHFAPTTPSHQFPEKPKPSFQSVFNLVGF

*Peptide sequence 1:* DVLTASFNIPEELLGR

| Residue | b | b+2 | y | y+2 |
| --- | --- | --- | --- | --- |
| D | 116.0342 | 58.5207 | 1773.9381 | **887.4727** |
| V | **215.1026** | 108.0550 | 1658.9112 | 829.9592 |
| L | **328.1867** | 164.5970 | 1559.8428 | **780.4250** |
| T | **429.2344** | **215.1208** | **1446.7587** | **723.8830** |
| A | **500.2715** | 250.6394 | **1345.7110** | **673.3592** |
| S | **587.3035** | 294.1554 | **1274.6739** | **637.8406** |
| F | **734.3719** | 367.6896 | **1187.6419** | **594.3246** |
| N | **848.4149** | 424.7111 | **1040.5735** | 520.7904 |
| I | **961.4989** | **481.2531** | **926.5306** | **463.7689** |
| P | **1058.5517** | 529.7795 | **813.4465** | **407.2269** |
| E | 1187.5943 | **594.3008** | **716.3937** | 358.7005 |
| E | **1316.6369** | 658.8221 | **587.3511** | 294.1792 |
| L | **1429.7209** | **715.3641** | **458.3085** | 229.6579 |
| L | 1542.8050 | **771.9061** | **345.2245** | **173.1159** |
| G | 1599.8265 | 800.4169 | **232.1404** | 116.5738 |
| R | 1755.9276 | **878.4674** | **175.1190** | 88.0631 |

*Peptide sequence 2:* ELTEHEHATSPSNK

| Residue | b | b+2 | y | y+2 |
| --- | --- | --- | --- | --- |
| E | 130.0499 | 65.5286 | 1579.7347 | 790.3710 |
| L | **243.1339** | 122.0706 | 1450.6921 | **725.8497** |
| T | 344.1816 | 172.5944 | 1337.6080 | **669.3077** |
| E | 473.2242 | 237.1157 | 1236.5604 | **618.7838** |
| H | **610.2831** | 305.6452 | **1107.5178** | **554.2625** |
| E | **739.3257** | 370.1665 | **970.4588** | **485.7331** |
| H | **876.3846** | **438.6959** | **841.4163** | **421.2118** |
| A | **947.4217** | **474.2145** | **704.3573** | 352.6823 |
| T | 1048.4694 | 524.7383 | **633.3202** | 317.1638 |
| S | 1135.5014 | 568.2544 | **532.2726** | 266.6399 |
| P | 1232.5542 | 616.7807 | **445.2405** | 223.1239 |
| S | 1319.5862 | 660.2968 | 348.1878 | 174.5975 |
| N | 1433.6292 | **717.3182** | **261.1557** | 131.0815 |
| K | 1561.7241 | 781.3657 | **147.1128** | 74.0600 |

*Peptide sequence 3:* GPQQEVGQGIIR

| Residue | b | b+2 | y | y+2 |
| --- | --- | --- | --- | --- |
| G | 58.0287 | 29.5180 | 1281.6910 | **641.3491** |
| P | **155.0815** | 78.0444 | 1224.6695 | **612.8384** |
| Q | **283.1401** | 142.0737 | **1127.6167** | **564.3120** |
| Q | **411.1987** | 206.1030 | **999.5582** | **500.2827** |
| E | **540.2413** | 270.6243 | **871.4996** | 436.2534 |
| V | **639.3097** | 320.1585 | **742.4570** | **371.7321** |
| G | **696.3311** | 348.6692 | **643.3886** | 322.1979 |
| Q | **824.3897** | 412.6985 | **586.3671** | 293.6872 |
| G | **881.4112** | **441.2092** | **458.3085** | 229.6579 |
| I | **994.4952** | 497.7513 | **401.2871** | **201.1472** |
| I | **1107.5793** | 554.2933 | **288.2030** | 144.6051 |
| R | 1263.6804 | **632.3438** | **175.1190** | 88.0631 |

*Peptide sequence 4:* GPQQEVGQGIIRR

| Residue | b | b+2 | y | y+2 |
| --- | --- | --- | --- | --- |
| G | 58.0287 | 29.5180 | 1437.7921 | 719.3997 |
| P | **155.0815** | 78.0444 | 1380.7706 | 690.8889 |
| Q | **283.1401** | 142.0737 | 1283.7179 | **642.3626** |
| Q | **411.1987** | 206.1030 | 1155.6593 | **578.3333** |
| E | **540.2413** | 270.6243 | **1027.6007** | **514.3040** |
| V | **639.3097** | 320.1585 | 898.5581 | **449.7827** |
| G | 696.3311 | 348.6692 | 799.4897 | **400.2485** |
| Q | 824.3897 | 412.6985 | 742.4682 | 371.7378 |
| G | **881.4112** | 441.2092 | 614.4097 | 307.7085 |
| I | 994.4952 | 497.7513 | 557.3882 | 279.1977 |
| I | 1107.5793 | 554.2933 | 444.3041 | 222.6557 |
| R | 1263.6804 | 632.3438 | 331.2201 | 166.1137 |
| R | 1419.7815 | 710.3944 | **175.1190** | 88.0631 |

*Peptide sequence 5:* GVIGFVTDTANESFQITK

| Residue | b | b+2 | y | y+2 |
| --- | --- | --- | --- | --- |
| G | 58.0287 | 29.5180 | 1926.9807 | **963.9940** |
| V | **157.0972** | 79.0522 | 1869.9593 | 935.4833 |
| I | **270.1812** | 135.5942 | 1770.8909 | **885.9491** |
| G | **327.2027** | 164.1050 | 1657.8068 | **829.4070** |
| F | **474.2711** | 237.6392 | 1600.7853 | **800.8963** |
| V | **573.3395** | 287.1734 | **1453.7169** | **727.3621** |
| T | **674.3872** | 337.6972 | **1354.6485** | **677.8279** |
| D | **789.4141** | 395.2107 | **1253.6008** | 627.3040 |
| T | **890.4618** | 445.7345 | **1138.5739** | 569.7906 |
| A | **961.4989** | 481.2531 | **1037.5262** | 519.2667 |
| N | **1075.5419** | 538.2746 | **966.4891** | 483.7482 |
| E | **1204.5844** | 602.7959 | **852.4462** | 426.7267 |
| S | 1291.6165 | 646.3119 | **723.4036** | 362.2054 |
| F | **1438.6849** | 719.8461 | **636.3715** | 318.6894 |
| Q | 1566.7435 | 783.8754 | **489.3031** | 245.1552 |
| I | 1679.8275 | 840.4174 | **361.2445** | 181.1259 |
| T | 1780.8752 | 890.9412 | **248.1605** | 124.5839 |
| K | 1908.9702 | **954.9887** | **147.1128** | 74.0600 |

*Peptide sequence 6:* IAVPANVPGHFQVFFPAHSGFHQSYFNGFSK

| Residue | b | b+2 | y | y+2 |
| --- | --- | --- | --- | --- |
| I | 114.0913 | 57.5493 | 3434.6858 | 1717.8465 |
| A | **185.1285** | 93.0679 | 3321.6017 | 1661.3045 |
| V | **284.1969** | 142.6021 | 3250.5646 | 1625.7859 |
| P | 381.2496 | 191.1285 | 3151.4962 | 1576.2517 |
| A | **452.2867** | 226.6470 | 3054.4434 | 1527.7253 |
| N | **566.3297** | 283.6685 | 2983.4063 | **1492.2068** |
| V | 665.3981 | 333.2027 | 2869.3634 | 1435.1853 |
| P | 762.4509 | 381.7291 | 2770.2950 | **1385.6511** |
| G | 819.4723 | 410.2398 | 2673.2422 | **1337.1247** |
| H | 956.5312 | 478.7693 | 2616.2207 | **1308.6140** |
| F | 1103.5996 | **552.3035** | 2479.1618 | **1240.0846** |
| Q | 1231.6582 | **616.3327** | 2332.0934 | **1166.5503** |
| V | 1330.7266 | 665.8670 | 2204.0348 | **1102.5211** |
| F | 1477.7950 | **739.4012** | 2104.9664 | **1052.9868** |
| F | 1624.8635 | 812.9354 | 1957.8980 | **979.4526** |
| P | 1721.9162 | 861.4618 | 1810.8296 | **905.9184** |
| A | 1792.9533 | 896.9803 | 1713.7768 | **857.3921** |
| H | 1930.0122 | 965.5098 | 1642.7397 | **821.8735** |
| S | 2017.0443 | 1009.0258 | 1505.6808 | **753.3440** |
| G | 2074.0657 | 1037.5365 | **1418.6488** | **709.8280** |
| F | 2221.1342 | 1111.0707 | **1361.6273** | **681.3173** |
| H | 2358.1931 | 1179.6002 | **1214.5589** | **607.7831** |
| Q | 2486.2516 | 1243.6295 | **1077.5000** | **539.2536** |
| S | 2573.2837 | 1287.1455 | **949.4414** | **475.2243** |
| Y | 2736.3470 | 1368.6771 | **862.4094** | **431.7083** |
| F | 2883.4154 | 1442.2113 | **699.3461** | 350.1767 |
| N | 2997.4583 | 1499.2328 | **552.2776** | 276.6425 |
| G | 3054.4798 | 1527.7435 | **438.2347** | 219.6210 |
| F | 3201.5482 | 1601.2777 | **381.2132** | 191.1103 |
| S | 3288.5802 | 1644.7938 | **234.1448** | 117.5761 |
| K | 3416.6752 | 1708.8412 | **147.1128** | 74.0600 |

*Peptide sequence 7:* LIRGPQQEVGQGIIR

| Residue | b | b+2 | y | y+2 |
| --- | --- | --- | --- | --- |
| L | 114.0913 | 57.5493 | 1663.9602 | 832.4837 |
| I | **227.1754** | 114.0913 | 1550.8761 | **775.9417** |
| R | **383.2765** | 192.1419 | 1437.7921 | **719.3997** |
| G | **440.2980** | 220.6526 | 1281.6910 | **641.3491** |
| P | **537.3507** | 269.1790 | 1224.6695 | 612.8384 |
| Q | **665.4093** | **333.2083** | **1127.6167** | 564.3120 |
| Q | **793.4679** | **397.2376** | **999.5582** | **500.2827** |
| E | **922.5105** | **461.7589** | **871.4996** | **436.2534** |
| V | **1021.5789** | **511.2931** | **742.4570** | **371.7321** |
| G | **1078.6004** | **539.8038** | **643.3886** | **322.1979** |
| Q | **1206.6589** | **603.8331** | **586.3671** | **293.6872** |
| G | **1263.6804** | **632.3438** | **458.3085** | 229.6579 |
| I | 1376.7645 | **688.8859** | **401.2871** | **201.1472** |
| I | 1489.8485 | **745.4279** | **288.2030** | 144.6051 |
| R | 1645.9496 | 823.4785 | **175.1190** | 88.0631 |

*Peptide sequence 8:* NPYHFGQWSFK

| Residue | b | b+2 | y | y+2 |
| --- | --- | --- | --- | --- |
| N | 115.0502 | 58.0287 | 1410.6589 | 705.8331 |
| P | **212.1030** | 106.5551 | 1296.6160 | **648.8116** |
| Y | **375.1663** | 188.0868 | **1199.5633** | **600.2853** |
| H | **512.2252** | 256.6162 | **1036.4999** | **518.7536** |
| F | **659.2936** | 330.1504 | **899.4410** | **450.2241** |
| G | **716.3151** | 358.6612 | **752.3726** | 376.6899 |
| Q | **844.3737** | **422.6905** | **695.3511** | 348.1792 |
| W | 1030.4530 | 515.7301 | **567.2926** | **284.1499** |
| S | **1117.4850** | 559.2461 | **381.2132** | 191.1103 |
| F | 1264.5534 | 632.7803 | **294.1812** | 147.5942 |
| K | 1392.6484 | 696.8278 | **147.1128** | 74.0600 |

*Peptide sequence 9:* RVSPEQIK

| Residue | b | b+2 | y | y+2 |
| --- | --- | --- | --- | --- |
| R | **157.1084** | 79.0578 | 956.5524 | **478.7798** |
| V | **256.1768** | 128.5920 | **800.4512** | 400.7293 |
| S | **343.2088** | 172.1081 | **701.3828** | 351.1951 |
| P | **440.2616** | **220.6344** | **614.3508** | 307.6790 |
| E | **569.3042** | **285.1557** | **517.2980** | 259.1527 |
| Q | **697.3628** | **349.1850** | **388.2554** | 194.6314 |
| I | **810.4468** | **405.7271** | **260.1969** | 130.6021 |
| K | 938.5418 | **469.7745** | **147.1128** | 74.0600 |

*Peptide sequence 10:* STFGSPFNLLTQDAIYSNNFGR

| Residue | b | b+2 | y | y+2 |
| --- | --- | --- | --- | --- |
| S | 88.0393 | 44.5233 | 2449.1783 | 1225.0928 |
| T | **189.0870** | 95.0471 | 2362.1462 | 1181.5768 |
| F | **336.1554** | 168.5813 | 2261.0986 | **1131.0529** |
| G | **393.1769** | 197.0921 | 2114.0301 | **1057.5187** |
| S | **480.2089** | 240.6081 | 2057.0087 | **1029.0080** |
| P | **577.2617** | **289.1345** | 1969.9767 | **985.4920** |
| F | **724.3301** | 362.6687 | 1872.9239 | 936.9656 |
| N | **838.3730** | **419.6901** | 1725.8555 | **863.4314** |
| L | **951.4571** | **476.2322** | 1611.8125 | **806.4099** |
| L | **1064.5411** | **532.7742** | **1498.7285** | **749.8679** |
| T | **1165.5888** | **583.2980** | **1385.6444** | **693.3258** |
| Q | **1293.6474** | **647.3273** | **1284.5967** | **642.8020** |
| D | **1408.6743** | **704.8408** | **1156.5382** | **578.7727** |
| A | **1479.7114** | **740.3594** | **1041.5112** | **521.2592** |
| I | 1592.7955 | **796.9014** | **970.4741** | **485.7407** |
| Y | 1755.8588 | **878.4331** | **857.3900** | **429.1987** |
| S | 1842.8909 | 921.9491 | **694.3267** | **347.6670** |
| N | 1956.9338 | 978.9705 | **607.2947** | **304.1510** |
| N | 2070.9767 | **1035.9920** | **493.2518** | **247.1295** |
| F | 2218.0451 | **1109.5262** | **379.2088** | **190.1081** |
| G | 2275.0666 | 1138.0369 | **232.1404** | 116.5738 |
| R | 2431.1677 | 1216.0875 | **175.1190** | 88.0631 |

*Peptide sequence 11:* TFLAGQDNMLSNINTVATR

| Residue | b | b+2 | y | y+2 |
| --- | --- | --- | --- | --- |
| T | 102.0550 | 51.5311 | 2066.0335 | 1033.5204 |
| F | **249.1234** | 125.0653 | 1964.9858 | 982.9966 |
| L | **362.2074** | 181.6074 | 1817.9174 | **909.4623** |
| A | **433.2445** | **217.1259** | 1704.8334 | **852.9203** |
| G | **490.2660** | 245.6366 | 1633.7962 | **817.4018** |
| Q | **618.3246** | 309.6659 | 1576.7748 | 788.8910 |
| D | **733.3515** | **367.1794** | **1448.7162** | 724.8617 |
| N | **847.3945** | **424.2009** | **1333.6893** | 667.3483 |
| M | **978.4349** | **489.7211** | **1219.6463** | **610.3268** |
| L | **1091.5190** | **546.2631** | **1088.6058** | **544.8066** |
| S | **1178.5510** | **589.7792** | **975.5218** | **488.2645** |
| N | **1292.5940** | 646.8006 | **888.4898** | **444.7485** |
| I | **1405.6780** | **703.3427** | **774.4468** | **387.7271** |
| N | 1519.7210 | 760.3641 | **661.3628** | **331.1850** |
| T | 1620.7686 | 810.8880 | **547.3198** | 274.1636 |
| V | 1719.8370 | 860.4222 | **446.2722** | 223.6397 |
| A | 1790.8742 | 895.9407 | **347.2037** | 174.1055 |
| T | 1891.9218 | 946.4646 | **276.1666** | 138.5870 |
| R | 2048.0229 | 1024.5151 | **175.1190** | 88.0631 |

*Peptide sequence 12:* VPSSVTHFFANTNGTVPLR

| Residue | b | b+2 | y | y+2 |
| --- | --- | --- | --- | --- |
| V | 100.0757 | 50.5415 | 2044.0610 | 1022.5342 |
| P | **197.1285** | 99.0679 | 1944.9926 | **973.0000** |
| S | **284.1605** | 142.5839 | 1847.9399 | **924.4736** |
| S | **371.1925** | **186.0999** | 1760.9078 | **880.9576** |
| V | 470.2609 | 235.6341 | 1673.8758 | **837.4415** |
| T | 571.3086 | 286.1579 | 1574.8074 | 787.9073 |
| H | **708.3675** | 354.6874 | 1473.7597 | **737.3835** |
| F | **855.4359** | **428.2216** | **1336.7008** | 668.8540 |
| F | **1002.5043** | 501.7558 | **1189.6324** | 595.3198 |
| A | **1073.5415** | **537.2744** | **1042.5640** | 521.7856 |
| N | **1187.5844** | **594.2958** | **971.5269** | **486.2671** |
| T | **1288.6321** | **644.8197** | **857.4839** | 429.2456 |
| N | 1402.6750 | **701.8411** | **756.4363** | 378.7218 |
| G | 1459.6965 | **730.3519** | **642.3933** | 321.7003 |
| T | 1560.7441 | 780.8757 | **585.3719** | 293.1896 |
| V | 1659.8125 | 830.4099 | **484.3242** | 242.6657 |
| P | 1756.8653 | 878.9363 | **385.2558** | 193.1315 |
| L | 1869.9494 | 935.4783 | **288.2030** | 144.6051 |
| R | 2026.0505 | 1013.5289 | **175.1190** | 88.0631 |

*Peptide sequence 13:* STFGSPFNLLTQDAIYSN[Dea]NFGR

| Residue | b | b+2 | y | y+2 |
| --- | --- | --- | --- | --- |
| S | 88.0393 | 44.5233 | 2450.1623 | 1225.5848 |
| T | **189.0870** | 95.0471 | 2363.1303 | 1182.0688 |
| F | **336.1554** | 168.5813 | 2262.0826 | **1131.5449** |
| G | **393.1769** | 197.0921 | 2115.0142 | 1058.0107 |
| S | **480.2089** | 240.6081 | 2057.9927 | **1029.5000** |
| P | **577.2617** | 289.1345 | 1970.9607 | **985.9840** |
| F | **724.3301** | 362.6687 | 1873.9079 | 937.4576 |
| N | **838.3730** | 419.6901 | 1726.8395 | 863.9234 |
| L | **951.4571** | **476.2322** | 1612.7966 | 806.9019 |
| L | **1064.5411** | 532.7742 | **1499.7125** | 750.3599 |
| T | **1165.5888** | **583.2980** | **1386.6284** | **693.8179** |
| Q | **1293.6474** | **647.3273** | **1285.5808** | 643.2940 |
| D | **1408.6743** | **704.8408** | **1157.5222** | 579.2647 |
| A | **1479.7114** | **740.3594** | **1042.4952** | 521.7513 |
| I | 1592.7955 | 796.9014 | **971.4581** | **486.2327** |
| Y | 1755.8588 | **878.4331** | **858.3741** | **429.6907** |
| S | 1842.8909 | 921.9491 | **695.3107** | **348.1590** |
| N[Dea] | 1957.9178 | 979.4625 | **608.2787** | 304.6430 |
| N | 2071.9607 | 1036.4840 | **493.2518** | 247.1295 |
| F | 2219.0291 | 1110.0182 | **379.2088** | **190.1081** |
| G | 2276.0506 | **1138.5289** | **232.1404** | 116.5738 |
| R | 2432.1517 | 1216.5795 | **175.1190** | 88.0631 |

*Peptide sequence 14:* STFGSPFNLLTQDAIYSNN[Dea]FGR

| Residue | b | b+2 | y | y+2 |
| --- | --- | --- | --- | --- |
| S | 88.0393 | 44.5233 | 2450.1623 | 1225.5848 |
| T | **189.0870** | 95.0471 | 2363.1303 | 1182.0688 |
| F | **336.1554** | 168.5813 | 2262.0826 | **1131.5449** |
| G | **393.1769** | 197.0921 | 2115.0142 | 1058.0107 |
| S | **480.2089** | 240.6081 | 2057.9927 | **1029.5000** |
| P | **577.2617** | 289.1345 | 1970.9607 | **985.9840** |
| F | **724.3301** | 362.6687 | 1873.9079 | 937.4576 |
| N | **838.3730** | 419.6901 | 1726.8395 | 863.9234 |
| L | **951.4571** | **476.2322** | 1612.7966 | 806.9019 |
| L | **1064.5411** | 532.7742 | **1499.7125** | 750.3599 |
| T | **1165.5888** | **583.2980** | **1386.6284** | **693.8179** |
| Q | **1293.6474** | **647.3273** | **1285.5808** | 643.2940 |
| D | **1408.6743** | **704.8408** | **1157.5222** | 579.2647 |
| A | **1479.7114** | **740.3594** | **1042.4952** | 521.7513 |
| I | 1592.7955 | 796.9014 | **971.4581** | **486.2327** |
| Y | 1755.8588 | **878.4331** | **858.3741** | **429.6907** |
| S | 1842.8909 | 921.9491 | **695.3107** | **348.1590** |
| N | 1956.9338 | 978.9705 | **608.2787** | 304.6430 |
| N[Dea] | 2071.9607 | 1036.4840 | **494.2358** | 247.6215 |
| F | 2219.0291 | 1110.0182 | **379.2088** | **190.1081** |
| G | 2276.0506 | **1138.5289** | **232.1404** | 116.5738 |
| R | 2432.1517 | 1216.5795 | **175.1190** | 88.0631 |

*Peptide sequence 15:* IAVPANVPGHFQVFFPAHSGFHQSYFN[Dea]GFSK

| Residue | b | b+2 | y | y+2 |
| --- | --- | --- | --- | --- |
| I | 114.0913 | 57.5493 | 3435.6698 | 1718.3385 |
| A | **185.1285** | 93.0679 | 3322.5857 | 1661.7965 |
| V | **284.1969** | 142.6021 | 3251.5486 | 1626.2779 |
| P | 381.2496 | 191.1285 | 3152.4802 | 1576.7437 |
| A | 452.2867 | 226.6470 | 3055.4274 | 1528.2174 |
| N | 566.3297 | 283.6685 | 2984.3903 | 1492.6988 |
| V | **665.3981** | 333.2027 | 2870.3474 | 1435.6773 |
| P | 762.4509 | 381.7291 | 2771.2790 | **1386.1431** |
| G | 819.4723 | 410.2398 | 2674.2262 | 1337.6167 |
| H | 956.5312 | 478.7693 | 2617.2048 | 1309.1060 |
| F | 1103.5996 | 552.3035 | 2480.1458 | **1240.5766** |
| Q | 1231.6582 | 616.3327 | 2333.0774 | **1167.0424** |
| V | 1330.7266 | 665.8670 | 2205.0189 | **1103.0131** |
| F | **1477.7950** | **739.4012** | 2105.9504 | **1053.4789** |
| F | 1624.8635 | 812.9354 | 1958.8820 | **979.9446** |
| P | 1721.9162 | 861.4618 | 1811.8136 | **906.4104** |
| A | 1792.9533 | 896.9803 | 1714.7608 | **857.8841** |
| H | 1930.0122 | 965.5098 | 1643.7237 | **822.3655** |
| S | 2017.0443 | 1009.0258 | 1506.6648 | **753.8360** |
| G | 2074.0657 | 1037.5365 | **1419.6328** | **710.3200** |
| F | 2221.1342 | 1111.0707 | **1362.6113** | 681.8093 |
| H | 2358.1931 | 1179.6002 | **1215.5429** | **608.2751** |
| Q | 2486.2516 | 1243.6295 | **1078.4840** | **539.7456** |
| S | 2573.2837 | 1287.1455 | **950.4254** | 475.7164 |
| Y | 2736.3470 | 1368.6771 | **863.3934** | 432.2003 |
| F | 2883.4154 | 1442.2113 | **700.3301** | 350.6687 |
| N[Dea] | 2998.4424 | 1499.7248 | **553.2617** | **277.1345** |
| G | 3055.4638 | 1528.2355 | **438.2347** | 219.6210 |
| F | 3202.5322 | 1601.7698 | **381.2132** | 191.1103 |
| S | 3289.5643 | 1645.2858 | **234.1448** | 117.5761 |
| K | 3417.6592 | 1709.3333 | **147.1128** | 74.0600 |

*Peptide sequence 16:* IAVPANVPGHFQ[Dea]VFFPAHSGFHQSYFNGFSK

| Residue | b | b+2 | y | y+2 |
| --- | --- | --- | --- | --- |
| I | 114.0913 | 57.5493 | 3435.6698 | 1718.3385 |
| A | **185.1285** | 93.0679 | 3322.5857 | 1661.7965 |
| V | **284.1969** | 142.6021 | 3251.5486 | 1626.2779 |
| P | 381.2496 | 191.1285 | 3152.4802 | 1576.7437 |
| A | 452.2867 | 226.6470 | 3055.4274 | 1528.2174 |
| N | **566.3297** | 283.6685 | 2984.3903 | 1492.6988 |
| V | 665.3981 | 333.2027 | 2870.3474 | 1435.6773 |
| P | 762.4509 | 381.7291 | 2771.2790 | **1386.1431** |
| G | 819.4723 | 410.2398 | 2674.2262 | **1337.6167** |
| H | 956.5312 | 478.7693 | 2617.2048 | **1309.1060** |
| F | 1103.5996 | **552.3035** | 2480.1458 | **1240.5766** |
| Q[Dea] | 1232.6422 | 616.8248 | 2333.0774 | **1167.0424** |
| V | 1331.7106 | **666.3590** | 2204.0348 | **1102.5211** |
| F | 1478.7791 | 739.8932 | 2104.9664 | **1052.9868** |
| F | 1625.8475 | **813.4274** | 1957.8980 | **979.4526** |
| P | 1722.9002 | 861.9538 | 1810.8296 | **905.9184** |
| A | 1793.9374 | 897.4723 | 1713.7768 | **857.3921** |
| H | 1930.9963 | 966.0018 | 1642.7397 | **821.8735** |
| S | 2018.0283 | 1009.5178 | 1505.6808 | **753.3440** |
| G | 2075.0498 | **1038.0285** | **1418.6488** | 709.8280 |
| F | 2222.1182 | 1111.5627 | 1361.6273 | **681.3173** |
| H | 2359.1771 | 1180.0922 | **1214.5589** | **607.7831** |
| Q | 2487.2357 | 1244.1215 | **1077.5000** | **539.2536** |
| S | 2574.2677 | 1287.6375 | **949.4414** | **475.2243** |
| Y | 2737.3310 | 1369.1691 | **862.4094** | 431.7083 |
| F | 2884.3994 | 1442.7034 | **699.3461** | 350.1767 |
| N | 2998.4424 | 1499.7248 | **552.2776** | 276.6425 |
| G | 3055.4638 | 1528.2355 | **438.2347** | 219.6210 |
| F | 3202.5322 | 1601.7698 | **381.2132** | 191.1103 |
| S | 3289.5643 | 1645.2858 | **234.1448** | 117.5761 |
| K | 3417.6592 | 1709.3333 | **147.1128** | 74.0600 |

*Peptide sequence 17:* STFGSPFNLLTQ[Dea]DAIYSNNFGR

| Residue | b | b+2 | y | y+2 |
| --- | --- | --- | --- | --- |
| S | 88.0393 | 44.5233 | 2450.1623 | 1225.5848 |
| T | **189.0870** | 95.0471 | 2363.1303 | 1182.0688 |
| F | 336.1554 | 168.5813 | 2262.0826 | **1131.5449** |
| G | **393.1769** | 197.0921 | 2115.0142 | 1058.0107 |
| S | **480.2089** | 240.6081 | 2057.9927 | **1029.5000** |
| P | 577.2617 | 289.1345 | 1970.9607 | **985.9840** |
| F | **724.3301** | 362.6687 | 1873.9079 | 937.4576 |
| N | **838.3730** | 419.6901 | 1726.8395 | 863.9234 |
| L | **951.4571** | 476.2322 | 1612.7966 | 806.9019 |
| L | **1064.5411** | 532.7742 | **1499.7125** | 750.3599 |
| T | **1165.5888** | **583.2980** | **1386.6284** | **693.8179** |
| Q[Dea] | **1294.6314** | 647.8193 | **1285.5808** | **643.2940** |
| D | **1409.6583** | **705.3328** | **1156.5382** | 578.7727 |
| A | **1480.6955** | **740.8514** | **1041.5112** | 521.2592 |
| I | 1593.7795 | **797.3934** | **970.4741** | 485.7407 |
| Y | 1756.8428 | 878.9251 | **857.3900** | **429.1987** |
| S | 1843.8749 | **922.4411** | **694.3267** | 347.6670 |
| N | 1957.9178 | 979.4625 | **607.2947** | 304.1510 |
| N | 2071.9607 | 1036.4840 | **493.2518** | 247.1295 |
| F | 2219.0291 | 1110.0182 | **379.2088** | 190.1081 |
| G | 2276.0506 | 1138.5289 | **232.1404** | 116.5738 |
| R | 2432.1517 | 1216.5795 | **175.1190** | 88.0631 |

*Peptide sequence 18:* TFLAGQDNM[Oxi]LSNINTVATR

**

| Residue | b | b+2 | y | y+2 |
| --- | --- | --- | --- | --- |
| T | 102.0550 | 51.5311 | 2082.0284 | 1041.5179 |
| F | **249.1234** | 125.0653 | 1980.9808 | 990.9940 |
| L | **362.2074** | 181.6074 | 1833.9123 | 917.4598 |
| A | **433.2445** | 217.1259 | 1720.8283 | 860.9178 |
| G | 490.2660 | 245.6366 | 1649.7912 | 825.3992 |
| Q | **618.3246** | 309.6659 | 1592.7697 | 796.8885 |
| D | 733.3515 | 367.1794 | 1464.7111 | 732.8592 |
| N | 847.3945 | 424.2009 | 1349.6842 | 675.3457 |
| M[Oxi] | **994.4299** | **497.7186** | 1235.6412 | **618.3243** |
| L | **1107.5139** | 554.2606 | **1088.6058** | 544.8066 |
| S | 1194.5459 | 597.7766 | **975.5218** | 488.2645 |
| N | **1308.5889** | 654.7981 | **888.4898** | 444.7485 |
| I | **1421.6729** | 711.3401 | **774.4468** | 387.7271 |
| N | 1535.7159 | 768.3616 | **661.3628** | 331.1850 |
| T | 1636.7635 | 818.8854 | **547.3198** | 274.1636 |
| V | 1735.8320 | 868.4196 | **446.2722** | 223.6397 |
| A | 1806.8691 | 903.9382 | **347.2037** | 174.1055 |
| T | 1907.9168 | 954.4620 | **276.1666** | 138.5870 |
| R | 2064.0179 | 1032.5126 | **175.1190** | 88.0631 |

# Sample Pool 3

## Protein: B.rapa.Ro18.Candidate-1, 17 peptides (95%)

*Protein sequence coverage*

MEKNKRIFTFLLVIVFFHGVMMMRSIGYEGEEEQGGGGRERGGFMMKESR**QVIKSEGGEMR**VVISPR**GRIIEKPMHIGFLTMEPK**TLFVPQYLDSNLLIFIRQGEATLGVICKDEFGEKR**LKGGDIYWIPAGSAFYLLNTGRGQR**LHVICSIDPSQSLGFETFQPFYIGGGPSSVLAGFDPDTITSALNVSRPEVQQLMTSQVRGPIVHITEHAPTMWTDFLGLRGEEKHKHLKKLLELKQGTSQEQEDNPWWSWKNIVSSILDVTGEKNRGSGSSKCEDSYNIYDR**KNDFENDYGWSKALDYDDYEPLR**YSGVGVYLVNLTAGSMMAPHMNPTATEYGIVLSGSGEIQVVLPNGTSAMNMR**VSPGDVFWIPR**YFAFCQIASR**IAPFEFVGFTTSAYKNRPQFLVGSNSLLR**SLNLTSLAMAFGVDEGTMKR**FVEAQR**EAVILPTASAAPPHEGEPERFGSDHIFT

*Peptide sequence 1:* ALDYDDYEPLR

| Residue | b | b+2 | y | y+2 |
| --- | --- | --- | --- | --- |
| A | 72.0444 | 36.5258 | 1369.6270 | 685.3172 |
| L | **185.1285** | 93.0679 | 1298.5899 | 649.7986 |
| D | **300.1554** | 150.5813 | **1185.5059** | **593.2566** |
| Y | **463.2187** | 232.1130 | **1070.4789** | 535.7431 |
| D | **578.2457** | 289.6265 | **907.4156** | **454.2114** |
| D | **693.2726** | 347.1399 | **792.3886** | 396.6980 |
| Y | **856.3359** | 428.6716 | **677.3617** | 339.1845 |
| E | **985.3785** | 493.1929 | **514.2984** | 257.6528 |
| P | 1082.4313 | 541.7193 | **385.2558** | 193.1315 |
| L | 1195.5154 | 598.2613 | 288.2030 | 144.6051 |
| R | 1351.6165 | 676.3119 | **175.1190** | 88.0631 |

*Peptide sequence 2:* GRIIEKPMHIGFLTMEPK

| Residue | b | b+2 | y | y+2 |
| --- | --- | --- | --- | --- |
| G | 58.0287 | 29.5180 | 2097.1347 | 1049.0710 |
| R | **214.1299** | 107.5686 | 2040.1133 | 1020.5603 |
| I | **327.2139** | 164.1106 | 1884.0122 | 942.5097 |
| I | **440.2980** | 220.6526 | 1770.9281 | 885.9677 |
| E | **569.3406** | 285.1739 | 1657.8440 | 829.4257 |
| K | **697.4355** | **349.2214** | 1528.8015 | **764.9044** |
| P | 794.4883 | **397.7478** | 1400.7065 | **700.8569** |
| M | **925.5288** | **463.2680** | **1303.6537** | **652.3305** |
| H | **1062.5877** | **531.7975** | **1172.6132** | **586.8103** |
| I | 1175.6718 | **588.3395** | **1035.5543** | **518.2808** |
| G | 1232.6932 | **616.8502** | **922.4703** | 461.7388 |
| F | 1379.7616 | **690.3845** | **865.4488** | **433.2280** |
| L | 1492.8457 | **746.9265** | **718.3804** | **359.6938** |
| T | 1593.8934 | **797.4503** | **605.2963** | **303.1518** |
| M | 1724.9339 | **862.9706** | **504.2486** | **252.6280** |
| E | 1853.9765 | **927.4919** | **373.2082** | 187.1077 |
| P | 1951.0292 | 976.0182 | **244.1656** | 122.5864 |
| K | 2079.1242 | 1040.0657 | **147.1128** | 74.0600 |

*Peptide sequence 3:* IAPFEFVGFTTSAYK

| Residue | b | b+2 | y | y+2 |
| --- | --- | --- | --- | --- |
| I | 114.0913 | 57.5493 | 1677.8523 | **839.4298** |
| A | **185.1285** | 93.0679 | 1564.7682 | **782.8877** |
| P | **282.1812** | 141.5942 | **1493.7311** | **747.3692** |
| F | **429.2496** | **215.1285** | **1396.6783** | **698.8428** |
| E | **558.2922** | 279.6498 | **1249.6099** | **625.3086** |
| F | **705.3606** | 353.1840 | **1120.5673** | **560.7873** |
| V | **804.4291** | 402.7182 | **973.4989** | **487.2531** |
| G | **861.4505** | 431.2289 | **874.4305** | 437.7189 |
| F | **1008.5189** | **504.7631** | **817.4090** | 409.2082 |
| T | **1109.5666** | 555.2869 | **670.3406** | 335.6740 |
| T | **1210.6143** | **605.8108** | **569.2930** | 285.1501 |
| S | **1297.6463** | **649.3268** | **468.2453** | 234.6263 |
| A | **1368.6834** | 684.8454 | **381.2132** | 191.1103 |
| Y | 1531.7468 | **766.3770** | **310.1761** | 155.5917 |
| K | 1659.8417 | **830.4245** | **147.1128** | 74.0600 |

*Peptide sequence 4:* IAPFEFVGFTTSAYKNRPQFLVGSNSLLR

| Residue | b | b+2 | y | y+2 |
| --- | --- | --- | --- | --- |
| I | 114.0913 | 57.5493 | 3259.7262 | 1630.3668 |
| A | **185.1285** | 93.0679 | 3146.6422 | 1573.8247 |
| P | **282.1812** | 141.5942 | 3075.6051 | 1538.3062 |
| F | **429.2496** | 215.1285 | 2978.5523 | **1489.7798** |
| E | **558.2922** | 279.6498 | 2831.4839 | **1416.2456** |
| F | **705.3606** | 353.1840 | 2702.4413 | **1351.7243** |
| V | **804.4291** | 402.7182 | 2555.3729 | **1278.1901** |
| G | **861.4505** | 431.2289 | 2456.3045 | **1228.6559** |
| F | **1008.5189** | **504.7631** | 2399.2830 | **1200.1451** |
| T | 1109.5666 | 555.2869 | 2252.2146 | **1126.6109** |
| T | 1210.6143 | 605.8108 | 2151.1669 | **1076.0871** |
| S | 1297.6463 | **649.3268** | 2050.1192 | **1025.5633** |
| A | 1368.6834 | 684.8454 | 1963.0872 | **982.0472** |
| Y | 1531.7468 | 766.3770 | 1892.0501 | **946.5287** |
| K | 1659.8417 | 830.4245 | 1728.9868 | **864.9970** |
| N | 1773.8846 | 887.4460 | 1600.8918 | **800.9495** |
| R | 1929.9858 | 965.4965 | **1486.8489** | **743.9281** |
| P | 2027.0385 | 1014.0229 | **1330.7478** | **665.8775** |
| Q | 2155.0971 | 1078.0522 | 1233.6950 | 617.3511 |
| F | 2302.1655 | 1151.5864 | **1105.6364** | 553.3218 |
| L | 2415.2496 | 1208.1284 | **958.5680** | 479.7876 |
| V | 2514.3180 | 1257.6626 | **845.4839** | **423.2456** |
| G | 2571.3395 | 1286.1734 | **746.4155** | 373.7114 |
| S | 2658.3715 | **1329.6894** | **689.3941** | 345.2007 |
| N | 2772.4144 | 1386.7108 | **602.3620** | 301.6847 |
| S | 2859.4464 | 1430.2269 | **488.3191** | 244.6632 |
| L | 2972.5305 | 1486.7689 | **401.2871** | **201.1472** |
| L | 3085.6146 | 1543.3109 | **288.2030** | 144.6051 |
| R | 3241.7157 | 1621.3615 | **175.1190** | 88.0631 |

*Peptide sequence 5:* IIEKPMHIGFLTMEPK

| Residue | b | b+2 | y | y+2 |
| --- | --- | --- | --- | --- |
| I | 114.0913 | 57.5493 | 1884.0122 | 942.5097 |
| I | **227.1754** | 114.0913 | 1770.9281 | 885.9677 |
| E | **356.2180** | 178.6126 | 1657.8440 | 829.4257 |
| K | **484.3130** | 242.6601 | 1528.8015 | **764.9044** |
| P | 581.3657 | 291.1865 | **1400.7065** | **700.8569** |
| M | **712.4062** | 356.7067 | 1303.6537 | 652.3305 |
| H | **849.4651** | **425.2362** | **1172.6132** | **586.8103** |
| I | **962.5492** | **481.7782** | **1035.5543** | **518.2808** |
| G | **1019.5706** | **510.2890** | **922.4703** | **461.7388** |
| F | **1166.6391** | **583.8232** | **865.4488** | 433.2280 |
| L | 1279.7231 | **640.3652** | **718.3804** | **359.6938** |
| T | **1380.7708** | **690.8890** | **605.2963** | **303.1518** |
| M | 1511.8113 | **756.4093** | **504.2486** | **252.6280** |
| E | 1640.8539 | **820.9306** | **373.2082** | **187.1077** |
| P | 1737.9066 | 869.4570 | **244.1656** | 122.5864 |
| K | 1866.0016 | 933.5044 | **147.1128** | 74.0600 |

*Peptide sequence 6:* KNDFENDYGWSK

| Residue | b | b+2 | y | y+2 |
| --- | --- | --- | --- | --- |
| K | **129.1022** | 65.0548 | 1502.6546 | 751.8310 |
| N | **243.1452** | 122.0762 | 1374.5597 | 687.7835 |
| D | **358.1721** | 179.5897 | 1260.5168 | 630.7620 |
| F | **505.2405** | 253.1239 | 1145.4898 | 573.2485 |
| E | **634.2831** | **317.6452** | 998.4214 | **499.7143** |
| N | **748.3260** | **374.6667** | **869.3788** | **435.1930** |
| D | **863.3530** | **432.1801** | **755.3359** | **378.1716** |
| Y | **1026.4163** | 513.7118 | **640.3089** | **320.6581** |
| G | **1083.4378** | 542.2225 | **477.2456** | **239.1264** |
| W | 1269.5171 | **635.2622** | **420.2241** | 210.6157 |
| S | 1356.5491 | 678.7782 | **234.1448** | 117.5761 |
| K | 1484.6441 | 742.8257 | **147.1128** | 74.0600 |

*Peptide sequence 7:* LKGGDIYWIPAGSAFYLLNTGR

| Residue | b | b+2 | y | y+2 |
| --- | --- | --- | --- | --- |
| L | 114.0913 | 57.5493 | 2412.2710 | 1206.6392 |
| K | 242.1863 | 121.5968 | 2299.1870 | 1150.0971 |
| G | 299.2078 | 150.1075 | 2171.0920 | 1086.0496 |
| G | 356.2292 | 178.6183 | 2114.0706 | 1057.5389 |
| D | **471.2562** | 236.1317 | 2057.0491 | 1029.0282 |
| I | **584.3402** | 292.6738 | 1942.0221 | 971.5147 |
| Y | **747.4036** | 374.2054 | 1828.9381 | **914.9727** |
| W | **933.4829** | **467.2451** | 1665.8748 | 833.4410 |
| I | **1046.5669** | 523.7871 | **1479.7954** | **740.4014** |
| P | 1143.6197 | 572.3135 | **1366.7114** | **683.8593** |
| A | **1214.6568** | 607.8320 | **1269.6586** | 635.3329 |
| G | 1271.6783 | 636.3428 | **1198.6215** | 599.8144 |
| S | **1358.7103** | 679.8588 | **1141.6000** | 571.3037 |
| A | **1429.7474** | **715.3774** | **1054.5680** | 527.7876 |
| F | 1576.8158 | **788.9116** | **983.5309** | **492.2691** |
| Y | 1739.8792 | **870.4432** | **836.4625** | 418.7349 |
| L | 1852.9632 | **926.9853** | **673.3991** | 337.2032 |
| L | 1966.0473 | **983.5273** | **560.3151** | 280.6612 |
| N | 2080.0902 | **1040.5488** | **447.2310** | 224.1191 |
| T | 2181.1379 | 1091.0726 | **333.1881** | 167.0977 |
| G | 2238.1594 | 1119.5833 | **232.1404** | 116.5738 |
| R | 2394.2605 | 1197.6339 | **175.1190** | 88.0631 |

*Peptide sequence 8:* LKGGDIYWIPAGSAFYLLNTGRGQR

| Residue | b | b+2 | y | y+2 |
| --- | --- | --- | --- | --- |
| L | 114.0913 | 57.5493 | 2753.4522 | 1377.2297 |
| K | **242.1863** | 121.5968 | 2640.3681 | 1320.6877 |
| G | 299.2078 | 150.1075 | 2512.2732 | 1256.6402 |
| G | **356.2292** | 178.6183 | 2455.2517 | 1228.1295 |
| D | **471.2562** | 236.1317 | 2398.2302 | 1199.6188 |
| I | **584.3402** | 292.6738 | 2283.2033 | 1142.1053 |
| Y | **747.4036** | 374.2054 | 2170.1192 | 1085.5633 |
| W | **933.4829** | **467.2451** | 2007.0559 | **1004.0316** |
| I | **1046.5669** | **523.7871** | 1820.9766 | **910.9919** |
| P | 1143.6197 | **572.3135** | 1707.8925 | **854.4499** |
| A | **1214.6568** | 607.8320 | 1610.8398 | **805.9235** |
| G | 1271.6783 | 636.3428 | 1539.8027 | **770.4050** |
| S | 1358.7103 | 679.8588 | 1482.7812 | **741.8942** |
| A | 1429.7474 | 715.3774 | 1395.7492 | **698.3782** |
| F | 1576.8158 | 788.9116 | **1324.7120** | **662.8597** |
| Y | 1739.8792 | 870.4432 | **1177.6436** | **589.3255** |
| L | 1852.9632 | 926.9853 | **1014.5803** | **507.7938** |
| L | 1966.0473 | 983.5273 | **901.4962** | **451.2518** |
| N | 2080.0902 | 1040.5488 | **788.4122** | 394.7097 |
| T | 2181.1379 | 1091.0726 | 674.3692 | 337.6883 |
| G | 2238.1594 | 1119.5833 | **573.3216** | 287.1644 |
| R | 2394.2605 | **1197.6339** | 516.3001 | 258.6537 |
| G | 2451.2819 | 1226.1446 | **360.1990** | 180.6031 |
| Q | 2579.3405 | 1290.1739 | 303.1775 | 152.0924 |
| R | 2735.4416 | 1368.2245 | **175.1190** | 88.0631 |

*Peptide sequence 9:* NRPQFLVGSNSLLR

| Residue | b | b+2 | y | y+2 |
| --- | --- | --- | --- | --- |
| N | 115.0502 | 58.0287 | 1600.8918 | 800.9495 |
| R | **271.1513** | **136.0793** | 1486.8489 | **743.9281** |
| P | **368.2041** | 184.6057 | 1330.7478 | **665.8775** |
| Q | **496.2627** | **248.6350** | **1233.6950** | 617.3511 |
| F | **643.3311** | **322.1692** | **1105.6364** | **553.3218** |
| L | **756.4151** | **378.7112** | **958.5680** | **479.7876** |
| V | **855.4835** | **428.2454** | **845.4839** | **423.2456** |
| G | **912.5050** | **456.7561** | **746.4155** | **373.7114** |
| S | **999.5370** | **500.2722** | **689.3941** | **345.2007** |
| N | **1113.5800** | **557.2936** | **602.3620** | **301.6847** |
| S | **1200.6120** | **600.8096** | **488.3191** | 244.6632 |
| L | **1313.6961** | **657.3517** | **401.2871** | **201.1472** |
| L | 1426.7801 | **713.8937** | **288.2030** | 144.6051 |
| R | 1582.8812 | 791.9443 | **175.1190** | 88.0631 |

*Peptide sequence 10:* VSPGDVFWIPR

| Residue | b | b+2 | y | y+2 |
| --- | --- | --- | --- | --- |
| V | 100.0757 | 50.5415 | 1272.6735 | **636.8404** |
| S | **187.1077** | 94.0575 | **1173.6051** | **587.3062** |
| P | 284.1605 | 142.5839 | **1086.5731** | **543.7902** |
| G | **341.1819** | 171.0946 | **989.5203** | 495.2638 |
| D | **456.2089** | 228.6081 | **932.4989** | 466.7531 |
| V | **555.2773** | 278.1423 | **817.4719** | 409.2396 |
| F | **702.3457** | 351.6765 | **718.4035** | 359.7054 |
| W | **888.4250** | 444.7162 | **571.3351** | 286.1712 |
| I | **1001.5091** | 501.2582 | **385.2558** | 193.1315 |
| P | 1098.5619 | 549.7846 | **272.1717** | 136.5895 |
| R | 1254.6630 | **627.8351** | **175.1190** | 88.0631 |

*Peptide sequence 11:* NRPQFLVGSN[Dea]SLLR

| Residue | b | b+2 | y | y+2 |
| --- | --- | --- | --- | --- |
| N | 115.0502 | 58.0287 | 1601.8758 | 801.4415 |
| R | **271.1513** | 136.0793 | 1487.8329 | 744.4201 |
| P | 368.2041 | 184.6057 | 1331.7318 | 666.3695 |
| Q | **496.2627** | **248.6350** | 1234.6790 | 617.8431 |
| F | **643.3311** | **322.1692** | 1106.6204 | 553.8139 |
| L | **756.4151** | **378.7112** | **959.5520** | 480.2796 |
| V | **855.4835** | **428.2454** | **846.4680** | **423.7376** |
| G | **912.5050** | **456.7561** | **747.3995** | **374.2034** |
| S | **999.5370** | 500.2722 | **690.3781** | 345.6927 |
| N[Dea] | 1114.5640 | 557.7856 | **603.3461** | 302.1767 |
| S | **1201.5960** | 601.3016 | **488.3191** | 244.6632 |
| L | 1314.6801 | **657.8437** | **401.2871** | **201.1472** |
| L | 1427.7641 | 714.3857 | **288.2030** | 144.6051 |
| R | 1583.8653 | 792.4363 | **175.1190** | 88.0631 |

*Peptide sequence 12:* NRPQ[Dea]FLVGSNSLLR

| Residue | b | b+2 | y | y+2 |
| --- | --- | --- | --- | --- |
| N | 115.0502 | 58.0287 | 1601.8758 | 801.4415 |
| R | **271.1513** | **136.0793** | 1487.8329 | 744.4201 |
| P | 368.2041 | 184.6057 | 1331.7318 | 666.3695 |
| Q[Dea] | **497.2467** | 249.1270 | 1234.6790 | 617.8431 |
| F | **644.3151** | **322.6612** | **1105.6364** | 553.3218 |
| L | **757.3991** | **379.2032** | **958.5680** | 479.7876 |
| V | **856.4676** | **428.7374** | **845.4839** | **423.2456** |
| G | 913.4890 | **457.2482** | **746.4155** | **373.7114** |
| S | 1000.5211 | 500.7642 | **689.3941** | **345.2007** |
| N | 1114.5640 | 557.7856 | **602.3620** | 301.6847 |
| S | 1201.5960 | 601.3016 | **488.3191** | 244.6632 |
| L | 1314.6801 | 657.8437 | **401.2871** | **201.1472** |
| L | 1427.7641 | 714.3857 | **288.2030** | 144.6051 |
| R | 1583.8653 | 792.4363 | **175.1190** | 88.0631 |

*Peptide sequence 13:* QVIKSEGGEMR

| Residue | b | b+2 | y | y+2 |
| --- | --- | --- | --- | --- |
| Q | 112.0393 | 56.5233 | 1216.5990 | **608.8032** |
| V | 211.1077 | 106.0575 | 1105.5670 | 553.2871 |
| I | **324.1918** | 162.5995 | **1006.4986** | **503.7529** |
| K | **452.2867** | 226.6470 | **893.4145** | **447.2109** |
| S | **539.3188** | 270.1630 | **765.3196** | 383.1634 |
| E | **668.3614** | 334.6843 | **678.2876** | 339.6474 |
| G | **725.3828** | 363.1951 | **549.2450** | 275.1261 |
| G | 782.4043 | 391.7058 | **492.2235** | 246.6154 |
| E | **911.4469** | 456.2271 | 435.2020 | 218.1047 |
| M | **1042.4874** | 521.7473 | **306.1594** | 153.5834 |
| R | 1198.5885 | **599.7979** | **175.1190** | 88.0631 |

*Peptide sequence 14:* IIEKPMHIGFLTM[Oxi]EPK

| Residue | b | b+2 | y | y+2 |
| --- | --- | --- | --- | --- |
| I | 114.0913 | 57.5493 | 1900.0071 | 950.5072 |
| I | **227.1754** | 114.0913 | 1786.9230 | 893.9652 |
| E | **356.2180** | 178.6126 | 1673.8390 | **837.4231** |
| K | 484.3130 | 242.6601 | 1544.7964 | **772.9018** |
| P | 581.3657 | 291.1865 | 1416.7014 | **708.8543** |
| M | **712.4062** | 356.7067 | 1319.6486 | **660.3280** |
| H | **849.4651** | **425.2362** | 1188.6082 | **594.8077** |
| I | 962.5492 | **481.7782** | **1051.5492** | 526.2783 |
| G | **1019.5706** | **510.2890** | **938.4652** | 469.7362 |
| F | **1166.6391** | **583.8232** | **881.4437** | 441.2255 |
| L | 1279.7231 | **640.3652** | **734.3753** | **367.6913** |
| T | 1380.7708 | 690.8890 | **621.2912** | **311.1493** |
| M[Oxi] | 1527.8062 | **764.4067** | **520.2436** | **260.6254** |
| E | 1656.8488 | 828.9280 | **373.2082** | 187.1077 |
| P | 1753.9016 | 877.4544 | **244.1656** | 122.5864 |
| K | 1881.9965 | **941.5019** | **147.1128** | 74.0600 |

*Peptide sequence 15:* GRIIEKPMHIGFLTM[Oxi]EPK

| Residue | b | b+2 | y | y+2 |
| --- | --- | --- | --- | --- |
| G | 58.0287 | 29.5180 | 2113.1297 | 1057.0685 |
| R | **214.1299** | 107.5686 | 2056.1082 | 1028.5577 |
| I | **327.2139** | 164.1106 | 1900.0071 | 950.5072 |
| I | **440.2980** | 220.6526 | 1786.9230 | 893.9652 |
| E | **569.3406** | 285.1739 | 1673.8390 | 837.4231 |
| K | **697.4355** | 349.2214 | 1544.7964 | 772.9018 |
| P | 794.4883 | 397.7478 | 1416.7014 | **708.8543** |
| M | 925.5288 | 463.2680 | 1319.6486 | 660.3280 |
| H | 1062.5877 | **531.7975** | 1188.6082 | 594.8077 |
| I | 1175.6718 | **588.3395** | **1051.5492** | **526.2783** |
| G | 1232.6932 | **616.8502** | **938.4652** | 469.7362 |
| F | 1379.7616 | **690.3845** | **881.4437** | 441.2255 |
| L | 1492.8457 | **746.9265** | **734.3753** | 367.6913 |
| T | 1593.8934 | 797.4503 | **621.2912** | 311.1493 |
| M[Oxi] | 1740.9288 | 870.9680 | **520.2436** | 260.6254 |
| E | 1869.9714 | 935.4893 | **373.2082** | 187.1077 |
| P | 1967.0241 | 984.0157 | **244.1656** | 122.5864 |
| K | 2095.1191 | 1048.0632 | **147.1128** | 74.0600 |

*Peptide sequence 16:* IIEKPM[Oxi]HIGFLTMEPK

| Residue | b | b+2 | y | y+2 |
| --- | --- | --- | --- | --- |
| I | 114.0913 | 57.5493 | 1900.0071 | 950.5072 |
| I | **227.1754** | 114.0913 | 1786.9230 | 893.9652 |
| E | **356.2180** | 178.6126 | 1673.8390 | 837.4231 |
| K | 484.3130 | 242.6601 | 1544.7964 | **772.9018** |
| P | 581.3657 | 291.1865 | 1416.7014 | **708.8543** |
| M[Oxi] | 728.4011 | 364.7042 | 1319.6486 | 660.3280 |
| H | **865.4600** | **433.2337** | **1172.6132** | 586.8103 |
| I | 978.5441 | **489.7757** | **1035.5543** | **518.2808** |
| G | **1035.5656** | **518.2864** | **922.4703** | **461.7388** |
| F | **1182.6340** | **591.8206** | **865.4488** | **433.2280** |
| L | 1295.7180 | **648.3627** | **718.3804** | **359.6938** |
| T | **1396.7657** | **698.8865** | **605.2963** | **303.1518** |
| M | 1527.8062 | **764.4067** | **504.2486** | **252.6280** |
| E | 1656.8488 | 828.9280 | **373.2082** | 187.1077 |
| P | 1753.9016 | 877.4544 | **244.1656** | 122.5864 |
| K | 1881.9965 | 941.5019 | **147.1128** | 74.0600 |

*Peptide sequence 17:* GRIIEKPM[Oxi]HIGFLTMEPK

| Residue | b | b+2 | y | y+2 |
| --- | --- | --- | --- | --- |
| G | 58.0287 | 29.5180 | 2113.1297 | 1057.0685 |
| R | **214.1299** | 107.5686 | 2056.1082 | 1028.5577 |
| I | 327.2139 | 164.1106 | 1900.0071 | 950.5072 |
| I | **440.2980** | 220.6526 | 1786.9230 | 893.9652 |
| E | 569.3406 | 285.1739 | 1673.8390 | 837.4231 |
| K | **697.4355** | 349.2214 | 1544.7964 | 772.9018 |
| P | 794.4883 | 397.7478 | 1416.7014 | **708.8543** |
| M[Oxi] | 941.5237 | 471.2655 | 1319.6486 | 660.3280 |
| H | 1078.5826 | **539.7949** | 1172.6132 | 586.8103 |
| I | 1191.6667 | 596.3370 | 1035.5543 | 518.2808 |
| G | 1248.6881 | **624.8477** | **922.4703** | 461.7388 |
| F | 1395.7566 | **698.3819** | **865.4488** | 433.2280 |
| L | 1508.8406 | **754.9239** | **718.3804** | **359.6938** |
| T | 1609.8883 | **805.4478** | **605.2963** | **303.1518** |
| M | 1740.9288 | 870.9680 | **504.2486** | 252.6280 |
| E | 1869.9714 | 935.4893 | **373.2082** | 187.1077 |
| P | 1967.0241 | 984.0157 | **244.1656** | 122.5864 |
| K | 2095.1191 | 1048.0632 | **147.1128** | 74.0600 |

## Protein: B.rapa.Ro18.Candidate-2, 4 peptides (95%)

*Protein sequence coverage*

MIRFTVLSFFVVFVLLFACNESSAKTAKYDKSDESVENDDLAAVPSCCGFSSPLLIK**KDQWKPIFANK**FGQISTVQIGDGCGGMGPYKIHSITLEPNALMLPLLLHSDMVFFVDSGSGILNWVEAQATSTEIRLGDVYRLRPGTVFYLQSKPVDIFLGTKLKIYAIFSNSQECLHDPCFGAYSSVTDLLFGFDETILKSAFGVPEEIIGLMRNRTQPPLIVHDMLTTPGEANTDTDTNTWPLQTRLLKLFSGDASADSVENKKVKKEKKEKKEKKKKPK**KATTFNVFESEPDFQSPNGQTITINRK**DLKVLQGSMVGVSMVNLTQGSMMGPHWNPWACEISVVVRGSGMVRVLRNSISRSSSECKNMR**FKVEKGDIFAVPR**LHPMAQMSFLNDSLVFVGFTTSAK**NNEPQFLAGK**NSALWSLDREVLAASFNVSSFMIAGLLEAQKEAAVLGCPACAEGELEKLKEDEEKKESPPQQPPQPFQPQPPGEKPQQPPQPFQPQPPQGEPQKPPQGEPQKPPQGEPEGPQKPFQPQPGQGEPQEPQASMETKMRDEERKREEEEAKKEEEERWKQEEKLWPTQPQWED

*Peptide sequence 1:* FKVEKGDIFAVPR

| Residue | b | b+2 | y | y+2 |
| --- | --- | --- | --- | --- |
| F | 148.0757 | 74.5415 | 1505.8475 | 753.4274 |
| K | **276.1707** | 138.5890 | 1358.7791 | 679.8932 |
| V | **375.2391** | 188.1232 | 1230.6841 | 615.8457 |
| E | **504.2817** | 252.6445 | 1131.6157 | 566.3115 |
| K | 632.3766 | 316.6919 | 1002.5731 | 501.7902 |
| G | **689.3981** | 345.2027 | 874.4781 | 437.7427 |
| D | **804.4250** | **402.7162** | 817.4567 | 409.2320 |
| I | **917.5091** | **459.2582** | 702.4297 | 351.7185 |
| F | **1064.5775** | **532.7924** | **589.3457** | **295.1765** |
| A | **1135.6146** | **568.3109** | **442.2772** | 221.6423 |
| V | 1234.6830 | **617.8452** | **371.2401** | **186.1237** |
| P | 1331.7358 | 666.3715 | **272.1717** | **136.5895** |
| R | 1487.8369 | 744.4221 | **175.1190** | 88.0631 |

*Peptide sequence 2:* KATTFNVFESEPDFQSPNGQTITINRK

| Residue | b | b+2 | y | y+2 |
| --- | --- | --- | --- | --- |
| K | **129.1022** | 65.0548 | 3069.5276 | 1535.2674 |
| A | **200.1394** | 100.5733 | 2941.4326 | 1471.2200 |
| T | **301.1870** | 151.0972 | 2870.3955 | 1435.7014 |
| T | **402.2347** | 201.6210 | 2769.3478 | 1385.1776 |
| F | **549.3031** | 275.1552 | 2668.3002 | 1334.6537 |
| N | **663.3461** | **332.1767** | 2521.2318 | 1261.1195 |
| V | **762.4145** | **381.7109** | 2407.1888 | 1204.0981 |
| F | 909.4829 | **455.2451** | 2308.1204 | **1154.5638** |
| E | **1038.5255** | **519.7664** | 2161.0520 | **1081.0296** |
| S | **1125.5575** | **563.2824** | 2032.0094 | **1016.5083** |
| E | **1254.6001** | **627.8037** | 1944.9774 | **972.9923** |
| P | 1351.6529 | 676.3301 | 1815.9348 | **908.4710** |
| D | **1466.6798** | 733.8435 | 1718.8820 | **859.9446** |
| F | 1613.7482 | **807.3777** | 1603.8551 | **802.4312** |
| Q | 1741.8068 | 871.4070 | **1456.7867** | **728.8970** |
| S | 1828.8388 | 914.9230 | **1328.7281** | **664.8677** |
| P | 1925.8916 | 963.4494 | **1241.6961** | **621.3517** |
| N | 2039.9345 | 1020.4709 | **1144.6433** | **572.8253** |
| G | 2096.9560 | 1048.9816 | **1030.6004** | **515.8038** |
| Q | 2225.0146 | 1113.0109 | **973.5789** | 487.2931 |
| T | 2326.0622 | 1163.5348 | **845.5203** | **423.2638** |
| I | 2439.1463 | 1220.0768 | **744.4726** | **372.7400** |
| T | 2540.1940 | 1270.6006 | **631.3886** | **316.1979** |
| I | 2653.2780 | 1327.1427 | **530.3409** | 265.6741 |
| N | 2767.3210 | 1384.1641 | **417.2568** | 209.1321 |
| R | 2923.4221 | 1462.2147 | **303.2139** | 152.1106 |
| K | 3051.5170 | 1526.2622 | **147.1128** | 74.0600 |

*Peptide sequence 3:* NNEPQFLAGK

| Residue | b | b+2 | y | y+2 |
| --- | --- | --- | --- | --- |
| N | 115.0502 | 58.0287 | 1117.5636 | **559.2855** |
| N | **229.0931** | 115.0502 | **1003.5207** | **502.2640** |
| E | **358.1357** | 179.5715 | **889.4778** | **445.2425** |
| P | 455.1885 | 228.0979 | **760.4352** | **380.7212** |
| Q | **583.2471** | 292.1272 | **663.3824** | 332.1949 |
| F | **730.3155** | 365.6614 | **535.3239** | 268.1656 |
| L | **843.3995** | 422.2034 | **388.2554** | 194.6314 |
| A | **914.4367** | 457.7220 | **275.1714** | 138.0893 |
| G | 971.4581 | 486.2327 | **204.1343** | 102.5708 |
| K | 1099.5531 | **550.2802** | **147.1128** | 74.0600 |

*Peptide sequence 4:* KATTFNVFESEPDFQSPN[Dea]GQTITINRK

| Residue | b | b+2 | y | y+2 |
| --- | --- | --- | --- | --- |
| K | **129.1022** | 65.0548 | 3070.5116 | 1535.7594 |
| A | **200.1394** | 100.5733 | 2942.4167 | 1471.7120 |
| T | **301.1870** | 151.0972 | 2871.3795 | 1436.1934 |
| T | **402.2347** | 201.6210 | 2770.3319 | 1385.6696 |
| F | **549.3031** | 275.1552 | 2669.2842 | 1335.1457 |
| N | **663.3461** | **332.1767** | 2522.2158 | 1261.6115 |
| V | **762.4145** | **381.7109** | 2408.1728 | 1204.5901 |
| F | **909.4829** | **455.2451** | 2309.1044 | **1155.0559** |
| E | **1038.5255** | **519.7664** | 2162.0360 | **1081.5216** |
| S | **1125.5575** | **563.2824** | 2032.9934 | **1017.0004** |
| E | **1254.6001** | 627.8037 | 1945.9614 | **973.4843** |
| P | 1351.6529 | 676.3301 | 1816.9188 | **908.9630** |
| D | **1466.6798** | 733.8435 | 1719.8660 | **860.4367** |
| F | 1613.7482 | 807.3777 | 1604.8391 | **802.9232** |
| Q | 1741.8068 | 871.4070 | **1457.7707** | **729.3890** |
| S | 1828.8388 | 914.9230 | **1329.7121** | **665.3597** |
| P | 1925.8916 | 963.4494 | **1242.6801** | **621.8437** |
| N[Dea] | 2040.9185 | 1020.9629 | **1145.6273** | **573.3173** |
| G | 2097.9400 | 1049.4736 | **1030.6004** | **515.8038** |
| Q | 2225.9986 | 1113.5029 | 973.5789 | 487.2931 |
| T | 2327.0462 | 1164.0268 | **845.5203** | 423.2638 |
| I | 2440.1303 | 1220.5688 | **744.4726** | 372.7400 |
| T | 2541.1780 | 1271.0926 | **631.3886** | **316.1979** |
| I | 2654.2621 | 1327.6347 | **530.3409** | 265.6741 |
| N | 2768.3050 | 1384.6561 | **417.2568** | 209.1321 |
| R | 2924.4061 | **1462.7067** | **303.2139** | 152.1106 |
| K | 3052.5011 | 1526.7542 | **147.1128** | 74.0600 |

## Protein: B.rapa.Ro18.Candidate-3, 2 peptides (95%)

*Protein sequence coverage*

MTKFTVLPLFVLLFLVLLCTKSWAKSEEFDESSDEENDVAAVPSCCGFSSPLLIKKDQWKPIFGTQFGQISTVQIGEGCGGMGPYKIHSITLEPNALLLPLLLHSDMVFFVESGSGILNWVEAEPTSSEIRRGDVYRLRPGTVFYLQSKPIDIFLGTKLRVYAIFSNTEECLHDPCFGAYSSITDLLFGFDEAILQSAFGVPEEIIGLMTNRTQPPLIVHDMLSTPGEANTYTWQLQVQPRLLKLFAGYVSAAEKKKKEKKTKK**AKTFNVFESEPDFQSPSGR**TITINRKDLEVLSGSMVGVSMVNLTQASMMGPHWNPWACEISIVLKGSGMVRVLRSSISSTSSSSSSSECKNMR**FKVEEGDIFAVPR**LHPMAQMSFINESLVFIGFTTSARNNEPQFLAGQRSALRLLDQEVLAASLNVSSVMIEGLLGAQKDAVVLGCPYCAEGELEKLKVETEMKKRDDERKREEEEAKKEEEERRKREEEEEEEKQWPPLPQQPPE

*Peptide sequence 1:* AKTFNVFESEPDFQSPSGR

| Residue | b | b+2 | y | y+2 |
| --- | --- | --- | --- | --- |
| A | 72.0444 | 36.5258 | 2143.0091 | 1072.0082 |
| K | 200.1394 | 100.5733 | 2071.9720 | 1036.4896 |
| T | 301.1870 | 151.0972 | 1943.8770 | 972.4421 |
| F | 448.2554 | 224.6314 | 1842.8293 | 921.9183 |
| N | **562.2984** | 281.6528 | 1695.7609 | 848.3841 |
| V | **661.3668** | 331.1870 | 1581.7180 | 791.3626 |
| F | **808.4352** | 404.7212 | 1482.6496 | 741.8284 |
| E | **937.4778** | 469.2425 | **1335.5811** | 668.2942 |
| S | **1024.5098** | 512.7585 | **1206.5386** | 603.7729 |
| E | **1153.5524** | 577.2798 | 1119.5065 | 560.2569 |
| P | 1250.6052 | 625.8062 | **990.4639** | **495.7356** |
| D | 1365.6321 | 683.3197 | **893.4112** | 447.2092 |
| F | 1512.7005 | 756.8539 | **778.3842** | 389.6958 |
| Q | 1640.7591 | 820.8832 | **631.3158** | 316.1615 |
| S | 1727.7911 | **864.3992** | **503.2572** | 252.1323 |
| P | 1824.8439 | 912.9256 | **416.2252** | 208.6162 |
| S | 1911.8759 | 956.4416 | 319.1724 | 160.0899 |
| G | 1968.8974 | 984.9523 | 232.1404 | 116.5738 |
| R | 2124.9985 | 1063.0029 | **175.1190** | 88.0631 |

*Peptide sequence 2:* FKVEEGDIFAVPR

| Residue | b | b+2 | y | y+2 |
| --- | --- | --- | --- | --- |
| F | 148.0757 | 74.5415 | 1506.7951 | 753.9012 |
| K | **276.1707** | 138.5890 | **1359.7267** | 680.3670 |
| V | **375.2391** | 188.1232 | 1231.6317 | **616.3195** |
| E | 504.2817 | 252.6445 | 1132.5633 | 566.7853 |
| E | **633.3243** | 317.1658 | 1003.5207 | 502.2640 |
| G | **690.3457** | 345.6765 | **874.4781** | 437.7427 |
| D | **805.3727** | **403.1900** | 817.4567 | 409.2320 |
| I | **918.4567** | **459.7320** | **702.4297** | **351.7185** |
| F | **1065.5251** | **533.2662** | **589.3457** | **295.1765** |
| A | **1136.5623** | **568.7848** | **442.2772** | 221.6423 |
| V | 1235.6307 | **618.3190** | **371.2401** | **186.1237** |
| P | 1332.6834 | 666.8454 | **272.1717** | 136.5895 |
| R | 1488.7845 | 744.8959 | **175.1190** | 88.0631 |

## Protein: B.rapa.Ro18.Candidate-4, 1 peptides (95%)

*Protein sequence coverage*

MSKFTIIPLCLLTLFLCTNSFSDQNDGVPSSQSPLLVKRHQRTQLVATEFGEISAVHIGEEYTIQFITLEPNALLLPLLLHSDMVFFVHTGSGVLNWVDEEKERTLELKRGDVFRLRYGTVFYLHCNLERDEVPEKLRVYAIFDVGKCLSDQCLGAYSSIRDLLWGFDEKTLRSAFAVPKDVFGRLRDAVKPPLITHAMPKNRTQGSEEETWGSRLAKLFVRVEDSIVVDEKDMDALKGSSFGVYMVNLTKGSMMGPHWNPNACEISIVLQGEGMIRVVNHPSYQSKNESERFMVEDGDVFVVPQFYPMAQLSFVNSSFMFMGFSTSAK**TNHPQFLVGQNSVLK**IFNRDVLATSFNMRYATVERLLGAQKDGLLLECVSCAEVELSRLMREIEERRRREEEEIERRKREEEEAKRQEEERRRREEEEAERKKKAEEEARKREKEREREEEAAKRREEERRRREEEEAERKRKEEEEARKREEERKREEEAAKKREEERRKREKEEEEARKREEAREREEEEAKKREEERRKREEEEAERKRRAEEEAREREEEEAKKREEEKEAARRREEEREKEEEMAKRREEERQRKEREDVERKKREEEEERKRREEEAMRREEERKREEEAAKRAEEERRKREEEAEHKKRPPPQGPQPPIHH

*Peptide sequence 1:* TNHPQFLVGQNSVLK

| Residue | b | b+2 | y | y+2 |
| --- | --- | --- | --- | --- |
| T | **102.0550** | 51.5311 | 1681.9020 | 841.4547 |
| N | 216.0979 | 108.5526 | 1580.8544 | 790.9308 |
| H | **353.1568** | 177.0820 | 1466.8114 | 733.9094 |
| P | 450.2096 | 225.6084 | 1329.7525 | 665.3799 |
| Q | **578.2681** | 289.6377 | 1232.6997 | 616.8535 |
| F | **725.3365** | **363.1719** | 1104.6412 | 552.8242 |
| L | **838.4206** | **419.7139** | **957.5728** | 479.2900 |
| V | **937.4890** | 469.2482 | **844.4887** | **422.7480** |
| G | **994.5105** | 497.7589 | **745.4203** | **373.2138** |
| Q | 1122.5691 | **561.7882** | **688.3988** | 344.7030 |
| N | 1236.6120 | 618.8096 | **560.3402** | 280.6738 |
| S | 1323.6440 | 662.3257 | **446.2973** | 223.6523 |
| V | 1422.7124 | 711.8599 | 359.2653 | 180.1363 |
| L | 1535.7965 | 768.4019 | **260.1969** | 130.6021 |
| K | 1663.8915 | 832.4494 | **147.1128** | 74.0600 |

## Protein: B.rapa.Ro18.Candidate-5, 20 peptides (95%)

*Protein sequence coverage*

MAINKLTITLFLLISLAVFHCLAFRVEVQEFEPPRQEGQEGPGGGSGEGWDEEATKNPYHFGQWSFKNFFQSKDGFVKMLPKFTKR**SSTLFR**GIENYRFLFQEMQPNTFLVPHHLDADYVFLVVQGK**GVIGFVTDTANESFQITKGDVVR**VPSSVTHFFANTNGTVPLRLAK**IAVPANVPGHFQVFFPAHSGFHQSYFNGFSKDVLTASFNIPEELLGRLIRGPQQEVGQGIIRR**VSPEQIKELTEHEHATSPSNKHKDKKDKHKDKDR**STFGSPFNLLTQDAIYSNNFGRYHEAHPK**RFSQLQDLDIAVGWVNMTQGSLFLPQYNSETTFVTFVENGCARYEMASPYTFQGEQQQPWFGPGQEEEVEEEMSGQVHKIVSRVCKGEVFILPAGHPFAILSQDENFVAVGFGIHASNSTR**TFLAGQDNMLSNINTVATR**LSFGLGSKMAEKLFTSQNYSHFAPTTPSHQFPEKPKPSFQSVFNLVGF

*Peptide sequence 1:* DVLTASFNIPEELLGR

| Residue | b | b+2 | y | y+2 |
| --- | --- | --- | --- | --- |
| D | 116.0342 | 58.5207 | 1773.9381 | **887.4727** |
| V | **215.1026** | 108.0550 | 1658.9112 | 829.9592 |
| L | **328.1867** | 164.5970 | 1559.8428 | **780.4250** |
| T | **429.2344** | **215.1208** | **1446.7587** | **723.8830** |
| A | **500.2715** | 250.6394 | **1345.7110** | **673.3592** |
| S | **587.3035** | 294.1554 | **1274.6739** | 637.8406 |
| F | **734.3719** | 367.6896 | **1187.6419** | 594.3246 |
| N | **848.4149** | 424.7111 | **1040.5735** | 520.7904 |
| I | **961.4989** | 481.2531 | **926.5306** | 463.7689 |
| P | **1058.5517** | 529.7795 | **813.4465** | 407.2269 |
| E | 1187.5943 | 594.3008 | **716.3937** | 358.7005 |
| E | 1316.6369 | 658.8221 | **587.3511** | 294.1792 |
| L | **1429.7209** | **715.3641** | **458.3085** | 229.6579 |
| L | 1542.8050 | **771.9061** | **345.2245** | **173.1159** |
| G | 1599.8265 | 800.4169 | **232.1404** | 116.5738 |
| R | 1755.9276 | **878.4674** | **175.1190** | 88.0631 |

*Peptide sequence 2:* GPQQEVGQGIIR

| Residue | b | b+2 | y | y+2 |
| --- | --- | --- | --- | --- |
| G | 58.0287 | 29.5180 | 1281.6910 | **641.3491** |
| P | **155.0815** | 78.0444 | 1224.6695 | **612.8384** |
| Q | **283.1401** | 142.0737 | **1127.6167** | **564.3120** |
| Q | **411.1987** | 206.1030 | **999.5582** | **500.2827** |
| E | **540.2413** | 270.6243 | **871.4996** | 436.2534 |
| V | **639.3097** | 320.1585 | **742.4570** | 371.7321 |
| G | **696.3311** | 348.6692 | **643.3886** | 322.1979 |
| Q | **824.3897** | 412.6985 | **586.3671** | 293.6872 |
| G | **881.4112** | 441.2092 | **458.3085** | 229.6579 |
| I | **994.4952** | 497.7513 | **401.2871** | **201.1472** |
| I | **1107.5793** | 554.2933 | **288.2030** | 144.6051 |
| R | 1263.6804 | **632.3438** | **175.1190** | 88.0631 |

*Peptide sequence 3:* GPQQEVGQGIIRR

| Residue | b | b+2 | y | y+2 |
| --- | --- | --- | --- | --- |
| G | 58.0287 | 29.5180 | 1437.7921 | 719.3997 |
| P | **155.0815** | 78.0444 | 1380.7706 | 690.8889 |
| Q | **283.1401** | 142.0737 | 1283.7179 | **642.3626** |
| Q | **411.1987** | 206.1030 | 1155.6593 | **578.3333** |
| E | **540.2413** | 270.6243 | **1027.6007** | **514.3040** |
| V | **639.3097** | 320.1585 | **898.5581** | **449.7827** |
| G | 696.3311 | 348.6692 | **799.4897** | **400.2485** |
| Q | 824.3897 | 412.6985 | **742.4682** | **371.7378** |
| G | **881.4112** | 441.2092 | **614.4097** | 307.7085 |
| I | 994.4952 | 497.7513 | 557.3882 | 279.1977 |
| I | 1107.5793 | 554.2933 | **444.3041** | 222.6557 |
| R | 1263.6804 | 632.3438 | 331.2201 | 166.1137 |
| R | 1419.7815 | 710.3944 | **175.1190** | 88.0631 |

*Peptide sequence 4:* GVIGFVTDTANESFQITK

| Residue | b | b+2 | y | y+2 |
| --- | --- | --- | --- | --- |
| G | 58.0287 | 29.5180 | 1926.9807 | 963.9940 |
| V | **157.0972** | 79.0522 | 1869.9593 | 935.4833 |
| I | **270.1812** | 135.5942 | 1770.8909 | **885.9491** |
| G | **327.2027** | 164.1050 | 1657.8068 | **829.4070** |
| F | **474.2711** | 237.6392 | 1600.7853 | **800.8963** |
| V | **573.3395** | 287.1734 | 1453.7169 | **727.3621** |
| T | **674.3872** | 337.6972 | **1354.6485** | **677.8279** |
| D | **789.4141** | 395.2107 | **1253.6008** | 627.3040 |
| T | **890.4618** | **445.7345** | **1138.5739** | **569.7906** |
| A | **961.4989** | 481.2531 | **1037.5262** | **519.2667** |
| N | **1075.5419** | 538.2746 | **966.4891** | **483.7482** |
| E | **1204.5844** | **602.7959** | **852.4462** | 426.7267 |
| S | 1291.6165 | **646.3119** | **723.4036** | **362.2054** |
| F | 1438.6849 | 719.8461 | **636.3715** | 318.6894 |
| Q | 1566.7435 | 783.8754 | **489.3031** | 245.1552 |
| I | 1679.8275 | 840.4174 | **361.2445** | 181.1259 |
| T | 1780.8752 | 890.9412 | **248.1605** | 124.5839 |
| K | 1908.9702 | 954.9887 | **147.1128** | 74.0600 |

*Peptide sequence 5:* GVIGFVTDTANESFQITKGDVVR

| Residue | b | b+2 | y | y+2 |
| --- | --- | --- | --- | --- |
| G | 58.0287 | 29.5180 | 2453.2671 | 1227.1372 |
| V | **157.0972** | 79.0522 | 2396.2456 | 1198.6264 |
| I | **270.1812** | 135.5942 | 2297.1772 | **1149.0922** |
| G | **327.2027** | 164.1050 | 2184.0931 | **1092.5502** |
| F | **474.2711** | 237.6392 | 2127.0717 | **1064.0395** |
| V | **573.3395** | 287.1734 | 1980.0033 | **990.5053** |
| T | **674.3872** | 337.6972 | 1880.9348 | **940.9711** |
| D | **789.4141** | 395.2107 | 1779.8872 | **890.4472** |
| T | **890.4618** | 445.7345 | 1664.8602 | **832.9338** |
| A | **961.4989** | 481.2531 | 1563.8125 | **782.4099** |
| N | 1075.5419 | 538.2746 | **1492.7754** | **746.8914** |
| E | 1204.5844 | 602.7959 | **1378.7325** | **689.8699** |
| S | 1291.6165 | 646.3119 | 1249.6899 | **625.3486** |
| F | 1438.6849 | 719.8461 | **1162.6579** | 581.8326 |
| Q | 1566.7435 | 783.8754 | **1015.5895** | 508.2984 |
| I | 1679.8275 | 840.4174 | **887.5309** | 444.2691 |
| T | 1780.8752 | 890.9412 | **774.4468** | 387.7271 |
| K | 1908.9702 | 954.9887 | **673.3991** | 337.2032 |
| G | 1965.9916 | 983.4995 | **545.3042** | **273.1557** |
| D | 2081.0186 | 1041.0129 | **488.2827** | 244.6450 |
| V | 2180.0870 | 1090.5471 | **373.2558** | 187.1315 |
| V | 2279.1554 | **1140.0813** | 274.1874 | 137.5973 |
| R | 2435.2565 | 1218.1319 | **175.1190** | 88.0631 |

*Peptide sequence 6:* IAVPAN[Dea]VPGHFQVFFPAHSGFHQSYFNGFSK

| Residue | b | b+2 | y | y+2 |
| --- | --- | --- | --- | --- |
| I | 114.0913 | 57.5493 | 3435.6698 | 1718.3385 |
| A | **185.1285** | 93.0679 | 3322.5857 | 1661.7965 |
| V | **284.1969** | 142.6021 | 3251.5486 | 1626.2779 |
| P | 381.2496 | 191.1285 | 3152.4802 | 1576.7437 |
| A | 452.2867 | 226.6470 | 3055.4274 | 1528.2174 |
| N[Dea] | 567.3137 | **284.1605** | 2984.3903 | **1492.6988** |
| V | 666.3821 | 333.6947 | 2869.3634 | 1435.1853 |
| P | 763.4349 | **382.2211** | 2770.2950 | **1385.6511** |
| G | 820.4563 | 410.7318 | 2673.2422 | 1337.1247 |
| H | 957.5152 | **479.2613** | 2616.2207 | 1308.6140 |
| F | 1104.5837 | 552.7955 | 2479.1618 | **1240.0846** |
| Q | 1232.6422 | 616.8248 | 2332.0934 | 1166.5503 |
| V | 1331.7106 | **666.3590** | 2204.0348 | **1102.5211** |
| F | 1478.7791 | 739.8932 | 2104.9664 | **1052.9868** |
| F | 1625.8475 | 813.4274 | 1957.8980 | **979.4526** |
| P | 1722.9002 | 861.9538 | 1810.8296 | **905.9184** |
| A | 1793.9374 | 897.4723 | 1713.7768 | **857.3921** |
| H | 1930.9963 | 966.0018 | 1642.7397 | **821.8735** |
| S | 2018.0283 | 1009.5178 | 1505.6808 | **753.3440** |
| G | 2075.0498 | 1038.0285 | 1418.6488 | 709.8280 |
| F | 2222.1182 | 1111.5627 | 1361.6273 | 681.3173 |
| H | 2359.1771 | 1180.0922 | **1214.5589** | **607.7831** |
| Q | 2487.2357 | 1244.1215 | **1077.5000** | 539.2536 |
| S | 2574.2677 | 1287.6375 | **949.4414** | **475.2243** |
| Y | 2737.3310 | 1369.1691 | **862.4094** | 431.7083 |
| F | 2884.3994 | 1442.7034 | **699.3461** | 350.1767 |
| N | 2998.4424 | 1499.7248 | **552.2776** | 276.6425 |
| G | 3055.4638 | 1528.2355 | **438.2347** | 219.6210 |
| F | 3202.5322 | 1601.7698 | **381.2132** | 191.1103 |
| S | 3289.5643 | 1645.2858 | **234.1448** | 117.5761 |
| K | 3417.6592 | 1709.3333 | 147.1128 | 74.0600 |

*Peptide sequence 7:* IAVPANVPGHFQVFFPAHSGFHQSYFNGFSK

| Residue | b | b+2 | y | y+2 |
| --- | --- | --- | --- | --- |
| I | 114.0913 | 57.5493 | 3434.6858 | 1717.8465 |
| A | **185.1285** | 93.0679 | 3321.6017 | 1661.3045 |
| V | **284.1969** | 142.6021 | 3250.5646 | 1625.7859 |
| P | **381.2496** | 191.1285 | 3151.4962 | 1576.2517 |
| A | 452.2867 | 226.6470 | 3054.4434 | 1527.7253 |
| N | 566.3297 | 283.6685 | 2983.4063 | 1492.2068 |
| V | 665.3981 | 333.2027 | 2869.3634 | 1435.1853 |
| P | 762.4509 | 381.7291 | 2770.2950 | **1385.6511** |
| G | 819.4723 | 410.2398 | 2673.2422 | **1337.1247** |
| H | 956.5312 | 478.7693 | 2616.2207 | 1308.6140 |
| F | 1103.5996 | 552.3035 | 2479.1618 | **1240.0846** |
| Q | 1231.6582 | 616.3327 | 2332.0934 | **1166.5503** |
| V | 1330.7266 | 665.8670 | 2204.0348 | **1102.5211** |
| F | 1477.7950 | **739.4012** | 2104.9664 | **1052.9868** |
| F | 1624.8635 | 812.9354 | 1957.8980 | **979.4526** |
| P | 1721.9162 | 861.4618 | 1810.8296 | **905.9184** |
| A | 1792.9533 | 896.9803 | 1713.7768 | **857.3921** |
| H | 1930.0122 | 965.5098 | 1642.7397 | **821.8735** |
| S | 2017.0443 | 1009.0258 | 1505.6808 | **753.3440** |
| G | 2074.0657 | 1037.5365 | **1418.6488** | **709.8280** |
| F | 2221.1342 | 1111.0707 | **1361.6273** | 681.3173 |
| H | 2358.1931 | 1179.6002 | **1214.5589** | **607.7831** |
| Q | 2486.2516 | 1243.6295 | **1077.5000** | **539.2536** |
| S | 2573.2837 | 1287.1455 | **949.4414** | **475.2243** |
| Y | 2736.3470 | 1368.6771 | **862.4094** | **431.7083** |
| F | 2883.4154 | 1442.2113 | **699.3461** | 350.1767 |
| N | 2997.4583 | 1499.2328 | **552.2776** | 276.6425 |
| G | 3054.4798 | 1527.7435 | **438.2347** | 219.6210 |
| F | 3201.5482 | 1601.2777 | **381.2132** | 191.1103 |
| S | 3288.5802 | 1644.7938 | **234.1448** | 117.5761 |
| K | 3416.6752 | 1708.8412 | **147.1128** | 74.0600 |

*Peptide sequence 8:* IAVPANVPGHFQ[Dea]VFFPAHSGFHQSYFNGFSK

| Residue | b | b+2 | y | y+2 |
| --- | --- | --- | --- | --- |
| I | 114.0913 | 57.5493 | 3435.6698 | 1718.3385 |
| A | **185.1285** | 93.0679 | 3322.5857 | 1661.7965 |
| V | **284.1969** | 142.6021 | 3251.5486 | 1626.2779 |
| P | 381.2496 | 191.1285 | 3152.4802 | 1576.7437 |
| A | 452.2867 | 226.6470 | 3055.4274 | 1528.2174 |
| N | 566.3297 | 283.6685 | 2984.3903 | **1492.6988** |
| V | 665.3981 | 333.2027 | 2870.3474 | **1435.6773** |
| P | 762.4509 | 381.7291 | 2771.2790 | 1386.1431 |
| G | 819.4723 | 410.2398 | 2674.2262 | **1337.6167** |
| H | 956.5312 | 478.7693 | 2617.2048 | 1309.1060 |
| F | 1103.5996 | **552.3035** | 2480.1458 | **1240.5766** |
| Q[Dea] | 1232.6422 | 616.8248 | 2333.0774 | **1167.0424** |
| V | 1331.7106 | **666.3590** | 2204.0348 | **1102.5211** |
| F | 1478.7791 | 739.8932 | 2104.9664 | **1052.9868** |
| F | 1625.8475 | 813.4274 | 1957.8980 | **979.4526** |
| P | 1722.9002 | 861.9538 | 1810.8296 | **905.9184** |
| A | 1793.9374 | 897.4723 | 1713.7768 | **857.3921** |
| H | 1930.9963 | 966.0018 | 1642.7397 | **821.8735** |
| S | 2018.0283 | 1009.5178 | 1505.6808 | **753.3440** |
| G | 2075.0498 | 1038.0285 | 1418.6488 | 709.8280 |
| F | 2222.1182 | 1111.5627 | 1361.6273 | 681.3173 |
| H | 2359.1771 | 1180.0922 | **1214.5589** | **607.7831** |
| Q | 2487.2357 | 1244.1215 | **1077.5000** | 539.2536 |
| S | 2574.2677 | 1287.6375 | **949.4414** | **475.2243** |
| Y | 2737.3310 | 1369.1691 | **862.4094** | 431.7083 |
| F | 2884.3994 | 1442.7034 | **699.3461** | 350.1767 |
| N | 2998.4424 | 1499.7248 | **552.2776** | 276.6425 |
| G | 3055.4638 | 1528.2355 | **438.2347** | 219.6210 |
| F | 3202.5322 | 1601.7698 | **381.2132** | 191.1103 |
| S | 3289.5643 | 1645.2858 | **234.1448** | 117.5761 |
| K | 3417.6592 | 1709.3333 | 147.1128 | 74.0600 |

*Peptide sequence 9:* IAVPANVPGHFQVFFPAHSGFHQSYFN[Dea]GFSK

| Residue | b | b+2 | y | y+2 |
| --- | --- | --- | --- | --- |
| I | 114.0913 | 57.5493 | 3435.6698 | 1718.3385 |
| A | **185.1285** | 93.0679 | 3322.5857 | 1661.7965 |
| V | **284.1969** | 142.6021 | 3251.5486 | 1626.2779 |
| P | 381.2496 | 191.1285 | 3152.4802 | 1576.7437 |
| A | 452.2867 | 226.6470 | 3055.4274 | 1528.2174 |
| N | 566.3297 | 283.6685 | 2984.3903 | 1492.6988 |
| V | 665.3981 | 333.2027 | 2870.3474 | 1435.6773 |
| P | 762.4509 | 381.7291 | 2771.2790 | **1386.1431** |
| G | 819.4723 | 410.2398 | 2674.2262 | 1337.6167 |
| H | 956.5312 | 478.7693 | 2617.2048 | 1309.1060 |
| F | 1103.5996 | 552.3035 | 2480.1458 | 1240.5766 |
| Q | 1231.6582 | 616.3327 | 2333.0774 | 1167.0424 |
| V | 1330.7266 | 665.8670 | 2205.0189 | **1103.0131** |
| F | 1477.7950 | 739.4012 | 2105.9504 | **1053.4789** |
| F | 1624.8635 | 812.9354 | 1958.8820 | 979.9446 |
| P | 1721.9162 | 861.4618 | 1811.8136 | **906.4104** |
| A | 1792.9533 | 896.9803 | 1714.7608 | 857.8841 |
| H | 1930.0122 | 965.5098 | 1643.7237 | 822.3655 |
| S | 2017.0443 | 1009.0258 | 1506.6648 | 753.8360 |
| G | 2074.0657 | 1037.5365 | 1419.6328 | 710.3200 |
| F | 2221.1342 | 1111.0707 | 1362.6113 | 681.8093 |
| H | 2358.1931 | 1179.6002 | 1215.5429 | 608.2751 |
| Q | 2486.2516 | 1243.6295 | 1078.4840 | 539.7456 |
| S | 2573.2837 | 1287.1455 | 950.4254 | 475.7164 |
| Y | 2736.3470 | 1368.6771 | **863.3934** | 432.2003 |
| F | 2883.4154 | 1442.2113 | **700.3301** | 350.6687 |
| N[Dea] | 2998.4424 | 1499.7248 | **553.2617** | 277.1345 |
| G | 3055.4638 | 1528.2355 | **438.2347** | 219.6210 |
| F | 3202.5322 | 1601.7698 | 381.2132 | 191.1103 |
| S | 3289.5643 | 1645.2858 | **234.1448** | 117.5761 |
| K | 3417.6592 | 1709.3333 | **147.1128** | 74.0600 |

*Peptide sequence 10:* IAVPANVPGHFQVFFPAHSGFHQ[Dea]SYFNGFSK

| Residue | b | b+2 | y | y+2 |
| --- | --- | --- | --- | --- |
| I | 114.0913 | 57.5493 | 3435.6698 | 1718.3385 |
| A | **185.1285** | 93.0679 | 3322.5857 | 1661.7965 |
| V | **284.1969** | 142.6021 | 3251.5486 | 1626.2779 |
| P | 381.2496 | 191.1285 | 3152.4802 | 1576.7437 |
| A | 452.2867 | 226.6470 | 3055.4274 | 1528.2174 |
| N | 566.3297 | 283.6685 | 2984.3903 | 1492.6988 |
| V | 665.3981 | 333.2027 | 2870.3474 | 1435.6773 |
| P | 762.4509 | 381.7291 | 2771.2790 | 1386.1431 |
| G | 819.4723 | 410.2398 | 2674.2262 | 1337.6167 |
| H | 956.5312 | 478.7693 | 2617.2048 | 1309.1060 |
| F | 1103.5996 | **552.3035** | 2480.1458 | **1240.5766** |
| Q | 1231.6582 | 616.3327 | 2333.0774 | **1167.0424** |
| V | 1330.7266 | 665.8670 | 2205.0189 | **1103.0131** |
| F | 1477.7950 | **739.4012** | 2105.9504 | **1053.4789** |
| F | 1624.8635 | 812.9354 | 1958.8820 | **979.9446** |
| P | 1721.9162 | 861.4618 | 1811.8136 | **906.4104** |
| A | 1792.9533 | 896.9803 | 1714.7608 | 857.8841 |
| H | 1930.0122 | 965.5098 | 1643.7237 | **822.3655** |
| S | 2017.0443 | 1009.0258 | 1506.6648 | **753.8360** |
| G | 2074.0657 | 1037.5365 | **1419.6328** | 710.3200 |
| F | 2221.1342 | 1111.0707 | **1362.6113** | 681.8093 |
| H | 2358.1931 | 1179.6002 | 1215.5429 | **608.2751** |
| Q[Dea] | 2487.2357 | 1244.1215 | 1078.4840 | 539.7456 |
| S | 2574.2677 | 1287.6375 | **949.4414** | **475.2243** |
| Y | 2737.3310 | 1369.1691 | **862.4094** | 431.7083 |
| F | 2884.3994 | 1442.7034 | **699.3461** | 350.1767 |
| N | 2998.4424 | 1499.7248 | **552.2776** | 276.6425 |
| G | 3055.4638 | 1528.2355 | **438.2347** | 219.6210 |
| F | 3202.5322 | 1601.7698 | **381.2132** | 191.1103 |
| S | 3289.5643 | 1645.2858 | **234.1448** | 117.5761 |
| K | 3417.6592 | 1709.3333 | **147.1128** | 74.0600 |

*Peptide sequence 11:* LIRGPQQEVGQGIIR

| Residue | b | b+2 | y | y+2 |
| --- | --- | --- | --- | --- |
| L | 114.0913 | 57.5493 | 1663.9602 | 832.4837 |
| I | **227.1754** | 114.0913 | 1550.8761 | **775.9417** |
| R | **383.2765** | 192.1419 | 1437.7921 | **719.3997** |
| G | **440.2980** | 220.6526 | **1281.6910** | **641.3491** |
| P | **537.3507** | **269.1790** | 1224.6695 | **612.8384** |
| Q | **665.4093** | **333.2083** | **1127.6167** | 564.3120 |
| Q | **793.4679** | **397.2376** | **999.5582** | **500.2827** |
| E | **922.5105** | **461.7589** | **871.4996** | **436.2534** |
| V | **1021.5789** | **511.2931** | **742.4570** | **371.7321** |
| G | **1078.6004** | **539.8038** | **643.3886** | **322.1979** |
| Q | **1206.6589** | **603.8331** | **586.3671** | **293.6872** |
| G | **1263.6804** | **632.3438** | **458.3085** | 229.6579 |
| I | **1376.7645** | **688.8859** | **401.2871** | **201.1472** |
| I | 1489.8485 | **745.4279** | **288.2030** | 144.6051 |
| R | 1645.9496 | 823.4785 | **175.1190** | 88.0631 |

*Peptide sequence 12:* LIRGPQQ[Dea]EVGQGIIR

| Residue | b | b+2 | y | y+2 |
| --- | --- | --- | --- | --- |
| L | 114.0913 | 57.5493 | 1664.9442 | 832.9758 |
| I | **227.1754** | 114.0913 | 1551.8602 | **776.4337** |
| R | **383.2765** | 192.1419 | 1438.7761 | **719.8917** |
| G | **440.2980** | 220.6526 | 1282.6750 | 641.8411 |
| P | 537.3507 | 269.1790 | 1225.6535 | **613.3304** |
| Q | **665.4093** | **333.2083** | **1128.6008** | 564.8040 |
| Q[Dea] | **794.4519** | **397.7296** | **1000.5422** | 500.7747 |
| E | **923.4945** | **462.2509** | **871.4996** | 436.2534 |
| V | **1022.5629** | **511.7851** | **742.4570** | 371.7321 |
| G | **1079.5844** | **540.2958** | **643.3886** | **322.1979** |
| Q | 1207.6430 | 604.3251 | **586.3671** | 293.6872 |
| G | **1264.6644** | 632.8359 | **458.3085** | 229.6579 |
| I | 1377.7485 | 689.3779 | **401.2871** | **201.1472** |
| I | 1490.8326 | 745.9199 | **288.2030** | 144.6051 |
| R | 1646.9337 | 823.9705 | **175.1190** | 88.0631 |

*Peptide sequence 13:* LIRGPQ[Dea]QEVGQGIIR

| Residue | b | b+2 | y | y+2 |
| --- | --- | --- | --- | --- |
| L | 114.0913 | 57.5493 | 1664.9442 | 832.9758 |
| I | **227.1754** | 114.0913 | 1551.8602 | **776.4337** |
| R | **383.2765** | 192.1419 | 1438.7761 | **719.8917** |
| G | **440.2980** | 220.6526 | 1282.6750 | 641.8411 |
| P | 537.3507 | 269.1790 | 1225.6535 | 613.3304 |
| Q[Dea] | **666.3933** | **333.7003** | **1128.6008** | 564.8040 |
| Q | **794.4519** | **397.7296** | **999.5582** | 500.2827 |
| E | **923.4945** | **462.2509** | **871.4996** | 436.2534 |
| V | **1022.5629** | 511.7851 | **742.4570** | **371.7321** |
| G | **1079.5844** | 540.2958 | **643.3886** | 322.1979 |
| Q | 1207.6430 | 604.3251 | **586.3671** | 293.6872 |
| G | 1264.6644 | 632.8359 | **458.3085** | 229.6579 |
| I | 1377.7485 | 689.3779 | **401.2871** | **201.1472** |
| I | 1490.8326 | 745.9199 | **288.2030** | 144.6051 |
| R | 1646.9337 | 823.9705 | **175.1190** | 88.0631 |

*Peptide sequence 14:* LIRGPQQEVGQGIIRR

| Residue | b | b+2 | y | y+2 |
| --- | --- | --- | --- | --- |
| L | 114.0913 | 57.5493 | 1820.0613 | 910.5343 |
| I | **227.1754** | 114.0913 | 1706.9773 | 853.9923 |
| R | **383.2765** | 192.1419 | 1593.8932 | 797.4502 |
| G | **440.2980** | 220.6526 | 1437.7921 | **719.3997** |
| P | **537.3507** | **269.1790** | 1380.7706 | 690.8889 |
| Q | **665.4093** | **333.2083** | 1283.7179 | **642.3626** |
| Q | **793.4679** | **397.2376** | 1155.6593 | **578.3333** |
| E | **922.5105** | **461.7589** | 1027.6007 | **514.3040** |
| V | 1021.5789 | **511.2931** | 898.5581 | **449.7827** |
| G | 1078.6004 | 539.8038 | **799.4897** | **400.2485** |
| Q | 1206.6589 | 603.8331 | **742.4682** | **371.7378** |
| G | 1263.6804 | 632.3438 | **614.4097** | **307.7085** |
| I | 1376.7645 | 688.8859 | 557.3882 | 279.1977 |
| I | 1489.8485 | 745.4279 | **444.3041** | 222.6557 |
| R | 1645.9496 | 823.4785 | 331.2201 | 166.1137 |
| R | 1802.0508 | 901.5290 | **175.1190** | 88.0631 |

*Peptide sequence 15:* STFGSPFNLLTQDAIYSNNFGR

| Residue | b | b+2 | y | y+2 |
| --- | --- | --- | --- | --- |
| S | 88.0393 | 44.5233 | 2449.1783 | 1225.0928 |
| T | **189.0870** | 95.0471 | 2362.1462 | 1181.5768 |
| F | **336.1554** | 168.5813 | 2261.0986 | **1131.0529** |
| G | **393.1769** | 197.0921 | 2114.0301 | **1057.5187** |
| S | **480.2089** | 240.6081 | 2057.0087 | **1029.0080** |
| P | **577.2617** | 289.1345 | 1969.9767 | **985.4920** |
| F | **724.3301** | 362.6687 | 1872.9239 | 936.9656 |
| N | **838.3730** | **419.6901** | 1725.8555 | **863.4314** |
| L | **951.4571** | **476.2322** | 1611.8125 | **806.4099** |
| L | **1064.5411** | **532.7742** | **1498.7285** | **749.8679** |
| T | **1165.5888** | **583.2980** | **1385.6444** | **693.3258** |
| Q | **1293.6474** | **647.3273** | **1284.5967** | **642.8020** |
| D | **1408.6743** | **704.8408** | **1156.5382** | 578.7727 |
| A | **1479.7114** | **740.3594** | **1041.5112** | **521.2592** |
| I | 1592.7955 | **796.9014** | **970.4741** | **485.7407** |
| Y | 1755.8588 | **878.4331** | **857.3900** | **429.1987** |
| S | 1842.8909 | 921.9491 | **694.3267** | 347.6670 |
| N | 1956.9338 | 978.9705 | **607.2947** | 304.1510 |
| N | 2070.9767 | **1035.9920** | **493.2518** | **247.1295** |
| F | 2218.0451 | **1109.5262** | **379.2088** | **190.1081** |
| G | 2275.0666 | 1138.0369 | **232.1404** | 116.5738 |
| R | 2431.1677 | 1216.0875 | **175.1190** | 88.0631 |

*Peptide sequence 16:* STFGSPFNLLTQDAIYSNNFGRYHEAHPK

| Residue | b | b+2 | y | y+2 |
| --- | --- | --- | --- | --- |
| S | 88.0393 | 44.5233 | 3311.5869 | 1656.2971 |
| T | 189.0870 | 95.0471 | 3224.5548 | 1612.7810 |
| F | **336.1554** | 168.5813 | 3123.5071 | 1562.2572 |
| G | **393.1769** | 197.0921 | 2976.4387 | 1488.7230 |
| S | **480.2089** | 240.6081 | 2919.4173 | 1460.2123 |
| P | **577.2617** | 289.1345 | 2832.3852 | 1416.6963 |
| F | **724.3301** | 362.6687 | 2735.3325 | 1368.1699 |
| N | **838.3730** | **419.6901** | 2588.2641 | 1294.6357 |
| L | **951.4571** | **476.2322** | 2474.2211 | 1237.6142 |
| L | **1064.5411** | 532.7742 | 2361.1371 | **1181.0722** |
| T | 1165.5888 | 583.2980 | 2248.0530 | **1124.5301** |
| Q | 1293.6474 | 647.3273 | 2147.0053 | **1074.0063** |
| D | 1408.6743 | 704.8408 | 2018.9467 | **1009.9770** |
| A | **1479.7114** | 740.3594 | 1903.9198 | **952.4635** |
| I | 1592.7955 | 796.9014 | 1832.8827 | **916.9450** |
| Y | 1755.8588 | 878.4331 | 1719.7986 | **860.4030** |
| S | 1842.8909 | 921.9491 | 1556.7353 | **778.8713** |
| N | 1956.9338 | 978.9705 | 1469.7033 | **735.3553** |
| N | 2070.9767 | 1035.9920 | 1355.6603 | **678.3338** |
| F | 2218.0451 | 1109.5262 | **1241.6174** | **621.3123** |
| G | 2275.0666 | 1138.0369 | **1094.5490** | **547.7781** |
| R | 2431.1677 | 1216.0875 | 1037.5275 | 519.2674 |
| Y | 2594.2310 | 1297.6192 | **881.4264** | 441.2169 |
| H | 2731.2899 | 1366.1486 | **718.3631** | 359.6852 |
| E | 2860.3325 | 1430.6699 | **581.3042** | 291.1557 |
| A | 2931.3696 | 1466.1885 | **452.2616** | 226.6344 |
| H | 3068.4286 | 1534.7179 | **381.2245** | 191.1159 |
| P | 3165.4813 | 1583.2443 | **244.1656** | 122.5864 |
| K | 3293.5763 | 1647.2918 | **147.1128** | 74.0600 |

*Peptide sequence 17:* STFGSPFN[Dea]LLTQDAIYSNNFGRYHEAHPK

| Residue | b | b+2 | y | y+2 |
| --- | --- | --- | --- | --- |
| S | 88.0393 | 44.5233 | 3312.5709 | 1656.7891 |
| T | **189.0870** | 95.0471 | 3225.5388 | 1613.2731 |
| F | 336.1554 | 168.5813 | 3124.4912 | 1562.7492 |
| G | 393.1769 | 197.0921 | 2977.4227 | 1489.2150 |
| S | **480.2089** | 240.6081 | 2920.4013 | 1460.7043 |
| P | **577.2617** | 289.1345 | 2833.3693 | 1417.1883 |
| F | 724.3301 | 362.6687 | 2736.3165 | **1368.6619** |
| N[Dea] | 839.3570 | 420.1821 | 2589.2481 | 1295.1277 |
| L | **952.4411** | 476.7242 | 2474.2211 | **1237.6142** |
| L | **1065.5251** | 533.2662 | 2361.1371 | **1181.0722** |
| T | 1166.5728 | 583.7900 | 2248.0530 | **1124.5301** |
| Q | **1294.6314** | 647.8193 | 2147.0053 | **1074.0063** |
| D | 1409.6583 | 705.3328 | 2018.9467 | **1009.9770** |
| A | 1480.6955 | 740.8514 | 1903.9198 | **952.4635** |
| I | 1593.7795 | 797.3934 | 1832.8827 | **916.9450** |
| Y | 1756.8428 | 878.9251 | 1719.7986 | **860.4030** |
| S | 1843.8749 | 922.4411 | 1556.7353 | **778.8713** |
| N | 1957.9178 | 979.4625 | 1469.7033 | **735.3553** |
| N | 2071.9607 | 1036.4840 | 1355.6603 | 678.3338 |
| F | 2219.0291 | 1110.0182 | **1241.6174** | 621.3123 |
| G | 2276.0506 | 1138.5289 | 1094.5490 | 547.7781 |
| R | 2432.1517 | 1216.5795 | 1037.5275 | 519.2674 |
| Y | 2595.2150 | 1298.1112 | **881.4264** | 441.2169 |
| H | 2732.2740 | 1366.6406 | **718.3631** | 359.6852 |
| E | 2861.3165 | 1431.1619 | 581.3042 | 291.1557 |
| A | 2932.3537 | 1466.6805 | **452.2616** | 226.6344 |
| H | 3069.4126 | 1535.2099 | **381.2245** | 191.1159 |
| P | 3166.4653 | 1583.7363 | **244.1656** | 122.5864 |
| K | 3294.5603 | 1647.7838 | **147.1128** | 74.0600 |

*Peptide sequence 18:* STFGSPFNLLTQ[Dea]DAIYSNNFGRYHEAHPK

| Residue | b | b+2 | y | y+2 |
| --- | --- | --- | --- | --- |
| S | 88.0393 | 44.5233 | 3312.5709 | 1656.7891 |
| T | **189.0870** | 95.0471 | 3225.5388 | 1613.2731 |
| F | 336.1554 | 168.5813 | 3124.4912 | 1562.7492 |
| G | 393.1769 | 197.0921 | 2977.4227 | 1489.2150 |
| S | **480.2089** | 240.6081 | 2920.4013 | 1460.7043 |
| P | 577.2617 | 289.1345 | 2833.3693 | 1417.1883 |
| F | **724.3301** | 362.6687 | 2736.3165 | 1368.6619 |
| N | **838.3730** | 419.6901 | 2589.2481 | **1295.1277** |
| L | 951.4571 | 476.2322 | 2475.2051 | **1238.1062** |
| L | 1064.5411 | 532.7742 | 2362.1211 | **1181.5642** |
| T | 1165.5888 | 583.2980 | 2249.0370 | **1125.0221** |
| Q[Dea] | 1294.6314 | 647.8193 | 2147.9893 | **1074.4983** |
| D | **1409.6583** | 705.3328 | 2018.9467 | **1009.9770** |
| A | 1480.6955 | 740.8514 | 1903.9198 | **952.4635** |
| I | 1593.7795 | 797.3934 | 1832.8827 | **916.9450** |
| Y | 1756.8428 | 878.9251 | 1719.7986 | **860.4030** |
| S | 1843.8749 | 922.4411 | 1556.7353 | **778.8713** |
| N | 1957.9178 | 979.4625 | 1469.7033 | 735.3553 |
| N | 2071.9607 | 1036.4840 | 1355.6603 | 678.3338 |
| F | 2219.0291 | 1110.0182 | 1241.6174 | 621.3123 |
| G | 2276.0506 | 1138.5289 | 1094.5490 | 547.7781 |
| R | 2432.1517 | 1216.5795 | 1037.5275 | 519.2674 |
| Y | 2595.2150 | 1298.1112 | 881.4264 | 441.2169 |
| H | 2732.2740 | 1366.6406 | **718.3631** | 359.6852 |
| E | 2861.3165 | 1431.1619 | **581.3042** | 291.1557 |
| A | 2932.3537 | 1466.6805 | **452.2616** | 226.6344 |
| H | 3069.4126 | 1535.2099 | **381.2245** | 191.1159 |
| P | 3166.4653 | 1583.7363 | **244.1656** | 122.5864 |
| K | 3294.5603 | 1647.7838 | **147.1128** | 74.0600 |

*Peptide sequence 19:* STFGSPFNLLTQDAIYSNN[Dea]FGRYHEAHPK

| Residue | b | b+2 | y | y+2 |
| --- | --- | --- | --- | --- |
| S | 88.0393 | 44.5233 | 3312.5709 | 1656.7891 |
| T | **189.0870** | 95.0471 | 3225.5388 | 1613.2731 |
| F | 336.1554 | 168.5813 | 3124.4912 | 1562.7492 |
| G | **393.1769** | 197.0921 | 2977.4227 | 1489.2150 |
| S | **480.2089** | 240.6081 | 2920.4013 | 1460.7043 |
| P | 577.2617 | 289.1345 | 2833.3693 | 1417.1883 |
| F | 724.3301 | 362.6687 | 2736.3165 | 1368.6619 |
| N | **838.3730** | 419.6901 | 2589.2481 | **1295.1277** |
| L | **951.4571** | 476.2322 | 2475.2051 | **1238.1062** |
| L | 1064.5411 | 532.7742 | 2362.1211 | **1181.5642** |
| T | 1165.5888 | 583.2980 | 2249.0370 | **1125.0221** |
| Q | 1293.6474 | 647.3273 | 2147.9893 | **1074.4983** |
| D | **1408.6743** | 704.8408 | 2019.9308 | **1010.4690** |
| A | **1479.7114** | 740.3594 | 1904.9038 | **952.9555** |
| I | 1592.7955 | 796.9014 | 1833.8667 | **917.4370** |
| Y | 1755.8588 | 878.4331 | 1720.7826 | **860.8950** |
| S | 1842.8909 | 921.9491 | 1557.7193 | **779.3633** |
| N | 1956.9338 | 978.9705 | 1470.6873 | **735.8473** |
| N[Dea] | 2071.9607 | 1036.4840 | 1356.6444 | 678.8258 |
| F | 2219.0291 | 1110.0182 | **1241.6174** | 621.3123 |
| G | 2276.0506 | 1138.5289 | 1094.5490 | 547.7781 |
| R | 2432.1517 | 1216.5795 | 1037.5275 | 519.2674 |
| Y | 2595.2150 | 1298.1112 | 881.4264 | 441.2169 |
| H | 2732.2740 | 1366.6406 | 718.3631 | 359.6852 |
| E | 2861.3165 | 1431.1619 | **581.3042** | 291.1557 |
| A | 2932.3537 | 1466.6805 | **452.2616** | 226.6344 |
| H | 3069.4126 | 1535.2099 | **381.2245** | 191.1159 |
| P | 3166.4653 | 1583.7363 | **244.1656** | 122.5864 |
| K | 3294.5603 | 1647.7838 | **147.1128** | 74.0600 |

*Peptide sequence 20:* STFGSPFNLLTQDAIYSN[Dea]NFGRYHEAHPK

| Residue | b | b+2 | y | y+2 |
| --- | --- | --- | --- | --- |
| S | 88.0393 | 44.5233 | 3312.5709 | 1656.7891 |
| T | **189.0870** | 95.0471 | 3225.5388 | 1613.2731 |
| F | 336.1554 | 168.5813 | 3124.4912 | 1562.7492 |
| G | **393.1769** | 197.0921 | 2977.4227 | 1489.2150 |
| S | **480.2089** | 240.6081 | 2920.4013 | 1460.7043 |
| P | 577.2617 | 289.1345 | 2833.3693 | 1417.1883 |
| F | 724.3301 | 362.6687 | 2736.3165 | 1368.6619 |
| N | **838.3730** | 419.6901 | 2589.2481 | **1295.1277** |
| L | **951.4571** | 476.2322 | 2475.2051 | **1238.1062** |
| L | 1064.5411 | 532.7742 | 2362.1211 | **1181.5642** |
| T | 1165.5888 | 583.2980 | 2249.0370 | **1125.0221** |
| Q | 1293.6474 | 647.3273 | 2147.9893 | **1074.4983** |
| D | **1408.6743** | 704.8408 | 2019.9308 | **1010.4690** |
| A | **1479.7114** | 740.3594 | 1904.9038 | **952.9555** |
| I | 1592.7955 | 796.9014 | 1833.8667 | **917.4370** |
| Y | 1755.8588 | 878.4331 | 1720.7826 | **860.8950** |
| S | 1842.8909 | 921.9491 | 1557.7193 | **779.3633** |
| N[Dea] | 1957.9178 | 979.4625 | 1470.6873 | **735.8473** |
| N | 2071.9607 | 1036.4840 | 1355.6603 | **678.3338** |
| F | 2219.0291 | 1110.0182 | **1241.6174** | 621.3123 |
| G | 2276.0506 | 1138.5289 | 1094.5490 | 547.7781 |
| R | 2432.1517 | 1216.5795 | 1037.5275 | 519.2674 |
| Y | 2595.2150 | 1298.1112 | 881.4264 | 441.2169 |
| H | 2732.2740 | 1366.6406 | 718.3631 | 359.6852 |
| E | 2861.3165 | 1431.1619 | **581.3042** | 291.1557 |
| A | 2932.3537 | 1466.6805 | **452.2616** | 226.6344 |
| H | 3069.4126 | 1535.2099 | **381.2245** | 191.1159 |
| P | 3166.4653 | 1583.7363 | **244.1656** | 122.5864 |
| K | 3294.5603 | 1647.7838 | **147.1128** | 74.0600 |

*Peptide sequence 21:* TFLAGQDNM[Oxi]LSNINTVATR

| Residue | b | b+2 | y | y+2 |
| --- | --- | --- | --- | --- |
| T | **102.0550** | 51.5311 | 2082.0284 | 1041.5179 |
| F | **249.1234** | 125.0653 | 1980.9808 | 990.9940 |
| L | **362.2074** | 181.6074 | 1833.9123 | 917.4598 |
| A | **433.2445** | 217.1259 | 1720.8283 | 860.9178 |
| G | **490.2660** | 245.6366 | 1649.7912 | 825.3992 |
| Q | **618.3246** | 309.6659 | 1592.7697 | 796.8885 |
| D | 733.3515 | 367.1794 | 1464.7111 | 732.8592 |
| N | **847.3945** | 424.2009 | 1349.6842 | 675.3457 |
| M[Oxi] | **994.4299** | **497.7186** | 1235.6412 | **618.3243** |
| L | **1107.5139** | 554.2606 | 1088.6058 | 544.8066 |
| S | 1194.5459 | 597.7766 | **975.5218** | **488.2645** |
| N | **1308.5889** | 654.7981 | **888.4898** | 444.7485 |
| I | **1421.6729** | 711.3401 | **774.4468** | 387.7271 |
| N | 1535.7159 | 768.3616 | **661.3628** | 331.1850 |
| T | 1636.7635 | 818.8854 | **547.3198** | 274.1636 |
| V | 1735.8320 | 868.4196 | **446.2722** | 223.6397 |
| A | 1806.8691 | 903.9382 | **347.2037** | 174.1055 |
| T | 1907.9168 | 954.4620 | **276.1666** | 138.5870 |
| R | 2064.0179 | 1032.5126 | **175.1190** | 88.0631 |

# Sample Pool 4

## Protein: B.rapa.Ro18.Candidate-1, 11 peptides (95%)

*Protein sequence coverage*

MEKNKRIFTFLLVIVFFHGVMMMRSIGYEGEEEQGGGGRERGGFMMKESRQVIKSEGGEMRVVISPRGR**IIEKPMHIGFLTMEPKTLFVPQYLDSNLLIFIR**QGEATLGVICKDEFGEKRLKGGDIYWIPAGSAFYLLNTGRGQRLHVICSIDPSQSLGFETFQPFYIGGGPSSVLAGFDPDTITSALNVSRPEVQQLMTSQVRGPIVHITEHAPTMWTDFLGLRGEEKHKHLKKLLELKQGTSQEQEDNPWWSWKNIVSSILDVTGEKNRGSGSSKCEDSYNIYDR**KNDFENDYGWSKALDYDDYEPLR**YSGVGVYLVNLTAGSMMAPHMNPTATEYGIVLSGSGEIQVVLPNGTSAMNMR**VSPGDVFWIPR**YFAFCQIASR**IAPFEFVGFTTSAYKNRPQFLVGSNSLLR**SLNLTSLAMAFGVDEGTMKR**FVEAQR**EAVILPTASAAPPHEGEPERFGSDHIFT

*Peptide sequence 1:* ALDYDDYEPLR

| Residue | b | b+2 | y | y+2 |
| --- | --- | --- | --- | --- |
| A | 72.0444 | 36.5258 | 1369.6270 | **685.3172** |
| L | 185.1285 | 93.0679 | **1298.5899** | **649.7986** |
| D | 300.1554 | 150.5813 | **1185.5059** | **593.2566** |
| Y | 463.2187 | **232.1130** | **1070.4789** | **535.7431** |
| D | 578.2457 | 289.6265 | **907.4156** | **454.2114** |
| D | 693.2726 | 347.1399 | **792.3886** | 396.6980 |
| Y | 856.3359 | 428.6716 | **677.3617** | 339.1845 |
| E | 985.3785 | 493.1929 | **514.2984** | 257.6528 |
| P | 1082.4313 | 541.7193 | **385.2558** | 193.1315 |
| L | 1195.5154 | 598.2613 | **288.2030** | 144.6051 |
| R | 1351.6165 | **676.3119** | **175.1190** | 88.0631 |

*Peptide sequence 2:* FVEAQR

| Residue | b | b+2 | y | y+2 |
| --- | --- | --- | --- | --- |
| F | 148.0757 | 74.5415 | 749.3941 | **375.2007** |
| V | **247.1441** | 124.0757 | **602.3257** | **301.6665** |
| E | **376.1867** | 188.5970 | **503.2572** | **252.1323** |
| A | **447.2238** | 224.1155 | **374.2146** | 187.6110 |
| Q | **575.2824** | 288.1448 | **303.1775** | 152.0924 |
| R | 731.3835 | **366.1954** | **175.1190** | 88.0631 |

*Peptide sequence 3:* IAPFEFVGFTTSAYK

| Residue | b | b+2 | y | y+2 |
| --- | --- | --- | --- | --- |
| I | 114.0913 | 57.5493 | 1677.8523 | 839.4298 |
| A | **185.1285** | 93.0679 | 1564.7682 | **782.8877** |
| P | **282.1812** | 141.5942 | 1493.7311 | **747.3692** |
| F | 429.2496 | **215.1285** | **1396.6783** | **698.8428** |
| E | **558.2922** | **279.6498** | **1249.6099** | 625.3086 |
| F | **705.3606** | **353.1840** | **1120.5673** | **560.7873** |
| V | **804.4291** | **402.7182** | **973.4989** | **487.2531** |
| G | **861.4505** | **431.2289** | **874.4305** | **437.7189** |
| F | **1008.5189** | **504.7631** | **817.4090** | **409.2082** |
| T | **1109.5666** | **555.2869** | **670.3406** | **335.6740** |
| T | 1210.6143 | 605.8108 | **569.2930** | **285.1501** |
| S | 1297.6463 | **649.3268** | **468.2453** | 234.6263 |
| A | **1368.6834** | 684.8454 | **381.2132** | 191.1103 |
| Y | 1531.7468 | 766.3770 | **310.1761** | 155.5917 |
| K | 1659.8417 | **830.4245** | **147.1128** | 74.0600 |

*Peptide sequence 4:* IIEKPMHIGFLTMEPK

| Residue | b | b+2 | y | y+2 |
| --- | --- | --- | --- | --- |
| I | 114.0913 | 57.5493 | 1884.0122 | 942.5097 |
| I | **227.1754** | 114.0913 | 1770.9281 | 885.9677 |
| E | **356.2180** | 178.6126 | 1657.8440 | 829.4257 |
| K | **484.3130** | 242.6601 | 1528.8015 | **764.9044** |
| P | 581.3657 | 291.1865 | **1400.7065** | **700.8569** |
| M | **712.4062** | 356.7067 | **1303.6537** | 652.3305 |
| H | **849.4651** | **425.2362** | 1172.6132 | **586.8103** |
| I | **962.5492** | **481.7782** | **1035.5543** | **518.2808** |
| G | **1019.5706** | **510.2890** | **922.4703** | **461.7388** |
| F | **1166.6391** | **583.8232** | **865.4488** | 433.2280 |
| L | 1279.7231 | **640.3652** | **718.3804** | **359.6938** |
| T | 1380.7708 | **690.8890** | **605.2963** | **303.1518** |
| M | 1511.8113 | **756.4093** | **504.2486** | **252.6280** |
| E | 1640.8539 | **820.9306** | **373.2082** | **187.1077** |
| P | 1737.9066 | 869.4570 | **244.1656** | 122.5864 |
| K | 1866.0016 | 933.5044 | **147.1128** | 74.0600 |

*Peptide sequence 5:* KNDFENDYGWSK

| Residue | b | b+2 | y | y+2 |
| --- | --- | --- | --- | --- |
| K | **129.1022** | 65.0548 | 1502.6546 | **751.8310** |
| N | **243.1452** | 122.0762 | **1374.5597** | 687.7835 |
| D | **358.1721** | 179.5897 | **1260.5168** | 630.7620 |
| F | **505.2405** | 253.1239 | **1145.4898** | 573.2485 |
| E | **634.2831** | 317.6452 | **998.4214** | 499.7143 |
| N | 748.3260 | 374.6667 | **869.3788** | 435.1930 |
| D | **863.3530** | 432.1801 | **755.3359** | 378.1716 |
| Y | **1026.4163** | 513.7118 | **640.3089** | 320.6581 |
| G | **1083.4378** | 542.2225 | **477.2456** | 239.1264 |
| W | **1269.5171** | **635.2622** | **420.2241** | 210.6157 |
| S | **1356.5491** | 678.7782 | **234.1448** | 117.5761 |
| K | 1484.6441 | **742.8257** | **147.1128** | 74.0600 |

*Peptide sequence 6:* NRPQFLVGSNSLLR

| Residue | b | b+2 | y | y+2 |
| --- | --- | --- | --- | --- |
| N | 115.0502 | 58.0287 | 1600.8918 | 800.9495 |
| R | **271.1513** | 136.0793 | 1486.8489 | 743.9281 |
| P | 368.2041 | 184.6057 | 1330.7478 | **665.8775** |
| Q | **496.2627** | **248.6350** | 1233.6950 | 617.3511 |
| F | **643.3311** | **322.1692** | **1105.6364** | **553.3218** |
| L | **756.4151** | **378.7112** | **958.5680** | **479.7876** |
| V | **855.4835** | **428.2454** | **845.4839** | **423.2456** |
| G | **912.5050** | **456.7561** | **746.4155** | **373.7114** |
| S | **999.5370** | **500.2722** | **689.3941** | **345.2007** |
| N | **1113.5800** | **557.2936** | **602.3620** | **301.6847** |
| S | **1200.6120** | **600.8096** | **488.3191** | 244.6632 |
| L | **1313.6961** | **657.3517** | **401.2871** | **201.1472** |
| L | 1426.7801 | **713.8937** | **288.2030** | 144.6051 |
| R | 1582.8812 | 791.9443 | **175.1190** | 88.0631 |

*Peptide sequence 7:* TLFVPQYLDSNLLIFIR

| Residue | b | b+2 | y | y+2 |
| --- | --- | --- | --- | --- |
| T | 102.0550 | 51.5311 | 2052.1528 | 1026.5801 |
| L | **215.1390** | 108.0731 | 1951.1051 | 976.0562 |
| F | **362.2074** | 181.6074 | 1838.0211 | **919.5142** |
| V | **461.2758** | 231.1416 | 1690.9527 | **845.9800** |
| P | **558.3286** | 279.6679 | 1591.8843 | **796.4458** |
| Q | **686.3872** | 343.6972 | **1494.8315** | 747.9194 |
| Y | **849.4505** | **425.2289** | **1366.7729** | 683.8901 |
| L | **962.5346** | 481.7709 | **1203.7096** | 602.3584 |
| D | **1077.5615** | 539.2844 | **1090.6255** | 545.8164 |
| S | 1164.5936 | 582.8004 | **975.5986** | 488.3029 |
| N | **1278.6365** | 639.8219 | **888.5665** | 444.7869 |
| L | **1391.7205** | **696.3639** | **774.5236** | 387.7654 |
| L | 1504.8046 | **752.9059** | **661.4396** | 331.2234 |
| I | 1617.8887 | 809.4480 | **548.3555** | 274.6814 |
| F | 1764.9571 | 882.9822 | **435.2714** | 218.1394 |
| I | 1878.0411 | 939.5242 | **288.2030** | 144.6051 |
| R | 2034.1423 | 1017.5748 | **175.1190** | 88.0631 |

*Peptide sequence 8:* VSPGDVFWIPR

| Residue | b | b+2 | y | y+2 |
| --- | --- | --- | --- | --- |
| V | 100.0757 | 50.5415 | 1272.6735 | **636.8404** |
| S | **187.1077** | 94.0575 | **1173.6051** | **587.3062** |
| P | 284.1605 | 142.5839 | **1086.5731** | **543.7902** |
| G | **341.1819** | 171.0946 | **989.5203** | 495.2638 |
| D | **456.2089** | 228.6081 | **932.4989** | 466.7531 |
| V | **555.2773** | 278.1423 | **817.4719** | 409.2396 |
| F | **702.3457** | 351.6765 | **718.4035** | 359.7054 |
| W | **888.4250** | 444.7162 | **571.3351** | 286.1712 |
| I | **1001.5091** | 501.2582 | **385.2558** | 193.1315 |
| P | 1098.5619 | 549.7846 | **272.1717** | 136.5895 |
| R | 1254.6630 | **627.8351** | **175.1190** | 88.0631 |

*Peptide sequence 9:* NRPQFLVGSN[Dea]SLLR

| Residue | b | b+2 | y | y+2 |
| --- | --- | --- | --- | --- |
| N | 115.0502 | 58.0287 | 1601.8758 | 801.4415 |
| R | **271.1513** | **136.0793** | 1487.8329 | 744.4201 |
| P | 368.2041 | 184.6057 | 1331.7318 | 666.3695 |
| Q | **496.2627** | 248.6350 | 1234.6790 | 617.8431 |
| F | **643.3311** | **322.1692** | 1106.6204 | 553.8139 |
| L | **756.4151** | **378.7112** | **959.5520** | 480.2796 |
| V | **855.4835** | **428.2454** | **846.4680** | **423.7376** |
| G | **912.5050** | 456.7561 | **747.3995** | **374.2034** |
| S | 999.5370 | 500.2722 | **690.3781** | **345.6927** |
| N[Dea] | 1114.5640 | 557.7856 | **603.3461** | 302.1767 |
| S | 1201.5960 | 601.3016 | **488.3191** | 244.6632 |
| L | 1314.6801 | 657.8437 | **401.2871** | **201.1472** |
| L | 1427.7641 | 714.3857 | **288.2030** | 144.6051 |
| R | 1583.8653 | 792.4363 | **175.1190** | 88.0631 |

*Peptide sequence 10:* IIEKPMHIGFLTM[Oxi]EPK

| Residue | b | b+2 | y | y+2 |
| --- | --- | --- | --- | --- |
| I | 114.0913 | 57.5493 | 1900.0071 | 950.5072 |
| I | **227.1754** | 114.0913 | 1786.9230 | 893.9652 |
| E | **356.2180** | 178.6126 | 1673.8390 | 837.4231 |
| K | 484.3130 | 242.6601 | 1544.7964 | **772.9018** |
| P | 581.3657 | 291.1865 | **1416.7014** | **708.8543** |
| M | 712.4062 | 356.7067 | 1319.6486 | 660.3280 |
| H | **849.4651** | **425.2362** | 1188.6082 | 594.8077 |
| I | 962.5492 | 481.7782 | 1051.5492 | 526.2783 |
| G | 1019.5706 | **510.2890** | **938.4652** | 469.7362 |
| F | **1166.6391** | **583.8232** | 881.4437 | 441.2255 |
| L | 1279.7231 | **640.3652** | **734.3753** | 367.6913 |
| T | 1380.7708 | 690.8890 | **621.2912** | **311.1493** |
| M[Oxi] | 1527.8062 | **764.4067** | **520.2436** | **260.6254** |
| E | 1656.8488 | 828.9280 | **373.2082** | 187.1077 |
| P | 1753.9016 | 877.4544 | **244.1656** | 122.5864 |
| K | 1881.9965 | 941.5019 | **147.1128** | 74.0600 |

*Peptide sequence 11:* IIEKPM[Oxi]HIGFLTMEPK

| Residue | b | b+2 | y | y+2 |
| --- | --- | --- | --- | --- |
| I | 114.0913 | 57.5493 | 1900.0071 | 950.5072 |
| I | **227.1754** | 114.0913 | 1786.9230 | 893.9652 |
| E | **356.2180** | 178.6126 | 1673.8390 | **837.4231** |
| K | 484.3130 | 242.6601 | 1544.7964 | **772.9018** |
| P | 581.3657 | 291.1865 | 1416.7014 | **708.8543** |
| M[Oxi] | 728.4011 | 364.7042 | 1319.6486 | 660.3280 |
| H | **865.4600** | **433.2337** | 1172.6132 | 586.8103 |
| I | 978.5441 | **489.7757** | **1035.5543** | **518.2808** |
| G | **1035.5656** | **518.2864** | **922.4703** | **461.7388** |
| F | **1182.6340** | **591.8206** | **865.4488** | **433.2280** |
| L | 1295.7180 | **648.3627** | **718.3804** | **359.6938** |
| T | 1396.7657 | **698.8865** | **605.2963** | **303.1518** |
| M | 1527.8062 | **764.4067** | **504.2486** | **252.6280** |
| E | 1656.8488 | 828.9280 | **373.2082** | 187.1077 |
| P | 1753.9016 | 877.4544 | **244.1656** | 122.5864 |
| K | 1881.9965 | **941.5019** | **147.1128** | 74.0600 |

## Protein: B.rapa.Ro18.Candidate-2, 10 peptides (95%)

*Protein sequence coverage*

MIRFTVLSFFVVFVLLFACNESSAKTAKYDKSDESVENDDLAAVPSCCGFSSPLLIKKDQWKPIFANKFGQISTVQIGDGCGGMGPYKIHSITLEPNALMLPLLLHSDMVFFVDSGSGILNWVEAQATSTEIRLGDVYRLRPGTVFYLQSKPVDIFLGTKLKIYAIFSNSQECLHDPCFGAYSSVTDLLFGFDETILK**SAFGVPEEIIGLMR**NRTQPPLIVHDMLTTPGEANTDTDTNTWPLQTRLLKLFSGDASADSVENKKVKKEKKEKKEKKKKPK**KATTFNVFESEPDFQSPNGQTITINRK**DLKVLQGSMVGVSMVNLTQGSMMGPHWNPWACEISVVVRGSGMVRVLRNSISRSSSECKNMR**FKVEKGDIFAVPR**LHPMAQMSFLNDSLVFVGFTTSAKNNEPQFLAGK**NSALWSLDR**EVLAASFNVSSFMIAGLLEAQKEAAVLGCPACAEGELEKLKEDEEKKESPPQQPPQPFQPQPPGEKPQQPPQPFQPQPPQGEPQKPPQGEPQKPPQGEPEGPQKPFQPQPGQGEPQEPQASMETKMRDEERKREEEEAKKEEEERWKQEEKLWPTQPQWED

*Peptide sequence 1:* ATTFNVFESEPDFQSPNGQTITINRK

| Residue | b | b+2 | y | y+2 |
| --- | --- | --- | --- | --- |
| A | 72.0444 | 36.5258 | 2941.4326 | 1471.2200 |
| T | **173.0921** | 87.0497 | 2870.3955 | 1435.7014 |
| T | **274.1397** | 137.5735 | 2769.3478 | **1385.1776** |
| F | **421.2082** | 211.1077 | 2668.3002 | **1334.6537** |
| N | **535.2511** | 268.1292 | 2521.2318 | **1261.1195** |
| V | **634.3195** | 317.6634 | 2407.1888 | **1204.0981** |
| F | **781.3879** | 391.1976 | 2308.1204 | **1154.5638** |
| E | **910.4305** | 455.7189 | 2161.0520 | **1081.0296** |
| S | 997.4625 | 499.2349 | 2032.0094 | **1016.5083** |
| E | 1126.5051 | 563.7562 | 1944.9774 | **972.9923** |
| P | 1223.5579 | 612.2826 | 1815.9348 | **908.4710** |
| D | 1338.5848 | 669.7961 | 1718.8820 | 859.9446 |
| F | **1485.6533** | 743.3303 | 1603.8551 | **802.4312** |
| Q | 1613.7118 | **807.3596** | **1456.7867** | 728.8970 |
| S | 1700.7439 | 850.8756 | **1328.7281** | **664.8677** |
| P | 1797.7966 | 899.4019 | **1241.6961** | **621.3517** |
| N | 1911.8395 | 956.4234 | 1144.6433 | 572.8253 |
| G | 1968.8610 | 984.9341 | 1030.6004 | 515.8038 |
| Q | 2096.9196 | 1048.9634 | 973.5789 | 487.2931 |
| T | 2197.9673 | 1099.4873 | 845.5203 | 423.2638 |
| I | 2311.0513 | 1156.0293 | 744.4726 | 372.7400 |
| T | 2412.0990 | 1206.5531 | **631.3886** | 316.1979 |
| I | 2525.1831 | 1263.0952 | **530.3409** | 265.6741 |
| N | 2639.2260 | 1320.1166 | **417.2568** | 209.1321 |
| R | 2795.3271 | 1398.1672 | 303.2139 | 152.1106 |
| K | 2923.4221 | 1462.2147 | **147.1128** | 74.0600 |

*Peptide sequence 2:* FKVEKGDIFAVPR

| Residue | b | b+2 | y | y+2 |
| --- | --- | --- | --- | --- |
| F | 148.0757 | 74.5415 | 1505.8475 | 753.4274 |
| K | **276.1707** | 138.5890 | 1358.7791 | **679.8932** |
| V | 375.2391 | 188.1232 | 1230.6841 | **615.8457** |
| E | 504.2817 | 252.6445 | 1131.6157 | **566.3115** |
| K | **632.3766** | 316.6919 | **1002.5731** | **501.7902** |
| G | 689.3981 | **345.2027** | **874.4781** | 437.7427 |
| D | **804.4250** | **402.7162** | 817.4567 | 409.2320 |
| I | **917.5091** | **459.2582** | **702.4297** | 351.7185 |
| F | **1064.5775** | **532.7924** | **589.3457** | **295.1765** |
| A | 1135.6146 | **568.3109** | **442.2772** | 221.6423 |
| V | 1234.6830 | 617.8452 | **371.2401** | 186.1237 |
| P | 1331.7358 | 666.3715 | **272.1717** | 136.5895 |
| R | 1487.8369 | 744.4221 | **175.1190** | 88.0631 |

*Peptide sequence 3:* GDIFAVPR

| Residue | b | b+2 | y | y+2 |
| --- | --- | --- | --- | --- |
| G | 58.0287 | 29.5180 | 874.4781 | **437.7427** |
| D | **173.0557** | 87.0315 | 817.4567 | 409.2320 |
| I | **286.1397** | 143.5735 | **702.4297** | **351.7185** |
| F | **433.2082** | 217.1077 | **589.3457** | **295.1765** |
| A | **504.2453** | 252.6263 | **442.2772** | 221.6423 |
| V | **603.3137** | 302.1605 | **371.2401** | 186.1237 |
| P | **700.3665** | 350.6869 | **272.1717** | 136.5895 |
| R | 856.4676 | **428.7374** | **175.1190** | 88.0631 |

*Peptide sequence 4:* KATTFNVFESEPDFQSPNGQTITINR

| Residue | b | b+2 | y | y+2 |
| --- | --- | --- | --- | --- |
| K | **129.1022** | 65.0548 | 2941.4326 | 1471.2200 |
| A | **200.1394** | 100.5733 | 2813.3377 | 1407.1725 |
| T | **301.1870** | 151.0972 | 2742.3006 | 1371.6539 |
| T | **402.2347** | 201.6210 | 2641.2529 | 1321.1301 |
| F | **549.3031** | 275.1552 | 2540.2052 | 1270.6062 |
| N | **663.3461** | **332.1767** | 2393.1368 | 1197.0720 |
| V | **762.4145** | 381.7109 | 2279.0939 | 1140.0506 |
| F | 909.4829 | 455.2451 | 2180.0255 | **1090.5164** |
| E | **1038.5255** | 519.7664 | 2032.9570 | **1016.9822** |
| S | **1125.5575** | **563.2824** | 1903.9144 | 952.4609 |
| E | **1254.6001** | 627.8037 | 1816.8824 | 908.9448 |
| P | 1351.6529 | 676.3301 | 1687.8398 | **844.4236** |
| D | **1466.6798** | 733.8435 | 1590.7871 | 795.8972 |
| F | 1613.7482 | **807.3777** | 1475.7601 | **738.3837** |
| Q | 1741.8068 | **871.4070** | **1328.6917** | **664.8495** |
| S | 1828.8388 | **914.9230** | **1200.6331** | **600.8202** |
| P | 1925.8916 | **963.4494** | **1113.6011** | **557.3042** |
| N | 2039.9345 | 1020.4709 | 1016.5483 | 508.7778 |
| G | 2096.9560 | 1048.9816 | **902.5054** | 451.7563 |
| Q | 2225.0146 | 1113.0109 | 845.4839 | 423.2456 |
| T | 2326.0622 | 1163.5348 | **717.4254** | 359.2163 |
| I | 2439.1463 | 1220.0768 | **616.3777** | 308.6925 |
| T | 2540.1940 | 1270.6006 | **503.2936** | 252.1504 |
| I | 2653.2780 | 1327.1427 | **402.2459** | 201.6266 |
| N | 2767.3210 | 1384.1641 | **289.1619** | 145.0846 |
| R | 2923.4221 | 1462.2147 | **175.1190** | 88.0631 |

*Peptide sequence 5:* KATTFNVFESEPDFQSPNGQTITINRK

| Residue | b | b+2 | y | y+2 |
| --- | --- | --- | --- | --- |
| K | **129.1022** | 65.0548 | 3069.5276 | 1535.2674 |
| A | **200.1394** | 100.5733 | 2941.4326 | 1471.2200 |
| T | **301.1870** | 151.0972 | 2870.3955 | 1435.7014 |
| T | **402.2347** | 201.6210 | 2769.3478 | 1385.1776 |
| F | **549.3031** | 275.1552 | 2668.3002 | 1334.6537 |
| N | **663.3461** | **332.1767** | 2521.2318 | 1261.1195 |
| V | **762.4145** | **381.7109** | 2407.1888 | 1204.0981 |
| F | 909.4829 | **455.2451** | 2308.1204 | **1154.5638** |
| E | **1038.5255** | **519.7664** | 2161.0520 | **1081.0296** |
| S | **1125.5575** | **563.2824** | 2032.0094 | **1016.5083** |
| E | **1254.6001** | **627.8037** | 1944.9774 | **972.9923** |
| P | 1351.6529 | 676.3301 | 1815.9348 | **908.4710** |
| D | 1466.6798 | 733.8435 | 1718.8820 | **859.9446** |
| F | 1613.7482 | 807.3777 | 1603.8551 | **802.4312** |
| Q | 1741.8068 | **871.4070** | **1456.7867** | **728.8970** |
| S | 1828.8388 | 914.9230 | **1328.7281** | **664.8677** |
| P | 1925.8916 | 963.4494 | **1241.6961** | **621.3517** |
| N | 2039.9345 | **1020.4709** | **1144.6433** | **572.8253** |
| G | 2096.9560 | 1048.9816 | **1030.6004** | **515.8038** |
| Q | 2225.0146 | 1113.0109 | **973.5789** | 487.2931 |
| T | 2326.0622 | 1163.5348 | **845.5203** | 423.2638 |
| I | 2439.1463 | 1220.0768 | **744.4726** | 372.7400 |
| T | 2540.1940 | 1270.6006 | **631.3886** | **316.1979** |
| I | 2653.2780 | 1327.1427 | **530.3409** | 265.6741 |
| N | 2767.3210 | 1384.1641 | **417.2568** | 209.1321 |
| R | 2923.4221 | 1462.2147 | **303.2139** | 152.1106 |
| K | 3051.5170 | 1526.2622 | **147.1128** | 74.0600 |

*Peptide sequence 6:* NSALWSLDR

| Residue | b | b+2 | y | y+2 |
| --- | --- | --- | --- | --- |
| N | 115.0502 | 58.0287 | 1061.5374 | **531.2724** |
| S | **202.0822** | 101.5448 | **947.4945** | **474.2509** |
| A | **273.1193** | 137.0633 | **860.4625** | 430.7349 |
| L | **386.2034** | 193.6053 | **789.4254** | 395.2163 |
| W | **572.2827** | 286.6450 | **676.3413** | 338.6743 |
| S | 659.3148 | 330.1610 | **490.2620** | 245.6346 |
| L | 772.3988 | 386.7030 | **403.2300** | 202.1186 |
| D | 887.4258 | 444.2165 | **290.1459** | 145.5766 |
| R | 1043.5269 | **522.2671** | **175.1190** | 88.0631 |

*Peptide sequence 7:* SAFGVPEEIIGLMR

| Residue | b | b+2 | y | y+2 |
| --- | --- | --- | --- | --- |
| S | 88.0393 | 44.5233 | 1518.7985 | **759.9029** |
| A | **159.0764** | 80.0418 | 1431.7664 | 716.3869 |
| F | **306.1448** | 153.5761 | **1360.7293** | **680.8683** |
| G | **363.1663** | 182.0868 | **1213.6609** | 607.3341 |
| V | **462.2347** | 231.6210 | **1156.6395** | **578.8234** |
| P | **559.2875** | 280.1474 | **1057.5710** | **529.2892** |
| E | **688.3301** | 344.6687 | **960.5183** | 480.7628 |
| E | **817.3727** | 409.1900 | **831.4757** | **416.2415** |
| I | **930.4567** | 465.7320 | **702.4331** | 351.7202 |
| I | **1043.5408** | 522.2740 | **589.3490** | 295.1782 |
| G | **1100.5623** | 550.7848 | **476.2650** | 238.6361 |
| L | **1213.6463** | 607.3268 | **419.2435** | 210.1254 |
| M | **1344.6868** | 672.8470 | **306.1594** | 153.5834 |
| R | 1500.7879 | **750.8976** | **175.1190** | 88.0631 |

*Peptide sequence 8:* KATTFNVFESEPDFQSPN[Dea]GQTITINRK

| Residue | b | b+2 | y | y+2 |
| --- | --- | --- | --- | --- |
| K | **129.1022** | 65.0548 | 3070.5116 | 1535.7594 |
| A | **200.1394** | 100.5733 | 2942.4167 | **1471.7120** |
| T | **301.1870** | 151.0972 | 2871.3795 | 1436.1934 |
| T | **402.2347** | 201.6210 | 2770.3319 | 1385.6696 |
| F | **549.3031** | 275.1552 | 2669.2842 | 1335.1457 |
| N | **663.3461** | **332.1767** | 2522.2158 | 1261.6115 |
| V | **762.4145** | **381.7109** | 2408.1728 | 1204.5901 |
| F | 909.4829 | **455.2451** | 2309.1044 | **1155.0559** |
| E | **1038.5255** | **519.7664** | 2162.0360 | **1081.5216** |
| S | **1125.5575** | **563.2824** | 2032.9934 | **1017.0004** |
| E | **1254.6001** | **627.8037** | 1945.9614 | 973.4843 |
| P | 1351.6529 | 676.3301 | 1816.9188 | **908.9630** |
| D | **1466.6798** | 733.8435 | 1719.8660 | **860.4367** |
| F | 1613.7482 | 807.3777 | 1604.8391 | **802.9232** |
| Q | 1741.8068 | 871.4070 | **1457.7707** | **729.3890** |
| S | 1828.8388 | 914.9230 | **1329.7121** | **665.3597** |
| P | 1925.8916 | 963.4494 | **1242.6801** | **621.8437** |
| N[Dea] | 2040.9185 | 1020.9629 | **1145.6273** | **573.3173** |
| G | 2097.9400 | 1049.4736 | **1030.6004** | **515.8038** |
| Q | 2225.9986 | 1113.5029 | 973.5789 | 487.2931 |
| T | 2327.0462 | 1164.0268 | **845.5203** | **423.2638** |
| I | 2440.1303 | 1220.5688 | **744.4726** | 372.7400 |
| T | 2541.1780 | 1271.0926 | **631.3886** | 316.1979 |
| I | 2654.2621 | 1327.6347 | **530.3409** | 265.6741 |
| N | 2768.3050 | 1384.6561 | **417.2568** | 209.1321 |
| R | 2924.4061 | 1462.7067 | 303.2139 | 152.1106 |
| K | 3052.5011 | 1526.7542 | **147.1128** | 74.0600 |

*Peptide sequence 9:* KATTFNVFESEPDFQSPNGQ[Dea]TITINRK

| Residue | b | b+2 | y | y+2 |
| --- | --- | --- | --- | --- |
| K | **129.1022** | 65.0548 | 3070.5116 | 1535.7594 |
| A | 200.1394 | 100.5733 | 2942.4167 | 1471.7120 |
| T | 301.1870 | 151.0972 | 2871.3795 | 1436.1934 |
| T | **402.2347** | 201.6210 | 2770.3319 | 1385.6696 |
| F | **549.3031** | 275.1552 | 2669.2842 | 1335.1457 |
| N | **663.3461** | 332.1767 | 2522.2158 | 1261.6115 |
| V | **762.4145** | **381.7109** | 2408.1728 | 1204.5901 |
| F | **909.4829** | 455.2451 | 2309.1044 | **1155.0559** |
| E | **1038.5255** | **519.7664** | 2162.0360 | **1081.5216** |
| S | **1125.5575** | **563.2824** | 2032.9934 | **1017.0004** |
| E | **1254.6001** | 627.8037 | 1945.9614 | 973.4843 |
| P | 1351.6529 | 676.3301 | 1816.9188 | **908.9630** |
| D | 1466.6798 | 733.8435 | 1719.8660 | **860.4367** |
| F | 1613.7482 | 807.3777 | 1604.8391 | **802.9232** |
| Q | 1741.8068 | 871.4070 | 1457.7707 | **729.3890** |
| S | 1828.8388 | 914.9230 | **1329.7121** | **665.3597** |
| P | 1925.8916 | 963.4494 | **1242.6801** | **621.8437** |
| N | 2039.9345 | 1020.4709 | **1145.6273** | 573.3173 |
| G | 2096.9560 | 1048.9816 | **1031.5844** | 516.2958 |
| Q[Dea] | 2225.9986 | 1113.5029 | 974.5629 | 487.7851 |
| T | 2327.0462 | 1164.0268 | **845.5203** | 423.2638 |
| I | 2440.1303 | 1220.5688 | **744.4726** | 372.7400 |
| T | 2541.1780 | 1271.0926 | **631.3886** | **316.1979** |
| I | 2654.2621 | 1327.6347 | **530.3409** | 265.6741 |
| N | 2768.3050 | 1384.6561 | **417.2568** | 209.1321 |
| R | 2924.4061 | 1462.7067 | 303.2139 | 152.1106 |
| K | 3052.5011 | 1526.7542 | 147.1128 | 74.0600 |

*Peptide sequence 10:* SAFGVPEEIIGLM[Oxi]R

| Residue | b | b+2 | y | y+2 |
| --- | --- | --- | --- | --- |
| S | 88.0393 | 44.5233 | 1534.7934 | 767.9003 |
| A | **159.0764** | 80.0418 | 1447.7614 | 724.3843 |
| F | **306.1448** | 153.5761 | 1376.7242 | **688.8658** |
| G | **363.1663** | 182.0868 | 1229.6558 | 615.3316 |
| V | **462.2347** | 231.6210 | 1172.6344 | 586.8208 |
| P | 559.2875 | 280.1474 | **1073.5660** | **537.2866** |
| E | **688.3301** | 344.6687 | **976.5132** | 488.7602 |
| E | **817.3727** | **409.1900** | 847.4706 | 424.2389 |
| I | **930.4567** | 465.7320 | **718.4280** | 359.7176 |
| I | 1043.5408 | 522.2740 | **605.3439** | **303.1756** |
| G | 1100.5623 | 550.7848 | **492.2599** | 246.6336 |
| L | 1213.6463 | 607.3268 | **435.2384** | 218.1228 |
| M[Oxi] | 1360.6817 | 680.8445 | **322.1544** | 161.5808 |
| R | 1516.7828 | 758.8951 | 175.1190 | 88.0631 |

## Protein: B.rapa.Ro18.Candidate-3, 3 peptides (95%)

*Protein sequence coverage*

MTKFTVLPLFVLLFLVLLCTKSWAKSEEFDESSDEENDVAAVPSCCGFSSPLLIKKDQWKPIFGTQFGQISTVQIGEGCGGMGPYKIHSITLEPNALLLPLLLHSDMVFFVESGSGILNWVEAEPTSSEIRRGDVYRLRPGTVFYLQSKPIDIFLGTKLRVYAIFSNTEECLHDPCFGAYSSITDLLFGFDEAILQSAFGVPEEIIGLMTNRTQPPLIVHDMLSTPGEANTYTWQLQVQPRLLKLFAGYVSAAEKKKKEKKTKKAK**TFNVFESEPDFQSPSGR**TITINRKDLEVLSGSMVGVSMVNLTQASMMGPHWNPWACEISIVLKGSGMVRVLRSSISSTSSSSSSSECKNMR**FKVEEGDIFAVPR**LHPMAQMSFINESLVFIGFTTSARNNEPQFLAGQRSALRLLDQEVLAASLNVSSVMIEGLLGAQKDAVVLGCPYCAEGELEKLKVETEMKKRDDERKREEEEAKKEEEERRKREEEEEEEKQWPPLPQQPPE

*Peptide sequence 1:* FKVEEGDIFAVPR

| Residue | b | b+2 | y | y+2 |
| --- | --- | --- | --- | --- |
| F | 148.0757 | 74.5415 | 1506.7951 | 753.9012 |
| K | **276.1707** | 138.5890 | 1359.7267 | 680.3670 |
| V | **375.2391** | 188.1232 | 1231.6317 | 616.3195 |
| E | **504.2817** | 252.6445 | 1132.5633 | 566.7853 |
| E | **633.3243** | 317.1658 | 1003.5207 | 502.2640 |
| G | **690.3457** | 345.6765 | 874.4781 | 437.7427 |
| D | **805.3727** | **403.1900** | 817.4567 | 409.2320 |
| I | **918.4567** | **459.7320** | **702.4297** | 351.7185 |
| F | **1065.5251** | **533.2662** | **589.3457** | **295.1765** |
| A | 1136.5623 | **568.7848** | **442.2772** | 221.6423 |
| V | 1235.6307 | 618.3190 | **371.2401** | 186.1237 |
| P | 1332.6834 | 666.8454 | **272.1717** | 136.5895 |
| R | 1488.7845 | 744.8959 | **175.1190** | 88.0631 |

*Peptide sequence 2:* GDIFAVPR

| Residue | b | b+2 | y | y+2 |
| --- | --- | --- | --- | --- |
| G | 58.0287 | 29.5180 | 874.4781 | **437.7427** |
| D | **173.0557** | 87.0315 | 817.4567 | 409.2320 |
| I | **286.1397** | 143.5735 | **702.4297** | **351.7185** |
| F | **433.2082** | 217.1077 | **589.3457** | **295.1765** |
| A | **504.2453** | 252.6263 | **442.2772** | 221.6423 |
| V | **603.3137** | 302.1605 | **371.2401** | 186.1237 |
| P | **700.3665** | 350.6869 | **272.1717** | 136.5895 |
| R | 856.4676 | **428.7374** | **175.1190** | 88.0631 |

*Peptide sequence 3:* TFNVFESEPDFQSPSGR

| Residue | b | b+2 | y | y+2 |
| --- | --- | --- | --- | --- |
| T | 102.0550 | 51.5311 | 1943.8770 | **972.4421** |
| F | **249.1234** | 125.0653 | 1842.8293 | 921.9183 |
| N | **363.1663** | 182.0868 | 1695.7609 | 848.3841 |
| V | **462.2347** | 231.6210 | 1581.7180 | 791.3626 |
| F | **609.3031** | 305.1552 | **1482.6496** | **741.8284** |
| E | **738.3457** | 369.6765 | **1335.5811** | 668.2942 |
| S | **825.3777** | 413.1925 | **1206.5386** | 603.7729 |
| E | **954.4203** | 477.7138 | **1119.5065** | 560.2569 |
| P | 1051.4731 | 526.2402 | **990.4639** | **495.7356** |
| D | **1166.5000** | 583.7537 | **893.4112** | **447.2092** |
| F | **1313.5685** | 657.2879 | **778.3842** | 389.6958 |
| Q | **1441.6270** | **721.3172** | **631.3158** | **316.1615** |
| S | 1528.6591 | 764.8332 | **503.2572** | 252.1323 |
| P | 1625.7118 | 813.3596 | **416.2252** | 208.6162 |
| S | 1712.7439 | 856.8756 | **319.1724** | 160.0899 |
| G | 1769.7653 | 885.3863 | 232.1404 | 116.5738 |
| R | 1925.8664 | **963.4369** | **175.1190** | 88.0631 |

## Protein: B.rapa.Ro18.Candidate-4, 2 peptides (95%)

*Protein sequence coverage*

MSKFTIIPLCLLTLFLCTNSFSDQNDGVPSSQSPLLVKRHQRTQLVATEFGEISAVHIGEEYTIQFITLEPNALLLPLLLHSDMVFFVHTGSGVLNWVDEEKERTLELKRGDVFRLRYGTVFYLHCNLERDEVPEKLRVYAIFDVGKCLSDQCLGAYSSIRDLLWGFDEKTLRSAFAVPKDVFGRLRDAVKPPLITHAMPKNRTQGSEEETWGSRLAKLFVRVEDVTDHLEMKPVVNKKKKKKKKKSSAYNVFESDPDFENDNGQSIVVDEKDMDALKGSSFGVYMVNLTKGSMMGPHWNPNACEISIVLQGEGMIRVVNHPSYQSKNESERFMVEDGDVFVVPQFYPMAQLSFVNSSFMFMGFSTSAK**TNHPQFLVGQNSVLK**IFNR**DVLATSFNMR**YATVERLLGAQKDGLLLECVSCAEVELSRLMREIEERRRREEEEIERRKREEEEAKRQEEERRRREEEEAERKKKAEEEARKREKEREREEEAAKRREEERRRREEEEAERKRKEEEEARKREEERKREEEAAKKREEERRKREKEEEEARKREEAREREEEEAKKREEERRKREEEEAERKRRAEEEAREREEEEAKKREEEKEAARRREEEREKEEEMAKRREEERQRKEREDVERKKREEEEERKRREEEAMRREEERKREEEAAKRAEEERRKREEEAEHKKRPPPQGPQPPIHH

*Peptide sequence 1:* TNHPQFLVGQNSVLK

| Residue | b | b+2 | y | y+2 |
| --- | --- | --- | --- | --- |
| T | 102.0550 | 51.5311 | 1681.9020 | 841.4547 |
| N | 216.0979 | 108.5526 | 1580.8544 | 790.9308 |
| H | 353.1568 | 177.0820 | 1466.8114 | 733.9094 |
| P | **450.2096** | 225.6084 | 1329.7525 | 665.3799 |
| Q | 578.2681 | 289.6377 | 1232.6997 | 616.8535 |
| F | **725.3365** | **363.1719** | 1104.6412 | 552.8242 |
| L | **838.4206** | **419.7139** | 957.5728 | 479.2900 |
| V | **937.4890** | **469.2482** | **844.4887** | **422.7480** |
| G | 994.5105 | **497.7589** | **745.4203** | 373.2138 |
| Q | 1122.5691 | **561.7882** | **688.3988** | 344.7030 |
| N | 1236.6120 | 618.8096 | 560.3402 | 280.6738 |
| S | 1323.6440 | **662.3257** | 446.2973 | 223.6523 |
| V | 1422.7124 | 711.8599 | 359.2653 | 180.1363 |
| L | 1535.7965 | 768.4019 | 260.1969 | 130.6021 |
| K | 1663.8915 | 832.4494 | **147.1128** | 74.0600 |

*Peptide sequence 1:* DVLATSFNM[Oxi]R

| Residue | b | b+2 | y | y+2 |
| --- | --- | --- | --- | --- |
| D | 116.0342 | 58.5207 | 1169.5619 | **585.2846** |
| V | **215.1026** | 108.0550 | 1054.5350 | **527.7711** |
| L | **328.1867** | 164.5970 | **955.4666** | **478.2369** |
| A | **399.2238** | 200.1155 | **842.3825** | 421.6949 |
| T | **500.2715** | 250.6394 | **771.3454** | 386.1763 |
| S | 587.3035 | 294.1554 | **670.2977** | 335.6525 |
| F | 734.3719 | 367.6896 | 583.2657 | 292.1365 |
| N | 848.4149 | 424.7111 | **436.1973** | 218.6023 |
| M[Oxi] | 995.4503 | 498.2288 | 322.1544 | 161.5808 |
| R | 1151.5514 | **576.2793** | **175.1190** | 88.0631 |

## Protein: B.rapa.Ro18.Candidate-5, 21 peptides (95%)

*Protein sequence coverage*

MAINKLTITLFLLISLAVFHCLAFRVEVQEFEPPRQEGQEGPGGGSGEGWDEEATK**NPYHFGQWSFKNFFQSK**DGFVKMLPKFTKR**SSTLFR**GIENYRFLFQEMQPNTFLVPHHLDADYVFLVVQGK**GVIGFVTDTANESFQITK**GDVVR**VPSSVTHFFANTNGTVPLR**LAK**IAVPANVPGHFQVFFPAHSGFHQSYFNGFSKDVLTASFNIPEELLGRLIRGPQQEVGQGIIRRVSPEQIK**ELTEHEHATSPSNKHKDKKDKHKDKDR**STFGSPFNLLTQDAIYSNNFGR**YHEAHPKRFSQLQDLDIAVGWVNMTQGSLFLPQYNSETTFVTFVENGCARYEMASPYTFQGEQQQPWFGPGQEEEVEEEMSGQVHKIVSRVCKGEVFILPAGHPFAILSQDENFVAVGFGIHASNSTR**TFLAGQDNMLSNINTVATRLSFGLGSK**MAEKLFTSQNYSHFAPTTPSHQFPEKPKPSFQSVFNLVGF

*Peptide sequence 1:* DVLTASFNIPEELLGR

| Residue | b | b+2 | y | y+2 |
| --- | --- | --- | --- | --- |
| D | 116.0342 | 58.5207 | 1773.9381 | 887.4727 |
| V | **215.1026** | 108.0550 | 1658.9112 | 829.9592 |
| L | **328.1867** | 164.5970 | 1559.8428 | **780.4250** |
| T | **429.2344** | **215.1208** | 1446.7587 | **723.8830** |
| A | **500.2715** | 250.6394 | **1345.7110** | **673.3592** |
| S | **587.3035** | 294.1554 | **1274.6739** | 637.8406 |
| F | **734.3719** | 367.6896 | **1187.6419** | 594.3246 |
| N | **848.4149** | **424.7111** | **1040.5735** | 520.7904 |
| I | **961.4989** | **481.2531** | **926.5306** | **463.7689** |
| P | 1058.5517 | 529.7795 | **813.4465** | **407.2269** |
| E | 1187.5943 | 594.3008 | **716.3937** | 358.7005 |
| E | **1316.6369** | 658.8221 | **587.3511** | 294.1792 |
| L | **1429.7209** | **715.3641** | **458.3085** | 229.6579 |
| L | 1542.8050 | **771.9061** | **345.2245** | **173.1159** |
| G | 1599.8265 | 800.4169 | **232.1404** | 116.5738 |
| R | 1755.9276 | 878.4674 | **175.1190** | 88.0631 |

*Peptide sequence 2:* GPQQEVGQGIIR

| Residue | b | b+2 | y | y+2 |
| --- | --- | --- | --- | --- |
| G | 58.0287 | 29.5180 | 1281.6910 | **641.3491** |
| P | **155.0815** | 78.0444 | 1224.6695 | **612.8384** |
| Q | **283.1401** | 142.0737 | **1127.6167** | **564.3120** |
| Q | **411.1987** | 206.1030 | **999.5582** | **500.2827** |
| E | **540.2413** | 270.6243 | **871.4996** | 436.2534 |
| V | **639.3097** | 320.1585 | **742.4570** | 371.7321 |
| G | **696.3311** | 348.6692 | **643.3886** | 322.1979 |
| Q | **824.3897** | 412.6985 | **586.3671** | 293.6872 |
| G | **881.4112** | **441.2092** | **458.3085** | 229.6579 |
| I | **994.4952** | 497.7513 | **401.2871** | **201.1472** |
| I | **1107.5793** | 554.2933 | **288.2030** | 144.6051 |
| R | 1263.6804 | **632.3438** | **175.1190** | 88.0631 |

*Peptide sequence 3:* GVIGFVTDTANESFQITK

| Residue | b | b+2 | y | y+2 |
| --- | --- | --- | --- | --- |
| G | 58.0287 | 29.5180 | 1926.9807 | 963.9940 |
| V | **157.0972** | 79.0522 | 1869.9593 | 935.4833 |
| I | **270.1812** | 135.5942 | 1770.8909 | **885.9491** |
| G | **327.2027** | 164.1050 | 1657.8068 | **829.4070** |
| F | **474.2711** | 237.6392 | 1600.7853 | **800.8963** |
| V | **573.3395** | 287.1734 | **1453.7169** | **727.3621** |
| T | **674.3872** | 337.6972 | **1354.6485** | **677.8279** |
| D | **789.4141** | 395.2107 | **1253.6008** | **627.3040** |
| T | **890.4618** | 445.7345 | **1138.5739** | **569.7906** |
| A | **961.4989** | 481.2531 | **1037.5262** | **519.2667** |
| N | **1075.5419** | 538.2746 | **966.4891** | **483.7482** |
| E | **1204.5844** | 602.7959 | **852.4462** | 426.7267 |
| S | 1291.6165 | **646.3119** | **723.4036** | **362.2054** |
| F | 1438.6849 | 719.8461 | **636.3715** | 318.6894 |
| Q | 1566.7435 | 783.8754 | **489.3031** | 245.1552 |
| I | 1679.8275 | 840.4174 | **361.2445** | 181.1259 |
| T | 1780.8752 | 890.9412 | **248.1605** | 124.5839 |
| K | 1908.9702 | 954.9887 | **147.1128** | 74.0600 |

*Peptide sequence 4:* IAVPANVPGHFQVFFPAHSGFHQSYFNGFSK

| Residue | b | b+2 | y | y+2 |
| --- | --- | --- | --- | --- |
| I | 114.0913 | 57.5493 | 3434.6858 | 1717.8465 |
| A | **185.1285** | 93.0679 | 3321.6017 | 1661.3045 |
| V | **284.1969** | 142.6021 | 3250.5646 | 1625.7859 |
| P | 381.2496 | 191.1285 | 3151.4962 | 1576.2517 |
| A | **452.2867** | 226.6470 | 3054.4434 | 1527.7253 |
| N | **566.3297** | 283.6685 | 2983.4063 | 1492.2068 |
| V | **665.3981** | 333.2027 | 2869.3634 | 1435.1853 |
| P | 762.4509 | 381.7291 | 2770.2950 | **1385.6511** |
| G | 819.4723 | 410.2398 | 2673.2422 | **1337.1247** |
| H | 956.5312 | 478.7693 | 2616.2207 | **1308.6140** |
| F | 1103.5996 | **552.3035** | 2479.1618 | **1240.0846** |
| Q | 1231.6582 | 616.3327 | 2332.0934 | **1166.5503** |
| V | 1330.7266 | 665.8670 | 2204.0348 | **1102.5211** |
| F | **1477.7950** | **739.4012** | 2104.9664 | **1052.9868** |
| F | 1624.8635 | 812.9354 | 1957.8980 | **979.4526** |
| P | 1721.9162 | 861.4618 | 1810.8296 | **905.9184** |
| A | 1792.9533 | 896.9803 | 1713.7768 | **857.3921** |
| H | 1930.0122 | 965.5098 | 1642.7397 | **821.8735** |
| S | 2017.0443 | 1009.0258 | 1505.6808 | **753.3440** |
| G | 2074.0657 | 1037.5365 | **1418.6488** | **709.8280** |
| F | 2221.1342 | 1111.0707 | **1361.6273** | 681.3173 |
| H | 2358.1931 | 1179.6002 | **1214.5589** | **607.7831** |
| Q | 2486.2516 | 1243.6295 | **1077.5000** | **539.2536** |
| S | 2573.2837 | 1287.1455 | **949.4414** | **475.2243** |
| Y | 2736.3470 | 1368.6771 | **862.4094** | **431.7083** |
| F | 2883.4154 | 1442.2113 | **699.3461** | 350.1767 |
| N | 2997.4583 | 1499.2328 | **552.2776** | 276.6425 |
| G | 3054.4798 | 1527.7435 | **438.2347** | 219.6210 |
| F | 3201.5482 | 1601.2777 | **381.2132** | 191.1103 |
| S | 3288.5802 | 1644.7938 | **234.1448** | 117.5761 |
| K | 3416.6752 | 1708.8412 | **147.1128** | 74.0600 |

*Peptide sequence 5:* LIRGPQQEVGQGIIR

| Residue | b | b+2 | y | y+2 |
| --- | --- | --- | --- | --- |
| L | 114.0913 | 57.5493 | 1663.9602 | 832.4837 |
| I | **227.1754** | 114.0913 | 1550.8761 | 775.9417 |
| R | **383.2765** | 192.1419 | 1437.7921 | **719.3997** |
| G | **440.2980** | 220.6526 | 1281.6910 | **641.3491** |
| P | 537.3507 | 269.1790 | 1224.6695 | 612.8384 |
| Q | **665.4093** | **333.2083** | 1127.6167 | 564.3120 |
| Q | **793.4679** | **397.2376** | **999.5582** | 500.2827 |
| E | **922.5105** | **461.7589** | **871.4996** | 436.2534 |
| V | **1021.5789** | **511.2931** | **742.4570** | **371.7321** |
| G | 1078.6004 | 539.8038 | **643.3886** | **322.1979** |
| Q | 1206.6589 | 603.8331 | **586.3671** | 293.6872 |
| G | 1263.6804 | 632.3438 | **458.3085** | 229.6579 |
| I | 1376.7645 | 688.8859 | **401.2871** | **201.1472** |
| I | 1489.8485 | 745.4279 | **288.2030** | 144.6051 |
| R | 1645.9496 | 823.4785 | **175.1190** | 88.0631 |

*Peptide sequence 6:* LSFGLGSK

| Residue | b | b+2 | y | y+2 |
| --- | --- | --- | --- | --- |
| L | 114.0913 | 57.5493 | 808.4563 | **404.7318** |
| S | **201.1234** | 101.0653 | **695.3723** | **348.1898** |
| F | **348.1918** | 174.5995 | **608.3402** | **304.6738** |
| G | **405.2132** | 203.1103 | **461.2718** | 231.1396 |
| L | **518.2973** | 259.6523 | **404.2504** | 202.6288 |
| G | **575.3188** | 288.1630 | **291.1663** | 146.0868 |
| S | **662.3508** | 331.6790 | **234.1448** | 117.5761 |
| K | 790.4458 | **395.7265** | **147.1128** | 74.0600 |

*Peptide sequence 7:* NPYHFGQWSFK

| Residue | b | b+2 | y | y+2 |
| --- | --- | --- | --- | --- |
| N | 115.0502 | 58.0287 | 1410.6589 | 705.8331 |
| P | 212.1030 | 106.5551 | 1296.6160 | **648.8116** |
| Y | 375.1663 | 188.0868 | 1199.5633 | **600.2853** |
| H | 512.2252 | 256.6162 | **1036.4999** | **518.7536** |
| F | 659.2936 | 330.1504 | **899.4410** | 450.2241 |
| G | 716.3151 | 358.6612 | **752.3726** | 376.6899 |
| Q | 844.3737 | 422.6905 | **695.3511** | 348.1792 |
| W | 1030.4530 | 515.7301 | **567.2926** | **284.1499** |
| S | 1117.4850 | 559.2461 | **381.2132** | 191.1103 |
| F | 1264.5534 | 632.7803 | **294.1812** | 147.5942 |
| K | 1392.6484 | 696.8278 | **147.1128** | 74.0600 |

*Peptide sequence 8:* RVSPEQIK

| Residue | b | b+2 | y | y+2 |
| --- | --- | --- | --- | --- |
| R | **157.1084** | 79.0578 | 956.5524 | **478.7798** |
| V | **256.1768** | 128.5920 | **800.4512** | 400.7293 |
| S | **343.2088** | 172.1081 | **701.3828** | 351.1951 |
| P | 440.2616 | 220.6344 | **614.3508** | 307.6790 |
| E | **569.3042** | **285.1557** | 517.2980 | 259.1527 |
| Q | **697.3628** | **349.1850** | **388.2554** | 194.6314 |
| I | **810.4468** | 405.7271 | **260.1969** | 130.6021 |
| K | 938.5418 | **469.7745** | **147.1128** | 74.0600 |

*Peptide sequence 9:* STFGSPFNLLTQDAIYSNNFGR

| Residue | b | b+2 | y | y+2 |
| --- | --- | --- | --- | --- |
| S | 88.0393 | 44.5233 | 2449.1783 | 1225.0928 |
| T | **189.0870** | 95.0471 | 2362.1462 | 1181.5768 |
| F | **336.1554** | 168.5813 | 2261.0986 | **1131.0529** |
| G | **393.1769** | 197.0921 | 2114.0301 | **1057.5187** |
| S | **480.2089** | 240.6081 | 2057.0087 | **1029.0080** |
| P | **577.2617** | 289.1345 | 1969.9767 | **985.4920** |
| F | **724.3301** | 362.6687 | 1872.9239 | 936.9656 |
| N | **838.3730** | **419.6901** | 1725.8555 | **863.4314** |
| L | **951.4571** | **476.2322** | 1611.8125 | **806.4099** |
| L | **1064.5411** | **532.7742** | **1498.7285** | **749.8679** |
| T | **1165.5888** | **583.2980** | **1385.6444** | **693.3258** |
| Q | **1293.6474** | **647.3273** | **1284.5967** | **642.8020** |
| D | **1408.6743** | **704.8408** | **1156.5382** | **578.7727** |
| A | **1479.7114** | **740.3594** | **1041.5112** | **521.2592** |
| I | 1592.7955 | **796.9014** | **970.4741** | **485.7407** |
| Y | 1755.8588 | **878.4331** | **857.3900** | **429.1987** |
| S | 1842.8909 | **921.9491** | **694.3267** | **347.6670** |
| N | 1956.9338 | 978.9705 | **607.2947** | **304.1510** |
| N | 2070.9767 | 1035.9920 | **493.2518** | **247.1295** |
| F | 2218.0451 | **1109.5262** | **379.2088** | **190.1081** |
| G | 2275.0666 | 1138.0369 | **232.1404** | 116.5738 |
| R | 2431.1677 | 1216.0875 | **175.1190** | 88.0631 |

*Peptide sequence 10:* TFLAGQDNMLSNINTVATR

| Residue | b | b+2 | y | y+2 |
| --- | --- | --- | --- | --- |
| T | 102.0550 | 51.5311 | 2066.0335 | 1033.5204 |
| F | **249.1234** | 125.0653 | 1964.9858 | 982.9966 |
| L | **362.2074** | 181.6074 | 1817.9174 | **909.4623** |
| A | **433.2445** | 217.1259 | 1704.8334 | **852.9203** |
| G | **490.2660** | 245.6366 | 1633.7962 | **817.4018** |
| Q | **618.3246** | 309.6659 | 1576.7748 | 788.8910 |
| D | **733.3515** | 367.1794 | 1448.7162 | 724.8617 |
| N | **847.3945** | **424.2009** | **1333.6893** | 667.3483 |
| M | **978.4349** | **489.7211** | **1219.6463** | 610.3268 |
| L | **1091.5190** | **546.2631** | **1088.6058** | **544.8066** |
| S | **1178.5510** | **589.7792** | **975.5218** | **488.2645** |
| N | **1292.5940** | 646.8006 | **888.4898** | **444.7485** |
| I | **1405.6780** | **703.3427** | **774.4468** | **387.7271** |
| N | 1519.7210 | 760.3641 | **661.3628** | 331.1850 |
| T | 1620.7686 | **810.8880** | **547.3198** | 274.1636 |
| V | 1719.8370 | 860.4222 | **446.2722** | 223.6397 |
| A | 1790.8742 | 895.9407 | **347.2037** | 174.1055 |
| T | 1891.9218 | 946.4646 | **276.1666** | 138.5870 |
| R | 2048.0229 | 1024.5151 | **175.1190** | 88.0631 |

*Peptide sequence 11:* VPSSVTHFFAN[Dea]TNGTVPLR

| Residue | b | b+2 | y | y+2 |
| --- | --- | --- | --- | --- |
| V | 100.0757 | 50.5415 | 2045.0451 | 1023.0262 |
| P | **197.1285** | 99.0679 | 1945.9767 | **973.4920** |
| S | **284.1605** | 142.5839 | 1848.9239 | **924.9656** |
| S | 371.1925 | 186.0999 | 1761.8919 | **881.4496** |
| V | 470.2609 | 235.6341 | 1674.8598 | 837.9336 |
| T | 571.3086 | 286.1579 | 1575.7914 | 788.3993 |
| H | **708.3675** | 354.6874 | 1474.7437 | 737.8755 |
| F | 855.4359 | **428.2216** | 1337.6848 | 669.3461 |
| F | 1002.5043 | **501.7558** | **1190.6164** | **595.8118** |
| A | **1073.5415** | **537.2744** | 1043.5480 | 522.2776 |
| N[Dea] | **1188.5684** | **594.7878** | **972.5109** | 486.7591 |
| T | **1289.6161** | **645.3117** | **857.4839** | 429.2456 |
| N | 1403.6590 | 702.3331 | **756.4363** | 378.7218 |
| G | 1460.6805 | 730.8439 | **642.3933** | 321.7003 |
| T | 1561.7281 | **781.3677** | **585.3719** | 293.1896 |
| V | 1660.7966 | 830.9019 | **484.3242** | 242.6657 |
| P | 1757.8493 | 879.4283 | **385.2558** | 193.1315 |
| L | 1870.9334 | 935.9703 | 288.2030 | 144.6051 |
| R | 2027.0345 | 1014.0209 | **175.1190** | 88.0631 |

*Peptide sequence 12:* VPSSVTHFFANTN[Dea]GTVPLR

| Residue | b | b+2 | y | y+2 |
| --- | --- | --- | --- | --- |
| V | 100.0757 | 50.5415 | 2045.0451 | 1023.0262 |
| P | **197.1285** | 99.0679 | 1945.9767 | **973.4920** |
| S | **284.1605** | 142.5839 | 1848.9239 | **924.9656** |
| S | 371.1925 | 186.0999 | 1761.8919 | **881.4496** |
| V | 470.2609 | 235.6341 | 1674.8598 | 837.9336 |
| T | 571.3086 | 286.1579 | 1575.7914 | 788.3993 |
| H | **708.3675** | 354.6874 | 1474.7437 | 737.8755 |
| F | 855.4359 | **428.2216** | 1337.6848 | 669.3461 |
| F | 1002.5043 | **501.7558** | **1190.6164** | **595.8118** |
| A | **1073.5415** | **537.2744** | 1043.5480 | 522.2776 |
| N | **1187.5844** | **594.2958** | **972.5109** | 486.7591 |
| T | **1288.6321** | **644.8197** | **858.4680** | 429.7376 |
| N[Dea] | 1403.6590 | 702.3331 | **757.4203** | 379.2138 |
| G | 1460.6805 | 730.8439 | **642.3933** | 321.7003 |
| T | 1561.7281 | **781.3677** | **585.3719** | 293.1896 |
| V | 1660.7966 | 830.9019 | **484.3242** | 242.6657 |
| P | 1757.8493 | 879.4283 | **385.2558** | 193.1315 |
| L | 1870.9334 | 935.9703 | 288.2030 | 144.6051 |
| R | 2027.0345 | 1014.0209 | **175.1190** | 88.0631 |

*Peptide sequence 13:* IAVPANVPGHFQVFFPAHSGFHQSYFN[Dea]GFSK

| Residue | b | b+2 | y | y+2 |
| --- | --- | --- | --- | --- |
| I | 114.0913 | 57.5493 | 3435.6698 | 1718.3385 |
| A | **185.1285** | 93.0679 | 3322.5857 | 1661.7965 |
| V | **284.1969** | 142.6021 | 3251.5486 | 1626.2779 |
| P | 381.2496 | 191.1285 | 3152.4802 | 1576.7437 |
| A | 452.2867 | 226.6470 | 3055.4274 | 1528.2174 |
| N | 566.3297 | 283.6685 | 2984.3903 | 1492.6988 |
| V | 665.3981 | 333.2027 | 2870.3474 | 1435.6773 |
| P | 762.4509 | 381.7291 | 2771.2790 | **1386.1431** |
| G | 819.4723 | 410.2398 | 2674.2262 | 1337.6167 |
| H | 956.5312 | 478.7693 | 2617.2048 | 1309.1060 |
| F | 1103.5996 | 552.3035 | 2480.1458 | **1240.5766** |
| Q | 1231.6582 | 616.3327 | 2333.0774 | **1167.0424** |
| V | 1330.7266 | 665.8670 | 2205.0189 | **1103.0131** |
| F | 1477.7950 | **739.4012** | 2105.9504 | **1053.4789** |
| F | 1624.8635 | 812.9354 | 1958.8820 | **979.9446** |
| P | 1721.9162 | 861.4618 | 1811.8136 | **906.4104** |
| A | 1792.9533 | 896.9803 | 1714.7608 | **857.8841** |
| H | 1930.0122 | 965.5098 | 1643.7237 | **822.3655** |
| S | 2017.0443 | 1009.0258 | 1506.6648 | **753.8360** |
| G | 2074.0657 | 1037.5365 | **1419.6328** | **710.3200** |
| F | 2221.1342 | 1111.0707 | **1362.6113** | 681.8093 |
| H | 2358.1931 | 1179.6002 | **1215.5429** | **608.2751** |
| Q | 2486.2516 | 1243.6295 | **1078.4840** | 539.7456 |
| S | 2573.2837 | 1287.1455 | **950.4254** | 475.7164 |
| Y | 2736.3470 | 1368.6771 | **863.3934** | **432.2003** |
| F | 2883.4154 | 1442.2113 | **700.3301** | 350.6687 |
| N[Dea] | 2998.4424 | 1499.7248 | **553.2617** | **277.1345** |
| G | 3055.4638 | 1528.2355 | **438.2347** | 219.6210 |
| F | 3202.5322 | 1601.7698 | **381.2132** | 191.1103 |
| S | 3289.5643 | 1645.2858 | **234.1448** | 117.5761 |
| K | 3417.6592 | 1709.3333 | **147.1128** | 74.0600 |

*Peptide sequence 14:* TFLAGQDNM[Oxi]LSNINTVATR

| Residue | b | b+2 | y | y+2 |
| --- | --- | --- | --- | --- |
| T | 102.0550 | 51.5311 | 2082.0284 | 1041.5179 |
| F | **249.1234** | 125.0653 | 1980.9808 | 990.9940 |
| L | **362.2074** | 181.6074 | 1833.9123 | 917.4598 |
| A | **433.2445** | 217.1259 | 1720.8283 | 860.9178 |
| G | 490.2660 | 245.6366 | 1649.7912 | **825.3992** |
| Q | **618.3246** | 309.6659 | 1592.7697 | 796.8885 |
| D | **733.3515** | 367.1794 | 1464.7111 | 732.8592 |
| N | **847.3945** | 424.2009 | 1349.6842 | 675.3457 |
| M[Oxi] | **994.4299** | **497.7186** | 1235.6412 | **618.3243** |
| L | **1107.5139** | **554.2606** | 1088.6058 | 544.8066 |
| S | **1194.5459** | 597.7766 | **975.5218** | 488.2645 |
| N | 1308.5889 | 654.7981 | **888.4898** | 444.7485 |
| I | 1421.6729 | **711.3401** | **774.4468** | 387.7271 |
| N | 1535.7159 | 768.3616 | **661.3628** | 331.1850 |
| T | 1636.7635 | 818.8854 | **547.3198** | 274.1636 |
| V | 1735.8320 | 868.4196 | **446.2722** | 223.6397 |
| A | 1806.8691 | 903.9382 | **347.2037** | 174.1055 |
| T | 1907.9168 | 954.4620 | **276.1666** | 138.5870 |
| R | 2064.0179 | 1032.5126 | **175.1190** | 88.0631 |
