# Supplementary material for: Shotgun proteomics of Brassica rapa seed proteins identifies vicilin as a major seed storage protein in the mature seed
Source: PLoS One. 2021 Jul 9;16(7):e0253384. doi: 10.1371/journal.pone.0253384 (PMC8270179; doi:10.1371/journal.pone.0253384)
Supplement: S2 Fig — (DOCX) [file pone.0253384.s002.docx]

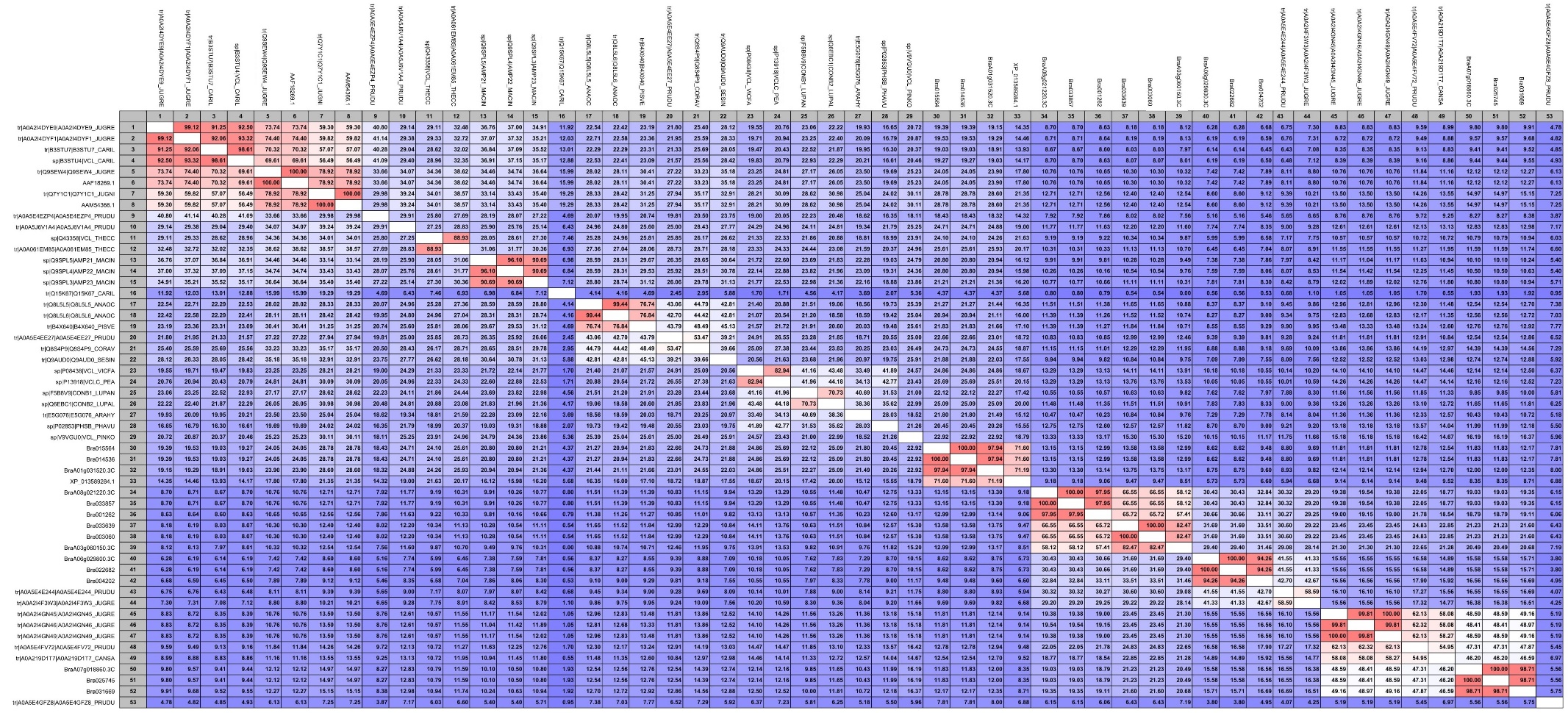


Employing the 48 full length 7S globulin-like vicilin sequences aligned in Supplementary Table S1, and S5 identified vicilin protein sequences in Supplementary Table S5, a pairwise analysis was undertaken using the CLC Main Workbench software (CLCbio, Katrinebjerg, AarhusN, Denmark) to obtain the number of conserved residues, *upper half* and percent identity, *bottom half*, between the proteins. Upper comparison gradient in the pairwise comparison among the sequences referred the distance between two sequences, and lower comparison gradient indicated percentage identity between two sequences. The software guided colour gradient will be applied here. The shades of red and blue colour represent, respectively, the maximal and minimal number of identical residues, or percent of identity, between the sequences.
