# Supplementary material for: Shotgun proteomics of Brassica rapa seed proteins identifies vicilin as a major seed storage protein in the mature seed
Source: PLoS One. 2021 Jul 9;16(7):e0253384. doi: 10.1371/journal.pone.0253384 (PMC8270179; doi:10.1371/journal.pone.0253384)
Supplement: S3 Fig — (DOCX) [file pone.0253384.s003.docx]

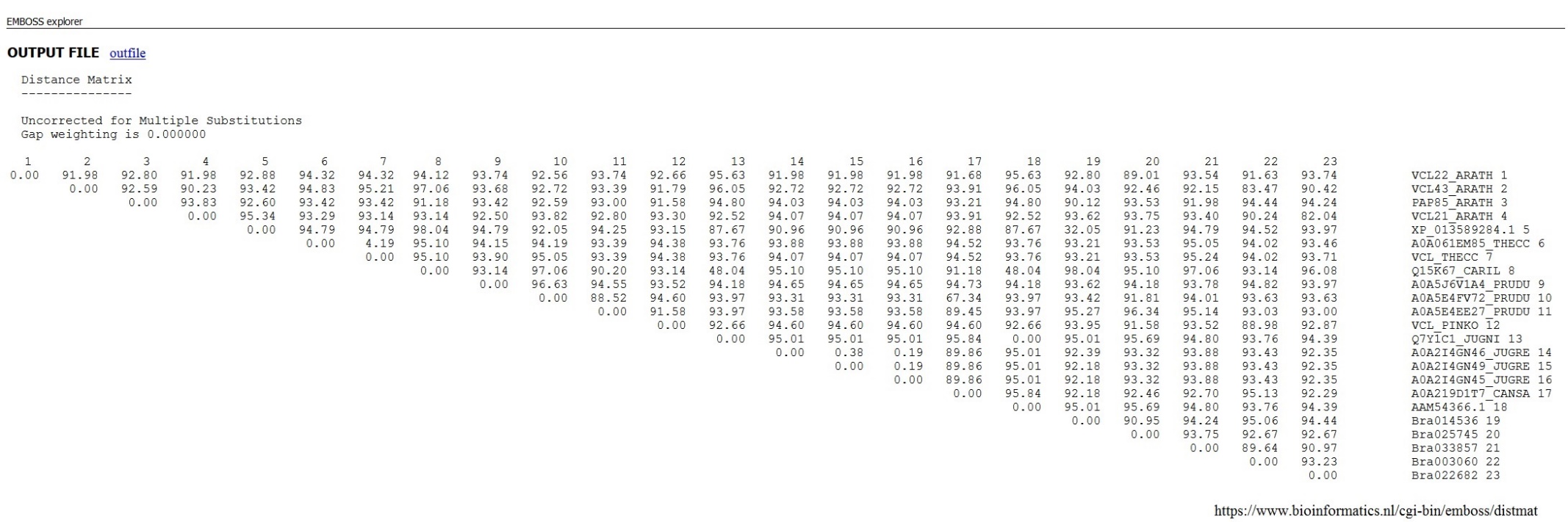


Employing the 18 full length 7S globulin-like vicilin sequences aligned in Supplementary Table S1, and S5 identified vicilin protein sequences in Supplementary Table S5, a Distance matrix was created using the distmat server (https://www.bioinformatics.nl/cgi-bin/emboss/distmat, 3, 08, 2020).
