# Supplementary figures and images for: Shotgun proteomics of Brassica rapa seed proteins identifies vicilin as a major seed storage protein in the mature seed
Source: PLoS One. 2021 Jul 9;16(7):e0253384. doi: 10.1371/journal.pone.0253384 (PMC8270179; doi:10.1371/journal.pone.0253384)

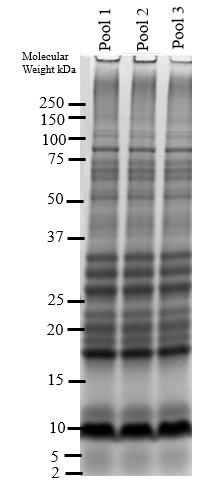

Supplement: S4 Fig — (DOCX) [file pone.0253384.s004.docx]
