# Supplementary material for: Shotgun proteomics of Brassica rapa seed proteins identifies vicilin as a major seed storage protein in the mature seed
Source: PLoS One. 2021 Jul 9;16(7):e0253384. doi: 10.1371/journal.pone.0253384 (PMC8270179; doi:10.1371/journal.pone.0253384)
Supplement: S1 Raw image — (PPTX) [file pone.0253384.s005.pptx]

## Slide 1
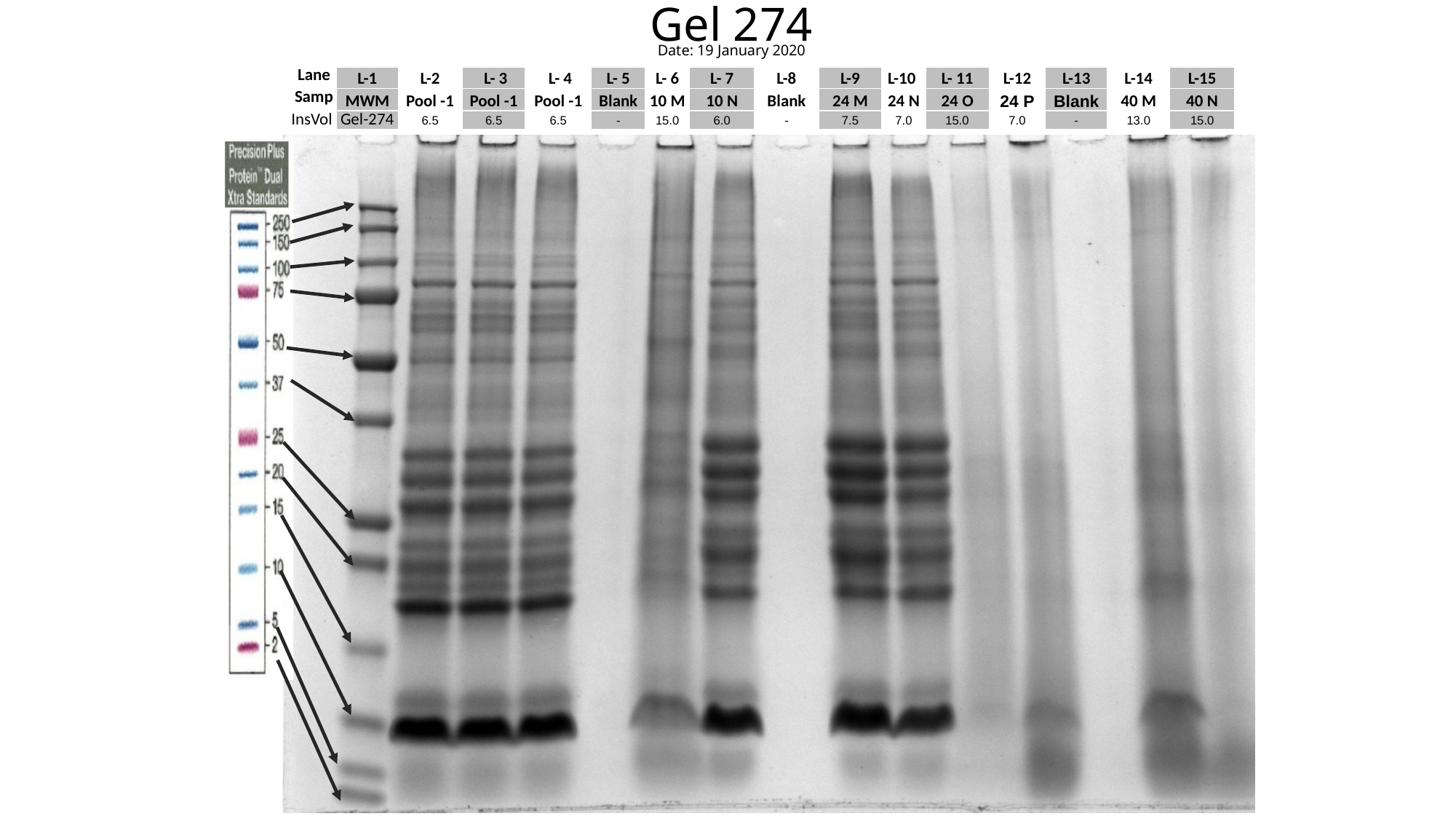

Gel 274
Date: 19 January 2020
| Lane | L-1 | L-2 | L- 3 | L- 4 | L- 5 | L- 6 | L- 7 | L-8 | L-9 | L-10 | L- 11 | L-12 | L-13 | L-14 | L-15 |
| --- | --- | --- | --- | --- | --- | --- | --- | --- | --- | --- | --- | --- | --- | --- | --- |
| Samp | MWM | Pool -1 | Pool -1 | Pool -1 | Blank | 10 M | 10 N | Blank | 24 M | 24 N | 24 O | 24 P | Blank | 40 M | 40 N |
| InsVol | Gel-274 | 6.5 | 6.5 | 6.5 | - | 15.0 | 6.0 | - | 7.5 | 7.0 | 15.0 | 7.0 | - | 13.0 | 15.0 |
