# Supplementary material for: Shotgun proteomics of Brassica rapa seed proteins identifies vicilin as a major seed storage protein in the mature seed
Source: PLoS One. 2021 Jul 9;16(7):e0253384. doi: 10.1371/journal.pone.0253384 (PMC8270179; doi:10.1371/journal.pone.0253384)
Supplement: S1 File — (DOCX) [file pone.0253384.s006.docx]

**S1 Table.** List of 7S globulin like vicilin sequences.

| Sl | Protein name | Gene name | Length | Sequences (5'-3') in FASTA format. OS= Organism species |
| --- | --- | --- | --- | --- |
|  | *Brassica rapa* Chiifu-401 | | |  |
| 1 | *B. rapa* Chiifu-401 Candidate-1 |  |  | >BraA07g018860.3C B.rapa.Chiifu.Candidate-1  MEKNKRIFTFLLVIMFFHGVMMMRSIGYEGEEEQGGGGRERGGFMMKESRQVIKSEGGEMRVVISPRGRIIEKPMHIGFLTMEPKTLFVPQYLDSNLLIF  IREATLGVICKDEFGEKRLKGGDIYWIPAGSAFYLLNTGRGQRLHVICSIDPSQSLGFETFQPFYIGGGPSSVLAGFDPDTITSALNVSRPEVQQLMTSQ  VRGPIVHITEHAPTMWTDFLGLRGEEKHKHLKKLLEMKQGTSQEQEFNPWWSWKNIVSSILDVTGEKNRGSGSSKCEDSYNIYDLKNDFENDYGWSKALD  YDDYEPLRYSGVGVYLVNLTAGSMMAPHMNPTATEYGIVLSGSGEIQVVLPNGTSAMNMRVSPGDVFWIPRYFAFCQIASRIAPFEFVGFTTSAYKNRPQ  FLVGSNSLLRSLNLTSLAMAFGVDEGTMKRFVEAQREAVILPTASAAPPHEGEPERFGSDHIFT |
| 2 | *B. rapa* Chiifu -401 Candidate-2 |  |  | >BraA08g021220.3C B.rapa.Chiifu.Candidate-2  MIRFTVLSFFVVFVLLFACNESSAKTAKYDKSDESVENDDLAAVPSCCGFSSPLLIKKDQWKPIFANKFGQISTVQIGDGCGGMGPYKIHSITLEPNALM  LPLLLHSDMVFFVDSGSGILNWVEAQATSTEIRLGDVYRLRPGTVFYLQSKPVDIFLGTKLKIYAIFSNSQECLHDPCFGAYSSVTDLLFGFDETILKSA  FGVPEEIIGLMRNRTQPPLIVHDMLTTPSEANTDTDTNTWPLQTRLLKLFSGDASADSVENKKVKKKKEKKEKKKKPKKATTFNVFESEPDFQSPNGQTI  TINRKDLKVLQGSMVGVSMVNLTQGSMMGPHWNPWACEISVVVRGSGMVRVLRNSISRSSSECKNMRFKVEKGDIFAVPRLHPMAQMSFLNDSLVFVGFT  TSAKNNEPQFLAGKNSALWSLDREVLAASFNVSSFMIAGLLEAQKEAAVLGCPACAEGELEKLKEDEEKKESPPQQPPQPFQPQPPEEKPQQPPQPFQPQ  PPQGEPQKPPQGEPQGPEKPFQPQPGQGEPQEPQASMETKMRDEERKREEEEAKKEEEERWKQEEKLWPTQPQWED |
| 3 | *B. rapa* Chiifu -401 Candidate-3 |  |  | >BraA03g060150.3C B.rapa.Chiifu.Candidate-3  MGPYKIHSITLEPNALLLPLLLHSDMVFFVESGSGILNWVEAEATSSEIRRGDVYRLRPGTVFYLQSKPIDIFLGTKLRVYAIFSNTEECLHDPCFGAYS  SITDLLFGFDEAILQSAFGVPEEIIGLMTNRTQPPLIVHDMLSTPGEANTYTWQLQPRLLKLFAGYVSAAEKKKKEKKTKKAKTFNVFESEPDFQSPNGR  TITINRKDLEVLSGSMVGVSMVNLTQASMMGPHWNPWACEISIVLKGSGMVRVLRSSISSTSSSSECKNMRFKVEEGDIFAVPRLHPMAQMSFINESLVF  IGFTTSARNNEPQFLAGQRSALRLLDQEVLAASLNVSSVMIEGLLGAQKDAVVLGCPYCAEGELEKLKVETEMKKRDDERKREEEEAKKEEEERRKREEE  EEEEKQWPPLPQQPPE |
| 4 | *B. rapa* Chiifu -401 Candidate-4 |  |  | >BraA06g029800.3C B.rapa.Chiifu.Candidate-4  MSKFTIIPLCLLTLFLCTNSFSDQNDGVPSSQSPLLVKRHQRTQLVATEFGEISAVHIGEEYTIQFITLEPNALLLPLLLHSDMVFFVHTGSGVLNWVDE  EKERTLELKRGDVFRLRYGTVFYLHCNLERDEVPEKLRVYAIFDVGKCLSDQCLGAYSSIRDLLWGFDEKTLRSAFAVPKDVFGRLRDAVKPPLITHAMP  KNRTQGSEEETWGSRLAKLFVRVEDSIVVDEKDMDALKGSSFGVYMVNLTKGSMMGPHWNPNACEISIVLQGEGMIRVVNHPSYQSKNESERFMVEDGDV  FVVPQFYPMAQLSFVNSSFMFMGFSTSAKTNHPQFLVGQNSVLKIFNRDVLATSFNMRYATVERLLGAQKDGLLLECVSCAEVELSRLMREIEERRRREE  EEIERRKREEEEAKRQEEERRRREEEEAERKKKAEEEARKREKEREREEEAAKRREEERRRREEEEAERKRKEEEEARKREEERKREEEAAKKREEERRK  REKEEEEARKREEAREREEEEAKKREEERRKREEEEAERKRRAEEEAREREEEEAKKREEEKEAARRREEEREKEEEMAKRREEERQRKEREDVERKKRE  EEEERKRREEEAMRREEERKREEEAAKRAEEERRKREEEAEHKKRPPPQGPQPPIHH |
| 5 | *B. rapa* Chiifu Candidate-5 |  |  | >BraA01g031520.3C B.rapa.Chiifu.Candidate-5 MAINKLTITLFLLISFAVFHCLAFRVEVQEFEPPRQEGQEGPGGGSGEGWDEEATKNPYHFGQWSFKNFFQSKDGFVKMLPKFTKRSSTLFRGIENYRFL  FQEMEPNTFLVPHHLDADYVFLVVQGKGVIGFVTDTANESFQITKGDVVRVPSSVTHFFANTNGTVPLRLAKIAVPANVPGHFQVFFPAHSGFHQSYFTG  FSKDVLTASFNIPEELLGRLIRGPQQQVGQGIIRRVSPEQIKELTEHEHATSPSNKHKDKKDKHKDKDRSTFGSPFNLLAQDAIYSNKFGRYHEAHPKRF  SQLQDLDIAVGWVNMTQGSLFLPQYNSETTFVTFVENGCARYEMASPYTFQAEQQQPWFGPGQEEEVEEEMSGQVHKIVSRVCKGEVFILPAGHPFAILS  QDENFVAVGFGIHASNSTRTFLAGQDNMLSNINTVATRLSFGLGSKLAEKLFTSQNYSHFAPTTPSHQFPEKPKPSFQSIFNLAGF |
|  | *Brassica rapa* R-o-18 | | |  |
| 6 | *B. rapa* R-o-18 Candidate-1 |  |  | >Bra031669 B.rapa.Ro18.Candidate-1 MEKNKRIFTFLLVIVFFHGVMMMRSIGYEGEEEQGGGGRERGGFMMKESRQVIKSEGGEMRVVISPRGRIIEKPMHIGFLTMEPKTLFVPQYLDSNLLIFIRQGEATLGVICKDEFGEKRLKGGDIYWIPAGSAFYLLNTGRGQRLHVICSIDPSQSLGFETFQPFYIGGGPSSVLAGFDPDTITSALNVSRPEVQQLMTSQVRGPIVHITEHAPTMWTDFLGLRGEEKHKHLKKLLELKQGTSQEQEDNPWWSWKNIVSSILDVTGEKNRGSGSSKCEDSYNIYDRKNDFENDYGWSKALDYDDYEPLRYSGVGVYLVNLTAGSMMAPHMNPTATEYGIVLSGSGEIQVVLPNGTSAMNMRVSPGDVFWIPRYFAFCQIASRIAPFEFVGFTTSAYKNRPQFLVGSNSLLRSLNLTSLAMAFGVDEGTMKRFVEAQREAVILPTASAAPPHEGEPERFGSDHIFT |
| 7 | *B. rapa* R-o-18 Candidate-2 |  |  | >Bra001262 B.rapa.Ro18.Candidate-2 MIRFTVLSFFVVFVLLFACNESSAKTAKYDKSDESVENDDLAAVPSCCGFSSPLLIKKDQWKPIFANKFGQISTVQIGDGCGGMGPYKIHSITLEPNALMLPLLLHSDMVFFVDSGSGILNWVEAQATSTEIRLGDVYRLRPGTVFYLQSKPVDIFLGTKLKIYAIFSNSQECLHDPCFGAYSSVTDLLFGFDETILKSAFGVPEEIIGLMRNRTQPPLIVHDMLTTPGEANTDTDTNTWPLQTRLLKLFSGDASADSVENKKVKKEKKEKKEKKKKPKKATTFNVFESEPDFQSPNGQTITINRKDLKVLQGSMVGVSMVNLTQGSMMGPHWNPWACEISVVVRGSGMVRVLRNSISRSSSECKNMRFKVEKGDIFAVPRLHPMAQMSFLNDSLVFVGFTTSAKNNEPQFLAGKNSALWSLDREVLAASFNVSSFMIAGLLEAQKEAAVLGCPACAEGELEKLKEDEEKKESPPQQPPQPFQPQPPGEKPQQPPQPFQPQPPQGEPQKPPQGEPQKPPQGEPEGPQKPFQPQPGQGEPQEPQASMETKMRDEERKREEEEAKKEEEERWKQEEKLWPTQPQWED |
| 8 | *B. rapa* R-o-18 Candidate-3 |  |  | >Bra033639 B.rapa.Ro18.Candidate-3 MTKFTVLPLFVLLFLVLLCTKSWAKSEEFDESSDEENDVAAVPSCCGFSSPLLIKKDQWKPIFGTQFGQISTVQIGEGCGGMGPYKIHSITLEPNALLLPLLLHSDMVFFVESGSGILNWVEAEPTSSEIRRGDVYRLRPGTVFYLQSKPIDIFLGTKLRVYAIFSNTEECLHDPCFGAYSSITDLLFGFDEAILQSAFGVPEEIIGLMTNRTQPPLIVHDMLSTPGEANTYTWQLQVQPRLLKLFAGYVSAAEKKKKEKKTKKAKTFNVFESEPDFQSPSGRTITINRKDLEVLSGSMVGVSMVNLTQASMMGPHWNPWACEISIVLKGSGMVRVLRSSISSTSSSSSSSECKNMRFKVEEGDIFAVPRLHPMAQMSFINESLVFIGFTTSARNNEPQFLAGQRSALRLLDQEVLAASLNVSSVMIEGLLGAQKDAVVLGCPYCAEGELEKLKVETEMKKRDDERKREEEEAKKEEEERRKREEEEEEEKQWPPLPQQPPE |
| 9 | *B. rapa* R-o-18 Candidate-4 |  |  | >Bra004202 B.rapa.Ro18.Candidate-4 MSKFTIIPLCLLTLFLCTNSFSDQNDGVPSSQSPLLVKRHQRTQLVATEFGEISAVHIGEEYTIQFITLEPNALLLPLLLHSDMVFFVHTGSGVLNWVDEEKERTLELKRGDVFRLRYGTVFYLHCNLERDEVPEKLRVYAIFDVGKCLSDQCLGAYSSIRDLLWGFDEKTLRSAFAVPKDVFGRLRDAVKPPLITHAMPKNRTQGSEEETWGSRLAKLFVRVEDVTDHLEMKPVVNKKKKKKKKKSSAYNVFESDPDFENDNGQSIVVDEKDMDALKGSSFGVYMVNLTKGSMMGPHWNPNACEISIVLQGEGMIRVVNHPSYQSKNESERFMVEDGDVFVVPQFYPMAQLSFVNSSFMFMGFSTSAKTNHPQFLVGQNSVLKIFNRDVLATSFNMRYATVERLLGAQKDGLLLECVSCAEVELSRLMREIEERRRREEEEIERRKREEEEAKRQEEERRRREEEEAERKKKAEEEARKREKEREREEEAAKRREEERRRREEEEAERKRKEEEEARKREEERKREEEAAKKREEERRKREKEEEEARKREEAREREEEEAKKREEERRKREEEEAERKRRAEEEAREREEEEAKKREEEKEAARRREEEREKEEEMAKRREEERQRKEREDVERKKREEEEERKRREEEAMRREEERKREEEAAKRAEEERRKREEEAEHKKRPPPQGPQPPIHH |
| 10 | *B. rapa* R-o-18 Candidate-5 |  |  | >Bra015564 B.rapa.Ro18.Candidate-5 MAINKLTITLFLLISLAVFHCLAFRVEVQEFEPPRQEGQEGPGGGSGEGWDEEATKNPYHFGQWSFKNFFQSKDGFVKMLPKFTKRSSTLFRGIENYRFLFQEMQPNTFLVPHHLDADYVFLVVQGKGVIGFVTDTANESFQITKGDVVRVPSSVTHFFANTNGTVPLRLAKIAVPANVPGHFQVFFPAHSGFHQSYFNGFSKDVLTASFNIPEELLGRLIRGPQQEVGQGIIRRVSPEQIKELTEHEHATSPSNKHKDKKDKHKDKDRSTFGSPFNLLTQDAIYSNNFGRYHEAHPKRFSQLQDLDIAVGWVNMTQGSLFLPQYNSETTFVTFVENGCARYEMASPYTFQGEQQQPWFGPGQEEEVEEEMSGQVHKIVSRVCKGEVFILPAGHPFAILSQDENFVAVGFGIHASNSTRTFLAGQDNMLSNINTVATRLSFGLGSKMAEKLFTSQNYSHFAPTTPSHQFPEKPKPSFQSVFNLVGF |
|  | *Brassica oleracea* | | |  |
| 11 | PREDICTED: vicilin [*Brassica oleracea* var. oleracea] | N/A |  | >XP_013589284.1 PREDICTED: vicilin [Brassica oleracea var. oleracea]  MAINKLTITLFLLISLAVFHCLAFRVEVQEFEPPQQEGQEGHGGGSGEGWDEEATKNPYHFGRWSFKNFF  QSQEGFVKMLPKFTKRSSTLFRGIENYRFLFQEMQPNTFLVPHHLDADYVFLVVQGKGVIGFVTDTENES  FQITKGDVVRVPSSVTHFFANTNGTVPLRLAKIAVPANVPGHFQVFFPSHSGFHQSYFTGFSKDVLTASF  NVPEELLGRLIRRPQQQVGQGIIRRVSPDQIKELTEHAISPSNKHKNKKDKHKDKDRSTFGSPFNLLTQD  AIYSNDFGRYHEAHPKKFSQLQDLDIAVGWVNMTQGSLFLPQYNSETTFVTFVENGCARYEMASPCTFQG  EQQQPWFGPGQEEEV |
|  | Pinus koraiensis (Korean pine) | | |  |
| 12 | Vicilin Pin k 2.0101 | N/A | 463 | >sp\|V9VGU0\|VCL_PINKO Vicilin Pin k 2.0101 OS=Pinus koraiensis OX=88728 PE=1 SV=1  MAFVSLLTILLAISSSSVALTEPVASMADQGVFPEQHGRGHHGVFPEEHGRGHHRGGREE  EREENPYVFHSDRFRIRASSEAGEIRALPNFGEVSELLEGISRYRVTCIEMKPNTVMLPH  YIDAKWILYVTGGRGYIAYVQQNELVKRKLEEGDVFGVPSGHTFYLVNNDDHNSLRIASL  LRTESTMRGEYEPFYVAGGRNPETVYSAFSDDVLEAAFNTDVQKLEHIFGAHRRGVIFYA  NEEQIREMMRRGGFSAESTSASEQPKPFNLRNQKPDFENDNGRFTRAGPKDNPFLDSVDV  TVGFGVLNPGTMTAPSHNTKATSIAIVMEGEGRIEMACPHLGQEHGWSSPRERGHQDINY  ERVRARLRTGTVYVVPAGHPITEIASTNGRLEILWFDINTSGNEREFLAGKNNVLQTLEK  EVRHLSFNIPRGEEIEEVLQAQKDQVILRGPQRQRRDEPRSSS |
|  | *Prunus dulcis* (Almond) (*Amygdalus dulcis*) | | |  |
| 13 | Vicilin | N/A | 547 | >tr\|A0A5J6V1A4\|A0A5J6V1A4_PRUDU Vicilin OS=Prunus dulcis OX=3755 PE=4 SV=1  MAIKITIKASYKLPFFFFFLSTLFLASSSVTPLINALSDYHNQKCQQSICRGVGGRHSLL  RSKDHPQDAREEYFYCSQSCGTSEDPEQCETECRERFDEQLKKEAEEQQKGQEEEEEEGP  TFNPNPYYFPKFGLRPRFLAEEGAYFVLGSFARLSHLLRGRIQNYRAALLQTTPGTFVLP  YHLDAESIFVVWNGRGTLTLVMKDTKQSFKIENGDVIRVPAGATTYLINNHTTENLSLVQ  LFQPVNTPDLFEEFFPAGYKDPEPGSDYSFLHGTESYYSVFSNDLLEAAFDVPREQLEKA  FGQQKREGMIIRASKEQLDALSKQAYPWWRKLVPWSMGSDLNFNLLSQRPLHSNNYGKFY  EASPQEFKQLQDMNVSVAMLDINPEAMMVPHYNSKATYLMMVVDGMGYFEMACPKFTIPA  SEEEMEYQEEQADQQSGVFSKVSGKLSLGDVFVIPAGHPVSIVAQNNNNNNNNNGNQNQK  LRIVGFGINAGNNIRNFLAGQEGNIMKQMEREATQLTFGQEMEQVLTSQKQSYFVPASRR  GSSTEKA |
| 14 | PREDICTED: vicilin | ALMOND_2B017533 | 534 | >tr\|A0A5E4FV72\|A0A5E4FV72_PRUDU PREDICTED: vicilin OS=Prunus dulcis OX=3755 GN=ALMOND_2B017533 PE=4 SV=1  MGNRVLGFGVLLLVMWYGVSVAVGYTGDPDEDWGRKKEEREGRPGSGDMRRPEYEKPESE  KEDWYLLPHSRQVVKTEAGEMSVVMRVGGRVVDKPMHIGFITMEPKSLFIPQYLDSNLVL  FVRRGEAKVGLIYRDELGERRLKSGDVYRIPAGSPFYLVNTGEGQRLHIICSLDTSESLG  LGSMQSFFIGGGSNPQSVLAGFDHDILTNAFNVSSSELMEVLTSQQKGPIVYLSDSHSPN  LWAKFLQLKEQDRLQEMKKMVDFQQEPDHHQDQTQTWSWRKLLNSVFGAGSDDNKKRAED  YDKGKGKAPHSYNLYDRKPDFRNNYGWSMELDESDYAPLKDSGVGVYLVNLTAGAMMAPH  VNPTATEYGIVLRGSGTIQIVFPNGTSAMNTNVQDGDVFWVPRYFPFCQIASRSGALEFF  GFTTSARKNRPQFLAGASSVLQTIRGPELAAAFGVSEDRLRKFIDAQREAVILPSAQAAP  PYKEDRQQPREEDEKKQPREGDERKQPKGDDRGTFERVPEVIKSFGTDMFMGFD |
| 15 | PREDICTED: vicilin | ALMOND_2B023004 | 514 | >tr\|A0A5E4EE27\|A0A5E4EE27_PRUDU PREDICTED: vicilin OS=Prunus dulcis OX=3755 GN=ALMOND_2B023004 PE=4 SV=1  MALKSKLLLVALLLSVLFLSVYVASATQDPELKQCRHQCEHQQGFDSKQREQCEQGCDKY  IKQKREEEKHRRKSEGGGSFYPISETGRAQEEEEEFQGRQQQDQNPYFFEDEHFETRVQT  EEGRFQLLQKFTERSDLLRAIENYRIGFLVTKPHAFVAPSHFDADTVLFVFQGRPAVTIV  RGEKRETHNLEHGDLFRIPAGTPVYMVNRDENEKLFIVNFMKPVSVPGEYEAFYAAGGEN  PESFFKAFSPQVLQAALKTEINKLERLFGQQRQGSITRASKEPIKKLSQQHGQGGSEGFL  PFHGGQSSSDAFNLFSKHPSQANKFGRLFEADFNDFKQLQDLDLLVSFANITQGAMVGPY  FNSRATKISFVLDGEGYFEMACPHVSSTGRQEPQPQPEQQPQPQQRRKSSPRYQKISGNL  RRGAVFVAPAGHPMTAIASRNSNLQIICFEVNAHDNIRVPLVGKKNVVSQFDREAKELAF  NVPAREVDRIFNNQDDEFFFEGPNEQPEHGRAYA |
| 16 | PREDICTED: vicilin | ALMOND_2B021144 | 811 | >tr\|A0A5E4EZP4\|A0A5E4EZP4_PRUDU PREDICTED: vicilin OS=Prunus dulcis OX=3755 GN=ALMOND_2B021144 PE=4 SV=1  MVIHIKPRLPLFLFLSLLFLAFSVSFTLGQDREQHCQKRCQDFKRPIIHSECIYQCRMWG  PWSLPPAESSNHHKQQQQHEQEQQCKQRCQPQHGHRQQQQCQQECYEQIRQQREREEMQQ  MCQQSCEMQGSGGQQQQRQQCQRQCEQQVEQLKQCQLRCQMQGQGQQQEQCQRTCRQELE  DQQRRQQQEQGPGGGDNKLEDETLNGEPRRRFEQCMQGCERQQQGQGEQEQCPRQCREQF  DKEKRQYQQCKQSCEKSSQHDDEKRQCKQQCKQQISLQQGGGNQEEDDDALNGSGYGGAK  ERFEQCKLSCQSQQGQGQQEQCPKQCKQQFEHEKHQYKQCKRGCENQARDDVQKEQCKQQ  CTQQMSQQYGQQQEGSTGGGGLNQQQEEEGQMGSQGQSQSQSQSNNPFYFPSHRFQPRFQ  SNEGGLYVLERFTQRQSEVLRGIKNYRLAIFEARPNTFVLPHHCDAEAIYVVLSGQCTCT  LLMQDRKESFNMEHGDVIRVPAGATTYLVNNNSDKTLRIAKLLQPVNNPGRFEEFFPAGS  RNSESYFSVFSNDILESAFNTPREQLEHGFKQGQQQQGQGIVMRAPREQLQALSQAASSR  RRGDRQSQGPFNLRQQRPVHSNNYGQFFEARPEEFNQLQDMDASVSCIEINQQAMMVPHF  NSKATHLIMVVEGKGLVEMACPYLANQSQEIMGQQEQQGEQSGRYMKVTAQLSPGDVFVI  PAGHPVALVAQNQNLRILGFGLYAQNNKRNFLAGQEDNIIMHMDREARQLAFGPEMEQIF  SKQQQSYFVPTQQGRSRNQHLSSILEFAGVI |
| 17 | PREDICTED: vicilin seed | ALMOND_2B032650 | 689 | >tr\|A0A5E4E244\|A0A5E4E244_PRUDU PREDICTED: vicilin OS=Prunus dulcis OX=3755 GN=ALMOND_2B032650 PE=4 SV=1  MLNNKSSNQQPFPFTFFILLFIGLSLPAAKAILRNEEQYGSGASAAAAGSLVKRDQRRAL  VVTEYGEISSIDISDGHRGPYHIQFITLEPNSLFLPVLLHADMVFYVHTGSGRLSWGDED  DIKRVAIKRGDLFRLRPGSIFFVQSDLQAERQKLRIYAIFATNTDDDLYDPAIGAYSSVR  DLVRAFDPKVLRSAFKVSDEVIESIINGTDQSGIVHAVPTKKETFWDLEARFLKTFLTGK  DGSAFNKNKKKTKTYNIFDEGPDFKNCNGWSLTVNKKNSQLLKGSNIGLFMVNLTKGSMM  GPHWNPRATEIAIVLHGQGMVRVVCSSTAAKKSECKSMRLRVHEGDVFAVPRFHPMAQMS  FNNDSLVFMGFSTTTRRNYPQFLAGKYSVLQSLDKQVLAASFNVSNTTVDQLLAAQADSV  IIDCTSCAEEEERRMMEEIEKEREEEEAKKWEEEEARKRAEEKEQRKREEEEAARKREEE  EAAARKREEEAAAARQREEEAAAARQREEEEAAARQREKEQEAERQREEEQAAKQREEEE  AKKREGEGRQREEERRQEEETERERQQQKEWERRGQEAQTEQEEARRQQEEREGKERPEE  SAREEEGRQREEERRQEEETERERQQQKEWERREQEAQTEQEEARRQQEEREGKERPEES  AGRQPEEREGGGRGIDVEEGRRYLRVLKV |
| 18 | PREDICTED: vicilin seed | ALMOND_2B028594 | 332 | >tr\|A0A5E4GFZ8\|A0A5E4GFZ8_PRUDU PREDICTED: vicilin seed OS=Prunus dulcis OX=3755 GN=ALMOND_2B028594 PE=4 SV=1  MGKDRNGSVQERRSKSSEKEDENERKSEKAIEHSDSQSEEDRKRRKSRRKARESSQSSDE  KRVRSDRRKRGSRRRYSSESDSDSDSKSESEESGSDSASESESESESDSDRRRKRKRRKE  KDDDRERKRRRREKEKKRRREEERKKKKEKRKKKRKEKKEKGNKGAVTNSWGKYGIIRET  DMWNKRPEFTAWLAEVKQVNLEHLPNWEEKQMFKQFMEDHNTATFPSKKYYSLDAYYRHK  LEKEIKKGYKKVGQTERTVFNDEEQRRQEMMRVREKQKEEEVEALKRSMQSGLAQAMKEQ  AQLREEMAYQYKIGNFEAAAAIQRRLDPDAAV |
|  | *Juglans regia* (English walnut) | | |  |
| 19 | Vicilin-like protein | N/A | 593 | >tr\|Q9SEW4\|Q9SEW4_JUGRE Vicilin-like protein (Fragment) OS=Juglans regia OX=51240 PE=2 SV=1  RGRDDDDEENPRDPREQYRQCQEYCRRQGQGQRQQQQCQIRCEERLEEDQRSQEERERRR  GRDVDDQNPRDPEQRYEQCQQQCERQRRGQEQTLCRRRCEQRRQQEERERQRGRDRQDPQ  QQYHRCQRRCQIQEQSPERQRQCQQRCERQYKEQQGRERGPEASPRRESRGREEEQQRHN  PYYFHSQSIRSRHESEEGEVKYLERFTERTELLRGIENYRVVILDANPNTSMLPHHKDAE  SVAVVTRGRATLTLVSQETRESFNLECGDVIRVPAGATVYVINQDSNERLEMVKLLQPVN  NPGQFREYYAAGAKSPDQSYLRVFSNDILVAALNTPRDRLERFFDQQEQREGVIIRASQE  KLRALSQHAMSAGQRPWGRRSSGGPISLKSESPSYSNQFGQFFEACPEEHRQLQEMDVLV  NYAEIKRGAMMVPHYNSKATVVVYVVEGTGRYEMACPHVSSQSYEGQGRREQEEEESTGR  FQKVTARLARGDIFVIPAGHPIAITASQNENLRLLGFDINGENNQRDFLAGQNNIINQLE  REAKELSFNMPREEIEEIFESQMESYFVPTERQSRRGQGRDHPLASILDFAFF |
| 20 | vicilin-like seed storage protein At2g18540 | LOC108995264 | 681 | >tr\|A0A2I4F3W3\|A0A2I4F3W3_JUGRE vicilin-like seed storage protein At2g18540 OS=Juglans regia OX=51240 GN=LOC108995264 PE=4 SV=1  MIISSQQLSSLDHLQEKTMRNIALISPFSFLFLLLILCQYSFFNLHAAEAFNVSDVGPLV  KKDERRSLVATEYGEVTATDISDGIRGLYHLQFITLEPNSLFLPVLLHADMVFYVHTGSG  RLRWAEDDDNQMKRVNLRRGDIYMLQAGSIFYVKSSLEPERKKLRIHAMFSNTPDDSYDP  LIGAYSSIRNLVRGFDKRVLRAAFKVPEDVIDDITQGTPAPAIVHAVQTKEENFWELEAR  FLKVFLGGKGGMTFNKKKKIRAYNILDADPDFENCNGWSLTVTRRNANLLKGSNIGLFMV  NLTKGSMMGPHWNPMATEIAIVLQGQGMVRVICSSSAKKPECKNMRLMVKEGDVFAVPRF  HPMAQMSFNNDSFVFMGFSTTRRRNHPQFLAGKSSVLQTLDKQALAVSFNVTNSTIDQLL  SGQADSFILECTSCAEEEKIILEEEIEREREEEKARKREREEREREEREREEEEARKREE  EARKREEREEKEREEREEKEARKREEEARKRQEEEEARKREEEEERREKKEAESKQEEER  RRREEEKEQRQEEEGRREREERESERREREREEQEQEERQKEEEKRRQEEAEWEEEEARR  EEERRRRRQEEEEEARREREREKERRRWEEARREREIQEPAGRQPEEREEQRRQNEKATS  EEQEEEDSREGGRRAPRMRWI |
| 21 | vicilin-like antimicrobial peptides 2-2 isoform X1 | LOC108984611 | 796 | >tr\|A0A2I4DYE9\|A0A2I4DYE9_JUGRE vicilin-like antimicrobial peptides 2-2 isoform X1 OS=Juglans regia OX=51240 GN=LOC108984611 PE=4 SV=1  MVIKAKIPLFLFLSALFLALVCSSLALETEDLSNELNPHHDPESHRREFQQCQERCQREE  RGQRQAQQCQRRCEEQLREREREREREEVVNPRDPRQQYEQCRETCEKQDPRQQPQCERR  CERQFQEQEERERRERGRGKGDDDQENPRDPREQYRQCQEHCRRQGKGQRQQQQCQSRCE  ERFEEEQRRQEERERRRGRDDDDEENPRDPREQYRQCQEYCRRQGQGQRQQQQCQSRCEE  RLEEEQRSQEERERRRGRDVDDQNPRDPEQRYEQCQQQCERQRRGQEQTLCRRRCEQRRQ  QEERERQRGRDRQDPQQQYHRCQRRCQIQEQSPERQRQCQQRCERQYKEQQGRERGPEAS  PRRESRGREEEQQRHNPYYFHSQSIRSRHESEEGEVKYLERFTERTELLRGIENYRVVIL  DANPNTFMLPHHKDAESVAVVTRGRATLTLVSQETRESFNLECGDVIRVPAGATVYVINQ  DSNERLEMVKLLQPVNNPGQFRFEFVVLQEYYAAGAKSPDQSYLRVFSNDILVAALNTPR  DRLERFFDQQEQREGVIIRASQEKLRALSQHAMSAGQRPWGRRSSGGPISLKSERPSYSN  QFGQFFEACPEEHRQLQEMDVLVNYAEIKRGAMMVPHYNSKATVVVYVVEGTGRYEMACP  HVSSQSYEGQGRREQEEEESTGRFQKVTARLARGDIFVIPAGHPIAITASQNENLRLLGF  GINGENNQRNFLAGQNNIINQLEREAKELSFNMPREEIEEIFESQMESYFVPTERQSRRG  QGRDHPLASILDFAFF |
| 22 | vicilin-like seed storage protein At2g28490 isoform X1 | LOC109009328 | 523 | >tr\|A0A2I4GN49\|A0A2I4GN49_JUGRE vicilin-like seed storage protein At2g28490 isoform X1 OS=Juglans regia OX=51240 GN=LOC109009328 PE=4 SV=1  MKAKMGNRATLLMLVLVLCYGMAKAMAVSFAEEEDFGREKEERKEREQRRSEREEGEEET  DDRFLLQDSKSVMRTEAGEMRVIKSLGGKIWDRPLHIGFITMEPQTLFIPQYLDSSLMIF  IRRGEAKIGLIYKDELGERRLKTGDLYRIPAGSAFYLVNTAEGQRLHIICSIDPSESLGI  GTFQSFFIGGGKYPTSVLAGFERETLSNAFNVSFSEVQDILSRQREGPIVYVPESHSPSV  WTKFLQMKERDRLQHLKKLVDFHEPRDDXEEEEEEQQQQITWSWRKLLNSVLGKEPSKRG  DKRTRRTPDSYNLYKRRPDFRNNYGWSVALDESDYTPLKHSGISVFLVNLTAGSMMAPHV  NPTATEYGIVLSGSGTIQIVYPNGTSAMNAKISEGDVFWVPKYFPFCQIASRTGPLEFFG  FTTSARTNRPQFLVGASSILRTMLGPELAAAFGVSEDKLQRFVYAQEEAVILPSPKAAPP  NEKMKSKKTGEKEKEKEKEKERMFEEVPKVIKSFGTEMIMGFD |
| 23 | vicilin-like antimicrobial peptides 2-3 isoform X2 | LOC108984611 | 789 | Juglans regia (English walnut)  >tr\|A0A2I4DYF1\|A0A2I4DYF1_JUGRE vicilin-like antimicrobial peptides 2-3 isoform X2 OS=Juglans regia OX=51240 GN=LOC108984611 PE=4 SV=1  MVIKAKIPLFLFLSALFLALVCSSLALETEDLSNELNPHHDPESHRREFQQCQERCQREE  RGQRQAQQCQRRCEEQLREREREREREEVVNPRDPRQQYEQCRETCEKQDPRQQPQCERR  CERQFQEQEERERRERGRGKGDDDQENPRDPREQYRQCQEHCRRQGKGQRQQQQCQSRCE  ERFEEEQRRQEERERRRGRDDDDEENPRDPREQYRQCQEYCRRQGQGQRQQQQCQSRCEE  RLEEEQRSQEERERRRGRDVDDQNPRDPEQRYEQCQQQCERQRRGQEQTLCRRRCEQRRQ  QEERERQRGRDRQDPQQQYHRCQRRCQIQEQSPERQRQCQQRCERQYKEQQGRERGPEAS  PRRESRGREEEQQRHNPYYFHSQSIRSRHESEEGEVKYLERFTERTELLRGIENYRVVIL  DANPNTFMLPHHKDAESVAVVTRGRATLTLVSQETRESFNLECGDVIRVPAGATVYVINQ  DSNERLEMVKLLQPVNNPGQFREYYAAGAKSPDQSYLRVFSNDILVAALNTPRDRLERFF  DQQEQREGVIIRASQEKLRALSQHAMSAGQRPWGRRSSGGPISLKSERPSYSNQFGQFFE  ACPEEHRQLQEMDVLVNYAEIKRGAMMVPHYNSKATVVVYVVEGTGRYEMACPHVSSQSY  EGQGRREQEEEESTGRFQKVTARLARGDIFVIPAGHPIAITASQNENLRLLGFGINGENN  QRNFLAGQNNIINQLEREAKELSFNMPREEIEEIFESQMESYFVPTERQSRRGQGRDHPL  ASILDFAFF |
| 24 | vicilin-like seed storage protein At2g28490 isoform X2 | LOC109009328 | 523 | >tr\|A0A2I4GN45\|A0A2I4GN45_JUGRE vicilin-like seed storage protein At2g28490 isoform X2 OS=Juglans regia OX=51240 GN=LOC109009328 PE=4 SV=1  MKAKMGNRATLLMLVLVLCYGMAKAMAVSFAEEEDFGREKEERKEREQRRSEREEGEEET  DDRFLLQDSKSVMRTEAGEMRVIKSLGGKIWDRPLHIGFITMEPQTLFIPQYLDSSLMIF  IRRGEAKIGLIYKDELGERRLKTGDLYRIPAGSAFYLVNTAEGQRLHIICSIDPSESLGI  GTFQSFFIGGGKYPTSVLAGFERETLSNAFNVSFSEVQDILSRQREGPIVYVPESHSPSV  WTKFLQMKERDRLQHLKKLVDFHEPRDDDEEEEEEQQQQITWSWRKLLNSVLGKEPSKRG  DKRTRRTPDSYNLYKRRPDFRNNYGWSVALDESDYTPLKHSGISVFLVNLTAGSMMAPHV  NPTATEYGIVLSGSGTIQIVYPNGTSAMNAKISEGDVFWVPKYFPFCQIASRTGPLEFFG  FTTSARTNRPQFLVGASSILRTMLGPELAAAFGVSEDKLQRFVYAQEEAVILPSPKAAPP  NEKMKSKKTGEKEKEKEKEKERMFEEVPKVIKSFGTEMIMGFD |
| 25 | vicilin-like seed storage protein At2g28490 isoform X3 | LOC109009328 | 523 | >tr\|A0A2I4GN46\|A0A2I4GN46_JUGRE vicilin-like seed storage protein At2g28490 isoform X3 OS=Juglans regia OX=51240 GN=LOC109009328 PE=4 SV=1  MKAKMGNRATLLMLVLVLCYGMAKAMAVSFAEEEDFGREKEERKEREQRRSEREEGEEET  DDRFLLQDSKSVMRTEAGEMRVIKSLGGKIWDRPLHIGFITMEPQTLFIPQYLDSSLMIF  IRRGEAKIGLIYKDELGERRLKTGDLYRIPAGSAFYLVNTAEGQRLHIICSIDPSESLGI  GTFQSFFIGGGKYPTSVLAGFERETLSNAFNVSFSEVQDILSRQREGPIVYVPESHSPSV  WTKFLQMKERDRLQHLKKLVDFHEPRDDDEEEEEEQQQQITWSWRKLLNSVLGKEPSKRG  DKRTRRTPDSYNLYKRRPDFRNNYGWSVALDESDYTPLKHSGISVFLVNLTAGSMMAPHV  NPTATEYGIVLRGSGTIQIVYPNGTSAMNAKISEGDVFWVPKYFPFCQIASRTGPLEFFG  FTTSARTNRPQFLVGASSILRTMLGPELAAAFGVSEDKLQRFVYAQEEAVILPSPKAAPP  NEKMKSKKTGEKEKEKEKEKERMFEEVPKVIKSFGTEMIMGFD |
| 26 | vicilin-like protein precursor, partial [Juglans regia] |  | 593 | >AAF18269.1 vicilin-like protein precursor, partial [Juglans regia]  RGRDDDDEENPRDPREQYRQCQEYCRRQGQGQRQQQQCQIRCEERLEEDQRSQEERERRRGRDVDDQNPR  DPEQRYEQCQQQCERQRRGQEQTLCRRRCEQRRQQEERERQRGRDRQDPQQQYHRCQRRCQIQEQSPERQ  RQCQQRCERQYKEQQGRERGPEASPRRESRGREEEQQRHNPYYFHSQSIRSRHESEEGEVKYLERFTERT  ELLRGIENYRVVILDANPNTSMLPHHKDAESVAVVTRGRATLTLVSQETRESFNLECGDVIRVPAGATVY  VINQDSNERLEMVKLLQPVNNPGQFREYYAAGAKSPDQSYLRVFSNDILVAALNTPRDRLERFFDQQEQR  EGVIIRASQEKLRALSQHAMSAGQRPWGRRSSGGPISLKSESPSYSNQFGQFFEACPEEHRQLQEMDVLV  NYAEIKRGAMMVPHYNSKATVVVYVVEGTGRYEMACPHVSSQSYEGQGRREQEEEESTGRFQKVTARLAR  GDIFVIPAGHPIAITASQNENLRLLGFDINGENNQRDFLAGQNNIINQLEREAKELSFNMPREEIEEIFE  SQMESYFVPTERQSRRGQGRDHPLASILDFAFF |
|  | *Juglans nigra* (Black walnut) (*Wallia nigra*) | | |  |
| 27 | Vicilin seed storage protein | N/A | 481 | >tr\|Q7Y1C1\|Q7Y1C1_JUGNI Vicilin seed storage protein (Fragment) OS=Juglans nigra OX=16719 PE=2 SV=1  GRDRQDPQQQYHRCQRRCQIQEQSPERQRQCQQRCERQYKEQQGRERGPEASPRRESKGR  EEEQQRHNPYYFHSQSIRSRHESEEGEVKYLERFAERTELLRGIENYRVVILDANPNTFM  LPHHKDAESVIVVTRGRATLTLVSQETRESFNLECGDVIRVPAGATEYVINQDSNERLEM  VKLLQPVNNPGQVREYYAAGAKSPDQSYLRVFSNDILVAALNTPRDRLERFFDQQEQREG  VIIRASQEKLRALSQHAMSAGQRPWGRRSSGGPISLKSERPSYSNQFGQFFEACPEEHRQ  LQEMDVLVNYAEIKRGAMMVPHYNSKATVVVYVVEGTGRYEMACPHVSSQSFEDQGRREQ  EEEESTGRFQKVTARLARGDIFVIPAGHPIAITASQNENLRLLGFGINGENNQRNFLAGQ  NSIINQLEREAKELSFNMPREEIEEIFESQMESYFVPTERQSRRGQGRDHPLASILGFAF  F |
| 28 | Vicilin seed storage protein, partial [Juglans nigra] |  |  | >AAM54366.1 vicilin seed storage protein, partial [Juglans nigra]  GRDRQDPQQQYHRCQRRCQIQEQSPERQRQCQQRCERQYKEQQGRERGPEASPRRESKGREEEQQRHNPY  YFHSQSIRSRHESEEGEVKYLERFAERTELLRGIENYRVVILDANPNTFMLPHHKDAESVIVVTRGRATL  TLVSQETRESFNLECGDVIRVPAGATEYVINQDSNERLEMVKLLQPVNNPGQVREYYAAGAKSPDQSYLR  VFSNDILVAALNTPRDRLERFFDQQEQREGVIIRASQEKLRALSQHAMSAGQRPWGRRSSGGPISLKSER  PSYSNQFGQFFEACPEEHRQLQEMDVLVNYAEIKRGAMMVPHYNSKATVVVYVVEGTGRYEMACPHVSSQ  SFEDQGRREQEEEESTGRFQKVTARLARGDIFVIPAGHPIAITASQNENLRLLGFGINGENNQRNFLAGQ  NSIINQLEREAKELSFNMPREEIEEIFESQMESYFVPTERQSRRGQGRDHPLASILGFAFF |
|  | *Carya illinoinensis* (Pecan) | | |  |
| 29 | 7S vicilin | pec1a1a1 | 784 | >tr\|B3STU7\|B3STU7_CARIL 7S vicilin (Fragment) OS=Carya illinoinensis OX=32201 GN=pec1a1a1 PE=2 SV=1  ARGFLSALFLALVCSSLALETEDLSNELNPHHDPESHRWEFQQCQERCQHEERGQRQAQQ  CQRRCEEQLREREREREREEIVDPREPRKQYEQCRETCEKQDPRQQPQCERRCERQFQEQ  QERERRERRRGRDDDDKENPRDPREQYRQCEEHCRRQGQGQRQQQQCQSRCEERFEEEQR  RQEERERRRGRDNDDEENPRDPREQYRQCQEHCRRQGQGQRQQQQCQSRCEERLEEEQRK  QEERERRRGRDEDDQNPRDPEQRYEQCQQQCERQRRGQEQQLCRRRCEQQRQQEERERQR  GRDRQDPQQQYHRCQRRCQTQEQSPERQRQCQQRCERQYKEQQGREWGPDQASPRRESRG  REEEQQRHNPYYFHSQGLRSRHESGEGEVKYLERFTERTELLRGIENYRVVILEANPNTF  VLPYHKDAESVIVVTRGRATLTFVSQERRESFNLEYGDVIRVPAGATEYVINQDSNERLE  MVKLLQPVNNPGQFREYYAAGAQSTESYLRVFSNDILVAALNTPRDRLERFFDQQEQREG  VIIRASQEKLRALSQHAMSAGQRPWGRRSSGGPISLKSQRSSYSNQFGQFFEACPEEHRQ  LQEMDVLVNYAEIKRGAMMVPHYNSKATVVVYVVEGTGRFEMACPHDVSSQSYEYKGRRE  QEEEESSTGQFQKVTARLARGDIFVIPAGHPIAITASQNENLRLVGFGINGKNNQRNFLA  GQNNIINQLEREAKELSFNMPREEIEEIFERQVESYFVPMERQSRRGQGRDHPLASILDF  AGFF |
| 30 | Vicilin Car i 2.0101 | pec2a1a | 792 | >sp\|B3STU4\|VCL_CARIL Vicilin Car i 2.0101 OS=Carya illinoinensis OX=32201 GN=pec2a1a PE=1 SV=1  MVTKAKIPLFLFLSALFLALVCSSLALETEDLSNELNPHHDPESHRWEFQQCQERCQHEE  RGQRQAQQCQRRCEEQLREREREREREEIVDPREPRKQYEQCRETCEKQDPRQQPQCERR  CERQFQEQQERERRERRRGRDDDDKENPRDPREQYRQCEEHCRRQGQGQRQQQQCQSRCE  ERFEEEQRRQEERERRRGRDNDDEENPRDPREQYRQCQEHCRRQGQGQRQQQQCQSRCEE  RLEEEQRKQEERERRRGRDEDDQNPRDPEQRYEQCQQQCERQRRGQEQQLCRRRCEQQRQ  QEERERQRGRDRQDPQQQYHRCQRRCQTQEQSPERQRQCQQRCERQYKEQQGREWGPDQA  SPRRESRGREEEQQRHNPYYFHSQGLRSRHESGEGEVKYLERFTERTELLRGIENYRVVI  LEANPNTFVLPYHKDAESVIVVTRGRATLTFVSQERRESFNLEYGDVIRVPAGATEYVIN  QDSNERLEMVKLLQPVNNPGQFREYYAAGAQSTESYLRVFSNDILVAALNTPRDRLERFF  DQQEQREGVIIRASQEKLRALSQHAMSAGQRPWGRRSSGGPISLKSQRSSYSNQFGQFFE  ACPEEHRQLQEMDVLVNYAEIKRGAMMVPHYNSKATVVVYVVEGTGRFEMACPHDVSSQS  YEYKGRREQEEEESSTGQFQKVTARLARGDIFVIPAGHPIAITASQNENLRLVGFGINGK  NNQRNFLAGQNNIINQLEREAKELSFNMPREEIEEIFERQVESYFVPMERQSRRGQGRDH  PLASILDFAGFF |
| 31 | Putative vicilin-like seed storage protein | N/A | 102 | >tr\|Q15K67\|Q15K67_CARIL Putative vicilin-like seed storage protein (Fragment) OS=Carya illinoinensis OX=32201 PE=4 SV=1  GRDRQDPQQQYHRCQRRCQTQEQSPERQRQCQQRCERQYKEQQGREWGPDQASPRRESRG  REEEQQRHNPYYFHSQGLRSRHESGEGEVKYLERFTERTELL |
|  | Phaseolus vulgaris (Kidney bean) (French bean) | | |  |
| 32 | Phaseolin, beta-type | N/A | 421 | >sp\|P02853\|PHSB_PHAVU Phaseolin, beta-type OS=Phaseolus vulgaris OX=3885 PE=1 SV=2  MMRARVPLLLLGILFLASLSASFATSLREEEESQDNPFYFNSDNSWNTLFKNQYGHIRVL  QRFDQQSKRLQNLEDYRLVEFRSKPETLLLPQQADAELLLVVRSGSAILVLVKPDDRREY  FFLTSDNPIFSDHQKIPAGTIFYLVNPDPKEDLRIIQLAMPVNNPQIHEFFLSSTEAQQS  YLQEFSKHILEASFNSKFEEINRVLFEEEGQQEGVIVNIDSEQIKELSKHAKSSSRKSLS  KQDNTIGNEFGNLTERTDNSLNVLISSIEMEEGALFVPHYYSKAIVILVVNEGEAHVELV  GPKGNKETLEYESYRAELSKDDVFVIPAAYPVAIKATSNVNFTGFGINANNNNRNLLAGK  TDNVISSIGRALDGKDVLGLTFSGSGDEVMKLINKQSGSYFVDAHHHQQEQQKGRKGAFV  Y |
|  | Theobroma cacao (Cacao) | | |  |
| 33 | Vicilin | CSV | 525 | >sp\|Q43358\|VCL_THECC Vicilin OS=Theobroma cacao OX=3641 GN=CSV PE=2 SV=1  MVISKSPFIVLIFSLLLSFALLCSGVSAYGRKQYERDPRQQYEQCQRRCESEATEEREQE  QCEQRCEREYKEQQRQQEEELQRQYQQCQGRCQEQQQGQREQQQCQRKCWEQYKEQERGE  HENYHNHKKNRSEEEEGQQRNNPYYFPKRRSFQTRFRDEEGNFKILQRFAENSPPLKGIN  DYRLAMFEANPNTFILPHHCDAEAIYFVTNGKGTITFVTHENKESYNVQRGTVVSVPAGS  TVYVVSQDNQEKLTIAVLALPVNSPGKYELFFPAGNNKPESYYGAFSYEVLETVFNTQRE  KLEEILEEQRGQKRQQGQQGMFRKAKPEQIRAISQQATSPRHRGGERLAINLLSQSPVYS  NQNGRFFEACPEDFSQFQNMDVAVSAFKLNQGAIFVPHYNSKATFVVFVTDGYGYAQMAC  PHLSRQSQGSQSGRQDRREQEEESEEETFGEFQQVKAPLSPGDVFVAPAGHAVTFFASKD  QPLNAVAFGLNAQNNQRIFLAGRPFFLNHKQNTNVIKFTVKASAY |
| 34 | Vicilin-A, putative | TCM_020665 | 566 | >tr\|A0A061EM85\|A0A061EM85_THECC Vicilin-A, putative OS=Theobroma cacao OX=3641 GN=TCM_020665 PE=4 SV=1  MVISKSPFIVLIFSLLLSFALLCSGVSAYGRKQYERDPRQQYEQCQRRCESEATEEREQE  QCEQRCEREYKEQQRQQEEELQRQYQQCQGRCQEQQQGQREQQQCQRKCWEQYKEQERGE  HENYHNHKKNRSEEEEGQQRNNPYYFPKRRSFQTRFRDEEGNFKILQRFAENSPPLKGIN  DYRLAMFEANPNTFILPHHCDAEAIYFVTNGKGTITFVTHENKESYNVQRGTVVSVPAGS  TVYVVSQDNQEKLTIAVLALPVNSPGKYELFFPAGNNKPESYYGAFSYEVLETVFNTQRE  KLEEILEEQRGQKRQQGQQGMFRKAKPEQIRAISQQATSPRHRGGERLAINLLSQSPVYS  NQNGRFFEACPEDFSQFQNMDVAVSAFKLNQGAIFVPHYNSKATFVVFVTDGYGYAQMAC  PHLSRQSQGSQSGRQDRREQEEESEEETFGEFQQVKAPLSPGDVFVAPAGHAVTFFASKD  QPLNAVAFGLNAQNNQRIFLAGKKNLVRQMDSEAKELSFGVPSKLVDNIFNNPDESYFMS  FSQQRQRGDERRGNPLASILDFARLF |
|  | Vicia faba (Broad bean) (Faba vulgaris) | | |  |
| 35 | Vicilin | N/A | 463 | >sp\|P08438\|VCL_VICFA Vicilin OS=Vicia faba OX=3906 PE=2 SV=1  MAATTLKDSFPLLTLLGIAFLASVCLSSRSDQDNPFVFESNRFQTLFENENGHIRLLQKF  DQHSKLLENLQNYRLLEYKSKPHTIFLPQQTDADFILVVLSGKAILTVLLPNDRNSFSLE  RGDTIKLPAGTIGYLVNRDDEEDLRVLDLVIPVNRPGEPQSFLLSGNQNQPSILSGFSKN  ILEASFNTDYKEIEKVLLEEHGKEKYHRRGLKDRRQRGQEENVIVKISRKQIEELNKNAK  SSSKKSTSSESEPFNLRSREPIYSNKFGKFFEITPKRNPQLQDLNIFVNYVEINEGSLLL  PHYNSRAIVIVTVNEGKGDFELVGQRNENQQGLREEYDEEKEQGEEEIRKQVQNYKAKLS  PGDVLVIPAGYPVAIKASSNLNLVGFGINAENNQRYFLAGEEDNVISQIHKPVKELAFPG  SAQEVDTLLENQKQSHFANAQPRERERGSQEIKDHLYSILGSF |
|  | *Pisum sativum* (Garden pea) | | |  |
| 36 | Vicilin | N/A | 459 | >sp\|P13918\|VCLC_PEA Vicilin OS=Pisum sativum OX=3888 PE=2 SV=2  MAATTMKASFPLLMLMGISFLASVCVSSRSDPQNPFIFKSNKFQTLFENENGHIRLLQKF  DQRSKIFENLQNYRLLEYKSKPHTIFLPQHTDADYILVVLSGKAILTVLKPDDRNSFNLE  RGDTIKLPAGTIAYLVNRDDNEELRVLDLAIPVNRPGQLQSFLLSGNQNQQNYLSGFSKN  ILEASFNTDYEEIEKVLLEEHEKETQHRRSLKDKRQQSQEENVIVKLSRGQIEELSKNAK  STSKKSVSSESEPFNLRSRGPIYSNEFGKFFEITPEKNPQLQDLDIFVNSVEIKEGSLLL  PHYNSRAIVIVTVNEGKGDFELVGQRNENQQEQRKEDDEEEEQGEEEINKQVQNYKAKLS  SGDVFVIPAGHPVAVKASSNLDLLGFGINAENNQRNFLAGDEDNVISQIQRPVKELAFPG  SAQEVDRILENQKQSHFADAQPQQRERGSRETRDRLSSV |
| 37 | 7S vicilin-like protein | Cs7S | 493 | >tr\|A0A219D1T7\|A0A219D1T7_CANSA 7S vicilin-like protein OS=Cannabis sativa OX=3483 GN=Cs7S PE=4 SV=2  MGRIKLTLLVLMLVLSYGVLGIMGFDEDEDWTREKERKKEEEREKEREREDERGELPFIL  RDSKHVMKTDAGEMRVMRSSDSKFAQGPLRIGFITMEPKTLFLPQYLDSELTIFIRRGEV  KLGFIYKDQLAERKLKTGDVYRISAGSAFYLVNTGEGQRLHIICSFDTSESLRIGTFQSF  FLGGGTNPASILSGFDSEILENAFNVTHAELKEILSSQQEGPIVYIPDSRSPKTWAKFLQ  LKQEDKLQHLKKIVGFHQGEEEEDEEELEEDINQEQNQKTTWSWRKLLDSLFANRDKKTK  DYKGKGKSPDSYNLYDGKKGFKNNYGWSIALDEFSYSPLRKSGCGVYLVNLTAGSMMAPH  LNPKATEYGIILKGTGMIQVVYPNGTSAMNTEVKEGDVFWVPRYFPFCQIASRAGPMEFF  GFTTSARKNRPQFLVGASSILRSMRGPELAAAFGLSLERLGNLTSYQRESVILPTSAASP  PVKLAKPIRSLKE |
|  | *Arachis hypogaea* (Peanut) | | |  |
| 38 | Ara h 1 allergen | ara h 1 | 619 | >tr\|E5G076\|E5G076_ARAHY Ara h 1 allergen OS=Arachis hypogaea OX=3818 GN=ara h 1 PE=4 SV=1  MRGRVSPLMLLLGILVLASVSATHAKSSPYQKKTENPCAQRCLQSCQQEPDDLKQKACES  RCTKLEYDPRCVYDPRGHTGTTNQRSPPGERTRGRQPGDYDDDRRQPRREEGGRWGPAGP  REREREEDWRQPREDWRRPSHQQPRKIRPEGREGEQEWGTPGSHVREETSRNNPFYFPSR  RFSTRYGNQNGRIRVLQRFDQRSRQFQNLQNHRIVQIEAKPNTLVLPKHADADNILVIQQ  GQATVTVANGNNRRALILTRAMHSESHPFHFLHLDDMTPELRVAKSHAVNTPGQFEDFFP  ASSRDQSSYLQGFSRNTLEAAFNAEFNEIRRVLLEENAGGEQEERGQRRWSTRSSENNEG  VIVEVSKEHVEELTKHAKSVSKKGSEEEGDITNPINLREGEPDLSDNFGRLFEVKPDKKN  PQLQDLDMMLTCVEIKEGALMLPHFNSKAMVIVVINKGTGNLELVAVRKEQQQRGRREQE  WEEEEEDEEEEGSNREVRRYTARLKEGDVFIMPAAHPVAINASSELHLLGFGINAENNHR  IFLAGDKDNVVDQIEKQAKDLAFPGSGEQVEKLIKNQRESHFVSARPQSQSPSSPEKEDQ  EEENQGGKGPLLSILKAFN |
|  | *Macadamia integrifolia* (Macadamia nut) | | |  |
| 39 | Vicilin-like antimicrobial peptides 2-1 | AMP2-1 | 666 | >sp\|Q9SPL5\|AMP21_MACIN Vicilin-like antimicrobial peptides 2-1 OS=Macadamia integrifolia OX=60698 GN=AMP2-1 PE=2 SV=1  MAINTSNLCSLLFLLSLFLLSTTVSLAESEFDRQEYEECKRQCMQLETSGQMRRCVSQCD  KRFEEDIDWSKYDNQEDPQTECQQCQRRCRQQESGPRQQQYCQRRCKEICEEEEEYNRQR  DPQQQYEQCQKHCQRRETEPRHMQTCQQRCERRYEKEKRKQQKRYEEQQREDEEKYEERM  KEEDNKRDPQQREYEDCRRRCEQQEPRQQHQCQLRCREQQRQHGRGGDMMNPQRGGSGRY  EEGEEEQSDNPYYFDERSLSTRFRTEEGHISVLENFYGRSKLLRALKNYRLVLLEANPNA  FVLPTHLDADAILLVIGGRGALKMIHHDNRESYNLECGDVIRIPAGTTFYLINRDNNERL  HIAKFLQTISTPGQYKEFFPAGGQNPEPYLSTFSKEILEAALNTQTEKLRGVFGQQREGV  IIRASQEQIRELTRDDSESRHWHIRRGGESSRGPYNLFNKRPLYSNKYGQAYEVKPEDYR  QLQDMDLSVFIANVTQGSMMGPFFNTRSTKVVVVASGEADVEMACPHLSGRHGGRGGGKR  HEEEEDVHYEQVRARLSKREAIVVLAGHPVVFVSSGNENLLLFAFGINAQNNHENFLAGR  ERNVLQQIEPQAMELAFAAPRKEVEESFNSQDQSIFFPGPRQHQQQSPRSTKQQQPLVSI  LDFVGF |
| 40 | Vicilin-like antimicrobial peptides 2-2 | AMP2-2 | 666 | >sp\|Q9SPL4\|AMP22_MACIN Vicilin-like antimicrobial peptides 2-2 OS=Macadamia integrifolia OX=60698 GN=AMP2-2 PE=2 SV=1  MAINTSNLCSLLFLLSLFLLSTTVSLAESEFDRQEYEECKRQCMQLETSGQMRRCVSQCD  KRFEEDIDWSKYDNQDDPQTDCQQCQRRCRQQESGPRQQQYCQRRCKEICEEEEEYNRQR  DPQQQYEQCQERCQRHETEPRHMQTCQQRCERRYEKEKRKQQKRYEEQQREDEEKYEERM  KEEDNKRDPQQREYEDCRRRCEQQEPRQQYQCQRRCREQQRQHGRGGDLINPQRGGSGRY  EEGEEKQSDNPYYFDERSLSTRFRTEEGHISVLENFYGRSKLLRALKNYRLVLLEANPNA  FVLPTHLDADAILLVTGGRGALKMIHRDNRESYNLECGDVIRIPAGTTFYLINRDNNERL  HIAKFLQTISTPGQYKEFFPAGGQNPEPYLSTFSKEILEAALNTQAERLRGVLGQQREGV  IISASQEQIRELTRDDSESRRWHIRRGGESSRGPYNLFNKRPLYSNKYGQAYEVKPEDYR  QLQDMDVSVFIANITQGSMMGPFFNTRSTKVVVVASGEADVEMACPHLSGRHGGRRGGKR  HEEEEDVHYEQVKARLSKREAIVVPVGHPVVFVSSGNENLLLFAFGINAQNNHENFLAGR  ERNVLQQIEPQAMELAFAAPRKEVEELFNSQDESIFFPGPRQHQQQSSRSTKQQQPLVSI  LDFVGF |
| 41 | Vicilin-like antimicrobial peptides 2-3 | AMP2-3 | 625 | >sp\|Q9SPL3\|AMP23_MACIN Vicilin-like antimicrobial peptides 2-3 (Fragment) OS=Macadamia integrifolia OX=60698 GN=AMP2-3 PE=1 SV=1  QCMQLETSGQMRRCVSQCDKRFEEDIDWSKYDNQEDPQTECQQCQRRCRQQESDPRQQQY  CQRRCKEICEEEEEYNRQRDPQQQYEQCQKRCQRRETEPRHMQICQQRCERRYEKEKRKQ  QKRYEEQQREDEEKYEERMKEGDNKRDPQQREYEDCRRHCEQQEPRLQYQCQRRCQEQQR  QHGRGGDLMNPQRGGSGRYEEGEEKQSDNPYYFDERSLSTRFRTEEGHISVLENFYGRSK  LLRALKNYRLVLLEANPNAFVLPTHLDADAILLVIGGRGALKMIHRDNRESYNLECGDVI  RIPAGTTFYLINRDNNERLHIAKFLQTISTPGQYKEFFPAGGQNPEPYLSTFSKEILEAA  LNTQTERLRGVLGQQREGVIIRASQEQIRELTRDDSESRRWHIRRGGESSRGPYNLFNKR  PLYSNKYGQAYEVKPEDYRQLQDMDVSVFIANITQGSMMGPFFNTRSTKVVVVASGEADV  EMACPHLSGRHGGRGGGKRHEEEEEVHYEQVRARLSKREAIVVLAGHPVVFVSSGNENLL  LFAFGINAQNNHENFLAGRERNVLQQIEPQAMELAFAASRKEVEELFNSQDESIFFPGPR  QHQQQSPRSTKQQQPLVSILDFVGF |
|  | *Anacardium occidentale* (Cashew) | | |  |
| 42 | Vicilin-like protein | Ana o 1.0101 | 538 | >tr\|Q8L5L5\|Q8L5L5_ANAOC Vicilin-like protein OS=Anacardium occidentale OX=171929 GN=Ana o 1.0101 PE=2 SV=1  MGPPTKFSFSLFLVSVLVLCLGFALAKIDPELKQCKHQCKVQRQYDEQQKEQCVKECEKY  YKEKKGREREHEEEEEEWGTGGVDEPSTHEPAEKHLSQCMRQCERQEGGQQKQLCRFRCQ  ERYKKERGQHNYKREDDEDEDEDEAEEEDENPYVFEDEDFTTKVKTEQGKVVLLPKFTQK  SKLLHALEKYRLAVLVANPQAFVVPSHMDADSIFFVSWGRGTITKILENKRESINVRQGD  IVSISSGTPFYIANNDENEKLYLVQFLRPVNLPGHFEVFHGPGGENPESFYRAFSWEILE  AALKTSKDTLEKLFEKQDQGTIMKASKEQIRAMSRRGEGPKIWPFTEESTGSFKLFKKDP  SQSNKYGQLFEAERIDYPPLEKLDMVVSYANITKGGMSVPFYNSRATKIAIVVSGEGCVE  IACPHLSSSKSSHPSYKKLRARIRKDTVFIVPAGHPFATVASGNENLEIVCFEVNAEGNI  RYTLAGKKNIIKVMEKEAKELAFKMEGEEVDKVFGKQDEEFFFQGPEWRKEKEGRADE |
| 43 | Vicilin-like protein | Ana o 1.0102 | 536 | >tr\|Q8L5L6\|Q8L5L6_ANAOC Vicilin-like protein (Fragment) OS=Anacardium occidentale OX=171929 GN=Ana o 1.0102 PE=2 SV=1  PPTKFSFSLFLVSVLVLCLGFALAKIDPELKQCKHQCKVQRQYDEQQKEQCVKECEKYYK  EKKGREREHEEEEEEWGTGGVDEPSTHEPAEKHLSQCMRQCERQEGGQQKQLCRFRCQER  YKKERGQHNYKREDDEDEDEDEAEEEDENPYVFEDEDFTTKVKTEQGKVVLLPKFTQKSK  LLHALEKYRLAVLVANPQAFVVPSHMDADSIFFVSWGRGTITKILENKRESINVRQGDIV  SISSGTPFYIANNDENEKLYLVQFLRPVNLPGHFEVFHGPGGENPESFYRAFSWEILEAA  LKTSKDTLEKLFEKQDQGTIMKASKEQVRAMSRRGEGPKIWPFTEESTGSFKLFKKDPSQ  SNKYGQLFEAERIDYPPLEKLDMVVSYANITKGGMSVPFYNSRATKIAIVVSGEGCVEIA  CPHLSSSKSSHPSYKKLRARIRKDTVFIVPAGHPFATVASGNENLEIVCFEVNAEGNIRY  TLAGKKNIIKVMEKEAKELAFKMEGEEVDKVFGKQDEEFFFQGPEWRKEKEGRADE |
|  | *Corylus avellana* (European hazel) | | |  |
| 44 | 48-kDa glycoprotein | N/A | 448 | >tr\|Q8S4P9\|Q8S4P9_CORAV 48-kDa glycoprotein OS=Corylus avellana OX=13451 PE=1 SV=1  MLPKEDPELKKCKHKCRDERQFDEQQRRDGKQICEEKARERQQEEGNSSEESYGKEQEEN  PYVFQDEHFESRVKTEEGRVQVLENFTKRSRLLSGIENFRLAILEANPHTFISPAHFDAE  LVLFVAKGRATITMVREEKRESFNVEHGDIIRIPAGTPVYMINRDENEKLFIVKILQPVS  APGHFEAFYGAGGEDPESFYRAFSWEVLEAALKVRREQLEKVFGEQSKGSIVKASREKIR  ALSQHEEGPPRIWPFGGESSGPINLLHKHPSQSNQFGRLYEAHPDDHKQLQDLDLMVSFA  NITKGSMAGPYYNSRATKISVVVEGEGFFEMACPHLSSSSGSYQKISARLRRGVVFVAPA  GHPVAVIASQNNNLQVLCFEVNAHGNSRFPLAGKGNIVNEFERDAKELAFNLPSREVERI  FKNQDQAFFFPGPNKQQEEGGRGGRAFE |
|  | *Pistacia vera* (Pistachio) | | |  |
| 45 | Vicilin | N/A | 519 | >tr\|B4X640\|B4X640_PISVE Vicilin (Fragment) OS=Pistacia vera OX=55513 PE=2 SV=1  KTDPELKQCKHQCKVQRQYDEEQKEQCAKGCEKYYKEKKGREQEEEEEEEWGSGRGRGDE  FSTHEPGEKRLSQCMKQCERQDGGQQKQLCRFRCQEKYKKERREHSYSRDEEEEEEGDEE  QEEEDENPYVFEDEHFTTRVKTEQGKVVVLPKFTKRSKLLRGLEKYRLAFLVANPQAFVV  PNHMDADSIFFVSWGRGTITKIRENKRESMNVKQGDIIRIRAGTPFYIVNTDENEKLYIV  KLLQPVNLPGHYEVFHGPGGENPESFYRAFSREVLEAALKTPRDKLEKLFEKQDEGAIVK  ASKEQIRAMSRRGEGPSIWPFTGKSTGTFNLFKKDPSQSNNYGQLFESEFKDYPPLQELD  IMVSYVNITKGGMSGPFYNSRATKIAIVVSGEGRLEIACPHLSSSKNSGQEKSGPSYKKL  SSSIRTDSVFVVPAGHPFVTVASGNQNLEILCFEVNAEGNIRYTLAGKKNIIEVMEKEAK  ELAFKTKGEEVDKVFGKQDEEFFFQGPKWRQHQQGRADE |
|  | *Sesamum indicum* (Oriental sesame) | | |  |
| 46 | 7S globulin | N/A | 585 | >tr\|Q9AUD0\|Q9AUD0_SESIN 7S globulin OS=Sesamum indicum OX=4182 PE=2 SV=1  MSCGGRLCLVLFALLLASAVVASESKDPELKQCKHQCKAQQQISKEQKEACIQACKEYIR  QKHQGEHGRGGGDILEEEVWNRKSPIERLRECSRGCEQQHGEQREECLRRCQEEYQREKG  RQDDDNPTDPEKQYQQCRLQCRRQGEGGGFSREHCERRREEKYREQQGREGGRGEMYEGR  EREEEQEEQGRGRIPYVFEDQHFITGFRTQHGRMRVLQKFTDRSELLRGIENYRVAILEA  EPQTFIVPNHWDAESVVFVAKGRGTISLVRQDRRESLNIKQGDILKINAGTTAYLINRDN  NERLVLAKLLQPVSTPGEFELFFGAGGENPESFFKSFSDEILEAAFNTRRDRLQRIFGQQ  RQGVIVKASEEQVRAMSRHEEGGIWPFGGESKGTINIYQQRPTHSNQYGQLHEVDASQYR  QLRDLDLTVSLANITQGAMTAPHYNSKATKIALVVDGEGYFEMACPHMSRSRGSYQGETR  GRPSYQRVASRLTRGTVVIIPAGHPFVAVASSNQNLQVLCFEVNANNNEKFPLAGRRNVM  NQLEREAKELAFGMPAREVEEVSRSQQEEFFFKGPRQQQQGRADA |
|  | *Lupinus angustifolius* (Narrow-leaved blue lupine) | | |  |
| 47 | Conglutin beta 1 | BETA1 | 611 | >sp\|F5B8V9\|CONB1_LUPAN Conglutin beta 1 OS=Lupinus angustifolius OX=3871 GN=BETA1 PE=1 SV=1  MAKMRVRLPMLILLLGVVFLLAASIGIAYGEKDFTKNPPKEREEEEHEPRQQPRPRQQEE  QEREHRREEKHDGEPSRGRSQSEESQEEEHERRREHHREREQEQQPRPQRRQEEEEEEEE  WQPRRQRPQSRREEREEREQEQGSSSGSQRGSGDERRQHRERRVHREEREQEQDSRSDSR  RQRNPYHFSSNRFQTYYRNRNGQIRVLERFNQRTNRLENLQNYRIIEFQSKPNTLILPKH  SDADFILVVLNGRATITIVNPDKRQVYNLEQGDALRLPAGTTSYILNPDDNQNLRVAKLA  IPINNPGKLYDFYPSTTKDQQSYFSGFSKNTLEATFNTRYEEIERVLLGDDELQENEKQR  RGQEQSHQDEGVIVRVSKKQIQELRKHAQSSSGEGKPSESGPFNLRSNKPIYSNKFGNFY  EITPDINPQFQDLNISLTFTEINEGALLLPHYNSKAIFIVVVDEGEGNYELVGIRDQQRQ  QDEQEEEYEQGEEEVRRYSDKLSKGDVFIIPAGHPLSINASSNLRLLGFGINANENQRNF  LAGSEDNVIKQLDREVKELTFPGSIEDVERLIKNQQQSYFANAQPQQQQQREKEGRRGRR  GPISSILNALY |
|  | *Lupinus albus* (White lupine) | | |  |
| 48 | Conglutin beta 2 | N/A | 533 | >sp\|Q6EBC1\|CONB2_LUPAL Conglutin beta 2 OS=Lupinus albus OX=3870 PE=1 SV=1  MGKMRVRFPTLVLVLGIVFLMAVSIGIAYGEKDVLKSHERPEEREQEEWQPRRQRPQSRR  EEREQEQEQGSPSYPRRQSGYERRQYHERSEQREEREQEQQQGSPSYSRRQRNPYHFSSQ  RFQTLYKNRNGKIRVLERFDQRTNRLENLQNYRIVEFQSKPNTLILPKHSDADYVLVVLN  GRATITIVNPDRRQAYNLEYGDALRIPAGSTSYILNPDDNQKLRVVKLAIPINNPGYFYD  FYPSSTKDQQSYFSGFSRNTLEATFNTRYEEIQRIILGNEDEQEYEEQRRGQEQSDQDEG  VIVIVSKKQIQKLTKHAQSSSGKDKPSDSGPFNLRSNEPIYSNKYGNFYEITPDRNPQVQ  DLNISLTYIKINEGALLLPHYNSKAIYVVVVDEGEGNYELVGIRDQQRQQDEQEEKEEEV  IRYSARLSEGDIFVIPAGYPISINASSNLRLLGFGINADENQRNFLAGSKDNVIRQLDRA  VNELTFPGSAEDIERLIKNQQQSYFANGQPQQQQQQQSEKEGRRGRRGSSLPF |

*Brassica rapa candidate* 7S globulins/vicilin sequences identified and characterised according to the same method described in (Rahman et al., 2020a) and sequences of 7S globulins/vicilin protein genes reported in *A. thaliana* and some other species available in the public domain databases.* Entries are included from Uniprot and NCBI protein database search with the key word “vicilin” and “7S globulin”. The entries other than these two key words were included based on the published literature (Jimenez-Lopez et al., 2016).

**S2 Table.** Sequences of oleosin protein genes reported in *A. thaliana* and some other species available in the public domain databases.

| Sl. | Oleosins Sequence (5'-3') in FASTA format. OS= Organism species. |
| --- | --- |
| 1 | >sp\|Q9LY09\|GRP17_ARATH Oleosin GRP-17 OS=Arabidopsis thaliana OX=3702 GN=GRP17 PE=1 SV=1  MSEELSQKPSSAQSLSLREGRNRFPFLSLSQREGRFFPSLSLSERDGRKFSFLSMFSFLM  PLLEVIKIIIASVASVIFVGFACVTLAGSAAALVVSTPVFIIFSPVLVPATIATVVLATG  FTAGGSFGATALGLIMWLVKRRMGVKPKDNPPPAGLPPNSGAGAGGAQSLIKKSKAKSKG  GLKAWCKKMLKSKFGGKKGKSGGGKSKFGGKGGKSEGEEGMSSGDEGMSGSEGGMSGGEG  GKSKSGKGKLKAKLEKKKGMSGGSESEEGMSGSEGGMSGGGGSKSKSKKSKLKAKLGKKK  GMSGGMSGSEEGMSGSEGGMSSGGGSKSKSKKSKLKAKLGKKKSMSGGMSGSEEGMSGSE  GGMSGGGGGKSKSRKSKLKANLGKKKCMSGGMSGSEGGMSRSEGGISGGGMSGGSGSKHK  IGGGKHGGLGGKFGKKRGMSGSGGGMSGSEGGVSGSEGSMSGGGMSGGSGSKHKIGGGKH  GGLRGKFGKKRGMSGSEGGMSGSEGGMSESGMSGSGGGKHKIGGGKHKFGGGKHGGGGGH  MAE |
| 2 | >sp\|Q9SQ57\|PXG_SESIN Peroxygenase OS=Sesamum indicum OX=4182 GN=SOP1 PE=1 SV=1  MATHVLAAAAERNAALAPDAPLAPVTMERPVRTDLETSIPKPYMARGLVAPDMDHPNGTP  GHVHDNLSVLQQHCAFFDQDDNGIIYPWETYSGLRQIGFNVIASLIMAIVINVALSYPTL  PGWIPSPFFPIYLYNIHKAKHGSDSGTYDTEGRYLPMNFENLFSKHARTMPDRLTLGELW  SMTEANREAFDIFGWIASKMEWTLLYILARDQDGFLSKEAIRRCYDGSLFEYCAKMQRGA  EDKMK |
| 3 | >sp\|Q00650\|OLNB1_BRANA Oleosin-B1 OS=Brassica napus OX=3708 GN=OlnB1 PE=1 SV=2  MGILRKKKHERNASFKSVLTSILATQAATFLLLISGVSLAGTAAAFIATMPLFVVFSPIL  VPAGITTGLLTTGLAAAGGAGATAVTIILWLYKRATGKEPPAVLSKVLKKIIPGAAAAPR  AAPAAAPAAAPAAAPAAAPAPKPAAAPAPKPAAPPAL |
| 4 | >sp\|Q39165\|OLEO2_ARATH Oleosin 21.2 kDa OS=Arabidopsis thaliana OX=3702 GN=At5g40420 PE=2 SV=1  MADTHRVDRTDRHFQFQSPYEGGRGQGQYEGDRGYGGGGYKSMMPESGPSSTQVLSLLIG  VPVVGSLLALAGLLLAGSVIGLMVALPLFLLFSPVIVPAALTIGLAMTGFLASGMFGLTG  LSSISWVMNYLRGTRRTVPEQLEYAKRRMADAVGYAGQKGKEMGQHVQNKAQDVKQYDIS  KPHDTTTKGHETQGRTTAA |
| 5 | >sp\|Q43402\|OLNB6_BRANA Oleosin-B6 OS=Brassica napus OX=3708 GN=OlnB6 PE=1 SV=3  MKEEIQNETAQTQLQREGRMFSFLFPVIEVIKVVMASVASVVFLGFGGVTLACSAVALAV  STPLFIIFSPILVPATIATTLLATGLGAGTTLGVTGMGLLMRLIKHPGKEGAASAPAAQP  SFLSLLEMPNFIKSKMLERLIHIPGVGKKSEGRGESKGKKGKKGKSEHGRGKHEGEGKSK  GRKGHRMGVNPENNPPPAGAPPTGSPPAAPAAPEAPAAPAAPAAPAAPAAPAAPAAPEDP  AAPAAPEAPATPAAPPAPAAAPAPAAPAAPPAPAAPPRPPSFLSLLEMPSFIKSKLIEAL  INIPGFGKKSNDRGKSKGGKKSKGKGKSNGRGKHEGEGKSKSRKSKSRGKDKEKSKGKGI  FGRSSRKGSSDDESS |
| 6 | >sp\|P29525\|OLEO1_ARATH Oleosin 18.5 kDa OS=Arabidopsis thaliana OX=3702 GN=At4g25140 PE=2 SV=1  MADTARGTHHDIIGRDQYPMMGRDRDQYQMSGRGSDYSKSRQIAKAATAVTAGGSLLVLS  SLTLVGTVIALTVATPLLVIFSPILVPALITVALLITGFLSSGGFGIAAITVFSWIYKYA  TGEHPQGSDKLDSARMKLGSKAQDLKDRAQYYGQQHTGGEHDRDRTRGGQHTT |
| 7 | >sp\|Q42431\|OLEO4_ARATH Oleosin 20.3 kDa OS=Arabidopsis thaliana OX=3702 GN=OL2 PE=2 SV=1  MANVDRDRRVHVDRTDKRVHQPNYEDDVGFGGYGGYGAGSDYKSRGPSTNQILALIAGVP  IGGTLLTLAGLTLAGSVIGLLVSIPLFLLFSPVIVPAALTIGLAVTGILASGLFGLTGLS  SVSWVLNYLRGTSDTVPEQLDYAKRRMADAVGYAGMKGKEMGQYVQDKAHEARETEFMTE  THEPGKARRGS |
| 8 | >sp\|Q42626\|OLNB3_BRANA Oleosin-B3 OS=Brassica napus OX=3708 GN=OlnB3 PE=1 SV=1  MRNEIQNETAQTDQTQGSMFSFFNLFPFLLPMFEVIKMVVASVASVVYLGFAGVTLSGSA  VALAVSTPLFIIFSPILLPAIAATTVLAAGLGSKKVAAAPAASPSLSLLGIPESIKPSNV  IPESIKPSNIIPESIKPSNIIPVSIKPSNIKDKIKDTIGKVKNKIKAKQEEKSKGKSEDS  SKGKGKSKGEDTTTDEDKHGKGESKHGKGESKHGKGESTHGKGGKHGSEGSSMDEGKHGG  KHGSGGSPMGGGKHGSGGKHESGGSPMGGGKHGSGGKHESGGASMGGGKHESVGKHGSGG  KHESGGSPMGGGKHGSGGKHESGGASMGGGKHGSGGRHEGGGSAMGGGKHGSGGKHGSEG  KHGGEGSSMGKNSLSKNKKEFHYRGQAMDASSTSESSDGSSSDGSSSDGSSSDGSSHGSG  GKHI |
| 9 | >sp\|Q42627\|OLNB4_BRANA Oleosin-B4 OS=Brassica napus OX=3708 GN=OlnB4 PE=1 SV=1  MRNEIQNETAQTDQTQGSMFSFFNLFPFLLPMFEVIKMVVASVASVVYLGFAGVTLSGSA  VALAVSTPLFIIFSPILLPAIAATTVLAAGLGGKKVAAAPEASPAASPSLSLLGIPESIK  PSNIIPESIKPSNIIPEGIKPSNIKDKIKDTIGKVKNKIKAKKEEKSKGKSEDSSKGKGK  SKGEDTTTDDDTTTDEDKHGSGAKHGKGESKHGKGESTHGKGGKHGSEGKHGSGGSSMGG  GKHGSGGKHETGGKHGSGGKHESGGSPMGGGKHGSEGKHGSGGASMGGGKHGSGGKHESG  GSAMGGGKHGSGGKHGSEGKHGGEGSSMGKNSLSKKKKEFHYRGQAMDASSTSESSDGSS  DGSSSDGSSHGSGGKHI |
| 10 | >sp\|P29526\|OLNB2_BRANA Oleosin-B2 (Fragment) OS=Brassica napus OX=3708 GN=OlnB2 PE=1 SV=1  QASIFSRFFRMFSFIFPFVNVIKLIIASVTSLVCLAFSCVALGGSAVALIVSTPLFIMFS  PILVPATIATTLLASGLMAGTTLGLTGIGLIMGLVRTAGGVSLLQSPLRKIIVNRIKARL  GGGGGGSRLARLKKILGLLNKLRGMGAGGAAAPAAEPAPAAEAAPAAEAAPAAAPAAAPA  AAP |
| 11 | >sp\|P29531\|OLEO2_SOYBN P24 oleosin isoform B OS=Glycine max OX=3847 PE=2 SV=1  MTTVPPHSVQVHTTTHRYEAGVVPPARFEAPRYEAGIKAPSSIYHSERGPTTSQVLAVVA  GLPVGGILLLLAGLTLAGTLTGLVVATPLFIIFSPVLIPATVAIGLAVAGFLTSGVFGLT  ALSSFSWILNYIRETQPASENLAAAAKHHLAEAAEYVGQKTKEVGQKTKEVGQDIQSKAQ  DTREAAARDARDAREAAARDARDAKVEARDVKRTTVTATTATA |
| 12 | >sp\|P29530\|OLEO1_SOYBN P24 oleosin isoform A OS=Glycine max OX=3847 PE=2 SV=2  MTTQVPPHSVQVHTTTTHRYEAGVVPPGARFETSYEAGVKAASIYHSERGPTTSQVLAVL  AGLPVGGILLLLAGLTLAGTLTGLAVATPLFVLFSPVLVPATVAIGLAVAGFLTSGAFGL  TALSSFSWILNYIRETQPASENLAAAAKHHLAEAAEYVGQKTKEVGQKTKEVGQDIQSKA  QDTREAAARDAREAAARDAREAAARDAKVEARDVKRTTVTATTATA |
| 13 | >sp\|C3S7F1\|OLES2_BRANA Oleosin S2-2 OS=Brassica napus OX=3708 GN=S2 PE=1 SV=1  MATVERRVQVDPTDKRIHLQPQYEGDVGYGYGYGGRADYKSSGPSSNQIVALIVGVPVGG  SLLALAGLTLAGSVIGLMLSVPLFLLFSPVIVPAAITIGLAVTAILASGLFGLTGLSSVS  WVLNYLRGTSDTVPEQLDYAKRRMADAVGYAGQKGKEMGQYVQDKAHEAHDTSLTTETTE  PGKTRRHT |
| 14 | >sp\|C3S7F0\|OLES1_BRANA Oleosin S1-2 OS=Brassica napus OX=3708 GN=S1 PE=1 SV=1  MADVRTHAHQVQVHPLRQHEGGIKVVYPQSGPSSTQVLAVVAGVPVGGTLLTLAGLTLAV  SVIGLILAFPLFLIFSPVIVPAAFVIGLAMTGFMASGAIGLTGLSSMSWVLNHIRRVRER  IPDELDEAKQRLADMAEYAGQRTKDAGQTIEDKAHDVRESKTYDVRDRDTKGHTASGGDR  DTKTTREVRVATT |
| 15 | >sp\|P29110\|OLEO3_BRANA Oleosin Bn-III OS=Brassica napus OX=3708 PE=2 SV=1  MTDTARTHHDITSRDQYPRDRDQYSMIGRDRDQYSMMGRDRDQYNMYGRDYSKSRQIAKA  VTAVTAGGSLLVLSSLTLVGTVIALTVATPLLVIFSPILVPALITVAMLITGFLSSGGFG  IAAITVFSWIYKYATGEHPQGSDKLDSARMKLGSKAQDLKDRAQYYGQQHTGGYGQQHTG  GEHDRDRTRGTQHTT |
| 16 | >sp\|P29109\|OLEO5_BRANA Oleosin Bn-V (Fragment) OS=Brassica napus OX=3708 PE=2 SV=1  PARTHHDITTRDQYPLISRDRDQYGMIGRDQYNMSGQNYSKSRQIAKATTAVTAGDSLLV  LSSLTLVGTVIALIVATPLLVIFSPILVPALITVALLITGFLSSGAFGIAAITVFSWIYK  YATGEHPQGSDKLDSARMKLGSKAQDMKDRAYYYGQQHTGEEHDRDRDHRTDRDRTRGTQ  HTT |
| 17 | >sp\|P29111\|OLEO2_BRANA Major oleosin NAP-II (Fragment) OS=Brassica napus OX=3708 PE=1 SV=1  RRDQYPRDRDQYSMIGRDRDKYSMIGRDRDQYNMYGRDYSKSRQIAKAVTAVTAGGSLLV  LSSLTLVGTVIALTVATPLLVIFSPILVPALITVALLITGFLSSGGFGIAAITVFSWIYK  YATGEHPQGSDKLDSARMKLGGKVQDMKDRAQYYGQQQTGGEDDRDRTRGTQHTT |
| 18 | >tr\|Q9XI93\|Q9XI93_ARATH At1g13930/F16A14.27 OS=Arabidopsis thaliana OX=3702 GN=F7A19.2 PE=1 SV=1  MNFISDQVKKLSSSTPEEPDHNKPVEGTETATRPATNAELMASAKVVAEAAQAAARNESD  KLDKGKVAGASADILDAAEKYGKFDEKSSTGQYLDKAEKYLNDYESSHSTGAGGPPPPTS  QAEPASQPEPAAKKDDEESGGGLGGYAKMAQGFLK |
| 19 | >sp\|Q43284\|OLEO3_ARATH Oleosin 14.9 kDa OS=Arabidopsis thaliana OX=3702 GN=OL3 PE=2 SV=2  MADQTRTHHEMISRDSTQEAHPKARQMVKAATAVTAGGSLLVLSGLTLAGTVIALTVATP  LLVIFSPVLVPAVVTVALIITGFLASGGFGIAAITAFSWLYRHMTGSGSDKIENARMKVG  SRVQDTKYGQHNIGVQHQQVS |
| 20 | >sp\|Q43804\|OLEO1_PRUDU Oleosin 1 OS=Prunus dulcis OX=3755 GN=OLE1 PE=2 SV=1  MADQHFQQPLHFQGSYGQQQPRSYQVAKAATAVTAGGSLLVLSGLVLAGTVIALTIATPL  LVIFSPVLVPALITVALITMGFLTSGGFGVAAVTVLSWIYKYVTGKQPPGADQLDQARHK  LAGKARDIKDRAEQFGQQHVPSGQQQSS |
| 21 | >sp\|P29529\|OLEO_HELAN Oleosin (Fragment) OS=Helianthus annuus OX=4232 PE=2 SV=1  TTTTYDRHFTTTQPHYRQDDRSRYDQQTHSQSTSRTLAIIALLPVGGILLGLAALTFIGT  LIGLALATPLFVIFSPIIVPAVLTIGLAVTGFLASGTFGLTGLSSLSYLFNMVRQTAGSV  PESLDYVKGTLQDAGEYAGQKTKDFGQKIQSTAHEMGDQGQVGVHAQVGGGKEGRKSGDR  T |
| 22 | >sp\|Q9SS98\|OLEO5_ARATH Oleosin 5 OS=Arabidopsis thaliana OX=3702 GN=At3g01570 PE=2 SV=1  MADVRTHSHQLQVHPQRQHEGGIKVLYPQSGPSSTQVLAVFVGVPIGGTLLTIAGLTLAG  SVIGLMLAFPLFLIFSPVIVPAAFVIGLAMTGFLASGAIGLTGLSSMSWVLNYIRRAGQH  IPEELEEAKHRLADMAEYVGQRTKDAGQTIEDKAHDVREAKTFDVRDRDTTKGTHNVRDT  KTT |
| 23 | >tr\|Q9LY08\|Q9LY08_ARATH Oleosin OS=Arabidopsis thaliana OX=3702 GN=ATGRP-6 PE=2 SV=1  MLSFLIPVVQFFQVVIAAVASVVFLVFAGITFGASIVGLTIATPLFVIFSPILVPATIAT  TFLVGGATAAVALGVTAFALILWLFKHRIGVKPKNNPAPKGAPTKADQPGASGGASGDKP  GEMSGAGGPSGDKPGGASGGGDKPGGASGGGPGGASGGASGGASGGASGGASGGASGGGP  GGASGGGPGGASGGGPGGASGGASGDKPEGAPGDKPGGAWGGKPGKKPGHKPEGARGGKR  LAWW |
| 24 | >tr\|Q42574\|Q42574_ARATH Oleosin OS=Arabidopsis thaliana OX=3702 GN=ATGRP19 PE=2 SV=1  MFEIIQAVFSAGVALALLTFAGITLGGSVVACIISTPLFVIFSPVLVPATIATTLLASGF  TASGSFGATAFTILSWLYKKRTGRDLPKIPGLTPPAPASNPAGSGV |
| 25 | >tr\|G7I2Z9\|G7I2Z9_MEDTR Oleosin OS=Medicago truncatula OX=3880 GN=11420287 PE=3 SV=1  MADTGGHYQPLRGYNQQHSTTTQQQPSSKLTQLLKSSTAVTAGGSLLILSGLVLAGTVIA  LTIATPLFLLFSPVLVPAVITVALLTLGFFVSGGFGVAAITVLAWIYRYVTGKHPPGADQ  LDTARHKLMNKAREIKDYGQQQISGTQNS |
| 26 | >tr\|D7MGJ2\|D7MGJ2_ARALL Oleosin OS=Arabidopsis lyrata subsp. lyrata OX=81972 GN=ARALYDRAFT_492319 PE=3 SV=1  MADTGRTTHHDIIGRDQYPMIGRDRDQYQMSGRGSDYSKSRQIAKAATAVTAGGSLLVLS  SLTLVGTVIALTVATPLLVIFSPILVPALITVALLITGFLSSGGFGIAAITVFSWIYKYA  TGEHPQGSDRLDSARMKLGSKAQDLKDRAQYYGQQHTGGEHDRDRTRVGQHTT |
| 27 | >tr\|A0A1P8BDM8\|A0A1P8BDM8_ARATH Oleosin OS=Arabidopsis thaliana OX=3702 GN=GRP17 PE=3 SV=1  MSEELSQKPSSAQSLSLREGRNRFPFLSLSQREGRFFPSLSLSERDGRKFSFLSMFSFLM  PLLEVIKIIIASVASVIFVGFACVTLAGSAAALVVSTPVFIIFSPVLVPATIATVVLATG  FTAGGSFGATALGLIMWLVKRRMGVKPKDNPPPAGLPPNSGAGAGGAQSLIKKSKAKSKG  GLKAWCKKMLKSKFGGKKGKSGGGKSKFGGKGGKSEGEEGMSSGDEGMSGSEGGMSGGEG  GKSKSGKGKLKAKLEKKKGMSGGSESEEGMSGSEGGMSGGGGSKSKSKKSKLKAKLGKKK  GMSGGMSGSEEGMSGSEGGMSSGGGSKSKSKKSKLKAKLGKKKSMSGGMSGSEEGMSGSE  GGMSGGGGGMSGSEGGMSRSEGGISGGGMSGGSGSKHKIGGGKHGGLGGKFGKKRGMSGS  GGGMSGSEGGVSGSEGSMSGGGMSGGSGSKHKIGGGKHGGLRGKFGKKRGMSGSEGGMSG  SEGGMSESGMSGSGGGKHKIGGGKHKFGGGKHGGGGGHMAE |
| 28 | >tr\|I1N747\|I1N747_SOYBN Oleosin OS=Glycine max OX=3847 GN=GLYMA_19G063400 PE=3 SV=1  MTTQVPPHSVQVHTTTHRYEAGVVPPGARFEPPRYEAGVKAPSIYHSERGPTTSQVLAVL  AGLPVGGILLLLAGLTLAGTLTGLAVATPLFVLFSPVLVPATVAIGLAVAGFLTSGAFGL  TALSSFSWILNYIRETQPASENLAAAAKHHLAEAAEYVGQKTKEVGQKTKEVGQDIQSKA  QDTREAAARDAREAAARDAREAAARDAKVEARDVKRTTVTATTATA |
| 29 | >tr\|M4E9X1\|M4E9X1_BRARP Oleosin OS=Brassica rapa subsp. pekinensis OX=51351 PE=3 SV=1  MADTHRVDRTDRHLQFQSPYEGGRVNIQFEGAGGGYGQSGYGGGGGYGQSGYGGGGYKSM  MPESGPSSTQVISFLVGVPLVGSLLAIAGLLLAGSVIGLMISIPLFLLFSPVIVPAAITI  GLATTGFLTSGMFGLTGLSSISWVMNYLRRTRGSVPDQLEYAKRRMADAVGYAGQKGKEV  GQFVQDKAHDAKQYDISKPHDTTTTTTTTTKGLETRTAAA |
| 30 | >tr\|M4DRP3\|M4DRP3_BRARP Oleosin OS=Brassica rapa subsp. pekinensis OX=51351 PE=3 SV=1  MTDTARTHHDITTRDPYPMMGRDRDQYAIIGRDQYQGYGQDYSKSRQIAKAATAVTAGGS  LLVLSSLTLVGTVIALIVATPLLVIFSPILVPALITVALLITGFLSSGGFGIAAITVFSW  IYKYATGEHPKGSDKLDSARMKLGSKAQDMKDRAHYYGQQHTGGEHVNTDYRNTDRDRTR  GNTT |
| 31 | >tr\|M4EI43\|M4EI43_BRARP Oleosin OS=Brassica rapa subsp. pekinensis OX=51351 PE=3 SV=1  MADTHRVDRTDRHLQFQSPYEGGRVNIQYEGGGGAGGYGGGRGGGYGAGGYKSMMPERGP  SSTQVLSFLVGVPIVGSLLAIAGLLLAGSVIGLLISIPLFLLFSPVIVPAALTIGLAATG  FLASGMFGLTGLSSVSWVMNYLRGTRKSSVPEQLEYAKKRMADAVGYAGQKGKEMGQHVQ  NKAQEAKQYDISKTHDTTTKGHETTQRTAAA |
| 32 | >tr\|M4CYF6\|M4CYF6_BRARP Oleosin OS=Brassica rapa subsp. pekinensis OX=51351 PE=3 SV=1  MLSSLIQIFQVFQVTSAVVVTAVLFALAGITLAGSVVGLIVATPLFVIFSPVLVPATIAS  TLLATNLSAGALFGVTAAALIVWLFKHRMGVHPKNNPPPAGAPPTEADKPAEGTTEKPKD  NSTGGAADKPEDKPVGGAADKPEGKPDGGATNKPESKPAGGPSNKPKDKPAGGPTDKPES  KPADKPAGGPTDKPGSKPVDKPAGGPTDKTESKLVGEASNKPKDKPAGGSTDMPEAGETS  NKPKDKSVGGPTNKPESKPAGETSHKPKDKPAGGPTDKPESKPAGETSHKPKDKLAGGPT  DKPESKPAGEASNKPKDKPAGGPTDKLAGGSVDKPKDKPAGGPTDKPTNKLTGGAANKPA  GEAANKPTGKPKNKPAGENKPPGWYR |
| 33 | >tr\|M4C9X3\|M4C9X3_BRARP Oleosin OS=Brassica rapa subsp. pekinensis OX=51351 PE=3 SV=1  MADVRTHAHQVQVHPLRQQEGGIKVVYPQSGPSSTQVLAVIAGVPVGGTLLTLAGLTLAG  SVIGLMLAFPLFLIFSPVIVPAAFVIGLAMTGFMASGAIGLTGLSSMSWVLNHIRRVRER  MPDELEEAKQRLADMAEYVGQRTKDAGQTIEEKAHDVRESKTYDVRDRDTKGHTATGGDR  DTKTTREVRVATT |
| 34 | >tr\|M4DBK6\|M4DBK6_BRARP Oleosin OS=Brassica rapa subsp. pekinensis OX=51351 PE=3 SV=1  MTDTARTHHDITSRDQYPRDRDQYSMIGRDRDKYSMIGRDRDQYNMYGRDYSKSRQIAKA  VTAVTAGGSLLVLSSLTLVGTVIALTVATPLLVIFSPILVPALITVALLITGFLSSGGFG  IAAITVFSWIYKYATGEHPQGSDKLDSARMKLGGKVQDMKDRAQYYGQQHTGGYGQQQTG  GEHDRDRTRGTQHTT |
| 35 | >tr\|A0A251RPT8\|A0A251RPT8_HELAN Oleosin OS=Helianthus annuus OX=4232 GN=HannXRQ_Chr17g0548541 PE=3 SV=1  MATTTYDRHHVTTTQPQYRHDQHTGDRLTHPQHQQQGPSTGKIMVIMALLPITGILFGLA  GITLVGTVIGLALATPLFVIFSPVIVPAMIAIGLAVTGFLTSGTFGLTGLSSLSYLFNMV  RRSTMSVPDQMDYVKGKLQDVGEYTGQKTKDLGQKIQHTAHEMGDQGQGQGGGKEGRKEG  GK |
| 36 | >tr\|M4F3T3\|M4F3T3_BRARP Oleosin OS=Brassica rapa subsp. pekinensis OX=51351 PE=3 SV=1  MANQTRTHQDIIVRDSRSTLDRDHPKTGAQMVKVATGVAAGGSLLVLSGLTLAGTVIALA  VATPLLIIFSPVLVPAVITVVLIITGFLASGGFGIAAITAFSWLYRHMTGSGSDQKIESA  RMKVGSRGYDTKSGQHNIGVHQQHQQAAS |
| 37 | >tr\|F4K823\|F4K823_ARATH Oleosin OS=Arabidopsis thaliana OX=3702 GN=GRP16 PE=3 SV=1  MLSFLIPVVQFFQVVIAAVASVVFLVFAGITFGASIVGLTIATPLFVIFSPILVPATIAT  TFLVGGATAAVALGVTAFALILWLFKHRIGVKPKNNPAPKGAPTKADQPGASGGASGDKP  GEMSGAGGPSGDKPGGASGGGDKPGGASGGGPGGASGGASGGATWRSVGRRTWRSVGRRT  WRSVGRSFGR |
| 38 | >tr\|Q6WEQ9\|Q6WEQ9_ARALL Oleosin OS=Arabidopsis lyrata subsp. lyrata OX=81972 GN=GRP17 PE=3 SV=1  MSEELSQNPSSAQSLSLREDKNRFPFLSLSQREGRFFPSLSLSEREGRKFSFLSMFSFLM  PLLEVIKIIIASVASVVFLGFACVTLAGSAVALAVSTPVFIIFSPVLVPATIATVVLTTG  FTAGGSFGATALGLILWLIKRRMGVKAKDNPPPAGLPPNSGAEAGGAQSLIKKSKSKGGL  KLPAWCKKMLGGKFGGKKGKSGGGKGKFGGKGGMSEGEEGMSSGDDGMSGGEGGKSKRGG  HKSGSRGGKSKSKKGMSGGSESEEGMSGSEGGMSGGGGGKSKRGGGKRGGLRDKFGKKGG  MSGGGGGMSGSEGAMSGSEGGMSGGKSKSRGGKLGGLRGKYGKKGGKSGGGGGMSGSEGG  MSESEGGMSEGGMSGGGMSGGGMSGGGMSGGGGGKHNGKGKHGGLGGKFGKKGGMSERGG  GMSGSEGGMSGSEGGMSGGGISGGGGSKHKIGGGKHGGLGSKFGKKGGMSGGGGGMSGSE  GGMSGSEGGMSEGGMSGGGGGKHKIGGGKHKLGGGKLGSGGSHMAE |
| 39 | >tr\|A0A251SJI7\|A0A251SJI7_HELAN Oleosin OS=Helianthus annuus OX=4232 GN=OLEO6 PE=3 SV=1  MATYDRDRHPQQVQVHTVHDQRIDRFGDRSHYQNQQGPSKSKVLAVMALLPVGGALLGLA  GITLVGTMIGLAVATPLFVIFSPILVPAVLMIGLAVAGFLTSGTFGLTGLSSLSYLVNSL  RQITGTVPEQVDSAKRRLQDLVEYTGQKTKDVGQTIQDKAHDIGPEGAVHAGSAVGGAKE  GRGTKA |
| 40 | >tr\|A0A251UPB8\|A0A251UPB8_HELAN Oleosin OS=Helianthus annuus OX=4232 GN=OLEO1 PE=3 SV=1  MTDIHQTYNPQHHSLGGATHQQHVSPRVHQAVKAATAATAGGSLLVLSGLILAGTVIALT  IATPLLVIFSPVLVPAVITVFLIVTGFLTSGGFGVAAVTVLSWIYRYATGGHPPGADSLD  QARDKLGYKAREMKGRAEHATGMGQHHITGGGLRADM |
| 41 | >tr\|A0A251S7K5\|A0A251S7K5_HELAN Oleosin OS=Helianthus annuus OX=4232 GN=OLEO PE=3 SV=1  MATTTTYDRHFTTTQPHYRQDDRSRYDQQTHSQSTSRTLAIIALLPVGGILLGLAGLTFI  GTLIGLALATPLFVIFSPIIVPAVLTIGLAVTGFLASGTFGLTGLSSLSYLFNMVRQTAG  SVPESLDYVKGTLQDAGEYAGQKTKDFGQKIQNTAHEMGDQGQGGQVGVHAQVGGGKEGR  KSGDRT |
| 42 | >tr\|C3S7G3\|C3S7G3_BRANA Oleosin OS=Brassica napus OX=3708 GN=S3 PE=2 SV=1  MTDTARTHHDITTRDQYPMMGRDRDQYAIIGRDQYQGYGQDYSKSRQIAKAATAVTAGGS  LLVLSSLTLVGTVIALIVATPLLVIFSPILVPALITVALLITGFLSSGGFGIAAITVFSW  IYKYATGEHPKGSDKLDSARMKLGSKAQDMKDRAHYYGQQHTGGEHVNTDYRNTDRDRTR  GTT |
| 43 | >tr\|I1N5I8\|I1N5I8_SOYBN Oleosin OS=Glycine max OX=3847 GN=100794783 PE=3 SV=1  MAEVRSQQPQHVQVHASTTHTPQQYRYYQGGAKTQHHGGEGGGVMSLFPEISLTGSQLLA  LLAGVPLGGMLLLLSGVSLIASLVGLAVATPLFIFFSPVLVPAAFVIGMAVTAVLAAGAC  GLVGLVSFSWLVNCLRQMPRGTTTKTTMMRPEQAKRHVADMEEYVGKKTKDVGQDIQTRA  HAQGTTGMV |
| 44 | >tr\|D7LPJ0\|D7LPJ0_ARALL Oleosin OS=Arabidopsis lyrata subsp. lyrata OX=81972 GN=ARALYDRAFT_484554 PE=3 SV=1  MANVDRDRRVHVDRTDKRVHLQPSYEDDVGFGGYGAGSDFKSRGPSTNQILALIAGVPIG  GTLLTLAGLTLAGSVIGLLVSIPLFLLFSPVIVPAALTIGLAVTGILASGLFGLTGLSSV  SWVLNYLRGTSDTVPEQLDYAKRRMADAVGYAGMKGKEMGQYVQDKAHEARETEFTTETH  EPGKARRLIS |
| 45 | >tr\|Q5VKJ8\|Q5VKJ8_RICCO Oleosin OS=Ricinus communis OX=3988 GN=OLE2 PE=2 SV=1  MAEHQQSPVVSHRPRVNQLVKAGTAATAGSSLLFLSGLTLTGTVIALALATPLMVLFSPV  LLPAVIIISLIGAGFLTSGGFGFGAILVLSWIYRYVTGKQPPGAESLDQARLKLAGKARE  MKDRAEQFGQHVTGQQTS |
| 46 | >tr\|M4D1X4\|M4D1X4_BRARP Oleosin OS=Brassica rapa subsp. pekinensis OX=51351 PE=3 SV=1  MADTARTHHDITSRDQYPILGRDRDQYPYGRSDYQTSGQDYSKTRQIAKAATAVTAGGSL  LVLSSLTLVGTVIALTVATPLLVIFSPILVPALITVALLITGFLSSGGFGIAAITVFSWI  YKYATGEHPQGSDKLDSARMKLGTKAQDIKDRAQYYGQQHTGGEHDRDRTRGTHHTTTTT |
| 47 | >tr\|M4EW38\|M4EW38_BRARP Oleosin OS=Brassica rapa subsp. pekinensis OX=51351 PE=3 SV=1  MANVDRRVNVDRTDKGLQLQPQYEDRVGYGYGYGGNTDYKSRGPSTNQIVALIAGVPIGG  SLLALAGLTLAGSVIGFMLSIPLFLLFSPVIVPAALTIGLAVTGILASGLFGLTGLSSVS  WVLNYIRGRSDTVPEQLDYAKRRMADAVGYAGQKGKEMGQYVQDKAHEAHDTSLTTETNG  KTRRAHIA |
| 48 | >tr\|M4CYF9\|M4CYF9_BRARP Oleosin OS=Brassica rapa subsp. pekinensis OX=51351 PE=3 SV=1  MLSFFSRLLDVIKVVVASVTSVVLFLFAGLTLSGSAVALVVSTPLFLIFSPILVPATIAT  TLLASGLTAGATLGITAISLIMGLIKTAEGSSLARLAQTPLKLFKFSGGFGGSWGGKSFS  GTFGNKGSQSSGNIPGWLKNLLNGIPAGGAVPAAGEAAPAPAAGGAAPAPAAPPG |
| 49 | >tr\|A0A251UGX9\|A0A251UGX9_HELAN Oleosin OS=Helianthus annuus OX=4232 GN=HannXRQ_Chr07g0205601 PE=3 SV=1  MTDIHTKEQGQQQSRWHVEPQHQSQHWTTQSQRQWNEPRAHQVVKAATAAAVGGSLLVLA  GLVLAGTVIALTLATPVLVIFSPVLVPALIAVFLLVSGFLTSGGFGVAAATVLAWMYRYV  TGEQPSGADTSDEVSHRLGAKARDIKDRGEHAGRGGHYGTAGVHTGGPGGGVGTYV |
| 50 | >tr\|M4CYF7\|M4CYF7_BRARP Oleosin OS=Brassica rapa subsp. pekinensis OX=51351 PE=3 SV=1  MVVASVASVVYLGFAGVTLSGSAVALAVSTPLFIIFSPILLPAIAATTVLAAGLGGKKVA  AAPEASPAASPSLSLLGIPESIKPSNIIPESIKPSNIIPEGIKPSNIKDKIKDTIGKVKN  KIKAKKEEKSKGKSEDSSKGKGKSKGEDTTTDDDTTTDEDKHGSGAKHGKGESKHGKGES  THGKGGKHGSEGKHGSGGSSMGGGKHGSGGKHETGGKHGSGGKHESGGSAMGGGKHGSGG  KHGSEGKHGGEGSSMGKNSLSKKKKEFHYRGQAMDASSTSESSDGSSDGSSSDGSSHGSG  GKHI |
| 51 | >tr\|A0A087H670\|A0A087H670_ARAAL Oleosin OS=Arabis alpina OX=50452 GN=AALP_AA3G007200 PE=3 SV=1  MADVRTHSHQVQVHPLRQHEGGIKVVYPQSGTGTGTGTGPSSTQVLTVVAGVPVGGTLLL  LAGLTLAGSVIGTILAFPLFLIFSPVIVPAAFLIGLAMAGFFTSGAIGLTGLSSMSWVLN  FFRRARENLPEELEEAKQRLADMAEYVGQRTKDAGQTIEDRAQDVRETKTDVRGDVHTKG  HARGDRDTKTTHEVRVASS |
| 52 | >tr\|A0A251U5P5\|A0A251U5P5_HELAN Oleosin OS=Helianthus annuus OX=4232 GN=HannXRQ_Chr08g0223251 PE=3 SV=1  MTEIHQTYNPQHQPYGGGVSHYQQQQHVSPRVHQAVKAATAITAGGSLLILSGLTLAGTV  IALTIATPLLVIFSPVLVPAAITLFLLATGFLMSGGFGVAAATVLSWIYRYATGGHPLGS  DSLDQARDKLGYKARDMKGRAEHATGGMGLHARADM |
| 53 | >tr\|A0A3P5ZTB8\|A0A3P5ZTB8_BRACM Oleosin OS=Brassica campestris OX=3711 GN=BRAA03T12417Z PE=3 SV=1  MTKRSLYQWNTLLKSLSRDKQWQQVLSQFIQMFRCEEKPDNFTIPVALKACVELRQIKCG  EIIHAFINKDASLASDLYVGSALIDMYAKCGRMTQALRVFDELEEKPDIVTWSSMVSGFE  RNGFPFEAVEFFRRMATSYHVSPDRVTLITLVSACTKLSDSKLGRCVHGFVMRRGFEKDL  SLVNSLLNCYAKSGAFKEAVHLFKVMAEKDVISWSTVIACYVQNGAAAEALPYAVFSRIP  KKDVVSWVALISGFTLNGMAHRSVEEFSKMLLENNTRPDSILMVKVLKSCSDLGFLEQAE  CFHSYLIKFGFDSNPFIGASLVELYSRCGSLGSACKVFDEITLKDTVVWTSLITGYGIHG  KGTKALETFNQMVESSEVEPNDVTFLSVLSACSHSGLIGEGLRIFELMVSQYGLVPNLEH  YAVLVDLLGRVGELDTAIEITKRMPFSPTPQVLGTLLGACRIHQNDEMAETVAKKLFELE  PNHAGYYMLMSNMYGVKGEWENVEKLRNAVRNRGIKNGLAESLIKIKRKVHRFVADDNMH  PENEPVYGLLKELDLLMKQGLEDSAYFQTEGGSLCHVSPLRLGGLMADVRTHAHQVQVHP  LRQQEGGIKVVYPQSGPSSTQVLAVIAGVPVGGTLLTLAGLTLAGSVIGLMLAFPLFLIF  SPVIVPAAFVIGLAMTGFMASGAIGLTGLSSMSWVLNHIRRVRERMPDELEEAKQRLADM  AEYVGQRTKDAGQTIEEKAHDVRESKTYDVRDRDTKGHTATGGDRDTKTTREVRVATT |
| 54 | >tr\|A0A178VDK3\|A0A178VDK3_ARATH Oleosin OS=Arabidopsis thaliana OX=3702 GN=AXX17_At3g00700 PE=3 SV=1  MADVRTHSHQLQVHPQRQHEGGIKVLYPQSGPSSTQVLAVFVGVPIGGTLLTIAGLTLAG  SVIGLMLAFPLFLIFSPVIVPAAFVIGLAMTGFLASGAIGLTGLSSMSWVLNYIRRAGQH  IPEELEEAKHRLADMAEYVGQRTKDAGQTIEDKAHDVREAKTFDVRDRDTTKGTHNVRDT  KTT |
| 55 | >tr\|A0A0D3C252\|A0A0D3C252_BRAOL Oleosin OS=Brassica oleracea var. oleracea OX=109376 GN=106340888 PE=3 SV=1  MADLHQHQQPMTRNLHESSSSPSTRQTVRFLTAATIGMSLLVLSGLTLTGTVIGLVVATP  LMVLFSPVLVPAVITMCLLTAGFLFSGGCGVAAATALSWIYRYITGKHPMGADKVDYARM  MISDNAKELGHYAQPQTDQTTTAPY |
| 56 | >tr\|A0A0D3EH64\|A0A0D3EH64_BRAOL Oleosin OS=Brassica oleracea var. oleracea OX=109376 GN=106316754 PE=3 SV=1  MLSSLIQIFQVFQVTSAVVVTAVLFALAGITLAGSVVGLIVATPLFVIFSPVLVPATIAS  TLLATNLSAGALFGVTAAALIVWLLKHRMGVHPKNNPPPAGAPPTEAAKPTDKPAEGATD  KPKDNPTGGAADNPGGKSDGGETDKPESKPAGGPVNKPKDKPAGGPTDKPGSKPADKPAG  GPTDKPENKPAEEASNKPKDKPAGEPTDKPESKPSREASNKRKDKPAGGPTDKRESKPAG  EASNKPKDKPAGGPTTKPESKPAGEVSNKPKDKPVGGPTDKPGNKPAGGPADKPKDNPAG  GPTDKPADKPTGGTENKPAEEAANKPIGKPKNKPAGENKPPAWYS |
| 57 | >tr\|A0A0D3EH63\|A0A0D3EH63_BRAOL Oleosin OS=Brassica oleracea var. oleracea OX=109376 GN=106317860 PE=3 SV=1  MRNEIQNETAQTDQTQGSMFSFFNLFPFLLPMFEVIKMVVASVASVVYLGFAGVTLSGSA  VALAVSTPLFIIFSPILLPAIAATTVLAAGLGSKKVAAAPAASPSLSLLGIPESIKPSNV  IPESIKPSNIIPESIKPSNIIPVSIKPSNIKDKIKDTIGKVKNKIKAKQEEKSKGKSEDS  SKGKGKSKGEDTTTDEDKHGKGESKHGKGESKHGKGESTHGKGGKHGSEGSSMDEGKHGG  KHGSGGSPMGGGKHGSGGKHESGGSPMGGGKHGSGGKHESGGSPMGGGKHGSGGKHESGG  SPESVGKHGSGGKHESGGSPMGGGKHGSGGKHESGGASMGGGKHGSGGRHEGGGSAMGGG  KHGSGGKHGSEGKHGGEGSSMGKNSLSKNKKEFHYRGQAMDASSTSESSDGSSSDGSSSD  GSSSDGSSHGSGGKHI |
| 58 | >tr\|A0A0D3C071\|A0A0D3C071_BRAOL Oleosin OS=Brassica oleracea var. oleracea OX=109376 GN=106337122 PE=3 SV=1  MADTHRVDRTDRHLQFQSPYEGGRVNIQFEGAGEGYGQSGYGGGGGYGQSGYGGGGYKSM  MPESGPSSTQVISFLVGVPIVGSLLAIAGLLLAGSVIGLMISIPLFLLFSPVIVPAAITI  GLATTGFLASGMFGLTGLSSISWVMNYLRRTRGGVPDQLEYAKRRMADAVGYAGQKGKEM  GQFVQDKAHDAKQYDISKPQDTTTTTTTTTKGHETRTAAA |
| 59 | >tr\|A0A0D3A665\|A0A0D3A665_BRAOL Oleosin OS=Brassica oleracea var. oleracea OX=109376 GN=106298955 PE=3 SV=1  MTDTARTHHDITSRDQYPRDRDQYSMIGRDRDQYSMMGRDRDQYNMYGRDYSKSRQIAKA  VTAVTAGGSLLVLSSLTLVGTVIALTVATPLLVIFSPILVPALITVAMLITGFLSSGGFG  IAAITVFSWIYKYATGEHPQGSDKLDSARMKLGSKAQDLKDRAQYYGQQHTGGYGQQHTG  GEHDRDRTRGTQHTT |
| 60 | >tr\|A0A0D3DG02\|A0A0D3DG02_BRAOL Oleosin OS=Brassica oleracea var. oleracea OX=109376 GN=106304893 PE=3 SV=1  MTDTARTHHDITTRDQYPLISRDRDQYGMIGRDQYNMSGQNYSKSRQIAKATTAVTAGGS  LLVLSSLTLVGTVIALIVATPLLVIFSPILVPALITVALLITGFLSSGGFGIAAITVFSW  IYKYATGEHPQGSDKLDSARMKLGSKAQDMKDRAYYYGQQHTGEEHDRDRDHRTDRDRTR  GTQHTT |
| 61 | >tr\|A0A078H704\|A0A078H704_BRANA Oleosin OS=Brassica napus OX=3708 GN=BnaA01g14480D PE=3 SV=1  MTDTARTHHDITNRDKYSMIGRDRDQYNMYGRDYSKSRQIAKAVTAVTAGGSLLVLSSLT  LVGTVIALTVATPLLVIFSPILVPALITVALLITGFLSSGGFGIAAITVFSWIYKYATGE  HPQGSDKLDSARMKLGGKVQDMKDRAQYYGQQQTGGEHDRDRTRGTQHTT |
| 62 | >tr\|A0A078FBB9\|A0A078FBB9_BRANA Oleosin OS=Brassica napus OX=3708 GN=BnaC06g12930D PE=3 SV=1  MADTHRVDRTDRHLQFQSPYEGGRVSIQYEGGGGYGAEGYKSMMPERGPSSTQVLSFLVG  VPIVGSLLAIAGLLLAGSVIGLLISIPLFLLFSPVIVPAALTIGLAATGFLASGMFGLTG  LSSVSWVLNYLRGTRKSSVPEQLEYAKKRMADAVGYAGQKGKEMGQHVQNKAQEAKQYDI  SKTHDTTTKGHETTQRTAAA |
| 63 | >tr\|C3S7F4\|C3S7F4_BRANA Oleosin OS=Brassica napus OX=3708 GN=S4 PE=2 SV=1  MADTHRVDRTDRHLQFQPPYEGGRVNIQFEGAGEGYGQSGYGGGGGYGQSGYGGGGYKSM  MPESGPSSTQVISFLVGVPIVGSLLAIAGLLLAGSVIGLMISIPLFLLFSPVIVPAAITI  GLATTGFLASGMFGLTGLSSISWVMNYLRRTRGGVPDQLEYAKRRMADAVGYAGQKGKEM  GQFVQDKAHDAKQYDISKPQDTTTTTTTTTKGHETRTAAA |
| 64 | >tr\|C3S7F2\|C3S7F2_BRANA Oleosin OS=Brassica napus OX=3708 GN=S4 PE=2 SV=1  MADTHRVDRTDRHLQFQSPYEGGRVSIQYEGGGGAGGYGGRGGGYGAEGYKSMMPERGPS  STQVLSFLVGVPIVGSLLAIAGLLLAGSVIGLLISIPLFLLFSPVIVPAALTIGLAATGF  LASGMFGLTGLSSVSWVLNYLRGTRKSSVPEQLEYAKKRMADAVGYAGQKGKGMGQHVQN  KAQEAKQYDISKTHDTTTKGHETTQRTAAA |
| 65 | >tr\|C3S7G1\|C3S7G1_BRANA Oleosin OS=Brassica napus OX=3708 GN=S3 PE=2 SV=1  MTDTARTHHDITSRDQYPRDRDQYSMIGRDRDKYSMIGRDRDQYNMYGRDYSKSRQIAKA  VTAVTAGGSLLVLSSLTLVGTVIALTVATPLLVIFSPILVPALITVALLITGFLSSGGFG  IAAITVFSWIYKYATGEHPQGSDKLDSARMKLGGKVQDMKDRAQYYGQQQTGGEHDRDRT  RGTQHTT |
| 66 | >tr\|A0A078IM60\|A0A078IM60_BRANA Oleosin OS=Brassica napus OX=3708 GN=BnaC04g32530D PE=3 SV=1  MADTHRVDRTDRHLQFQSPYEGGRVNIQFEGAGEGYGQSGYGGGGGYGQSGYGGGGYKSM  MPEIYIYSLTFMHTKLMRFFLGPNKTFPIVGSLLAIAGLLLAGSVIGLMISIPLFLLFSP  VIVPAAITIGLATTGFLASGMFGLTGLSSISWVMNYLRRTRGGVPDQLEYAKRRMADAVG  YAGQKGKEMGQFVQDKAHDAKQYDISKPQDTTTTTTTTTKGHETRTAAA |
| 67 | >tr\|A0A078J3L4\|A0A078J3L4_BRANA Oleosin (Fragment) OS=Brassica napus OX=3708 GN=BnaCnng36370D PE=3 SV=1  MLSSLIQIFQVFQVTSAVVVTAVLFALAGITLAGSVVGLIVATPLFVIFSPVLVPATIAS  TLLATNLSAGALFGVTAAALIVWLLKHRMGVHPKNNPPPAGAPPTEAAKPTDKPAEGATD  KPKDNPTGGAADNPGGKSDGGETDKPESKPAGGPVNKPKDKPAGGPTDKPGSKPADKPAG  GPTDKPENKPAEEASNKPKDKPAGEPTDKPESKPSREASNKRKDKPAGGP |
| 68 | >tr\|Q9LY07\|Q9LY07_ARATH Glycine-rich protein 20 OS=Arabidopsis thaliana OX=3702 GN=ATGRP20 PE=2 SV=1  MAPFPLSLIFGKKKRRRDDEIRRQKPTLKGVMTAFFATEAAICLLLLAGISLTGTAVALF  ASMPLFLVFSPVLVPAGIATTILASGLMAGGTSGVSGLTILMWLYKKYTGRDFPIKIPGA  AAAGGAAPAAPAAPAPAAPAAKPAAKPAAKPGA |
| 69 | >tr\|A0A078JR26\|A0A078JR26_BRANA Oleosin (Fragment) OS=Brassica napus OX=3708 GN=BnaCnng60890D PE=3 SV=1  MRNEIQNETAQTDQTQGSMFSFFNLFPFLLPMFEVIKMVVASVASVVYLGFAGVTLSGSA  VALAVSTPLFIIFSPILLPAIAATTVLAAGLGSKKVAAAPAASPSLSLLGIPESIKPSNV  IPESIKPSNIIPESIKPSNIIPVSIKPSNIKDKIKDTIGKVKNKIKAKQEEKSKGKSEDS  SKGKGKSKGEDTTTDEDKHGKGESKHGKGESKHGKGESTHGKGGKHGSEGKHGSGGSPMG  GGKHGSGGKHESGGSPM |
| 70 | >tr\|C3S7F3\|C3S7F3_BRANA Oleosin OS=Brassica napus OX=3708 GN=S4 PE=2 SV=1  MADTHRVDRTDRHLQFQSPYEGGRVNIQYEGGGGAGGYGGGRGGGYGAGGYKSMMPERGP  SNTQVLSFLVGVPIVGSLLAIAGLLLAGSVIGLLISIPLFLLFSPVIVPAALTIGLAATG  FLASGMFGLTGLSSVSWVMNYLRGTRKSSVPEQLEYAKKRMADAVGYAGQKGKEMGQHVQ  NKAHEAKQYDISKTHDTTTTKGHETTQRTAAA |
| 71 | >tr\|C3S7E9\|C3S7E9_BRANA Oleosin OS=Brassica napus OX=3708 GN=S1 PE=2 SV=1  MADVRTHAHQVQVHPLRQQEGGIKVVYPQSGPSSTQVLAVIAGVPVGGTLLTLAGLTLAG  SVIGLMLAFPLFLIFSPVIVPAAFVIGLAMTGFMASGAIGLTGLSSMSWVLNHIRRVRER  MPDELEEAKQRLADMAEYVGQRTKDAGQTIEEKAHDVRESKTYDVRDRDTKGHTATGGDR  DTKTTREVRVATT |
| 72 | >tr\|Q42640\|Q42640_BRAOL Oleosin OS=Brassica oleracea OX=3712 GN=GRP3 PE=2 SV=1  MRNEIQNETAQTDQTQGSMFSFFDLFPFLLPMFEVIKMVVASVASVVYLGFAGVTLSGSA  VALAVSTPLFIIFSPILLPAIAATTVLAAGLGSKKVAAAPAASPSLSLLGIPESIKPSNV  IPESIKPSNIIPESIKPSNIIPESVKPSNIKDKIKDTIGKVKNKINAKKEEKSKGKSEDS  SKGKGKSKGEDTTTDEDKPGSGGKHGKGESKHGKGESTHGKGGKHGSEGSSMDEGKHGGK  HGSGGSPMGGGKHGSGGKHESGGSPMGGGKHGSGGKHESGGASMGGGKHGSGGKHGSEGK  HGGEGSSMGKNSQSKNKKEFHYRGQAMDASSTSESSDGSSDGSSDGSSSDGSSHGSGGKH  I |
| 73 | >tr\|C3S7G2\|C3S7G2_BRANA Oleosin OS=Brassica napus OX=3708 GN=S3 PE=2 SV=1  MTDTARTHHDITTRDQYPMMGRDRDQYAIIGRDQYQGYGQDYSKSRQIAKAATAVTAGGS  LLVLSSLTLVGTVIALIVATPLLVIFSPILVPALITVALLITGFLSSGGFGIAAITVFSW  IYKYATGEHPKGSDKLDSARMKPGSKAQDMKDRAHYYGQQHTGGEHVNTDYRNTDRDRTR  GTT |
| 74 | >tr\|C3S7G0\|C3S7G0_BRANA Oleosin OS=Brassica napus OX=3708 GN=S3 PE=2 SV=1  MTDTARTHHDITSRDQYPRDRDQYSTIGRDRDKYSMIGRDRDQYNMYGRDYSKSRQIAKA  VTAVTAGGSLLVLSSLTLVGTVIALTVATPLLVIFSPILVPALITVALLITGFLSSGGFG  IAAITVFSWIYKYATGEHPQGSDKLDSARMKLGGKVQDMKDRAQYYGQQQTGGEHDRDRT  RGTQHTT |
| 75 | >tr\|Q65ZR4\|Q65ZR4_BRANA Oleosin (Fragment) OS=Brassica napus OX=3708 GN=oleosin napII PE=2 SV=1  RRDQYPRDRDQYSMIGRDRDKYSMIGRDRDQYNMYGRDYSKSRQIAKAVTAVTAGGSLLV  LSSLTLVGTVIALTVATPLLVIFSPILVPALITVALLITGFLSSGGFGIAAITVFSWIYK  YATGEHPQGSDKLDSARMKLGGKVQDMKDRAQYYGQQQTGGEDDRDRTRGTQHTT |
| 76 | >tr\|C3S7G6\|C3S7G6_BRANA Oleosin OS=Brassica napus OX=3708 GN=S3 PE=2 SV=1  MTDTARTHHDITTRDQYPLISRDRDQYGMIGRDQYNMSGQNYSKSRQIAKATTAVTAGGS  LLVLSSLTLVGTVIASIVATPLLVIFSPILVPALITVALLITGFLSSGGFGIAAITVFSW  IYKYATGEHPQGSDKLDSARMKLGSKAQDMKDRAYYYGQQHTGEEHDRDRDHRTDRDRTR  GTQHTT |
| 77 | >tr\|A8MSB7\|A8MSB7_ARATH Oleosin OS=Arabidopsis thaliana OX=3702 GN=GRP19 PE=3 SV=1  MFEIIQAVFSAGVALALLTFAGITLGGSVVACIISTPLFVIFSPVLVPATIATTLLASGF  TASGSFGATAFTILSWLYKEGPTKNSRIDATGSGV |
| 78 | >tr\|C3S7G4\|C3S7G4_BRANA Oleosin OS=Brassica napus OX=3708 GN=S3 PE=2 SV=1  MTDTARTHHDITSRDQYPRDRDQYSMIGRDRDQYSMMGRDRDQYNMYGRDYSKSRQIAKA  VTAVTAGGSLLVLSSLTLVGTVIALTVATPLLVIFSPILVPALITVAMLITGFLSSGGFG  IAAITVFSWIYKYATGEHPQGSDKLDSARMKLGSKAQDLKDRAQYYGQQHTGGYGQQHTG  GEHDRDRTRGTQHTT |
| 79 | >tr\|Q94CI2\|Q94CI2_BRAOL Oleosin OS=Brassica oleracea OX=3712 GN=GRP2 PE=3 SV=1  MLSSLIQIFQVFQVTSAVVVTAVLFALAGITLAGSVVGLIVATPLFVIFSPVLVPATIAS  TLLATNLSAGALFGVTAAALIVWLLKHRMGVHPKNNPPPAGAPPTEAAKPTDEPAEGATD  KPKDNPTGGAADKPGDKPAGGAADNSGGKSDGGETDKPESKPAGGPVNKPKDKPAGGPTD  KPGSKPADKPAGGPTDKPESKPAGDASNKPKDKPTGEPTDKPESKPAREASNKRKDKPAG  GPTDKRESKPAGEVSHKPKDKSAGGPTTKPESKPAGEVSHKPKDKSAGGPTDKPGNKPVG  GPADKPKDNPAGRPTDKPTGGTENKPAGEAANKPIGKPKNKPAGENKPPAWYS |
| 80 | >tr\|A0A251RWY6\|A0A251RWY6_HELAN Oleosin OS=Helianthus annuus OX=4232 GN=HannXRQ_Chr16g0504661 PE=3 SV=1  MAEHHHQQQHHPINTNTHFHQNNVYSTSKLLAVLTLFPIGGLCFLLSGLILTGTLIGLTI  ATPVFFIFSPVLLPAILTVGLMVTGFLTSVAFGITALSSLAYIVSYFRRTSGWRRKRRGY  TTGYLGQKVKDMGLKTQETDWA |
| 81 | >tr\|A0A0R0JP17\|A0A0R0JP17_SOYBN Oleosin OS=Glycine max OX=3847 GN=GLYMA_05G004300 PE=3 SV=1  MANLKLVPHANPQLSILHARVNTPKLHISMKHHRHYFNPTLRSQQQQPQHVQVHATTTHT  PQHHGGEALLAGVPLGAMLLLLSGISLIASLLGLAVATPLFIFFSPLLVPAAFAIGMAVT  AVLAAGACGLAGLVLFSWVVNYLRQMPRGTTMMTVLPEQAKRHVADMAEYVGQKTKEVGQ  DIQTRAHHAQGTTRMTVH |
| 82 | >tr\|M4EIT0\|M4EIT0_BRARP Oleosin OS=Brassica rapa subsp. pekinensis OX=51351 PE=3 SV=1  MLSFLISLLDIIKLVIASATFLFFLAFSGLTFAGSAVALVVSTPLFIIFSPILVPATIST  TLLASGLAAGTSLGLSAIGLIMRLIKYY |
| 83 | >tr\|M4CYF8\|M4CYF8_BRARP Oleosin OS=Brassica rapa subsp. pekinensis OX=51351 PE=3 SV=1  MSEELVQHESHSQASIFSRFFRMFSFIFPLLNVIKLIIASVTSLVCLAFSCVALGGSAVA  LIVSTPLFIMFSPILVPATIATTLLASGLMAGTTLGLTGIGLIMGLVR |
| 84 | >tr\|M4CYF4\|M4CYF4_BRARP Oleosin OS=Brassica rapa subsp. pekinensis OX=51351 PE=3 SV=1  MFFQIIQGVFTGVEALALLAFAGITLGGSAVGLALSTPLFILFSPILVPATIATTLLTTG  FTTSGGLGIVALRIFWKLFK |
| 85 | >tr\|A0A178V1V3\|A0A178V1V3_ARATH Oleosin OS=Arabidopsis thaliana OX=3702 GN=AXX17_At4g29040 PE=3 SV=1  MADTARGTHHDIIGRDQYPMMGRDRDQYQMSGRGSDYSKSRQIAKAATAVTAGGSLLVLS  SLTLVGTVIALTVATPLLVIFSPILVPALITVALLITGFLSSGGFGIAAITVFSWIYKYA  TGEHPQGSDKLDSARMKLGSKAQDLKDRAQYYGQQHTGGEHDRDRTRGGQHTT |
| 86 | >tr\|Q5HZ52\|Q5HZ52_ARATH Oleosin OS=Arabidopsis thaliana OX=3702 GN=C24_LOCUS25198 PE=2 SV=1  MADQTRTHHEMISRDSTQEAHPKARQMVKAATAVTAGGSLLVLSGLTLAGTVIALTVATP  LLVIFSPVLVPAVVTVALIITGFLASGGFGIAAITAFSWLYRHMTGSGSDKIENARMKVG  SRVQDTKYGQHNIGVQHQQVS |
| 87 | >tr\|A0A384LN43\|A0A384LN43_ARATH Oleosin OS=Arabidopsis thaliana OX=3702 GN=AXX17_At5g50000 PE=3 SV=1  MADQTRTHHEMISRDSTQEAHPKARQMVKAATAVTAGGSLLVLSGLTLAGTVIALTVATP  LLVIFSPVLVPAVVTVALIITGFLASGGFGIAAITAFSWLYRHMTGSGSDKIENARMKVG  SRVQDTKYGQHNIGVQHQQVS |
| 88 | >tr\|D7L9N5\|D7L9N5_ARALL Oleosin OS=Arabidopsis lyrata subsp. lyrata OX=81972 GN=ARALYDRAFT_477358 PE=3 SV=1  MADVRTHSHQLQVHPQRQHEGGIKVLYPQSGPSSTQVLAVFVGVPVGGTLLTIAGLTLAG  SVIGLMLAFPLFLIFSPVIVPAAFVIGLAMTGFLASGAIGLTGLSSMSWVLNYIRWAGEH  IPEELEEAKQRLADMAEYVGQRTKDAGQTIEDKAHDVRETKTFDVRDRDTTKGTHNVRDT  KTT |
| 89 | >tr\|A0A0D3B993\|A0A0D3B993_BRAOL Oleosin OS=Brassica oleracea var. oleracea OX=109376 PE=3 SV=1  MTKRSLYQWNTLLKSLSRDKQWQQVLSQFIQMFRCEEKPDNFTIPVALKACVELRQIKCG  EIIHAFINKDASLASDLYVGSALIDMYAKCGRMTQALRVFDELEEKPDIVTWSSMVSGFE  RNGFPFEAVEFFRRMATSSHVSPDRVTLITLVSACTKLSDSKLGRCVHGFVMRRGFEKDL  SLVNSLLNCYAKSGAFKEAVHLFKVMAEKDVISWSTVIACYVQNGAAAEALRVFNEMMGS  GTEPSAATMLSVFQACAASHDLEQGRKSHELAIRKGIETEVKVSTALVDMYMKCFSPEEA  YAVFSRIPKKDVVSWVALISGFTLNGMAHRSVEEFSKMLLENNTRPDSILMVKVLKACSD  LGFLEQAECFHSYVIKFGFDSNPFIGASLVELYSRCGSLGSACKVFDEITLKDVVVWTSL  ITGYGIHGKGTKALETFTQMVESSEVEPNEVTFLSVLSACSHSGLIHEGLRIFELMVSQY  GLVPNLEHYAVLVDLLGRVGELDAAIEITKGMPFSPTPQVLGTLLGACRIHQNDEMAETV  AKKLFELEPNHAGYYMLMSNMYGVKGEWENVEKLRNAVRNRGIKKGLAESLIEIKRKVHS  FVADDNMHPENEPVYGLLKELDLHMKQDFEDSVYFQTEGGSYYLTKLYLGGLMADVRTHA  HQVQVHPLRQHEGGIKVVYPQSGPSSTQVLAVVAGVPVGGTLLTLAGLTLAGSVIGLILA  FPLFLIFSPVIVPAAFVIGLAMTGFMASGAIGLTGLSSMSWVLNHIRRVRERIPDELDEA  KQRLADMAEYVGQRTKDAGQTIEDKAHDVRESKTYDVRDRDTKGHTASGGDRDTKTTREV  RVATT |
| 90 | >tr\|Q94FQ4\|Q94FQ4_ARATH Oleosin OS=Arabidopsis thaliana OX=3702 PE=3 SV=1  MSEELSQKPSSAQSLSLREGRNRFPFLSLSQREGRFFPSLSLSERDGRKFSFLSMFSFLM  PLLEVIKIIIASVASVIFVGFACVTLAGSAAALVVSTPVFIIFSPVLVPATIATVVLATG  FTAGGSFGATALGLIMWLVKRRMGVKPKDNPPPAGLPPNSGAGAGGAQSLIKKSKAKSKG  GLKAWCKKMLKSKFGGKKGKSGGGKSKFGGKGGKSEGEEGMSSGDERMSGSEGGMSGGEG  GKSKSGKGKLKAKLEKKKGMSGGSESEEGMSGSEGGMSGGGGSKSKSKKSKLKAKLGKKK  GMSGGISGSEEGMSGSEGGMSSGGGSKSKSKKSKLKAKLGKKNCMSGGMSGSEEGMSGSE  GGMSGGGGGKSKSRKSKLKAKLGKKKSMSGGMSGSEGGMSGSEGGMSGGGMSGGSGSKHK  IGGGKHGGLGGKFGKKRGMSGSGGGMSGSEGGMSGSEGSMSGGGMSRGSGSKHKIGGGKH  GGLRGKFGKKRGMSGSEGSMSGSEGGMSESGMSGSGGGKHKIGGGKHKFGGGKHGGGGGH  MAE |
| 91 | >tr\|Q94FQ0\|Q94FQ0_ARATH Oleosin OS=Arabidopsis thaliana OX=3702 PE=3 SV=1  MSEELSQKPSSAQSLSLREGRNRFPFLSLSQREGRFFPSLSLSERDGRKFSFLSMFSFLM  PLLEVIKIIIASVASVIFVGFACVTLAGSAAALVVSTPVFIIFSPVLVPATIATVVLATG  FTAGGSFGATALGLIMWLVKRRMGVKPKDNPPPAGLPPNSGAGAGGAQSLIKKSKAKSKG  GLKAWCKKMLKSKFGGKKGKSGGGKSKFGGKGGKSEGEEGMSSGDEGMSGSEGGMSGGEG  GKSKSGKGKLKAKLEKKKGMSGGSESEEGMSGSEGGMSGGGGSKSKSKKSKLKAKLGKKK  GMSGGMSGSEEGMFGSEGGMSSGGGSKSKSKKSKLKAKLGKKKGMSGGMSGSEEGMSGSE  GGMSGGGGGKSKSRKSKLKAKLGKKKCMYGGMSGSEGGMSGSEGGISGGGMSGGSGSKHK  IGGGKHGGLGGKFGKKRGMSGSGGGMSGSEGGVSGSEGSMSGGGMSGGSGSKHKIGGGKH  GGLRGKFGKKRGMSGSEGGMSGSEGGVSESGMSGSGGGKHKIGGGKHKFGGGKHGGGGGH  MAE |
| 92 | >tr\|Q9FUJ9\|Q9FUJ9_SESIN Oleosin OS=Sesamum indicum OX=4182 PE=2 SV=1  MADRDRPHPHQIQVHPQHPHRYEGGVKSLLPQKGPSTTQILAIITLLPISGTLLCLAGIT  LVGTLIGLAVATPVFVIFSPVLVPAAILIAGAVTAFLTSGAFGLTGLSSLSWVLNSFRRA  TGQGPLEYAKRGVQEGTLYVGEKTKQAGEAIKSTAKEGGREGTART |
| 93 | >tr\|Q94FP9\|Q94FP9_ARATH Oleosin OS=Arabidopsis thaliana OX=3702 PE=3 SV=1  MSEELSQKPSSAQSLSLREGRNRFPFLSLSQREGRFFPSLSLSERDGRKFSFLSMFSFLM  PLLEVIKIIIASVASVIFVGFACVTLAGSAAALVVSTPVFIIFSPVLVPATIATVVLATG  FTAGGSFGATALGLIMWLVKRRMGVKPKDNPPPAGLPPNSGAGAGGAQSLIKKSKAKSKG  GLKAWCKKMLKSKFGGKKGKSGGGKSKFGGKGGKSEGEEGMSSGDEGMSGSEGGMSGGEG  GKSKSGKGKLKAKLEKKKGMSGGSESEEGMSGSEGGMSGGGGSKSKSKKSKLKAKLGKKK  CMSGGMSGSEEGMSGSEGGMSSGGGSKSKSKKSKLKAKLGKKKGMSGGMSGSEEGMSGSE  GGMSGGGGGKSKSRKSKLKAKLEKKKGMSGGMSGIEGGMSGSEGGISGGGMSGGSGSKHK  IGGGKHGGLGGKFGKKRGMSGSGGGMSGSEGGVSGSEGSMSGGGMSGGSGSKHKIGGGKH  GGLRGKFEKKRGMSGSEGGMSGSEGGMSESGMSGSGGGKHKIGGGKHKFGGGKHGGGGGH  MAE |
| 94 | >tr\|A0A0D3CT24\|A0A0D3CT24_BRAOL Oleosin OS=Brassica oleracea var. oleracea OX=109376 PE=3 SV=1  MADTHRVDRTDRHLQFQSPYEGGRVSIQYEGGGGAGGYGGRGGGYGAEGYKSMMPERGPS  STQVTFFFTFSMLIHFLNFMHSILNLLDSIFRYGVILFGGGPYRWCLAGSVIGLLISIPL  FLLFSPVIVPAALTIGLAATGFLASGMFGLTGLSSVSWVLNYLRGTRKSSVPEQLEYAKK  RMADAVGYAGQKGKEMGQHVQNKAQEAKQYDISKTHDTTTKGHETTQRTAAA |
| 95 | >tr\|A0A0D3DMT4\|A0A0D3DMT4_BRAOL Oleosin OS=Brassica oleracea var. oleracea OX=109376 PE=3 SV=1  MADTARTHHDITSRDQYPLLGRDRDQYPYGRSDYQTSSQDYSKTRQIAKAATAVTAGGSL  LVLSSLTLVGTVIALTVATPLLVIFSPILVPALITVALLITGFLSSGGFGIAAITVFSWI  YKYATGEHPQGSDKLDSARMKLGSKAQDIKDRAQYYGQQHTGGEPDRDRTRGTHHNTT |
|  | >tr\|A0A397ZZI8\|A0A397ZZI8_BRACM Oleosin OS=Brassica campestris OX=3711 GN=BRARA_C02941 PE=3 SV=1  MADVRTHAHQVQVHPLRQQEGGIKVVYPQSGPSSTQVLAVIAGVPVGGTLLTLAGLTLAG  SVIGLMLAFPLFLIFSPVIVPAAFVIGLAMTGFMASGAIGLTGLSSMSWVLNHIRRVRER  MPDELEEAKQRLADMAEYVGQRTKDAGQTIEEKAHDVRESKTYDVRDRDTKGHTATGGDR  DTKTTREVRVATT |
| 96 | >tr\|A0A397YL15\|A0A397YL15_BRACM Oleosin OS=Brassica campestris OX=3711 GN=BRARA_G01479 PE=3 SV=1  MADTHRVDRTDRHLQFQSPYEGGRVNIQYEGGGGAGGYGGGRGGGYGAGGYKSMMPERGP  SSTQVLSFLVGVPIVGSLLAIAGLLLAGSVIGLLISIPLFLLFSPVIVPAALTIGLAATG  FLASGMFGLTGLSSVSWVMNYLRGTRKSSVPEQLEYAKKRMADAVGYAGQKGKEMGQHVQ  NKAQEAKQYDISKTHDTTTKGHETTQRTAAA |
| 97 | >tr\|A0A087GGK1\|A0A087GGK1_ARAAL Oleosin OS=Arabis alpina OX=50452 GN=AALP_AA7G076700 PE=3 SV=1  MADTARTTHHDILNRDRDQYGRDQFGRDQYGRDQYGRDQYGRDQYGQMSSRGDYSKSRQI  AKAATAVTAGGSLLVLSSLTLVGTVIALTVATPLLVIFSPILVPALITVALLITGFLSSG  GFGIAAITVFSWIYKYATGEHPQGSDRLDSARMKLGSKAQDLKDRAHYYGQQHTGGEHDR  DRDRTTGRVGQQTS |
| 98 | >tr\|A0A087GA32\|A0A087GA32_ARAAL Oleosin OS=Arabis alpina OX=50452 GN=AALP_AA8G285900 PE=3 SV=1  MANQTRTHHEIISRDSRTRDHPKARQMVKAATAVTAGGSLLVLSGLTLAGTVIALTVATP  LLVIFSPVLVPAVITVVLIITGFLASGGFGIAAITAFSWLYRHLTGSGSERLENARMKLG  SRSQDTKYGQHNTGVQHHQQQQAS |
| 99 | >tr\|A0A0R0FZF4\|A0A0R0FZF4_SOYBN Oleosin OS=Glycine max OX=3847 GN=GLYMA_16G071800 PE=3 SV=1  MTTVPPHSVQVHTTTHRYEAGVVPPARFEAPRYEAGIKAPSSIYHSERGPTTSQVLAVVA  GLPVGGILLLLAGLTLAGTLTGLVVATPLFIIFSPVLIPATVAIGLAVAGFLTSGVFGLT  ALSSFSWILNYIRETQPASENLAAAAKHHLAEAAEYVGQKTKEVGQKTKEVGQDIQSKAQ  DTREAAARDARDAREAAARDARDAKVEARDVKRTTVTATTATA |
| 100 | >tr\|A0A3P6D7W5\|A0A3P6D7W5_BRACM Oleosin OS=Brassica campestris OX=3711 GN=BRASC35T46007Z PE=3 SV=1  MANQTRTHQDIIVRDSRSTLDRDHPKTGAQMVKVATGVAAGGSLLVLSGLTLAGTVIALA  VATPLLIIFSPVLVPAVITVVLIITGFLASGGFGIAAITAFSWLYRHMTGSGSDQKIESA  RMKVGSRGYDTKSGQHNIGVHQQHQQAAS |
| 101 | >tr\|A0A087G5I9\|A0A087G5I9_ARAAL Oleosin OS=Arabis alpina OX=50452 GN=AALP_AA8G071100 PE=3 SV=1  MREELAQTGSQPQSTQSMREGRLFSFLETFSFLMPLLDLIKLVIASVASVALLGFAGLTL  AGSAVGFLISTPLFIIFSPILVPATVVTTLLATGLTAGSSIGATALGLIIWLIKHRMGVK  PGNNPIPKGAPPTGIRERIRKRIKGGGAGAKPEEGAKPEEGAKPEGASPPPSFLSFFQLP  DLKKIPEKIKSKFSNKSKGGEGGSMGGEGGSMGEGGNSGGEGKSGGKGKSRGEGSRGEGK  SRGLGKSRGEGSMGGEGKSRSGGKSKFGKKIKSKLGKKFGHGGSPEDGSSSGGGSSHGGG  SSHGGSSPRGGNSHGGEGSMGGEGKSRSGGKSKFGEKIKSKLGSKFGHGGSHGGGSSPGG  GSSHGGGSSHGGSSPGGXXXXXXXXXXXXXXXXXXXXXXXXXXXXSSPGGGSSHGSVGKS  NFRENIRRKFQSKFHSKFGRGGLHGGSSSPGGGSSHGGGSSHGDSSPGGGSSHGGSSGGS  SPRGGGGRSGMDGGSHME |
| 102 | >tr\|A0A087G5I8\|A0A087G5I8_ARAAL Oleosin OS=Arabis alpina OX=50452 GN=AALP_AA8G071000 PE=3 SV=1  MREELAQTGSQPQSTQSMQEGRLFSFLKTFSFIMPLLDLIKLVIASVASVALLGFAGLTL  AGSAVGFLISTPLFIIFSPILVPATVVTTLLATGLIAGSSIGATALGLIIWLIKHRMGVK  PGNNPIPKGAPPTGIRERIRKRIKGGGAGAKPEEGAKPEEGAKQEGASPPPAPSFFQIPS  FMQLAAQGVKNKFSQKPMGGGGGSMVEGGSMGEGGSMGDGGKPIGEGKSKGEGKSKSKGK  GKGKGKCKCEGKSKDKSKGKGKGKCKCEGKSKCGGKSKGGEGKSKRGGKSKRGSSPGGGS  SPGGGSSPGGGSSPGGGSSPGGGSSPGGGTSPGGGSSPGGGDMGGGSDME |
| 103 | >tr\|A0A3P6DBL6\|A0A3P6DBL6_BRACM Oleosin OS=Brassica campestris OX=3711 GN=BRAA10T44973Z PE=3 SV=1  MRNEIQNETAQTDQTQGSMFSFFNLFPFLLPMFEVIKMVVASVASVVYLGFAGVTLSGSA  VALAVSTPLFIIFSPILLPAIAATTVLAAGLGGKKVAAAPEASPAASPSLSLLGIPESIK  PSNIIPESIKPSNIIPEGIKPSNIKDKIKDTIGKVKNKIKAKKEEKSKGKSEDSSKGKGK  SKGEDTTTDDDTTTDEDKHGSGAKHGKGESKHGKGESTHGKGGKHGSEGKHGSGGSSMGG  GKHGSGGKHETGGKHGSGGKHESGGSAMGGGKHGSGGKHGSEGKHGGEGSSMGKNSLSKK  KKEFHYRGQAMDASSTSESSDGSSDGSSSDGSSHGSGGKHI |
| 104 | >tr\|Q84WT0\|Q84WT0_ARATH Oleosin OS=Arabidopsis thaliana OX=3702 GN=At5g51210 PE=2 SV=1  MADQTRTHHEMISRDSTQEAHPKARQMVKAATAVTAGGSLLVLSGLTLAGTVVALTVATP  LLVIFSPVLVPAVVTVALIITGFLASGGFGIAAITAFSWLYRHMTGSGSDKIENARMKVG  SRVQDTKYGQHNIGVQHQQVS |
| 105 | >tr\|Q42638\|Q42638_BRAOL Oleosin (Fragment) OS=Brassica oleracea OX=3712 PE=2 SV=1  EIQNETAQTDQTQGSMFSFFDLFPFLLPMFEVIKMVVASVASVVYLGFAGVTLSGSAVAL  AVSTPLFIIFSPILLPAIAATTVLAAGLGSKKVAAAPAASPSLSLLGIPESIKPSNVIPE  SIKPSNIIPESIKPSNIIPESVKPSNIKDKIKDTIGKVKNKINAKKEEKSKGKSEDSSKG  KGKSKGEDTTTDEDKPGSGGKHGKGESKHGKGESTHGKGGKHGSEGSSMDEGKHGGKHGS  GGSPMGGGKHGSGGKHESGGSPMGGGKHGSGGKHESGGASMGGGKHESVGKHGSGGKHES  GGSPMSGGKHGSGGKHESGGASMGGGKHGSGGRHEGGGSAMGGGKHGSGGKHGSEGKHGG  EGSSMGKNSLSKNKKEFHYRDQAMDASSTSESSDGSSDGSSSDGSSSDGSSHGSGGKHI |
| 106 | >tr\|Q42639\|Q42639_BRAOL Oleosin (Fragment) OS=Brassica oleracea OX=3712 PE=2 SV=1  TDQTQGSMFSFFDLFPFLLPMFEVIKMVVASVASVVYLGFAGVTLSGSAVALAVSTPLFI  IFSPILLPAIAATTVLAAGLGSKKVAAAPAASPSLSLLGIPESIKPSNVIPESIKPSNII  PESIKPSNIIPESVKPSNIKDKIKDTIGKVKNKINAKKEEKSKGKSEDSSKGKGKSKGED  TTTDEDKPGSGGKHGKGESKHGKGESTHGKGGKHGSEGSSMDEGKHGSGGKHESGGASMG  GGKHGSGGRHEGGGSAMGGGKHGSGGKHGSEGKHGGEGSSMGKNSLSKNKKEFHYRDQAM  DASSTSESSDGSSDGSSSDGSSSDGSSHGSGGKHI |
| 107 | >tr\|Q42637\|Q42637_BRAOL Oleosin OS=Brassica oleracea OX=3712 PE=2 SV=1  MRNEIQNETAQTDQTQGSMFSFFDLFPFLLPMFEVIKMVVASVASVVYLGFAGVTLSGSA  VALAVSTPLFIIFSPILLPAIAATTVLAAGLGSKKVAAAPAASPSLSLLGIPESIKPSNV  IPESIKPSNIIPESIKPSNIIPESVKPSNIKDKIKDTIGKVKNKINAKKEEKSKGKSEDS  SKGKGKSKGEDTTTDEDKPGSGGKHGKGESKHGKGESTHGKGGKHGSEGSSMDEGKHGGK  HGSGGSPMGVGKHGSGGKHESGGSPMGGGKHGSGGKHESGGASMGGGKHGSGGRHEGGGS  AMGGGKHGSGGKHGSEGKHGGEGSSMGKNSLSKNKKEFHYRDQAMDASSTSESSDGSSDG  SSSDGSSSDGSSHGSGGKHI |
| 108 | >tr\|A0A087G5I7\|A0A087G5I7_ARAAL Oleosin OS=Arabis alpina OX=50452 GN=AALP_AA8G070900 PE=3 SV=1  MVIASVASLALLGFAGLTLAGSTVGLVVSTPLFIIFSPVLVPATIAITLLATGLTAGASL  GGMALGLIMWLIKSSASYKGRKAPSALLPFFGTFVDKPPAPPTAGTTPPGGATPPSEAAP  ASGAAPAGGNSPSNGFKWSWSWGGKPFFGGAPSGGSNPVGGLKWNWSWGGKSYSGTIPFG  GKSAGGSTPTGGTEPATGSPPAD |
| 109 | >tr\|Q94FR0\|Q94FR0_ARATH Oleosin OS=Arabidopsis thaliana OX=3702 PE=3 SV=1  MSEELSQKPSSAQSLSLREGRNRFPFLSLSQREGRFFSSLSLSERDGRKFSFLSMFSFLM  PLLEVIKIIIASVASVIFVGFACVTLAGSAAALVVSTPVFIIFSPVLVPATIATVVLATG  FTAGGSFGVTALGLIMWLVKRRMGVKPKDNPPPAGLPPNSGAGAGGAQSLIKKSKAKSKG  GLKAWCKKMLKSKFGGKKGKSGGGKSKFGGKGGKSEGEEGMSSGDEGMSGSEGGMSGGEG  GKSKSGKGKLKAKLEKKKGMSGGSESEEGMSGSEGGMSGGGGSKSKSKKSKLKAKLGKKK  GMSGGMSGSEEGMSGSEGGMSSGGGSKSKSKKSKLKAKLGKKKGMSGGMSGSEEGMSGSE  GGMSGGGGGKSKSRKSKLKAKLGKKKCMSGGMSGSEGGMSGSEGGISGGGMSGGSGSKHK  IGGGKHGGLGGKFGKKRGMSGSGGGMSGSEGGVSGSEGSMSGGGMSGGSGSKHKIGGGKH  GGLRGKFGKKRGMSGSEGGMSGSEGGMSESGMSGSGGGKHKIGGGKHKFGGGKHGGGGGH  MAE |
| 110 | >tr\|A0A178UIH4\|A0A178UIH4_ARATH Oleosin OS=Arabidopsis thaliana OX=3702 GN=AXX17_At5g07220 PE=3 SV=1  MFSFLMPLLEVIKIIIASVASVIFVGFACVTLAGSAAALVVSTPVFIIFSPVLVPATIAT  VVLATGFTAGGSFGVTALGLIMWLVKRRMGVKPKDNPPPAGLPPNSGAGAGGAQSLIKKS  KAKSKGGLKAWCKKMLKSKFGGKKGKSGGGKSKFGGKGGKSEGEEGMSSGDEGMSGSEGG  MSGGEGGKSKSGKGKLKAKLEKKKGMSGGSESEEGMSGSEGGMSGGGGSKSKSKKSKLKA  KLGKKKGMSGGMSGSEEGMSGSEGGMSGGGGGKSKSRKSKLKAKLGKKKCMSGGMSGSEG  GMSGSEGGISGGGMSGGSGSKHKIGGGKHGGLGGKFGKKRGMSGSGGGMSGSEGGVSGSE  GSMSGGGMSGGSGSKHKIGGGKHGGLRGKFGKKRGMSGSEGGMSGSEGGMSESGMSGSGG  GKHKIGGGKHKFGGGKHGGGGGHMAE |
| 111 | >tr\|Q6J1J8\|Q6J1J8_ARAHY Oleosin OS=Arachis hypogaea OX=3818 GN=Ahy_A05g023475 PE=2 SV=1  MATATDRAPHQVQVHTPTTQRVDVPRRGYDVSGGGIKTLLPERGPSTSQIIAVLVGVPTG  GTLLLLSGLSLLGTIIGLAIATPVFIFFSPVIVPAVVTIGLAVTGILTAGACGLTGLMSL  SWMINFIRQVHGTTVPDQLDSVKRRMADMADYVGQKTKDAGQEIQTKAQDVKRSSS |
| 112 | >tr\|A0A398AP76\|A0A398AP76_BRACM Oleosin OS=Brassica campestris OX=3711 GN=BRARA_A01514 PE=3 SV=1  MTDTARTHHDITSRDQYPRDRDQYSMIGRDRDKYSMIGRDRDQYNMYGRDYSKSRQIAKA  VTAVTAGGSLLVLSSLTLVGTVIALTVATPLLVIFSPILVPALITVALLITGFLSSGGFG  IAAITVFSWIYKYATGEHPQGSDKLDSARMKLGGKVQDMKDRAQYYGQQQTGGEHDRDRT  RGTQHTT |
| 113 | >tr\|A0A397ZJS4\|A0A397ZJS4_BRACM Oleosin OS=Brassica campestris OX=3711 GN=BRARA_D01064 PE=3 SV=1  MADTHRVDRTDRHLQFQSPYEGGRVNIQFEGAGGGYGQSGYGDGYGQSGYGGGGYKSMMP  ESGPSSTQVISFLVGVPLVGSLLAIAGLLLAGSVIGLMISIPLFLLFSPVIVPAAITIGL  ATTGFLTSGMFGLTGLSSISWVMNYLRRTRGSVPDQLEYAKRRMADAVGYAGQKGKEVGQ  FVQDKAHDAKQYDISKPHDTTTTTTTTTKGLETRTAAA |
| 114 | >tr\|Q49L62\|Q49L62_BRACM Oleosin (Fragment) OS=Brassica campestris OX=3711 PE=2 SV=1  PRDRDQYSMIGRDRDKYSMIGRDRDQYNMYGRDYSKSRQIAKAVTAVTAGGSLLVLSSLT  LVGTVIALTVATPLLVIFSPILVPALITVALLITGFLSSGGFGIAAITVFSWIYKYATGE  HPQGSDKLDSARMKLGGKVQDMKDRAQYYGQQHTGGYGQQQTGGEHDRDRTRGTQHTT |
| 115 | >tr\|A0A445EPH4\|A0A445EPH4_ARAHY Oleosin OS=Arachis hypogaea OX=3818 GN=Ahy_A01g001806 PE=3 SV=1  MADIQQYYQQDHQDVSATKERSSSHILALATLLPFGASLLFLAGVTLLATLIGVALATPL  FIIFSPILIPAALVIAFSVSGFLTSGAFGVTSVSSFAWMASYLRRSRLQESLLHAKDRAQ  QIMSNMAQRAKEAGATVVSEAQDTAQQAHDAPTDTDSTVSVTPSESQTSDTQSGSGSGSG  GGTQTRDTASTTETKDEGGGGGKSKDRKKTSS |
| 116 | >tr\|A0A445AU69\|A0A445AU69_ARAHY Oleosin OS=Arachis hypogaea OX=3818 GN=Ahy_B01g054716 PE=3 SV=1  MADIQQYYQQDHQDVSATKERSSSHILALATLLPFGASLLFLAGVTLLATLIGVALATPL  FIIFSPILIPAALVIAFSVSGFLTSGAFGVTSVSSFAWMASYFRRSRLQESLLHAKDRAQ  QIMSNMAQRAKEAGDTVVSKAQDTAQQAHDAPTDTDSTISVTPSESQTSDTQSGSGSGGG  TQTRDTASTTETKDEGGGGGKSKDRKKTSS |
| 117 | >tr\|A0A397KW15\|A0A397KW15_BRACM Oleosin OS=Brassica campestris OX=3711 GN=BRAA03T14647Z PE=3 SV=1  MTDTARTHHDITTRDQYPMMGRDRDQYAIIGRDQYQGYGQDYSKSRQIAKAATAVTAGGS  LLVLSSLTLVGTVIALIVATPLLVIFSPILVPALITVALLITGFLSSGGFGIAAITVFSW  IYKYATGEHPKGSDKLDSARMKLGSKAQDMKDRAHYYGQQHTGGEHVNTDYRNTDRDRTR  GTT |
| 118 | >tr\|O82308\|O82308_ARATH Oleosin OS=Arabidopsis thaliana OX=3702 GN=At2g25890 PE=3 SV=1  MVSLLKLQKQHRTLNPYSLRKRKKEMADHQQHQQQQQPIMRSLHESSPSTRQIVRFVTAA  TIGLSLLVLSGLTLTGTVIGLIVATPLMVLFSPVLVPAVITIGLLTMGFLFSGGCGVAAA  TALTWIYKYVTGKHPMGADKVDYARMRIAEKAKELGHYTHSQPQQTHQTTTTTH |
| 119 | >tr\|Q2F3K0\|Q2F3K0_BRANA Oleosin OS=Brassica napus OX=3708 PE=3 SV=1  MTDTARTHHDITSRDQYPRDRDQYSMIGRDRDKYSMIGRDRDQYNMYGRDYSKSRQIAKA  VTAVTAGGSLLVLSSLTLVGTVIALTVATPLLVIFSPILVPALITVALLITGFLSSGGFG  IAAVTVFSWIYKYATGEHPQGSDKLDSARMKLGGKVQDMKDRAQYYGQQQTGGEHDRDRT  RGTQHTT |
| 120 | >tr\|A0A445KI27\|A0A445KI27_GLYSO Oleosin OS=Glycine soja OX=3848 GN=D0Y65_010876 PE=3 SV=1  MAEVRSQQQQPQHVQVHATTTHTPQHHGGEGGSGILNLIPEMSLTGSQLLALLAGVPLGA  MLLLLSGISLIASLLGLAVATPLFIFFSPLLVPAAFAIGMAVTAVLAAGACGLAGLVLFS  WVVNYLRQMPRGTTMMTVLPEQAKRHVADMAEYVGQKTKEVGQDIQTRAHHAQGTTRMTV  H |
| 121 | >tr\|Q6V5K1\|Q6V5K1_BRAOL Oleosin OS=Brassica oleracea OX=3712 GN=Bo_7530 PE=3 SV=1  MRNEIQNETAQTDQTQGSMFSFFDLFPFLLPMFEVIKMVVASVASVVYLGFAGVTLSGSA  VALAVSTPLFIIFSPILLPAIAATTVLAAGLGSKKVAAAPAASPSLSLLGIPESIKPSNV  IPESIKPSNIIPESIKPSNIIPESVKPSNIKDKIKDTIGKVKNKINAKKEEKSKGKSEDS  SKGKGKSKGEDTTTDEDKPGSGGKHGKGESKHGKGESTHGKGGKHGSEGSSMDEGKHGGK  HGSGGSPMGGGKHGSGGKHESGGASMGGGKHGSGGRHESGSSAMGGGKHGSGGKHGSEGK  HGGEGSSMGKNSQSKNKKEFHYRGQAMDASSTSESSDGSSDGSSDGSSSDGSSHGSGGKH  I |
| 122 | >tr\|A0A3N6Q709\|A0A3N6Q709_BRACR Oleosin OS=Brassica cretica OX=69181 GN=DY000_00001052 PE=3 SV=1  MTDTARTHHDVTSRDQYPRDRDQYSMIGRDRDQYSMMGRDRDQYNMYGRDYSKSRQIAKA  VTAVTAGGSLLVLSSLTLVGTVIALTVATPLLVIFSPILVPALITVAMLITGFLSSGGFG  IAAITVFSWIYKYATGEHPQGTDKLDSARMKLGSKAQDLKDRAQYYGQQHTGGEHDRDRT  RGTQHTT |
| 123 | >tr\|A0A3N6PVU1\|A0A3N6PVU1_BRACR Oleosin OS=Brassica cretica OX=69181 GN=DY000_00003179 PE=3 SV=1  MADLHQHQQPMTRNLHESSSSPSTRQTVRFLTAATIGMSLLVLSGLTLTGTVIGLVVATP  LMVLFSPVLVPAVITMCLLTAGFLFSGGCGVAAATALSWIYRYVTGKHPMGADKVDYARV  MISDKAKELGHYAQPQTDQTTTAPY |
| 124 | >tr\|A0A3N6R1B3\|A0A3N6R1B3_BRACR Oleosin OS=Brassica cretica OX=69181 GN=DY000_00015073 PE=3 SV=1  MADTHRVDRTDRHLQFQSPYEGGRVNIQFEGAGEGYGQSGYGGGGGYGQSGYGGGGYKSM  MPESGPSSTQVISFLVGVPIVGSLLAIAGLLLAGSVIGLMISIPLFLLFSPVIVPAAITI  GLATTGFLTSGMFGLTGLSSISWVMNYLRRTRGSVPDQLEYAKRRMADAVGYAGQKGKEM  GQFVQDKAHDAKQYDISKPHDTTTTTTTTTKGHETRTAAA |
| 125 | >tr\|A0A3N6S1Y1\|A0A3N6S1Y1_BRACR Oleosin OS=Brassica cretica OX=69181 GN=DY000_00029368 PE=3 SV=1  MADTHRVDRTDRHLQFQSPYEGGRVNIQYEGGGGAGGYGGRGGGYGAKGYKSIMPERGPS  STQVLFFLVGVPIVGSLLAIAGLLLAGSVIGLLISIPLFLLFSPVIVPAALTIGLAATGF  LASGMFGLTGLSSVSWVLNYLPGTRKSSVPEQLEYAKKRMADAVGYAGQKGKEMGQHVQN  KAQEAKQYDISKTHDTTTKGHETTQRTAAA |
| 126 | >tr\|A0A5E4GEY2\|A0A5E4GEY2_PRUDU Oleosin OS=Prunus dulcis OX=3755 GN=ALMOND_2B024808 PE=3 SV=1  MAEQHPRPQEHQGYQTQHQYDQQQQHQGFQYDDQQQPKGFLPQNGPSATHIVAMLTLVPI  GGTLLFLSGVTLAGTILGLAVSTPLFVIFSPILVPAALVIGLSVVGILTSGAFGITALSS  FSWLARFLRRSRLPEKMGQKVQETTGYLGLKVQETAGYLGQKMQETGGQVGHLLQETGGQ  VGQKTRETGQNLDKAQDAGRDQEGGRTKEGGRGREGVTVTVEP |
| 127 | >tr\|A0A445FCW9\|A0A445FCW9_GLYSO Oleosin OS=Glycine soja OX=3848 GN=D0Y65_050659 PE=3 SV=1  MTTQVPPHSVQVHTTTHRYEAGVVPPGARFEPPRYEAGVKAPSIYHSERGPTTSQVLAVL  AGLPVGGILLLLAGLTLAGTLTGLAVATPLFVLFSPVLVPATVAIGLAVAGFLTSGAFGL  TALSSFSWILNYIRETQPASENLAAAAKHHLAEAAEYVGQKTKEVGQKTKEVGQDIQSKA  QDTREAAARDAREAAARDAREAAARDAKVEARDVKRTTVTATTATA |
| 128 | >tr\|A0A445GFF8\|A0A445GFF8_GLYSO Oleosin OS=Glycine soja OX=3848 GN=D0Y65_042947 PE=3 SV=1  MTTVPPHSVQVHTTTHRYEAGVVPPARFEAPRYEAGIKAPSSIYHSERGPTTSQVLAVVA  GLPVGGILLLLAGLTLAGTLTGLVVATPLFIIFSPVLIPATVAIGLAVAGFLTSGVFGLT  ALSSFSWILNYIRETQPASENLAAAAKHHLAEAAEYVGQKTKEVGQKTKEVGQDIQSKAQ  DTREAAARDARDAREAAARDARDAKVEARDVKRTTVTATTATA |
| 129 | >tr\|A0A3P6CEU7\|A0A3P6CEU7_BRACM Oleosin OS=Brassica campestris OX=3711 GN=BRAA04T17003Z PE=3 SV=1  MADTHRVDRTDRHLQFQSPYEGGRVNIQFEGAGGGYGQSGYGGGGGYGQSGYGGGGYKSM  MPESGPSSTQVISFLVGVPLVGSLLAIAGLLLAGSVIGLMISIPLFLLFSPVIVPAAITI  GLATTGFLTSGMFGLTGLSSISWVMNYLRRTRGSVPDQLEYAKRRVADAVGYAGQKGKEV  GQFVQDKAHDAKQYDISKPHDTTTTTTTTTKGLETRTAAA |
| 130 | >tr\|A0A3P6CQ91\|A0A3P6CQ91_BRACM Oleosin OS=Brassica campestris OX=3711 GN=BRAA10T44974Z PE=3 SV=1  MLSSLIQNFQVFQVTSAVVVTAVLFALAGITLAGSVVGLIVATPLFVIFSPVLVPATIAS  TLLATNLSAGALFGVTAAALIVWLFKHRMGVHPKNNPPPAGAPPTEADKPAEGTIDKPKD  NSTGGAADKPGDKPAGGAADKPGGKPDGGATDKPESKPTKGPSNKPKDKPDGRPTDKPRS  KPADKLAGGPTYNPESKPVGEASNKPKDKPAGGPTDNPESKPVEEASNKPKDKPAGGSTD  TPEAGKTSNKPKDKPAGRPTDKPESKPAGEASNKPKDKPVGGPTDKQENKPAEGSADKPK  DKPAEGPTDKPTGGAANKPAGEAANKPTGKPKNKPAGENKPPAWYR |
| 131 | >tr\|A0A3P6BRG2\|A0A3P6BRG2_BRACM Oleosin OS=Brassica campestris OX=3711 GN=BRAA07T29727Z PE=3 SV=1  MADTHRVDRTDRHLQFQSPYEGGRVNIQYEGGGGAGGYGGRGGGYSAGGYKSMMPERGPS  STQVLSFLVGVPIVGSLLAIAGLLLAGSVIGLLISIPLFLLFSPVIVPAALTIGLAATGF  LASGMFGLTGLSSVSWVMNYLRGTRKSSVPEQLEYAKKRMADAVGYAGQKGKEMGQHVQN  KAHEAKQYDISKTHDTTTKGHETTQRTAAA |
| 132 | >tr\|A0A3P6FKT4\|A0A3P6FKT4_BRAOL Oleosin OS=Brassica oleracea OX=3712 GN=BOLC1T01985H PE=3 SV=1  MTDTARTHHDVTSRDQYPRDRDQYSMIGRDRDQYSMMGRDRDQYNMYGRDYSKSRQIAKA  VTAVTAGGSLLVLSSLTLVGTVIALTVATPLLVIFSPILVPALITVAMLITGFLSSGGFG  IAAITVFSWIYKYATGEHPQGSDKLDSARMKLGSKAQDLKDRAQYYGQQHTGGYGQQHTG  GEHDRDRTRGTQHTT |
| 133 | >tr\|A0A3P6E7Z2\|A0A3P6E7Z2_BRAOL Oleosin OS=Brassica oleracea OX=3712 GN=BOLC7T45893H PE=3 SV=1  MTDTARTHHDITTRDQYPLISRDRDQYGMIGRDQYNMSGQNYSKSRQIAKATTAVTAGGS  LLVLSSLTLVGTVIALIVATPLLVIFSPILVPALITVALLITGFLSSGGFGIAAITVFSW  IYKYATGEHPQGSDKLDSARMKLGSKAQDMKDRAYYYGQQHTGEEHDRDRDHRTDRDRTR  GTQHTT |
| 134 | >tr\|A0A3P6CHK0\|A0A3P6CHK0_BRAOL Oleosin (Fragment) OS=Brassica oleracea OX=3712 GN=BOLC4T27103H PE=3 SV=1  HKSQNSINKQTEKEKMADLHQHQQPMTRNLHESSSSPSTRQTVRFLTAATIGMSLLVLSG  LTLTGTVIGLVVATPLMVLFSPVLVPAVITMCLLTAGFLFSGGCGVAAATALSWIYRYIT  GKHPMGADKVDYARMMISDNAKELGHYAQPQTDQTTTAPY |
| 135 | >tr\|A0A3P6DS72\|A0A3P6DS72_BRAOL Oleosin OS=Brassica oleracea OX=3712 GN=BOLC9T59710H PE=3 SV=1  MLSSLIQIFQVFQVTSAVVVTAVLFALAGITLAGSVVGLIVATPLFVIFSPVLVPATIAS  TLLATNLSAGALFGVTAAALIVWLLKHRMGVHPKNNPPPAGAPPTEAAKPTDKPAEGATD  KPKDNPTGGAADNPGGKSDGGETDKPESKPAGGPVNKPKDKPAGGPTDKPGSKPADKPAG  GPTDKPENKPAEEASNKPKDKPAGEPTDKPESKPSREASNKRKDKPAGGPTDKRESKPAG  EASNKPKDKPAGGPTTKPESKPAGEVSNKPKDKPVGGPTDKPGNKPAGGPADKPKDNPAG  GPTDKPADKPTGGTENKPAEEAANKPIGKPKNKPAGENKPPAWYS |
| 136 | >tr\|A0A3P6E9Q1\|A0A3P6E9Q1_BRAOL Oleosin OS=Brassica oleracea OX=3712 GN=BOLC9T59709H PE=3 SV=1  MRNEIQNETAQTDQTQGSMFSFFNLFPFLLPMFEVIKMVVASVASVVYLGFAGVTLSGSA  VALAVSTPLFIIFSPILLPAIAATTVLAAGLGSKKVAAAPAASPSLSLLGIPESIKPSNV  IPESIKPSNIIPESIKPSNIIPVSIKPSNIKDKIKDTIGKVKNKIKAKQEEKSKGKSEDS  SKGKGKSKGEDTTTDEDKHGKGESKHGKGESKHGKGESTHGKGGKHGSEGSSMDEGKHGG  KHGSGGSPMGGGKHGSGGKHESGGSPMGGGKHGSGGKHESGGASMGGGKHESVGKHGSGG  KHESGGSPMGGGKHGSGGKHESGGASMGGGKHGSGGRHEGGGSAMGGGKHGSGGKHGSEG  KHGGEGSSMGKNSLSKNKKEFHYRGQAMDASSTSESSDGSSSDGSSSDGSSSDGSSHGSG  GKHI |
| 137 | >tr\|A0A3P6C0K5\|A0A3P6C0K5_BRAOL Oleosin OS=Brassica oleracea OX=3712 GN=BOLC4T26321H PE=3 SV=1  MADTHRVDRTDRHLQFQSPYEGGRVNIQFEGAGEGYGQSGYGGGGGYGQSGYGGGGYKSM  MPESGPSSTQVISFLVGVPIVGSLLAIAGLLLAGSVIGLMISIPLFLLFSPVIVPAAITI  GLATTGFLASGMFGLTGLSSISWVMNYLRRTRGGVPDQLEYAKRRMADAVGYAGQKGKEM  GQFVQDKAHDAKQYDISKPQDTTTTTTTTTKGHETRTAAA |
| 138 | >tr\|A0A3P6H0L1\|A0A3P6H0L1_BRAOL Oleosin OS=Brassica oleracea OX=3712 GN=BOLC6T37105H PE=3 SV=1  MADTHRVDRTDRHLQFQSPYEGGRVSIQYEGGGGAGGYGGRGGGYGAEGYKSMMPERGPS  STQVTFFFTFSMLIHFLNFMHSILNLLDSIFRYGSVILFGGGPYPGLLLAGSVIGLLISI  PLFLLFSPVIVPAALTIGLAATGFLASGMFGLTGLSSVSWVLNYLRGTRKSSVPEQLEYA  KKRMADAVGYAGQKGKEMGQHVQNKAQEAKQYDISKTHDTTTKGHETTQRTAAA |
| 139 | >tr\|A0A3T0QHI0\|A0A3T0QHI0_COCNU Oleosin OS=Cocos nucifera OX=13894 PE=2 SV=1  MAERRPEGLKGFLSEKGPSKSQVLAVVAVFPIGGLLLILSGVTLTASVIGLAVATPLFIL  FSPVLVPAAIAIGLAVTGFLTSGAFGVTGLSSLSCFVEDARRLASKASEQLEQARQRMTE  AGGQLGKQTKEATQGTQGRT |
| 140 | >tr\|A0A3P5Z5E2\|A0A3P5Z5E2_BRACM Oleosin OS=Brassica campestris OX=3711 GN=BRAA01T01594Z PE=3 SV=1  MTDTARTHHDITSRDQYPRDRDQYSMIGRDRDKYSMIGRDRDQYNMYGRDYSKSRQIAKA  VTAVTAGGSLLVLSSLTLVGTVIALIVATPLLVIFSPILVPALITVALLITGFLSSGGFG  IAAITVFSWIYKYATGEHPQGSDKLDSARMKLGGKVQDMKDRAQYYGQQQTGGEHDRDRT  RGTQHTT |
| 141 | >tr\|Q6WEQ8\|Q6WEQ8_ARALL Oleosin OS=Arabidopsis lyrata subsp. lyrata OX=81972 GN=GRP16 PE=3 SV=1  MFSFLIPVVQLFQVVIAGAASVVFLLFAGITFGASIVGLTIATPLFVIFSPILVPATIAT  TLVVGGATATVALGVTAFALIFWLFKHRIGVKPKNNPAPKGAPTKADQPGASEGASGDKP  GEMSGAGGLSGDKSGGAPGDKRGEMSGDKPGGASGGGPGGASGGVSGGAPGGASGGPGGA  SGGEPGGASGGAPGGASGGASGDKPGGASGDKPGETPGHKPAGARGGKRLAWW |
| 142 | >tr\|A0A0D3AW96\|A0A0D3AW96_BRAOL Oleosin OS=Brassica oleracea var. oleracea OX=109376 GN=106325704 PE=3 SV=1  MTTVDRRVNVDRTDKGLQLKPQYEDRVGYGAGYDYGYGADYKSRGPSTNQVVALIAGVPI  GGSLLALAGLTLAGSVIGLMLSIPLFLLFSPVIVPAALTIGLAVTGILASGLFGLTGLSS  VSWVLNYIRGTSDTVPEQLDYAKRRMADAVGYAGQKGKEMGQYVQDKAHEAHDTSLTPET  REPVQAKRTLA |
| 143 | >tr\|A0A078GHK4\|A0A078GHK4_BRANA Oleosin OS=Brassica napus OX=3708 GN=BnaA08g14540D PE=3 SV=1  MADTARTHHDITSRDQYPILGRDRDQYPYGRSDYQTSGQDYSKTRQIAKAATAVTAGGSL  LVLSSLTLVGTVIALTVATPLLVIFSPILVPALITVALLITGFLSSGGFGIAAITVFSWI  YKYATGEHPQGSDKLDSARMKLGTKAQDIKDRAQYYGQQHTGGEHDRDRTRGTHHTTTTT |
| 144 | >tr\|A0A078HC36\|A0A078HC36_BRANA Oleosin OS=Brassica napus OX=3708 GN=BnaA04g15210D PE=3 SV=1  MADLHQQHQQPMTRNLHESSSSPSTRQTVRFLTAATIGMSLLVLSGLTLTGTVIGLVVAT  PLMVLFSPVLVPAVITMCLLTAGFLFSGGCGVAAATALSWIYRYVTGKHPMGADKVDYAR  MMISEKAKELGHYAQPQTDQTTTAPY |
| 145 | >tr\|I1JUP4\|I1JUP4_SOYBN Oleosin OS=Glycine max OX=3847 GN=100301903 PE=3 SV=1  MATISDQPRGSYSYGTSYGAPYGTTYETNTSINNPPSRQTVKFITAATIGITLLLLSGLT  LTGTVIGLIIATPLLVIFSPILVPAAFVLFLVASGFLFSGGCGVAAIAALSWIYNYVSGN  QPAGYDTLDYAKGYLADKARDVKERAKDYGSYAQGREGSRFVCKVAKH |
| 146 | >tr\|A0A0D3EH65\|A0A0D3EH65_BRAOL Oleosin OS=Brassica oleracea var. oleracea OX=109376 GN=106317110 PE=3 SV=1  MFFQIIQGVFTGVEALALLAFAGITLGGSAVGLALSTPLFILFSPILVPATIATTLLTTG  FTTSGGLGMVALRIFWKLFKRLRKKGKGTPKIPGLAPGAPDSDPVSGG |
| 147 | >tr\|A0A0D3EH61\|A0A0D3EH61_BRAOL Oleosin OS=Brassica oleracea var. oleracea OX=109376 GN=106316427 PE=3 SV=1  MFSFLSPLLDVIKVVVASVTSVVLFVFAGLTLSGSAVALVVSTPFFLIFSPILVPATIAT  TLLASGVTAGATLGITAISLIMGLIKTAEGSSLARLAQTPLKLFKFSGGFGGSWGGKPFS  GTFGNKGSQSSGNIPGWLKNLLNGIPGGGAAPAAGGAAPAPAAPAPAAPPG |
| 148 | >tr\|C3S7G5\|C3S7G5_BRANA Oleosin OS=Brassica napus OX=3708 GN=S3 PE=2 SV=1  MTDTARTHHDITTRDQYPLISRDRDQYGMIGRDQYNMSGQNYSKSRQIAKATTAVTAGGS  LLVLSSLTLVGTVIALIVATPLLVIFSPILVPALITVALLITGFLSSGGFGIAAITVFSW  IYKYATGEHPQGSDKLDSARMKLGSKAQDMKDRAYYYGQQHTGEEHDRDRDHRTDRDRTR  GTQHTT |
| 149 | >tr\|A0A078JFK5\|A0A078JFK5_BRANA Oleosin OS=Brassica napus OX=3708 GN=BnaC09g47930D PE=3 SV=1  MFSFLSPLLDVIKVVVASVTSVVLFVFAGLTLSGSAVALVVSTPFFLIFSPILVPATIAT  TLLASGVTAGATLGITAISLIMGLIKTAEGSSLARLAQTPLKLFKFSGGFGGSWGGKPFS  GTFGNKGSQSSGNIPGWLKNLLNGIPGGGAAPAAGGAAPAPAAPAPAAPPG |
| 150 | >tr\|M4EZN7\|M4EZN7_BRARP Oleosin OS=Brassica rapa subsp. pekinensis OX=51351 PE=3 SV=1  MADLHQQHQQPMTRNLHESSSSPSTRQTVRFLTAATIGMSLLVLSGLTLTGTVIGLVVAT  PLMVLFSPVLVPAVITMCLLTAGFLFSGGCGVAAATALSWIYRYVTGKHPMGADKVDYAR  MMISEKAKELGHYAQPQTDQTTTAPY |
| 151 | >tr\|A0A078JDY8\|A0A078JDY8_BRANA Oleosin OS=Brassica napus OX=3708 GN=BnaA10g30190D PE=3 SV=1  MFFQIIQGVFTGVEALALLAFAGITLGGSAVGLALSTPLFILFSPILVPATIATTLLTTG  FTTSGGLGIVALRIFWKLFKRLRKKGKGTPKIPGLAPGAPDSNSVSGG |
| 152 | >tr\|A0A078IIH2\|A0A078IIH2_BRANA Oleosin OS=Brassica napus OX=3708 GN=BnaA02g29000D PE=3 SV=1  MANVDRRVNVDRTDKGLQLQPQYEDRVGYGYGYGGNTDYKSRGPSTNQIVALIAGVPIGG  SLLALAGLTLAGSVIGFMLSIPLFLLFSPVIVPAALTIGLAVTGILASGLFGLTGLSSVS  WVLNYIRGRSDTVPEQLDYAKRRMADAVGYAGQKGKEMGQYVQDKAHEAHDTSLTTETNG  KTRRAHIA |
| 153 | >tr\|C3S7F7\|C3S7F7_BRANA Oleosin OS=Brassica napus OX=3708 GN=S5 PE=2 SV=1  MANQTRTHQDIIVRDSRSTLDRDHPKTGAQMVKVATGVAAGGSLLVLSGLTLAGTVIALA  VATPLLIIFSPVLVPAVITVVLIITGFLASGGFGIAAITAFSWLYRHMTGSGSDQKIESA  RMKVGSRGYDTKSGQHNIGVHQQHQQAAS |
| 154 | >tr\|A0A078J6X3\|A0A078J6X3_BRANA Oleosin OS=Brassica napus OX=3708 GN=BnaCnng36380D PE=3 SV=1  MFFQIIQGVFTGVEALALLAFAGITLGGSAVGLALSTPLFILFSPILVPATIATTLLTTG  FTTSGGLGMVALRIFWKLFKRLRKKGKGTPKIPGLAPGAPDSDPVSGG |
| 155 | >tr\|C3S7E8\|C3S7E8_BRANA Oleosin OS=Brassica napus OX=3708 GN=S2 PE=2 SV=1  MANVDRRVNVDRTDKGLQLQPQYEDRVGYGYGYGGNTDYKSCGPSTNQIVALIAGVPIGG  SLLALAGLTLAGSVIGFMLSIPLFLLFSPVIVPAALTIGLAVTGILASGLFGLTGLSSVS  WVLNYIRGRSDTVPEQLDYAKRRMADAVGYAGQKGKEMGQYVQDKAHEAHDTSLTTETNG  KTRRAHIA |
| 156 | >tr\|C3S7F9\|C3S7F9_BRANA Oleosin OS=Brassica napus OX=3708 GN=S3 PE=2 SV=1  MADTARTHHDITSRDQYPILGRDRDQYPYGRSDYQTSGQDYSKTRQIAKAATAVTAGGSL  LVLSSLTLVGTVIALTVATPLLVIFSPILVPALITVALLITGSLSSGGFGIAAITVFSWI  YKYATGEHPQGSDKLDSARMKLGTKAQDIKDRAQYYGQQHTGGEHDRDRTRGTHHTTTTT |
| 157 | >tr\|Q94CI3\|Q94CI3_BRAOL Oleosin OS=Brassica oleracea OX=3712 GN=GRP1 PE=3 SV=1  MFFQIIQGVFTGVEALALLAFAGITLGGSAVGLALSTPLFILFSPILVPATIATTLLTTG  FTTSGGLGMVALRIFWKLFKRLRKKGKGTPKIPGLAPGAPDSDPVSGG |
| 158 | >tr\|Q94CI0\|Q94CI0_BRAOL Oleosin OS=Brassica oleracea OX=3712 GN=GRP5 PE=3 SV=1  MFSFLSPLLDVIKVVVASVTSVVLFVFAGLTLSGSAVALVVSTPLFLIFSPILVPATIAT  TLLASGVTAGATLGITAISLIMGLIKTAEGSSLARLAQTPLKLFKFSGGFGGSWGGKPFS  GTFGNKGSQSSGNIPGWLKNLLNGIPGGGAAPAAGGAAPAPAAPAPAAPPG |
| 159 | >tr\|B3H6L6\|B3H6L6_ARATH Oleosin OS=Arabidopsis thaliana OX=3702 GN=GRP19 PE=3 SV=1  MFEIIQAVFSAGVALALLTFAGITLGGSVVACIISTPLFVIFSPVLVPATIATTLLASGN  VQGGTYQKFQD |
| 160 | >tr\|Q9FLS1\|Q9FLS1_ARATH Oleosin family protein OS=Arabidopsis thaliana OX=3702 GN=MBK20.3 PE=4 SV=1  MFSFLIFLLEVYKVVIAVVASIVFFVFSGLTLAGTAVGLTVTTPLFIIFSPILVPATIAI  TLLTTGFTTGGALGATAIALIRRGMGVKSKNNIPAIGAPPTMFAQFSLTPKINYEGTFKG  SWGGKSSPQATPNFSYGGTWTANWGGRSFTGKFGDQSGGGSTAGGSTPEAAGAGAGAAAA  GAGAAAAAGAAAAAVPGLGAASAGAGPGAGAGTPAPAPTGKAGKAGSKKK |
| 161 | >tr\|Q494N8\|Q494N8_ARATH Oleosin OS=Arabidopsis thaliana OX=3702 GN=At2g25890 PE=2 SV=1  MADHQQHQQQQQPIMRSLHESSPSTRQIVRFVTAATIGLSLLVLSGLTLTGTVIGLIVAT  PLMVLFSPVLVPAVITIGLLTMGFLFSGGCGVAAATALTWIYKYVTGKHPMGADKVDYAR  MRIAEKAKELGHYTHSQPQQTHQTTTTTH |
| 162 | >tr\|D7MQY4\|D7MQY4_ARALL Oleosin OS=Arabidopsis lyrata subsp. lyrata OX=81972 GN=ARALYDRAFT_495191 PE=3 SV=1  MADQTRTHLEMISRDSTQESHPKERQMVKAATAVTAGGSLLVLSGLTLAGTVIALTVATP  LLVIFSPVLVPAVITVALIITGFLASGGFGIAAITAFSWVYRHLTGSGSEKIENARMKVG  SRVQDTKYGQHNIGVQHQQAS |
| 163 | >tr\|C3VHQ8\|C3VHQ8_SOYBN Oleosin OS=Glycine max OX=3847 GN=100301903 PE=2 SV=1  MATISDQPRGSYSYGTSYGAPYGTTYETNTSINNPPSRQTVKFITAATIGITLLLLSGLT  LTGTVIGLIIATPLLVIFSPILVPAAFVLFLVASGFLFSGGCGVAAIAALSWIYNYVSGN  QPAGYDTLDYAKGYLADKARDVKERAKDYGSYAQGRINEATQGAY |
| 164 | >tr\|K7KTR9\|K7KTR9_SOYBN Oleosin OS=Glycine max OX=3847 GN=GLYMA_06G078700 PE=3 SV=1  MQSRHDMQSTLTSCRVTSHTSSYTTIYLSHSILSSTLGLAREKIPTMATISTDQPRGSYS  YGTSYGAPYGTTYETNSSINNPPSRQTVKFITAATIGITLLLLSGLTLTGTVIGLIIATP  LLVIFSPILVPAAFVLFLVASGFLFSGGCGVAAIAALSWIYNYVSGNQPAGSDTLDYAKG  YLTDKARDVKERAKDYGSYAQGRINEATQGTY |
| 165 | >tr\|Q6WEQ7\|Q6WEQ7_ARALL Oleosin OS=Arabidopsis lyrata subsp. lyrata OX=81972 GN=GRP19 PE=3 SV=1  MFEIIQAVFAAGAALALLTFSAITLGGSVVAFAISTPLFVVFSPVLVPATIATTLLASGF  TASGSFGATAFSILAWIYKRRTGRDLPKIPGLTPPAAPASNPAGSGV |
| 166 | >tr\|A0A0D3EA08\|A0A0D3EA08_BRAOL Oleosin OS=Brassica oleracea var. oleracea OX=109376 GN=106317459 PE=3 SV=1  MANQTRTHQDIIVRDSRITLDRDHPKTGAQMVKVATGVAAGGSLLVLSGLTLAGTVIAFA  VATPLLIIFSPVLVPAVITVVLIITGFLASGGFGIAAITAFSWLYRHMTGSGSDQKIESA  RMKVGSRGYDTKYGQHNIGVHQQHQQAAS |
| 167 | >tr\|A0A0D3EH62\|A0A0D3EH62_BRAOL Oleosin OS=Brassica oleracea var. oleracea OX=109376 GN=106316426 PE=3 SV=1  MSEELVQHESHSQASIFSRFFRMFSFIFPLLNVIKLIIASVTSLVCLAFSCVTLGGSAVA  LIVSTPLFIIFSPILVPATIATTLLASGLMAGTTLGLTGIGLITGLVRTAGGVTLAESPI  RRIIINRIKARLGGGGGSRLAMLKKILGLIKKLRGMSSGGAAPAAEAAPAAAPADGAAPA  AAPAPT |
| 168 | >tr\|A0A178VRV6\|A0A178VRV6_ARATH Oleosin OS=Arabidopsis thaliana OX=3702 GN=AXX17_At2g21730 PE=3 SV=1  MADHQQHQQQQQPIMRSLHESSPSTRQIVRFVTAATIGLSLLVLSGLTLTGTVIGLIVAT  PLMVLFSPVLVPAVITIGLLTMGFLFSGGCGVAAATALTWIYKYVTGKHPMGADKVDYAR  MRIAEKAKELGHYTHSQPQQTHQTTTTTH |
| 169 | >tr\|F4K3K4\|F4K3K4_ARATH Oleosin family protein OS=Arabidopsis thaliana OX=3702 GN=At5g61610 PE=4 SV=1  MHFFGTKDKAPIYAGIAVVSFMILTGLTFAGTAVALTVMIPVLVVLSPILVPAVITSSFL  ATGFLASGSLGALGIALLIWLYKKEEYSRATLHARGGRPDGPNKLAESGKQSGGDNPLKE  DKPPERDKLPRKDKPSKEDNLLKGDKPVEEDKLPAEEEKPPQKDKPAEGHKPPQKDKPAE  GDKPVEEDKPPQKDKPAEGDKHVEEDMPLGGVEHLSIPEIPKVMVVEQNPIVPKCCGPST  CTKRCHPFRFAMDANATKARSIFMKEYQKVPRVGAFAYKYFTSRDKKYVNHFHI |
| 170 | >tr\|Q84WN2\|Q84WN2_ARATH Oleosin (Fragment) OS=Arabidopsis thaliana OX=3702 GN=At2g25890 PE=2 SV=1  QKQHRTLNPYSLRKRKKEMADHQQHQQQQQPIMRSLHESSPSTRQIVRFVTAATIGLSLL  VLSGLTLTGTVIGLIVATPLMVLFSPVLVPAVITIGLLTMGFLFSGGCGVAAATALTWIY  KYVTGKHPMGADKVDYARMRIAEKAKELGHYTHSQPQQTHQTTTTTH |
| 171 | >tr\|Q94FP8\|Q94FP8_ARATH Oleosin OS=Arabidopsis thaliana OX=3702 GN=C24_LOCUS21470 PE=3 SV=1  MLSFLIPVVQFFQVVIAAVASVVFLVFAGITFGASIVGLTIATPLFVIFSPILVPATIAT  TFLVGGATAAVALGVTAFALILWLFKHRIGVKPKNNPAPKGAPTKADQPGASGGASGDKP  GEMSGAGGPSGDKPGGASGGGDKPGGASGGGPGGASGGASGGASGGGPGGASGGGPGGAS  GGASGDKPEGAPGDKPGGASGGKPGKKPGHKPAGARGGKRLAWW |
| 172 | >tr\|A0A251RQ07\|A0A251RQ07_HELAN Oleosin OS=Helianthus annuus OX=4232 GN=HannXRQ_Chr17g0549281 PE=3 SV=1  MAHNQQYYQQHQHRQPHLSHQVVKTATAATLCGSLMVLSGLTLAATVIGLVVATPLLVIF  SPVLVPALITLSLIFGGFLASGGLGATASFVCYWMYRYVTGKHPVGSRQLDMARDKIAGA  AMEARHKAEQLGHQTGRTAAAGGGVGQGHQIRVEHQAA |
| 173 | >tr\|A0A2I4GNJ1\|A0A2I4GNJ1_JUGRE Oleosin OS=Juglans regia OX=51240 GN=LOC109009462 PE=3 SV=1  MSDHSRPVSQALYDPSSTSSRQAVKFLTAVTIGATLLILSGLTLTGTVIALILATPVLVL  FSPILVPAGIVLFLVAAGLVVSGGCGVVAMTALSWIYNYVTGKHPMGADRLDYARMRIAD  KARDMKEKAKEYGQFVQHKAREATQGA |
| 174 | >tr\|A0A397YDD7\|A0A397YDD7_BRACM Oleosin OS=Brassica campestris OX=3711 GN=BRAA08T34077Z PE=3 SV=1  MADTARTHHDITSRDQYPILGRDRDQYPYGRSDYQTSGQDYSKTRQIAKAATAVTAGGSL  LVLSSLTLVGTVIALTVATPLLVIFSPILVPALITVALLITGFLSSGGFGIAAITVFSWI  YKYATGEHPQGSDKLDSARMKLGTKAQDIKDRAQYYGQQHTGGEHDRDRTRGTHHTTTTT |
| 175 | >tr\|A0A397XNR6\|A0A397XNR6_BRACM Oleosin OS=Brassica campestris OX=3711 GN=BRAA10T44975Z PE=3 SV=1  MFFQIIQGVFTGVEALALLAFAGITLGGSAVGLALSTPLFILFSPILVPATIATTLLTTG  FTTSGGLGIVALRIFWKLFKRLRKKGKGTPKIPGLAPGAPDSNSVSGG |
| 176 | >tr\|A0A397ZLA6\|A0A397ZLA6_BRACM Oleosin OS=Brassica campestris OX=3711 GN=BRAA04T17591Z PE=3 SV=1  MADLHQQHQQPMTRNLHESSSSPSTRQTVRFLTAATIGMSLLVLSGLTLTGTVIGLVVAT  PLMVLFSPVLVPAVITMCLLTAGFLFSGGCGVAAATALSWIYRYVTGKHPMGADKVDYAR  MMISEKAKELGHYAQPQTDQTTTAPY |
| 177 | >tr\|A0A3P6B1V9\|A0A3P6B1V9_BRACM Oleosin OS=Brassica campestris OX=3711 GN=BRAA02T08560Z PE=3 SV=1  MANVDRRVNVDRTDKGLQLQPQYEDRVGYGYGYGGNTDYKSRGPSTNQIVALIAGVPIGG  SLLALAGLTLAGSVIGFMLSIPLFLLFSPVIVPAALTIGLAVTGILASGLFGLTGLSSVS  WVLNYIRGRSDTVPEQLDYAKRRMADAVGYAGQKGKEMGQYVQDKAHEAHDTSLTTETNG  KTRRAHIA |
| 178 | >tr\|Q94FQ5\|Q94FQ5_ARATH Oleosin OS=Arabidopsis thaliana OX=3702 PE=3 SV=1  MLSFLIPVVQFFQVVIAAVASVVFLVFAGITFGASIVGLTIATPLFVIFSPILVPATIAT  TFLVGGATAAVALGVTAFALILWLFKHRIGVKPKNNPAPKGAPTKADQPGASGGASGDKP  GEMSGAGGPSGDKPGGASGGGDKPGGASGGASGGGPGGASGGASGGGPGGASGGASGGGP  GGASGGGPGGASGGASGDKPEGAPGDKPGGASGGKPGKKPGHKPAGARGGKRLAWW |
| 179 | >tr\|Q94FP7\|Q94FP7_ARATH Oleosin OS=Arabidopsis thaliana OX=3702 PE=3 SV=1  MLSFLIPVVQFFQVVIAAVASVVFFVFAGITFGASIVGLTIATPLFVIFSPILVPATIAT  TFLVGGATAAVALGVTAFALILWLFKHRIGVKPKNNPAPKGAPTKADQPGASGGASGDKP  GEMSGAGGPSGDKPGGASGGGDKPGGASGGASGGGPGGASGGASGGGPGGASGGGPGGAS  GGASGDKPEGAPGDKPGGASGGKPGKKPGHKPAGARGGKRLAWW |
| 180 | >tr\|Q94FQ6\|Q94FQ6_ARATH Oleosin OS=Arabidopsis thaliana OX=3702 PE=3 SV=1  MFEIIQAVFSAGVALALLTFAGITLGGSVVACIISTPLLVIFSPVLVPATIATTLLASGF  TASGSFGATAFTILSWLYKKRTGRDLPKIPGLTPPAPASNPAGSGV |
| 181 | >tr\|A0A178UC12\|A0A178UC12_ARATH Oleosin OS=Arabidopsis thaliana OX=3702 GN=AXX17_At5g07240 PE=3 SV=1  MLSFLIPVVQFFQVVIAAVASVVFLVFAGITFGASIVGLTIATPLFVIFSPILVPATIAT  TFLVGGATAAVALGVTAFALILWLFKHRIGVKPKNNPAPKGAPTKADQPGASGDKPGEMS  GAGGPSGDKPGGASGGGDKPGGASGGASGGGPGGASGGASGGGPGGASGGASGGGPGGAS  GGGPGGASGGASGDKPEGAPGDKPGGASGGKPGKKPGHKPAGARGGKRLAWW |
| 182 | >tr\|Q94FR1\|Q94FR1_ARATH Oleosin OS=Arabidopsis thaliana OX=3702 PE=3 SV=1  MLSFLIPVVQFFQVVIAAVASVVFLVFAGITFGASIVGLTIATPLFVIFSPILVPATIAT  TFLVGGATAAVALGVTAFALILWLFKHRIGVKPKNNPAPKGAPTKADQPGASGDKPGEMS  GAGGPSGDKPGGASGGGDKPGGASGGASGGGPGGASGGASGGGPGGASGGASGGGPGGAS  GGGPGGASGGASGDKPEGAPGDKPGGASGGKPGKKPGHKPAGARGGKRLAWW |
| 183 | >tr\|O04925\|O04925_SESIN Oleosin OS=Sesamum indicum OX=4182 PE=2 SV=1  MADEPHDQRPTDVIKSYLPEKGPSTSQVLAVVTLFPLGAVLLCLAGLILTGTIIGLAVAT  PLFVIFSPILVPAALTIALAVTGFLTSGAFGITALSSISWLLNYVRRMRGSLPEQLDHAR  RRVQETVGQKTREAGQRSQDVIRP |
| 184 | >tr\|A0A398AHE7\|A0A398AHE7_BRACM Oleosin OS=Brassica campestris OX=3711 GN=BRARA_B03306 PE=3 SV=1  MANVDRRVNVDRTDKGLQLQPQYEDRVGYGYGYGGNTDYKSRGPSTNQIVALIAGVPIGG  SLLALAGLTLAGSVIGFMLSIPLFLLFSPVIVPAALTIGLAVTGILASGLFGLTGLSSVS  WVLNYIRGRSDTVPEQLDYAKRRMADAVGYAGQKGKEMGQYVQDKAHEAHDTSLTTETNG  KARRAHIA |
| 185 | >tr\|A0A444YZD4\|A0A444YZD4_ARAHY Oleosin OS=Arachis hypogaea OX=3818 GN=Ahy_B05g074500 PE=3 SV=1  MATATDRAPHQVQVHTPTTQRVDVQRRGYDVSGGGVKTLFPDRGPSTSQIIAVLVGVPTG  GTLLLLSGLSLLGTIIGLAIATPVFIFFSPVIVPAVVTIGLAVIGILTAGACGLTGLMSL  SWMINFIRQVHGTTVPDQLDSAKRRMADMADYVGQKTKDAGQEIQTKAQDVKRSS |
| 186 | >tr\|A0A397XNC0\|A0A397XNC0_BRACM Oleosin OS=Brassica campestris OX=3711 GN=BRAA10T44971Z PE=3 SV=1  MLSFFSPLLDVIKVVVASVTSVVLFLFAGLTLSGSAVALVVSTPLFLIFSPILVPATIAT  TLLASGLTAGATLGITAISLIMGLIKTAEGSSLARLAQTPLKLFKFSGGFGGSWGGKSFS  GTFGNKGSQSSGNIPGWLKNLLNGIPAGGAVPAAGEAAPAPAAGGAAPAPAAPPG |
| 187 | >tr\|B5TMA5\|B5TMA5_SESIN Oleosin OS=Sesamum indicum OX=4182 PE=2 SV=1  MAEHYGQQQQTRAPHPQLQPRAQRVVKAATAVTAGGSLLVLSGLTLAGTVIALTIATPLL  VIFSPVLVPAVITIFLLGAGFLASGGFGVAALSVLSWIYRYLTGKHPPGADQLESAKTKL  ASKAREMKDRAEQFSQQPVAGSQTS |
| 188 | >tr\|A0A3N6QIR7\|A0A3N6QIR7_BRACR Oleosin OS=Brassica cretica OX=69181 GN=DY000_00012937 PE=3 SV=1  MADTARTHHDITSRDQYPMLGRDRDQYPYGRSDYQTSGQDYSKTRQIAKAATAVTAGGSL  LVLSSLTLVGTVIALTVATPLLVIFSPILVPALITVALLITGFLSSGGFGIAAITVFSWI  YKYATGEHPQGSDKLDSARMKLGSKAQDIKDRAQYYGQQHTGGEHDRDRTRGTHHTTTTT |
| 189 | >tr\|A0A3N6S1W5\|A0A3N6S1W5_BRACR Oleosin OS=Brassica cretica OX=69181 GN=DY000_00000294 PE=3 SV=1  MTTVDRRVNVDRTDKGLQLKPQYEDRVGYGAGYDYGYGADYKSRGPSTNQVVALIAGVPI  GGSLLALAGLTLAGSVIGLMLSIPLFLLFSPVIVPAALTIGLAVTGILASGLFGLTGLSS  VSWVLNYIRGTSDTVPEQLDYAKRRMADAVGYAGQKGKEVGQYVQDKAHEAHDTSLTTET  REPVQAKRTLA |
| 190 | >tr\|Q94CI1\|Q94CI1_BRAOL Oleosin OS=Brassica oleracea OX=3712 GN=GRP4 PE=3 SV=1  MSEELVRHESHSQASIFSRFFRMFSFIFPLLNVIKLIIASVTSLVCLAFSCVTLGGSAVA  LIVSTPLFIIFSPILVPATIATTLLASGLMAGTTLGLTGIGLITGLVRTAGGVTLAESPI  RRIIINRIKARLGGGGGSRLAMLKKILGLIKKLRGMSSGGAAPAAEAAPAAAPADGAAPA  AAPAPT |
| 191 | >tr\|A0A3P6DW75\|A0A3P6DW75_BRAOL Oleosin OS=Brassica oleracea OX=3712 GN=BOLC2T11502H PE=3 SV=1  MTTVDRRVNVDRTDKGLQLKPQYEDRVGYGAGYDYGYGADYKSRGPSTNQVVALIAGVPI  GGSLLALAGLTLAGSVIGLMLSIPLFLLFSPVIVPAALTIGLAATGILASGLFGLTGLSS  VSWVLNYIRGTSDTVPEQLDYAKRRMADAVGYAGQKGKEMGQYVQDKAHEAHDTSLTTET  REPVQAKRTLA |
| 192 | >tr\|A0A3P6GN93\|A0A3P6GN93_BRAOL Oleosin OS=Brassica oleracea OX=3712 GN=BOLC8T48598H PE=3 SV=1  MADTARTHHDITSRDQYPMLGRDRDQYPYGRSDYQTSGQDYSKTRQIAKAATAVTAGGSL  LVLSSLTLVGTVIALTVATPLLVIFSPILVPALITVALLITGFLSSGGFGIAAITVFSWI  YKYATGEHPQGSDKLDSARMKLGSKAQDIKDRAQYYGQQHTGGEHDRDRTRGTHHTTTTT |
| 193 | >tr\|B9RAW7\|B9RAW7_RICCO Oleosin 18.2 kDa, putative OS=Ricinus communis OX=3988 GN=RCOM_1509400 PE=4 SV=1  MAERLQQQQQQGHDKKGPSTSQILAVVTLLPLSGTLLFLAGATLVGALTGLGVTTPLFVI  FSPVLVPAAFVIGLAVLGFSVSGAFGITALSSLSWMLNNFRRMIGLLPQQMEHAKRRVQE  TTGQLGQKARDVGQTVQSKAQ |
| 194 | >tr\|A0A251SMH6\|A0A251SMH6_HELAN Putative oleosin OS=Helianthus annuus OX=4232 GN=HannXRQ_Chr14g0460941 PE=4 SV=1  MTDFYHHRGQAMHHRQYNESRAHQVVKAATAATAGGSLLVLSGLTLVGTVITLTVATPLL  VIFSPVLVPAVMTVFLLATGFLTSGGFGLAAATVFSWMYRYVTGERRTGVESYDQMRRKV  GSKGRARDDHASGKGHHATGGVQLTTGARTGLFES |
| 195 | >tr\|Q9FKT7\|Q9FKT7_ARATH Glycine-rich protein / oleosin OS=Arabidopsis thaliana OX=3702 GN=MDA7.16 PE=2 SV=1  MENPGGRRRRDIAITDGERTTGSSVVAATVAAVAVVGPLFGLMSFSFVATVTLFLIASPL  LLIFAPAFMVTVAVLVSAMVGVGVAAAMWMMGIAALVCCGREIGIETGLAGRMVESVVRE  LGYGRSRYLRDKSEDSYSTSSPSSRDYSSS |
| 196 | >tr\|A0A397XXY0\|A0A397XXY0_BRACM Oleosin OS=Brassica campestris OX=3711 GN=BRARA_J02523 PE=3 SV=1  MSEELVQHESHSQASIFSRFFRMFSFIFPLLNVIKLIIASVTSLVCLAFSCVALGGSAVA  LIVSTPLFIMFSPILVPATIATTLLASGLMAGTTLGLTGIGLIMGLVRTAGGVSLLQSPL  RKIIVNRIKARLGGGGGGSRLARLKKILGLLNKLRGMGAGGAAAPAAEPAPAAEAAPAAE  AAPAAAHAAAPAAAP |
| 197 | >tr\|A0A397XIT4\|A0A397XIT4_BRACM Oleosin OS=Brassica campestris OX=3711 GN=BRARA_J00613 PE=3 SV=1  MANQTRTHQDIIVRDSRSTLDRDHPKTGAQMVKVATGVAAGGSLLVLSGLTLAGTVIALA  VATPLLIIFSPVLVPAVITVVLIITGFLASGGFGIAAITAFSWLYRHMTGSGSDQKIESA  RMKVGSRGYDTKYGQHNIGVHQQHQQAAS |
| 198 | >tr\|A0A398A5G3\|A0A398A5G3_BRACM Oleosin OS=Brassica campestris OX=3711 GN=BRARA_B00248 PE=3 SV=1  MLSFLISLLDIIKLVIASATFLFFLAFSGLTFAGSAVALVVSTPLFIIFSPILVPATIST  TLLASGLAAGTSLGLTAIGLIMRLIKPAGGTSLLFSSPTPLNLVTYSGQFEGSVLGKTYT  GTFDNKSGGGIKWTVTWGSRTFSGTIPLPAAAAPAAAPAAPAAAPAAPAAEAAPAAPAAA  PAAPAAPAAAPAAPAAGAAPPAW |
| 199 | >tr\|A0A445C916\|A0A445C916_ARAHY Oleosin OS=Arachis hypogaea OX=3818 GN=Ahy_A07g033289 PE=3 SV=1  MSDQTRTGYGGGGSYGSSYGGGGTYGSSYGTSYDPSTNQPIRQAIKFMTASAIGVSFLIL  SGLILTGTVIGLIIATPLLVIFSPILVPAAITLALAAGGFLFSGGCGVAAIAALSWLYSY  VTGKHPAGSDRLDYAKGVIADKARDVKDRAKDYAGAGRAQEGTQGY |
| 200 | >tr\|A0A445BU36\|A0A445BU36_ARAHY Oleosin OS=Arachis hypogaea OX=3818 GN=Ahy_A08g038688 PE=3 SV=1  MSDQTRTGYGGGGSYGSSYGGGGTYGSSYGTSYDPSTNQPIRQAIKFMTASTIGVSFLIL  SGLILTGTVIGLIIATPLLVIFSPILVPAAITLALAAGGFLFSGGCGVAAIAALSWLYSY  VTGKHPAGSDRLDYAKGVIADKARDVKDRAKDYAGAGRAQEGTQGY |
| 201 | >tr\|Q647G3\|Q647G3_ARAHY Oleosin OS=Arachis hypogaea OX=3818 PE=2 SV=1  MSDQTRTGYGGGGSYGSSYGGGGTYGSSYGTSYDPSTNQPIRQAIKFMTASTIGVSFLIL  SGLILTGTVIGLIIATPLLVIFSPILVPAAITLALAAGGFLFSGGCGVAAIAALSWLYSY  VTGKHPAGSDRLDYAKGVIADKARDVKDRAKDYAGAGRAQEGTPGY |
| 202 | >tr\|A0A5E4EAT1\|A0A5E4EAT1_PRUDU Oleosin OS=Prunus dulcis OX=3755 GN=ALMOND_2B006731 PE=3 SV=1  MADQHFQQPLHFQGSYGQQQPRSYQVAKAATAVTAGGSLLVLSGLVLAGTVIALTIATPL  LVIFSPVLVPALITVALITMGFLTSGGFGVAAVTVLSWIYKYVTGKQPPGADQLDQARHK  LAGKARDIKDRAEQFGQQHVPSGQQQSS |
| 203 | >tr\|A0A445FAH8\|A0A445FAH8_GLYSO Oleosin OS=Glycine soja OX=3848 GN=D0Y65_050056 PE=3 SV=1  MAEVRSQQPQHVQVHASTTHTPQQYRYYQGGAKTQHHGGEGGGVMSLFPEISLTGSQLLA  LLAGVPLGGMLLLLSGVSLIASLVGLAVATPLFIFFSPVLVPAAFVIGMAVTAVLAAGAC  GLVGLVSFSWLVNCLRQMPRGTTTKTTMMRPEQAKRHVADMEEYVGKKTKDVGQDIQTRA  HAQGTTGMLGLWVWVFDAVLVVVWVWVFDAVLVVVWVFGVGLAASGDLVEQDWTGKIGSD  PTSHHKHSAIFNGDDATRVYQNSDGVKSAATDGE |
| 204 | >tr\|A0A3P6EKY2\|A0A3P6EKY2_BRAOL Oleosin OS=Brassica oleracea OX=3712 GN=BOLC9T59708H PE=3 SV=1  MSEELVQHESHSQASIFSRFFRMFSFIFPLLNVIKLIIASVTSLVCLAFSCVTLGGSAVA  LIVSTPLFIIFSPILVPATIATTLLASGLMAGTTLGLTGIGLITGLVRTAGGVTLAESPI  RRIIINRIKARLGGGGGSRLAMLKKILGLIKKLRGMSSGGAAPAAEAAPAAAPADGAAPA  AAPAPT |
| 205 | >tr\|C3S7F6\|C3S7F6_BRANA Oleosin OS=Brassica napus OX=3708 GN=S5 PE=2 SV=1  MANQTRTHQDIIVRDSRITLDRDHPKTGAQMVKVATGVAAGGSLLVLSGLTLAGTVIAFA  VATPLLIIFSPVLVPAVITVVLIITGFLASGGFGIAAITAFSWLYRHMTGSGSDQKIESA  RMKVGSRGYDTKYGQHNIGVHQQHQQAAS |
| 206 | >tr\|A0A5E4FAC4\|A0A5E4FAC4_PRUDU Oleosin OS=Prunus dulcis OX=3755 GN=ALMOND_2B028251 PE=3 SV=1  MADQSRHVTLKLQDHDHSAAPPSSLRLTGKFLTAGAAGTTLLVLSGLTLTGTVMALIMAT  PVLVVFSPILVPAGIVVFLTAAGLVFSGGCGVAAVTTLTLMYKYISSYVATKKRAYLYGQ  YFCPF |
| 207 | >tr\|B9RE45\|B9RE45_RICCO Oleosin, putative OS=Ricinus communis OX=3988 GN=RCOM_1618240 PE=4 SV=1  MADRDRASNPAQRPTRTATSNATPNNHVSTFLRKLQSHAPNSTQFMGILTLLISGSILLL  LIGITVTALVMGLVFFTPLIIVSSPIWFPIGVLLFFAAAGFFSVCGFLVATIGGLSWMYR  YYRGMHPPGSDRFDYARSRIYDTASHVKDYAREYGGYLQSKVKDAAPGA |

**S3 Table.** Identification yield at false discovery rate (FDR) threshold for *B. rapa* R-o-18 database used to analyses mass spectroscopic data.

| Data level | FDR | Brassica rapa R-o-18 database | | | |
| --- | --- | --- | --- | --- | --- |
| Sample number |  | 10 | 16 | 19 | 49 |
| Number of spectra in search |  | 20712 | 18200 | 16257 | 15282 |
| FDR type: Local |  |  |  |  |  |
| FDR of protein | 1% | 449 | 418 | 315 | 332 |
|  | 5% | 449 | 436 | 315 | 332 |
|  | 10% | 449 | 449 | 315 | 332 |
| FDR of distinct peptide | 1% | 1677 | 1751 | 1246 | 1236 |
|  | 5% | 1784 | 1891 | 1330 | 1333 |
|  | 10% | 1833 | 1952 | 1367 | 1377 |
| False discovery rate of spectra | 1% | 7871 | 8324 | 5679 | 6316 |
|  | 5% | 8587 | 9017 | 6036 | 6694 |
|  | 10% | 8916 | 9666 | 6197 | 6869 |
| Number of identified proteins |  | 323 | | | |
| Number of non-redundant proteins identified in at least 3 out of 4 biological replicates with at least 2 unique peptides and a p value of < 0.05 |  | 233 | | | |

FDR=False discovery rate.

**S4 Table.** List of proteins identified from liquid chromatography–mass spectrometry (LC/MS) analysis using *Brassica rapa* R-o-18 database.

| # | Identified Proteins |  |  | Total Spectrum Count | Total Spectrum Count | Total Spectrum Count | Total Spectrum Count |
| --- | --- | --- | --- | --- | --- | --- | --- |
|  |  |  |  | Uncategorized Sample | Uncategorized Sample | Uncategorized Sample | Uncategorized Sample |
|  |  | Accession Number | Molecular Weight | Pool 1 | Pool 2 | Pool 3 | Pool 4 |
| 1 | Brassica rapa ro18 1.5 Candidate 4 cruciferin | Bra022801 (+5) |  | 656 | 452 | 367 | 343 |
| 2 | PREDICTED: Brassica napus embryonic protein DC-8-like (LOC106447584), transcript variant X1, mRNA ; PREDICTED: Brassica napus embryonic protein DC-8-like (LOC106447584), transcript variant X2, mRNA ; Similar to SBP65 Seed biotin-containing protein SBP65 (Glycine max)::Bra040977:0-729 | Bra040977 | 78 kDa | 347 | 428 | 450 | 213 |
| 3 | Brassica rapa ro18 1.5 Candidate 3 cruciferin | Bra035434 (+5) |  | 429 | 306 | 209 | 236 |
| 4 | Brassica rapa ro18 1.5 Candidate 1 cruciferin | Bra002906 (+2) |  | 364 | 278 | 293 | 225 |
| 5 | PREDICTED: Brassica napus jacalin-related lectin 36-like (LOC106376924), mRNA ; PREDICTED: Brassica napus jacalin-related lectin 36-like (LOC106454993), mRNA ; PREDICTED: Brassica oleracea var. oleracea jacalin-related lectin 36 (LOC106324420), mRNA ; PREDICTED: Brassica rapa jacalin-related lectin 36 (LOC103835222), mRNA ; Similar to JAL36 Jacalin-related lectin 36 (Arabidopsis thaliana)::Bra014461:0-460 | Bra014461 | 49 kDa | 250 | 290 | 271 | 268 |
| 6 | PREDICTED: Brassica rapa oleosin S2-2 (LOC103854221), mRNA ; Similar to S2 Oleosin S2-2 (Brassica napus)::Bra019493:0-188 | Bra019493 (+1) | 20 kDa | 220 | 300 | 250 | 174 |
| 7 | Similar to JAL5 Jacalin-related lectin 5 (Arabidopsis thaliana)::Bra029497:0-970 | Bra029497 |  | 286 | 294 | 95 | 205 |
| 8 | PREDICTED: Brassica oleracea var. oleracea oleosin S2-2 (LOC106316842), mRNA ; PREDICTED: Brassica rapa oleosin S2-2 (LOC103837001), mRNA ; Similar to S2 Oleosin S2-2 (Brassica napus)::Bra000167:0-188 | Bra000167 (+1) | 20 kDa | 337 | 391 | 375 | 338 |
| 9 | Oleosin OS=Brassica napus OX=3708 GN=BnaA08g14540D PE=3 SV=1 | A0A078GHK4_BRANA (+3) | 20 kDa | 150 | 202 | 136 | 233 |
| 10 | PREDICTED: Brassica napus jacalin-related lectin 36-like (LOC106397692), mRNA ; PREDICTED: Brassica napus jacalin-related lectin 36 (LOC106346621), mRNA ; PREDICTED: Brassica napus jacalin-related lectin 36 (LOC106397691), mRNA ; PREDICTED: Brassica rapa jacalin-related lectin 36-like (LOC103872285), mRNA ; Similar to JAL36 Jacalin-related lectin 36 (Arabidopsis thaliana)::Bra021102:0-312 | Bra021102 | 34 kDa | 237 | 294 | 304 | 300 |
| 11 | Similar to At5g40420 Oleosin 21.2 kDa (Arabidopsis thaliana)::Bra039545:0-220 | Bra039545 (+1) | 23 kDa | 245 | 335 | 312 | 225 |
| 12 | Brassica napus beta-glucosidase 19 (LOC106376923), mRNA ; PREDICTED: Brassica napus beta-glucosidase 19 (LOC106376923), transcript variant X1, mRNA ; PREDICTED: Brassica oleracea var. oleracea beta-glucosidase 19 (LOC106295008), mRNA ; PREDICTED: Brassica rapa beta-glucosidase 19 (LOC103835230), mRNA ; Similar to BGLU19 Beta-glucosidase 19 (Arabidopsis thaliana)::Bra014460:0-528 | Bra014460 | 60 kDa | 239 | 185 | 57 | 150 |
| 13 | Brassica napus oleosin S3-5 (S3) mRNA, complete cds ; Brassica napus oleosin S3-6 (S3) mRNA, complete cds ; PREDICTED: Brassica napus oleosin 18.5 kDa (LOC106361728), mRNA ; PREDICTED: Brassica rapa oleosin 18.5 kDa-like (LOC103861532), mRNA ; Similar to Oleosin Bn-V (Fragment) (Brassica napus)::Bra011140:0-183 | Bra011140 (+3) | 20 kDa | 141 | 190 | 88 | 227 |
| 14 | PREDICTED: Brassica napus late embryogenesis abundant protein At3g53040-like (LOC106399842), mRNA ; PREDICTED: Brassica napus late embryogenesis abundant protein At3g53040-like (LOC106409388), mRNA ; PREDICTED: Brassica oleracea var. oleracea embryonic protein DC-8 (LOC106340390), mRNA ; PREDICTED: Brassica rapa embryonic protein DC-8 (LOC103872167), mRNA ; Similar to Embryonic protein DC-8 (Daucus carota)::Bra039225:0-451 | Bra039225 | 49 kDa | 102 | 120 | 133 | 94 |
| 15 | Brassica rapa chiifu v2 candidate 8 cruciferin | BraA09006896 | 49 kDa | 157 | 143 | 72 | 96 |
| 16 | Oleosin OS=Brassica campestris OX=3711 GN=BRARA_C02941 PE=3 SV=1 | A0A397ZZI8_BRACM (+3) | 21 kDa | 94 | 135 | 110 | 114 |
| 17 | Similar to PER1 1-Cys peroxiredoxin PER1 (Arabidopsis thaliana)::Bra029708:0-231 | Bra029708 | 26 kDa | 92 | 126 | 125 | 105 |
| 18 | Brassica napus oleosin S3-3 (S3) mRNA, complete cds ; Brassica napus oleosin S3-4 (S3) mRNA, complete cds ; Brassica napus oleosin S3-7 (S3) mRNA, complete cds ; Brassica rapa oleosin-like protein mRNA, partial cds ; PREDICTED: Brassica napus major oleosin NAP-II-like (LOC106442597), mRNA ; PREDICTED: Brassica napus oleosin Bn-III-like (LOC106346567), mRNA ; PREDICTED: Brassica napus oleosin Bn-III (LOC111202192), mRNA ; PREDICTED: Brassica oleracea var. oleracea oleosin Bn-III (LOC106298955), transcript variant X1, misc_RNA ; PREDICTED: Brassica oleracea var. oleracea oleosin Bn-III (LOC106298955), transcript variant X2, mRNA ; PREDICTED: Brassica rapa oleosin Bn-III (LOC103862927), mRNA ; Similar to Oleosin Bn-III (Brassica napus)::Bra012836:0-195 | Bra012836 (+1) | 22 kDa | 91 | 122 | 46 | 113 |
| 19 | Similar to HSD1 11-beta-hydroxysteroid dehydrogenase 1A (Arabidopsis thaliana)::Bra017248:0-349 | Bra017248 | 39 kDa | 100 | 101 | 49 | 62 |
| 20 | PREDICTED: Brassica napus late embryogenesis abundant protein (LOC106365978), mRNA ; PREDICTED: Brassica rapa late embryogenesis abundant protein (LOC103842523), mRNA ; Similar to Late embryogenesis abundant protein (Raphanus sativus)::Bra005778:0-183 | Bra005778 | 19 kDa | 87 | 96 | 76 | 96 |
| 21 | PREDICTED: Brassica napus vicilin-like seed storage protein At3g22640 (LOC106454009), mRNA ; PREDICTED: Brassica oleracea var. oleracea vicilin (LOC106297624), mRNA ; PREDICTED: Brassica rapa vicilin-like seed storage protein At3g22640 (LOC103834417), mRNA ; Similar to PAP85 Vicilin-like seed storage protein At3g22640 (Arabidopsis thaliana)::Bra014536:0-486 | Bra014536 (+1) | 55 kDa | 124 | 90 | 68 | 53 |
| 22 | PREDICTED: Brassica napus dehydrin Rab18-like (LOC106366343), mRNA ; PREDICTED: Brassica oleracea var. oleracea dehydrin Rab18-like (LOC106315749), mRNA ; PREDICTED: Brassica rapa dehydrin Rab18-like (LOC103837679), mRNA ; Similar to RAB18 Dehydrin Rab18 (Arabidopsis thaliana)::Bra000838:0-179 | Bra000838 | 18 kDa | 66 | 87 | 113 | 62 |
| 23 | Brassica rapa R-o-18 V1.5 candidate 1 napin | Bra041165 (+1) |  | 84 | 93 | 101 | 66 |
| 24 | PREDICTED: Brassica napus vicilin-like seed storage protein At2g28490 (LOC106352587), mRNA ; PREDICTED: Brassica napus vicilin-like seed storage protein At2g28490 (LOC111204718), mRNA ; PREDICTED: Brassica oleracea var. oleracea vicilin-like antimicrobial peptides 2-2 (LOC106333272), mRNA ; PREDICTED: Brassica rapa vicilin-like seed storage protein At2g28490 (LOC103829747), mRNA ; Similar to At2g28490 Vicilin-like seed storage protein At2g28490 (Arabidopsis thaliana)::Bra025745:0-466 | Bra025745 (+2) | 52 kDa | 95 | 81 | 94 | 73 |
| 25 | Brassica napus peroxygenase 1 (LOC106429504), mRNA ; Similar to PXG1 Peroxygenase 1 (Arabidopsis thaliana)::Bra033733:0-245 | Bra033733 | 28 kDa | 74 | 106 | 67 | 83 |
| 26 | Brassica oleracea HDEM genome, scaffold: C1 ; Brassica rapa genome, scaffold: A01 ; Brassica rapa ME-leaC4 mRNA for late embryogenesis-abundant protein, complete cds ; PREDICTED: Brassica napus late embryogenesis abundant protein 76-like (LOC106349612), mRNA ; PREDICTED: Brassica rapa late embryogenesis abundant protein 76 (LOC103840578), mRNA ; Similar to Late embryogenesis abundant protein 76 (Brassica napus)::Bra014978:0-226 | Bra014978 | 25 kDa | 65 | 73 | 99 | 57 |
| 27 | Similar to Late embryogenesis abundant protein D-34 (Gossypium hirsutum)::Bra030208:0-262 | Bra030208 | 27 kDa | 108 | 139 | 52 | 75 |
| 28 | PREDICTED: Brassica napus NADPH-dependent aldehyde reductase 1, chloroplastic (LOC106392387), mRNA ; PREDICTED: Brassica napus NADPH-dependent aldehyde reductase 1, chloroplastic (LOC111212037), mRNA ; PREDICTED: Brassica oleracea var. oleracea glucose and ribitol dehydrogenase homolog 1 (LOC106297131), mRNA ; PREDICTED: Brassica rapa glucose and ribitol dehydrogenase homolog 1 (LOC103844522), mRNA ; Similar to At1g54870 Glucose and ribitol dehydrogenase homolog 1 (Arabidopsis thaliana)::Bra013750:0-289 | Bra013750 | 31 kDa | 138 | 105 | 20 | 47 |
| 29 | PREDICTED: Brassica napus oil body-associated protein 1A (BNAA10G03690D), mRNA ; PREDICTED: Brassica napus oil body-associated protein 1A (BNAC05G03600D), mRNA ; PREDICTED: Brassica oleracea var. oleracea uncharacterized LOC106294119 (LOC106294119), mRNA ; PREDICTED: Brassica rapa oil body-associated protein 1A (LOC103844119), mRNA ; Protein of unknown function::Bra036039:0-264 | Bra036039 | 30 kDa | 83 | 83 | 69 | 46 |
| 30 | PREDICTED: Brassica napus late embryogenesis abundant protein 31-like (LOC106366934), mRNA ; PREDICTED: Brassica napus late embryogenesis abundant protein 31-like (LOC106439628), mRNA ; PREDICTED: Brassica oleracea var. oleracea late embryogenesis abundant protein D-34-like (LOC106335925), mRNA ; PREDICTED: Brassica rapa late embryogenesis abundant protein 31-like (LOC103859958), mRNA ; Similar to Late embryogenesis abundant protein D-34 (Gossypium hirsutum)::Bra010153:0-256 | Bra010153 | 27 kDa | 66 | 91 | 59 | 38 |
| 31 | Brassica oleracea HDEM genome, scaffold: C5 ; Brassica rapa genome, scaffold: A06 ; PREDICTED: Brassica napus heat shock 70 kDa protein 5-like (LOC106346793), mRNA ; PREDICTED: Brassica napus heat shock 70 kDa protein 5-like (LOC106396610), mRNA ; PREDICTED: Brassica oleracea var. oleracea heat shock 70 kDa protein 5 (LOC106295243), mRNA ; PREDICTED: Brassica rapa heat shock 70 kDa protein 5 (LOC103872405), mRNA ; Similar to HSP70-5 Heat shock 70 kDa protein 5 (Arabidopsis thaliana)::Bra021189:0-647 | Bra021189 | 71 kDa | 80 | 72 | 39 | 54 |
| 32 | Similar to GAPC Glyceraldehyde-3-phosphate dehydrogenase, cytosolic (Sinapis alba)::Bra034599:0-290 | Bra034599 | 32 kDa | 80 | 76 | 14 | 48 |
| 33 | PREDICTED: Brassica napus vicilin-like seed storage protein At4g36700 (LOC106359072), partial mRNA ; PREDICTED: Brassica napus vicilin-like seed storage protein At4g36700 (LOC106361437), transcript variant X1, mRNA ; PREDICTED: Brassica napus vicilin-like seed storage protein At4g36700 (LOC106361437), transcript variant X2, mRNA ; PREDICTED: Brassica rapa vicilin-like seed storage protein At4g36700 (LOC103834998), mRNA ; Similar to At4g36700 Vicilin-like seed storage protein At4g36700 (Arabidopsis thaliana)::Bra033857:0-585 | Bra033857 (+2) | 65 kDa | 73 | 77 | 21 | 31 |
| 34 | PREDICTED: Brassica napus protein disulfide isomerase-like 1-1 (LOC106425960), mRNA ; PREDICTED: Brassica napus protein disulfide isomerase-like 1-1 (LOC106425964), mRNA ; PREDICTED: Brassica napus protein disulfide isomerase-like 1-1 (LOC106431998), mRNA ; PREDICTED: Brassica napus protein disulfide isomerase-like 1-1 (LOC106432001), mRNA ; PREDICTED: Brassica oleracea var. oleracea protein disulfide isomerase-like 1-1 (LOC106312445), mRNA ; PREDICTED: Brassica rapa protein disulfide isomerase-like 1-1 (LOC103835729), mRNA ; Similar to PDIL1-1 Protein disulfide isomerase-like 1-1 (Arabidopsis thaliana)::Bra034333:0-501 | Bra034333 | 56 kDa | 66 | 79 | 13 | 50 |
| 35 | PREDICTED: Brassica napus late seed maturation protein P8B6 (LOC106438038), transcript variant X1, mRNA ; PREDICTED: Brassica napus late seed maturation protein P8B6 (LOC106438038), transcript variant X2, mRNA ; PREDICTED: Brassica rapa late seed maturation protein P8B6-like (LOC103857913), mRNA ; Similar to Late seed maturation protein P8B6 (Raphanus sativus)::Bra008633:0-88 | Bra008633 | 10 kDa | 41 | 41 | 49 | 16 |
| 36 | Brassica oleracea HDEM genome, scaffold: C4 ; Brassica rapa genome, scaffold: A05 ; PREDICTED: Brassica napus 17.6 kDa class I heat shock protein 2-like (LOC106451531), mRNA ; PREDICTED: Brassica napus 17.6 kDa class I heat shock protein 2 (LOC106451510), mRNA ; PREDICTED: Brassica oleracea var. oleracea 17.6 kDa class I heat shock protein 2 (LOC106342847), mRNA ; PREDICTED: Brassica rapa 17.6 kDa class I heat shock protein 2 (LOC103868031), mRNA ; Similar to HSP17.8 17.8 kDa class I heat shock protein (Arabidopsis thaliana) ; Zea mays clone 10568 mRNA sequence::Bra029133:0-156 | Bra029133 | 18 kDa | 31 | 46 | 46 | 36 |
| 37 | Similar to A1 Elongation factor 1-alpha 1 (Arabidopsis thaliana)::Bra020415:0-1456 | Bra020415 | 162 kDa | 45 | 67 | 31 | 28 |
| 38 | Similar to Putative invertase inhibitor (Platanus acerifolia)::Bra018664:0-761 | Bra018664 | 82 kDa | 65 | 62 | 26 | 33 |
| 39 | PREDICTED: Brassica napus chaperone protein ClpB1-like (LOC106354381), mRNA ; PREDICTED: Brassica napus chaperone protein ClpB1 (LOC106354344), mRNA ; PREDICTED: Brassica rapa chaperone protein ClpB1 (LOC103831900), mRNA ; Similar to CLPB1 Chaperone protein ClpB1 (Arabidopsis thaliana)::Bra027348:0-911 | Bra027348 | 101 kDa | 50 | 66 | 22 | 34 |
| 40 | PREDICTED: Brassica napus oil body-associated protein 2A (BNACNNG34690D), mRNA ; PREDICTED: Brassica napus oil body-associated protein 2A (LOC106386443), mRNA ; PREDICTED: Brassica oleracea var. oleracea uncharacterized LOC106305268 (LOC106305268), mRNA ; PREDICTED: Brassica rapa oil body-associated protein 2A (LOC103827782), mRNA ; Protein of unknown function::Bra023699:0-246 | Bra023699 | 28 kDa | 57 | 60 | 20 | 29 |
| 41 | PREDICTED: Brassica napus GDSL esterase/lipase At1g54020-like (LOC106345820), transcript variant X1, mRNA ; PREDICTED: Brassica napus GDSL esterase/lipase At1g54020 (LOC106348443), transcript variant X1, mRNA ; PREDICTED: Brassica rapa GDSL esterase/lipase At1g54020-like (LOC103871087), transcript variant X1, mRNA ; Similar to At1g54020 GDSL esterase/lipase At1g54020 (Arabidopsis thaliana)::Bra020125:0-383 | Bra020125 | 43 kDa | 49 | 45 | 29 | 28 |
| 42 | PREDICTED: Brassica rapa low-temperature-induced 65 kDa protein-like (LOC103857129), mRNA ; Similar to LTI65 Low-temperature-induced 65 kDa protein (Arabidopsis thaliana)::Bra007884:0-674 | Bra007884 | 73 kDa | 30 | 52 | 39 | 40 |
| 43 | Brassica napus late embryogenesis abundant protein 76-like (LOC106403035), mRNA ; Brassica napus lea3-1 gene for group 3 late embryogenesis abundant protein, exons 1-2 ; Brassica oleracea HDEM genome, scaffold: C5 ; Brassica oleracea HDEM genome, scaffold: C5 ; Brassica rapa genome, scaffold: A05 ; Brassica rapa subsp. pekinensis clone KBrB021B05, complete sequence ; PREDICTED: Brassica napus late embryogenesis abundant protein 76-like (LOC106397930), mRNA ; PREDICTED: Brassica napus late embryogenesis abundant protein 76-like (LOC106403035), transcript variant X1, mRNA ; PREDICTED: Brassica oleracea var. oleracea late embryogenesis abundant protein 76 (LOC106294785), mRNA ; PREDICTED: Brassica rapa late embryogenesis abundant protein 76-like (LOC103869890), mRNA ; Similar to Late embryogenesis abundant protein 76 (Brassica napus)::Bra030867:0-265 | Bra030867 | 29 kDa | 43 | 59 | 63 | 39 |
| 44 | Similar to Aldose reductase (Hordeum vulgare)::Bra006614:0-322 | Bra006614 | 37 kDa | 50 | 53 | 31 | 26 |
| 45 | Brassica oleracea HDEM genome, scaffold: C7 ; Brassica rapa cultivar Tsuda peptidylprolyl isomerase (ROC1) mRNA, complete cds ; Brassica rapa genome, scaffold: A06 ; PREDICTED: Brassica napus peptidyl-prolyl cis-trans isomerase CYP18-3-like (LOC106349476), mRNA ; PREDICTED: Brassica napus peptidyl-prolyl cis-trans isomerase CYP18-3-like (LOC106349478), mRNA ; PREDICTED: Brassica napus peptidyl-prolyl cis-trans isomerase CYP18-3-like (LOC106410133), mRNA ; PREDICTED: Brassica oleracea var. oleracea peptidyl-prolyl cis-trans isomerase CYP18-3-like (LOC106305761), mRNA ; PREDICTED: Brassica rapa peptidyl-prolyl cis-trans isomerase CYP18-3-like (LOC103828088), mRNA ; Similar to CYP18-3 Peptidyl-prolyl cis-trans isomerase CYP18-3 (Arabidopsis thaliana) ; Zea mays clone 9139 mRNA sequence::Bra023885:0-172 | Bra023885 | 18 kDa | 38 | 51 | 27 | 26 |
| 46 | PREDICTED: Brassica napus alcohol dehydrogenase class-P-like (LOC106356941), mRNA ; PREDICTED: Brassica napus alcohol dehydrogenase class-P (LOC106386015), transcript variant X2, misc_RNA ; PREDICTED: Brassica rapa alcohol dehydrogenase class-P-like (LOC103830574), transcript variant X1, mRNA ; PREDICTED: Brassica rapa alcohol dehydrogenase class-P-like (LOC103830574), transcript variant X2, mRNA ; Similar to ADH1 Alcohol dehydrogenase class-P (Arabidopsis thaliana)::Bra026424:0-413 | Bra026424 | 45 kDa | 65 | 46 | 4 | 29 |
| 47 | Brassica rapa 17.6 kDa class II heat shock protein (LOC103249161), mRNA ; Brassica rapa genome, scaffold: A03 ; PREDICTED: Brassica napus 17.6 kDa class II heat shock protein (LOC106419901), mRNA ; Similar to HSP17.6 17.6 kDa class II heat shock protein (Arabidopsis thaliana)::Bra007053:0-154 | Bra007053 | 18 kDa | 36 | 29 | 9 | 19 |
| 48 | PREDICTED: Brassica napus nitrilase 2 (LOC106387438), transcript variant X1, mRNA ; PREDICTED: Brassica napus nitrilase 2 (LOC106387438), transcript variant X2, mRNA ; PREDICTED: Brassica napus nitrilase 2 (LOC106403172), mRNA ; PREDICTED: Brassica oleracea var. oleracea nitrilase 2-like (LOC106333257), mRNA ; PREDICTED: Brassica rapa nitrilase 2 (LOC103856931), mRNA ; Similar to NIT2 Nitrilase 2 (Arabidopsis thaliana)::Bra007719:0-343 | Bra007719 | 38 kDa | 45 | 39 | 37 | 13 |
| 49 | PREDICTED: Brassica napus em-like protein GEA1 (LOC106437913), mRNA ; PREDICTED: Brassica rapa em-like protein GEA1 (LOC103848642), mRNA ; Similar to EM1 Em-like protein GEA1 (Arabidopsis thaliana)::Bra014069:0-172 | Bra014069 | 19 kDa | 38 | 37 | 32 | 11 |
| 50 | PREDICTED: Brassica napus poly [ADP-ribose] polymerase 3 (LOC106371650), mRNA ; PREDICTED: Brassica oleracea var. oleracea poly [ADP-ribose] polymerase 3 (LOC106316987), transcript variant X1, mRNA ; PREDICTED: Brassica oleracea var. oleracea poly [ADP-ribose] polymerase 3 (LOC106316987), transcript variant X2, mRNA ; PREDICTED: Brassica rapa poly [ADP-ribose] polymerase 3 (LOC103845539), mRNA ; Similar to PARP3 Poly [ADP-ribose] polymerase 3 (Arabidopsis thaliana)::Bra036910:0-809 | Bra036910 | 91 kDa | 27 | 42 | 19 | 38 |
| 51 | PREDICTED: Brassica napus glutathione S-transferase F9 (LOC106387849), transcript variant X2, mRNA ; PREDICTED: Brassica napus glutathione S-transferase F9 (LOC106437571), mRNA ; PREDICTED: Brassica napus glutathione S-transferase F9 (LOC106437572), mRNA ; PREDICTED: Brassica rapa glutathione S-transferase F9 (LOC103857341), mRNA ; Similar to GSTF9 Glutathione S-transferase F9 (Arabidopsis thaliana)::Bra008026:0-215 | Bra008026 | 24 kDa | 42 | 53 | 9 | 18 |
| 52 | Brassica oleracea var. viridis disulfide isomerase-like protein 1-2 mRNA, complete cds ; PREDICTED: Brassica napus protein disulfide isomerase-like 1-2 (LOC106354602), mRNA ; PREDICTED: Brassica napus protein disulfide isomerase-like 1-2 (LOC106389517), mRNA ; PREDICTED: Brassica napus protein disulfide isomerase-like 1-2 (LOC106400427), mRNA ; PREDICTED: Brassica oleracea var. oleracea protein disulfide isomerase-like 1-2 (LOC106300838), mRNA ; PREDICTED: Brassica rapa protein disulfide isomerase-like 1-2 (LOC103832197), mRNA ; Similar to PDIL1-2 Protein disulfide isomerase-like 1-2 (Arabidopsis thaliana)::Bra027552:0-509 | Bra027552 | 56 kDa | 31 | 51 | 15 | 37 |
| 53 | PREDICTED: Brassica napus aspartyl protease AED3-like (LOC106419485), mRNA ; PREDICTED: Brassica napus aspartyl protease AED3-like (LOC106446100), mRNA ; PREDICTED: Brassica oleracea var. oleracea protein ASPARTIC PROTEASE IN GUARD CELL 2 (LOC106319255), mRNA ; PREDICTED: Brassica rapa aspartyl protease AED3 (LOC103847097), mRNA ; Protein of unknown function::Bra037658:0-439 | Bra037658 | 47 kDa | 34 | 35 | 20 | 26 |
| 54 | PREDICTED: Brassica napus UPF0098 protein CT_736-like (LOC106435166), mRNA ; PREDICTED: Brassica napus UPF0098 protein CT_736-like (LOC106435872), mRNA ; PREDICTED: Brassica oleracea var. oleracea UPF0098 protein CT_736 (LOC106317405), mRNA ; Protein of unknown function::Bra038002:0-162 | Bra038002 | 18 kDa | 30 | 42 | 24 | 23 |
| 55 | Brassica oleracea HDEM genome, scaffold: C4 ; Brassica rapa genome, scaffold: A05 ; PREDICTED: Brassica napus late embryogenesis abundant protein 18 (BNAC04G09820D), mRNA ; PREDICTED: Brassica napus late embryogenesis abundant protein 18-like (LOC106414888), mRNA ; PREDICTED: Brassica napus late embryogenesis abundant protein 18-like (LOC111211095), mRNA ; PREDICTED: Brassica oleracea var. oleracea uncharacterized LOC106341460 (LOC106341460), mRNA ; PREDICTED: Brassica rapa uncharacterized LOC103867375 (LOC103867375), mRNA ; Protein of unknown function::Bra028656:0-98 | Bra028656 | 11 kDa | 36 | 39 | 35 | 6 |
| 56 | Brassica napus oleosin 21.2 kDa-like (LOC106352688), mRNA ; Brassica napus oleosin 21.2 kDa-like (LOC106402820), mRNA ; PREDICTED: Brassica rapa oleosin 21.2 kDa-like (LOC103850173), mRNA ; Similar to At5g40420 Oleosin 21.2 kDa (Arabidopsis thaliana)::Bra025845:0-211 | Bra025845 (+2) | 22 kDa | 183 | 234 | 260 | 139 |
| 57 | Similar to GSTF3 Glutathione S-transferase F3 (Arabidopsis thaliana)::Bra000078:0-216 | Bra000078 | 25 kDa | 43 | 38 | 16 | 16 |
| 58 | PREDICTED: Brassica napus GDSL esterase/lipase At5g14450-like (LOC106418385), mRNA ; PREDICTED: Brassica napus GDSL esterase/lipase At5g14450 (LOC106372098), mRNA ; PREDICTED: Brassica oleracea var. oleracea GDSL esterase/lipase At5g14450 (LOC106318861), mRNA ; PREDICTED: Brassica rapa GDSL esterase/lipase At5g14450 (LOC103846482), mRNA ; Similar to At5g14450 GDSL esterase/lipase At5g14450 (Arabidopsis thaliana)::Bra037317:0-389 | Bra037317 | 43 kDa | 31 | 35 | 21 | 26 |
| 59 | PREDICTED: Brassica napus mediator of RNA polymerase II transcription subunit 37a-like (LOC106387846), misc_RNA ; PREDICTED: Brassica napus mediator of RNA polymerase II transcription subunit 37f-like (LOC106437759), mRNA ; PREDICTED: Brassica oleracea var. oleracea mediator of RNA polymerase II transcription subunit 37f-like (LOC106335110), mRNA ; PREDICTED: Brassica rapa mediator of RNA polymerase II transcription subunit 37f (LOC103857349), mRNA ; PREDICTED: Raphanus sativus mediator of RNA polymerase II transcription subunit 37f-like (LOC108847038), mRNA ; Similar to MED37A Mediator of RNA polymerase II transcription subunit 37a (Arabidopsis thaliana)::Bra008034:0-669 | Bra008034 (+1) | 74 kDa | 49 | 54 | 32 | 32 |
| 60 | Brassica oleracea HDEM genome, scaffold: C4 ; Brassica rapa genome, scaffold: A05 ; PREDICTED: Brassica napus heme-binding protein 2-like (LOC106362450), mRNA ; PREDICTED: Brassica napus heme-binding protein 2-like (LOC106372750), mRNA ; PREDICTED: Brassica rapa uncharacterized LOC103867148 (LOC103867148), mRNA ; Protein of unknown function::Bra028439:0-199 | Bra028439 | 22 kDa | 27 | 36 | 22 | 19 |
| 61 | Similar to ADH1 Alcohol dehydrogenase class-P (Arabidopsis thaliana)::Bra027518:0-415 | Bra027518 (+1) | 45 kDa | 65 | 47 | 4 | 20 |
| 62 | PREDICTED: Brassica napus phosphoglycerate kinase 3, cytosolic-like (LOC106381505), transcript variant X1, mRNA ; PREDICTED: Brassica napus phosphoglycerate kinase 3, cytosolic-like (LOC106430726), mRNA ; PREDICTED: Brassica napus phosphoglycerate kinase 3, cytosolic (LOC106354791), mRNA ; PREDICTED: Brassica oleracea var. oleracea phosphoglycerate kinase, cytosolic-like (LOC106300883), mRNA ; PREDICTED: Brassica rapa phosphoglycerate kinase, cytosolic (LOC103832426), transcript variant X1, mRNA ; PREDICTED: Brassica rapa phosphoglycerate kinase, cytosolic (LOC103832426), transcript variant X2, mRNA ; Similar to Phosphoglycerate kinase, cytosolic (Nicotiana tabacum)::Bra027664:0-401 | Bra027664 | 42 kDa | 42 | 31 | 11 | 21 |
| 63 | Brassica oleracea HDEM genome, scaffold: C2 ; Brassica rapa genome, scaffold: A02 ; Brassica rapa subsp. pekinensis clone KBrH010D15, complete sequence ; PREDICTED: Brassica napus histone H2B.7-like (LOC106357790), mRNA ; PREDICTED: Brassica napus histone H2B.7-like (LOC106385623), mRNA ; PREDICTED: Brassica oleracea var. oleracea histone H2B.7-like (LOC106322833), mRNA ; PREDICTED: Brassica rapa histone H2B.7-like (LOC103851535), mRNA ; Similar to H2B-3 Histone H2B.3 (Fragment) (Solanum lycopersicum) ; Zea mays clone 10590 mRNA sequence::Bra016706:0-142 | Bra016706 | 15 kDa | 31 | 35 | 9 | 13 |
| 64 | Brassica rapa Chiifu-401 V2 candidate 4 napin | BraA01001883 (+1) | 20 kDa | 89 | 92 | 82 | 91 |
| 65 | Similar to TIL Temperature-induced lipocalin-1 (Arabidopsis thaliana)::Bra016829:0-333 | Bra016829 | 39 kDa | 21 | 25 | 35 | 18 |
| 66 | PREDICTED: Brassica napus glutathione S-transferase DHAR1, mitochondrial (LOC106346950), mRNA ; PREDICTED: Brassica napus glutathione S-transferase DHAR1, mitochondrial (LOC106401061), mRNA ; PREDICTED: Brassica oleracea var. oleracea glutathione S-transferase DHAR1, mitochondrial (LOC106295217), mRNA ; PREDICTED: Brassica rapa glutathione S-transferase DHAR1, mitochondrial (LOC103872760), mRNA ; Similar to DHAR1 Glutathione S-transferase DHAR1, mitochondrial (Arabidopsis thaliana)::Bra021474:0-213 | Bra021474 | 24 kDa | 21 | 42 | 16 | 20 |
| 67 | PREDICTED: Brassica napus myrosinase-binding protein 2-like (LOC111201427), mRNA ; PREDICTED: Brassica oleracea var. oleracea myrosinase-binding protein 2-like (LOC106316347), mRNA ; PREDICTED: Brassica rapa myrosinase-binding protein 2-like (LOC103845081), transcript variant X1, mRNA ; PREDICTED: Brassica rapa myrosinase-binding protein 2-like (LOC103845081), transcript variant X2, mRNA ; PREDICTED: Brassica rapa myrosinase-binding protein 2-like (LOC103845081), transcript variant X3, mRNA ; Similar to MBP2 Myrosinase-binding protein 2 (Arabidopsis thaliana)::Bra036569:0-550 | Bra036569 | 58 kDa | 23 | 23 | 17 | 20 |
| 68 | PREDICTED: Brassica napus probable inactive serine/threonine-protein kinase fnkC (LOC106365884), mRNA ; PREDICTED: Brassica napus probable inactive serine/threonine-protein kinase fnkC (LOC106440360), mRNA ; PREDICTED: Brassica rapa probable inactive serine/threonine-protein kinase fnkC (LOC103837118), mRNA ; Protein of unknown function::Bra000418:0-367 | Bra000418 | 42 kDa | 48 | 23 | 2 | 21 |
| 69 | Similar to MSD2 Superoxide dismutase [Mn] 2, mitochondrial (Arabidopsis thaliana)::Bra005106:0-430 | Bra005106 | 49 kDa | 40 | 22 | 17 | 13 |
| 70 | PREDICTED: Brassica napus actin-7 (LOC106384924), transcript variant X1, mRNA ; PREDICTED: Brassica oleracea var. oleracea actin-7-like (LOC106335398), mRNA ; PREDICTED: Brassica rapa actin-7-like (LOC103855835), mRNA ; Similar to ACT7 Actin-7 (Arabidopsis thaliana)::Bra006948:0-377 | Bra006948 (+2) | 42 kDa | 35 | 26 | 13 | 13 |
| 71 | PREDICTED: Brassica napus 11-beta-hydroxysteroid dehydrogenase-like 5 (LOC106436433), mRNA ; PREDICTED: Brassica oleracea var. oleracea 11-beta-hydroxysteroid dehydrogenase-like 5 (LOC106328447), mRNA ; Similar to HSD5 11-beta-hydroxysteroid dehydrogenase-like 5 (Arabidopsis thaliana)::Bra009101:0-461 | Bra009101 | 52 kDa | 17 | 24 | 17 | 13 |
| 72 | Similar to SSL9 Protein STRICTOSIDINE SYNTHASE-LIKE 9 (Arabidopsis thaliana)::Bra040149:0-853 | Bra040149 | 96 kDa | 30 | 27 | 10 | 25 |
| 73 | PREDICTED: Brassica napus oleosin 14.9 kDa (LOC106375666), mRNA ; PREDICTED: Brassica napus oleosin 14.9 kDa (LOC106402530), mRNA ; PREDICTED: Brassica rapa oleosin 14.9 kDa (LOC103864856), mRNA ; Similar to OL3 Oleosin 14.9 kDa (Arabidopsis thaliana)::Bra035756:0-205 | Bra035756 | 22 kDa | 25 | 19 | 20 | 12 |
| 74 | PREDICTED: Brassica napus late seed maturation protein P8B6-like (LOC106450837), mRNA ; PREDICTED: Brassica napus late seed maturation protein P8B6 (LOC106397804), mRNA ; PREDICTED: Brassica oleracea var. oleracea late seed maturation protein P8B6 (LOC106342772), mRNA ; PREDICTED: Brassica rapa late seed maturation protein P8B6 (LOC103866975), mRNA ; Similar to EM6 Em-like protein GEA6 (Arabidopsis thaliana)::Bra028273:0-84 | Bra028273 | 9 kDa | 46 | 35 | 49 | 17 |
| 75 | PREDICTED: Brassica napus non-specific lipid-transfer protein-like 1 (LOC106402434), mRNA ; PREDICTED: Brassica napus non-specific lipid-transfer protein-like 1 (LOC106447070), transcript variant X1, mRNA ; PREDICTED: Brassica napus non-specific lipid-transfer protein-like 1 (LOC106447070), transcript variant X2, mRNA ; PREDICTED: Brassica oleracea var. oleracea non-specific lipid-transfer protein-like 1 (LOC106315711), mRNA ; PREDICTED: Brassica rapa non-specific lipid-transfer protein-like 1 (LOC103839274), mRNA ; Protein of unknown function::Bra001600:0-123 | Bra001600 | 14 kDa | 17 | 20 | 20 | 21 |
| 76 | PREDICTED: Brassica napus late embryogenesis abundant protein 46 (LOC106419166), mRNA ; PREDICTED: Brassica rapa 18 kDa seed maturation protein (LOC103847016), mRNA ; Similar to GMPM1 18 kDa seed maturation protein (Glycine max)::Bra037704:0-159 | Bra037704 | 16 kDa | 26 | 28 | 18 | 8 |
| 77 | Brassica oleracea var. botrytis peroxidase 7 mRNA, complete cds ; Brassica rapa peroxidase 12 mRNA, partial cds ; PREDICTED: Brassica napus peroxidase 12 (LOC106354161), mRNA ; PREDICTED: Brassica napus peroxidase 12 (LOC106381085), mRNA ; PREDICTED: Brassica oleracea var. oleracea peroxidase 12 (LOC106300904), mRNA ; PREDICTED: Brassica rapa peroxidase 12 (LOC103249149), mRNA ; PREDICTED: Raphanus sativus peroxidase 12 (LOC108828655), mRNA ; Similar to PER12 Peroxidase 12 (Arabidopsis thaliana)::Bra027198:0-362 | Bra027198 | 40 kDa | 18 | 26 | 4 | 22 |
| 78 | Brassica oleracea HDEM genome, scaffold: C2 ; Brassica oleracea HDEM genome, scaffold: C3 ; Brassica oleracea HDEM genome, scaffold: C3 ; Brassica rapa genome, scaffold: A02 ; Brassica rapa genome, scaffold: A08 ; Brassica rapa genome, scaffold: A08 ; Brassica rapa subsp. pekinensis clone KBrB027K16, complete sequence ; Brassica rapa subsp. pekinensis clone KBrB027K16, complete sequence ; PREDICTED: Brassica napus elongation factor 2-like (LOC106361586), mRNA ; PREDICTED: Brassica napus elongation factor 2-like (LOC106382668), mRNA ; PREDICTED: Brassica napus elongation factor 2-like (LOC106418012), mRNA ; PREDICTED: Brassica napus elongation factor 2-like (LOC111213817), mRNA ; PREDICTED: Brassica napus elongation factor 2 (LOC106361587), mRNA ; PREDICTED: Brassica rapa elongation factor 2 (LOC103835224), mRNA ; Similar to LOS1 Elongation factor 2 (Arabidopsis thaliana)::Bra034035:0-843 | Bra034035 (+1) | 94 kDa | 21 | 30 | 4 | 16 |
| 79 | Protein of unknown function::Bra016576:0-634 | Bra016576 | 69 kDa | 13 | 33 | 8 | 16 |
| 80 | PREDICTED: Brassica napus cell division control protein 48 homolog D-like (LOC106352759), mRNA ; PREDICTED: Brassica napus cell division control protein 48 homolog D (LOC106452445), mRNA ; PREDICTED: Brassica oleracea var. oleracea cell division control protein 48 homolog D (LOC106296744), mRNA ; PREDICTED: Brassica rapa cell division control protein 48 homolog D (LOC103829916), mRNA ; Similar to CDC48D Cell division control protein 48 homolog D (Arabidopsis thaliana)::Bra025942:0-810 | Bra025942 | 90 kDa | 26 | 26 | 11 | 6 |
| 81 | PREDICTED: Brassica napus adenylate kinase 3 (LOC106388427), mRNA ; PREDICTED: Brassica napus adenylate kinase 3 (LOC106388472), mRNA ; PREDICTED: Brassica oleracea var. oleracea adenylate kinase 3 (LOC106334666), mRNA ; PREDICTED: Brassica rapa adenylate kinase 3 (LOC103858450), mRNA ; Similar to ADK2 Adenylate kinase 3 (Arabidopsis thaliana)::Bra009032:0-245 | Bra009032 | 27 kDa | 10 | 23 | 16 | 22 |
| 82 | Brassica rapa genome, scaffold: A01 ; Brassica rapa subsp. pekinensis clone KBrB061L05, complete sequence ; PREDICTED: Brassica napus 23.6 kDa heat shock protein, mitochondrial (LOC106443195), mRNA ; PREDICTED: Brassica rapa 23.6 kDa heat shock protein, mitochondrial (LOC103862989), mRNA ; Similar to HSP23.6 23.6 kDa heat shock protein, mitochondrial (Arabidopsis thaliana)::Bra012843:0-149 | Bra012843 | 17 kDa | 21 | 25 | 12 | 11 |
| 83 | PREDICTED: Brassica napus fructose-bisphosphate aldolase 8, cytosolic (LOC106367801), mRNA ; PREDICTED: Brassica napus fructose-bisphosphate aldolase 8, cytosolic (LOC106367922), transcript variant X1, mRNA ; PREDICTED: Brassica napus fructose-bisphosphate aldolase 8, cytosolic (LOC106367922), transcript variant X2, mRNA ; PREDICTED: Brassica oleracea var. oleracea fructose-bisphosphate aldolase, cytoplasmic isozyme-like (LOC106312678), mRNA ; PREDICTED: Brassica rapa fructose-bisphosphate aldolase, cytoplasmic isozyme-like (LOC103841225), mRNA ; Similar to Fructose-bisphosphate aldolase, cytoplasmic isozyme (Spinacia oleracea)::Bra004845:0-358 | Bra004845 | 38 kDa | 26 | 20 | 9 | 12 |
| 84 | Brassica juncea basic glucanase mRNA, complete cds ; Brassica rapa genome, scaffold: A07 ; Brassica rapa subsp. pekinensis glucanase 1 (Glu1) mRNA, complete cds ; PREDICTED: Brassica napus probable glucan endo-1,3-beta-glucosidase BG3 (LOC106353239), mRNA ; PREDICTED: Brassica napus probable glucan endo-1,3-beta-glucosidase BG3 (LOC106410962), mRNA ; PREDICTED: Brassica napus probable glucan endo-1,3-beta-glucosidase BG3 (LOC106445917), mRNA ; PREDICTED: Brassica napus probable glucan endo-1,3-beta-glucosidase BG3 (LOC106445918), mRNA ; PREDICTED: Brassica oleracea var. oleracea glucan endo-1,3-beta-glucosidase, acidic isoform-like (LOC106296725), mRNA ; PREDICTED: Brassica rapa probable glucan endo-1,3-beta-glucosidase BG3 (LOC103830091), mRNA ; PREDICTED: Raphanus sativus probable glucan endo-1,3-beta-glucosidase BG3 (LOC108815042), mRNA ; Similar to BG3 Probable glucan endo-1,3-beta-glucosidase BG3 (Arabidopsis thaliana)::Bra026071:0-341 | Bra026071 | 38 kDa | 10 | 27 | 11 | 18 |
| 85 | PREDICTED: Brassica napus nitrogen regulatory protein P-II homolog (LOC106438650), mRNA ; PREDICTED: Brassica rapa nitrogen regulatory protein P-II homolog (LOC103858794), mRNA ; Similar to GLB1 Nitrogen regulatory protein P-II homolog (Arabidopsis thaliana)::Bra009285:0-196 | Bra009285 | 21 kDa | 15 | 21 | 19 | 11 |
| 86 | B.napus mRNA for myrosinase MC ; PREDICTED: Brassica napus myrosinase-like (LOC106366548), transcript variant X1, mRNA ; PREDICTED: Brassica napus myrosinase-like (LOC106366548), transcript variant X2, mRNA ; PREDICTED: Brassica napus myrosinase-like (LOC106382308), mRNA ; PREDICTED: Brassica oleracea var. oleracea myrosinase-like (LOC106310472), mRNA ; PREDICTED: Brassica rapa myrosinase-like (LOC103836047), mRNA ; Similar to TGG1 Myrosinase 1 (Arabidopsis thaliana)::Bra034560:0-510 | Bra034560 | 59 kDa | 30 | 12 | 5 | 10 |
| 87 | PREDICTED: Brassica napus 60S ribosomal protein L7a-2-like (LOC106415818), mRNA ; PREDICTED: Brassica napus 60S ribosomal protein L7a-2-like (LOC106420535), mRNA ; PREDICTED: Brassica oleracea var. oleracea 60S ribosomal protein L7a-2-like (LOC106307692), mRNA ; PREDICTED: Brassica rapa 60S ribosomal protein L7a-2-like (LOC103842092), mRNA ; Similar to RPL7AA 60S ribosomal protein L7a-1 (Arabidopsis thaliana) ; Zea mays clone 14482 mRNA sequence::Bra005473:0-242 | Bra005473 (+3) | 28 kDa | 12 | 17 | 12 | 15 |
| 88 | Similar to Embryonic protein DC-8 (Daucus carota)::Bra040652:0-387 | Bra040652 | 42 kDa | 18 | 36 | 33 | 19 |
| 89 | PREDICTED: Brassica napus heat shock 70 kDa protein 10, mitochondrial-like (LOC106430600), mRNA ; PREDICTED: Brassica napus heat shock 70 kDa protein 10, mitochondrial (LOC106384930), mRNA ; PREDICTED: Brassica oleracea var. oleracea heat shock 70 kDa protein 10, mitochondrial (LOC106335389), mRNA ; PREDICTED: Brassica rapa heat shock 70 kDa protein 10, mitochondrial-like (LOC103855827), mRNA ; Similar to HSP70-10 Heat shock 70 kDa protein 10, mitochondrial (Arabidopsis thaliana)::Bra006940:0-681 | Bra006940 | 73 kDa | 15 | 23 | 8 | 14 |
| 90 | PREDICTED: Brassica napus embryo-specific protein ATS3 (LOC106419203), mRNA ; PREDICTED: Brassica rapa uncharacterized LOC103847082 (LOC103847082), mRNA ; Protein of unknown function::Bra037669:0-205 | Bra037669 | 22 kDa | 1 | 15 | 26 | 15 |
| 91 | Similar to OEP162 Outer envelope pore protein 16-2, chloroplastic (Arabidopsis thaliana)::Bra013303:0-179 | Bra013303 | 19 kDa | 26 | 14 | 0 | 16 |
| 92 | Brassica rapa genome, scaffold: A01 ; PREDICTED: Brassica napus uncharacterized BNAA01G30160D (BNAA01G30160D), mRNA ; PREDICTED: Brassica rapa uncharacterized LOC103849726 (LOC103849726), mRNA ; Protein of unknown function::Bra042033:0-88 | Bra042033 | 10 kDa | 10 | 13 | 21 | 8 |
| 93 | PREDICTED: Brassica napus AIG2-like protein A (LOC106351782), mRNA ; PREDICTED: Brassica napus AIG2-like protein A (LOC106356653), mRNA ; PREDICTED: Brassica oleracea var. oleracea AIG2-like protein (LOC106299568), mRNA ; PREDICTED: Brassica rapa AIG2-like protein (LOC103850151), mRNA ; Similar to At5g39720 AIG2-like protein (Arabidopsis thaliana)::Bra025831:0-169 | Bra025831 | 20 kDa | 10 | 17 | 10 | 9 |
| 94 | Similar to At1g53240 Malate dehydrogenase 1, mitochondrial (Arabidopsis thaliana)::Bra031854:0-338 | Bra031854 | 35 kDa | 27 | 22 | 1 | 7 |
| 95 | Brassica rapa chiifu v2 candidate 4 cruciferin | BraA08002105 (+3) | 55 kDa | 127 | 0 | 0 | 0 |
| 96 | Similar to FER2 Ferritin-2, chloroplastic (Arabidopsis thaliana)::Bra031179:0-633 | Bra031179 | 72 kDa | 19 | 20 | 7 | 10 |
| 97 | 12S seed storage protein CRB OS=Arabidopsis thaliana OX=3702 GN=CRB PE=1 SV=2 cruciferin | CRU2_ARATH | 51 kDa | 109 | 82 | 43 | 42 |
| 98 | PREDICTED: Brassica napus malate dehydrogenase 1, cytoplasmic-like (LOC106366626), misc_RNA ; PREDICTED: Brassica napus malate dehydrogenase 1, cytoplasmic (LOC106366627), mRNA ; PREDICTED: Brassica oleracea var. oleracea malate dehydrogenase, cytoplasmic 1 (LOC106319444), mRNA ; PREDICTED: Brassica rapa malate dehydrogenase, cytoplasmic 1 (LOC103839246), mRNA ; Similar to MDH1 Malate dehydrogenase, cytoplasmic 1 (Arabidopsis thaliana)::Bra001572:0-332 | Bra001572 | 36 kDa | 34 | 15 | 1 | 5 |
| 99 | PREDICTED: Brassica napus late embryogenesis abundant protein 1-like (LOC106348819), mRNA ; PREDICTED: Brassica napus late embryogenesis abundant protein 1-like (LOC106373675), mRNA ; Protein of unknown function::Bra012478:0-264 | Bra012478 | 29 kDa | 16 | 14 | 16 | 3 |
| 100 | PREDICTED: Brassica napus late embryogenesis abundant protein 46-like (LOC106413949), mRNA ; PREDICTED: Brassica napus late embryogenesis abundant protein 46-like (LOC106426361), mRNA ; PREDICTED: Brassica oleracea var. oleracea 18 kDa seed maturation protein-like (LOC106335780), transcript variant X1, mRNA ; PREDICTED: Brassica rapa 18 kDa seed maturation protein-like (LOC103855696), mRNA ; Similar to GMPM1 18 kDa seed maturation protein (Glycine max)::Bra006839:0-159 | Bra006839 | 16 kDa | 23 | 30 | 19 | 10 |
| 101 | PREDICTED: Brassica napus glycine-rich RNA-binding protein 2-like (LOC106433663), mRNA ; PREDICTED: Brassica rapa glycine-rich RNA-binding protein 10-like (LOC103846048), mRNA ; Similar to Glycine-rich RNA-binding protein (Daucus carota)::Bra024171:0-159 | Bra024171 | 16 kDa | 10 | 19 | 7 | 10 |
| 102 | Similar to PEC-2 Thioredoxin H-type (Brassica campestris)::Bra001593:0-123 | Bra001593 | 14 kDa | 22 | 11 | 10 | 11 |
| 103 | PREDICTED: Brassica napus probable phospholipid hydroperoxide glutathione peroxidase 6, mitochondrial (LOC106380476), mRNA ; PREDICTED: Brassica napus probable phospholipid hydroperoxide glutathione peroxidase 6, mitochondrial (LOC106396928), mRNA ; PREDICTED: Brassica oleracea var. oleracea probable phospholipid hydroperoxide glutathione peroxidase 6, mitochondrial (LOC106321579), transcript variant X1, mRNA ; PREDICTED: Brassica oleracea var. oleracea probable phospholipid hydroperoxide glutathione peroxidase 6, mitochondrial (LOC106321579), transcript variant X2, mRNA ; Similar to GPX6 Probable phospholipid hydroperoxide glutathione peroxidase 6, mitochondrial (Arabidopsis thaliana)::Bra018546:0-229 | Bra018546 | 25 kDa | 12 | 19 | 10 | 12 |
| 104 | Brassica rapa Chiifu-401 V2 candidate 6 napin | BraA01001887 (+1) | 20 kDa | 72 | 0 | 75 | 0 |
| 105 | Brassica rapa Chiifu-401 V2 candidate 8 napin | BraA03000889 (+3) | 21 kDa | 0 | 62 | 57 | 50 |
| 106 | Brassica rapa genome, scaffold: A03 ; PREDICTED: Brassica napus late embryogenesis abundant protein 29-like (LOC106439419), mRNA ; PREDICTED: Brassica rapa late embryogenesis abundant protein 76-like (LOC103859635), mRNA ; Similar to Late embryogenesis abundant protein 76 (Brassica napus)::Bra009893:0-204 | Bra009893 | 22 kDa | 47 | 57 | 66 | 36 |
| 107 | PREDICTED: Brassica napus UTP--glucose-1-phosphate uridylyltransferase 2-like (LOC106450527), mRNA ; PREDICTED: Brassica napus UTP--glucose-1-phosphate uridylyltransferase 2 (LOC106450722), mRNA ; PREDICTED: Brassica napus UTP--glucose-1-phosphate uridylyltransferase 2 (LOC111207933), mRNA ; PREDICTED: Brassica oleracea var. oleracea probable UTP--glucose-1-phosphate uridylyltransferase 2 (LOC106343185), mRNA ; PREDICTED: Brassica rapa UTP--glucose-1-phosphate uridylyltransferase 2-like (LOC103855145), mRNA ; Similar to At3g03250 Probable UTP--glucose-1-phosphate uridylyltransferase 2 (Arabidopsis thaliana)::Bra031582:0-469 | Bra031582 | 52 kDa | 18 | 22 | 7 | 3 |
| 108 | PREDICTED: Brassica napus aconitate hydratase 1-like (LOC106351933), mRNA ; PREDICTED: Brassica napus aconitate hydratase 1 (LOC106347211), mRNA ; PREDICTED: Brassica oleracea var. oleracea aconitate hydratase 1 (LOC106308688), mRNA ; PREDICTED: Brassica rapa aconitate hydratase 1 (LOC103873312), mRNA ; PREDICTED: Raphanus sativus aconitate hydratase 1-like (LOC108842639), misc_RNA ; Similar to ACO1 Aconitate hydratase 1 (Arabidopsis thaliana)::Bra011617:0-898 | Bra011617 | 98 kDa | 18 | 16 | 7 | 8 |
| 109 | Similar to GSTU21 Glutathione S-transferase U21 (Arabidopsis thaliana)::Bra027601:0-450 | Bra027601 | 52 kDa | 13 | 13 | 9 | 9 |
| 110 | Brassica rapa genome, scaffold: A03 ; Brassica rapa heat shock protein 18 (hsp18) mRNA, complete cds ; Brassica rapa subsp. pekinensis cultivar inbred line Chiifu HSP18.2C (HSP18.2C) gene, partial cds ; Brassica rapa subsp. pekinensis cultivar inbred line Kenshin HSP18.2C (HSP18.2C) gene, partial cds ; PREDICTED: Brassica napus 18.1 kDa class I heat shock protein (LOC106443275), mRNA ; Similar to HSP18.1 18.1 kDa class I heat shock protein (Arabidopsis thaliana)::Bra007554:0-160 | Bra007554 | 18 kDa | 12 | 25 | 23 | 14 |
| 111 | PREDICTED: Brassica napus 17.4 kDa class III heat shock protein-like (LOC106443219), mRNA ; PREDICTED: Brassica rapa 17.4 kDa class III heat shock protein-like (LOC103832638), mRNA ; Similar to HSP17.4B 17.4 kDa class III heat shock protein (Arabidopsis thaliana)::Bra031806:0-154 | Bra031806 | 17 kDa | 13 | 21 | 2 | 6 |
| 112 | PREDICTED: Brassica napus probable histone H2AXa (LOC106346303), mRNA ; PREDICTED: Brassica rapa probable histone H2AXa (LOC103871626), mRNA ; Similar to At1g08880 Probable histone H2AXa (Arabidopsis thaliana)::Bra020481:0-142 | Bra020481 (+1) | 15 kDa | 10 | 11 | 11 | 7 |
| 113 | PREDICTED: Brassica napus desiccation-related protein PCC13-62-like (LOC111213365), transcript variant X2, mRNA ; PREDICTED: Brassica napus desiccation-related protein PCC13-62 (LOC106351214), mRNA ; PREDICTED: Brassica oleracea var. oleracea desiccation-related protein PCC13-62 (LOC106299167), mRNA ; PREDICTED: Brassica rapa desiccation-related protein PCC13-62 (LOC103871506), mRNA ; Similar to Desiccation-related protein PCC13-62 (Craterostigma plantagineum)::Bra020390:0-309 | Bra020390 | 34 kDa | 15 | 16 | 3 | 10 |
| 114 | Similar to GRXC1 Glutaredoxin-C1 (Arabidopsis thaliana)::Bra022411:0-120 | Bra022411 | 13 kDa | 11 | 12 | 13 | 7 |
| 115 | PREDICTED: Brassica napus uncharacterized protein At5g02240-like (LOC106372572), mRNA ; PREDICTED: Brassica napus uncharacterized protein At5g02240 (LOC106433551), mRNA ; PREDICTED: Brassica oleracea var. oleracea uncharacterized protein At5g02240 (LOC106318266), mRNA ; PREDICTED: Brassica rapa uncharacterized protein At5g02240 (LOC103847467), mRNA ; Similar to At5g02240 Uncharacterized protein At5g02240 (Arabidopsis thaliana)::Bra037977:0-253 | Bra037977 | 27 kDa | 13 | 18 | 5 | 5 |
| 116 | Similar to FBA Fructose-bisphosphate aldolase cytoplasmic isozyme (Oryza sativa subsp. japonica)::Bra040649:0-1556 | Bra040649 | 170 kDa | 31 | 14 | 5 | 13 |
| 117 | Similar to GRP10 Glycine-rich RNA-binding protein 10 (Brassica napus)::Bra011387:0-146 | Bra011387 | 14 kDa | 9 | 10 | 13 | 3 |
| 118 | Similar to XYLA Xylose isomerase (Arabidopsis thaliana)::Bra036666:0-654 | Bra036666 | 73 kDa | 11 | 13 | 1 | 15 |
| 119 | PREDICTED: Brassica napus 60S ribosomal protein L17-2-like (LOC106372951), mRNA ; PREDICTED: Brassica napus 60S ribosomal protein L17-2 (LOC106392065), mRNA ; PREDICTED: Brassica oleracea var. oleracea 60S ribosomal protein L17-2-like (LOC106323561), mRNA ; PREDICTED: Brassica rapa 60S ribosomal protein L17-2 (LOC103852402), mRNA ; Similar to RPL17B 60S ribosomal protein L17-2 (Arabidopsis thaliana)::Bra017394:0-179 | Bra017394 (+2) | 20 kDa | 10 | 13 | 6 | 11 |
| 120 | Amphidasya ambigua voucher Clark & Watt 736 (QCNE, MO, UPS) ATP synthase F1 subunit 1 (atp1) gene, complete cds; mitochondrial ; Anthospermum spathulatum voucher Bremer et al. 4405 (UPS) ATP synthase F1 subunit 1 (atp1) gene, complete cds; mitochondrial ; Arabidopsis thaliana ATPase, F1 complex, alpha subunit protein (AT2G07698), mRNA ; Arabidopsis thaliana chromosome 2 BAC T18C6 genomic sequence, complete sequence ; Arabidopsis thaliana chromosome 2 sequence ; Arabidopsis thaliana ecotype C24 mitochondrion, complete genome ; Arabidopsis thaliana ecotype Col-0 mitochondrion, complete genome ; Arabidopsis thaliana ecotype Col-0 mitochondrion, complete genome ; Arabidopsis thaliana ecotype Columbia ATPase subunit 1 mRNA, partial cds; mitochondrial ; Arabidopsis thaliana ecotype Landsberg erecta mitochondrion, complete genome ; Arabidopsis thaliana mitochondrial genome ; Arabidopsis thaliana other for ATSL087910 ; Arabidopsis thaliana other::Bra014393:0-507 | Bra014393 | 55 kDa | 10 | 12 | 5 | 11 |
| 121 | PREDICTED: Brassica napus aldehyde dehydrogenase family 2 member B4, mitochondrial (LOC106408937), mRNA ; PREDICTED: Brassica oleracea var. oleracea aldehyde dehydrogenase family 2 member B4, mitochondrial (LOC106311632), mRNA ; PREDICTED: Brassica rapa aldehyde dehydrogenase family 2 member B4, mitochondrial (LOC103873180), mRNA ; Similar to ALDH2B4 Aldehyde dehydrogenase family 2 member B4, mitochondrial (Arabidopsis thaliana)::Bra021698:0-539 | Bra021698 | 59 kDa | 10 | 17 | 3 | 9 |
| 122 | PREDICTED: Brassica napus superoxide dismutase [Cu-Zn] (LOC106420167), mRNA ; PREDICTED: Brassica rapa superoxide dismutase [Cu-Zn] (LOC103843378), mRNA ; Similar to SODCC Superoxide dismutase [Cu-Zn] (Brassica oleracea var. capitata) ; Zea mays clone 20042 mRNA sequence::Bra006323:0-152 | Bra006323 | 15 kDa | 8 | 12 | 13 | 4 |
| 123 | Similar to ATL5 60S ribosomal protein L5-1 (Arabidopsis thaliana)::Bra024735:0-302 | Bra024735 | 35 kDa | 8 | 8 | 11 | 8 |
| 124 | Similar to HSP90-2 Heat shock protein 90-2 (Arabidopsis thaliana)::Bra016954:0-324 | Bra016954 (+1) | 36 kDa | 7 | 15 | 2 | 9 |
| 125 | Similar to CSD1 Superoxide dismutase [Cu-Zn] 1 (Arabidopsis thaliana)::Bra020480:0-446 | Bra020480 | 49 kDa | 10 | 14 | 9 | 4 |
| 126 | PREDICTED: Brassica napus myrosinase-like (LOC106397816), mRNA ; Similar to TGG2 Myrosinase 2 (Arabidopsis thaliana)::Bra026687:0-546 | Bra026687 | 62 kDa | 34 | 13 | 4 | 10 |
| 127 | PREDICTED: Brassica napus uncharacterized BNAANNG12230D (BNAANNG12230D), mRNA ; PREDICTED: Brassica rapa uncharacterized LOC103843642 (LOC103843642), mRNA ; Protein of unknown function::Bra006472:0-183 | Bra006472 | 20 kDa | 12 | 9 | 5 | 7 |
| 128 | Similar to LTI65 Low-temperature-induced 65 kDa protein (Arabidopsis thaliana)::Bra017183:0-1131 | Bra017183 | 122 kDa | 14 | 26 | 16 | 14 |
| 129 | Brassica oleracea HDEM genome, scaffold: C3 ; Brassica rapa genome, scaffold: A03 ; Brassica rapa subsp. pekinensis clone KBrH007P05, complete sequence ; PREDICTED: Brassica napus 60S ribosomal protein L12-2-like (LOC106392722), mRNA ; PREDICTED: Brassica napus 60S ribosomal protein L12-2-like (LOC106441066), mRNA ; PREDICTED: Brassica oleracea var. oleracea 60S ribosomal protein L12-2 (LOC106335634), mRNA ; PREDICTED: Brassica rapa 60S ribosomal protein L12-2 (LOC103857662), mRNA ; Similar to RPL12A 60S ribosomal protein L12-1 (Arabidopsis thaliana)::Bra008329:0-166 | Bra008329 (+2) | 18 kDa | 0 | 17 | 6 | 11 |
| 130 | PREDICTED: Brassica napus 60S ribosomal protein L4-1-like (LOC111215776), mRNA ; PREDICTED: Brassica napus 60S ribosomal protein L4-1 (LOC106452757), mRNA ; PREDICTED: Brassica oleracea var. oleracea 60S ribosomal protein L4-1-like (LOC106295520), mRNA ; PREDICTED: Brassica rapa 60S ribosomal protein L4-1-like (LOC103870529), mRNA ; Similar to RPL4A 60S ribosomal protein L4-1 (Arabidopsis thaliana)::Bra031264:0-406 | Bra031264 | 45 kDa | 4 | 14 | 8 | 6 |
| 131 | PREDICTED: Brassica napus 40S ribosomal protein S18-like (LOC106360957), mRNA ; PREDICTED: Brassica napus 40S ribosomal protein S18-like (LOC111210018), transcript variant X1, mRNA ; PREDICTED: Brassica napus 40S ribosomal protein S18-like (LOC111210018), transcript variant X2, mRNA ; PREDICTED: Brassica oleracea var. oleracea 40S ribosomal protein S18 (LOC106313634), mRNA ; PREDICTED: Brassica rapa 40S ribosomal protein S18 (LOC103838875), mRNA ; Similar to RPS18A 40S ribosomal protein S18 (Arabidopsis thaliana)::Bra002515:0-173 | Bra002515 (+3) | 20 kDa | 10 | 15 | 4 | 6 |
| 132 | Similar to Endochitinase CH25 (Brassica napus)::Bra031091:0-536 | Bra031091 | 59 kDa | 10 | 14 | 1 | 7 |
| 133 | Brassica rapa genome, scaffold: A03 ; PREDICTED: Brassica napus eukaryotic initiation factor 4A-1-like (LOC106389163), transcript variant X1, mRNA ; PREDICTED: Brassica napus eukaryotic initiation factor 4A-1-like (LOC106389163), transcript variant X2, mRNA ; PREDICTED: Brassica napus eukaryotic initiation factor 4A-1-like (LOC106390450), mRNA ; PREDICTED: Brassica oleracea var. oleracea eukaryotic initiation factor 4A-1-like (LOC106335974), mRNA ; PREDICTED: Brassica rapa eukaryotic initiation factor 4A-1-like (LOC103859548), transcript variant X1, mRNA ; PREDICTED: Brassica rapa eukaryotic initiation factor 4A-1-like (LOC103859548), transcript variant X2, mRNA ; Similar to TIF4A-1 Eukaryotic initiation factor 4A-1 (Arabidopsis thaliana)::Bra009832:0-426 | Bra009832 (+2) | 48 kDa | 10 | 14 | 1 | 8 |
| 134 | Similar to ENO2 Bifunctional enolase 2/transcriptional activator (Arabidopsis thaliana)::Bra028552:0-363 | Bra028552 | 39 kDa | 12 | 9 | 5 | 9 |
| 135 | Brassica oleracea HDEM genome, scaffold: C3 ; Brassica rapa genome, scaffold: A03 ; PREDICTED: Brassica napus histone H4-like (LOC106386836), mRNA ; PREDICTED: Brassica napus histone H4 (LOC106436220), mRNA ; PREDICTED: Brassica oleracea var. oleracea histone H4 (LOC106331525), mRNA ; PREDICTED: Brassica rapa histone H4 (LOC103856615), mRNA ; Similar to Histone H4 (Glycine max)::Bra007556:0-103 | Bra007556 (+4) | 11 kDa | 14 | 13 | 1 | 3 |
| 136 | PREDICTED: Brassica napus triosephosphate isomerase, cytosolic-like (LOC106445356), mRNA ; PREDICTED: Brassica rapa triosephosphate isomerase, cytosolic (LOC103863163), mRNA ; Similar to CTIMC Triosephosphate isomerase, cytosolic (Arabidopsis thaliana)::Bra038454:0-254 | Bra038454 | 27 kDa | 14 | 12 | 3 | 5 |
| 137 | PREDICTED: Brassica napus uncharacterized BNAA10G02700D (BNAA10G02700D), mRNA ; PREDICTED: Brassica napus uncharacterized BNAC05G02680D (BNAC05G02680D), transcript variant X1, mRNA ; PREDICTED: Brassica rapa uncharacterized LOC103844243 (LOC103844243), mRNA ; Protein of unknown function::Bra035274:0-185 | Bra035274 | 20 kDa | 8 | 14 | 6 | 5 |
| 138 | PREDICTED: Brassica napus glutathione S-transferase U19 (LOC106418428), mRNA ; PREDICTED: Brassica oleracea var. oleracea glutathione S-transferase U19-like (LOC106324691), mRNA ; Similar to GSTU19 Glutathione S-transferase U19 (Arabidopsis thaliana)::Bra017892:0-219 | Bra017892 | 26 kDa | 15 | 2 | 3 | 9 |
| 139 | PREDICTED: Brassica napus vicilin-like seed storage protein At4g36700 (LOC106407622), mRNA ; PREDICTED: Brassica napus vicilin-like seed storage protein At4g36700 (LOC106451973), mRNA ; PREDICTED: Brassica oleracea var. oleracea provicilin-like (LOC106305419), mRNA ; PREDICTED: Brassica rapa vicilin-like seed storage protein At4g36700 (LOC103862486), mRNA ; Similar to At4g36700 Vicilin-like seed storage protein At4g36700 (Arabidopsis thaliana)::Bra003060:0-502 | Bra003060 (+1) | 56 kDa | 17 | 19 | 3 | 15 |
| 140 | Brassica oleracea HDEM genome, scaffold: C3 ; Brassica rapa genome, scaffold: A03 ; PREDICTED: Brassica napus 40S ribosomal protein S2-2-like (LOC106437974), mRNA ; PREDICTED: Brassica napus 40S ribosomal protein S2-2 (LOC106388267), mRNA ; PREDICTED: Brassica oleracea var. oleracea 40S ribosomal protein S2-2-like (LOC106335906), mRNA ; PREDICTED: Brassica rapa 40S ribosomal protein S2-2-like (LOC103857998), mRNA ; Similar to RPS2A 40S ribosomal protein S2-1 (Arabidopsis thaliana)::Bra008698:0-282 | Bra008698 (+1) | 31 kDa | 9 | 8 | 6 | 9 |
| 141 | PREDICTED: Brassica napus 60S acidic ribosomal protein P2-2-like (LOC106352563), mRNA ; PREDICTED: Brassica napus 60S acidic ribosomal protein P2-2-like (LOC106352564), mRNA ; PREDICTED: Brassica oleracea var. oleracea 60S acidic ribosomal protein P2-2-like (LOC106339404), mRNA ; PREDICTED: Brassica rapa 60S acidic ribosomal protein P2-2-like (LOC103829681), mRNA ; Similar to RPP2A 60S acidic ribosomal protein P2A (Zea mays)::Bra025686:0-113 | Bra025686 | 11 kDa | 7 | 10 | 11 | 4 |
| 142 | Brassica oleracea HDEM genome, scaffold: C5 ; PREDICTED: Brassica napus stress-response A/B barrel domain-containing protein DABB1 (BNAA05G15190D), mRNA ; PREDICTED: Brassica napus stress-response A/B barrel domain-containing protein DABB1-like (LOC106356988), mRNA ; PREDICTED: Brassica napus stress-response A/B barrel domain-containing protein DABB1-like (LOC106397779), mRNA ; PREDICTED: Brassica oleracea var. oleracea uncharacterized LOC106344145 (LOC106344145), mRNA ; PREDICTED: Brassica rapa stress-response A/B barrel domain-containing protein UP3 (LOC103868574), mRNA ; Protein of unknown function::Bra042262:0-121 | Bra042262 | 13 kDa | 9 | 7 | 8 | 7 |
| 143 | Similar to PRXIIF Peroxiredoxin-2F, mitochondrial (Arabidopsis thaliana)::Bra042236:0-433 | Bra042236 | 48 kDa | 3 | 11 | 8 | 9 |
| 144 | PREDICTED: Brassica napus 60S ribosomal protein L24-2 (LOC106367917), mRNA ; PREDICTED: Brassica napus 60S ribosomal protein L24-2 (LOC106382591), mRNA ; PREDICTED: Brassica rapa 60S ribosomal protein L24-2-like (LOC103841233), mRNA ; Similar to RPL24B 60S ribosomal protein L24-2 (Arabidopsis thaliana)::Bra004854:0-180 | Bra004854 (+3) | 21 kDa | 7 | 7 | 9 | 8 |
| 145 | Brassica napus 11-beta-hydroxysteroid dehydrogenase 1B-like (LOC106388429), mRNA ; PREDICTED: Brassica napus 11-beta-hydroxysteroid dehydrogenase 1B (LOC106388464), mRNA ; PREDICTED: Brassica oleracea var. oleracea 11-beta-hydroxysteroid dehydrogenase 1B-like (LOC106333477), mRNA ; PREDICTED: Brassica rapa 11-beta-hydroxysteroid dehydrogenase 1B-like (LOC103858452), mRNA ; PREDICTED: Raphanus sativus 11-beta-hydroxysteroid dehydrogenase 1B-like (LOC108846709), mRNA ; Similar to HSD1 11-beta-hydroxysteroid dehydrogenase 1A (Arabidopsis thaliana)::Bra009034:0-341 | Bra009034 | 38 kDa | 46 | 44 | 8 | 37 |
| 146 | PREDICTED: Brassica napus plastid-lipid-associated protein 6, chloroplastic (LOC106357886), transcript variant X1, mRNA ; PREDICTED: Brassica napus plastid-lipid-associated protein 6, chloroplastic (LOC106357886), transcript variant X2, mRNA ; PREDICTED: Brassica rapa probable plastid-lipid-associated protein 6, chloroplastic (LOC103828744), mRNA ; Similar to PAP6 Probable plastid-lipid-associated protein 6, chloroplastic (Arabidopsis thaliana)::Bra024890:0-277 | Bra024890 | 30 kDa | 11 | 8 | 2 | 8 |
| 147 | PREDICTED: Brassica napus protein disulfide-isomerase like 2-1 (LOC106438331), transcript variant X1, mRNA ; PREDICTED: Brassica napus protein disulfide-isomerase like 2-1 (LOC106438331), transcript variant X2, mRNA ; PREDICTED: Brassica napus protein disulfide-isomerase like 2-1 (LOC106438331), transcript variant X3, mRNA ; PREDICTED: Brassica oleracea var. oleracea protein disulfide-isomerase like 2-1-like (LOC106328378), mRNA ; PREDICTED: Brassica rapa protein disulfide-isomerase like 2-1 (LOC103858248), mRNA ; Similar to PDIL2-1 Protein disulfide-isomerase like 2-1 (Arabidopsis thaliana)::Bra008871:0-397 | Bra008871 | 44 kDa | 6 | 13 | 2 | 9 |
| 148 | PREDICTED: Brassica napus nascent polypeptide-associated complex subunit alpha-like protein 1 (LOC106351941), mRNA ; PREDICTED: Brassica napus nascent polypeptide-associated complex subunit alpha-like protein 1 (LOC106436932), mRNA ; PREDICTED: Brassica oleracea var. oleracea nascent polypeptide-associated complex subunit alpha-like protein 1 (LOC106341702), mRNA ; PREDICTED: Brassica rapa nascent polypeptide-associated complex subunit alpha-like protein 1 (LOC103845577), mRNA ; Similar to At3g12390 Nascent polypeptide-associated complex subunit alpha-like protein 1 (Arabidopsis thaliana)::Bra015175:0-172 | Bra015175 | 19 kDa | 7 | 8 | 7 | 6 |
| 149 | PREDICTED: Brassica napus late embryogenesis abundant protein 76 (LOC111200976), transcript variant X1, mRNA ; Similar to ECU03_1610 Uncharacterized protein ECU03_1610 (Encephalitozoon cuniculi (strain GB-M1))::Bra001919:0-351 | Bra001919 | 40 kDa | 8 | 5 | 13 | 3 |
| 150 | Brassica rapa peroxiredoxin-2B (LOC103852305), mRNA ; Brassica rapa subsp. pekinensis type 2 peroxiredoxin (PrxII) mRNA, complete cds ; PREDICTED: Brassica napus peroxiredoxin-2B-like (LOC106441213), mRNA ; PREDICTED: Brassica napus peroxiredoxin-2B (LOC106381333), mRNA ; PREDICTED: Brassica oleracea var. oleracea peroxiredoxin-2B (LOC106322672), mRNA ; Similar to PRXIIB Peroxiredoxin-2B (Arabidopsis thaliana)::Bra017320:0-162 | Bra017320 | 17 kDa | 7 | 8 | 4 | 9 |
| 151 | PREDICTED: Brassica rapa 40S ribosomal protein S24-1 (LOC103837315), mRNA ; Similar to RPS24A 40S ribosomal protein S24-1 (Arabidopsis thaliana)::Bra000338:0-133 | Bra000338 (+2) | 15 kDa | 7 | 10 | 12 | 4 |
| 152 | Brassica rapa genome, scaffold: A06 ; Brassica rapa subsp. pekinensis epithiospecifier protein (ESP) mRNA, complete cds ; PREDICTED: Brassica napus epithiospecifier protein-like (LOC111207173), mRNA ; PREDICTED: Brassica rapa epithiospecifier protein-like (LOC103827899), mRNA ; Similar to ESP Epithiospecifier protein (Arabidopsis thaliana)::Bra023756:0-357 | Bra023756 | 39 kDa | 20 | 6 | 2 | 1 |
| 153 | Brassica napus probable mediator of RNA polymerase II transcription subunit 37e (LOC106361615), mRNA ; Brassica oleracea HDEM genome, scaffold: C4 ; Brassica oleracea HDEM genome, scaffold: C9 ; Brassica rapa genome, scaffold: A10 ; Brassica rapa genome, scaffold: A10 ; PREDICTED: Brassica napus probable mediator of RNA polymerase II transcription subunit 37e (LOC106361614), mRNA ; PREDICTED: Brassica napus probable mediator of RNA polymerase II transcription subunit 37e (LOC106372589), misc_RNA ; PREDICTED: Brassica oleracea var. oleracea probable mediator of RNA polymerase II transcription subunit 37e (LOC106317538), mRNA ; PREDICTED: Brassica oleracea var. oleracea probable mediator of RNA polymerase II transcription subunit 37e (LOC106341975), mRNA ; PREDICTED: Brassica rapa probable mediator of RNA polymerase II transcription subunit 37e (LOC103847444), mRNA ; Similar to MED37E Probable mediator of RNA polymerase II transcription sub::Bra037958:0-634 | Bra037958 (+1) | 70 kDa | 41 | 26 | 20 | 22 |
| 154 | PREDICTED: Brassica napus SNF1-related protein kinase regulatory subunit gamma-like PV42a (LOC106346666), transcript variant X1, mRNA ; PREDICTED: Brassica napus SNF1-related protein kinase regulatory subunit gamma-like PV42a (LOC106346666), transcript variant X2, mRNA ; PREDICTED: Brassica napus SNF1-related protein kinase regulatory subunit gamma-like PV42a (LOC106452179), mRNA ; PREDICTED: Brassica oleracea var. oleracea SNF1-related protein kinase regulatory subunit gamma-like PV42a (LOC106292693), mRNA ; PREDICTED: Brassica rapa SNF1-related protein kinase regulatory subunit gamma-like PV42a (LOC103872324), mRNA ; Similar to PV42A SNF1-related protein kinase regulatory subunit gamma-like PV42a (Arabidopsis thaliana)::Bra021133:0-355 | Bra021133 | 39 kDa | 17 | 5 | 1 | 5 |
| 155 | PREDICTED: Brassica napus S-formylglutathione hydrolase-like (LOC106396216), mRNA ; PREDICTED: Brassica napus S-formylglutathione hydrolase-like (LOC106450663), mRNA ; PREDICTED: Brassica rapa S-formylglutathione hydrolase-like (LOC103866563), mRNA ; Similar to SFGH S-formylglutathione hydrolase (Arabidopsis thaliana)::Bra027961:0-281 | Bra027961 | 31 kDa | 7 | 14 | 0 | 7 |
| 156 | Similar to Acyl-CoA-binding protein (Brassica napus)::Bra030139:0-92 | Bra030139 | 10 kDa | 12 | 8 | 4 | 4 |
| 157 | PREDICTED: Brassica napus reticulon-like protein B1 (LOC106438459), transcript variant X1, mRNA ; PREDICTED: Brassica napus reticulon-like protein B1 (LOC106438459), transcript variant X2, misc_RNA ; PREDICTED: Brassica rapa reticulon-like protein B1 (LOC103861418), mRNA ; Similar to RTNLB1 Reticulon-like protein B1 (Arabidopsis thaliana)::Bra011059:0-268 | Bra011059 (+2) | 30 kDa | 8 | 7 | 1 | 8 |
| 158 | Brassica napus seed specific protein Bn15D33A mRNA, complete cds ; PREDICTED: Brassica napus uncharacterized BNAA02G24090D (BNAA02G24090D), mRNA ; PREDICTED: Brassica napus uncharacterized LOC106379664 (LOC106379664), mRNA ; PREDICTED: Brassica rapa uncharacterized LOC103853665 (LOC103853665), mRNA ; Protein of unknown function::Bra018166:0-116 | Bra018166 (+1) | 13 kDa | 17 | 8 | 0 | 3 |
| 159 | PREDICTED: Brassica napus 40S ribosomal protein S4-1-like (LOC106411885), mRNA ; PREDICTED: Brassica napus 40S ribosomal protein S4-1-like (LOC106426312), mRNA ; PREDICTED: Brassica napus 40S ribosomal protein S4-1-like (LOC106429399), mRNA ; PREDICTED: Brassica rapa 40S ribosomal protein S4-1-like (LOC103855714), mRNA ; Similar to RPS4A 40S ribosomal protein S4-1 (Arabidopsis thaliana)::Bra006848:0-279 | Bra006848 (+4) | 32 kDa | 8 | 10 | 4 | 5 |
| 160 | Similar to pyrH Uridylate kinase (Fusobacterium nucleatum subsp. nucleatum (strain ATCC 25586 / CIP 101130 / JCM 8532 / LMG 13131))::Bra031240:0-703 | Bra031240 | 77 kDa | 2 | 10 | 7 | 4 |
| 161 | Brassica oleracea HDEM genome, scaffold: C1 ; Brassica rapa genome, scaffold: A01 ; PREDICTED: Brassica napus uncharacterized BNAA01G05030D (BNAA01G05030D), mRNA ; PREDICTED: Brassica napus uncharacterized LOC106371401 (LOC106371401), mRNA ; PREDICTED: Brassica napus uncharacterized LOC106375395 (LOC106375395), mRNA ; PREDICTED: Brassica oleracea var. oleracea uncharacterized LOC106303707 (LOC106303707), mRNA ; PREDICTED: Brassica rapa uncharacterized LOC103851502 (LOC103851502), mRNA ; Protein of unknown function::Bra011941:0-100 | Bra011941 | 11 kDa | 10 | 12 | 5 | 0 |
| 162 | PREDICTED: Brassica napus uncharacterized LOC106365887 (LOC106365887), transcript variant X1, mRNA ; PREDICTED: Brassica napus uncharacterized LOC106365887 (LOC106365887), transcript variant X2, mRNA ; PREDICTED: Brassica napus uncharacterized LOC106440359 (LOC106440359), transcript variant X1, mRNA ; PREDICTED: Brassica napus uncharacterized LOC106440359 (LOC106440359), transcript variant X3, misc_RNA ; PREDICTED: Brassica oleracea var. oleracea uncharacterized LOC106316619 (LOC106316619), mRNA ; PREDICTED: Brassica rapa uncharacterized LOC103837119 (LOC103837119), mRNA ; Protein of unknown function::Bra000417:0-346 | Bra000417 | 40 kDa | 24 | 2 | 0 | 1 |
| 163 | PREDICTED: Brassica napus 60S ribosomal protein L7-2 (LOC106381922), mRNA ; PREDICTED: Brassica napus 60S ribosomal protein L7-2 (LOC106388681), mRNA ; PREDICTED: Brassica oleracea var. oleracea 60S ribosomal protein L7-2 (LOC106322552), mRNA ; PREDICTED: Brassica rapa 60S ribosomal protein L7-2 (LOC103832784), mRNA ; Similar to RPL7B 60S ribosomal protein L7-2 (Arabidopsis thaliana)::Bra019175:0-242 | Bra019175 (+1) | 28 kDa | 9 | 11 | 4 | 1 |
| 164 | PREDICTED: Brassica napus ATP synthase subunit beta-1, mitochondrial-like (LOC106380543), transcript variant X1, mRNA ; PREDICTED: Brassica napus ATP synthase subunit beta-1, mitochondrial-like (LOC106380543), transcript variant X2, mRNA ; PREDICTED: Brassica napus ATP synthase subunit beta-1, mitochondrial-like (LOC106396303), mRNA ; PREDICTED: Brassica napus ATP synthase subunit beta-1, mitochondrial-like (LOC106396307), mRNA ; PREDICTED: Brassica napus ATP synthase subunit beta-2, mitochondrial-like (LOC106380544), mRNA ; PREDICTED: Brassica napus ATP synthase subunit beta-2, mitochondrial (LOC106377686), mRNA ; PREDICTED: Brassica oleracea var. oleracea ATP synthase subunit beta-1, mitochondrial-like (LOC106327128), mRNA ; PREDICTED: Brassica oleracea var. oleracea ATP synthase subunit beta-1, mitochondrial (LOC106327662), mRNA ; PREDICTED: Brassica rapa ATP synthase subunit beta-1, mitochondrial-like (LOC103850708), misc_RNA ; PREDIC::Bra000218:0-533 | Bra000218 (+6) | 57 kDa | 12 | 5 | 3 | 5 |
| 165 | Brassica juncea mRNA for O-acetylserine(thiol) lyase, clone OAS-TL4 ; Brassica rapa subsp. chinensis O-acetylserine(thiol)lyase isoform A4 (OASA4) mRNA, complete cds ; PREDICTED: Brassica napus cysteine synthase-like (LOC106435443), transcript variant X1, mRNA ; PREDICTED: Brassica napus cysteine synthase-like (LOC106435443), transcript variant X2, mRNA ; PREDICTED: Brassica napus cysteine synthase (LOC106360617), transcript variant X1, mRNA ; PREDICTED: Brassica napus cysteine synthase (LOC106360617), transcript variant X2, mRNA ; PREDICTED: Brassica oleracea var. oleracea cysteine synthase (LOC106307369), mRNA ; PREDICTED: Brassica rapa cysteine synthase (OASA4), transcript variant X1, mRNA ; PREDICTED: Brassica rapa cysteine synthase (OASA4), transcript variant X2, mRNA ; Similar to Cysteine synthase (Brassica juncea)::Bra041810:0-322 | Bra041810 | 34 kDa | 9 | 13 | 1 | 3 |
| 166 | PREDICTED: Brassica napus 60S ribosomal protein L10-3-like (LOC106406321), mRNA ; PREDICTED: Brassica napus 60S ribosomal protein L10-3-like (LOC106432562), mRNA ; PREDICTED: Brassica oleracea var. oleracea 60S ribosomal protein L10-3-like (LOC106300530), mRNA ; PREDICTED: Brassica rapa 60S ribosomal protein L10-3-like (LOC103831514), mRNA ; Similar to RPL10C 60S ribosomal protein L10-3 (Arabidopsis thaliana)::Bra027071:0-220 | Bra027071 | 25 kDa | 10 | 9 | 4 | 2 |
| 167 | Similar to At1g47710 Serpin-ZX (Arabidopsis thaliana)::Bra036377:0-550 | Bra036377 | 61 kDa | 12 | 7 | 0 | 5 |
| 168 | Similar to SUS3 Sucrose synthase 3 (Arabidopsis thaliana)::Bra000059:0-822 | Bra000059 | 94 kDa | 19 | 5 | 0 | 1 |
| 169 | PREDICTED: Brassica napus 40S ribosomal protein S13-2 (LOC106411904), mRNA ; PREDICTED: Brassica napus 40S ribosomal protein S13-2 (LOC106412000), mRNA ; PREDICTED: Brassica oleracea var. oleracea 40S ribosomal protein S13-2 (LOC106309894), mRNA ; PREDICTED: Brassica rapa 40S ribosomal protein S13-2 (LOC103841918), mRNA ; Similar to RPS13B 40S ribosomal protein S13-2 (Arabidopsis thaliana) ; Zea mays clone 11839 mRNA sequence::Bra005344:0-151 | Bra005344 (+4) | 17 kDa | 7 | 7 | 4 | 6 |
| 170 | Similar to PIN5 Putative auxin efflux carrier component 5 (Arabidopsis thaliana)::Bra037288:0-653 | Bra037288 | 71 kDa | 8 | 8 | 4 | 4 |
| 171 | Protein of unknown function::Bra017629:0-127 | Bra017629 | 14 kDa | 4 | 5 | 12 | 3 |
| 172 | Similar to ANN1 Annexin D1 (Arabidopsis thaliana)::Bra029908:0-370 | Bra029908 | 42 kDa | 12 | 5 | 6 | 1 |
| 173 | Similar to UREG Urease accessory protein G (Arabidopsis thaliana)::Bra040534:0-435 | Bra040534 | 49 kDa | 4 | 10 | 4 | 1 |
| 174 | PREDICTED: Brassica napus germin-like protein subfamily 2 member 1 (LOC106419894), mRNA ; PREDICTED: Brassica rapa germin-like protein subfamily 2 member 1 (LOC103843347), mRNA ; Similar to GLP4 Germin-like protein subfamily 2 member 1 (Arabidopsis thaliana)::Bra006300:0-216 | Bra006300 | 23 kDa | 8 | 9 | 4 | 2 |
| 175 | Similar to RPL18B 60S ribosomal protein L18-2 (Arabidopsis thaliana)::Bra019708:0-397 | Bra019708 (+1) | 44 kDa | 5 | 4 | 5 | 7 |
| 176 | Brassica juncea clone 575 60S ribosomal protein L13-2 mRNA, partial sequence ; Brassica napus 60S ribosomal protein L13-1 (LOC106447867), mRNA ; PREDICTED: Brassica napus 60S ribosomal protein L13-1 (LOC106447867), transcript variant X1, mRNA ; PREDICTED: Brassica napus 60S ribosomal protein L13-2-like (LOC111197723), transcript variant X1, mRNA ; PREDICTED: Brassica napus 60S ribosomal protein L13-2-like (LOC111197723), transcript variant X2, mRNA ; PREDICTED: Brassica oleracea var. oleracea 60S ribosomal protein L13-2-like (LOC106311292), mRNA ; PREDICTED: Brassica rapa 60S ribosomal protein L13-1 (LOC103874976), mRNA ; Similar to 60S ribosomal protein L13-1 (Brassica napus) ; Zea mays clone 14550 mRNA sequence::Bra013909:0-206 | Bra013909 (+1) | 24 kDa | 7 | 8 | 3 | 1 |
| 177 | PREDICTED: Brassica napus 40S ribosomal protein S6-2 (LOC106384903), mRNA ; PREDICTED: Brassica napus 40S ribosomal protein S6-2 (LOC106430360), mRNA ; PREDICTED: Brassica napus 40S ribosomal protein S6-2 (LOC106430367), mRNA ; PREDICTED: Brassica oleracea var. oleracea 40S ribosomal protein S6-2 (LOC106328438), mRNA ; PREDICTED: Brassica rapa 40S ribosomal protein S6-2 (LOC103855860), mRNA ; Similar to RPS6B 40S ribosomal protein S6-2 (Arabidopsis thaliana) ; Zea mays clone 13559 mRNA sequence::Bra006969:0-249 | Bra006969 | 28 kDa | 4 | 2 | 1 | 9 |
| 178 | PREDICTED: Brassica napus aspartyl protease AED3 (LOC106431380), mRNA ; PREDICTED: Brassica napus aspartyl protease AED3 (LOC106445461), mRNA ; PREDICTED: Brassica oleracea var. oleracea protein ASPARTIC PROTEASE IN GUARD CELL 2-like (LOC106342769), mRNA ; PREDICTED: Brassica rapa aspartyl protease AED3-like (LOC103863237), mRNA ; Protein of unknown function::Bra038558:0-466 | Bra038558 | 50 kDa | 4 | 8 | 3 | 4 |
| 179 | Similar to DJ1A Protein DJ-1 homolog A (Arabidopsis thaliana)::Bra030915:0-407 | Bra030915 | 43 kDa | 9 | 8 | 1 | 4 |
| 180 | PREDICTED: Brassica napus ADP,ATP carrier protein 3, mitochondrial-like (LOC106361198), transcript variant X1, mRNA ; PREDICTED: Brassica napus ADP,ATP carrier protein 3, mitochondrial-like (LOC106361198), transcript variant X2, mRNA ; PREDICTED: Brassica napus ADP,ATP carrier protein 3, mitochondrial-like (LOC106361198), transcript variant X3, mRNA ; PREDICTED: Brassica rapa ADP,ATP carrier protein 3, mitochondrial (LOC103834762), transcript variant X1, mRNA ; PREDICTED: Brassica rapa ADP,ATP carrier protein 3, mitochondrial (LOC103834762), transcript variant X2, mRNA ; Similar to AAC3 ADP,ATP carrier protein 3, mitochondrial (Arabidopsis thaliana)::Bra033682:0-402 | Bra033682 | 44 kDa | 13 | 5 | 0 | 4 |
| 181 | Similar to RPS25E 40S ribosomal protein S25-4 (Arabidopsis thaliana)::Bra011385:0-108 | Bra011385 (+2) | 12 kDa | 0 | 10 | 5 | 7 |
| 182 | Brassica oleracea HDEM genome, scaffold: C4 ; Brassica rapa genome, scaffold: A05 ; PREDICTED: Brassica napus heat shock 70 kDa protein 8-like (LOC106451327), mRNA ; PREDICTED: Brassica napus heat shock 70 kDa protein 8 (LOC106396657), transcript variant X1, mRNA ; PREDICTED: Brassica napus heat shock 70 kDa protein 8 (LOC106396657), transcript variant X2, mRNA ; PREDICTED: Brassica oleracea var. oleracea heat shock 70 kDa protein 8 (LOC106342916), transcript variant X1, mRNA ; PREDICTED: Brassica oleracea var. oleracea heat shock 70 kDa protein 8 (LOC106342916), transcript variant X2, mRNA ; PREDICTED: Brassica rapa heat shock 70 kDa protein 8 (LOC103867668), mRNA ; Similar to HSP70-8 Heat shock 70 kDa protein 8 (Arabidopsis thaliana)::Bra028928:0-564 | Bra028928 | 61 kDa | 11 | 8 | 0 | 3 |
| 183 | PREDICTED: Brassica napus universal stress protein PHOS34 (LOC106410769), mRNA ; PREDICTED: Brassica oleracea var. oleracea universal stress protein YxiE-like (LOC106296807), mRNA ; Protein of unknown function ; Zea mays clone 13463 mRNA sequence ; Zea mays clone 14034 mRNA sequence::Bra025973:0-159 | Bra025973 | 18 kDa | 8 | 11 | 0 | 3 |
| 184 | PREDICTED: Brassica napus early nodulin-like protein 2 (LOC106376112), mRNA ; PREDICTED: Brassica oleracea var. oleracea early nodulin-like protein 2 (LOC106327489), mRNA ; PREDICTED: Brassica rapa early nodulin-like protein 2 (LOC103867244), mRNA ; Similar to At4g27520 Early nodulin-like protein 2 (Arabidopsis thaliana)::Bra013070:0-328 | Bra013070 | 33 kDa | 3 | 8 | 6 | 2 |
| 185 | PREDICTED: Brassica napus GDSL esterase/lipase At3g09930 (LOC111213910), mRNA ; PREDICTED: Brassica oleracea var. oleracea GDSL esterase/lipase At3g09930 (LOC106292495), mRNA ; PREDICTED: Brassica rapa GDSL esterase/lipase At3g09930 (LOC103870502), mRNA ; Similar to At3g09930 GDSL esterase/lipase At3g09930 (Arabidopsis thaliana)::Bra031244:0-354 | Bra031244 | 40 kDa | 1 | 8 | 6 | 4 |
| 186 | PREDICTED: Brassica napus low-temperature-induced 65 kDa protein-like (LOC106440851), transcript variant X1, mRNA ; PREDICTED: Brassica rapa low-temperature-induced 65 kDa protein (LOC103861574), mRNA ; Similar to LTI65 Low-temperature-induced 65 kDa protein (Arabidopsis thaliana)::Bra011172:0-559 | Bra011172 | 60 kDa | 7 | 7 | 6 | 1 |
| 187 | Similar to LRP 40S ribosomal protein SA (Brassica napus)::Bra017597:0-296 | Bra017597 (+4) | 32 kDa | 7 | 7 | 2 | 3 |
| 188 | PREDICTED: Brassica napus uncharacterized LOC106346992 (LOC106346992), transcript variant X3, mRNA ; PREDICTED: Brassica napus uncharacterized LOC106402967 (LOC106402967), transcript variant X2, mRNA ; PREDICTED: Brassica oleracea var. oleracea uncharacterized LOC106324852 (LOC106324852), transcript variant X3, mRNA ; Protein of unknown function::Bra017274:0-233 | Bra017274 | 25 kDa | 1 | 5 | 10 | 4 |
| 189 | PREDICTED: Brassica napus dehydrin Xero 1-like (LOC106454853), mRNA ; PREDICTED: Brassica napus dehydrin Xero 1 (LOC106399795), transcript variant X1, mRNA ; PREDICTED: Brassica oleracea var. oleracea dehydrin Xero 1-like (LOC106311856), mRNA ; PREDICTED: Brassica rapa dehydrin Xero 1-like (LOC103841019), mRNA ; Similar to XERO1 Dehydrin Xero 1 (Arabidopsis thaliana)::Bra004681:0-134 | Bra004681 | 14 kDa | 6 | 6 | 7 | 0 |
| 190 | Similar to MBP2 Myrosinase-binding protein 2 (Arabidopsis thaliana)::Bra031923:0-334 | Bra031923 | 37 kDa | 16 | 2 | 0 | 3 |
| 191 | PREDICTED: Brassica napus protein disulfide-isomerase like 2-1-like (LOC106450433), mRNA ; PREDICTED: Brassica rapa protein disulfide-isomerase like 2-1 (LOC103866377), transcript variant X1, mRNA ; Similar to PDIL2-1 Protein disulfide-isomerase like 2-1 (Arabidopsis thaliana)::Bra027783:0-361 | Bra027783 | 39 kDa | 9 | 10 | 4 | 10 |
| 192 | PREDICTED: Brassica napus probable pectinesterase/pectinesterase inhibitor 36 (LOC106414961), mRNA ; PREDICTED: Brassica oleracea var. oleracea probable pectinesterase/pectinesterase inhibitor 36 (LOC106311324), mRNA ; PREDICTED: Brassica rapa probable pectinesterase/pectinesterase inhibitor 36 (LOC103841916), transcript variant X1, misc_RNA ; PREDICTED: Brassica rapa probable pectinesterase/pectinesterase inhibitor 36 (LOC103841916), transcript variant X2, mRNA ; Similar to PME36 Probable pectinesterase/pectinesterase inhibitor 36 (Arabidopsis thaliana)::Bra005342:0-521 | Bra005342 | 58 kDa | 1 | 8 | 3 | 6 |
| 193 | PREDICTED: Brassica napus dnaJ homolog subfamily B member 13 (LOC106368780), mRNA ; PREDICTED: Brassica napus dnaJ homolog subfamily B member 13 (LOC106436699), mRNA ; PREDICTED: Brassica oleracea var. oleracea dnaJ homolog subfamily B member 13 (LOC106312462), mRNA ; PREDICTED: Brassica rapa dnaJ homolog subfamily B member 13 (LOC103842598), mRNA ; Similar to DNAJB6 DnaJ homolog subfamily B member 6 (Homo sapiens)::Bra005828:0-338 | Bra005828 | 37 kDa | 7 | 8 | 4 | 1 |
| 194 | PREDICTED: Brassica napus uncharacterized LOC106395796 (LOC106395796), mRNA ; PREDICTED: Brassica napus uncharacterized LOC106433462 (LOC106433462), mRNA ; PREDICTED: Brassica napus uncharacterized LOC106433482 (LOC106433482), mRNA ; PREDICTED: Brassica oleracea var. oleracea uncharacterized LOC106325614 (LOC106325614), mRNA ; PREDICTED: Brassica rapa uncharacterized LOC103852751 (LOC103852751), mRNA ; Protein of unknown function::Bra017618:0-459 | Bra017618 | 51 kDa | 2 | 9 | 6 | 2 |
| 195 | PREDICTED: Brassica napus 60S ribosomal protein L6-1-like (LOC106369059), mRNA ; PREDICTED: Brassica napus 60S ribosomal protein L6-1 (LOC106436769), mRNA ; PREDICTED: Brassica oleracea var. oleracea 60S ribosomal protein L6-1 (LOC106309544), mRNA ; PREDICTED: Brassica rapa 60S ribosomal protein L6-1 (LOC103842696), mRNA ; Similar to RPL6A 60S ribosomal protein L6-1 (Arabidopsis thaliana)::Bra005912:0-234 | Bra005912 | 26 kDa | 1 | 9 | 7 | 1 |
| 196 | PREDICTED: Brassica napus desiccation-related protein PCC13-62-like (LOC106412444), misc_RNA ; PREDICTED: Brassica napus desiccation-related protein PCC13-62-like (LOC106429407), misc_RNA ; PREDICTED: Brassica napus desiccation-related protein PCC13-62 (LOC106420540), mRNA ; PREDICTED: Brassica oleracea var. oleracea desiccation-related protein PCC13-62 (LOC106310506), mRNA ; PREDICTED: Brassica rapa desiccation-related protein PCC13-62 (LOC103842077), mRNA ; Similar to Desiccation-related protein PCC13-62 (Craterostigma plantagineum)::Bra005466:0-311 | Bra005466 | 34 kDa | 7 | 4 | 5 | 4 |
| 197 | PREDICTED: Brassica napus probable elongation factor 1-gamma 1 (LOC106420176), mRNA ; PREDICTED: Brassica rapa probable elongation factor 1-gamma 1 (LOC103843341), mRNA ; Similar to At1g09640 Probable elongation factor 1-gamma 1 (Arabidopsis thaliana)::Bra006296:0-413 | Bra006296 | 46 kDa | 6 | 8 | 3 | 2 |
| 198 | Similar to At3g53970 Probable proteasome inhibitor (Arabidopsis thaliana)::Bra025972:0-325 | Bra025972 | 35 kDa | 6 | 5 | 3 | 4 |
| 199 | PREDICTED: Brassica napus transketolase-1, chloroplastic-like (LOC106414826), mRNA ; PREDICTED: Brassica napus transketolase-1, chloroplastic-like (LOC106414846), mRNA ; PREDICTED: Brassica oleracea var. oleracea transketolase-1, chloroplastic (LOC106311760), mRNA ; PREDICTED: Brassica rapa transketolase-1, chloroplastic (LOC103841917), mRNA ; Similar to TKL-1 Transketolase-1, chloroplastic (Arabidopsis thaliana)::Bra005343:0-736 | Bra005343 (+1) | 79 kDa | 7 | 8 | 0 | 3 |
| 200 | Similar to APA1 Aspartic proteinase A1 (Arabidopsis thaliana)::Bra006206:0-588 | Bra006206 | 64 kDa | 6 | 6 | 0 | 5 |
| 201 | PREDICTED: Brassica napus probable inactive serine/threonine-protein kinase fnkC (LOC106389282), transcript variant X1, mRNA ; PREDICTED: Brassica napus probable inactive serine/threonine-protein kinase fnkC (LOC106389282), transcript variant X2, mRNA ; PREDICTED: Brassica napus probable inactive serine/threonine-protein kinase fnkC (LOC106424470), transcript variant X1, mRNA ; PREDICTED: Brassica napus probable inactive serine/threonine-protein kinase fnkC (LOC106424470), transcript variant X2, mRNA ; PREDICTED: Brassica rapa probable inactive serine/threonine-protein kinase fnkC (LOC103859852), transcript variant X1, mRNA ; PREDICTED: Brassica rapa probable inactive serine/threonine-protein kinase fnkC (LOC103859852), transcript variant X2, mRNA ; Protein of unknown function::Bra010074:0-382 | Bra010074 | 44 kDa | 11 | 0 | 0 | 0 |
| 202 | Similar to At5g07830 Heparanase-like protein 1 (Arabidopsis thaliana)::Bra037650:0-525 | Bra037650 | 58 kDa | 2 | 6 | 5 | 3 |
| 203 | Similar to At1g23190 Probable phosphoglucomutase, cytoplasmic 1 (Arabidopsis thaliana)::Bra034284:0-583 | Bra034284 | 63 kDa | 6 | 9 | 1 | 1 |
| 204 | PREDICTED: Brassica napus vicilin-like seed storage protein At2g18540 (LOC106348195), mRNA ; PREDICTED: Brassica napus vicilin-like seed storage protein At2g18540 (LOC106385437), mRNA ; PREDICTED: Brassica oleracea var. oleracea nuclear speckle splicing regulatory protein 1 (LOC106333178), mRNA ; PREDICTED: Brassica rapa vicilin-like seed storage protein At2g18540 (LOC103874069), mRNA ; Similar to At2g18540 Vicilin-like seed storage protein At2g18540 (Arabidopsis thaliana)::Bra022682:0-697 | Bra022682 (+2) | 83 kDa | 9 | 3 | 2 | 1 |
| 205 | PREDICTED: Brassica napus uncharacterized BNACNNG75970D (BNACNNG75970D), mRNA ; PREDICTED: Brassica napus uncharacterized LOC106356495 (LOC106356495), mRNA ; PREDICTED: Brassica napus uncharacterized LOC106358345 (LOC106358345), mRNA ; PREDICTED: Brassica oleracea var. oleracea uncharacterized LOC106306577 (LOC106306577), mRNA ; PREDICTED: Brassica rapa uncharacterized LOC103829566 (LOC103829566), mRNA ; Protein of unknown function::Bra025598:0-117 | Bra025598 | 13 kDa | 8 | 6 | 2 | 0 |
| 206 | Similar to ADH2 Alcohol dehydrogenase class-3 (Arabidopsis thaliana)::Bra001897:0-399 | Bra001897 (+1) | 43 kDa | 5 | 6 | 0 | 5 |
| 207 | Similar to EMB2247 Valine--tRNA ligase, chloroplastic/mitochondrial 2 (Arabidopsis thaliana)::Bra037183:0-1183 | Bra037183 | 134 kDa | 0 | 8 | 8 | 0 |
| 208 | Similar to RRP5 rRNA biogenesis protein RRP5 (Arabidopsis thaliana)::Bra015151:0-2134 | Bra015151 | 237 kDa | 4 | 9 | 1 | 1 |
| 209 | PREDICTED: Brassica napus 60S ribosomal protein L8-3-like (LOC106451593), mRNA ; PREDICTED: Brassica napus 60S ribosomal protein L8-3-like (LOC111215659), mRNA ; PREDICTED: Brassica oleracea var. oleracea 60S ribosomal protein L8-3-like (LOC106307103), mRNA ; PREDICTED: Brassica rapa 60S ribosomal protein L8-3-like (LOC103862457), mRNA ; Similar to RPL8C 60S ribosomal protein L8-3 (Arabidopsis thaliana)::Bra003034:0-258 | Bra003034 (+2) | 28 kDa | 2 | 3 | 2 | 0 |
| 210 | PREDICTED: Brassica napus heat shock 70 kDa protein 14-like (LOC106345936), transcript variant X1, mRNA ; PREDICTED: Brassica napus heat shock 70 kDa protein 14-like (LOC106345936), transcript variant X2, mRNA ; PREDICTED: Brassica rapa heat shock 70 kDa protein 14 (LOC103871120), transcript variant X1, mRNA ; PREDICTED: Brassica rapa heat shock 70 kDa protein 14 (LOC103871120), transcript variant X2, mRNA ; Similar to HSP70-14 Heat shock 70 kDa protein 14 (Arabidopsis thaliana)::Bra020199:0-818 | Bra020199 | 90 kDa | 6 | 5 | 0 | 4 |
| 211 | PREDICTED: Brassica napus 3-oxoacyl-[acyl-carrier-protein] synthase I, chloroplastic-like (LOC106381762), transcript variant X1, mRNA ; PREDICTED: Brassica napus 3-oxoacyl-[acyl-carrier-protein] synthase I, chloroplastic-like (LOC106381763), transcript variant X1, mRNA ; PREDICTED: Brassica napus 3-oxoacyl-[acyl-carrier-protein] synthase I, chloroplastic (LOC106411349), mRNA ; PREDICTED: Brassica rapa 3-oxoacyl-[acyl-carrier-protein] synthase I, chloroplastic-like (LOC103853696), mRNA ; Similar to KAS1 3-oxoacyl-[acyl-carrier-protein] synthase I, chloroplastic (Arabidopsis thaliana)::Bra018214:0-521 | Bra018214 | 56 kDa | 10 | 4 | 0 | 1 |
| 212 | Brassica napus enoyl-[acyl-carrier-protein] reductase [NADH], chloroplastic-like (LOC106439777), mRNA ; PREDICTED: Brassica napus enoyl-[acyl-carrier-protein] reductase [NADH], chloroplastic-like (LOC106439776), mRNA ; PREDICTED: Brassica napus enoyl-[acyl-carrier-protein] reductase [NADH], chloroplastic-like (LOC106439777), transcript variant X1, mRNA ; PREDICTED: Brassica napus enoyl-[acyl-carrier-protein] reductase [NADH], chloroplastic-like (LOC106439777), transcript variant X2, mRNA ; Similar to Enoyl-[acyl-carrier-protein] reductase [NADH], chloroplastic (Brassica napus)::Bra010588:0-394 | Bra010588 | 42 kDa | 5 | 6 | 0 | 4 |
| 213 | Brassica oleracea var. alboglabra S-adenosyl-L-homocystein hydrolase mRNA, complete cds ; PREDICTED: Brassica napus adenosylhomocysteinase 1-like (LOC106399668), transcript variant X1, mRNA ; PREDICTED: Brassica napus adenosylhomocysteinase 1-like (LOC106437676), transcript variant X1, mRNA ; PREDICTED: Brassica oleracea var. oleracea adenosylhomocysteinase 1-like (LOC106311804), transcript variant X1, mRNA ; Similar to SAHH1 Adenosylhomocysteinase 1 (Arabidopsis thaliana)::Bra032371:0-485 | Bra032371 (+1) | 53 kDa | 6 | 6 | 0 | 3 |
| 214 | Similar to PIMT2 Protein-L-isoaspartate O-methyltransferase 2 (Arabidopsis thaliana)::Bra009023:0-301 | Bra009023 | 32 kDa | 6 | 6 | 0 | 3 |
| 215 | PREDICTED: Brassica napus probable protein phosphatase 2C 39 (LOC106397444), mRNA ; PREDICTED: Brassica napus probable protein phosphatase 2C 39 (LOC106403039), mRNA ; PREDICTED: Brassica oleracea var. oleracea probable protein phosphatase 2C 39 (LOC106344001), mRNA ; PREDICTED: Brassica rapa probable protein phosphatase 2C 39 (LOC103869944), mRNA ; Similar to At3g15260 Probable protein phosphatase 2C 39 (Arabidopsis thaliana)::Bra030903:0-288 | Bra030903 | 31 kDa | 5 | 9 | 0 | 1 |
| 216 | PREDICTED: Brassica napus 60S ribosomal protein L23a-1 (LOC106438085), mRNA ; PREDICTED: Brassica rapa 60S ribosomal protein L23a-1-like (LOC103857868), mRNA ; Similar to RPL23AA 60S ribosomal protein L23a-1 (Arabidopsis thaliana)::Bra008599:0-233 | Bra008599 (+3) | 26 kDa | 0 | 2 | 9 | 4 |
| 217 | PREDICTED: Brassica napus probable voltage-gated potassium channel subunit beta (LOC111200420), transcript variant X1, mRNA ; PREDICTED: Brassica napus probable voltage-gated potassium channel subunit beta (LOC111200420), transcript variant X2, mRNA ; PREDICTED: Brassica rapa probable voltage-gated potassium channel subunit beta (LOC103836560), mRNA ; Similar to KAB1 Probable voltage-gated potassium channel subunit beta (Arabidopsis thaliana)::Bra034911:0-328 | Bra034911 (+1) | 37 kDa | 9 | 6 | 0 | 0 |
| 218 | Similar to At4g30910 Leucine aminopeptidase 2, chloroplastic (Arabidopsis thaliana)::Bra039978:0-523 | Bra039978 | 55 kDa | 15 | 0 | 0 | 0 |
| 219 | PREDICTED: Brassica napus 60S ribosomal protein L11-1 (LOC106406958), mRNA ; PREDICTED: Brassica oleracea var. oleracea 60S ribosomal protein L11-1 (LOC106315662), mRNA ; Similar to RPL11A 60S ribosomal protein L11-1 (Arabidopsis thaliana) ; Zea mays clone 19991 mRNA sequence::Bra003588:0-182 | Bra003588 (+6) | 21 kDa | 3 | 2 | 4 | 5 |
| 220 | PREDICTED: Brassica napus 40S ribosomal protein S14-2 (LOC106388134), mRNA ; PREDICTED: Brassica napus 40S ribosomal protein S14-2 (LOC106391301), transcript variant X1, mRNA ; PREDICTED: Brassica napus 40S ribosomal protein S14-2 (LOC106391301), transcript variant X2, mRNA ; PREDICTED: Brassica oleracea var. oleracea 40S ribosomal protein S14-2 (LOC106331850), mRNA ; PREDICTED: Brassica rapa 40S ribosomal protein S14-2 (LOC103857606), mRNA ; Similar to RPS14B 40S ribosomal protein S14-2 (Arabidopsis thaliana)::Bra008277:0-150 | Bra008277 (+4) | 16 kDa | 3 | 4 | 5 | 2 |
| 221 | PREDICTED: Brassica napus non-classical arabinogalactan protein 31-like (LOC106358056), mRNA ; PREDICTED: Brassica rapa non-classical arabinogalactan protein 31-like (LOC103829021), mRNA ; Similar to AGP31 Non-classical arabinogalactan protein 31 (Arabidopsis thaliana)::Bra025193:0-328 | Bra025193 | 35 kDa | 2 | 3 | 7 | 2 |
| 222 | PREDICTED: Brassica napus 40S ribosomal protein S20-1-like (LOC106345888), mRNA ; PREDICTED: Brassica napus 40S ribosomal protein S20-1 (LOC106387934), mRNA ; PREDICTED: Brassica oleracea var. oleracea 40S ribosomal protein S20-1-like (LOC106335881), mRNA ; PREDICTED: Brassica rapa 40S ribosomal protein S20-1-like (LOC103857176), mRNA ; Similar to RPS20A 40S ribosomal protein S20-1 (Arabidopsis thaliana)::Bra007923:0-123 | Bra007923 (+1) | 14 kDa | 4 | 4 | 2 | 4 |
| 223 | PREDICTED: Brassica napus cinnamoyl-CoA reductase 1-like (LOC106367237), mRNA ; PREDICTED: Brassica oleracea var. oleracea cinnamoyl-CoA reductase 1 (LOC106342015), mRNA ; PREDICTED: Brassica rapa cinnamoyl-CoA reductase 1 (LOC103865283), mRNA ; Similar to CCR1 Cinnamoyl-CoA reductase 1 (Arabidopsis thaliana)::Bra040499:0-321 | Bra040499 | 36 kDa | 6 | 7 | 1 | 0 |
| 224 | Similar to MFT Protein MOTHER of FT and TF 1 (Arabidopsis thaliana)::Bra005928:0-173 | Bra005928 | 19 kDa | 5 | 2 | 7 | 0 |
| 225 | PREDICTED: Brassica napus 40S ribosomal protein S19-1-like (LOC106388645), mRNA ; PREDICTED: Brassica napus 40S ribosomal protein S19-1-like (LOC106438916), mRNA ; PREDICTED: Brassica oleracea var. oleracea 40S ribosomal protein S19-1-like (LOC106328670), mRNA ; PREDICTED: Brassica rapa 40S ribosomal protein S19-1-like (LOC103858939), mRNA ; PREDICTED: Raphanus sativus 40S ribosomal protein S19-1-like (LOC108856952), mRNA ; Similar to RPS19A 40S ribosomal protein S19-1 (Arabidopsis thaliana) ; Zea mays clone 9201 mRNA sequence::Bra009396:0-143 | Bra009396 | 16 kDa | 5 | 8 | 1 | 0 |
| 226 | PREDICTED: Brassica oleracea var. oleracea HVA22-like protein b (LOC106326526), mRNA ; Similar to HVA22B HVA22-like protein b (Arabidopsis thaliana)::Bra019937:0-165 | Bra019937 | 19 kDa | 4 | 3 | 1 | 5 |
| 227 | Similar to HSP70-10 Heat shock 70 kDa protein 10, mitochondrial (Arabidopsis thaliana)::Bra015956:0-1090 | Bra015956 | 119 kDa | 12 | 20 | 3 | 13 |
| 228 | Similar to AAC2 ADP,ATP carrier protein 2, mitochondrial (Arabidopsis thaliana)::Bra007113:0-395 | Bra007113 (+2) | 43 kDa | 6 | 5 | 0 | 3 |
| 229 | PREDICTED: Brassica napus peroxiredoxin Q, chloroplastic (LOC106436659), transcript variant X1, mRNA ; PREDICTED: Brassica napus peroxiredoxin Q, chloroplastic (LOC106436659), transcript variant X2, mRNA ; PREDICTED: Brassica oleracea var. oleracea peroxiredoxin Q, chloroplastic (LOC106320257), transcript variant X1, mRNA ; PREDICTED: Brassica oleracea var. oleracea peroxiredoxin Q, chloroplastic (LOC106320257), transcript variant X2, mRNA ; PREDICTED: Brassica rapa peroxiredoxin Q, chloroplastic (LOC103854405), transcript variant X1, mRNA ; PREDICTED: Brassica rapa peroxiredoxin Q, chloroplastic (LOC103854405), transcript variant X2, mRNA ; Similar to PRXQ Peroxiredoxin Q, chloroplastic (Arabidopsis thaliana)::Bra022015:0-216 | Bra022015 | 24 kDa | 1 | 6 | 6 | 0 |
| 230 | Similar to SSL9 Protein STRICTOSIDINE SYNTHASE-LIKE 9 (Arabidopsis thaliana)::Bra026064:0-1565 | Bra026064 | 175 kDa | 11 | 10 | 0 | 4 |
| 231 | PREDICTED: Brassica napus aspartic proteinase A3 (LOC106366922), mRNA ; PREDICTED: Brassica napus aspartic proteinase A3 (LOC106376996), transcript variant X1, mRNA ; PREDICTED: Brassica napus aspartic proteinase A3 (LOC106376996), transcript variant X2, mRNA ; PREDICTED: Brassica napus aspartic proteinase A3 (LOC106376996), transcript variant X3, mRNA ; PREDICTED: Brassica oleracea var. oleracea aspartic proteinase A3 (LOC106312989), mRNA ; PREDICTED: Brassica rapa aspartic proteinase A3 (LOC103839838), transcript variant X1, mRNA ; PREDICTED: Brassica rapa aspartic proteinase A3 (LOC103839838), transcript variant X2, mRNA ; Similar to APA3 Aspartic proteinase A3 (Arabidopsis thaliana)::Bra002262:0-532 | Bra002262 | 58 kDa | 9 | 4 | 0 | 0 |
| 232 | PREDICTED: Brassica napus protein usf (LOC106447086), mRNA ; Protein of unknown function::Bra040434:0-239 | Bra040434 | 26 kDa | 0 | 8 | 0 | 3 |
| 233 | Similar to NUCL1 Nucleolin 1 (Arabidopsis thaliana)::Bra020321:0-419 | Bra020321 (+1) | 45 kDa | 1 | 7 | 3 | 1 |
| 234 | Similar to RPS15A 40S ribosomal protein S15a (Brassica napus)::Bra007545:0-292 | Bra007545 (+3) | 33 kDa | 0 | 8 | 0 | 3 |
| 235 | PREDICTED: Brassica napus probable desiccation-related protein LEA14 (LOC106407032), mRNA ; PREDICTED: Brassica oleracea var. oleracea probable desiccation-related protein LEA14 (LOC106329308), mRNA ; PREDICTED: Brassica rapa probable desiccation-related protein LEA14 (LOC103836663), mRNA ; Similar to LEA14 Probable desiccation-related protein LEA14 (Arabidopsis thaliana)::Bra034987:0-151 | Bra034987 | 16 kDa | 10 | 0 | 0 | 2 |
| 236 | PREDICTED: Brassica napus 40S ribosomal protein S8-1-like (LOC106371762), mRNA ; PREDICTED: Brassica napus 40S ribosomal protein S8-1-like (LOC106439493), mRNA ; PREDICTED: Brassica oleracea var. oleracea 40S ribosomal protein S8-1 (LOC106313191), mRNA ; PREDICTED: Brassica rapa 40S ribosomal protein S8-1 (LOC103845690), mRNA ; Similar to RPS8A 40S ribosomal protein S8-1 (Arabidopsis thaliana)::Bra036995:0-228 | Bra036995 | 26 kDa | 2 | 4 | 1 | 4 |
| 237 | PREDICTED: Brassica napus uncharacterized protein At2g27730, mitochondrial-like (LOC106352562), transcript variant X1, mRNA ; PREDICTED: Brassica rapa uncharacterized protein At2g27730, mitochondrial-like (LOC103829682), transcript variant X1, mRNA ; Similar to At2g27730 Uncharacterized protein At2g27730, mitochondrial (Arabidopsis thaliana)::Bra025687:0-114 | Bra025687 | 12 kDa | 2 | 2 | 5 | 1 |
| 238 | Similar to CFIS2 Pre-mRNA cleavage factor Im 25 kDa subunit 2 (Arabidopsis thaliana)::Bra033759:0-453 | Bra033759 | 51 kDa | 3 | 2 | 6 | 0 |
| 239 | Brassica oleracea HDEM genome, scaffold: C1 ; Brassica rapa genome, scaffold: A01 ; PREDICTED: Brassica napus peptidyl-prolyl cis-trans isomerase (LOC106375290), mRNA ; PREDICTED: Brassica napus peptidyl-prolyl cis-trans isomerase (LOC106434732), transcript variant X2, mRNA ; PREDICTED: Brassica oleracea var. oleracea peptidyl-prolyl cis-trans isomerase CYP18-4 (LOC106341430), mRNA ; PREDICTED: Brassica rapa peptidyl-prolyl cis-trans isomerase (LOC103833770), mRNA ; Similar to PCKR1 Peptidyl-prolyl cis-trans isomerase (Catharanthus roseus) ; Zea mays clone 13538 mRNA sequence ; Zea mays clone 93630 mRNA sequence::Bra011689:0-171 | Bra011689 | 18 kDa | 0 | 6 | 2 | 3 |
| 240 | PREDICTED: Brassica napus 60S ribosomal protein L13a-2-like (LOC106434833), mRNA ; PREDICTED: Brassica napus 60S ribosomal protein L13a-2-like (LOC106435375), mRNA ; PREDICTED: Brassica oleracea var. oleracea 60S ribosomal protein L13a-2-like (LOC106301537), mRNA ; PREDICTED: Brassica rapa 60S ribosomal protein L13a-2-like (LOC103828595), mRNA ; PREDICTED: Raphanus sativus 60S ribosomal protein L13a-2-like (LOC108856366), mRNA ; Similar to RPL13AB 60S ribosomal protein L13a-2 (Arabidopsis thaliana) ; Zea mays clone 12538 mRNA sequence::Bra024793:0-206 | Bra024793 | 24 kDa | 4 | 5 | 0 | 2 |
| 241 | PREDICTED: Brassica napus glutaredoxin-C2 (LOC106446144), mRNA ; PREDICTED: Brassica rapa glutaredoxin-C2 (LOC103863982), mRNA ; Similar to GRXC2 Glutaredoxin-C2 (Arabidopsis thaliana) ; Zea mays clone 11723 mRNA sequence::Bra039532:0-111 | Bra039532 | 12 kDa | 0 | 6 | 2 | 2 |
| 242 | PREDICTED: Brassica napus fasciclin-like arabinogalactan protein 1 (LOC106371391), mRNA ; PREDICTED: Brassica napus fasciclin-like arabinogalactan protein 1 (LOC106390893), mRNA ; PREDICTED: Brassica oleracea var. oleracea fasciclin-like arabinogalactan protein 1 (LOC106319394), mRNA ; PREDICTED: Brassica rapa fasciclin-like arabinogalactan protein 1 (LOC103845019), mRNA ; Similar to FLA1 Fasciclin-like arabinogalactan protein 1 (Arabidopsis thaliana)::Bra036516:0-426 | Bra036516 | 45 kDa | 7 | 0 | 1 | 3 |
| 243 | PREDICTED: Brassica napus 60S ribosomal protein L34-2-like (LOC106394022), mRNA ; PREDICTED: Brassica napus 60S ribosomal protein L34-2 (LOC106372741), mRNA ; PREDICTED: Brassica oleracea var. oleracea 60S ribosomal protein L34-2 (LOC106324938), mRNA ; PREDICTED: Brassica rapa 60S ribosomal protein L34-2-like (LOC103852598), mRNA ; Similar to RPL34B 60S ribosomal protein L34-2 (Arabidopsis thaliana)::Bra017546:0-120 | Bra017546 (+3) | 14 kDa | 0 | 6 | 2 | 3 |
| 244 | Similar to AGAL1 Alpha-galactosidase 1 (Arabidopsis thaliana)::Bra006906:0-741 | Bra006906 (+1) | 82 kDa | 4 | 4 | 0 | 3 |
| 245 | Brassica oleracea HDEM genome, scaffold: C7 ; Brassica rapa genome, scaffold: A03 ; PREDICTED: Brassica napus 60S ribosomal protein L26-1-like (LOC106402150), mRNA ; PREDICTED: Brassica napus 60S ribosomal protein L26-1-like (LOC106439994), mRNA ; PREDICTED: Brassica napus 60S ribosomal protein L26-1-like (LOC106451943), mRNA ; PREDICTED: Brassica oleracea var. oleracea 60S ribosomal protein L26-1-like (LOC106301991), mRNA ; PREDICTED: Brassica rapa 60S ribosomal protein L26-1-like (LOC103860635), mRNA ; PREDICTED: Raphanus sativus 60S ribosomal protein L26-1 (LOC108852068), mRNA ; PREDICTED: Raphanus sativus 60S ribosomal protein L26-1 (LOC108852079), mRNA ; Similar to RPL26A 60S ribosomal protein L26-1 (Arabidopsis thaliana)::Bra010383:0-146 | Bra010383 (+1) | 17 kDa | 0 | 7 | 0 | 4 |
| 246 | PREDICTED: Brassica napus ATP-citrate synthase beta chain protein 2 (LOC106365855), transcript variant X1, mRNA ; PREDICTED: Brassica napus ATP-citrate synthase beta chain protein 2 (LOC106365855), transcript variant X2, mRNA ; PREDICTED: Brassica napus ATP-citrate synthase beta chain protein 2 (LOC111197906), transcript variant X1, mRNA ; PREDICTED: Brassica napus ATP-citrate synthase beta chain protein 2 (LOC111197906), transcript variant X2, mRNA ; PREDICTED: Brassica oleracea var. oleracea ATP-citrate synthase beta chain protein 2 (LOC106318884), transcript variant X1, mRNA ; PREDICTED: Brassica oleracea var. oleracea ATP-citrate synthase beta chain protein 2 (LOC106318884), transcript variant X2, mRNA ; PREDICTED: Brassica rapa ATP-citrate synthase beta chain protein 2 (LOC103837286), transcript variant X1, mRNA ; PREDICTED: Brassica rapa ATP-citrate synthase beta chain protein 2 (LOC103837286), transcript variant X2, mRNA ; Similar::Bra000310:0-738 | Bra000310 (+1) | 81 kDa | 11 | 0 | 0 | 0 |
| 247 | PREDICTED: Brassica napus hsp70 nucleotide exchange factor fes1-like (LOC111215787), mRNA ; PREDICTED: Brassica rapa uncharacterized LOC103870549 (LOC103870549), mRNA ; Protein of unknown function::Bra031278:0-372 | Bra031278 | 41 kDa | 3 | 3 | 1 | 2 |
| 248 | PREDICTED: Brassica napus 2-hydroxyacyl-CoA lyase-like (LOC106445967), mRNA ; PREDICTED: Brassica rapa 2-hydroxyacyl-CoA lyase (LOC103833165), mRNA ; Similar to HACL 2-hydroxyacyl-CoA lyase (Arabidopsis thaliana)::Bra037154:0-574 | Bra037154 | 61 kDa | 1 | 5 | 1 | 3 |
| 249 | Brassica oleracea HDEM genome, scaffold: C9 ; Brassica rapa genome, scaffold: A09 ; PREDICTED: Brassica napus 40S ribosomal protein S28-2 (LOC106357658), mRNA ; PREDICTED: Brassica napus 40S ribosomal protein S28-2 (LOC106364133), mRNA ; PREDICTED: Brassica napus 40S ribosomal protein S28-2 (LOC106364145), transcript variant X1, mRNA ; PREDICTED: Brassica napus 40S ribosomal protein S28-2 (LOC106364145), transcript variant X2, mRNA ; PREDICTED: Brassica oleracea var. oleracea 40S ribosomal protein S28-2 (LOC106317812), mRNA ; PREDICTED: Brassica rapa 40S ribosomal protein S28-2 (LOC103837515), transcript variant X1, mRNA ; PREDICTED: Brassica rapa 40S ribosomal protein S28-2 (LOC103837515), transcript variant X2, mRNA ; PREDICTED: Raphanus sativus 40S ribosomal protein S28-2 (LOC108827482), mRNA ; Similar to RPS28C 40S ribosomal protein S28-2 (Arabidopsis thaliana)::Bra000692:0-64 | Bra000692 (+4) | 7 kDa | 3 | 3 | 1 | 3 |
| 250 | Brassica rapa copper/zinc superoxide dismutase (CSD) mRNA, partial cds ; PREDICTED: Brassica napus superoxide dismutase [Cu-Zn] 2, chloroplastic (LOC106446718), mRNA ; PREDICTED: Brassica oleracea var. oleracea superoxide dismutase [Cu-Zn] 2, chloroplastic (LOC106336916), mRNA ; PREDICTED: Brassica rapa superoxide dismutase [Cu-Zn] 2, chloroplastic (LOC103864828), mRNA ; Similar to CSD2 Superoxide dismutase [Cu-Zn] 2, chloroplastic (Arabidopsis thaliana) ; Zea mays clone 13950 mRNA sequence::Bra040196:0-207 | Bra040196 | 21 kDa | 5 | 2 | 1 | 2 |
| 251 | PREDICTED: Brassica napus aspartyl protease AED3-like (LOC106415755), mRNA ; PREDICTED: Brassica napus aspartyl protease AED3 (LOC106415612), mRNA ; PREDICTED: Brassica oleracea var. oleracea protein ASPARTIC PROTEASE IN GUARD CELL 2-like (LOC106312619), mRNA ; PREDICTED: Brassica rapa aspartyl protease AED3-like (LOC103841353), mRNA ; PREDICTED: Raphanus sativus aspartyl protease AED3-like (LOC108862032), transcript variant X1, mRNA ; PREDICTED: Raphanus sativus aspartyl protease AED3-like (LOC108862032), transcript variant X2, mRNA ; Protein of unknown function::Bra004945:0-430 | Bra004945 | 46 kDa | 2 | 2 | 0 | 2 |
| 252 | Brassica oleracea HDEM genome, scaffold: C8 ; Brassica rapa genome, scaffold: A08 ; PREDICTED: Brassica napus major oleosin NAP-II-like (LOC106419670), mRNA ; PREDICTED: Brassica napus major oleosin NAP-II-like (LOC111208674), mRNA ; PREDICTED: Brassica oleracea var. oleracea major oleosin NAP-II-like (LOC106319905), mRNA ; PREDICTED: Brassica rapa oleosin 16 kDa-like (LOC103832967), mRNA ; Protein of unknown function::Bra032113:0-166 | Bra032113 | 18 kDa | 4 | 0 | 3 | 2 |
| 253 | PREDICTED: Brassica napus uncharacterized protein ECU03_1610 (BNAA02G22630D), mRNA ; PREDICTED: Brassica rapa uncharacterized protein ECU03_1610-like (LOC103848870), mRNA ; Similar to ECU03_1610 Uncharacterized protein ECU03_1610 (Encephalitozoon cuniculi (strain GB-M1))::Bra018080:0-253 | Bra018080 | 28 kDa | 4 | 2 | 2 | 0 |
| 254 | PREDICTED: Brassica napus tubulin alpha-3 chain-like (LOC106425816), transcript variant X1, mRNA ; PREDICTED: Brassica napus tubulin alpha-3 chain-like (LOC106425817), transcript variant X1, mRNA ; PREDICTED: Brassica rapa tubulin alpha-3 chain-like (LOC103851297), mRNA ; Similar to TUBA5 Tubulin alpha-5 chain (Arabidopsis thaliana)::Bra016485:0-450 | Bra016485 (+5) | 50 kDa | 0 | 5 | 1 | 4 |
| 255 | Similar to RPP0B 60S acidic ribosomal protein P0-2 (Arabidopsis thaliana)::Bra009661:0-690 | Bra009661 (+1) | 77 kDa | 3 | 0 | 1 | 6 |
| 256 | PREDICTED: Brassica napus 60S ribosomal protein L3-1-like (LOC106371594), mRNA ; PREDICTED: Brassica napus 60S ribosomal protein L3-1-like (LOC106418390), transcript variant X1, mRNA ; PREDICTED: Brassica napus 60S ribosomal protein L3-1-like (LOC106418390), transcript variant X2, mRNA ; PREDICTED: Brassica oleracea var. oleracea 60S ribosomal protein L3-1-like (LOC106327742), transcript variant X1, mRNA ; PREDICTED: Brassica rapa 60S ribosomal protein L3-1 (LOC103848547), mRNA ; Similar to ARP1 60S ribosomal protein L3-1 (Arabidopsis thaliana)::Bra018503:0-280 | Bra018503 | 32 kDa | 5 | 5 | 0 | 0 |
| 257 | PREDICTED: Brassica napus basic transcription factor 3 (LOC106369038), mRNA ; PREDICTED: Brassica oleracea var. oleracea transcription factor BTF3 homolog 4-like (LOC106309603), mRNA ; PREDICTED: Brassica rapa basic transcription factor 3-like (LOC103842730), mRNA ; Similar to BTF3L4 Transcription factor BTF3 homolog 4 (Bos taurus)::Bra005936:0-169 | Bra005936 (+1) | 18 kDa | 4 | 4 | 1 | 0 |
| 258 | PREDICTED: Brassica napus 60S ribosomal protein L9-2-like (LOC106381958), mRNA ; PREDICTED: Brassica napus 60S ribosomal protein L9-2-like (LOC106391789), transcript variant X1, mRNA ; PREDICTED: Brassica napus 60S ribosomal protein L9-2-like (LOC106391789), transcript variant X2, mRNA ; PREDICTED: Brassica oleracea var. oleracea 60S ribosomal protein L9-2 (LOC106313639), mRNA ; PREDICTED: Brassica rapa 60S ribosomal protein L9-2-like (LOC103838914), mRNA ; Similar to RPL9D 60S ribosomal protein L9-2 (Arabidopsis thaliana)::Bra002476:0-194 | Bra002476 (+1) | 22 kDa | 6 | 2 | 1 | 0 |
| 259 | PREDICTED: Brassica napus glycerophosphodiester phosphodiesterase GDPDL4-like (LOC106371412), mRNA ; PREDICTED: Brassica napus glycerophosphodiester phosphodiesterase GDPDL4-like (LOC106418457), mRNA ; PREDICTED: Brassica oleracea var. oleracea glycerophosphodiester phosphodiesterase GDPDL4 (LOC106318551), mRNA ; PREDICTED: Brassica rapa glycerophosphodiester phosphodiesterase GDPDL4 (LOC103844984), mRNA ; Similar to GDPDL4 Glycerophosphodiester phosphodiesterase GDPDL4 (Arabidopsis thaliana)::Bra036498:0-768 | Bra036498 | 84 kDa | 5 | 3 | 0 | 1 |
| 260 | PREDICTED: Brassica napus V-type proton ATPase catalytic subunit A-like (LOC106381450), mRNA ; PREDICTED: Brassica napus V-type proton ATPase catalytic subunit A-like (LOC106381541), mRNA ; PREDICTED: Brassica napus V-type proton ATPase catalytic subunit A (LOC106353430), mRNA ; PREDICTED: Brassica napus V-type proton ATPase catalytic subunit A (LOC106405672), transcript variant X1, mRNA ; PREDICTED: Brassica napus V-type proton ATPase catalytic subunit A (LOC106405672), transcript variant X2, mRNA ; PREDICTED: Brassica oleracea var. oleracea V-type proton ATPase catalytic subunit A-like (LOC106327423), mRNA ; PREDICTED: Brassica oleracea var. oleracea V-type proton ATPase catalytic subunit A (LOC106300466), transcript variant X1, mRNA ; PREDICTED: Brassica oleracea var. oleracea V-type proton ATPase catalytic subunit A (LOC106300466), transcript variant X2, mRNA ; PREDICTED: Brassica rapa V-type proton ATPase catalytic subunit A-like (LO::Bra017919:0-697 | Bra017919 | 77 kDa | 4 | 4 | 0 | 1 |
| 261 | PREDICTED: Brassica napus AIG2-like protein A (LOC106432325), mRNA ; PREDICTED: Brassica napus AIG2-like protein A (LOC106446035), mRNA ; PREDICTED: Brassica oleracea var. oleracea AIG2-like protein (LOC106342351), mRNA ; PREDICTED: Brassica rapa AIG2-like protein (LOC103863913), mRNA ; Similar to At5g39720 AIG2-like protein (Arabidopsis thaliana)::Bra039463:0-166 | Bra039463 | 19 kDa | 3 | 3 | 0 | 3 |
| 262 | Brassica oleracea HDEM genome, scaffold: C9 ; Brassica rapa genome, scaffold: A09 ; Brassica rapa subsp. pekinensis sulfotransferase 5b (ST5b-7) gene, complete cds ; PREDICTED: Brassica napus cytosolic sulfotransferase 18-like (LOC106447906), mRNA ; PREDICTED: Brassica napus cytosolic sulfotransferase 18 (LOC106405028), mRNA ; PREDICTED: Brassica oleracea var. oleracea cytosolic sulfotransferase 18-like (LOC106314384), mRNA ; PREDICTED: Brassica rapa cytosolic sulfotransferase 18-like (LOC103838473), mRNA ; Similar to SOT18 Cytosolic sulfotransferase 18 (Arabidopsis thaliana)::Bra001541:0-368 | Bra001541 | 42 kDa | 3 | 4 | 0 | 2 |
| 263 | PREDICTED: Brassica napus probable pectinesterase/pectinesterase inhibitor 16 (LOC106351635), mRNA ; PREDICTED: Brassica napus probable pectinesterase/pectinesterase inhibitor 16 (LOC106391703), mRNA ; PREDICTED: Brassica oleracea var. oleracea probable pectinesterase/pectinesterase inhibitor 16 (LOC106339744), mRNA ; PREDICTED: Brassica rapa probable pectinesterase/pectinesterase inhibitor 16 (LOC103866037), mRNA ; Similar to PME16 Probable pectinesterase/pectinesterase inhibitor 16 (Arabidopsis thaliana)::Bra000006:0-516 | Bra000006 | 56 kDa | 4 | 4 | 0 | 0 |
| 264 | Similar to SULTR3;1 Sulfate transporter 3.1 (Arabidopsis thaliana)::Bra004751:0-794 | Bra004751 | 88 kDa | 8 | 1 | 0 | 0 |
| 265 | Similar to NAP1;2 Nucleosome assembly protein 1;2 (Arabidopsis thaliana)::Bra024503:0-308 | Bra024503 (+1) | 35 kDa | 6 | 3 | 0 | 0 |
| 266 | Similar to RAD23C Ubiquitin receptor RAD23c (Arabidopsis thaliana)::Bra009412:0-359 | Bra009412 | 38 kDa | 1 | 2 | 2 | 0 |
| 267 | PREDICTED: Brassica napus alpha-L-fucosidase 2-like (LOC106396165), mRNA ; PREDICTED: Brassica napus alpha-L-fucosidase 2 (LOC106354862), mRNA ; PREDICTED: Brassica oleracea var. oleracea alpha-L-fucosidase 2 (LOC106308629), mRNA ; PREDICTED: Brassica rapa alpha-L-fucosidase 2 (LOC103839946), mRNA ; Similar to FUC95A Alpha-L-fucosidase 2 (Arabidopsis thaliana)::Bra011731:0-836 | Bra011731 | 93 kDa | 2 | 3 | 0 | 1 |
| 268 | PREDICTED: Brassica napus 5-methyltetrahydropteroyltriglutamate--homocysteine methyltransferase 1 (LOC106386536), transcript variant X1, mRNA ; PREDICTED: Brassica napus 5-methyltetrahydropteroyltriglutamate--homocysteine methyltransferase 1 (LOC106386536), transcript variant X2, mRNA ; PREDICTED: Brassica napus 5-methyltetrahydropteroyltriglutamate--homocysteine methyltransferase 1 (LOC106386536), transcript variant X3, mRNA ; PREDICTED: Brassica napus 5-methyltetrahydropteroyltriglutamate--homocysteine methyltransferase 1 (LOC106433609), transcript variant X1, mRNA ; PREDICTED: Brassica napus 5-methyltetrahydropteroyltriglutamate--homocysteine methyltransferase 1 (LOC106433609), transcript variant X2, mRNA ; PREDICTED: Brassica oleracea var. oleracea 5-methyltetrahydropteroyltriglutamate--homocysteine methyltransferase 1 (LOC106332023), mRNA ; PREDICTED: Brassica rapa 5-methyltetrahydropteroyltriglutamate--homocysteine methyltransferase::Bra007311:0-765 | Bra007311 (+1) | 84 kDa | 2 | 4 | 2 | 0 |
| 269 | PREDICTED: Brassica napus phosphoglycerate kinase 1, chloroplastic-like (LOC106353104), mRNA ; PREDICTED: Brassica rapa phosphoglycerate kinase 1, chloroplastic (LOC103859466), mRNA ; Similar to PGK1 Phosphoglycerate kinase 1, chloroplastic (Arabidopsis thaliana)::Bra009772:0-484 | Bra009772 (+2) | 50 kDa | 3 | 4 | 0 | 5 |
| 270 | PREDICTED: Brassica napus 26S proteasome non-ATPase regulatory subunit 1 homolog A-like (LOC106447099), mRNA ; PREDICTED: Brassica napus 26S proteasome non-ATPase regulatory subunit 1 homolog A (LOC106427681), mRNA ; PREDICTED: Brassica oleracea var. oleracea 26S proteasome non-ATPase regulatory subunit 1 homolog A-like (LOC106341990), transcript variant X1, mRNA ; PREDICTED: Brassica oleracea var. oleracea 26S proteasome non-ATPase regulatory subunit 1 homolog A-like (LOC106341990), transcript variant X2, mRNA ; PREDICTED: Brassica rapa 26S proteasome non-ATPase regulatory subunit 1 homolog A-like (LOC103865226), mRNA ; Similar to RPN2A 26S proteasome non-ATPase regulatory subunit 1 homolog A (Arabidopsis thaliana)::Bra040450:0-1004 | Bra040450 | 109 kDa | 1 | 0 | 4 | 2 |
| 271 | PREDICTED: Brassica napus glutathione S-transferase F8, chloroplastic-like (LOC106438339), transcript variant X1, mRNA ; PREDICTED: Brassica napus glutathione S-transferase F8, chloroplastic-like (LOC106438339), transcript variant X2, mRNA ; PREDICTED: Brassica oleracea var. oleracea glutathione S-transferase F8, chloroplastic-like (LOC106335128), mRNA ; PREDICTED: Brassica rapa glutathione S-transferase F8, chloroplastic-like (LOC103858256), mRNA ; Similar to GSTF8 Glutathione S-transferase F8, chloroplastic (Arabidopsis thaliana)::Bra008877:0-215 | Bra008877 | 24 kDa | 4 | 3 | 0 | 0 |
| 272 | Similar to GSTL1 Glutathione S-transferase L1 (Arabidopsis thaliana)::Bra015689:0-411 | Bra015689 | 47 kDa | 4 | 0 | 0 | 4 |
| 273 | PREDICTED: Brassica rapa beta-glucosidase 19-like (LOC103872284), mRNA ; Similar to BGLU19 Beta-glucosidase 19 (Arabidopsis thaliana)::Bra021101:0-574 | Bra021101 | 65 kDa | 0 | 124 | 0 | 0 |
| 274 | PREDICTED: Brassica napus ATP synthase subunit d, mitochondrial (LOC106440060), mRNA ; PREDICTED: Brassica rapa ATP synthase subunit d, mitochondrial (LOC103860757), mRNA ; Similar to At3g52300 ATP synthase subunit d, mitochondrial (Arabidopsis thaliana)::Bra010299:0-168 | Bra010299 (+1) | 20 kDa | 1 | 3 | 2 | 1 |
| 275 | Similar to CYS4 Cysteine proteinase inhibitor 4 (Arabidopsis thaliana)::Bra041705:0-281 | Bra041705 | 32 kDa | 4 | 1 | 1 | 0 |
| 276 | PREDICTED: Brassica napus pyruvate kinase 1, cytosolic (LOC106367797), mRNA ; PREDICTED: Brassica napus pyruvate kinase 1, cytosolic (LOC106367920), mRNA ; PREDICTED: Brassica oleracea var. oleracea pyruvate kinase, cytosolic isozyme-like (LOC106311653), mRNA ; PREDICTED: Brassica rapa pyruvate kinase 1, cytosolic-like (LOC103841229), transcript variant X1, mRNA ; PREDICTED: Brassica rapa pyruvate kinase 1, cytosolic-like (LOC103841229), transcript variant X2, mRNA ; Similar to OsI_35105 Pyruvate kinase 1, cytosolic (Oryza sativa subsp. indica)::Bra004850:0-527 | Bra004850 (+2) | 58 kDa | 2 | 3 | 0 | 2 |
| 277 | PREDICTED: Brassica napus 2-oxoglutarate dehydrogenase, mitochondrial-like (LOC106348097), transcript variant X1, mRNA ; PREDICTED: Brassica napus 2-oxoglutarate dehydrogenase, mitochondrial-like (LOC106348097), transcript variant X2, mRNA ; PREDICTED: Brassica napus 2-oxoglutarate dehydrogenase, mitochondrial-like (LOC106348184), mRNA ; PREDICTED: Brassica oleracea var. oleracea 2-oxoglutarate dehydrogenase, mitochondrial (LOC106334854), mRNA ; PREDICTED: Brassica rapa 2-oxoglutarate dehydrogenase, mitochondrial (LOC103874014), mRNA ; Similar to sucA 2-oxoglutarate dehydrogenase E1 component (Leptospira interrogans serogroup Icterohaemorrhagiae serovar copenhageni (strain Fiocruz L1-130))::Bra022629:0-1014 | Bra022629 | 115 kDa | 4 | 1 | 0 | 1 |
| 278 | Brassica oleracea HDEM genome, scaffold: C5 ; Brassica rapa genome, scaffold: A06 ; PREDICTED: Brassica napus germin-like protein subfamily T member 2 (LOC106400858), mRNA ; PREDICTED: Brassica napus germin-like protein subfamily T member 2 (LOC111199002), mRNA ; PREDICTED: Brassica oleracea var. oleracea germin-like protein subfamily T member 2 (LOC106344049), mRNA ; PREDICTED: Brassica rapa germin-like protein subfamily T member 2 (LOC103872702), mRNA ; PREDICTED: Raphanus sativus germin-like protein subfamily T member 2 (LOC108854527), mRNA ; Similar to At1g18980 Germin-like protein subfamily T member 2 (Arabidopsis thaliana)::Bra021427:0-214 | Bra021427 | 23 kDa | 0 | 1 | 3 | 1 |
| 279 | Similar to INO80 Putative DNA helicase INO80 (Ashbya gossypii (strain ATCC 10895 / CBS 109.51 / FGSC 9923 / NRRL Y-1056))::Bra014441:0-691 | Bra014441 | 79 kDa | 3 | 1 | 0 | 1 |
| 280 | PREDICTED: Brassica napus proteasome subunit alpha type-5-A-like (LOC106360399), transcript variant X5, mRNA ; PREDICTED: Brassica rapa proteasome subunit alpha type-5-A-like (LOC103832649), transcript variant X2, mRNA ; Similar to PAE1 Proteasome subunit alpha type-5-A (Arabidopsis thaliana)::Bra031823:0-237 | Bra031823 | 26 kDa | 4 | 3 | 0 | 0 |
| 281 | PREDICTED: Brassica napus kynurenine formamidase-like (LOC106393997), mRNA ; PREDICTED: Brassica napus kynurenine formamidase-like (LOC106441136), mRNA ; PREDICTED: Brassica napus kynurenine formamidase-like (LOC111207619), mRNA ; PREDICTED: Brassica oleracea var. oleracea kynurenine formamidase (LOC106306226), mRNA ; PREDICTED: Brassica rapa kynurenine formamidase (LOC103862325), mRNA ; Protein of unknown function::Bra002912:0-259 | Bra002912 | 29 kDa | 6 | 1 | 0 | 0 |
| 282 | PREDICTED: Brassica napus 10 kDa chaperonin-like (LOC106364276), mRNA ; PREDICTED: Brassica napus 10 kDa chaperonin-like (LOC106382313), mRNA ; PREDICTED: Brassica rapa 10 kDa chaperonin (LOC103836048), mRNA ; Similar to 10 kDa chaperonin (Brassica napus)::Bra034561:0-98 | Bra034561 | 11 kDa | 0 | 0 | 4 | 2 |
| 283 | PREDICTED: Brassica napus ras-related protein RABD2b (LOC106430002), mRNA ; PREDICTED: Brassica rapa ras-related protein RABD2b (LOC103839540), mRNA ; PREDICTED: Brassica rapa ras-related protein RABD2b (LOC103853792), mRNA ; PREDICTED: Raphanus sativus ras-related protein RABD2b-like (LOC108812580), transcript variant X2, mRNA ; PREDICTED: Raphanus sativus ras-related protein RABD2b-like (LOC108824031), mRNA ; Similar to RABD2B Ras-related protein RABD2b (Arabidopsis thaliana)::Bra003498:0-202 | Bra003498 (+2) | 22 kDa | 7 | 0 | 0 | 0 |
| 284 | PREDICTED: Brassica napus 60S ribosomal protein L28-1-like (LOC106447120), mRNA ; Similar to RPL28A 60S ribosomal protein L28-1 (Arabidopsis thaliana)::Bra001082:0-203 | Bra001082 | 23 kDa | 1 | 3 | 2 | 0 |
| 285 | PREDICTED: Brassica napus 40S ribosomal protein S3-2 (LOC106437728), mRNA ; PREDICTED: Brassica rapa 40S ribosomal protein S3-2-like (LOC103857385), transcript variant X1, mRNA ; PREDICTED: Brassica rapa 40S ribosomal protein S3-2-like (LOC103857385), transcript variant X2, mRNA ; Similar to RPS3B 40S ribosomal protein S3-2 (Arabidopsis thaliana)::Bra008066:0-249 | Bra008066 (+2) | 27 kDa | 1 | 4 | 0 | 1 |
| 286 | PREDICTED: Brassica napus 40S ribosomal protein S15-1 (LOC106369246), mRNA ; Similar to RPS15A 40S ribosomal protein S15-1 (Arabidopsis thaliana)::Bra006484:0-152 | Bra006484 (+2) | 17 kDa | 3 | 3 | 0 | 0 |
| 287 | PREDICTED: Brassica napus protein disulfide isomerase-like 1-4 (LOC106365754), mRNA ; PREDICTED: Brassica napus protein disulfide isomerase-like 1-4 (LOC106371613), mRNA ; PREDICTED: Brassica oleracea var. oleracea protein disulfide isomerase-like 1-4 (LOC106318458), mRNA ; PREDICTED: Brassica rapa protein disulfide isomerase-like 1-4 (LOC103845478), mRNA ; Similar to PDIL1-4 Protein disulfide isomerase-like 1-4 (Arabidopsis thaliana)::Bra036870:0-596 | Bra036870 | 66 kDa | 0 | 2 | 3 | 0 |
| 288 | PREDICTED: Brassica napus proteasome subunit alpha type-7-A-like (LOC111200303), mRNA ; PREDICTED: Brassica napus proteasome subunit alpha type-7-A (LOC106367724), mRNA ; PREDICTED: Brassica oleracea var. oleracea proteasome subunit alpha type-7-A (LOC106307482), mRNA ; PREDICTED: Brassica rapa proteasome subunit alpha type-7-A (LOC103841046), mRNA ; Similar to PAD1 Proteasome subunit alpha type-7-A (Arabidopsis thaliana)::Bra004701:0-250 | Bra004701 (+1) | 27 kDa | 3 | 3 | 0 | 0 |
| 289 | PREDICTED: Brassica napus 6-phosphogluconate dehydrogenase, decarboxylating 3-like (LOC106352176), transcript variant X1, mRNA ; PREDICTED: Brassica napus 6-phosphogluconate dehydrogenase, decarboxylating 3-like (LOC106352176), transcript variant X2, mRNA ; PREDICTED: Brassica napus 6-phosphogluconate dehydrogenase, decarboxylating 3-like (LOC106352176), transcript variant X3, mRNA ; PREDICTED: Brassica napus 6-phosphogluconate dehydrogenase, decarboxylating 3 (LOC106349212), transcript variant X1, mRNA ; PREDICTED: Brassica napus 6-phosphogluconate dehydrogenase, decarboxylating 3 (LOC106349212), transcript variant X2, mRNA ; PREDICTED: Brassica oleracea var. oleracea 6-phosphogluconate dehydrogenase, decarboxylating 3 (LOC106340355), transcript variant X2, mRNA ; PREDICTED: Brassica oleracea var. oleracea 6-phosphogluconate dehydrogenase, decarboxylating 3 (LOC106340355), transcript variant X5, mRNA ; PREDICTED: Brassica rapa 6-phosphog::Bra015223:0-486 | Bra015223 (+1) | 54 kDa | 4 | 2 | 0 | 0 |
| 290 | Similar to PGIC Glucose-6-phosphate isomerase, cytosolic (Arabidopsis thaliana)::Bra001613:0-542 | Bra001613 | 60 kDa | 4 | 0 | 0 | 2 |
| 291 | Similar to PAB8 Polyadenylate-binding protein 8 (Arabidopsis thaliana)::Bra020285:0-627 | Bra020285 | 68 kDa | 3 | 1 | 1 | 0 |
| 292 | PREDICTED: Brassica napus glycine--tRNA ligase, mitochondrial 1-like (LOC111206420), misc_RNA ; PREDICTED: Brassica napus glycine--tRNA ligase, mitochondrial 1 (LOC106367376), mRNA ; PREDICTED: Brassica oleracea var. oleracea glycine--tRNA ligase 1, mitochondrial (LOC106295514), mRNA ; PREDICTED: Brassica rapa glycine--tRNA ligase, mitochondrial 1 (LOC103840454), mRNA ; Similar to At1g29880 Glycine--tRNA ligase, mitochondrial 1 (Arabidopsis thaliana)::Bra003997:0-690 | Bra003997 (+1) | 77 kDa | 2 | 2 | 1 | 0 |
| 293 | PREDICTED: Brassica napus alpha-xylosidase 1-like (LOC106354019), misc_RNA ; PREDICTED: Brassica napus alpha-xylosidase 1-like (LOC106405609), mRNA ; PREDICTED: Brassica oleracea var. oleracea alpha-xylosidase 1 (LOC106300147), mRNA ; PREDICTED: Brassica rapa alpha-xylosidase 1 (LOC103831266), mRNA ; Similar to XYL1 Alpha-xylosidase 1 (Arabidopsis thaliana)::Bra026970:0-926 | Bra026970 | 103 kDa | 2 | 3 | 0 | 0 |
| 294 | PREDICTED: Brassica napus dihydropyrimidine dehydrogenase (NADP(+)), chloroplastic-like (LOC106452022), mRNA ; PREDICTED: Brassica napus dihydropyrimidine dehydrogenase (NADP(+)), chloroplastic (LOC106427199), mRNA ; PREDICTED: Brassica rapa dihydropyrimidine dehydrogenase (NADP(+)), chloroplastic (LOC103869649), mRNA ; Similar to PYD1 Dihydropyrimidine dehydrogenase (NADP(+)), chloroplastic (Arabidopsis thaliana)::Bra030728:0-431 | Bra030728 | 47 kDa | 3 | 1 | 0 | 0 |
| 295 | PREDICTED: Brassica napus FAM10 family protein At4g22670-like (LOC106376536), transcript variant X1, mRNA ; PREDICTED: Brassica napus FAM10 family protein At4g22670-like (LOC106376536), transcript variant X2, mRNA ; PREDICTED: Brassica rapa FAM10 family protein At4g22670 (LOC103860206), transcript variant X1, mRNA ; PREDICTED: Brassica rapa FAM10 family protein At4g22670 (LOC103860206), transcript variant X2, mRNA ; Similar to At4g22670 FAM10 family protein At4g22670 (Arabidopsis thaliana)::Bra012611:0-416 | Bra012611 | 45 kDa | 3 | 2 | 0 | 0 |
| 296 | PREDICTED: Brassica napus ubiquitin carboxyl-terminal hydrolase 3-like (LOC111214250), mRNA ; PREDICTED: Brassica rapa ubiquitin carboxyl-terminal hydrolase 3-like (LOC103860918), mRNA ; Similar to UCH3 Ubiquitin carboxyl-terminal hydrolase 3 (Arabidopsis thaliana)::Bra010752:0-234 | Bra010752 | 26 kDa | 0 | 0 | 1 | 4 |
| 297 | Similar to VDAC1 Mitochondrial outer membrane protein porin 1 (Arabidopsis thaliana)::Bra031639:0-326 | Bra031639 | 35 kDa | 0 | 1 | 0 | 4 |
| 298 | Similar to LAZ1 Protein LAZ1 (Arabidopsis thaliana)::Bra011359:0-1735 | Bra011359 | 194 kDa | 3 | 2 | 0 | 0 |
| 299 | PREDICTED: Brassica napus uncharacterized BNAC03G41230D (BNAC03G41230D), mRNA ; PREDICTED: Brassica oleracea var. oleracea uncharacterized LOC106334556 (LOC106334556), mRNA ; PREDICTED: Brassica rapa uncharacterized LOC103859827 (LOC103859827), mRNA ; Protein of unknown function::Bra010054:0-416 | Bra010054 | 45 kDa | 0 | 3 | 0 | 2 |
| 300 | PREDICTED: Brassica napus adenylate kinase 4-like (LOC106347908), mRNA ; PREDICTED: Brassica napus adenylate kinase 4-like (LOC106431226), mRNA ; PREDICTED: Brassica napus adenylate kinase 4-like (LOC106431257), mRNA ; PREDICTED: Brassica napus adenylate kinase 4-like (LOC111208808), mRNA ; PREDICTED: Brassica oleracea var. oleracea adenylate kinase 4-like (LOC106328009), mRNA ; PREDICTED: Brassica rapa adenylate kinase 4-like (LOC103873752), mRNA ; Similar to ADK1 Adenylate kinase 4 (Arabidopsis thaliana)::Bra022431:0-240 | Bra022431 | 26 kDa | 8 | 0 | 6 | 0 |
| 301 | Similar to MNR1 (+)-neomenthol dehydrogenase (Capsicum annuum)::Bra035009:0-295 | Bra035009 | 32 kDa | 5 | 0 | 0 | 0 |
| 302 | PREDICTED: Brassica napus nucleoside diphosphate kinase 1 (LOC106366685), mRNA ; Similar to NDK1 Nucleoside diphosphate kinase 1 (Arabidopsis thaliana)::Bra001958:0-148 | Bra001958 | 16 kDa | 5 | 0 | 0 | 0 |
| 303 | PREDICTED: Brassica napus 60S ribosomal protein L35-1-like (LOC106388959), mRNA ; PREDICTED: Brassica napus 60S ribosomal protein L35-1 (LOC106439191), mRNA ; PREDICTED: Brassica oleracea var. oleracea 60S ribosomal protein L35-1-like (LOC106331644), mRNA ; PREDICTED: Brassica rapa 60S ribosomal protein L35-1 (LOC103859293), mRNA ; Similar to RPL35A 60S ribosomal protein L35-1 (Arabidopsis thaliana) ; Zea mays clone 10876 mRNA sequence::Bra009646:0-123 | Bra009646 (+3) | 14 kDa | 0 | 5 | 0 | 0 |
| 304 | Brassica rapa subsp. campestris polygalacturonase inhibitory protein (MF19) mRNA, complete cds ; PREDICTED: Brassica napus leucine-rich repeat protein FLOR 1 (LOC106351771), mRNA ; PREDICTED: Brassica napus leucine-rich repeat protein FLOR 1 (LOC106416600), mRNA ; PREDICTED: Brassica oleracea var. oleracea polygalacturonase inhibitor 1-like (LOC106323568), mRNA ; PREDICTED: Brassica rapa polygalacturonase inhibitor 1-like (LOC103845755), mRNA ; Similar to PGIP Polygalacturonase inhibitor (Pyrus communis)::Bra015163:0-325 | Bra015163 | 36 kDa | 0 | 5 | 0 | 0 |
| 305 | PREDICTED: Brassica napus heat shock 70 kDa protein 6, chloroplastic-like (LOC106348936), mRNA ; PREDICTED: Brassica napus heat shock 70 kDa protein 6, chloroplastic (LOC106440803), transcript variant X1, mRNA ; PREDICTED: Brassica napus heat shock 70 kDa protein 6, chloroplastic (LOC106440803), transcript variant X2, mRNA ; PREDICTED: Brassica oleracea var. oleracea heat shock 70 kDa protein 6, chloroplastic-like (LOC106306084), mRNA ; PREDICTED: Brassica rapa heat shock 70 kDa protein 6, chloroplastic-like (LOC103861480), mRNA ; Similar to HSP70-6 Heat shock 70 kDa protein 6, chloroplastic (Arabidopsis thaliana)::Bra011095:0-711 | Bra011095 (+1) | 76 kDa | 3 | 1 | 0 | 1 |
| 306 | PREDICTED: Brassica napus 26S proteasome regulatory subunit 6A homolog (LOC106388875), mRNA ; PREDICTED: Brassica napus 26S proteasome regulatory subunit 6A homolog (LOC111214163), mRNA ; PREDICTED: Brassica oleracea var. oleracea 26S protease regulatory subunit 6A homolog (LOC106328377), mRNA ; PREDICTED: Brassica rapa 26S protease regulatory subunit 6A homolog (LOC103859097), mRNA ; PREDICTED: Raphanus sativus 26S protease regulatory subunit 6A homolog (LOC108861240), mRNA ; Similar to TBP1 26S protease regulatory subunit 6A homolog (Brassica campestris)::Bra009512:0-424 | Bra009512 (+2) | 47 kDa | 2 | 0 | 1 | 1 |
| 307 | PREDICTED: Brassica napus peptidyl-prolyl cis-trans isomerase FKBP16-3, chloroplastic-like (LOC106391832), mRNA ; PREDICTED: Brassica napus peptidyl-prolyl cis-trans isomerase FKBP16-3, chloroplastic-like (LOC106424086), mRNA ; PREDICTED: Brassica napus peptidyl-prolyl cis-trans isomerase FKBP16-3, chloroplastic (LOC106391828), mRNA ; PREDICTED: Brassica oleracea var. oleracea peptidyl-prolyl cis-trans isomerase FKBP16-3, chloroplastic (LOC106337573), mRNA ; PREDICTED: Brassica rapa peptidyl-prolyl cis-trans isomerase FKBP16-3, chloroplastic-like (LOC103866085), mRNA ; Similar to FKBP16-3 Peptidyl-prolyl cis-trans isomerase FKBP16-3, chloroplastic (Arabidopsis thaliana)::Bra041096:0-216 | Bra041096 | 23 kDa | 2 | 2 | 0 | 0 |
| 308 | PREDICTED: Brassica napus late embryogenesis abundant protein 1-like (LOC106359738), mRNA ; PREDICTED: Brassica rapa late embryogenesis abundant protein 1-like (LOC103848743), mRNA ; Protein of unknown function::Bra033313:0-248 | Bra033313 | 27 kDa | 0 | 7 | 13 | 0 |
| 309 | PREDICTED: Brassica napus beta-D-xylosidase 4-like (LOC106371739), mRNA ; PREDICTED: Brassica napus beta-D-xylosidase 4-like (LOC106402417), mRNA ; PREDICTED: Brassica napus beta-D-xylosidase 4 (LOC106366244), mRNA ; PREDICTED: Brassica oleracea var. oleracea beta-D-xylosidase 4 (LOC106319519), mRNA ; PREDICTED: Brassica rapa beta-D-xylosidase 4-like (LOC103837535), mRNA ; Similar to BXL4 Beta-D-xylosidase 4 (Arabidopsis thaliana)::Bra000708:0-776 | Bra000708 (+1) | 84 kDa | 4 | 0 | 0 | 0 |
| 310 | PREDICTED: Brassica napus V-type proton ATPase subunit B3 (LOC106347084), transcript variant X1, mRNA ; PREDICTED: Brassica napus V-type proton ATPase subunit B3 (LOC106347084), transcript variant X2, mRNA ; PREDICTED: Brassica napus V-type proton ATPase subunit B3 (LOC106433719), transcript variant X1, mRNA ; PREDICTED: Brassica napus V-type proton ATPase subunit B3 (LOC106433719), transcript variant X2, mRNA ; PREDICTED: Brassica oleracea var. oleracea V-type proton ATPase subunit B3-like (LOC106295185), transcript variant X1, mRNA ; PREDICTED: Brassica oleracea var. oleracea V-type proton ATPase subunit B3-like (LOC106295185), transcript variant X2, mRNA ; PREDICTED: Brassica rapa V-type proton ATPase subunit B3 (LOC103872821), transcript variant X1, mRNA ; PREDICTED: Brassica rapa V-type proton ATPase subunit B3 (LOC103872821), transcript variant X2, mRNA ; Similar to VHA-B3 V-type proton ATPase subunit B3 (Arabidopsis thaliana)::Bra021536:0-486 | Bra021536 (+4) | 54 kDa | 4 | 0 | 0 | 0 |
| 311 | PREDICTED: Brassica napus tripeptidyl-peptidase 2-like (LOC106421156), mRNA ; PREDICTED: Brassica napus tripeptidyl-peptidase 2-like (LOC106440420), misc_RNA ; PREDICTED: Brassica oleracea var. oleracea tripeptidyl-peptidase 2 (LOC106303335), partial mRNA ; PREDICTED: Brassica rapa tripeptidyl-peptidase 2-like (LOC103861204), mRNA ; Similar to TPP2 Tripeptidyl-peptidase 2 (Arabidopsis thaliana)::Bra010903:0-1310 | Bra010903 | 144 kDa | 2 | 1 | 0 | 0 |
| 312 | Brassica oleracea HDEM genome, scaffold: C6 ; Brassica rapa genome, scaffold: A05 ; PREDICTED: Brassica napus phospholipase D alpha 2-like (LOC106449389), mRNA ; PREDICTED: Brassica napus phospholipase D alpha 2 (LOC106405207), mRNA ; PREDICTED: Brassica oleracea var. oleracea phospholipase D alpha 2 (LOC106299943), mRNA ; PREDICTED: Brassica rapa phospholipase D alpha 2 (LOC103868492), mRNA ; Similar to PLDALPHA1 Phospholipase D alpha 1 (Arabidopsis thaliana)::Bra029460:0-779 | Bra029460 (+1) | 88 kDa | 1 | 2 | 0 | 0 |
| 313 | Similar to HOP2 Hsp70-Hsp90 organizing protein 2 (Arabidopsis thaliana)::Bra001413:0-575 | Bra001413 | 65 kDa | 1 | 0 | 2 | 0 |
| 314 | PREDICTED: Brassica napus benzaldehyde dehydrogenase (NAD(+))-like (LOC106371001), mRNA ; PREDICTED: Brassica napus benzaldehyde dehydrogenase (NAD(+)) (LOC106402186), transcript variant X1, mRNA ; PREDICTED: Brassica napus benzaldehyde dehydrogenase (NAD(+)) (LOC106402186), transcript variant X2, mRNA ; PREDICTED: Brassica oleracea var. oleracea benzaldehyde dehydrogenase (NAD(+)) (LOC106295510), transcript variant X1, mRNA ; PREDICTED: Brassica oleracea var. oleracea benzaldehyde dehydrogenase (NAD(+)) (LOC106295510), transcript variant X2, mRNA ; PREDICTED: Brassica rapa benzaldehyde dehydrogenase (NAD(+)) (LOC103844240), transcript variant X1, mRNA ; PREDICTED: Brassica rapa benzaldehyde dehydrogenase (NAD(+)) (LOC103844240), transcript variant X2, mRNA ; Similar to AAO4 Benzaldehyde dehydrogenase (NAD(+)) (Arabidopsis thaliana)::Bra035276:0-1411 | Bra035276 | 155 kDa | 3 | 0 | 0 | 0 |
| 315 | PREDICTED: Brassica napus lysine--tRNA ligase, cytoplasmic-like (LOC106450425), mRNA ; PREDICTED: Brassica napus lysine--tRNA ligase, cytoplasmic (LOC106351199), mRNA ; PREDICTED: Brassica oleracea var. oleracea lysine--tRNA ligase (LOC106336618), mRNA ; PREDICTED: Brassica rapa lysine--tRNA ligase, cytoplasmic (LOC103846034), mRNA ; Similar to At3g11710 Lysine--tRNA ligase, cytoplasmic (Arabidopsis thaliana)::Bra015138:0-623 | Bra015138 | 71 kDa | 0 | 3 | 0 | 0 |
| 316 | Similar to HSP90-7 Endoplasmin homolog (Arabidopsis thaliana)::Bra012756:0-719 | Bra012756 | 82 kDa | 2 | 0 | 0 | 0 |
| 317 | PREDICTED: Brassica napus 26.5 kDa heat shock protein, mitochondrial (LOC106376590), mRNA ; PREDICTED: Brassica napus 26.5 kDa heat shock protein, mitochondrial (LOC106404842), transcript variant X1, mRNA ; PREDICTED: Brassica napus 26.5 kDa heat shock protein, mitochondrial (LOC106404842), transcript variant X2, mRNA ; PREDICTED: Brassica oleracea var. oleracea 26.5 kDa heat shock protein, mitochondrial (LOC106299182), mRNA ; PREDICTED: Brassica rapa 26.5 kDa heat shock protein, mitochondrial (LOC103871208), mRNA ; Similar to HSP26.5 26.5 kDa heat shock protein, mitochondrial (Arabidopsis thaliana)::Bra021021:0-232 | Bra021021 | 27 kDa | 0 | 2 | 0 | 0 |
| 318 | Similar to At3g01520 Universal stress protein A-like protein (Arabidopsis thaliana)::Bra026122:0-204 | Bra026122 | 23 kDa | 2 | 0 | 0 | 0 |
| 319 | Similar to RPT2A 26S proteasome regulatory subunit 4 homolog A (Arabidopsis thaliana)::Bra033654:0-901 | Bra033654 | 99 kDa | 2 | 0 | 0 | 0 |
| 320 | PREDICTED: Brassica napus 60S ribosomal protein L15-1 (LOC106440269), mRNA ; PREDICTED: Brassica rapa 60S ribosomal protein L15-1-like (LOC103860880), mRNA ; Similar to RPL15A 60S ribosomal protein L15-1 (Arabidopsis thaliana)::Bra010723:0-264 | Bra010723 (+3) | 30 kDa | 2 | 0 | 0 | 0 |
| 321 | PREDICTED: Brassica napus hsp70-Hsp90 organizing protein 3-like (LOC106362025), mRNA ; PREDICTED: Brassica napus hsp70-Hsp90 organizing protein 3 (LOC106402021), mRNA ; PREDICTED: Brassica rapa hsp70-Hsp90 organizing protein 3 (LOC103868140), mRNA ; Similar to HOP3 Hsp70-Hsp90 organizing protein 3 (Arabidopsis thaliana)::Bra018790:0-553 | Bra018790 | 63 kDa | 2 | 0 | 0 | 0 |
| 322 | PREDICTED: Brassica napus 14-3-3-like protein GF14 kappa (LOC106347979), transcript variant X1, mRNA ; PREDICTED: Brassica napus 14-3-3-like protein GF14 kappa (LOC106347979), transcript variant X2, mRNA ; Similar to GRF8 14-3-3-like protein GF14 kappa (Arabidopsis thaliana)::Bra022594:0-223 | Bra022594 | 25 kDa | 2 | 0 | 0 | 0 |
| 323 | PREDICTED: Brassica napus dihydrolipoyl dehydrogenase 1, chloroplastic (LOC106358154), mRNA ; PREDICTED: Brassica napus dihydrolipoyl dehydrogenase 1, chloroplastic (LOC106439448), transcript variant X1, mRNA ; PREDICTED: Brassica napus dihydrolipoyl dehydrogenase 1, chloroplastic (LOC106439448), transcript variant X2, mRNA ; PREDICTED: Brassica napus dihydrolipoyl dehydrogenase 1, chloroplastic (LOC106439448), transcript variant X3, mRNA ; PREDICTED: Brassica napus dihydrolipoyl dehydrogenase 1, chloroplastic (LOC106439448), transcript variant X4, mRNA ; PREDICTED: Brassica oleracea var. oleracea dihydrolipoyl dehydrogenase 1, chloroplastic (LOC106328211), mRNA ; PREDICTED: Brassica rapa dihydrolipoyl dehydrogenase 1, chloroplastic (LOC103859689), transcript variant X1, mRNA ; PREDICTED: Brassica rapa dihydrolipoyl dehydrogenase 1, chloroplastic (LOC103859689), transcript variant X2, mRNA ; PREDICTED: Brassica rapa dihydrolipoyl dehydrog::Bra009942:0-618 | Bra009942 (+1) | 66 kDa | 2 | 0 | 0 | 0 |

**S5 Table.** List of protein sequences of identified vicilin, oleosin and oil-body proteins *Brassica rapa* R-o-18 seed samples.

| Protein name (identified in *Brassica rapa* R-o-18) by LC-MS/MS | Amino acid length | Molecular weight (Da) | % identity | % coverage | Sequences (5'-3') in FASTA format |
| --- | --- | --- | --- | --- | --- |
| Identified vicilins | | | | | |
| Bra014536 | 486 | 54,657.4 Da | 100% with *B. napus* vicilin-like SSP At3g22640 | 40% | >Bra014536  MAINKLTITLFLLISLAVFHCLAFRVEVQEFEPPRQEGQEGPGGGSGEGWDEEATKNP  YHFGQWSFKNFFQSKDGFVKMLPKFTKRSSTLFRGIENYRFLFQEMQPNTFLVPHHLD  ADYVFLVVQGKGVIGFVTDTANESFQITKGDVVRVPSSVTHFFANTNGTVPLRLAKIA  VPANVPGHFQVFFPAHSGFHQSYFNGFSKDVLTASFNIPEELLGRLIRGPQQEVGQGI  IRRVSPEQIKELTEHEHATSPSNKHKDKKDKHKDKDRSTFGSPFNLLTQDAIYSNNFG  RYHEAHPKRFSQLQDLDIAVGWVNMTQGSLFLPQYNSETTFVTFVENGCARYEMASPY  TFQGEQQQPWFGPGQEEEVEEEMSGQVHKIVSRVCKGEVFILPAGHPFAILSQDENFV  AVGFGIHASNSTRTFLAGQDNMLSNINTVATRLSFGLGSKMAEKLFTSQNYSHFAPTT  PSHQFPEKPKPSFQSVFNLVGF |
| Bra025745 | 464 | 52,074.5 Da | 100% with *B. napus* vicilin-like SSP At2g28490 | 27% | >Bra025745  MEKNKRIFTFLLVIMFFHGVMMMRSIGYEGEEEQGGGGRERGGFMMKESRQVIKSEGG  EMRVVISPRGRIIEKPMHIGFLTMEPKTLFVPQYLDSNLLIFIREATLGVICKDEFGE  KRLKGGDIYWIPAGSAFYLLNTGRGQRLHVICSIDPSQSLGFETFQPFYIGGGPSSVL  AGFDPDTITSALNVSRPEVQQLMTSQVRGPIVHITEHAPTMWTDFLGLRGEEKHKHLK  KLLEMKQGTSQEQEFNPWWSWKNIVSSILDVTGEKNRGSGSSKCEDSYNIYDLKNDFE  NDYGWSKALDYDDYEPLRYSGVGVYLVNLTAGSMMAPHMNPTATEYGIVLSGSGEIQV  VLPNGTSAMNMRVSPGDVFWIPRYFAFCQIASRIAPFEFVGFTTSAYKNRPQFLVGSN  SLLRSLNLTSLAMAFGVDEGTMKRFVEAQREAVILPTASAAPPHEGEPERFGSDHIFT |
| Bra033857 | 576 | 65,332.1 Da | 100% with *B. napus* vicilin-like SSP At4g36700 | 18% | >Bra033857  MIRFTVLSFFVVFVLLFACNESSAKTAKYDKSDESVENDDLAAVPSCCGFSSPLLIKK  DQWKPIFANKFGQISTVQIGDGCGGMGPYKIHSITLEPNALMLPLLLHSDMVFFVDSG  SGILNWVEAQATSTEIRLGDVYRLRPGTVFYLQSKPVDIFLGTKLKIYAIFSNSQECL  HDPCFGAYSSVTDLLFGFDETILKSAFGVPEEIIGLMRNRTQPPLIVHDMLTTPSEAN  TDTDTNTWPLQTRLLKLFSGDASADSVENKKVKKKKEKKEKKKKPKKATTFNVFESEP  DFQSPNGQTITINRKDLKVLQGSMVGVSMVNLTQGSMMGPHWNPWACEISVVVRGSGM  VRVLRNSISRSSSECKNMRFKVEKGDIFAVPRLHPMAQMSFLNDSLVFVGFTTSAKNN  EPQFLAGKNSALWSLDREVLAASFNVSSFMIAGLLEAQKEAAVLGCPACAEGELEKLK  EDEEKKESPPQQPPQPFQPQPPEEKPQQPPQPFQPQPPQGEPQKPPQGEPQGPEKPFQ  PQPGQGEPQEPQASMETKMRDEERKREEEEAKKEEEERWKQEEKLWPTQPQWED |
| Bra003060 | 502 | 56,142.3 Da | 98% with *B. napus* vicilin-like SSP At4g36700 | 3% | >Bra003060  MTKFTVLPLFVLLFLVLLCTKSWAKSEEFDESSDEENDVAAVPSCCGFSSPLLIKKDQ  WKPIFGTQFGQISTVQIGEGCGGMGPYKIHSITLEPNALLLPLLLHSDMVFFVESGSG  ILNWVEAEPTSSEIRRGDVYRLRPGTVFYLQSKPIDIFLGTKLRVYAIFSNTEECLHD  PCFGAYSSITDLLFGFDEAILQSAFGVPEEIIGLMTNRTQPPLIVHDMLSTPGEANTY  TWQLQVQPRLLKLFAGYVSAAEKKKKEKKTKKAKTFNVFESEPDFQSPSGRTITINRK  DLEVLSGSMVGVSMVNLTQASMMGPHWNPWACEISIVLKGSGMVRVLRSSISSTSSSS  SSSECKNMRFKVEEGDIFAVPRLHPMAQMSFINESLVFIGFTTSARNNEPQFLAGQRS  ALRLLDQEVLAASLNVSSVMIEGLLGAQKDAVVLGCPYCAEGELEKLKVETEMKKRDD  ERKREEEEAKKEEEERRKREEEEEEEKQWPPLPQQPPE |
| Bra022682 | 657 | 83,300.9 Da | 100% with *B. napus* vicilin-like SSP At2g18540 | 3% | >Bra022682  MSKFTIIPLCLLTLFLCTNSFSDQNDGVPSSQSPLLVKRHQRTQLVATEFGEISAVHI  GEEYTIQFITLEPNALLLPLLLHSDMVFFVHTGSGVLNWVDEEKERTLELKRGDVFRL  RYGTVFYLHCNLERDEVPEKLRVYAIFDVGKCLSDQCLGAYSSIRDLLWGFDEKTLRS  AFAVPKDVFGRLRDAVKPPLITHAMPKNRTQGSEEETWGSRLAKLFVRVEDSIVVDEK  DMDALKGSSFGVYMVNLTKGSMMGPHWNPNACEISIVLQGEGMIRVVNHPSYQSKNES  ERFMVEDGDVFVVPQFYPMAQLSFVNSSFMFMGFSTSAKTNHPQFLVGQNSVLKIFNR  DVLATSFNMRYATVERLLGAQKDGLLLECVSCAEVELSRLMREIEERRRREEEEIERR  KREEEEAKRQEEERRRREEEEAERKKKAEEEARKREKEREREEEAAKRREEERRRREE  EEAERKRKEEEEARKREEERKREEEAAKKREEERRKREKEEEEARKREEAREREEEEA  KKREEERRKREEEEAERKRRAEEEAREREEEEAKKREEEKEAARRREEEREKEEEMAK  RREEERQRKEREDVERKKREEEEERKRREEEAMRREEERKREEEAAKRAEEERRKREE  EAEHKKRPPPQGPQPPIHH |
| Identified oleosins | | | | | |
| Bra019493 | 188 | 19,972.4 Da | 100% identity with Bra019493:0-188 | 44% | >Bra019493 PREDICTED: Brassica rapa oleosin S2-2 (LOC103854221), mRNA ; Similar to S2 Oleosin S2-2 (Brassica napus)::Bra019493:0-188  MANVDRRVNVDRTDKGLQLQPQYEDRVGYGYGYGGNTDYKSRGPSTNQIVALIAGVPIGGSLLALAGLTLAGSVIGFMLSIPLFLLFSPVIVPAALTIGLAVTGILASGLFGLTGLSSVSWVLNYIRGRSDTVPEQLDYAKRRMADAVGYAGQKGKEMGQYVQDKAHEAHDTSLTTETNGKARRAHIA |
| Bra000167 | 188 | 19,879.9 Da | 100% identity with  Bra000167 | 46% | >Bra000167 PREDICTED: Brassica oleracea var. oleracea oleosin S2-2 (LOC106316842), mRNA ; PREDICTED: Brassica rapa oleosin S2-2 (LOC103837001), mRNA ; Similar to S2 Oleosin S2-2 (Brassica napus)::Bra000167:0-188  MATVERRVQVDPTDKRIHLQPQYEGDVGYGYGYGGRADYKSSGPSSNQIVALIVGVPVGGSLLALAGLTLAGSVIGLMLSVPLFLLFSPVIVPAAITIGLAVTAILASGLFGLTGLSSVSWVLNYLRGTSDTVPEQLDYAKRRMADAVGYAGQKGKEMGQYVQDKAHEAHDTSLTTETTEPGKTRRHT |
| A0A078GHK4 | 180 | 19,515.6 Da | 100% identity with A0A078GHK4_BRANA | 42% | >tr\|A0A078GHK4\|A0A078GHK4_BRANA Oleosin OS=Brassica napus OX=3708 GN=BnaA08g14540D PE=3 SV=1  MADTARTHHDITSRDQYPILGRDRDQYPYGRSDYQTSGQDYSKTRQIAKAATAVTAGGSL  LVLSSLTLVGTVIALTVATPLLVIFSPILVPALITVALLITGFLSSGGFGIAAITVFSWI  YKYATGEHPQGSDKLDSARMKLGTKAQDIKDRAQYYGQQHTGGEHDRDRTRGTHHTTTTT |
| M4E9X1 | 220 | 23,024.7 Da | 100% identity with BRARP Oleosin | 50% | >tr\|M4E9X1\|M4E9X1_BRARP Oleosin OS=Brassica rapa subsp. pekinensis OX=51351 PE=3 SV=1  MADTHRVDRTDRHLQFQSPYEGGRVNIQFEGAGGGYGQSGYGGGGGYGQSGYGGGGYKSMMPESGPSSTQVISFLVGVPLVGSLLAIAGLLLAGSVIGLMISIPLFLLFSPVIVPAAITIGLATTGFLTSGMFGLTGLSSISWVMNYLRRTRGSVPDQLEYAKRRMADAVGYAGQKGKEVGQFVQDKAHDAKQYDISKPHDTTTTTTTTTKGLETRTAAA |
| A0A397KW15 | 183 | 20,0002.4 Da | 100% identity with A0A397KW15_BRACM Oleosin | 36% | >tr\|A0A397KW15\|A0A397KW15_BRACM Oleosin OS=Brassica campestris OX=3711 GN=BRAA03T14647Z PE=3 SV=1  MTDTARTHHDITTRDQYPMMGRDRDQYAIIGRDQYQGYGQDYSKSRQIAKAATAVTAGGS  LLVLSSLTLVGTVIALIVATPLLVIFSPILVPALITVALLITGFLSSGGFGIAAITVFSW  IYKYATGEHPKGSDKLDSARMKLGSKAQDMKDRAHYYGQQHTGGEHVNTDYRNTDRDRTR  GTT |
| A0A397ZZI8 | 193 | 20,790.6 Da | 100% identity with A0A397ZZI8_BRACM Oleosin | 24% | >tr\|A0A397ZZI8\|A0A397ZZI8_BRACM Oleosin OS=Brassica campestris OX=3711 GN=BRARA_C02941 PE=3 SV=1  MADVRTHAHQVQVHPLRQQEGGIKVVYPQSGPSSTQVLAVIAGVPVGGTLLTLAGLTLAG  SVIGLMLAFPLFLIFSPVIVPAAFVIGLAMTGFMASGAIGLTGLSSMSWVLNHIRRVRER  MPDELEEAKQRLADMAEYVGQRTKDAGQTIEEKAHDVRESKTYDVRDRDTKGHTATGGDR  DTKTTREVRVATT |
| M4DBK6 | 195 | 21,511.9 Da | 100% identity with M4DBK6_BRARP Oleosin | 44% | >tr\|M4DBK6\|M4DBK6_BRARP Oleosin OS=Brassica rapa subsp. pekinensis OX=51351 PE=3 SV=1  MTDTARTHHDITSRDQYPRDRDQYSMIGRDRDKYSMIGRDRDQYNMYGRDYSKSRQIAKA  VTAVTAGGSLLVLSSLTLVGTVIALTVATPLLVIFSPILVPALITVALLITGFLSSGGFG  IAAITVFSWIYKYATGEHPQGSDKLDSARMKLGGKVQDMKDRAQYYGQQHTGGYGQQQTG  GEHDRDRTRGTQHTT |
| M4EI43 | 211 | 22,095.6 Da | 100% identity with M4EI43_BRARP Oleosin | 35% | >tr\|M4EI43\|M4EI43_BRARP Oleosin OS=Brassica rapa subsp. pekinensis OX=51351 PE=3 SV=1  MADTHRVDRTDRHLQFQSPYEGGRVNIQYEGGGGAGGYGGGRGGGYGAGGYKSMMPERGP  SSTQVLSFLVGVPIVGSLLAIAGLLLAGSVIGLLISIPLFLLFSPVIVPAALTIGLAATG  FLASGMFGLTGLSSVSWVMNYLRGTRKSSVPEQLEYAKKRMADAVGYAGQKGKEMGQHVQ  NKAQEAKQYDISKTHDTTTKGHETTQRTAAA |
| Bra035756 | 205 | 21734.5 Da | 100% identity with Bra035756 | 24% | >Bra035756 PREDICTED: Brassica napus oleosin 14.9 kDa (LOC106375666), mRNA ; PREDICTED: Brassica napus oleosin 14.9 kDa (LOC106402530), mRNA ; PREDICTED: Brassica rapa oleosin 14.9 kDa (LOC103864856), mRNA ; Similar to OL3 Oleosin 14.9 kDa (Arabidopsis thaliana)::Bra035756:0-205  MKTFSTRGTDKQRGLTSFLPISLIHISSLLTPNILLSNLPLLSSSIFVSERSQQESMANQTRTHQDVIVRDSRSTLDRDHPKTGAQMVKVATGVAAGGSLLVLSGLTLAGTVIALAVATPLLIIFSPVLVPAVITVVLIITGFLASGGFGIAAITAFSWLYRHMTGSGSDQKIESARMKVGSRGYDTKSGQHNIGVHQQHQQAAS |
| Bra032113 | 166 | 18,301.4 Da | 100% identity with Bra032113 | 19% | >Bra032113 Brassica oleracea HDEM genome, scaffold: C8 ; Brassica rapa genome, scaffold: A08 ; PREDICTED: Brassica napus major oleosin NAP-II-like (LOC106419670), mRNA ; PREDICTED: Brassica napus major oleosin NAP-II-like (LOC111208674), mRNA ; PREDICTED: Brassica oleracea var. oleracea major oleosin NAP-II-like (LOC106319905), mRNA ; PREDICTED: Brassica rapa oleosin 16 kDa-like (LOC103832967), mRNA ; Protein of unknown function::Bra032113:0-166  MADRTSPSHIQQRPYGSPIAPPRGNNINHPIASFLRQLQSQSPEHSRQRFGLLAFFISGGILLLLTGITVTAFVLGFIAFLPIIIISSPIWIPLFLLVTGFLSVAGFLFSTAIVMSWMYRYFKGMHPVGSEQVDYARSRIYDTAAHVKDYAGGYFHGKLKDAAPGA |
| Identified oil-body proteins | | | | | |
| Bra036039 | 264 | 30,002.6 Da | 100% identity with Bra036039 | 35% | >Bra036039 PREDICTED: Brassica napus oil body-associated protein 1A (BNAA10G03690D), mRNA ; PREDICTED: Brassica napus oil body-associated protein 1A (BNAC05G03600D), mRNA ; PREDICTED: Brassica oleracea var. oleracea uncharacterised LOC106294119 (LOC106294119), mRNA ; PREDICTED: Brassica rapa oil body-associated protein 1A (LOC103844119), mRNA ; Protein of unknown function::Bra036039:0-264  MFFFTIRLNNSRDREGSRESICAMEKAVHLSTKAGPEVPGEPTKMGTSMVDSAAAAVQSFTPINQIHQHLCAFHFYAYDMTRQVEAHHFCGHINEDMRQCLIYDGPDANARLIGLEYIVSEKLFMTLPDDEKKLWHTHEWEVKGGFLFMPGVPEPIQRQDLEIVAKTYGKVYHFWQVDLGHELPIGLPNIMMAVTRDGQLYPEMVKETEKKFGISIDKERESRAYMTGPDHGIHPLANGGGKGLKLEMREVDIKPVESVPRVFV |
| Bra036099 | 562 | 27,737.6 Da | 100% identity with Bra036099 | 56% | >Bra036099 PREDICTED: Brassica napus putative 1-phosphatidylinositol-3-phosphate 5-kinase FAB1D (LOC106371146), mRNA ; Similar to FAB1D Putative 1-phosphatidylinositol-3-phosphate 5-kinase FAB1D (Arabidopsis thaliana)::Bra036099:0-562  MEEAAERLRQFLLTSANSLGIKDDWIIPCADLLEIVAKERDGLHNYIKFKCVATSDSPSLFYGLVFTKAPLDENMPALIIPTNLLVWEGSLDMDKEDKSAVSETYTLLDQYPVNVVMVEGSISEEYKGYFLSREITAVQNMKRRLQRYPSLGYSCTALRCEKTIESLNDPINEKPLMFLDGCGSLTILLKGSSTAELKLMKRMLKTGYNQFRNELLSSDYFLVALPPSKIIPWEMDGGQDEVVTIREEEVSSYIAYSLQQVHDESSESRPKSSVFCQHRDSFHELRSKCNMTESQYISSLSRCDTWEAKGGKSGALFAKSRDTRLIIKEINQAEFESFAKFGPMYFEYMKEANKTFLTKIYGVYKVTLGQAKFLMVMENLNFDRRIAMQYDLKGLVHGRLAPDSAQVRLDQNFLNDMKRLRLHLNPYLKQDLQTVIRNDTAFLSGINVMDYSLLVGVDMENHELVCGIIDYLAPYSLKKKLETVGKSLLLLGKETRPTVIRPSEYKKRFVDFMVKQFLGEIDLRTKVKELEQQLRKERDARLDAEKRLEELNKKKPIRSRSF |

List of protein sequences of identified vicilin, oleosin and oil-body proteins in *Brassica rapa* R-o-18 seed samples using liquid chromatography–mass spectrometry (LC-MS/MS) analysis.

**S6 Table.** Three dimensional models of vicilin candidate proteins.

| 3D model for *Brassica rapa* R-o-18 | Template | Seqence Identity | GMQE | QMEAN | Oligo-State |
| --- | --- | --- | --- | --- | --- |
| *Brassica rapa* R-o-18 vicilin-1 |  |  |  |  |  |
| 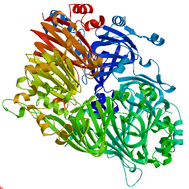 | 3smh.1.A (Allergen Ara h 1, clone P41B) | 34.51% | 0.57 | -1.57 | Homo-trimer (matching prediction) |
| 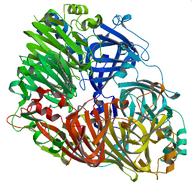 | 2ea7.1.A (Adzuki Bean 7S globulin-1) | 34.55% | 0.58 | -2.18 | Homo-trimer (matching prediction) |
| 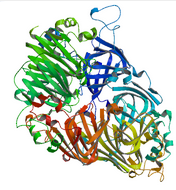 | 4lej.1 (Korean pine vicilin) | 30.38% | 0.50 | -2.35 | Homo-trimer (matching prediction) |
| 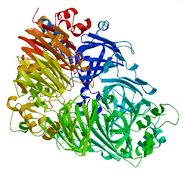 | 5e1r.1.A (pecan 7S vicilin) | 37.32% | 0.56 | -2.13 | Homo-trimer (matching prediction) |
| Predicted vicilin-2, *Brassica rapa* R-o-18 |  |  |  |  |  |
| 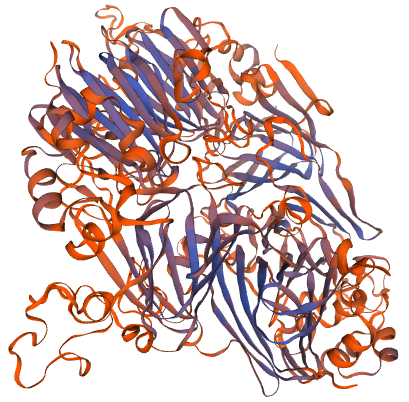 | 4lej.1.A (Korean pine vicilin) | 24.59% | 0.51 | -2.96 | Homo-trimer (matching prediction) |
| 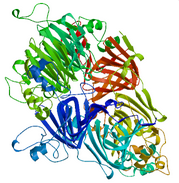 | 2ea7.1.A (Adzuki Bean 7S globulin-1) | 20.22% | 0.51 | -3.99 | Homo-trimer (matching prediction) |
| 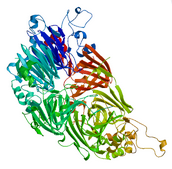 | 3smh.1.A (Allergen Ara h 1, clone P41B) | 21.22% | 0.49 | -4.49 | Homo-trimer (matching prediction) |
| 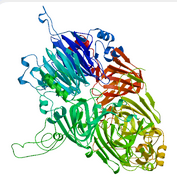 | 5e1r.1.A (pecan 7S vicilin) | 22.65% | 0.49 | -4.46 | Homo-trimer (matching prediction) |
| Predicted vicilin-3, *Brassica rapa* R-o-18 |  |  |  |  |  |
| 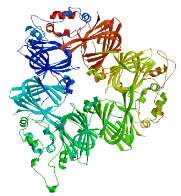 | 3smh.1.A (Allergen Ara h 1, clone P41B) | 21.19% | 0.41 | -3.52 | Homo-trimer (matching prediction) |
| 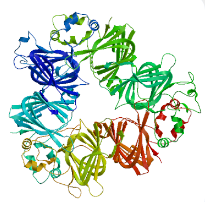 | 4lej.1.A (Korean pine vicilin) | 23.37% | 0.40 | -4.68 | Homo-trimer (matching prediction) |
| 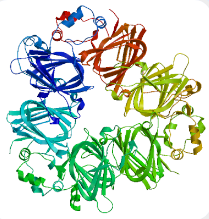 | 5e1r.1.A (pecan 7S vicilin) | 21.62% | 0.40 | -4.97 | Homo-trimer (matching prediction) |
| Predicted vicilin-4, *Brassica rapa* R-o-18 |  |  |  |  |  |
| 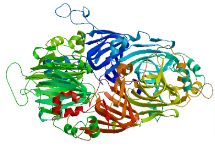 | 4lej.1.A (Korean pine vicilin) | 22.99% | 0.46 | -3.88 | Homo-trimer (matching prediction) |
| 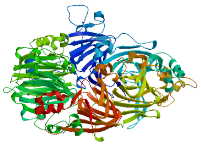 | 2ea7.1.A (Adzuki Bean 7S globulin-1) | 22.04% | 0.48 | -3.64 | Homo-trimer (matching prediction) |
| 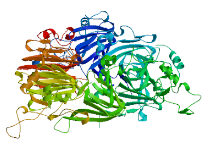 | 3smh.1.A (Allergen Ara h 1, clone P41B) | 25.54% | 0.42 | -4.44 | Homo-trimer (matching prediction) |
| 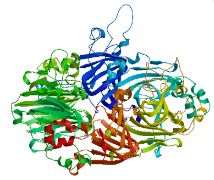 | 4lej.1 (Korean pine vicilin) | 27.65% | 0.41 | -5.17 | Homo-trimer (matching prediction) |

| Predicted vicilin-5, *Brassica rapa* R-o-18 |  |  |  |  |  |
| --- | --- | --- | --- | --- | --- |
| 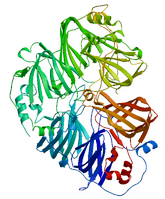 | 3smh.1.A (Allergen Ara h 1, clone P41B) | 29.11% | 0.24 | -3.78 | Homo-trimer (matching prediction) |
| 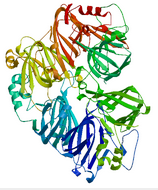 | 2ea7.1.A (Adzuki Bean 7S globulin-1) | 27.10% | 0.26 | -4.96 | Homo-trimer (matching prediction) |
| 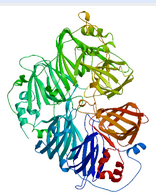 | 3s7i.1 (Major Peanut Allergen Ara h 1) | 29.45% | 0.23 | -4.47 | Homo-trimer (matching prediction) |
| 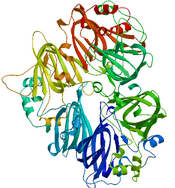 | 4lej.1 (Korean pine vicilin) | 30.45% | 0.26 | -5.08 | Homo-trimer (matching prediction) |

Three dimensional models of vicilin candidate proteins identified in the seed samples using liquid chromatography–mass spectrometry (LC/MS) analysis. Analysis found in at least 3 out of 4 biological replicates with at least 2 unique peptides and a p value of < 0.05 using *Brassica rapa* R-o-18 database. The candidate proteins were identified according to the method described in (Rahman et al., 2020a). The three dimensional models are cyphered using the automated SWISS-MODEL server homology modelling pipeline. The GMQE (Global Model Quality Estimation) was based on the QSQE (Quaternary Structure Quality Estimation) while local estimates of the model quality based on the QMEAN (Qualitative Model Energy Analysis) scoring function, both were used for the model rankings. The sequence identity represents target–template sequence identity.

**S7 Table.** List of protein sequences *Arabidopsis thaliana* oleosins representing four different lineages.

| Lineages | Representing *Arabidopsis thaliana* oleosin | Molecular weight | Sequences |
| --- | --- | --- | --- |
|  |  |  |  |
| U | AtOLE5 | 169 | >tr\|Q9M9A1\|Q9M9A1_ARATH F27J15.22 OS=Arabidopsis thaliana OX=3702 GN=At1g48990 PE=1 SV=1 MADRTNPSSHTQQRPIYNSTTVPRSNTTTNHPLSSLLRQLLQSQSPNHSGQLFGFLAFFI  SGGILLLLTGITVTAFVLGFIAFLPLIIISSPIWIPLFLIVTGFLSLAGLILATGAVVSW  LYRYFKGMHPLRSDQVDYARSRIHDTAAHVKDYAGGYFHGTLKDAAPGA |
| SL | AtOLE4 | 191 | >sp\|Q42431\|OLEO4_ARATH Oleosin 20.3 kDa OS=Arabidopsis thaliana OX=3702 GN=OL2 PE=2 SV=1 MANVDRDRRVHVDRTDKRVHQPNYEDDVGFGGYGGYGAGSDYKSRGPSTNQILALIAGVP  IGGTLLTLAGLTLAGSVIGLLVSIPLFLLFSPVIVPAALTIGLAVTGILASGLFGLTGLS  SVSWVLNYLRGTSDTVPEQLDYAKRRMADAVGYAGMKGKEMGQYVQDKAHEARETEFMTE  THEPGKARRGS |
| SH | AtOLEO6 | 149 | >tr\|Q494N8\|Q494N8_ARATH Oleosin OS=Arabidopsis thaliana OX=3702 GN=At2g25890 PE=1 SV=1  MADHQQHQQQQQPIMRSLHESSPSTRQIVRFVTAATIGLSLLVLSGLTLTGTVIGLIVAT  PLMVLFSPVLVPAVITIGLLTMGFLFSGGCGVAAATALTWIYKYVTGKHPMGADKVDYAR  MRIAEKAKELGHYTHSQPQQTHQTTTTTH |
| T | AtOLE13 | 106 | >tr\|Q42574\|Q42574_ARATH Oleosin OS=Arabidopsis thaliana OX=3702 GN=ATGRP19 PE=1 SV=1 MFEIIQAVFSAGVALALLTFAGITLGGSVVACIISTPLFVIFSPVLVPATIATTLLASGF  TASGSFGATAFTILSWLYKKRTGRDLPKIPGLTPPAPASNPAGSGV |

Adapted from (Chen et al., 2019).

**S8 Table.** Status of *B. rapa* R-o-18 putative napins corresponding to the identified proteins.

| Sl | *Brassica rapa* | Protein name | Length | Status of putative napins corresponding to the identified proteins using LC-MS/MS |
| --- | --- | --- | --- | --- |
| R-o-18 | | | | |
| 1 | Candidate napin 1 | Bra041165 | 178 | Identified in LC-MS/MS data.  BraA03000889: 100% cov and 87.0% pid,  BraA01001883: 100% cov and 93.9% pid. |
| 2 | Candidate napin 2 | Bra022319 | 166 | Bra041165: 88.2 % cov and 51.3% pid,  BraA03000889: 94.6 % cov and 47.7% pid,  BraA01001883: 94.0 % cov and 94.0% pid. |
| 3 | Candidate napin 3 | Bra022317 | 135 | Bra041165: 74.2 % cov and 65.7% pid,  BraA03000889: 98.5 % cov and 49.7% pid,  BraA01001883: 97.0 % cov and 50.0% pid. |
| 4 | Candidate napin 4 | Bra001100 | 121 | Bra041165: 68.0 % cov and 62.3% pid,  BraA03000889: 100 % cov and 41.3% pid,  BraA01001883: 99.2 % cov and 42.5% pid. |
| 5 | Candidate napin 5 | Bra029497 | 546 | Bra041165: 99.4 % cov and 29.6% pid,  BraA03000889: 98.4 % cov and 30.3% pid,  BraA01001883: 99.4 % cov and 30.9% pid. |
| Chiifu | | | | |
| 6 | Candidate napin 1 | BraA03005709 | 173 | Bra041165: 95.5 % cov and 71.3% pid,  BraA03000889: 92.9 % cov and 67.7% pid,  BraA01001883: 94.4 % cov and 69.4% pid. |
| 7 | Candidate napin 2 | BraA01002186 | 179 | Bra041165: 100 % cov and 99.4% pid, indicating identified in LC-MS/MS data.  BraA03000889: 97.3 % cov and 89.1% pid,  BraA01001883: 99.4 % cov and 94.4% pid. |
| 8 | Candidate napin 3 | BraA01001885 | 180 | Bra041165: 100 % cov and 94.4% pid,  BraA03000889: 97.8 % cov and 92.9% pid,  BraA01001883: 100 % cov and 96.7% pid indicating identified in LC-MS/MS data. |
| 9 | Candidate napin 4 | BraA01001883 | 180 | Identified in LC-MS/MS data,  Bra041165: 100 % cov and 93.9% pid,  BraA03000889: 97.8 % cov and 92.9% pid. |
| 10 | Candidate napin 5 | BraA01001886 | 179 | Bra041165: 99.4 % cov and 93.3% pid,  BraA03000889: 97.3 % cov and 92.9% pid,  BraA01001883: 99.4 % cov and 95.6% pid  indicating identified in LC-MS/MS data. |
| 11 | Candidate napin 6 | BraA01001887 | 182 | Bra041165: 99.4 % cov and 90.7% pid,  BraA03000889: 98.4 % cov and 92.4% pid,  BraA01001883: 99.4% cov and 94.5% pid. |
| 12 | Candidate napin 7 | BraA03005713 | 158 | Bra041165: 88.2 % cov and 53.6% pid,  BraA03000889: 85.3 % cov and 50.0% pid,  BraA01001883: 87.2 % cov and 52.5% pid. |
| 13 | Candidate napin 8 | BraA03000889 | 184 | Identified in LC-MS/MS data.  Bra041165: 100% cov and 87.0% pid,  BraA01001883: 100 % cov and 92.4% pid. |
| 14 | Candidate napin 9 | BraA08002165 | 171 | Bra041165: 94.9 % cov and 64.4% pid,  BraA03000889: 92.4 % cov and 57.8% pid,  BraA01001883: 93.9 % cov and 62.1% pid. |
| 15 | Candidate napin 10 | BraA03000890 | 76 | Bra041165: 42.7 % cov and 94.7% pid,  BraA03000889:41.3 % cov and 98.7% pid,  BraA01001883: 42.2 % cov and 94.7% pid. |
| 16 | Candidate napin 11 | BraA01001889 | 325 | Bra041165: 99.4 % cov and 48.2% pid,  BraA03000889: 98.9 % cov and 49.2% pid,  BraA01001883: 99.4 % cov and 50.3% pid. |

Status of *B. rapa* putative napins characterised in (Rahman, Baten, et al., 2020) corresponding to the identified proteins using LC-MS/MS analysis in *B. rapa* R-o-18 seeds. Bra041165 (length 178), BraA03000889 (length 184) and BraA01001883 (length 180) are the identified napin in *B. rapa* R-o-18 seed using LC-MS/MS analysis. Cov=coverage, pid=percent identity.The coverage and percent identity among the putative napins characterised in (Rahman, Baten, et al., 2020) and the napin sequences identified in *B. rapa* R-o-18 seeds using LC-MS/MS analysis (S4 Table) were determined using Clustal Omega (https://www.ebi.ac.uk/Tools/msa/clustalo/) (Madeira et al., 2019) and MView (Brown et al., 1998) tools. The proteins that had complete match in LC-MS/MS analysis are marked as “Identified in LC-MS/MS data” and those which had over 95.0%pid are referred as “indicating identified in LC-MS/MS data”.

**S9 Table.** Sequence analysis outcome of the identified 7S globulin-like vicilins.

| Amino acid | Bra014536 | Bra025745 | Bra033857 | Bra003060 | Bra022682 | Side Chain  Acidity / Basicity | % count |
| --- | --- | --- | --- | --- | --- | --- | --- |
| A=Ala | 26 | 22 | 32 | 27 | 43 | Neutral | 5.584512 |
| C=Cys | 3 | 4 | 10 | 10 | 8 | Neutral | 1.303053 |
| D=Asp | 16 | 18 | 26 | 18 | 21 | Acidic | 3.685778 |
| E=Glu | 33 | 35 | 51 | 52 | 143 | Acidic | 11.69025 |
| F=Phe | 47 | 28 | 32 | 26 | 25 | Neutral | 5.882353 |
| G=Gly | 39 | 47 | 35 | 32 | 25 | Neutral | 6.626955 |
| H=His | 19 | 10 | 6 | 6 | 12 | Basic (weakly) | 1.973194 |
| I=Ile | 19 | 29 | 22 | 25 | 17 | Neutral | 4.169769 |
| K=Lys | 26 | 21 | 48 | 33 | 53 | Basic (weakly) | 6.738645 |
| L=Leu | 33 | 36 | 46 | 51 | 45 | Basic | 7.855547 |
| M=Met | 8 | 20 | 17 | 15 | 16 | Neutral | 2.829486 |
| N=Asn | 25 | 17 | 22 | 15 | 16 | Neutral | 3.536858 |
| P=Pro | 29 | 25 | 50 | 29 | 22 | Neutral | 5.770663 |
| Q=Gln | 31 | 16 | 37 | 20 | 18 | Neutral | 4.54207 |
| R=Arg | 18 | 25 | 19 | 22 | 92 | Basic (strong) | 6.552494 |
| S=Ser | 36 | 37 | 46 | 48 | 32 | Neutral | 7.408786 |
| T=Thr | 29 | 24 | 27 | 25 | 19 | Neutral | 4.61653 |
| V=Val | 35 | 28 | 35 | 33 | 37 | Neutral | 6.254654 |
| W=Trp | 4 | 7 | 9 | 8 | 4 | Neutral | 1.191363 |
| Y=Tyr | 10 | 15 | 6 | 8 | 9 | Neutral | 1.787044 |
| Residue | 486 | 464 | 576 | 502 | 657 | - | 100 |
| Molecular weight | 54657.59 | 51927.33 | 64442.47 | 56141.32 | 78666.16 | - | - |
| Isoelectric point | 6.9 | 6.19 | 5.2668 | 4.8806 | 5.3778 | - | - |

Amino acid composition and isoelectric point of 7S globulin type vicilins were calculated using (https://www.ebi.ac.uk/Tools/seqstats/emboss_pepstats/, 30/08/2020). % amino acid count is calculated from total number of amino acids of five vicilins. Side chain acidity / basicity is from (http://www.thinkpeptides.com/aminoacidproperties.html, 30/08/2020).
